# Determining lineage-specific bacterial growth curves with a novel approach based on amplicon reads normalization using internal standard (ARNIS)

**DOI:** 10.1038/s41396-018-0213-y

**Published:** 2018-07-06

**Authors:** Kasia Piwosz, Tanja Shabarova, Jürgen Tomasch, Karel Šimek, Karel Kopejtka, Silke Kahl, Dietmar H. Pieper, Michal Koblížek

**Affiliations:** 1 Center Algatech, Institute of Microbiology CAS, Novohradská 237, 37981 Třeboň, Czech Republic; 2 Biology Centre CAS, Institute of Hydrobiology, Na Sádkách 7, 37005 Česke Budějovice, Czech Republic; 3 Helmholtz Centre for Infection Research, 38124 Braunschweig, Germany; 4 Faculty of Science, University of South Bohemia in České Budějovice, CZ-37005 České Budějovice, Czech Republic

## Abstract

The growth rate is a fundamental characteristic of bacterial species, determining its contributions to the microbial community and carbon flow. High-throughput sequencing can reveal bacterial diversity, but its quantitative inaccuracy precludes estimation of abundances and growth rates from the read numbers. Here, we overcame this limitation by normalizing Illumina-derived amplicon reads using an internal standard: a constant amount of *Escherichia coli* cells added to samples just before biomass collection. This approach made it possible to reconstruct growth curves for 319 individual OTUs during the grazer-removal experiment conducted in a freshwater reservoir Římov. The high resolution data signalize significant functional heterogeneity inside the commonly investigated bacterial groups. For instance, many Actinobacterial phylotypes, a group considered to harbor slow-growing defense specialists, grew rapidly upon grazers’ removal, demonstrating their considerable importance in carbon flow through food webs, while most Verrucomicrobial phylotypes were particle associated. Such differences indicate distinct life strategies and roles in food webs of specific bacterial phylotypes and groups. The impact of grazers on the specific growth rate distributions supports the hypothesis that bacterivory reduces competition and allows existence of diverse bacterial communities. It suggests that the community changes were driven mainly by abundant, fast, or moderately growing, and not by rare fast growing, phylotypes. We believe amplicon read normalization using internal standard (ARNIS) can shed new light on in situ growth dynamics of both abundant and rare bacteria.

## Introduction

Growth is one of the main characteristics of all living organisms. In microbial ecology, growth provides an ultimate measure of metabolic activity of a particular organism, and its contribution to the fluxes of matter and energy [1, 2]. The type of growth response also reflects the physiological limitations of a particular organism, as well as their ecology or position in the microbial food webs.

The growth rate can be directly determined in microbial cultures as the relative change of biomass (frequently approximated by microscopy counts) per unit of time [3, 4]. Despite the fact that laboratory experiments provide invaluable information on bacterial growth and physiology [5, 6], they cannot be directly applied in natural planktonic communities, where a fraction of the biomass is constantly removed at lineage-specific rates by protozoan grazing, viral lysis, or UV damage [7, 8]. Therefore, in situ bacterial specific growth rates are typically determined using manipulation experiments, in which mortality is reduced using pre-filtration and/or dilution, and the growth is followed by microscopy [9]. The response of individual bacterial groups can be determined using fluorescence in situ hybridization (FISH) technique [10]. This approach has largely expanded our knowledge about the activity and ecology of the main bacterial groups [1, 7]. However, this labor-intensive approach allows only for a handful of main phylotypes to be followed in a single study.

The development of high-throughput sequencing technologies revolutionized our ability to study natural microbial communities at high taxonomic resolution [11–15]. Currently, the most common approach to study bacterial diversity and community structure is 16S rRNA amplicon sequencing, and a number of studies have provided an immense amount of information on microbial diversity in many habitats [11, 16–21].

Unfortunately, amplicon data cannot provide truly quantitative information on the abundance of individual lineages. The reasons for low quantitative accuracy of sequencing methods (whether high throughput or classical) in translating the read numbers to bacterial phylotype-specific cell numbers, are biases connected to the variable number of copies of the rRNA genes in different bacterial species, and sample processing: DNA extraction, amplification, and sequencing [22–24]. In analytical methods, biases resulting from sample processing are often accounted for with an internal standard [25]: a known amount of an easily quantifiable standard substance is added to every sample, which makes it possible to correct for the losses during the sample extraction and handling. Normalization on internal standards can also correct certain biases introduced by the analytical procedure and instrumentation. Genomic DNA and synthetic spike-in standards have been proposed as internal standards for environmental metagenomic studies to assess the post DNA extraction biases [26, 27], but the initial steps of the protocol, i.e., collection of the biomass by filtration, storage of the samples, cell lysis, and DNA extraction efficiency, are not accounted for in such approaches.

Building on these ideas, we tested whether an internal standard can be used to account for the biases connected with high-throughput sequencing. The use of an internal standard would allow a relative comparison of the normalized read numbers among the collected samples and reconstruction of growth response curves for each individual operational taxonomic unit (OTU). Our aim was to provide information about growth dynamics of individual bacterial phylotypes present in a natural freshwater community. This high taxonomic resolution should allow us to enhance our comprehension of complex responses of microbial communities exposed to different levels of grazing pressure.

## Material and methods

### Preparation of the internal standard

*Escherichia coli* strain K-12 was grown in LB medium [28] for 20 h at 37 °C. The cells were harvested from 2 mL of culture by centrifugation (5 min, 4000×*g*), re-suspended in 0.6 mL of phosphate-buffered saline (pH 7.4), and fixed with 1.4 mL absolute ethanol.

### Mock communities

We prepared five artificial communities with different amounts of four bacterial species from distinct phyla that have been demonstrated to be present and quantitatively important in freshwater communities [29]: *Rhodoluna lacicola* (Actinobacteria), *Sphingomonas* sp. AAP5 strain (Alphaproteobacteria), *Gemmatimonas phototrophica* (Gemmatimonadetes), and *Flavobacterium* sp. (Bacteroidetes). *Sphingomonas* sp. and *Flavobacterium* sp. were grown in R2A medium [30], *R*. *lacicola* in NSY medium [31] at 22 °C in 12:12 light:dark cycle. *G. phototrophica* was grown on a solid R2A medium at 10% oxygen tension. The cell abundance in pure cultures was estimated by microscopy (see below), and the cultures were mixed in different proportions in 300 mL of inorganic basal medium [31], in order to mimic the anticipated responses of natural bacterial communities. The abundance of all bacteria in mock communities varied between 0.4 and 5.1 × 10^6^ cells mL^−1^. The final abundance of each species in each mock community is given in Supplementary Table 1.

Two milliliters of each mock community was prepared for microscopic and catalyzed reporter deposition-FISH (CARD-FISH) evaluations as described below. Seventy-five microliters of the internal standard (representing a total of ca. 7.5 × 10^7^ *E. coli* cells, equivalent to 2.5 × 10^5^ cells mL^−1^) were added to the remaining volume of each mock community, subsequently divided into three aliquots of 95 mL, and filtered onto polycarbonate filters (0.2 µm pore size, 47 mm diameter, Whatman). The filters were stored at −20 °C until the DNA extraction within 1 month. The samples were processed for Illumina sequencing as described below.

### Study site and experimental design

The experiment was conducted using water collected from a freshwater reservoir Římov in the Czech Republic, 250 m from the dam (for details, see Šimek et al. [32]). Thirty liters of water was collected with a 2-L Friedinger sampler from a depth of 0.5 m on 14 September 2015 into a clean plastic container. The following background chemical parameters were measured at time zero: dissolved organic carbon (DOC), total phosphorus (TP), dissolved reactive phosphorus (DRP as PO_4_-P), and chlorophyll *a* concentration (Chl-*a*). DOC was analyzed from the samples filtered through glass-fiber filters of 0.4 µm pore size (GF-5, Macherey-Nagel) with a total organic carbon (TOC) 5000A analyzer (Shimadzu). TP was determined according to Kopáček and Hejzlar [33], DRP according to Murphy and Riley [34], and Chl-*a* according to Lorenzen [35].

The experimental design was based on the setup described in detail by Šimek et al. [32]. Fifteen liters of the collected water was filtered through 1-μm pore-size filters (147 mm diameter, Osmonic) to remove all bacterivores (the bacterivore-free treatment), allowing the determination of net bacterioplankton specific growth rates. Filtration through 1-μm removed only 2.4% of free-living bacteria. The presence of bacterivores (HNF and ciliates) was monitored in both treatments (see below). The unfiltered water represented control treatments with all bacterivores present. Both treatments were prepared in triplicates and incubated in sterilized 2-L glass bottles for 69 h at in situ temperature (17.2 °C) in the dark. The initial concentrations of measured background chemical parameters in the original sample at *T*_0_ of the experiment were as follows: DOC—5.26 mg L^−1^; TP—19 µg L^−1^; DRP—1.62 µg L^−1^; Chl-*a*—36.3 µg L^−1^.

Subsamples (∼300 mL) were taken from each triplicate bottle after 0, 12, 21, 45, and 69 h of incubation to analyze bacterial abundance, and heterotrophic nanoflagellate (HNF) abundance and bacterial community composition (Illumina sequencing and CARD-FISH, see below). Twenty-five microliters of the internal standard was added to 250 ml of the sample for DNA extraction (a final abundance: 10^5^ *E. coli* cells mL^−1^, approx. 5% of natural bacterial community) that was immediately filtered onto 0.2-μm pore-size sterile polycarbonate filters, as described for the mock community experiments.

### Bacterial and HNF abundance

Samples were fixed with formaldehyde (2% final concentration), concentrated on 0.2-μm (bacteria) or 1-μm pore-size filters (HNF; Osmonic, 25 mm diameter), stained with DAPI (4′,6-diamidino-2-phenylindole; 0.1 μg mL^−1^ final concentration), and enumerated by epifluorescence microscopy (Olympus BX 60) [32].

### CARD-FISH

The CARD-FISH analysis was conducted as previously described [36]. Treatment time with achromopeptidase was optimized to 25 min to avoid overdigestion of bacterial cells [37]. For the mock communities, *R. lacicola* was targeted with the probe HGC69a [38], *Sphingomonas* sp. AAP5 with the probe Alf968 [39], and Flavobacterium sp. with the probe CF319a [40]. In the experimental setup, we used the HGC69a probe for hybridization of Actinobacteria, Ac1-852 probe for acI lineage of Actinobacteria [41], Bet42a probe for Betaproteobacteria [42], R-BT065 probe for genus *Limnohabitans* [32], and the CF968 probe for the phylum Bacteroidetes [43]. The list of all the oligonucleotides used (probes, competitors, and helpers) and the hybridization conditions is given in Supplementary Table 2. Carboxyfluorescein-labeled tyramides were used for the amplification step. Hybridized and amplified samples were counterstained with DAPI, and proportions of probe-positive bacteria were determined by inspecting 500–1000 DAPI stained cells (with larger numbers of cells inspected in samples with less abundant bacterial phyla) with epifluorescence microscopy (Olympus BX-53F) using UNWU, U-WB, and U-WG optical filter sets.

### DNA extraction, PCR, and sequencing

Filters were cut into pieces under sterile conditions, and DNA was isolated using phenol/chloroform/isoamyl alcohol extraction [44]. Precipitated DNA was re-suspended in 100 μL of PCR-clean water (Sigma) and further purified on a column using TIANquick Midi Purification Kit (TIANGEN). DNA concentrations were determined using a NanoDrop (Thermo Scientific), and samples were stored at −80 °C. The V5–V6 regions of 16S rRNA genes were amplified with 807F and 1050R primers [45] as described before [46], and sequenced on the Illumina MiSeq sequencer using a paired-end strategy with 250 bp single read length. Raw sequence files have been deposited at the ENA database (http://www.ebi.ac.uk/ena) under accession PRJEB24102.

### Bioinformatics analysis

The obtained raw sequence files were demultiplexed using the sabre tool (http://github.com/ucdavis-bioinformatics/sabre). Cutadapt was used for primer trimming [47]. VSearch was used for merging overlapping forward and reverse read pairs, removing chimeras, and calculating OTUs based on 97% similarity [48]. Taxonomic classification was performed using the online SINA classifier tool [49]. Proportion of reads was calculated as the number of reads of the particular OTU divided by the total number of reads in the sample.

### Normalization of the amplicon reads: calculating the ARNIS ratio

Amplicon reads normalization using internal standard (ARNIS) ratio and bacterial specific growth rates were calculated in R [50], using packages: dplyr [51], ggplot2 [52], and reshape2 [53].

### Statistical analysis

Specific growth rates were calculated based on changes in the cell number of the CARD-FISH-positive phylotypes and ARNIS values, and the proportion of reads were compared using *t*-test in SigmaPlot 13.0 (Systat Sofware, Inc.).

Distances between the samples, and between most abundant OTUs (>0.1% of reads), were calculated from Bray–Curtis indices using the vegdist function of the R-package vegan [54]. Samples and OTUs were clustered based on average distance using the hclust function. The heat map plot was created with the heatmap.2 function of the R-package gplots [55].

## Results

### Work flow for ARNIS

**Fig. 1:**
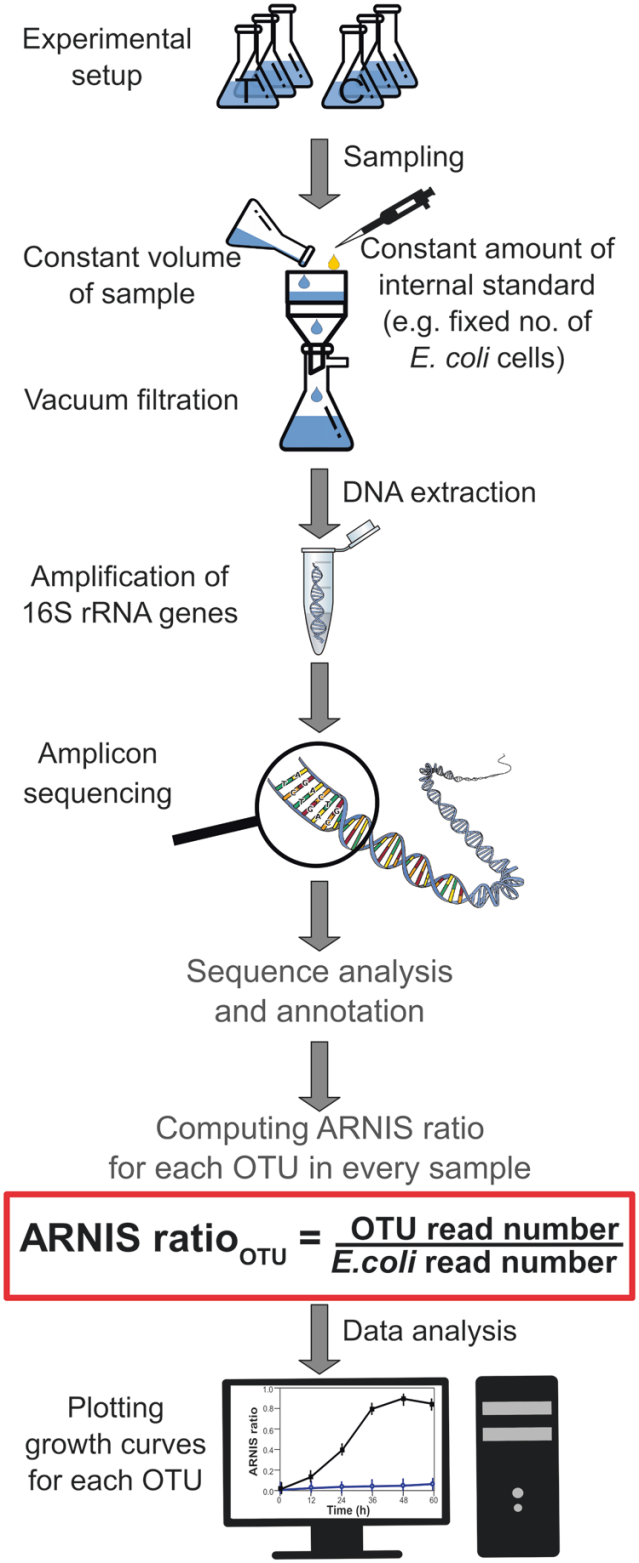
Schematic representation of sample processing and analysis for the ARNIS approach with internal standard for estimating microbial specific growth rates from high-throughput sequencing data. Upon collection of the samples, a constant number of *E. coli* cells (the internal standard) is spiked into a constant volume of each sample just prior to biomass collection on a filter. Subsequently, DNA is extracted, and the gene of interest (here 16S rRNA) is amplified and sequenced. After analysis of the sequencing data using a bioinformatics pipeline of choice, reads coming from the internal standard are extracted, and the amplicon reads of each OTU are normalized for each sample, namely the read number for a given OTU is divided by the read number originating from the internal standard in the sample (ARNIS ratio). The values of ARNIS ratio for a given OTU can be then analyzed and compared between the samples. Images used to create this figure are under creative commons license, and were downloaded from the NounProject (http://thenounproject.com) and the Pixabay (http://pixabay.com/)

To assess the bacterial specific growth rates for a high number of phylotypes, we propose to use an internal standard normalization, which allows to minimize the methodological bias associated with the amplicon sequencing. Figure 1 shows the key steps in the ARNIS workflow. Upon collection of samples, exactly the same amount of *E. coli* cells (the internal standard—an organism absent in the studied environment) was spiked into a constant volume of each sample just before the biomass was collected by filtration. After DNA extraction, PCR amplification of 16S rRNA gene fragments, sequencing and bioinformatic analysis, the amplicon reads of each OTU were normalized for each sample by dividing the read numbers for a given OTU by the read numbers originating from the internal standard in the sample (ARNIS ratio):

$${\mathrm{ARNIS}}\,{\mathrm{ratio}}_{{\rm OTU}} \\ = \frac{{{\mathrm{Number}}\,{\mathrm{of}}\,{\mathrm{reads}}\,{\mathrm{of}}\,{\mathrm{the}}\,{\mathrm{OTU}}\,{\mathrm{in}}\,{\mathrm{a}}\,{\mathrm{sample}}}}{{{\mathrm{Number}}\,{\mathrm{of}}\,{\mathrm{reads}}\,{\mathrm{affiliated}}\,{\mathrm{with}}\,{E}{.coli}\,{\mathrm{in}}\,{\mathrm{a}}\,{\mathrm{sample}}}}$$

### Concept verification using mock communities

**Fig. 2:**
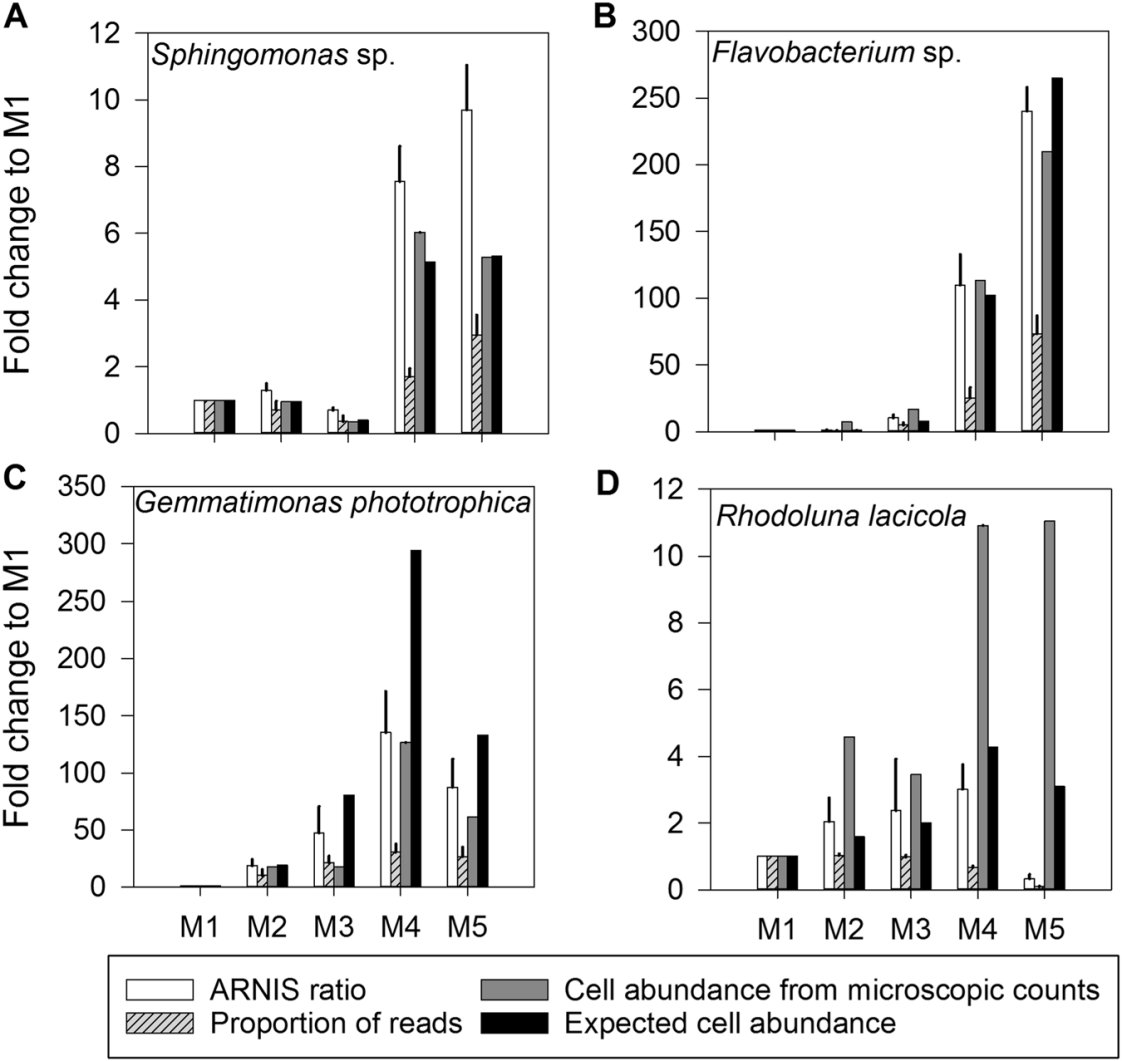
Fold-differences between mock communities for a given bacterial species estimated by ARNIS, proportions of reads, microscopic cell count, and expected cell abundance based on the volume of pure cultures added to the mock community. Error bars show standard deviation (available only for sequencing-based data). **a** *Sphingomonas* sp. strain AAP5, **b** *Flavobacterium* sp., **c** *Gemmatimonas phototrophica*, **d** *Rhodoluna lacicola*

The ARNIS approach was first tested using five artificial mock communities prepared by mixing known amounts of four cultured species representing different bacterial phyla (Supplementary Table 1). The theoretical differences in contribution of species between the communities were compared with the numbers analyzed by CARD-FISH, a proportion of reads specific for each OTU, and ARNIS ratios (Fig. 2). The agreement between the latter approaches, except for the proportion of reads, was excellent in case of *Sphingomonas* sp. AAP5 (Fig. 2a, fold-change between communities M5 and M3: ARNIS: 13.8, expected abundance: 13.2, CARD-FISH: 14.8; proportion of reads: 8.7) and *Flavobacterium* sp. (Fig. 2b, fold-change between communities M5 and M1: ARNIS: 238.4-fold, CARD-FISH: 209.9-fold, expected abundance: 265.2-fold difference; proportion of reads: 71.9). The fold-changes obtained by ARNIS and microscopy for *G. phototrophica* matched tightly (Fig. 2c, fold-change between communities M4 and M1: 129.6 and 126.7, respectively), less with the expected abundance (294.2), and poorly with the proportion of reads (29.4). The fold-change between communities M4 and M2 calculated for *R. lacicola* were 1.5 by ARNIS, 0.65 by proportion of reads, 2.4 by CARD-FISH, and 2.7 by expected abundance, but differences between cell and read numbers in community M5 were substantial (Fig. 2d). Nevertheless, the overall agreement between ARNIS and the abundance was satisfactory, in contrast to the numbers based on the not-normalized proportion of reads.

### Proof of concept—the manipulation experiment

**Fig. 3:**
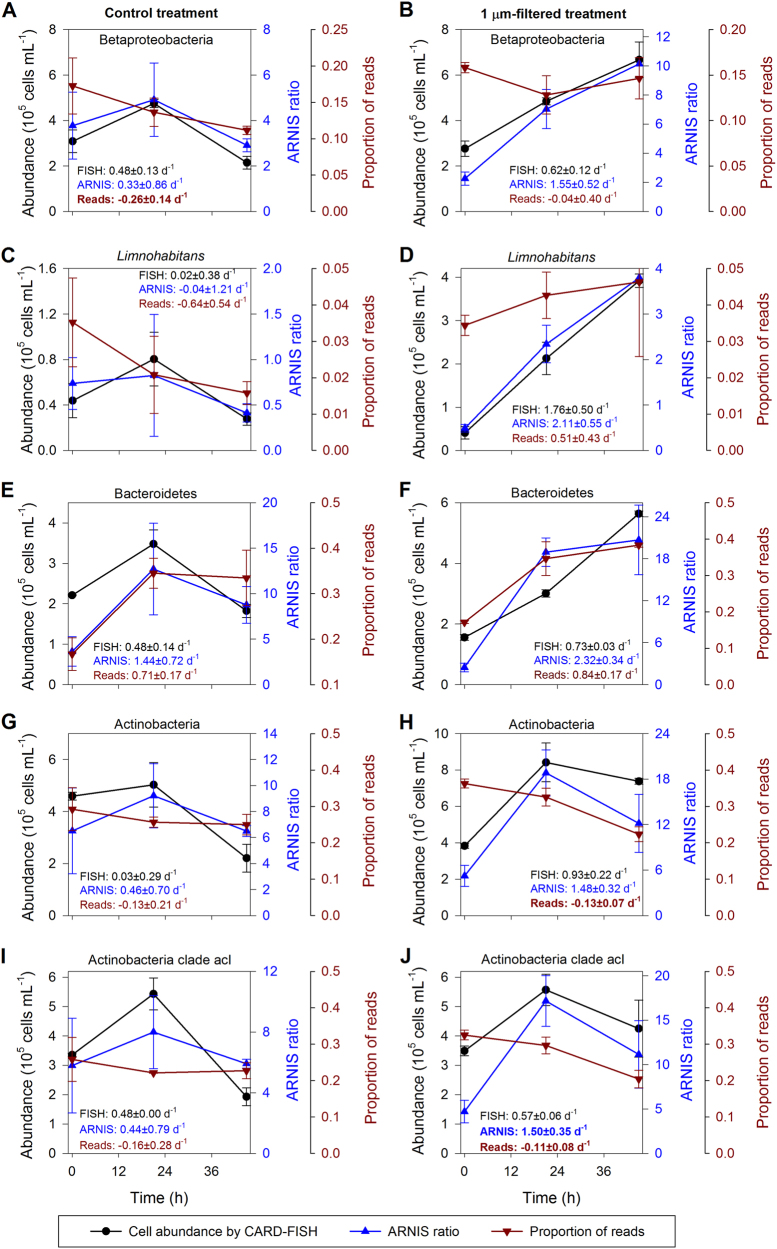
Comparison of time-course changes (0–45 h) of different bacterial groups estimated by CARD-FISH, ARNIS, and proportion of reads. GR: growth rate estimated for the time interval between 0–21 h, bold font indicates significant difference by *t*-test (*p* < 0.05). **a**, **b** Betaproteobacteria, **c**, **d** Betaproteobacteria subgroup detected with the R-BT065 probe (*Limnohabitans*), **e**, **f** Bacteroidetes, **g**, **h** Actinobacteria, **i**, **j** Actinobacteria clade acI

We subsequently verified the ARNIS approach using a grazer manipulation experiment based on the comparison of population dynamics of the same bacterial phyla in grazer-free versus whole-water control treatments (Fig. 3). The grazers were removed using 1 µm pore-size filters and the response of the bacterial community was followed over 69 h (for details, see Materials and Methods). The 16S rRNA amplicon sequences from the analyzed samples were assigned to 319 individual OTUs. We compared the ARNIS ratios with CARD-FISH counts of several phylogenetic groups to further validate our method (Fig. 3). The dynamics revealed with ARNIS ratios matching well with those done by CARD-FISH for all studied groups in both treatments: class Betaproteobacteria (Fig. 3a, b), including common freshwater *Limnohabitans* lineage (Fig. 3c, d), phyla Bacteroidetes (Fig. 3e, f) and Actinobacteria (Fig. 3g, h), including common in freshwaters acI lineage (Fig. 3i, j).

Bacterial specific growth rates for each OTU (*µ*_OTU_) were then estimated from fold-change between the calculated values of the ARNIS ratio at two different time points, following the equation:

$$\mu _{{\rm OTU}}\, = \,	{\rm ln}\left( {\frac{{{\rm ARNIS}\,{\rm ratio}\,{\rm for}\,{\rm the}\,{\rm OTU}\,{\rm at}\,{\rm time}\,t_N}}{{{\rm ARNIS}\,{\rm ratio}\,{\rm for}\,{\rm the}\,{\rm OTU}\,{\rm at}\,{\rm time}\,t_{\left( {N - 1} \right)}}}} \right)\\ 	\times \left( {t_N - t_{\left( {N - 1} \right)}} \right)^{ - 1}$$

The specific growth rates calculated using either FISH or ARNIS data did not differ significantly (*t*-test, *p* < 0.05). In contrast, the match between the CARD-FISH results and the simple proportion of reads was very poor, sometimes even showing the opposite trends (Fig. 3).

Altogether, the performed comparisons indicate that ARNIS should give decent estimates of bacterial specific growth rates in natural microbial communities at an unpreceded level of phylogenetic resolution.

### Types of bacterial response to the experimental grazer removal

**Fig. 4:**
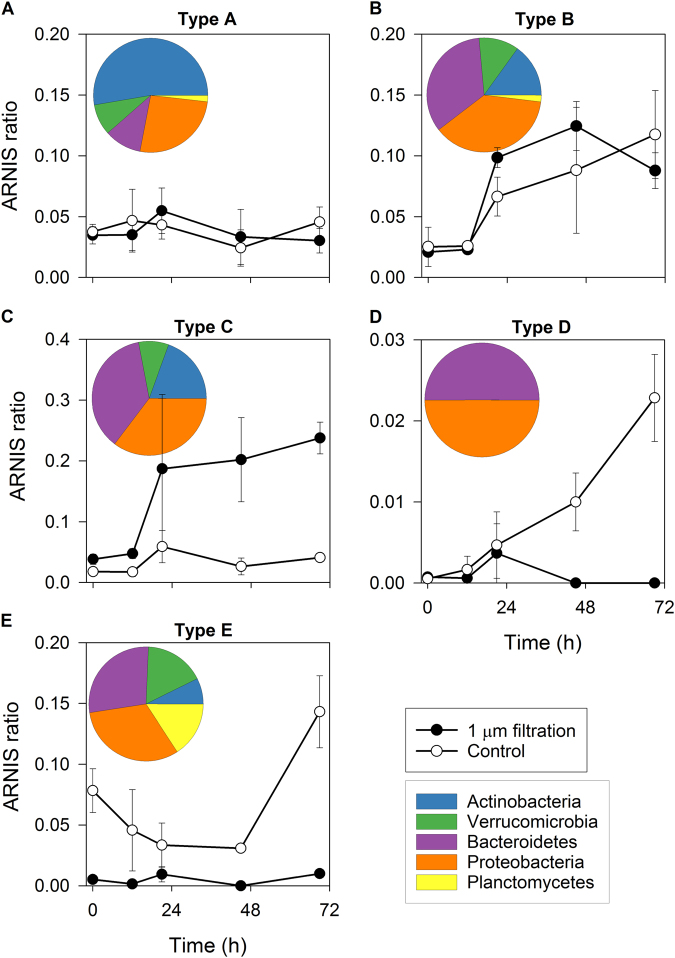
Types of bacterial responses to food web manipulations (bacterivore-free treatment versus control). **a** Type A response: no growth in both treatments (Actinobacteria, Microbacteriaceae); **b** Type B response: growth in both treatments (Bacteroidetes, Chitinophagaceae); **c** Type C response: growth only in the bacterivore-free treatments (Betaproteobacteria, *Limnohabitans*); **d** Type D response: growth only in the control (Deltaproteobacteria, *Peredibacter*); **e** Type E response: OTUs removed by the filtration and present only in the control treatment (Planctomycetes, OM190). Error bars show standard deviation from triplicates. Pie charts show contributions of the main bacterial phyla complementing the response types 1 to 5. Note different scales on Y-axes
- **Type B**: OTUs that grew in both control and the bacterivore-free treatments (Fig. 4b),
- **Type C**: OTUs that grew only in the bacterivore-free treatment (Fig. 4c),
- **Type D**: OTUs that grew only in the control treatment (Fig. 4d), and
- **Type E**: OTUs removed by the filtration (Fig. 4e).

**Fig. 5:**
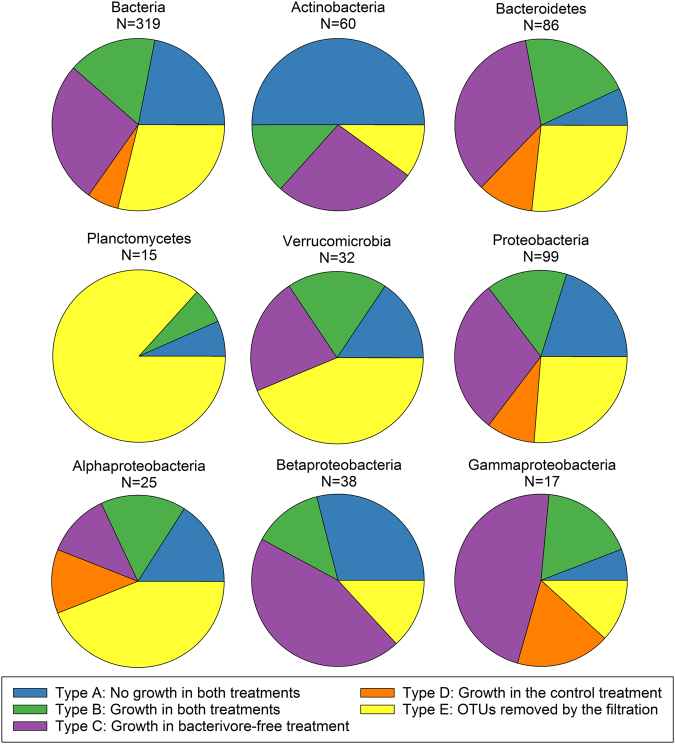
Distribution of OTUs showing different types of response to removal of bacterivores among bacterial phyla and proteobacterial classes. N: number of OTUs affiliated with a given phylum/class

**Fig. 6:**
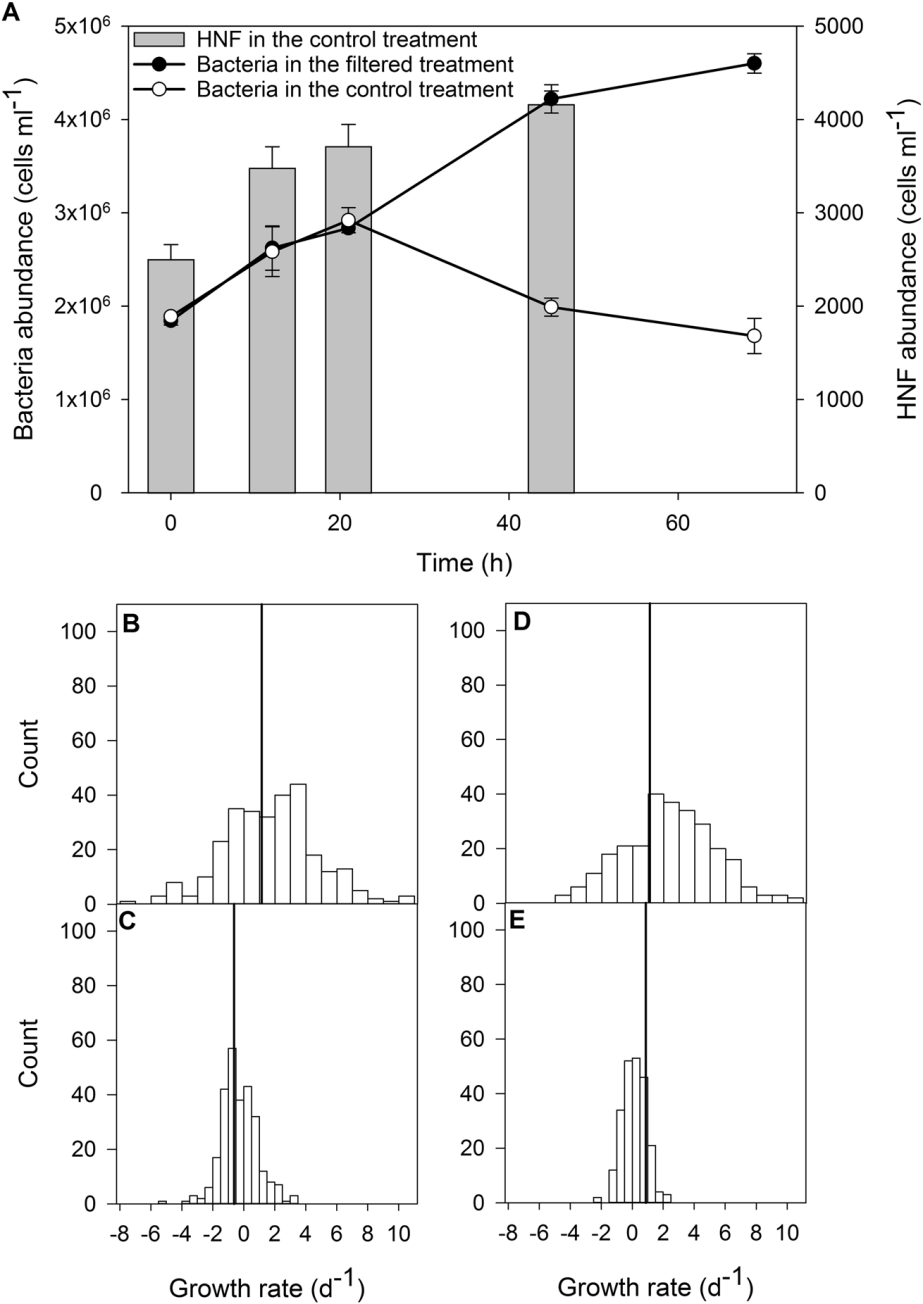
**a** Changes in abundance of bacteria and heterotrophic nanoflagellates (HNF) in the experimental treatments (average from triplicate treatments, error bars show standard deviation); **b** distribution of the initial specific growth rates (0–21 h) in the control treatment; **c** distribution of the late specific growth rates (21–69 h) in the control treatment; **d** distribution of the initial specific growth rates (0–21 h) in the bacterivore-free treatment; **e** distribution of the late specific growth rates (21–69 h) in the bacterivore-free treatment. The vertical line in **b**–**d** corresponds to the average specific growth rates calculated from changes in abundance of bacteria shown in **a**

The analyzed phylotypes responded in many different ways to grazer removal (Supplementary Table 3, Supplementary Figure 1) that could be generally classified into five basic groups:

- **Type A**: OTUs that did not grow in any of the treatments (Fig. 4a),

Growth of more than one-third of the OTUs was unaffected by the filtration (Fig. 5), which means they fell either into Type A (22%) or Type B (16%, Fig. 5); 27% OTUs were stimulated by the grazer removal (Type C), while less than 6% grew only in the control treatment (Type D). A remarkably high proportion of OTUs (29%) did not pass through 1-µm pore-size filter, being likely retained on its surface (Type E), which is interesting considering the fact that bacterial numbers did not differ between the treatments at *T*_0_ (Fig. 6a).

This general picture of bacterial responses differed at the resolution level of phyla and classes (Fig. 5, Supplementary File 1). Type A response was mostly observed among phylotypes affiliated with Actinobacteria (>50%), Proteobacteria, and Bacteroidetes (Fig. 4a insert, Fig. 5). Type B response was detected mainly within Proteobacteria, Bacteroidetes, Actinobacteria, and Verrucomicrobia (Fig. 4b insert), but it was not the dominant response type for any of these phyla (Fig. 5). Type C was observed for the same phyla like Type B (Fig. 4c insert), but it was much more common, especially among Bacteroidetes, Beta-, and Gammaproteobacteria (Fig. 5). In contrast, Type D was rather rare and was found only within Bacteroidetes and Proteobacteria (Fig. 4d insert, Fig. 5). Finally, Type E was detected among all main phyla (Fig. 4e insert), and was especially common in Planctomycetes, Verrucomicrobia, and Alphaproteobacteria (Fig. 5). Detailed analyses of the response patterns at a phylotype level can be found in Supplementary File 1 and Supplementary Table 3.

### Lineage-specific growth rates and whole community response to the experimental grazer removal

The main focus of our study was method verification focusing on the variability in specific growth rates among different bacterial phylotypes. The initial specific growth rates in both treatments were calculated for the time period 0–21 h, and then later for the time period 21–69 h. The dynamics in total bacterial abundance indicated a moderate grazing pressure in situ. The initial growth was similar in both treatments (1.14 ± 0.01 d^−1^ in the control and 1.12 ± 0.01 d^−1^ in the bacterivore-free treatment). Bacteria continued to grow in the bacterivore-free treatment at the rate of 0.88 ± 0.05 d^−1^ between 21 and 69 h. In the control treatment, they were grazed, yielding the negative rate −0.62 ± 0.07 d^−1^, which coincided with the increased abundance of HNF (Fig. 6a).

We observed clear differences in the distribution of specific growth rates of each phylotype between the bacterivore-free and control treatments. The initial specific growth rates in the filtered 1 µm treatment had normal distribution, and ranged from −4.1 (Burkholderiales) to 10.4 d^−1^ (Candidate division SR1, Fig. 6d). The other fastest growing OTUs (specific growth rates >1.3 d^−1^) were affiliated with Bacteroidetes (*Emticicia*), Firmicutes, Verrucomicrobia (*Prosthecobacter*), and Proteobacteria (OTUs affiliated with Comamonadaceae and *Legionella*), while the fastest vanishing (specific negative growth rates <−3.0) belonged to Actinobacteria (OTUs affiliated with clade acI, *Limnoluna*, and *Ilumatobacter*), Verrucomicrobia (Opitutae), unclassified Chloroflexi, and Proteobacteria (OTUs affiliated with Burkholderiales, *Duganella*, and Sorangiineae). In the second phase (after 21 h) of the filtered treatment, the distribution of the growth rates remained normal but the range narrowed and varied from −2.2 (Comamonadaceae) to 2.2 d^−1^ (*Gemmatimonas* Fig. 6e). The 10 fastest growing OTUs (specific growth rates >1.3 d^−1^) were affiliated with Actinobacteria (OTUs affiliated with clade acI, Microbacteriaceae, and *Ilumatobacter*), Chloroflexi (*Roseiflexus*), Gemmatimonadetes (Gemmatimonadaceae), Verrucomicrobia (OPB35 soil group), and Proteobacteria (OTUs affiliated with *Roseomonas* and *Variovorax*). The 10 OTUs with the highest mortality rate (<−1.3) belonged to Actinobacteria (OTUs affiliated with clade acI, Acidimicrobiales, Microbacteriaceae, *Planktoluna*, and *Ilumatobacter*), Firmicutes, and Proteobacteria (OTUs affiliated with Comamonadaceae and *Polynucleobacter*).

**Fig. 7:**
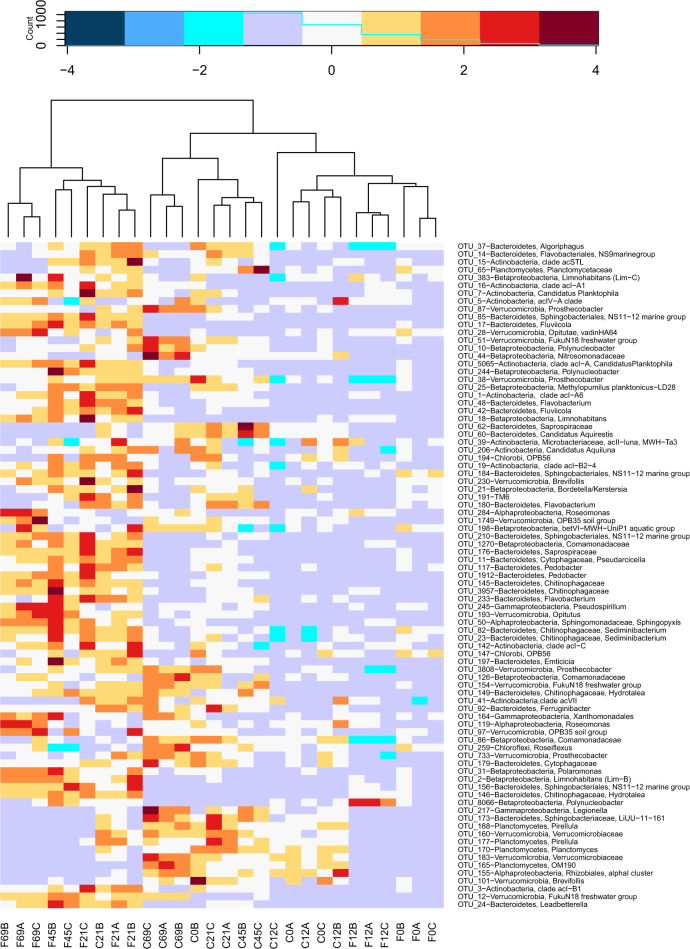
Heatmap showing the changes in the bacterial communities upon removal of bacterivores. Clustering of the samples based on Bray–Curtis distance matrix calculated on ARNIS data. Sample codes: F bacterivore-free treatment, C control treatment; number corresponds to the time of sample collection (hours), A, B, and C behind the number denote replicates

As could be expected from the differences in the specific growth rates of particular OTUs between the treatments, the composition of bacterial communities changed with time (Fig. 7). Bacterial communities in the control treatment were more similar to the initial bacterial communities, while changes in the filtered, bacterivore-free treatment were more conspicuous. The main phylotypes that contributed together ~50% to the differences between the bacterivore-free and the control treatments at the end of the experiment belonged to: (i) Type C response that increased in the bacterivore-free treatment (i.e., *Leadbetterella* and *Fluviicola* from Bacteroidetes, *Limnohabitans* (Betaproteobacteria), two Actinobacterial OTUs from the acI clade, and Verrucomicrobium from FukuN18 group); (ii) Type E that increased in the control treatment: Nitrosomonadaceae (Betaproteobacteria) and Candidatus *Aquirestis* (Bacteroidetes), and (iii) Type B (one OTU Verrucomicrobia from FukuN18 group) that initially grew in both treatments (the initial growth rate in the filtered treatment: 4.3 ± 0.6 d^−1^; in the control treatment: 3.7 ± 1.2 d^−1^), but continued to grow at the rate of 1.0 ± 0.5 d^−1^ only in the control treatment.

## Discussion

High-throughput sequencing methods enormously expanded our knowledge on species diversity and the genetic potential of natural microbial communities [14]. In contrast, current methods for the determination of bacterial growth and activity mostly rely on labor-intensive approaches such as microscopy, FISH, and radiolabeling. These low-throughput methods limit the number of samples and phylotypes that can be feasibly studied. Therefore, we attempted to combine a standard manipulation experiment with high-throughput sequencing to obtain information about growth responses of individual bacterial groups. Here, we estimated specific growth rates at a phylotype level from high-throughput sequencing data by amplicon read normalization using an internal standard (Fig. 1). However, one needs to bear in mind that the ARNIS method does not provide any information about bacterial abundance. The method only allows a relative comparison of the collected samples for each individual OTU. The tests performed with mock communities and on the experimental samples verified our approach (Fig. 2). The only significant problem occurred with *R. lacicola* in mock communities M4 and M5. Unfortunately, we were unable to pinpoint the cause of the substantial under-representation of its sequences in these two libraries. The problem is apparently not caused by any random errors during the handling or processing of the samples since the results were consistent in all triplicates. It also does not seem to reflect any heterogeneity in the *R. lacicola*’s cells (different no. of chromosomes or spore formation), since all mock communities were prepared from the same batch and processed within a short period of time (<30 min). Therefore, we hypothesize that the discrepancy may originate from sequencing artefacts generated at high concentrations of sequences originating from *R. lacicola*. Due to this disagreement, we put more focus on Actinobacteria in our manipulation experiment, in which the results by ARNIS and CARD-FISH were congruent (Fig. 3). Nevertheless, it is advisable to routinely provide controls when using ARNIS, for instance by combining it with FISH on a subset of samples, at least for Actinobacteria. The moderate agreement between the average specific growth rates calculated from all OTUs and the community growth rates obtained from the DAPI counts can be explained by the fact that the community growth rate corresponds to an average weighted with abundance of each phylotype, i.e., rare phylotypes contribute less to the total abundance change even if they grow at a faster pace [9, 56]. Because the proportions of reads in libraries poorly reflects microbial abundances in the samples (Figs. 2 and 3), we lack a verification proxy for our data. Still, the bacterial specific growth rates calculated from DAPI counts fell into the modal bin of the growth rates’ distribution in each treatment (Fig. 6), which further strengthens the use of ARNIS in environmental studies.

A different number of copies of genes encoding rRNA in specific bacterial phylotypes can affect snap shot studies of bacterial communities. However, the increase in number of rRNA genes during each genome replication (and subsequent cell division) is constant for a given phylotype, which makes the comparison of its read numbers between the time points feasible. *E. coli* strain K12 that we used as an internal standard contains seven rRNA operons in its genome [57]. This did not affect our results, because the addition of constant number of *E. coli* cells to the DNA samples provided always the same total number of 16S rRNA gene copies used for the ARNIS normalization.

### Insight into specific growth rates and roles in food webs

Size fractionation experiments allow to identify bacterial phylotypes that are vulnerable to grazing [32]. Here, we observed five types of bacterial responses to grazer removal (Fig. 4), suggesting different bacterial life styles and thus their distinct roles in food webs. The growth of OTUs from Type A (Fig. 4a) was limited by factors other than grazing (bottom–up control), most likely by DRP (in situ concentration 1.62 µg L^−1^; [58–60]). The specific growth rates of OTUs assigned to this type were usually negative. In contrast, OTUs assigned to Type B seemed to be unaffected by bottom–up and top–down controlling modes (Fig. 4b). These bacteria were likely released from resource limitation at the experimental *T*_0_, yet being little affected by the present community of grazers [61, 62]. Thus, it is plausible that at least some of Type B OTUs would become Type C in the course of the experiment. Such phylotypes, growing only after the removal of grazers (Fig. 4c), contribute considerably to the carbon transformation and flow to higher trophic levels [62]. In contrast, Type D, i.e., bacteria that grew only in the control treatment (Fig. 4d), represents rather a carbon pool or a sink for limiting nutrients in the microbial fraction because they are poorly grazed [63]. Interestingly, this type of response was very rare in our experiment, even among Actinobacteria that are typically considered to be defense specialists [59, 62, 64]. Finally, Type E OTUs (most common among Planktomycetes, e.g., *Rhodopirellula*, *Pirellula*) were removed by filtration, and values of their ARNIS ratio were very different between the treatments already from the beginning (Fig. 4e). The reason could be their large cell sizes and specific morphologies, like filaments or colonies [65], or association with particles or algae [66].

All types of responses could be found among most of the main bacterial phyla, but at different proportions (Fig. 5). OTUs that showed Types B and C responses were affiliated with phylotypes already known to respond quickly to grazer removal, for instance, *Flavobacterium* [9, 56, 67], or proteobacterial genera *Pseudomonas* or *Polynucleobacter* [68, 69]. Interestingly, closely related phylotypes grew at different rates, e.g., OTUs affiliated with the genus *Limnohabitans*, an important component of freshwater food webs [59, 61, 62, 70, 71] with high substrate and environmental versatility [37, 72, 73]. Three OTUs from the *Limnohabitans* lineages LimB and LimC grew at rates 0.5, 2.0, and 3.3 d^−1^, which likely corresponded to their in situ activity and substrate preferences at the time of the experiment [74].

Bacteroidetes and Proteobacteria also contributed to grazing-resistant bacteria (Type D, Fig. 4d, Fig. 5, [69, 75]), but typical defense specialists belong to Actinobacteria from the acI clade [59, 62, 64] or Luna 2 cluster [7, 70]. However, in our study, most Actinobacterial OTUs did not grow in any treatment (Fig. 5, Type A). This corroborates with the recent discovery that acI Actinobacteria (Ca. ‘Nanopelagicales’) are auxotrophs [76]. Maxima of actinobacterial abundance occurs shortly after phytoplankton blooms, which is attributed to their grazing resistance [56, 67, 77, 78]. Here, many Actinobacteria, including half of the OTUs affiliated with acI clade, and genera *Auqiluna* and *Planktoluna*, grew in the bacterivore-free treatment at rates up to 6 d^−1^ (Fig. 5). This indicates more diverse lifestyles of some Actinobacteria that might substantially contribute not only to the bacterial abundance but also to the activity and carbon fluxes, at least in early autumn.

Our approach allowed for a closer look at specific growth rates and role in food web also of less-known freshwater bacterial phyla like Verrucomicrobia. They are a common component of bacterial communities [29], whose abundance in freshwater seems to correlate with phytoplankton and rotifers maxima [79], temperature, and water retention time [16, 80]. Many verrucomicrobial OTUs were associated with Type E (Fig. 4e, Fig. 5), and they were affiliated with species known to degrade macromolecules (e.g., cellulose, starch, proteins, [81–84]). Considering their rather high specific growth rates (0.98 up to 4.40 d^−1^) and the metabolic versatility, Verrucomicrobia with Type E response might be an important indirect source of simple organic matter for other bacteria via, e.g., cross-feeding effect in mixed communities [85]. However, the most common verrucomicrobial OTUs related to FukuN18 phylotypes belonged to Types B or C, indicating its importance in carbon transfer to higher trophic levels. This suggestion is in line with the fact that their cell sizes are in a suitable range for being HNF bacterivores [86, 87].

### Toward understanding bacterial communities

Freshwater bacterial communities are highly dynamic, and it is still little understood how they are assembled, and how this reflects their growth potential [8, 32, 63, 88]. Bacterial communities consist of hundreds of phylotypes, each occupying a different niche [11, 16–21]. The composition and changes of bacterial communities depend on how the growth of specific phylotypes corresponds to the given environmental conditions. The shift in our experimental communities could be explained by the growth of bacteria from Types B and C in the filtered treatments, and Types D and E bacteria in the control treatment (Fig. 7). The OTUs that contributed to the observed differences (SIMPER analysis) were usually abundant and growing at rates above the community average. In contrast, OTUs exposed to high mortality seemed to be less important for structuring the bacterial community in our experiment (Fig. 7).

The removal of bacterivores modulated the growth response type of different phylotypes, shifting the distribution of specific growth rate from bimodal to unimodal (Fig. 6b–e). This effect was less conspicuous in the control treatment, where two modes, in negative (grazed cells) and positive (growing cells) values, were observed. This supports the hypothesis that bacterivores reduce the competition for resources and allow the existence of many bacterial phylotypes [7, 69, 71]. This also indicates that most of the specific growth rates here were likely below the maximum potential that can be reached under optimal conditions.

### Perspectives

We believe that the proposed approach has a large potential to advance the field of environmental microbiology. Its use is not limited to grazer-removal experiments: the ARNIS can be used in any manipulation experiments, e.g., in dilution experiments to determine the potential specific growth rates; in experiments with enhanced HNF grazing upon zooplankton removal to identify its impact on the community; in nutrient enrichments or temperature shift experiments, etc. The use of Illumina amplicon sequencing makes it possible to obtain high phylogenetic resolution and depth of sequencing at an affordable price. This makes our approach especially suitable for investigation within the “rare biosphere”. The rare species (<0.1% of total cells) are impossible to enumerate using FISH, but deep enough sequencing can still capture their growth responses with sufficient accuracy.

In summary, ARNIS has a large potential to be applied in various environmental studies, where it can enhance our understanding of the activity, growth, and life styles of aquatic bacteria at high phylogenetic resolution.

## Electronic supplementary material

Supplementary File 1

Supplementary Figure 1

Supplementary Table 1

Supplementary Table 2

Supplementary Table 3
