# Supplementary material for: Determining lineage-specific bacterial growth curves with a novel approach based on amplicon reads normalization using internal standard (ARNIS)
Source: ISME J. 2018 Jul 6;12(11):2640–54. doi: 10.1038/s41396-018-0213-y (PMC6194029; doi:10.1038/s41396-018-0213-y)
Supplement: Supplementary file 1 [file 41396_2018_213_MOESM1_ESM.pdf]

Determining lineage-specific bacterial growth curves with a novel approach based on amplicon reads normalization using internal standard (ARNIS).

Kasia Piwosz[1], Tanja Shabarova[2], Jürgen Tomasch[3], Karel Šimek[2], Karel Kopejtka[1,4], Silke Kahl[3], Dietmar H. Pieper[3], Michal Koblížek[1,4#]

[1] Center Algatech, Institute of Microbiology CAS, 37981 Třeboň, Czech Republic

[2] Biology Centre CAS, Institute of Hydrobiology, Na Sádkách 7, 37005 Česke Budějovice, Czech Republic

[3] Helmholtz Centre for Infection Research, 38124 Braunschweig, Germany

[4] Faculty of Science, University of South Bohemia in České Budějovice, CZ-37005 České Budějovice, Czech Republic

[#]Corresponding author: koblizek@alga.cz

Running Head: High resolution data on bacterial growth rates

Results

Types of bacterial response to the experimental grazer removal

The general picture of bacterial responses differed for phyla and classes (Fig. 5). 50% of OTUs affiliated with Actinobacteria belonged to Type A, including many OTUs affiliated with an abundant freshwater clade Ac1. Type B comprised 13% of Actinobacteria, e.g. *Aquiluna* and *Planktoluna*. Interestingly, 27% of Actinobacterial OTUs grew only in the filtered treatment (Type C), e.g. OTUs affiliated with clade Ac1, *Planktophila* and *Limnoluna.* None of Actinobacteria belonged to Type D, and only 6% to Type E, for example Microbacteriaceae and Acidimicrobineae (Fig. 5).

Contrasting pattern was observed for Bacteroidetes, of which only 7% belonged to Type A (mainly uncultured Sphingobacteriales from marine group NS11-12, Fig. 5), and 21% to Type B (e.g. *Algoriphagus*, *Flavobacterium* and *Pedobacter*). Most Bacteroidetes, including many Chitinophagaceae, belonged to Type C. Only 10% belonged to Type D (e.g. *Chryseobacterium*, *Flavobacterium* and *Pedobacter*), and 27% to Type E (Fig. 5), for instance Candidatus *Aquirestis*, *Ferruginibacter*, *Chryseobacterium* and *Hydrotalea*.

Almost all OTUs affiliated with Planctomycetes (including genera *Pirellula*, *Rhodopirellula* and many unclassified OTUs) belonged to Type E, while Type A and Type B each included one unclassified OTU from class Planctomycetaceae (Fig. 5).

Five Verrucomicrobial OTUs belonged to Type A (mainly unclassified), six to Type B (e.g. *Prosthecobacter, Brevifollis* but mainly FukuN18 freshwater group), and seven to Type C (FukuN18 group, OPB35 soil group, *Luteolibacter*, Chthoniobacterales, Opitutae). Type E was

dominant (14 OTUs, mainly *Prosthecobacter* or unclassified), but not a single OTU belonged to Type D (Fig. 5).

The distribution of response types among phylum Proteobacteria was similar to all bacteria (Type A: 20%, Type B: 15%, Type C: 29%, Type D: 9% and Type E: 26%), but showed pronounced differences at the class level (Fig. 5). Four of Alphaproteobacterial OTUs belonged to Type A (e.g. *Methylobacterium*), and four to Type B (e.g. *Roseomonas*). Three belonged to Type C (e.g. LD12 group) or Type D (e.g. *Brevundimonas*), while clearly dominant Type E contained eleven OTUs (e.g. Sphingomonadales, Hyphomonadaceae, *Caulobacter*, *Phenylobacterium*, Fig. 5). In contrast, almost half of Betaproteobacterial OTUs were classified to Type C (17 OTUs, e.g. *Limnohabitans*, *Polynucleobacter*), while only five belonged to Type B (from Comamonadaceae) or Type E (e.g. Comamonadaceae and Nitrosomonadaceae), and eleven to Type A (Comamonadaceae and Burkholderiales, Fig. 5). Type C was also the most common for Gammaproteobacteria (eight OTUs, e.g. *Pseudomonas*, *Rheinheimera*). Single unclassified OTU belonged to Type A, three OTUs to Type B (*Acinetobacter*, *Legionella*), three to Type D (*Legionella* and CHAB XI-27 clade), and two to Type E (*Aeromonas* and BD1-7 clade, Fig. 5).

Bacterial growth rates and whole community response to the experimental grazer removal

The observed bulk growth rates were resultant of individual growth rates of different bacterial phylotypes in the treatments (Fig. 6B-E). In the control treatment, the initial growth rates ranged from -7.1 (*Leptospira*, Spirochaetes) to 10.7 d^{-1} (*Aeromonas*, Gammaproteobacteria), and showed bimodal distribution (Fig. 6B). Top 10 of the fastest growing (> 7.4 d^{-1}) OTUs were affiliated within Bacteroidetes (e.g. *Emticicia*, Saprospiraceae, Chryseobacterium), Firmicutes, and Proteobacteria (Comamonadaceae), while the top 10 OTUs with the fastest decay rates (< -4.2 d^{-1}) belonged to Actinobacteria (clade Ac1, Microbacteriaceae), Verrucomicrobia (Opitutae,

Verrucomicrobiaceae), Bacteroidetes (e.g. Chitinophagaceae, Sphingobacteriales), Planctomycetes, and Proteobacteria (*Brevundimonas*, Comamonadaceae). The distribution of the growth rates remained bimodal in the second stage of the control experiments (after 21h), but it become more skewed toward negative values, and the range narrowed (Fig. 6C): it varied from -5.0 (*Staphylococcus*, Firmicutes) to 3.3 d^{-1} (uncultured Planctomycetes). Top 10 of the fasted growing (> 2.3 d^{-1}) OTUs in this second stage were affiliated with Actinobacteria (clade Ac1, *Limnoluna*), Bacteroidetes, Planctomycetes, Aquificae), and Proteobacteria (Acetobacteraceae, *Aquabacterium*). The top 10 OTUs with fastest decay rates (< -1.8 d^{-1}) belonged to Actinobacteria (Corynebacteriaceae, *Propionibacterium*), Bacteroidetes, Planctomycetes, and Proteobacteria (*Methylobacterium*, *Acinetobacter*, *Aeromonas*).
