# Supplementary material for: Determining lineage-specific bacterial growth curves with a novel approach based on amplicon reads normalization using internal standard (ARNIS)
Source: ISME J. 2018 Jul 6;12(11):2640–54. doi: 10.1038/s41396-018-0213-y (PMC6194029; doi:10.1038/s41396-018-0213-y)
Supplement: Supplementary file 2 — Supplementary Figure 1 [file 41396_2018_213_MOESM2_ESM.pdf]

Supplementary Figure 1. Dynamics of specific bacterial lineages determined with the ARNIS ratio in the control treatment (red colour) and bacterivore-free (Filtered−1micron) treatment (blue colour). Circles and triangles show values in replicates of the control and bacterivore-free treatments, respectively. Lines connect average values (crossed circles) from the treatments.

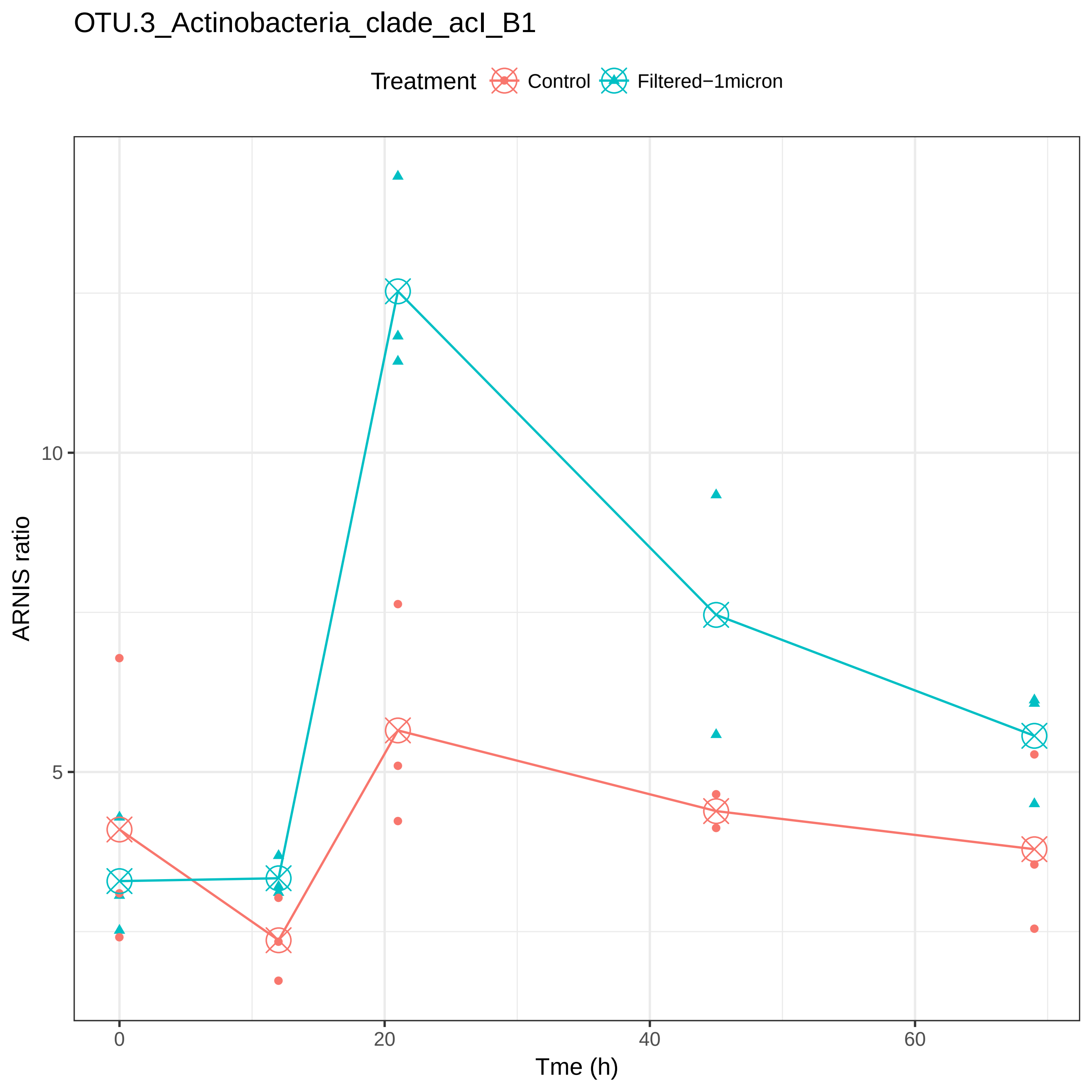
OTU.3_Actinobacteria_clade_acI_B1
Treatment
Control
Filtered−1micron
ARNIS ratio
10
5
0
20
40
60
Tme (h)

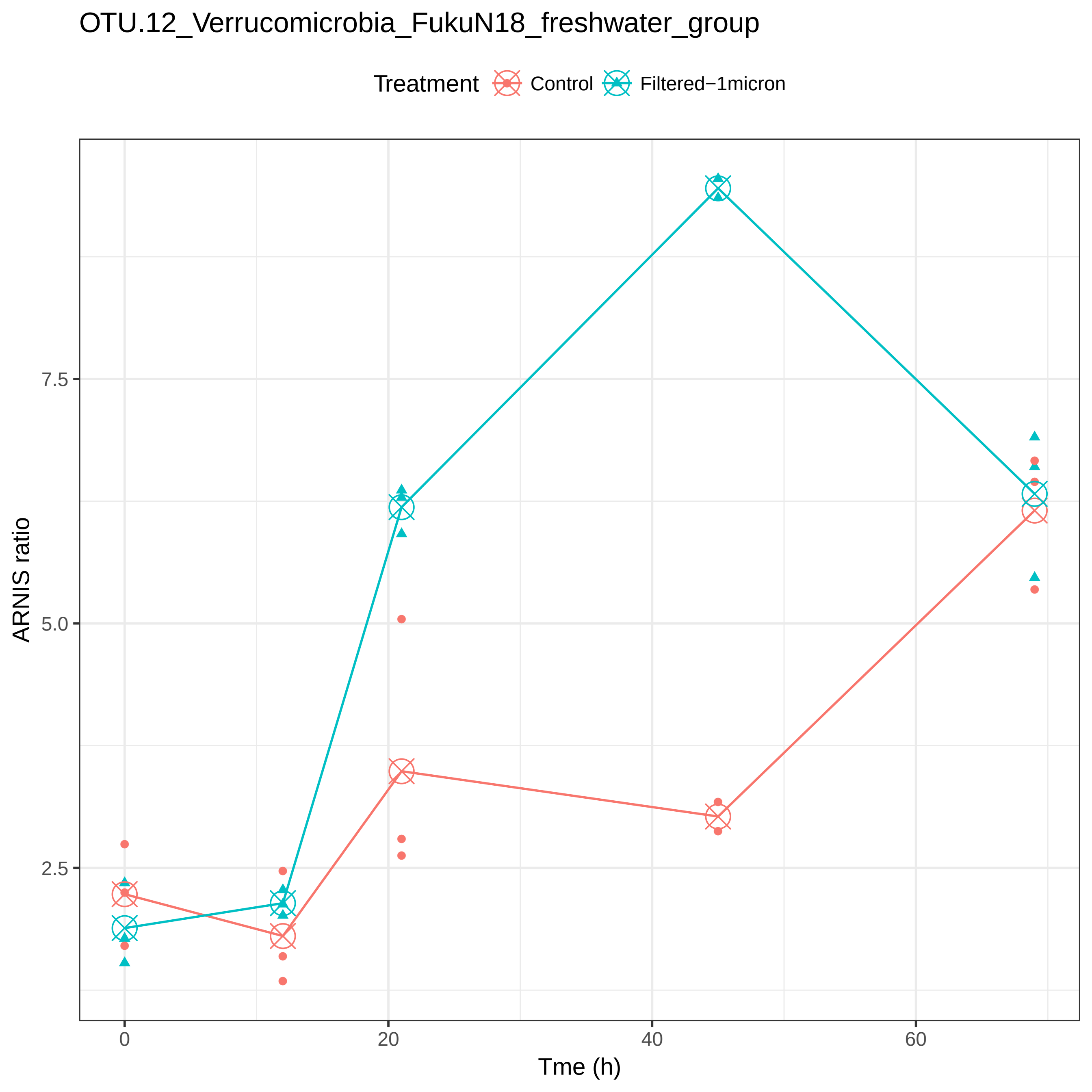

OTU.12_Verrucomicrobia_FukuN18_freshwater_group
Treatment
Control
Filtered−1micron
ARNIS ratio
7.5
5.0
2.5
0
20
40
60
Tme (h)

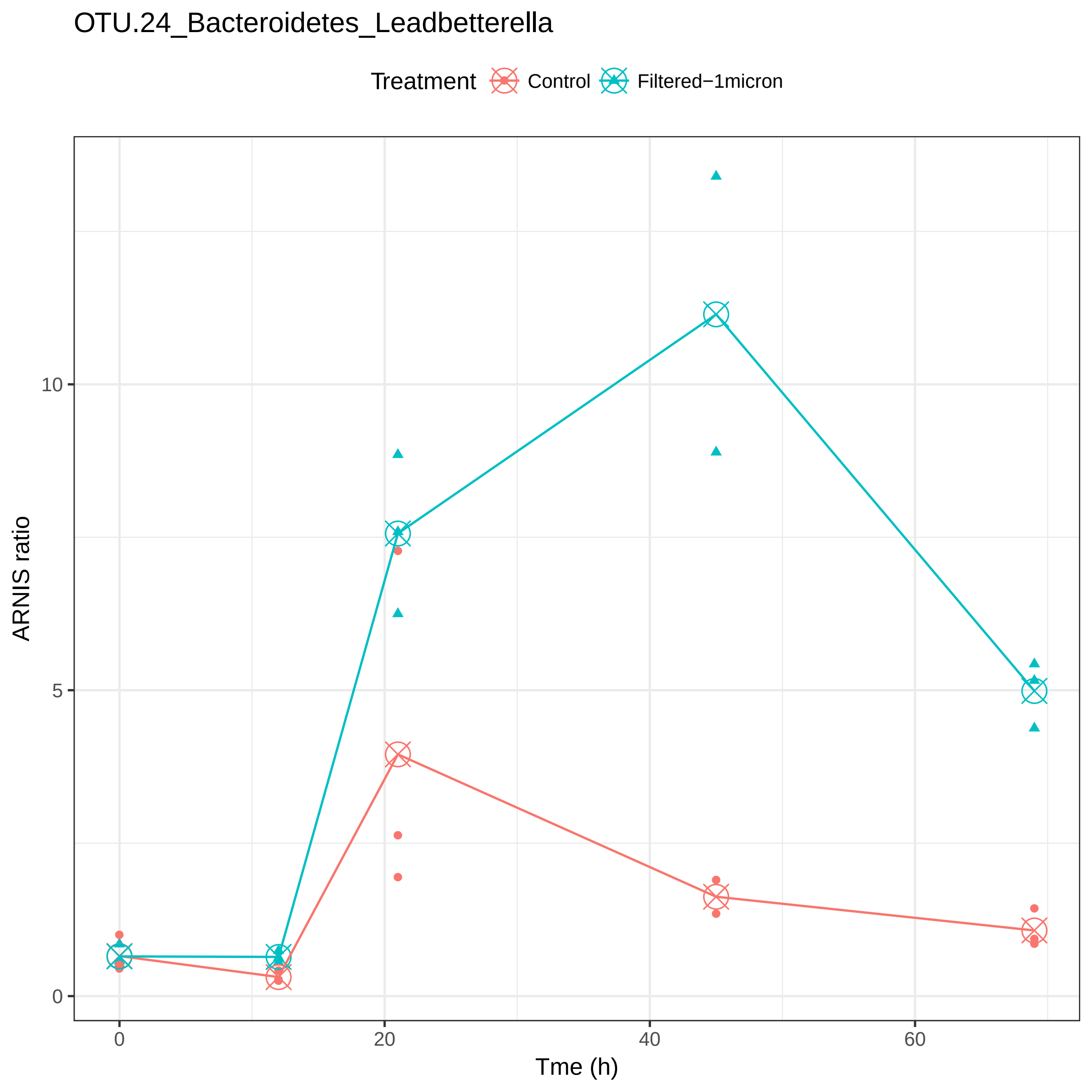
OTU.24_Bacteroidetes_Leadbetterella
Treatment
Control
Filtered−1micron
ARNIS ratio
10
5
0
0
20
40
60
Tme (h)

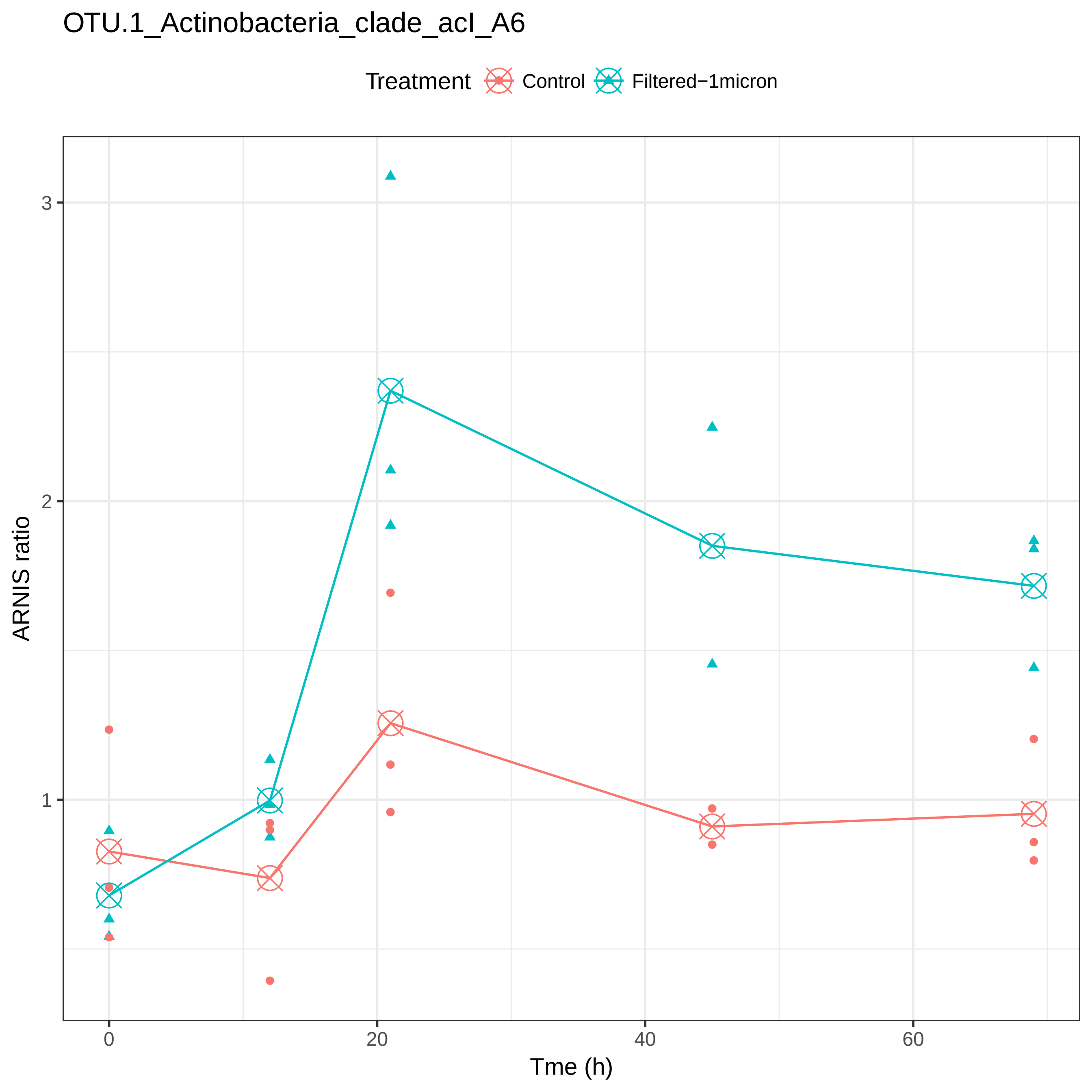
OTU.1_Actinobacteria_clade_acI_A6
Treatment
Control
Filtered−1micron
ARNIS ratio
3
2
1
0
20
40
60
Tme (h)

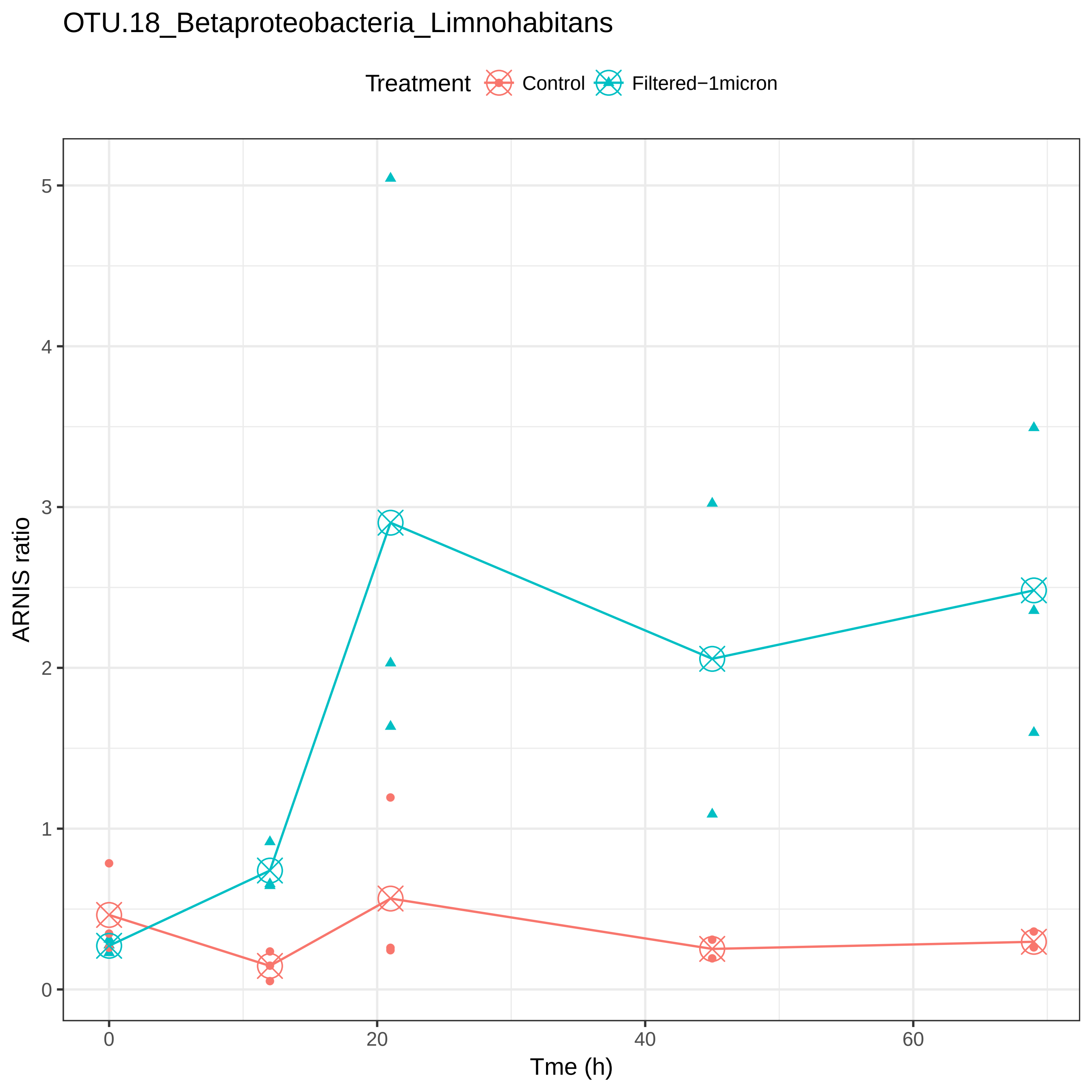
OTU.18_Betaproteobacteria_Limnohabitans
Treatment
Control
Filtered−1micron
ARNIS ratio
Tme (h)
0
1
2
3
4
5
0
20
40
60

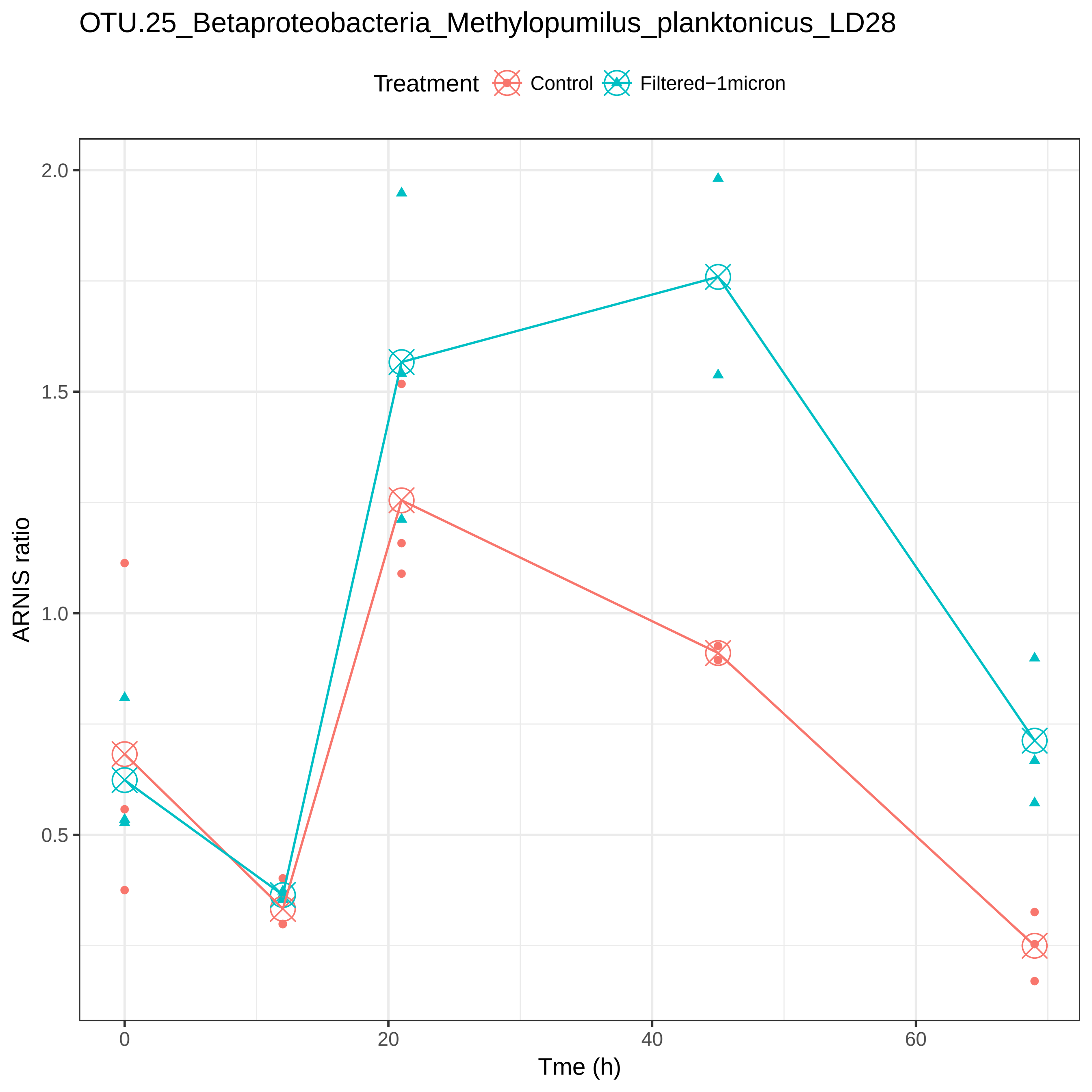

OTU.25_Betaproteobacteria_Methylopumilus_planktonicus_LD28
Treatment
Control
Filtered−1micron
ARNIS ratio
2.0
1.5
1.0
0.5
0
20
40
60
Tme (h)

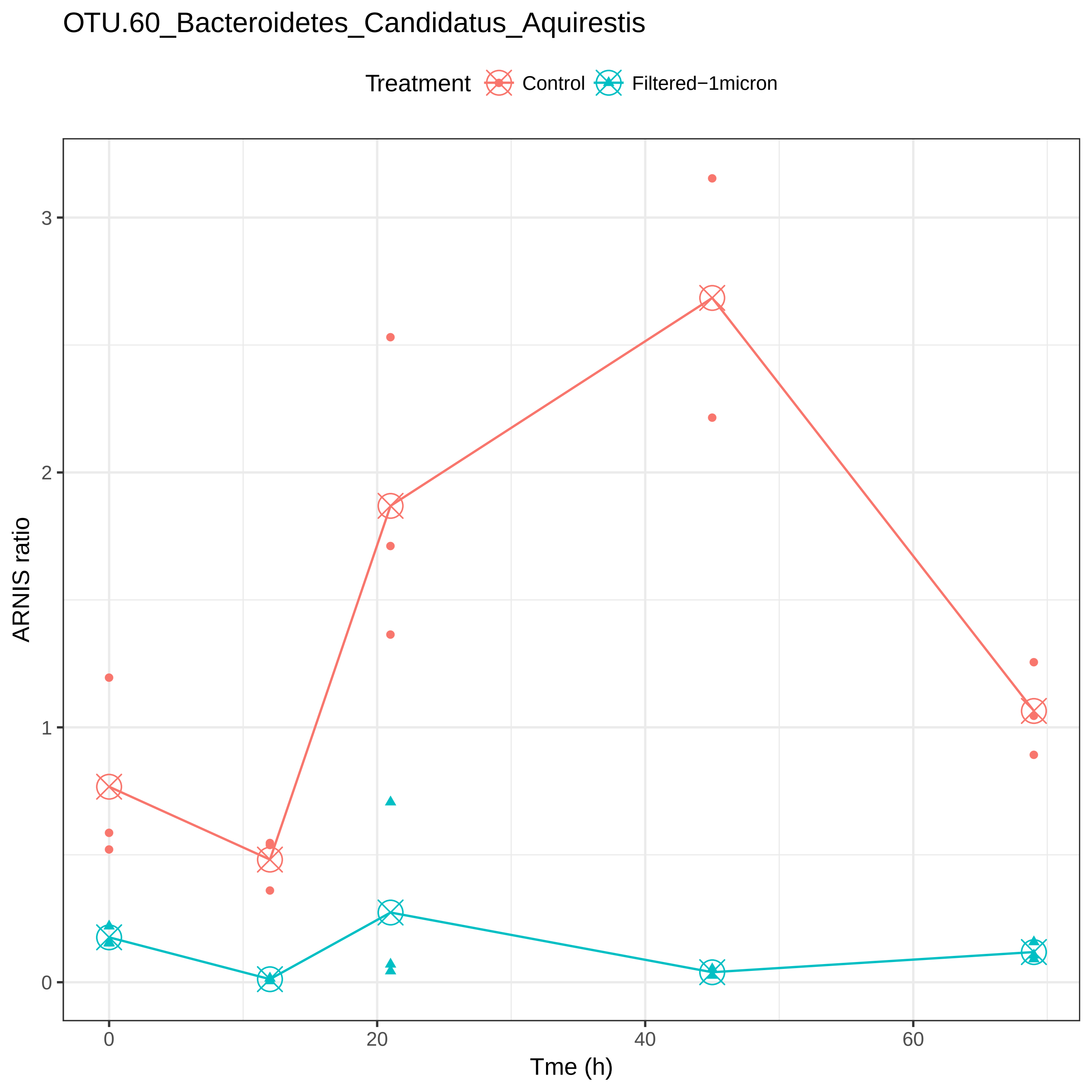

OTU.60_Bacteroidetes_Candidatus_Aquirestis
Treatment
Control
Filtered−1micron
ARNIS ratio
0
1
2
3
0
20
40
60
Tme (h)

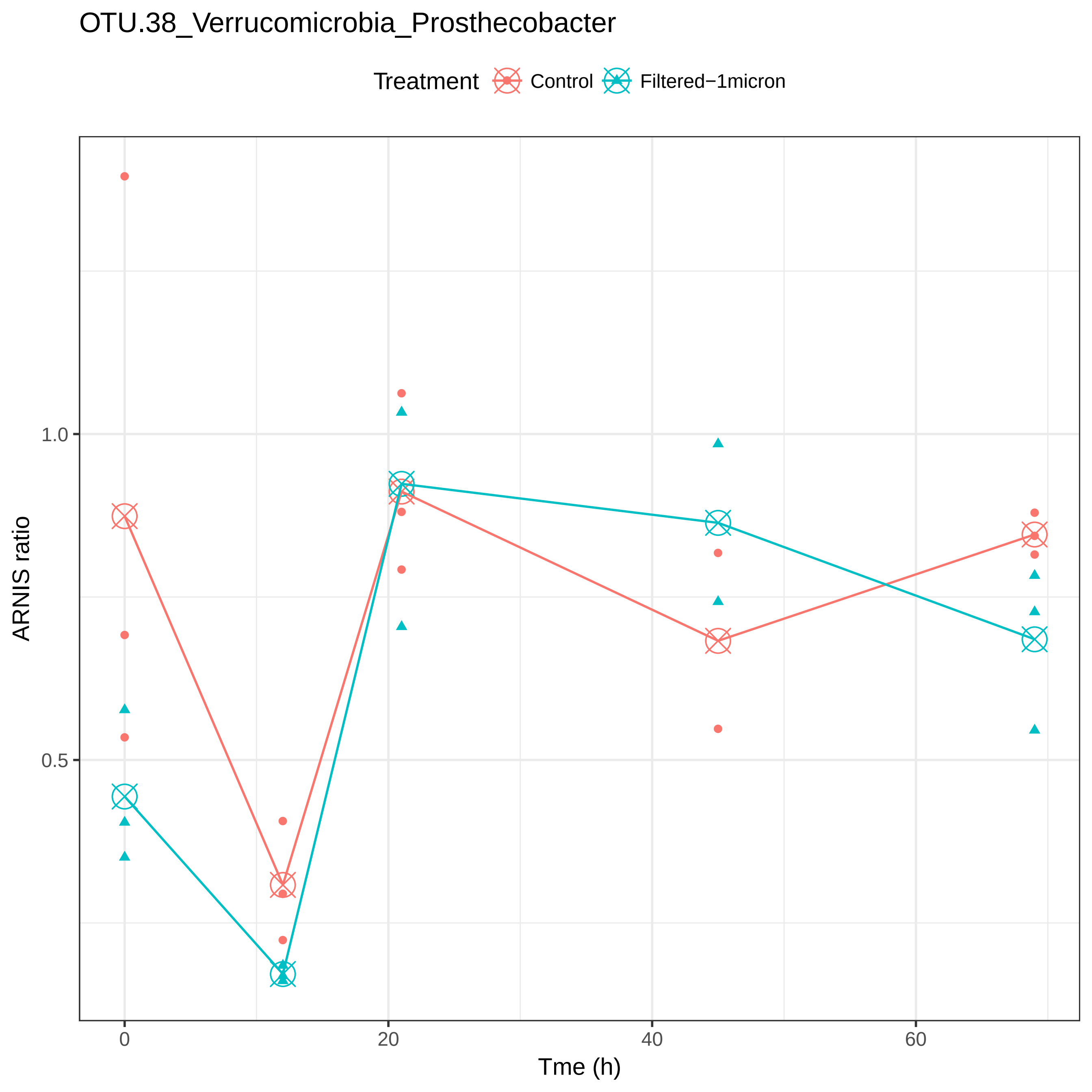
OTU.38_Verrucomicrobia_Prosthecobacter
Treatment
Control
Filtered−1micron
ARNIS ratio
1.0
0.5
0
20
40
60
Tme (h)

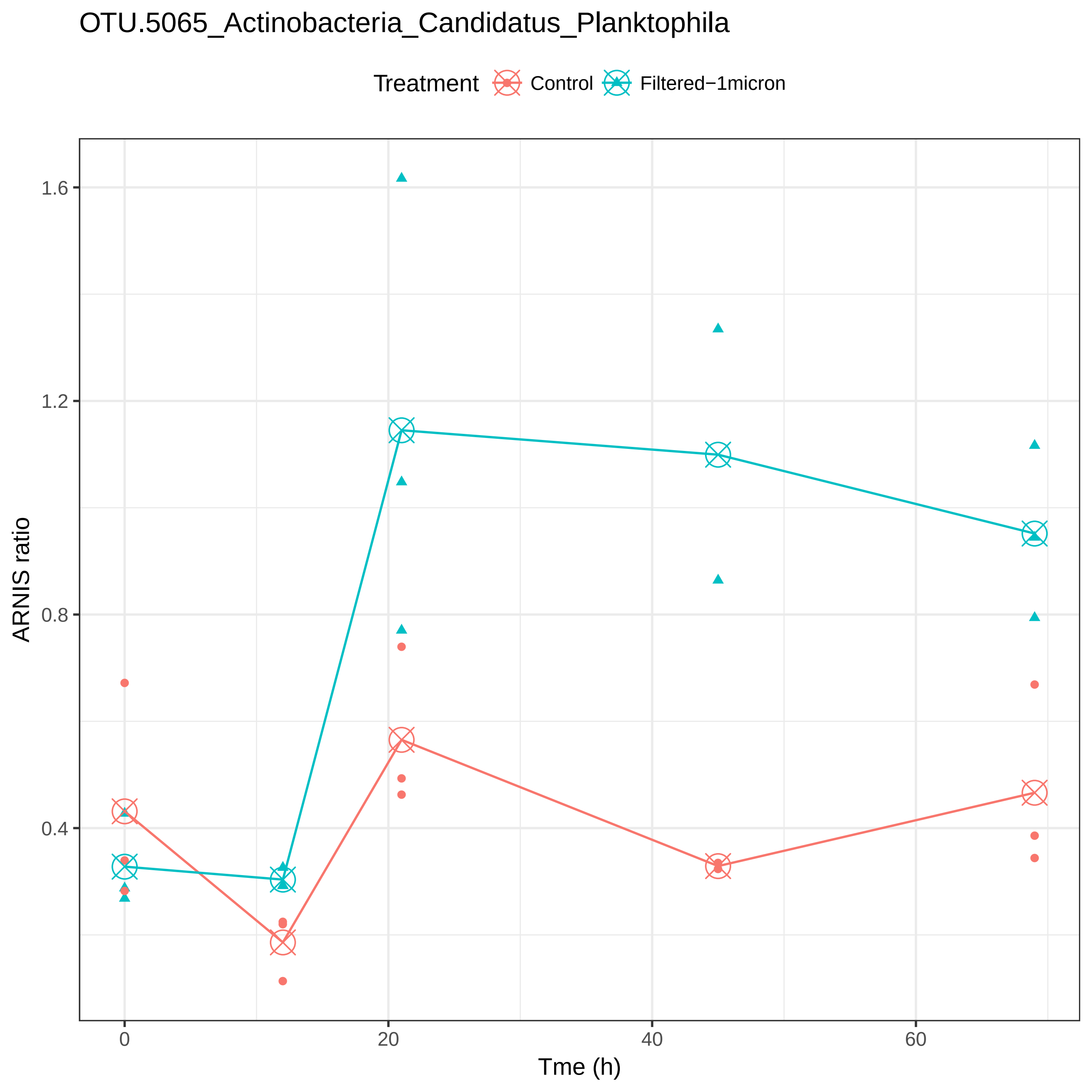

OTU.5065_Actinobacteria_Candidatus_Planktophila
Treatment
Control
Filtered−1micron
ARNIS ratio
1.6
1.2
0.8
0.4
0
20
40
60
Tme (h)

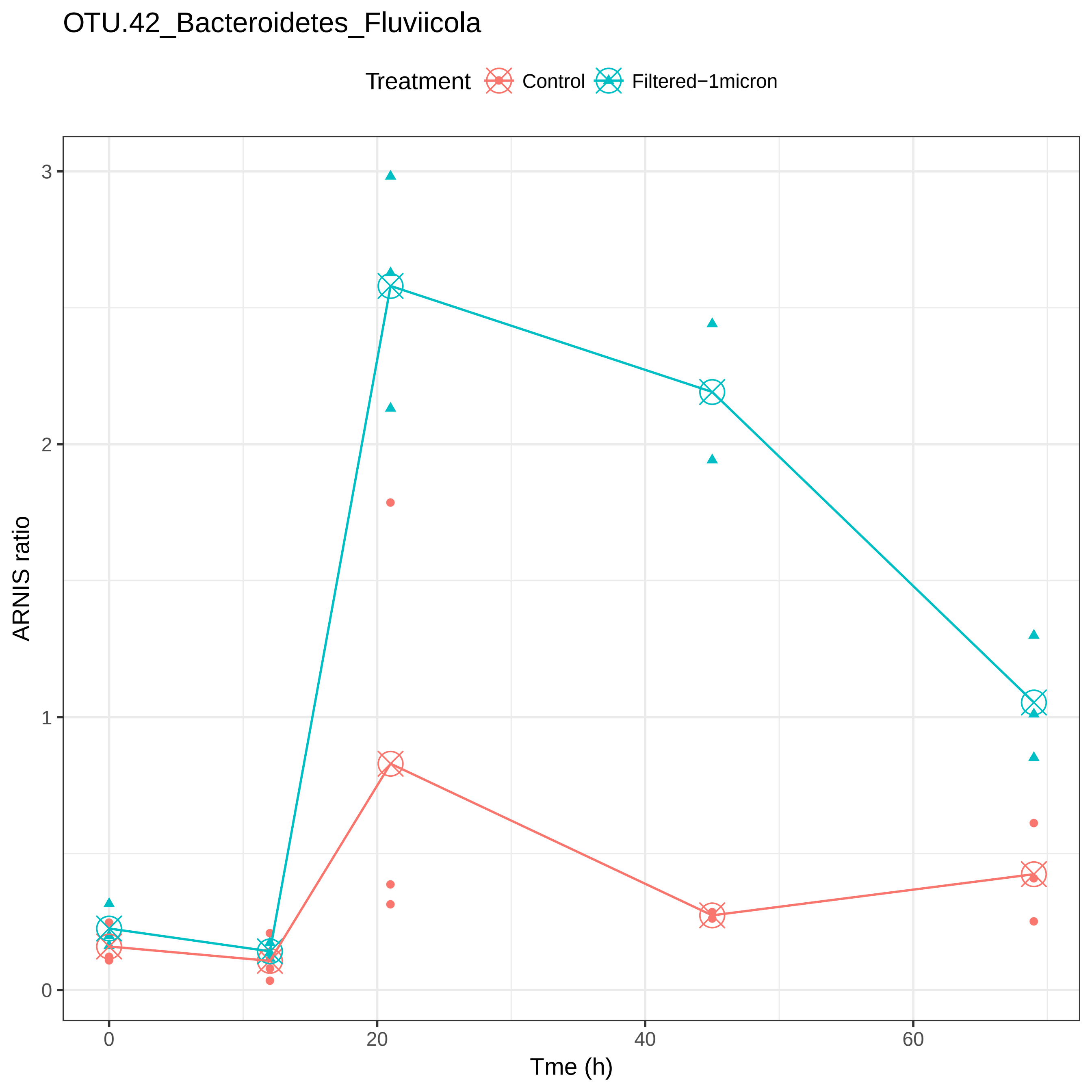

OTU.42_Bacteroidetes_Fluviicola
Treatment
Control
Filtered−1micron
ARNIS ratio
0
1
2
3
0
20
40
60
Tme (h)

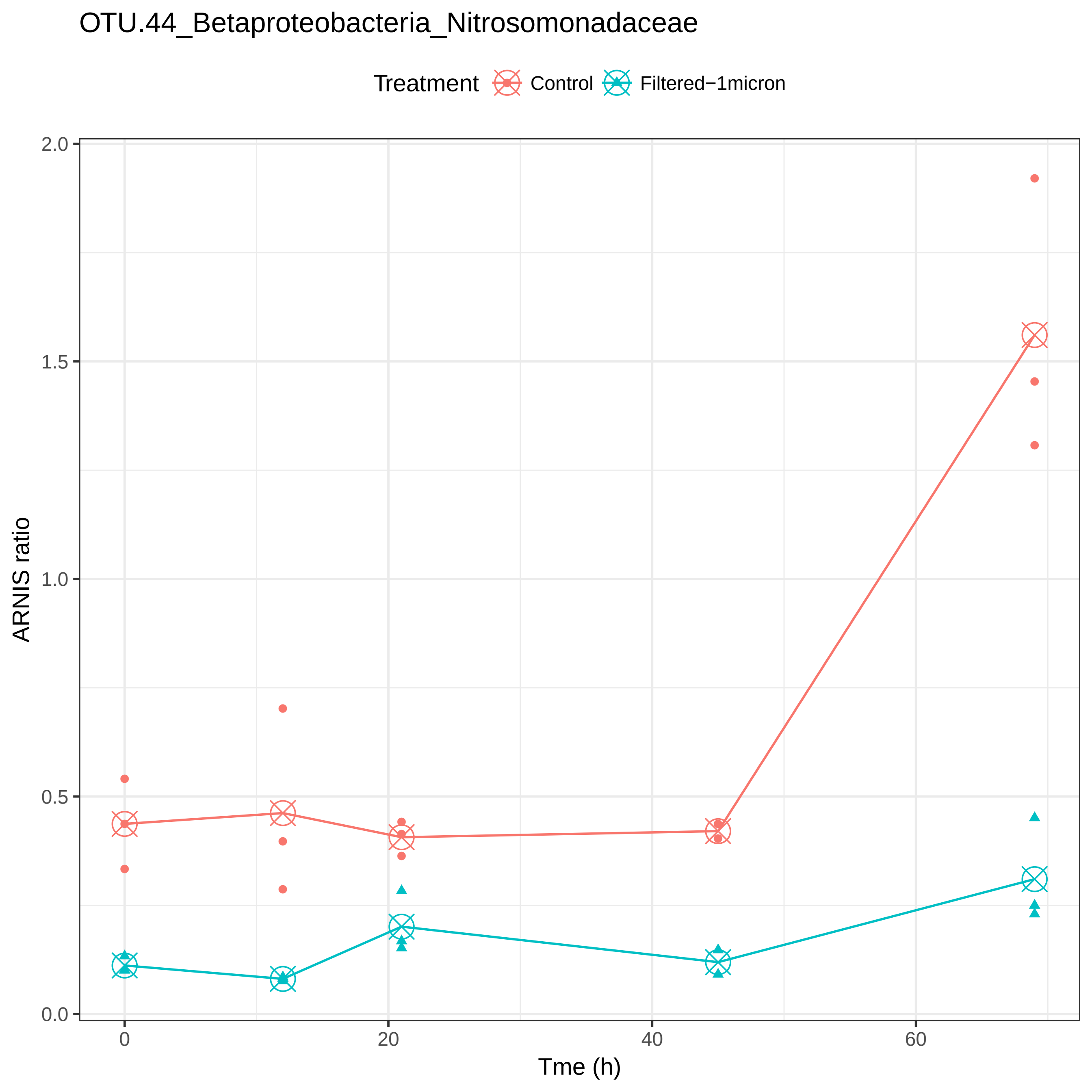

OTU.44_Betaproteobacteria_Nitrosomonadaceae
Treatment
Control
Filtered−1micron
ARNIS ratio
2.0
1.5
1.0
0.5
0.0
0
20
40
60
Tme (h)

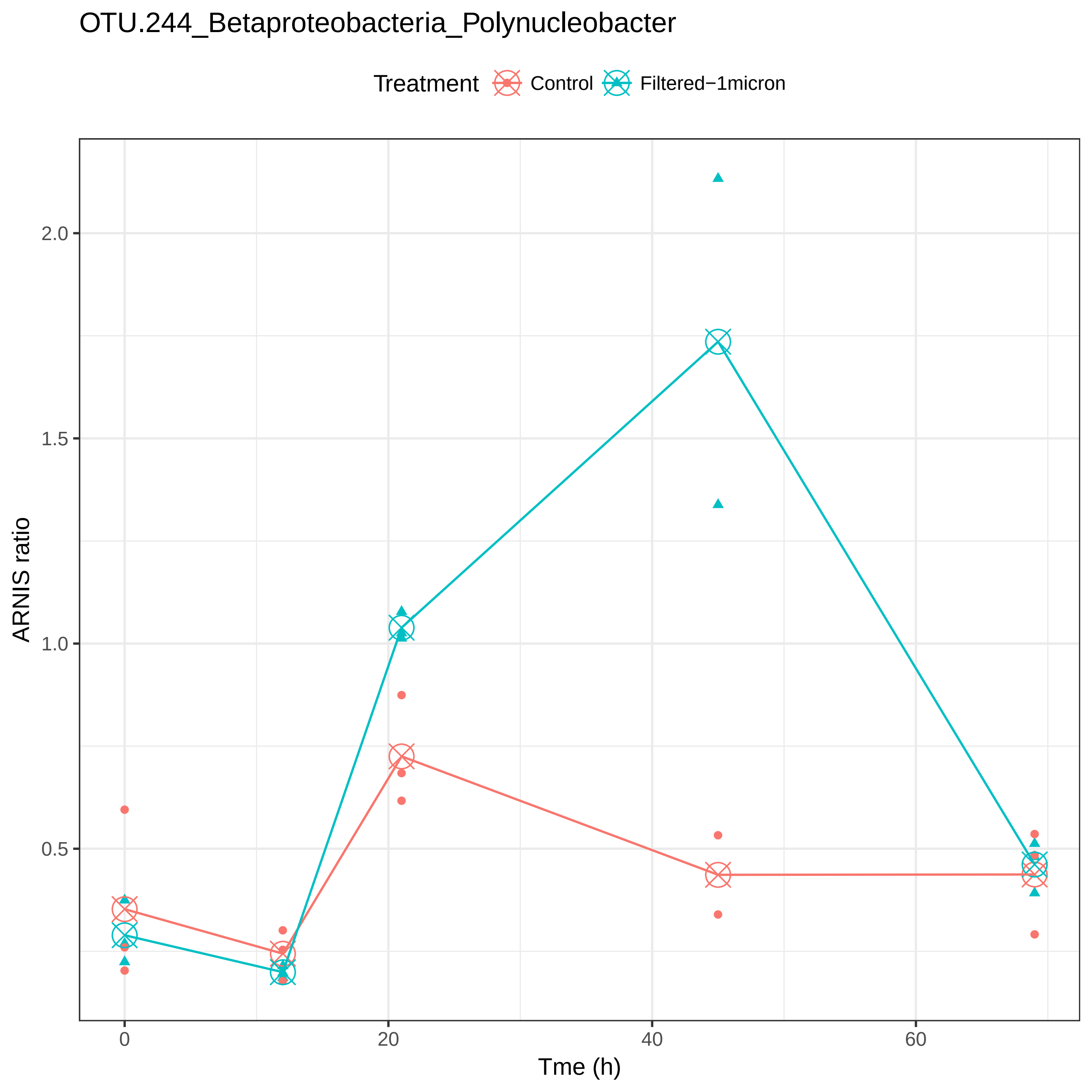

OTU.244_Betaproteobacteria_Polynucleobacter
Treatment
Control
Filtered−1micron
ARNIS ratio
2.0
1.5
1.0
0.5
0
20
40
60
Tme (h)

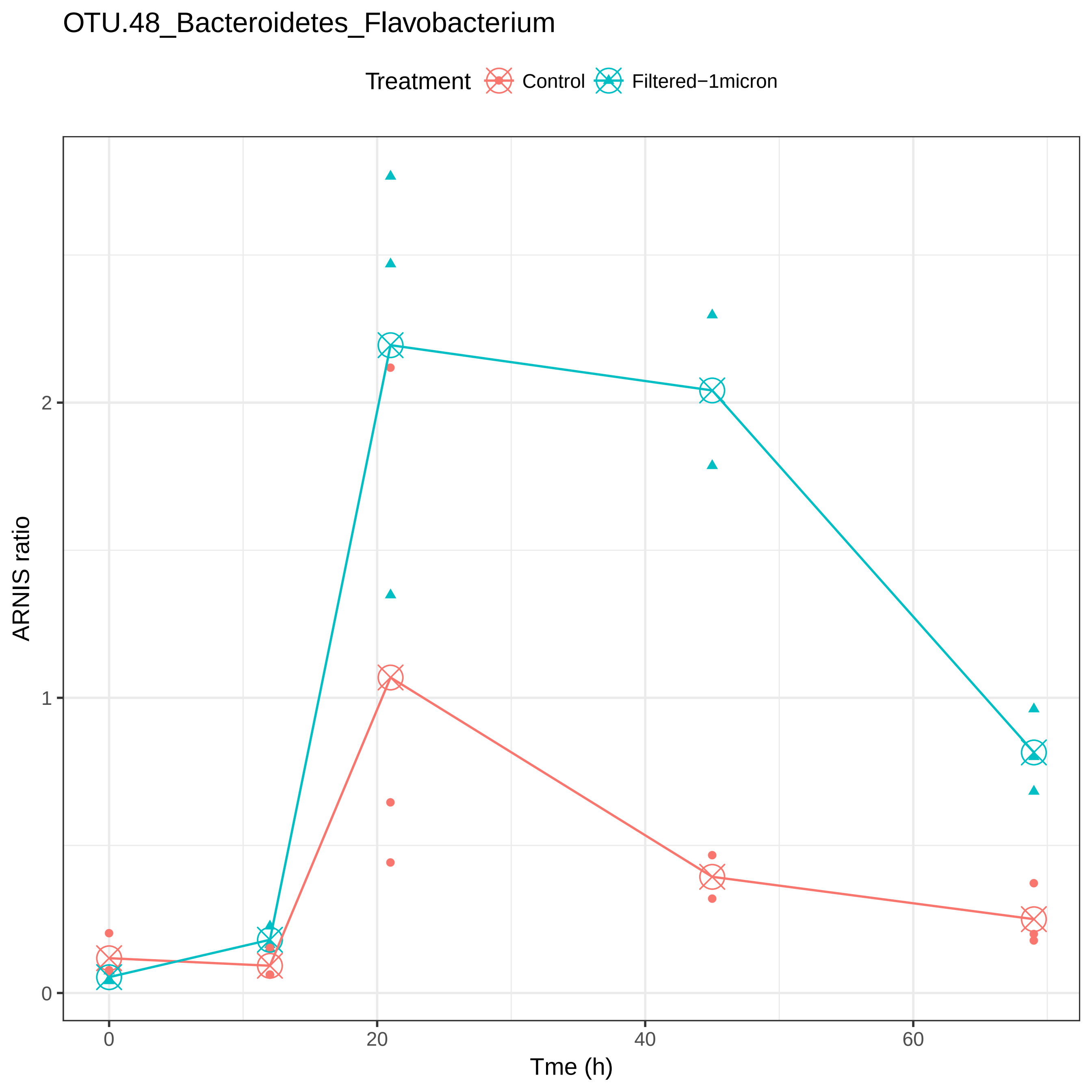

OTU.48_Bacteroidetes_Flavobacterium
Treatment
Control
Filtered−1micron
ARNIS ratio
0
1
2
0
20
40
60
Tme (h)

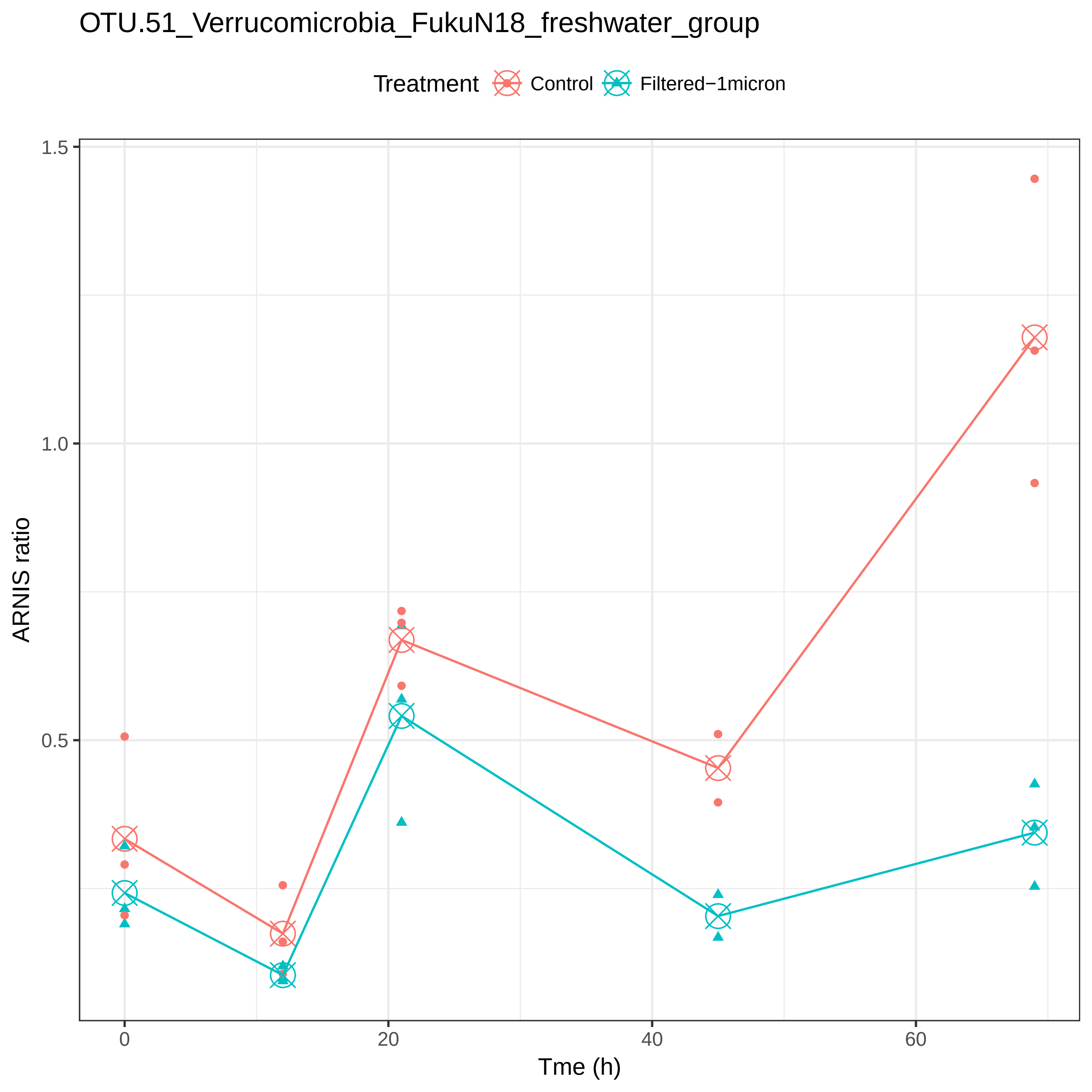

OTU.51_Verrucomicrobia_FukuN18_freshwater_group
Treatment
Control
Filtered−1micron
ARNIS ratio
1.5
1.0
0.5
0
20
40
60
Tme (h)

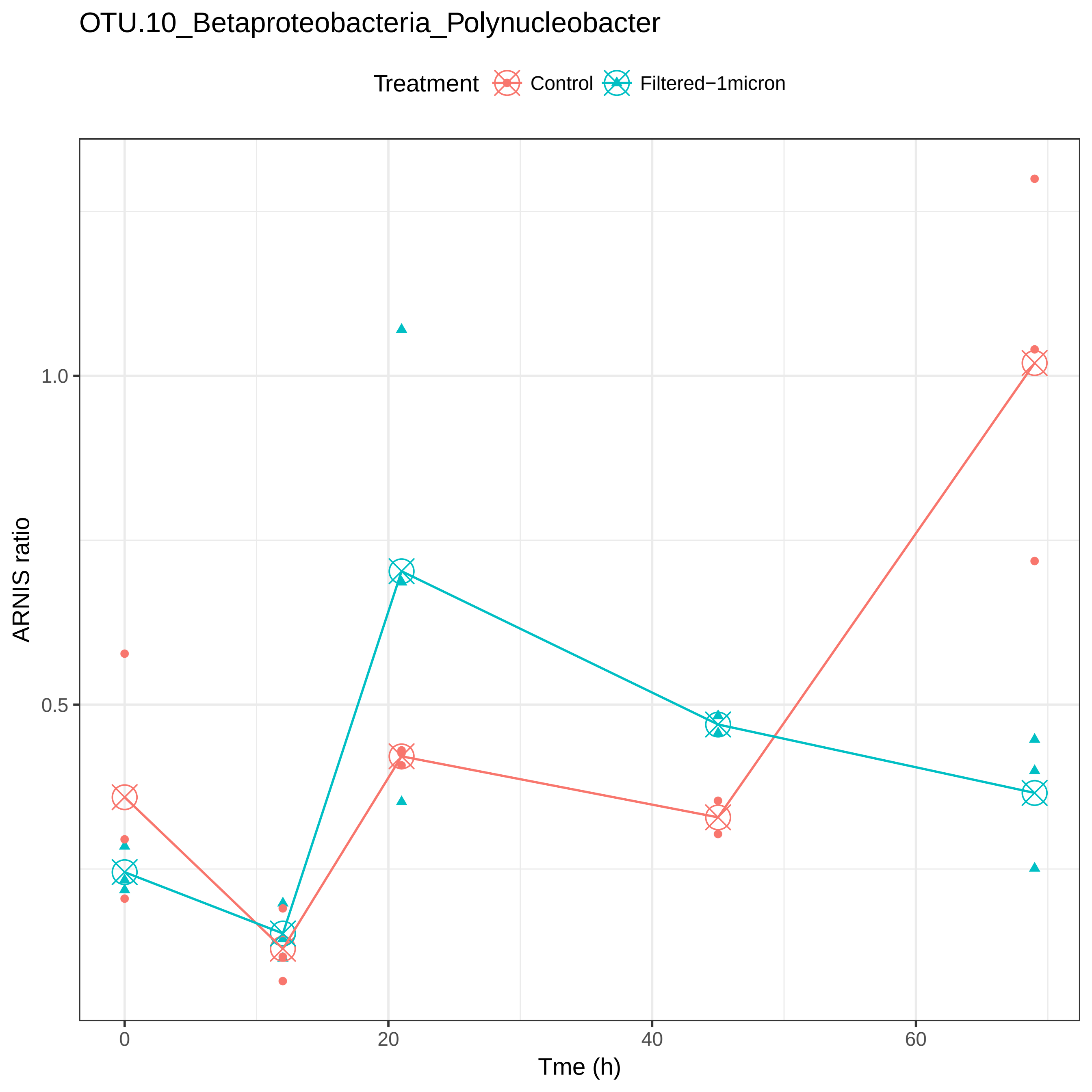

OTU.10_Betaproteobacteria_Polynucleobacter
Treatment
Control
Filtered−1micron
ARNIS ratio
1.0
0.5
0
20
40
60
Tme (h)

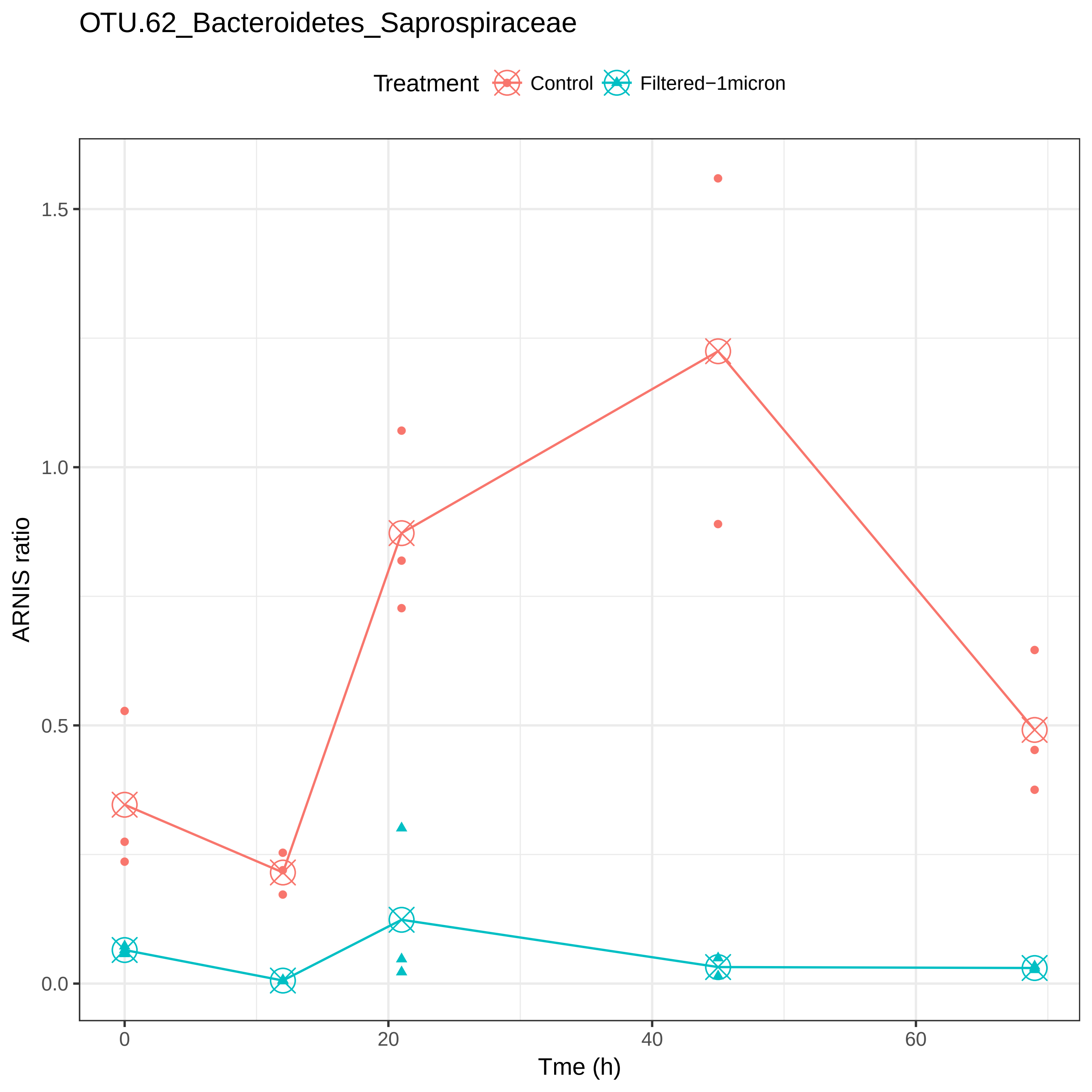
OTU.62_Bacteroidetes_Saprospiraceae
Treatment
Control
Filtered−1micron
ARNIS ratio
1.5
1.0
0.5
0.0
0
20
40
60
Tme (h)

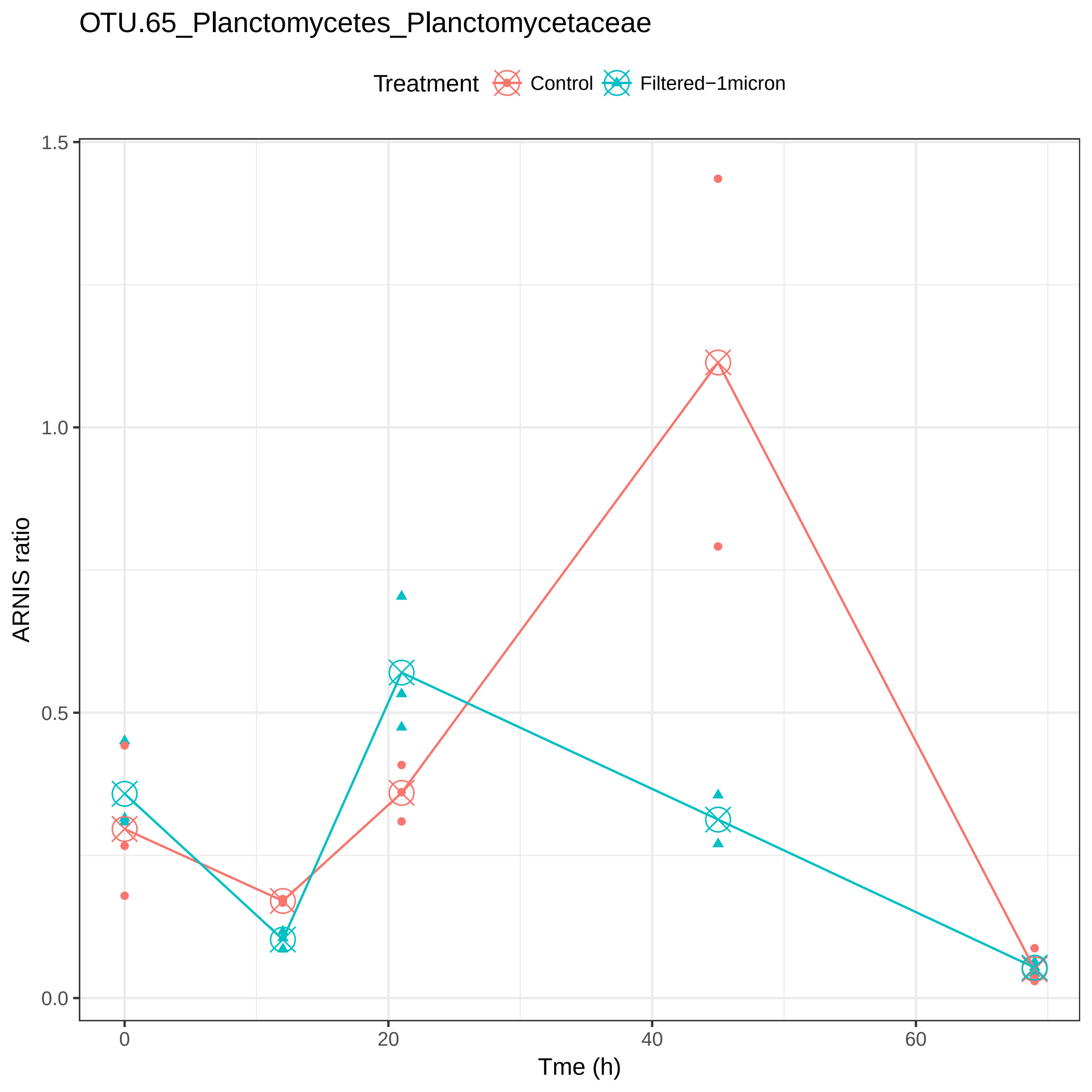

OTU.65_Planctomycetes_Planctomycetaceae
Treatment
Control
Filtered−1micron
ARNIS ratio
1.5
1.0
0.5
0.0
0
20
40
60
Tme (h)

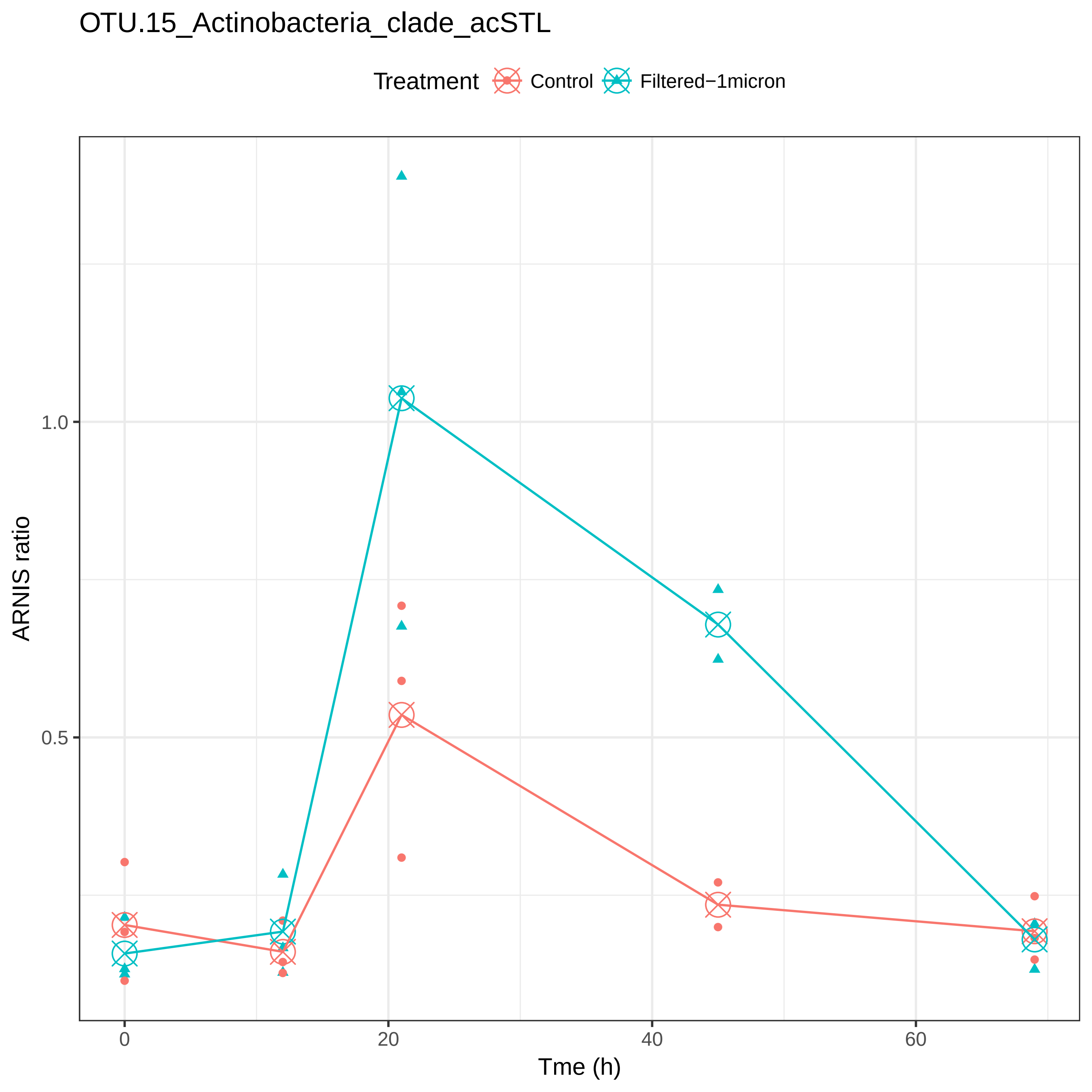
OTU.15_Actinobacteria_clade_acSTL
Treatment
Control
Filtered−1micron
ARNIS ratio
1.0
0.5
0
20
40
60
Tme (h)

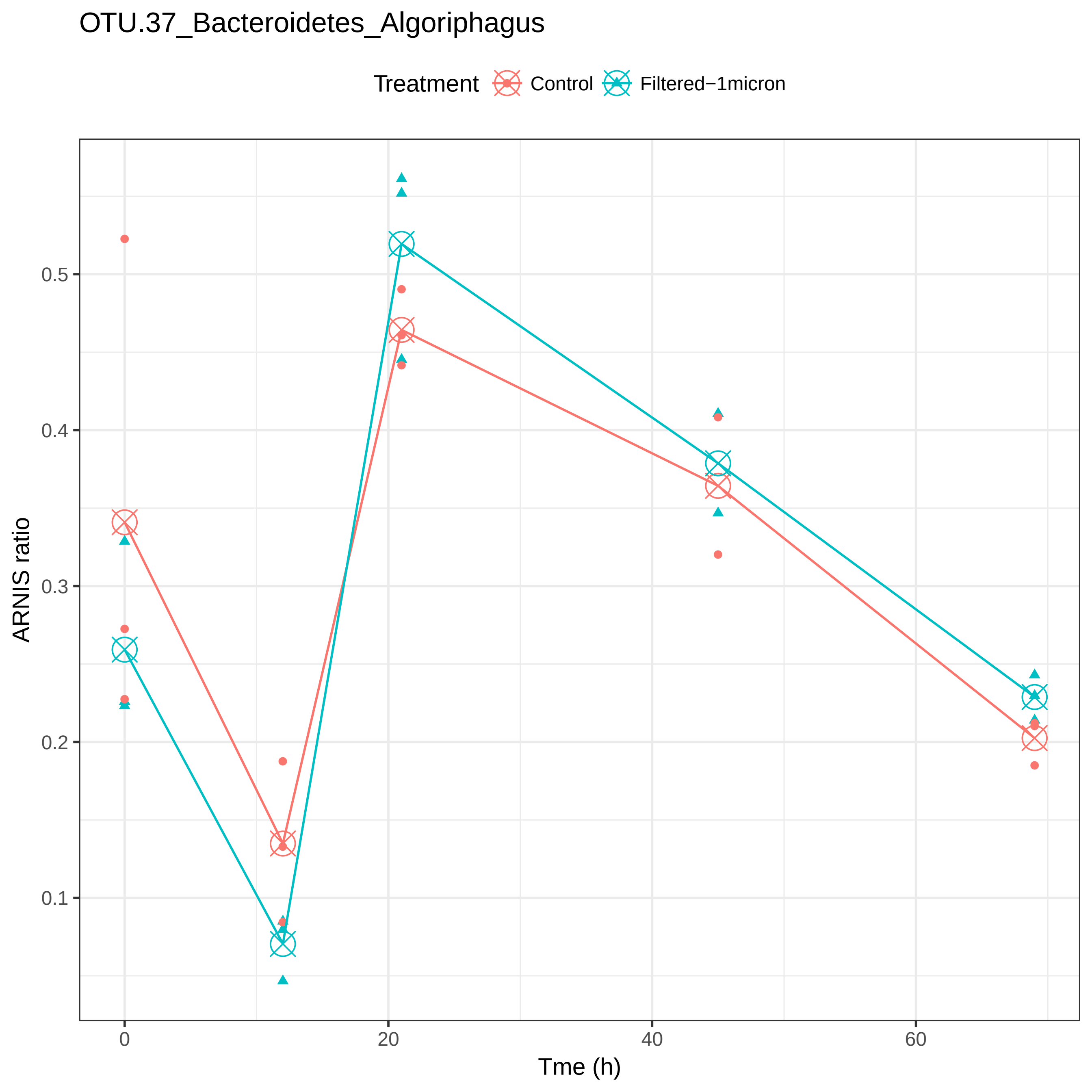

OTU.37_Bacteroidetes_Algoriphagus
Treatment
Control
Filtered−1micron
ARNIS ratio
0.1
0.2
0.3
0.4
0.5
0
20
40
60
Tme (h)

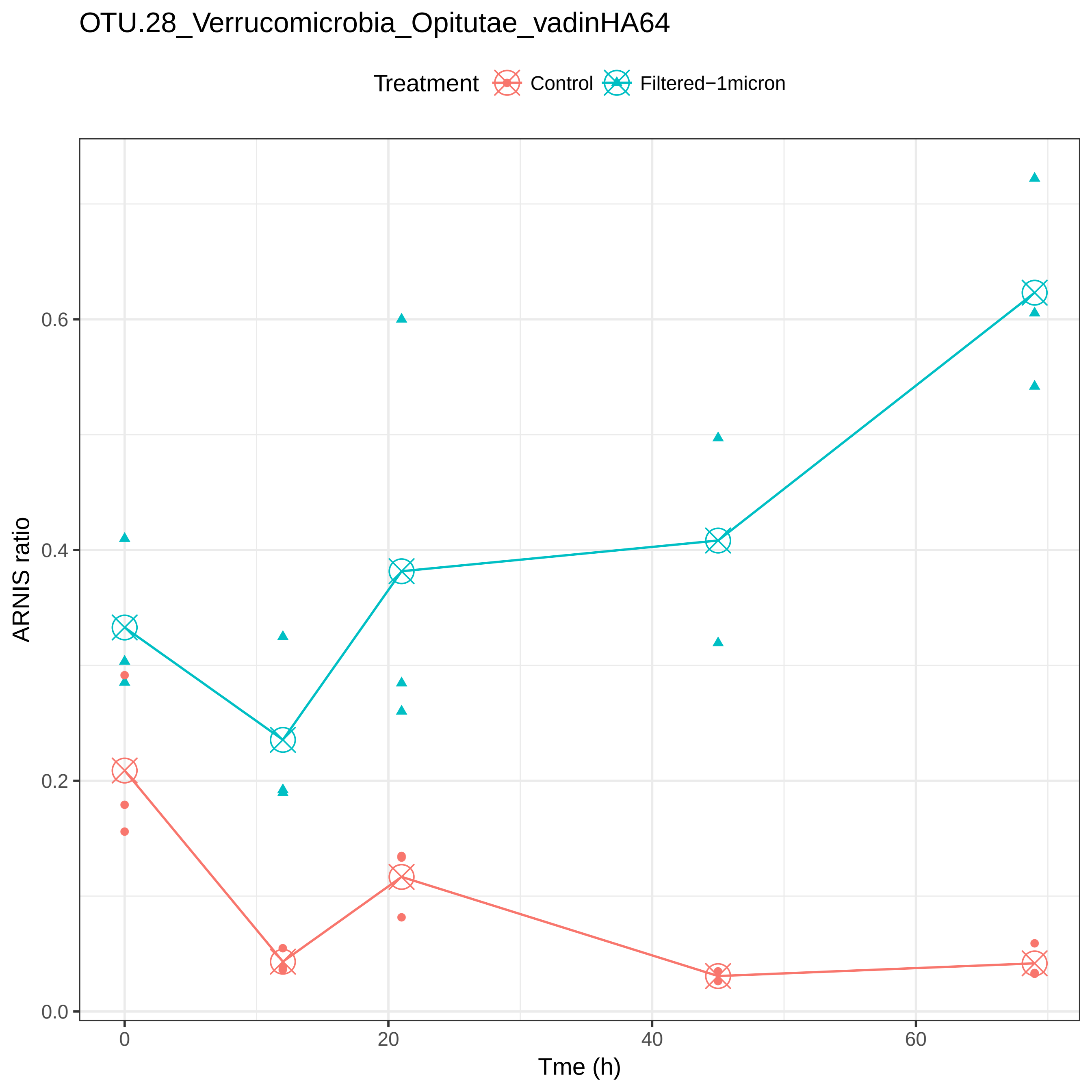

OTU.28_Verrucomicrobia_Opitutae_vadinHA64
Treatment
Control
Filtered−1micron
ARNIS ratio
0.0
0.2
0.4
0.6
0
20
40
60
Tme (h)

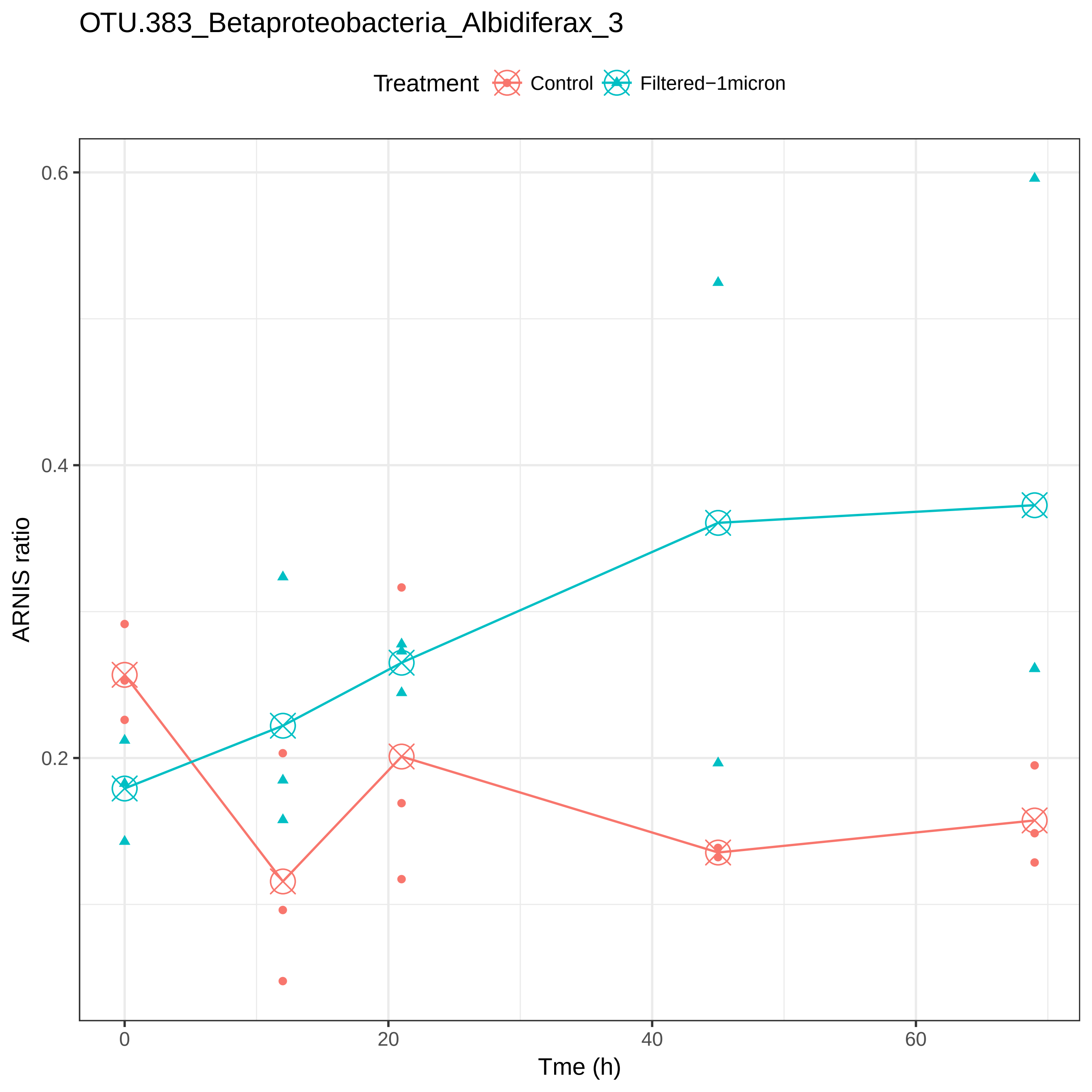
OTU.383_Betaproteobacteria_Albidiferax_3
Treatment
Control
Filtered−1micron
ARNIS ratio
0.6
0.4
0.2
0
20
40
60
Tme (h)

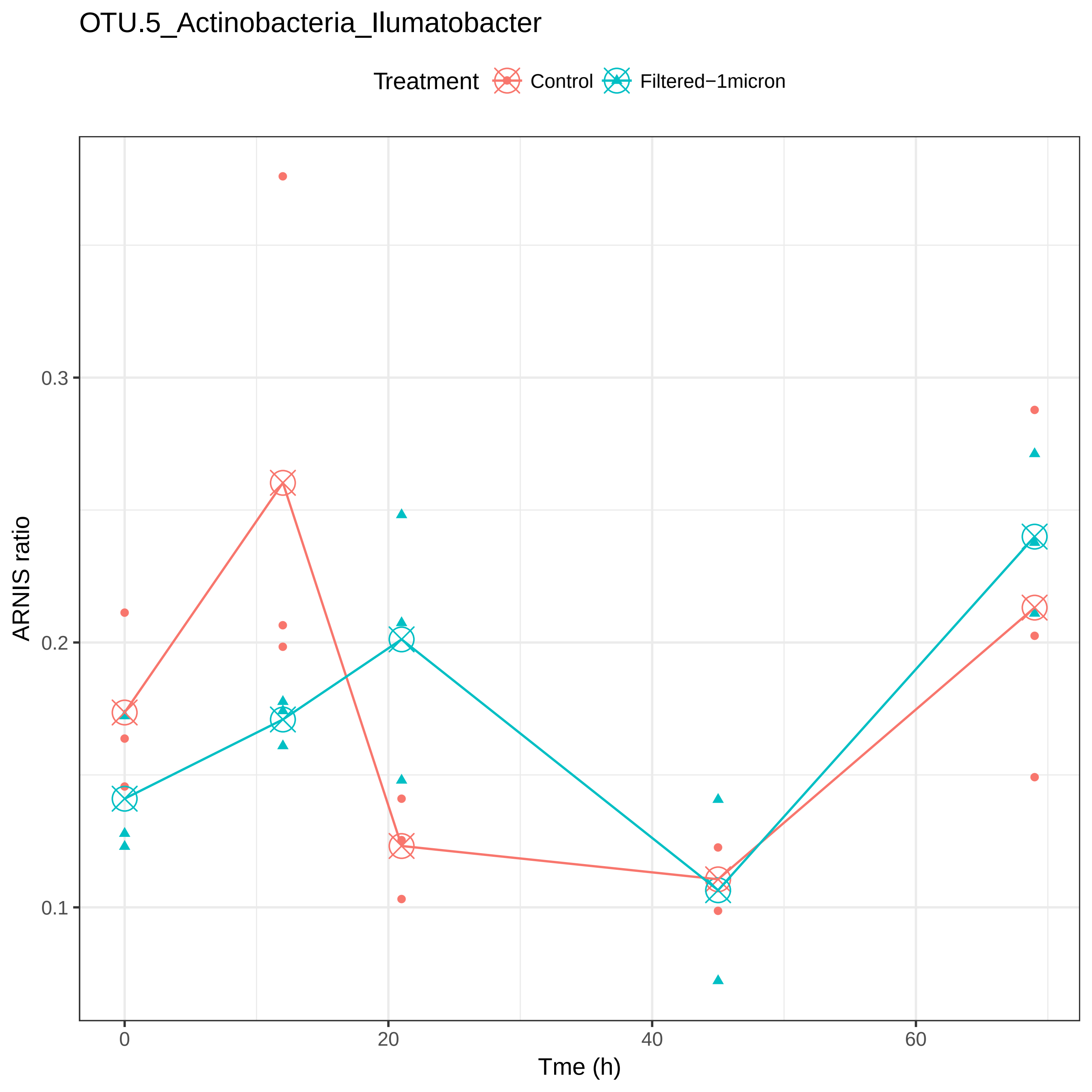

OTU.5_Actinobacteria_Ilumatobacter
Treatment
Control
Filtered−1micron
ARNIS ratio
0.1
0.2
0.3
0
20
40
60
Tme (h)

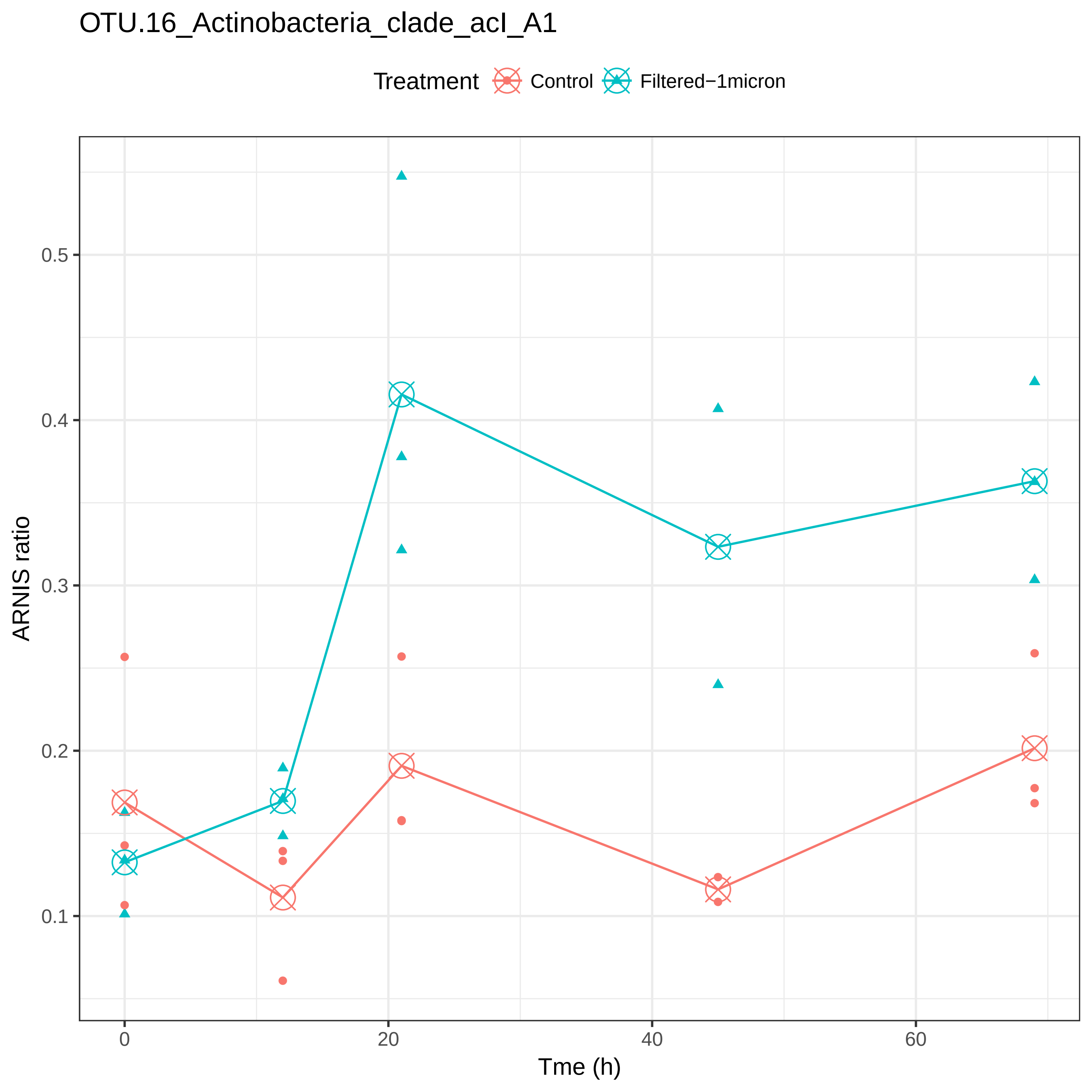

OTU.16_Actinobacteria_clade_acI_A1
Treatment
Control
Filtered−1micron
ARNIS ratio
0.1
0.2
0.3
0.4
0.5
0
20
40
60
Tme (h)

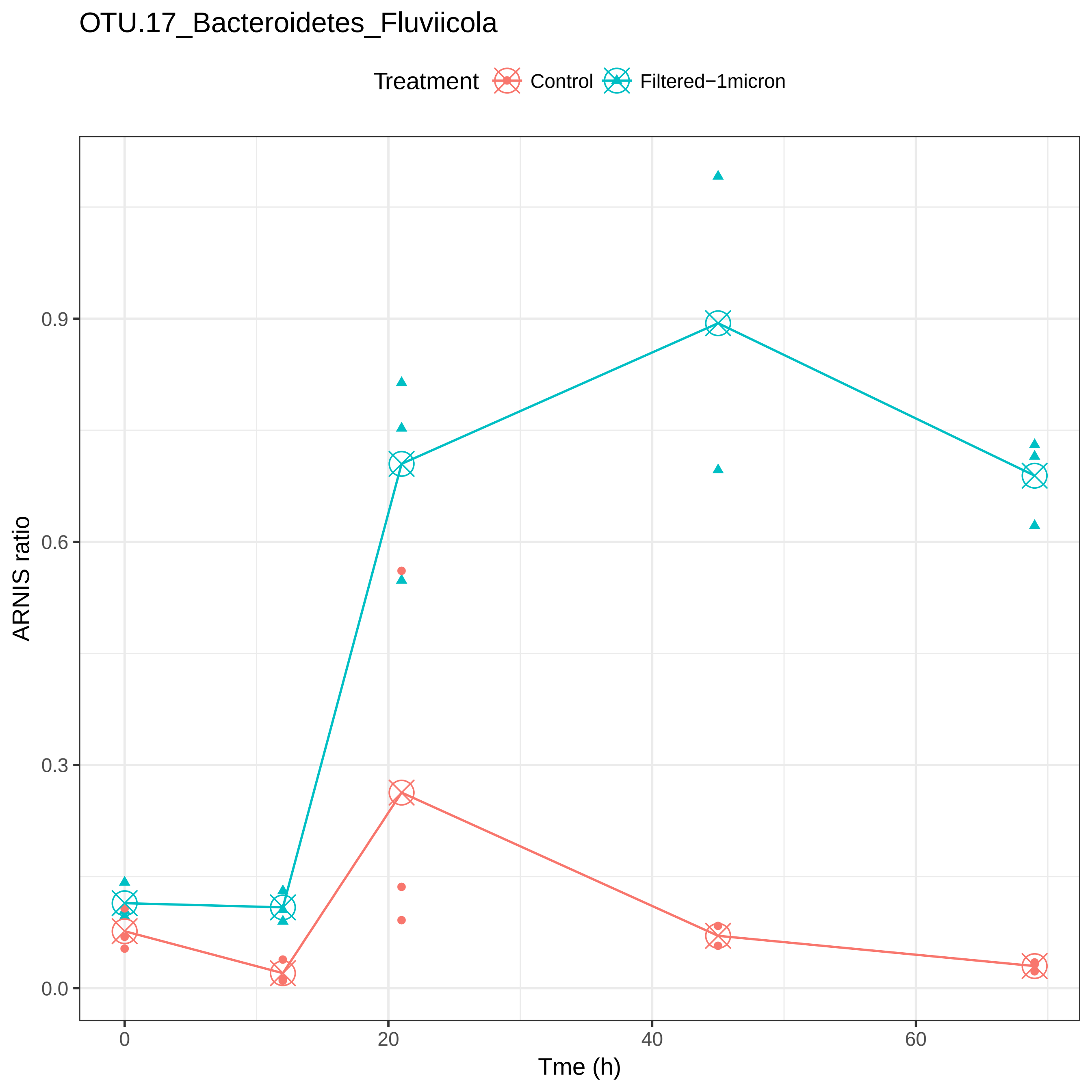
OTU.17_Bacteroidetes_Fluviicola
Treatment
Control
Filtered−1micron
ARNIS ratio
0.0
0.3
0.6
0.9
0
20
40
60
Tme (h)

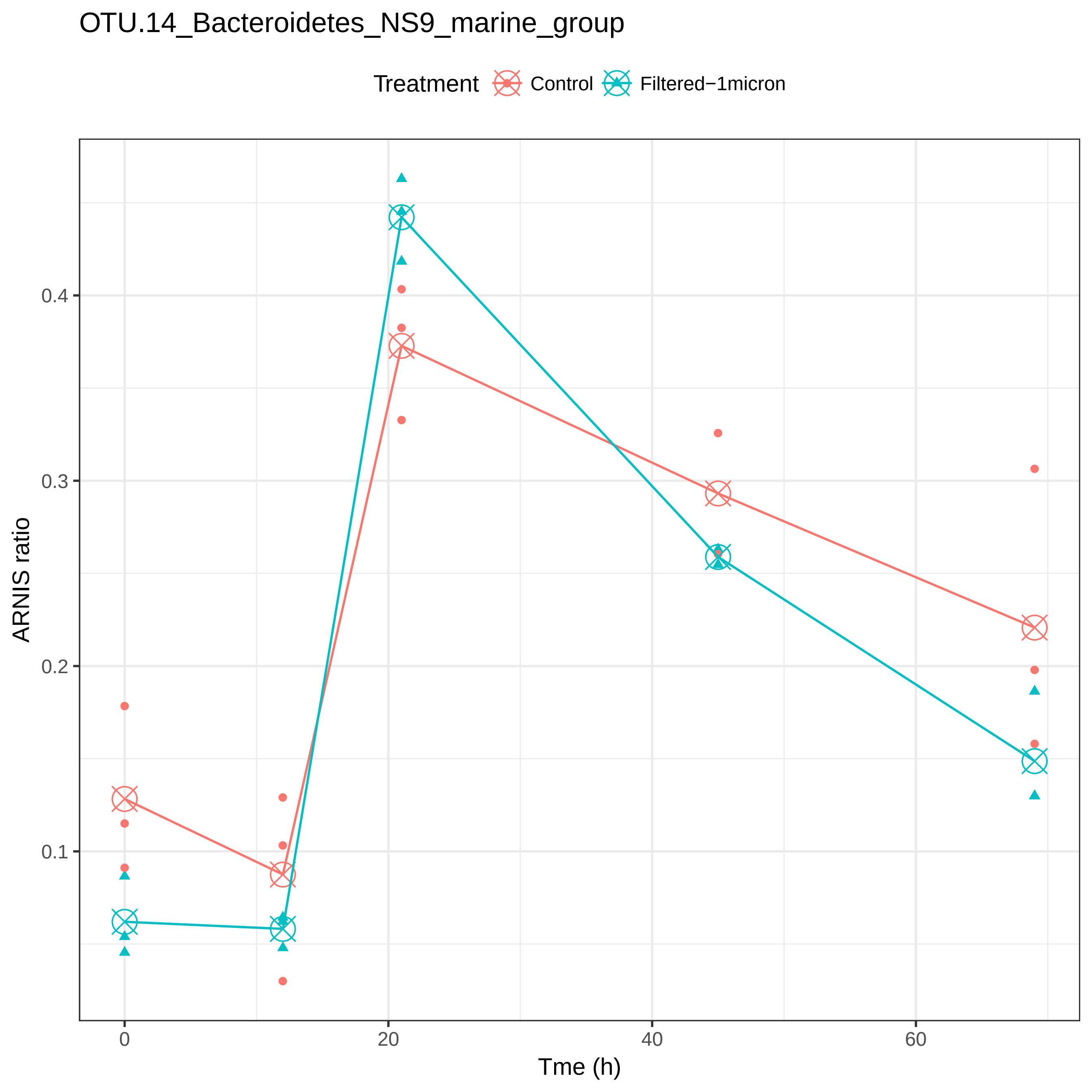
OTU.14_Bacteroidetes_NS9_marine_group
Treatment
Control
Filtered−1micron
ARNIS ratio
0.4
0.3
0.2
0.1
0
20
40
60
Tme (h)

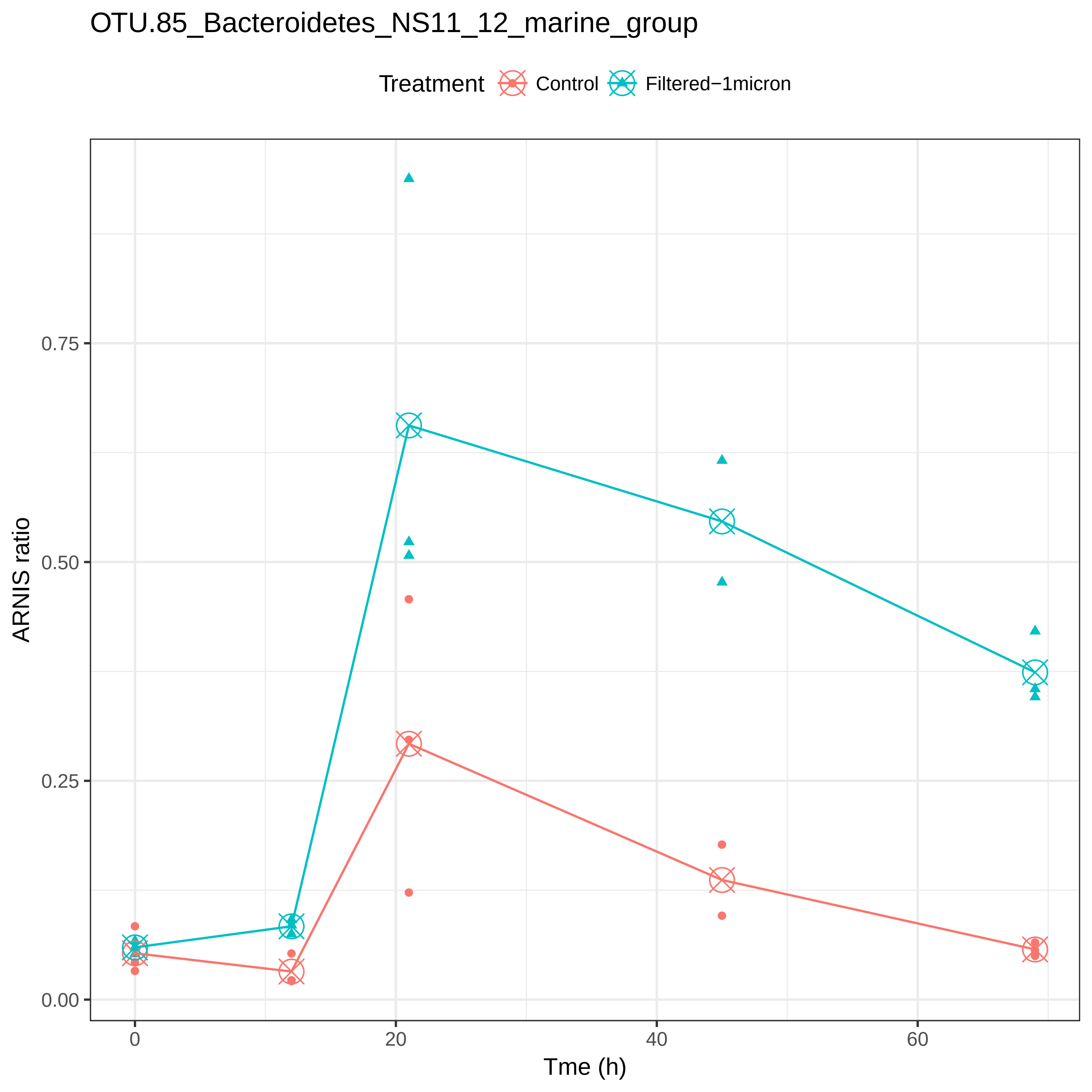
OTU.85_Bacteroidetes_NS11_12_marine_group
Treatment
Control
Filtered−1micron
ARNIS ratio
0.00
0.25
0.50
0.75
0
20
40
60
Tme (h)

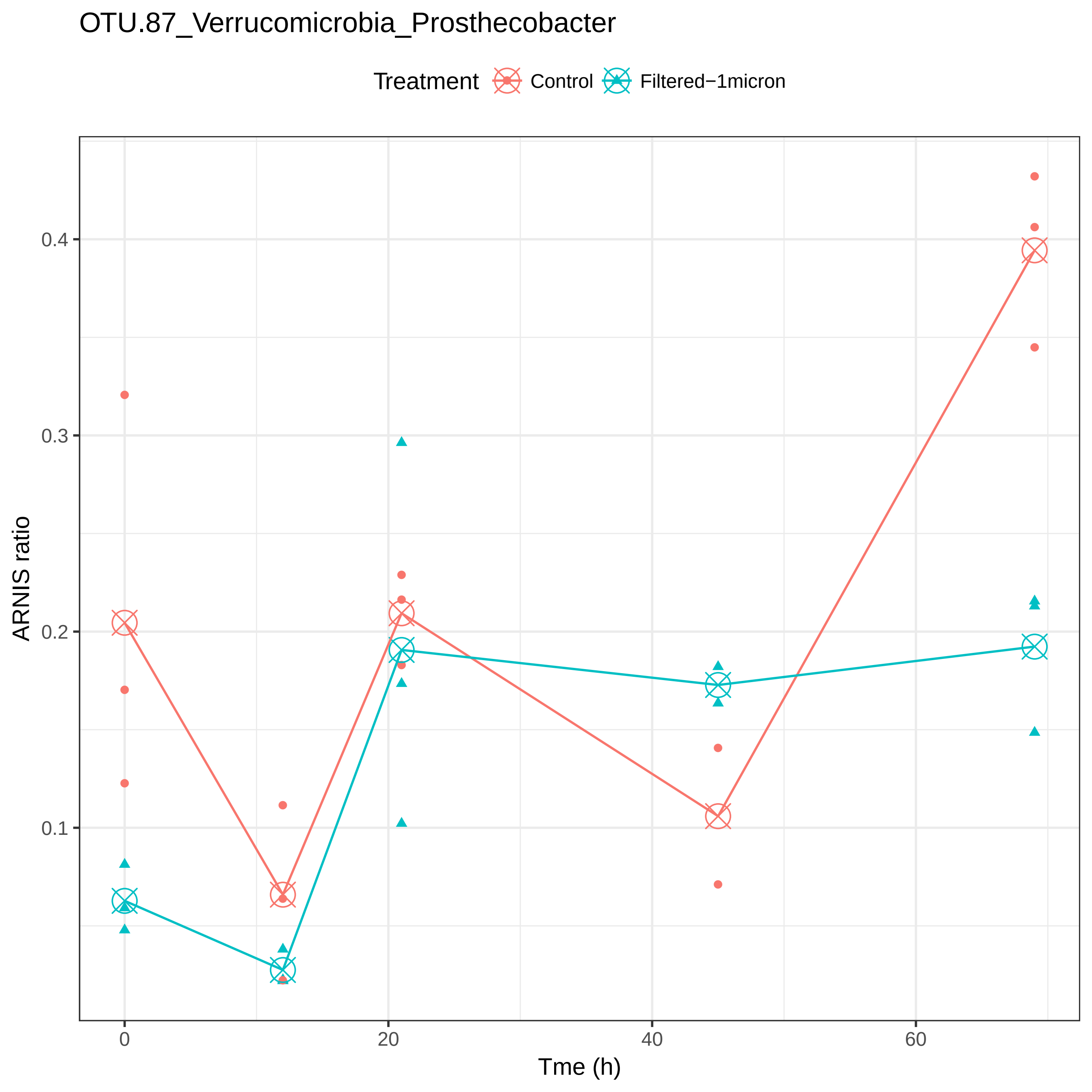
OTU.87_Verrucomicrobia_Prosthecobacter
Treatment
Control
Filtered−1micron
ARNIS ratio
0.4
0.3
0.2
0.1
0
20
40
60
Tme (h)

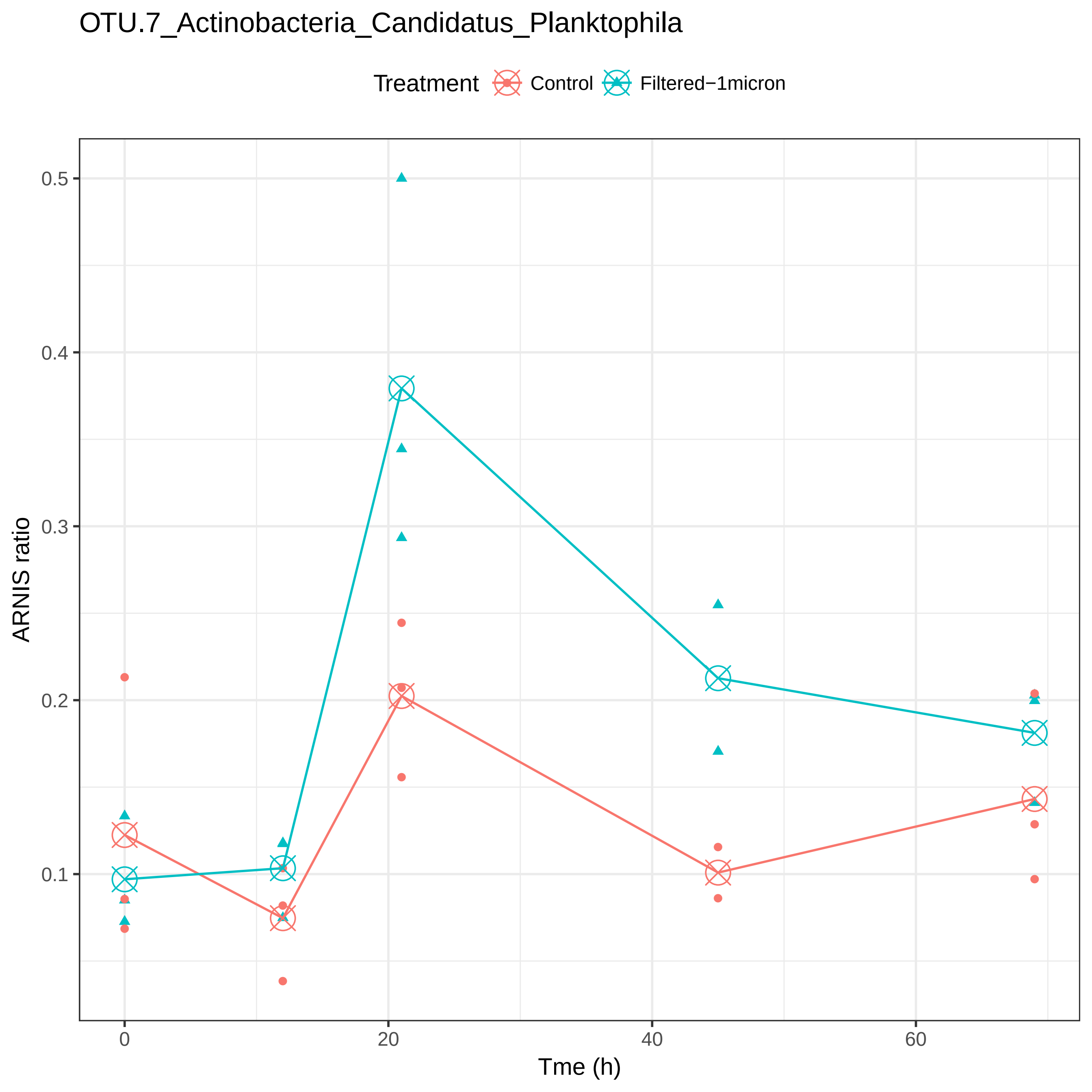

OTU.7_Actinobacteria_Candidatus_Planktophila
Treatment
Control
Filtered−1micron
ARNIS ratio
0.1
0.2
0.3
0.4
0.5
0
20
40
60
Tme (h)

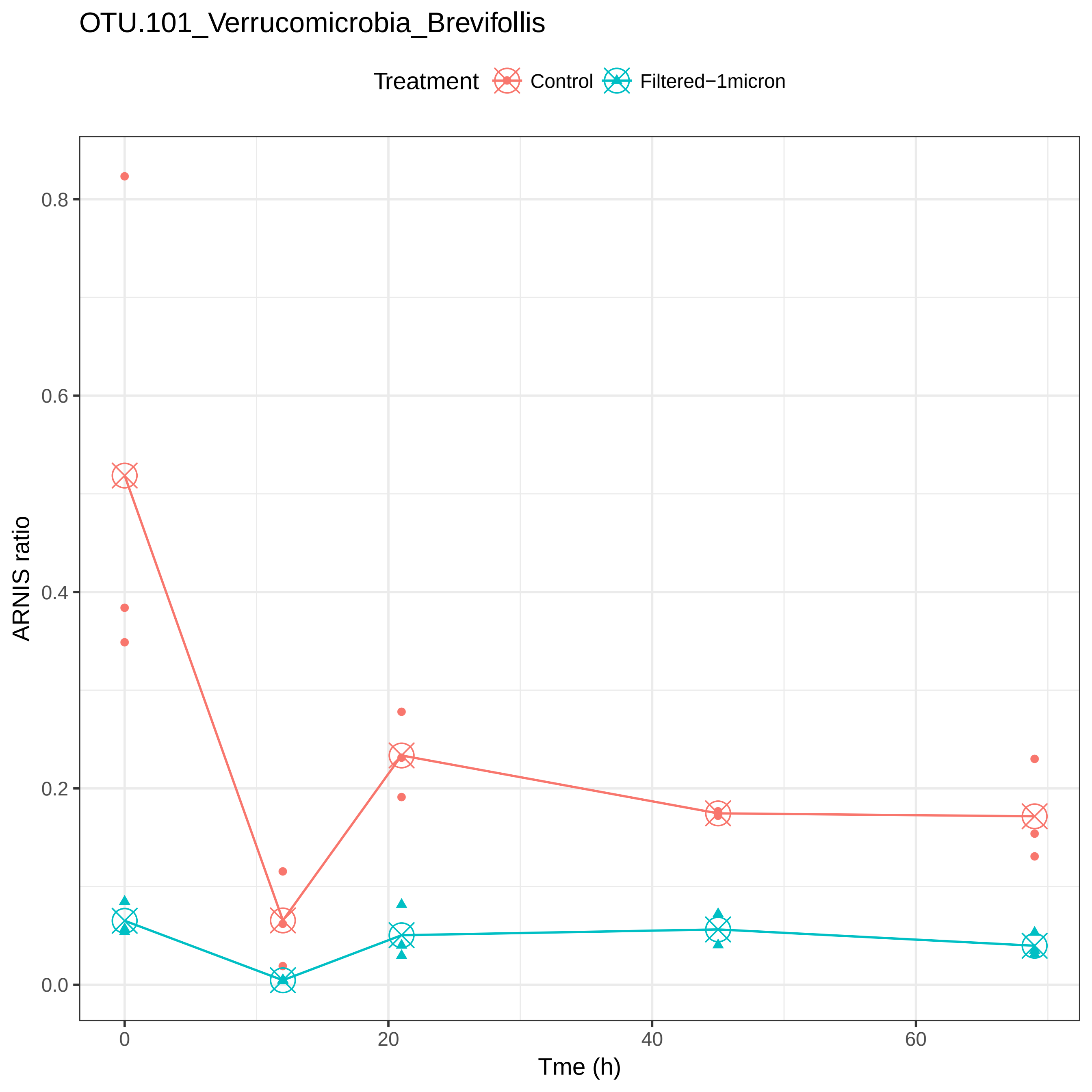

OTU.101_Verrucomicrobia_Brevifollis
Treatment
Control
Filtered−1micron
ARNIS ratio
0.0
0.2
0.4
0.6
0.8
0
20
40
60
Tme (h)

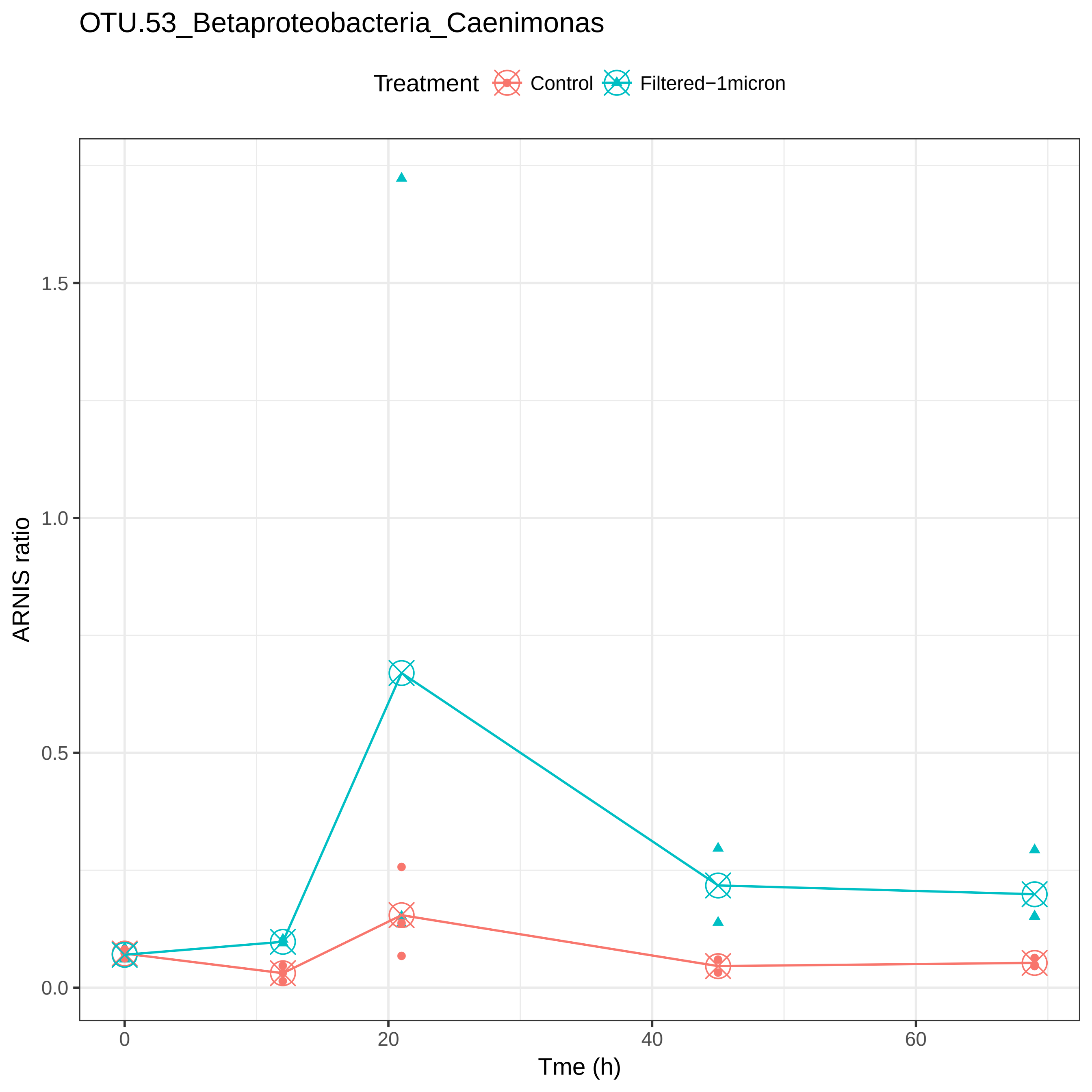

OTU.53_Betaproteobacteria_Caenimonas
Treatment
Control
Filtered−1micron
ARNIS ratio
0.0
0.5
1.0
1.5
0
20
40
60
Tme (h)

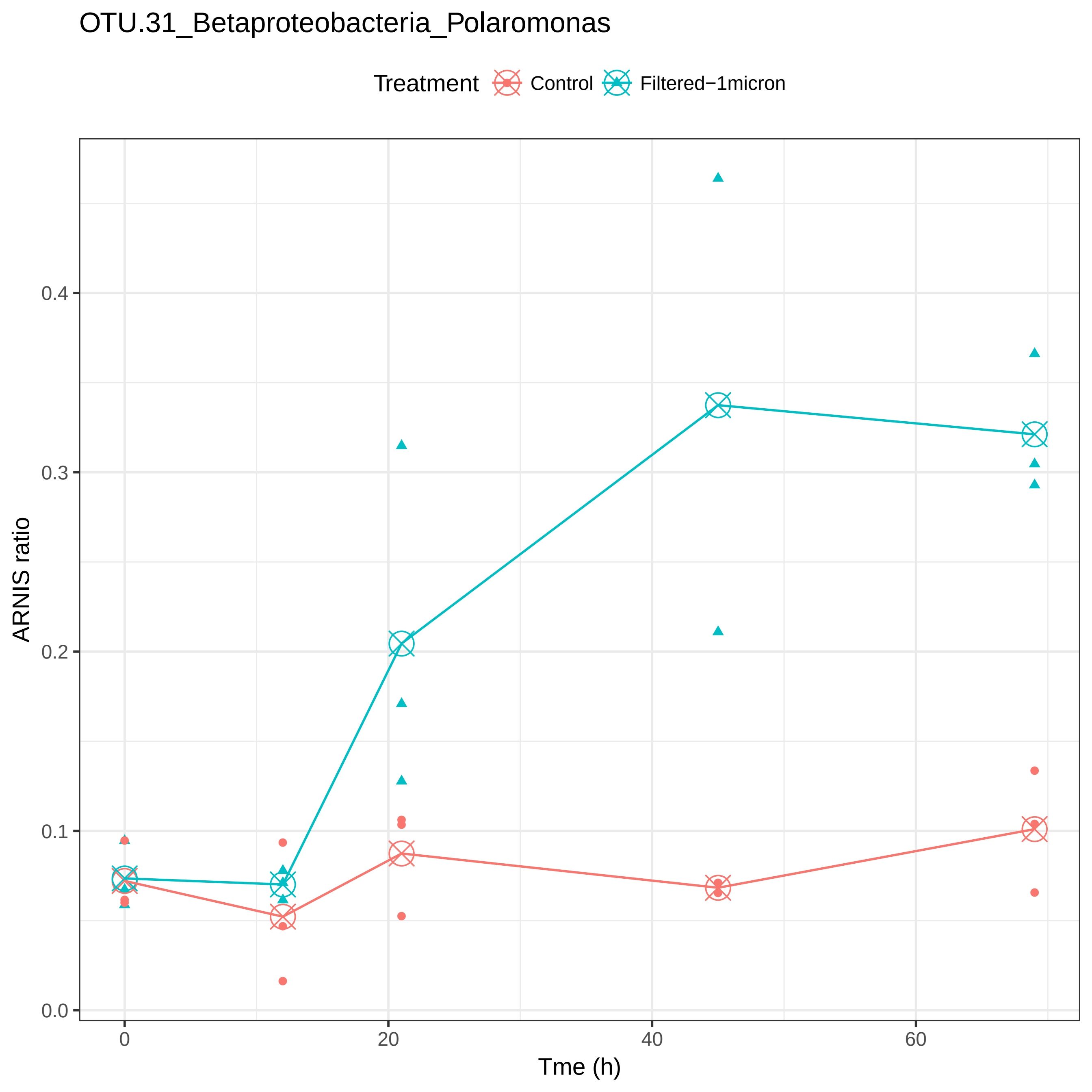
OTU.31_Betaproteobacteria_Polaromonas
Treatment
Control
Filtered−1micron
ARNIS ratio
0.0
0.1
0.2
0.3
0.4
0
20
40
60
Tme (h)

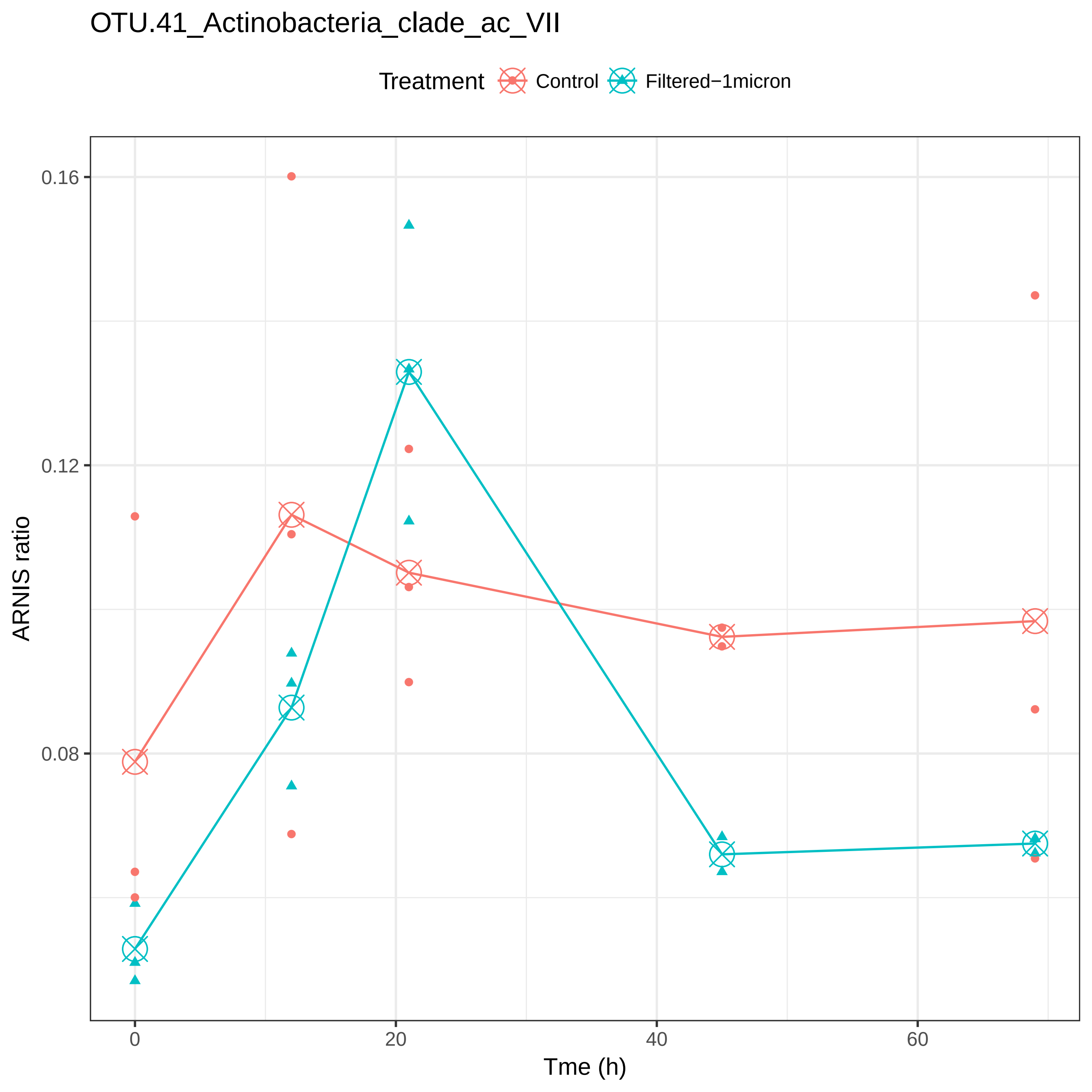

OTU.41_Actinobacteria_clade_ac_VII
Treatment
Control
Filtered−1micron
ARNIS ratio
0.16
0.12
0.08
0
20
40
60
Tme (h)

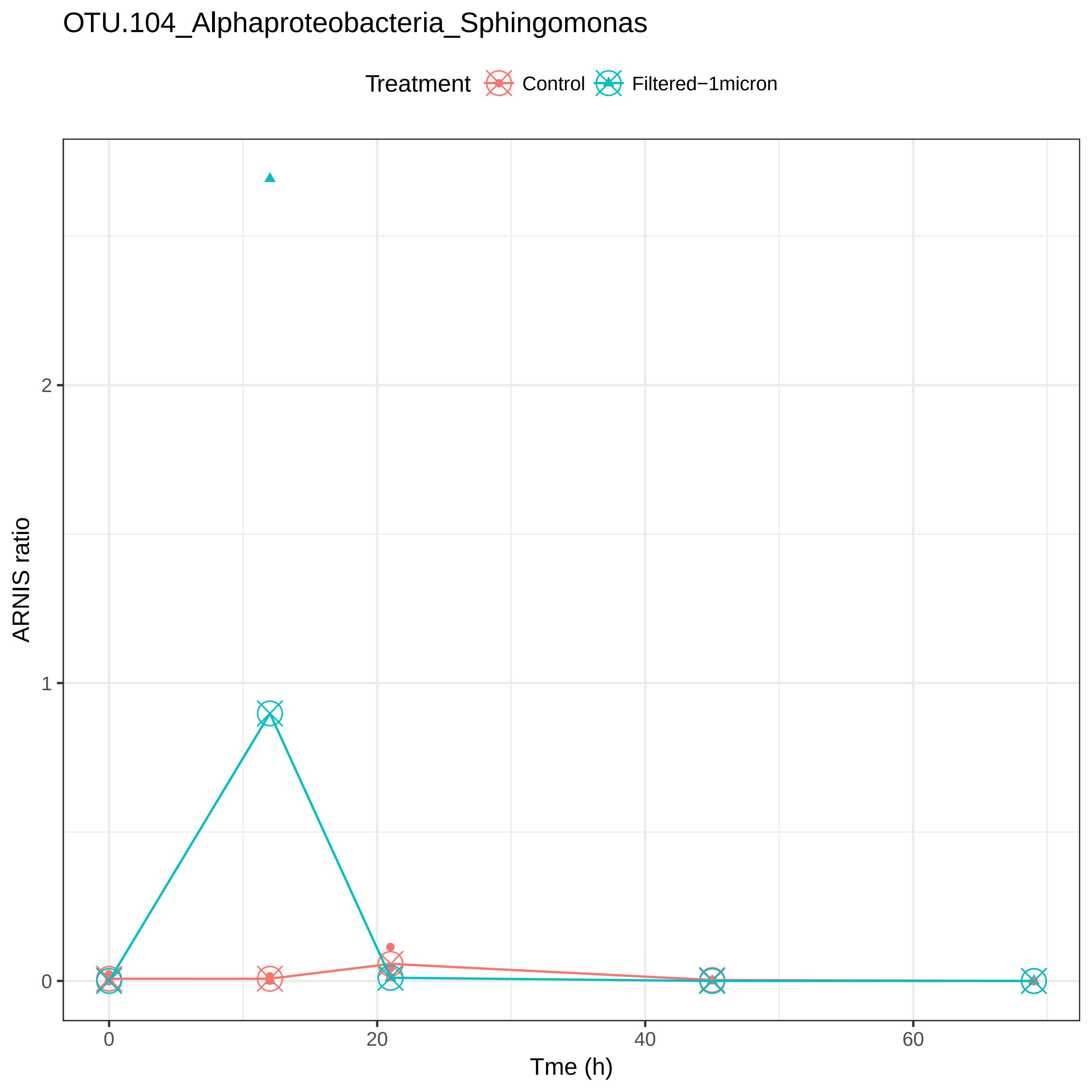

OTU.104_Alphaproteobacteria_Sphingomonas
Treatment
Control
Filtered−1micron
ARNIS ratio
2
1
0
0
20
40
60
Tme (h)

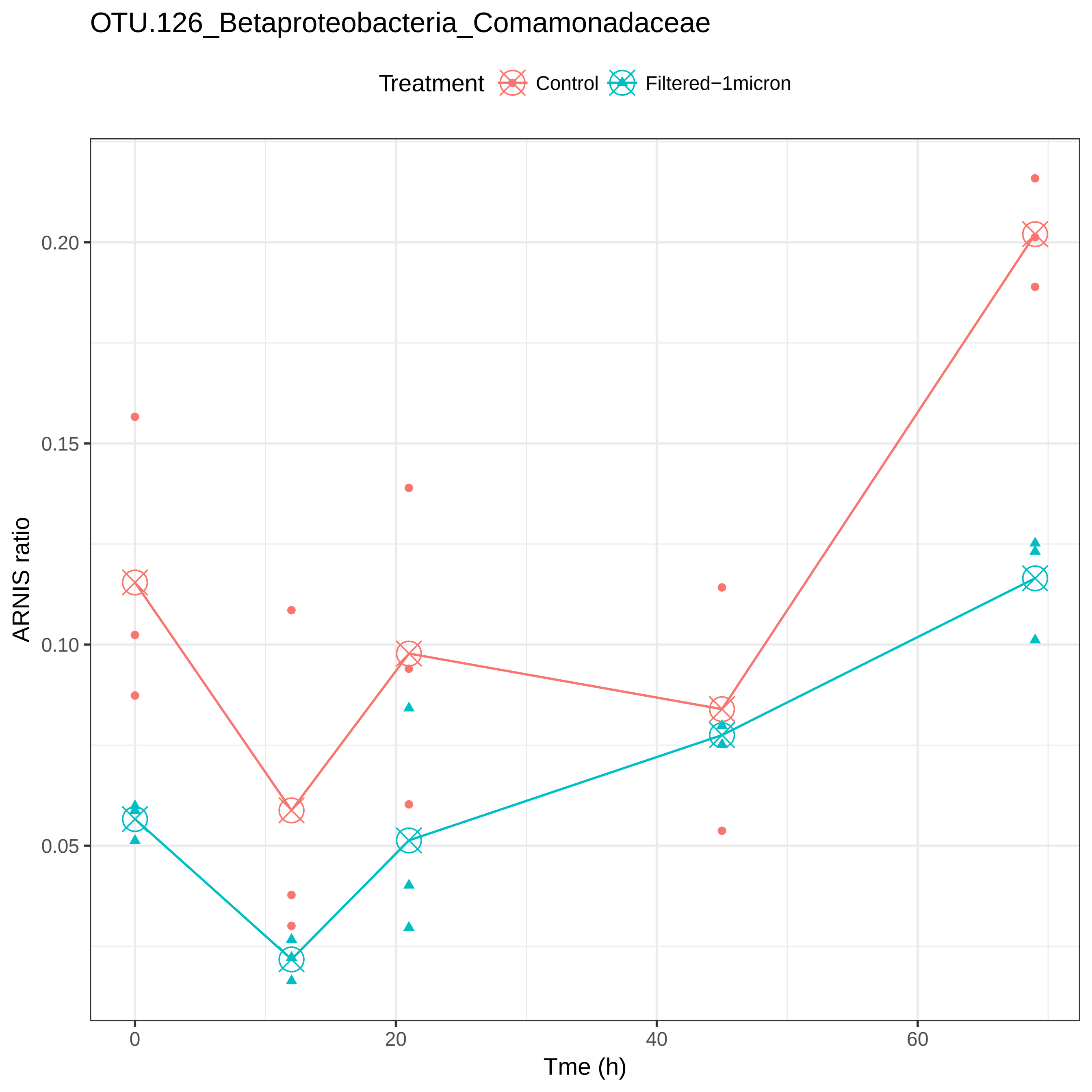
OTU.126_Betaproteobacteria_Comamonadaceae
Treatment
Control
Filtered−1micron
ARNIS ratio
0.20
0.15
0.10
0.05
0
20
40
60
Tme (h)

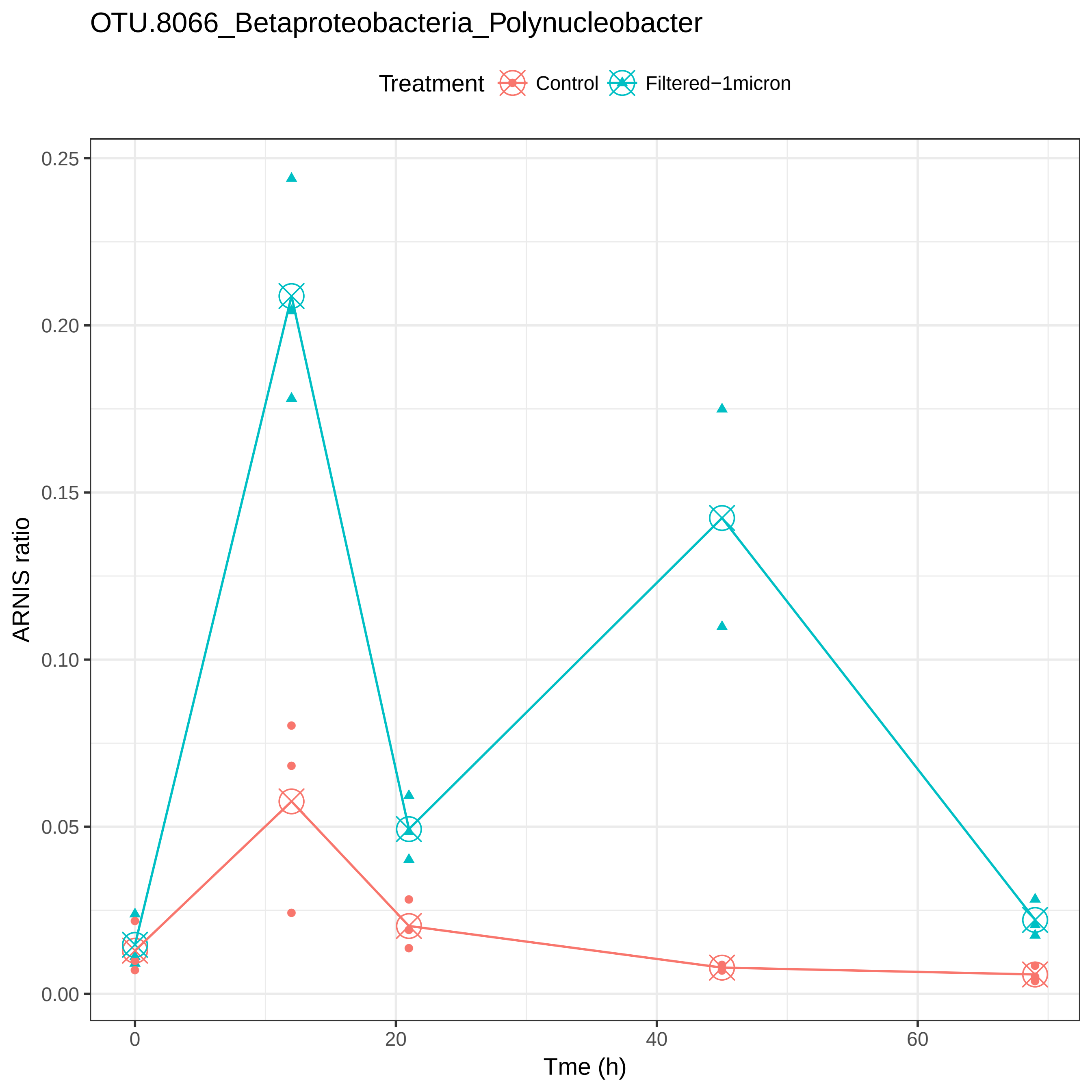
OTU.8066_Betaproteobacteria_Polynucleobacter
Treatment
Control
Filtered−1micron
ARNIS ratio
0.25
0.20
0.15
0.10
0.05
0.00
0
20
40
60
Tme (h)

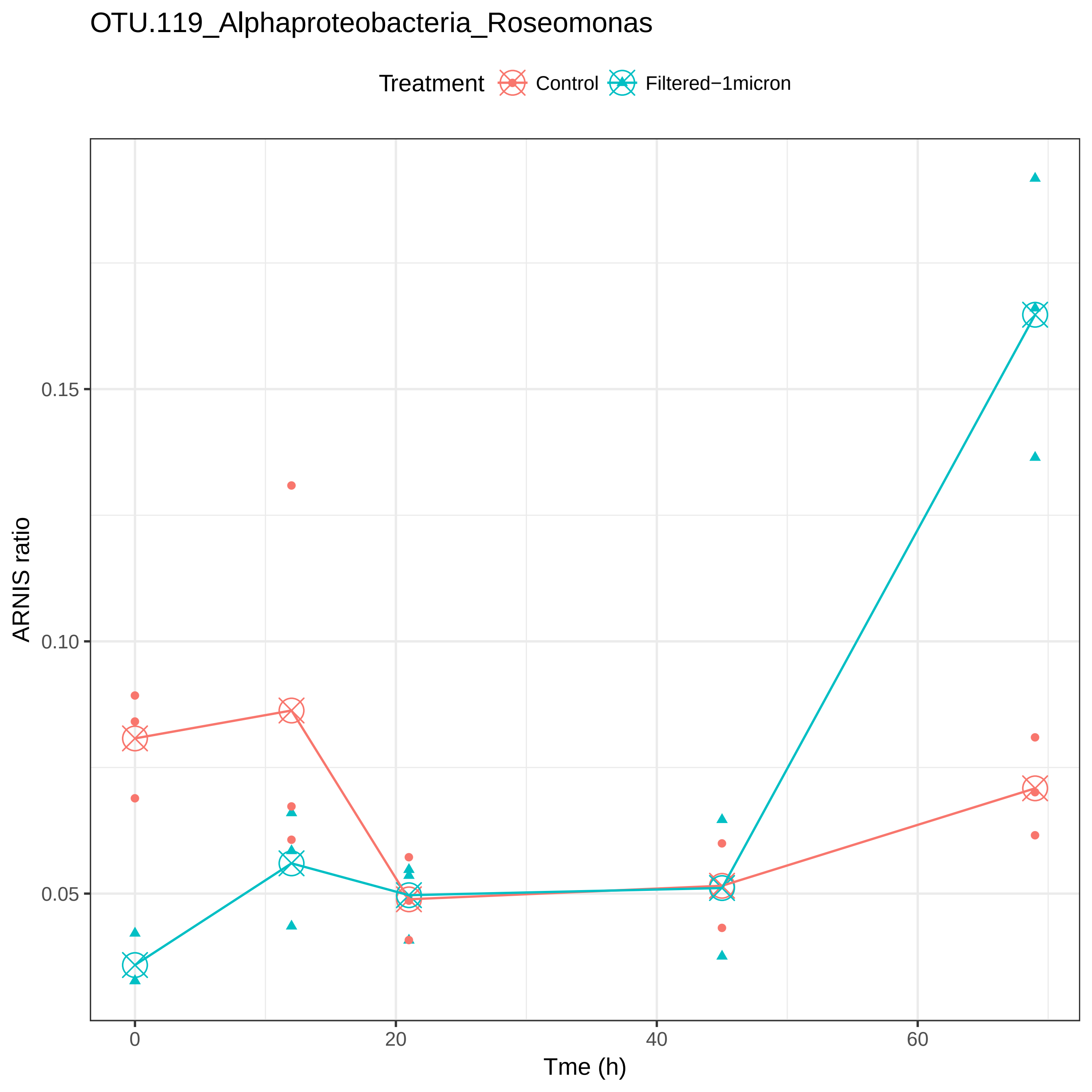

OTU.119_Alphaproteobacteria_Roseomonas
Treatment
Control
Filtered−1micron
ARNIS ratio
0.15
0.10
0.05
0
20
40
60
Tme (h)

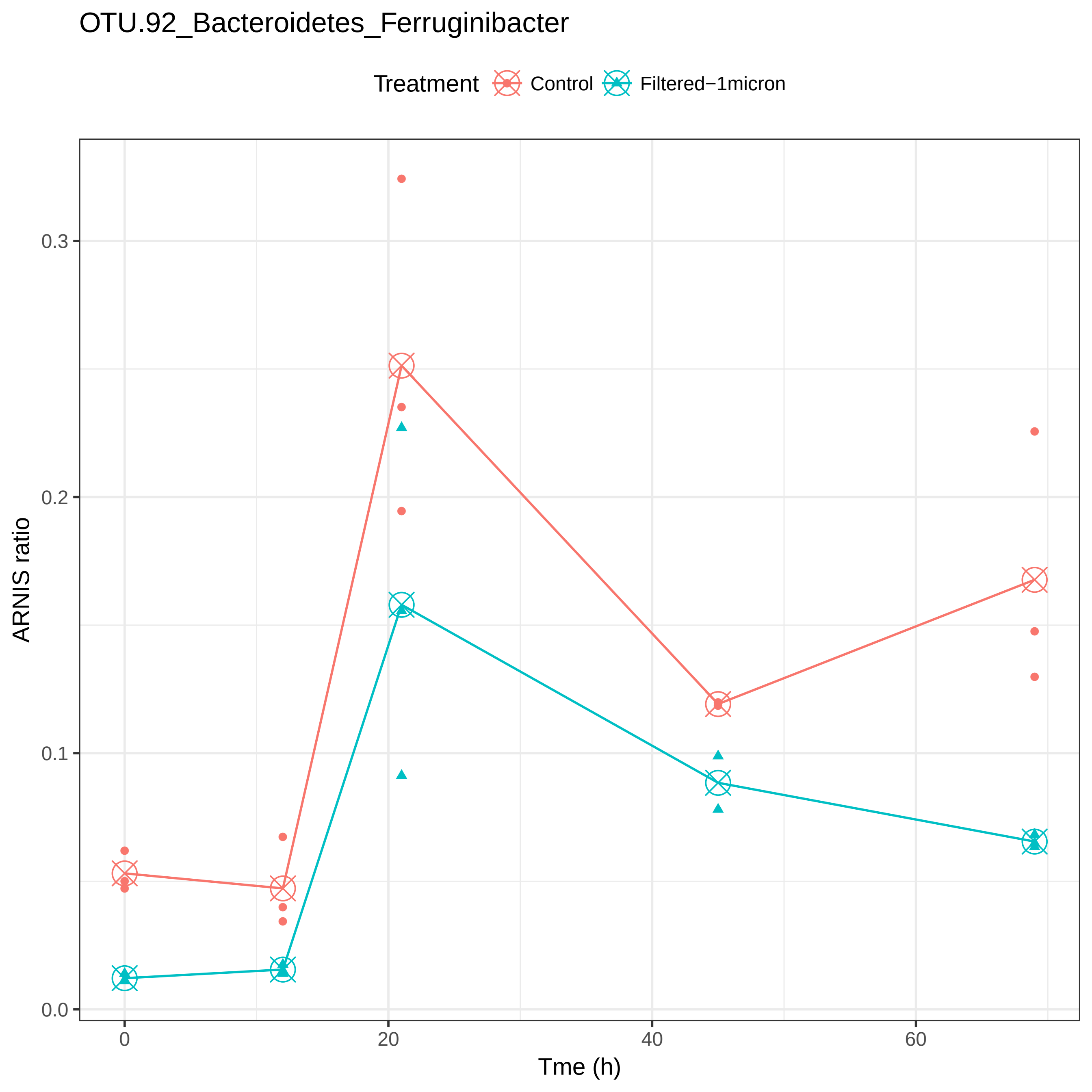

OTU.92_Bacteroidetes_Ferruginibacter
Treatment
Control
Filtered−1micron
ARNIS ratio
0.0
0.1
0.2
0.3
0
20
40
60
Tme (h)

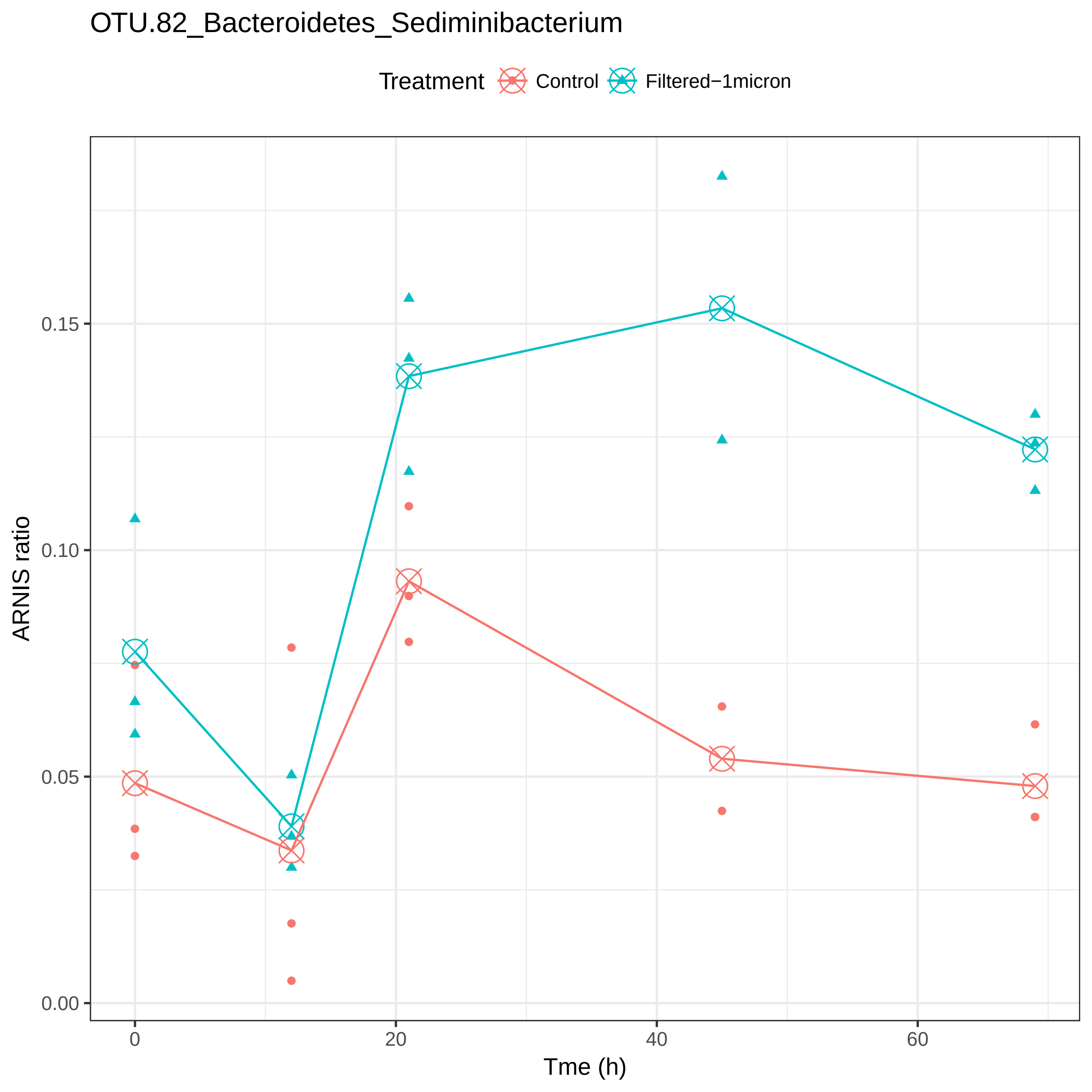

OTU.82_Bacteroidetes_Sediminibacterium
Treatment
Control
Filtered−1micron
ARNIS ratio
0.00
0.05
0.10
0.15
0
20
40
60
Tme (h)

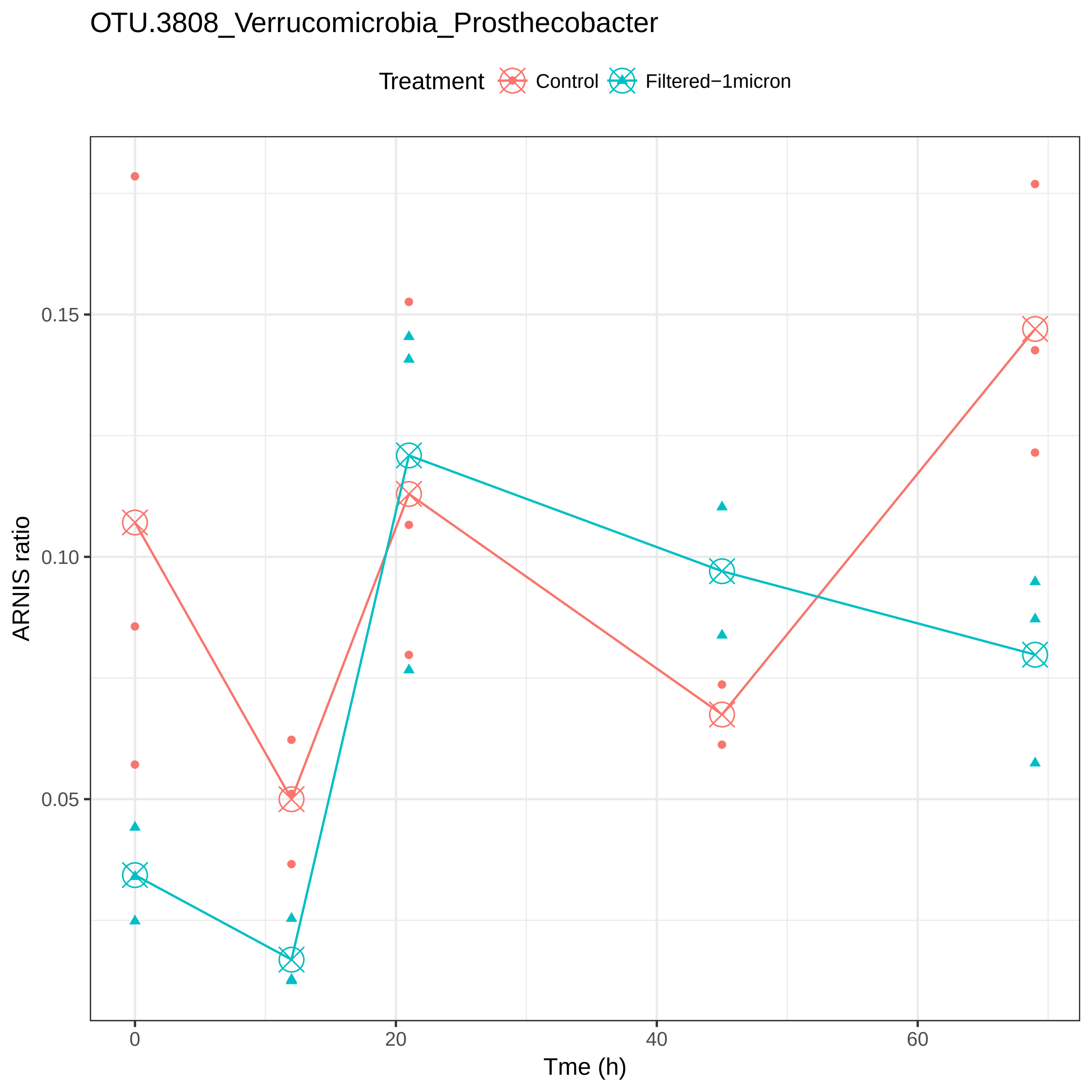
OTU.3808_Verrucomicrobia_Prosthecobacter
Treatment
Control
Filtered−1micron
ARNIS ratio
0.15
0.10
0.05
0
20
40
60
Tme (h)

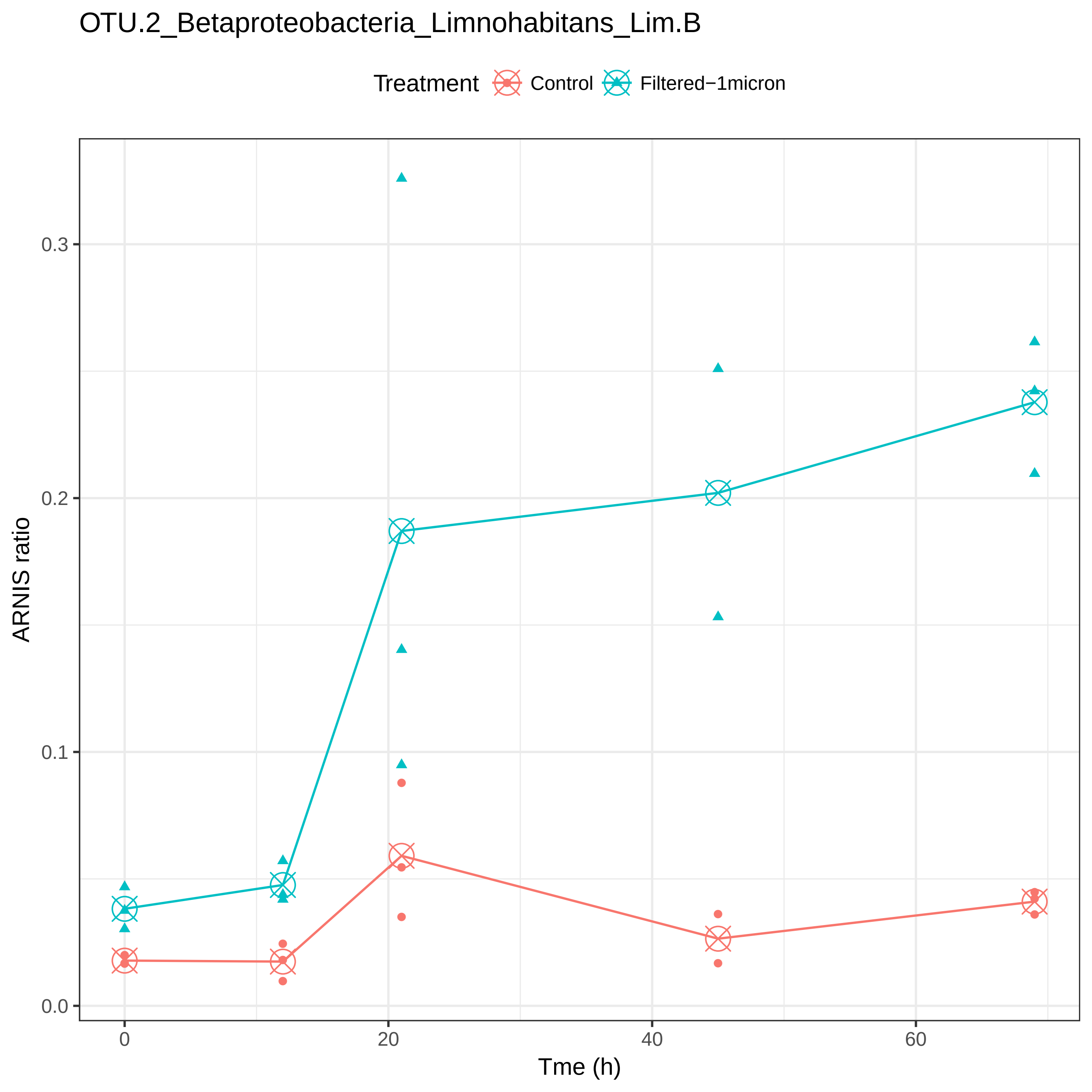
OTU.2_Betaproteobacteria_Limnohabitans_Lim.B
Treatment
Control
Filtered−1micron
ARNIS ratio
0.0
0.1
0.2
0.3
0
20
40
60
Tme (h)

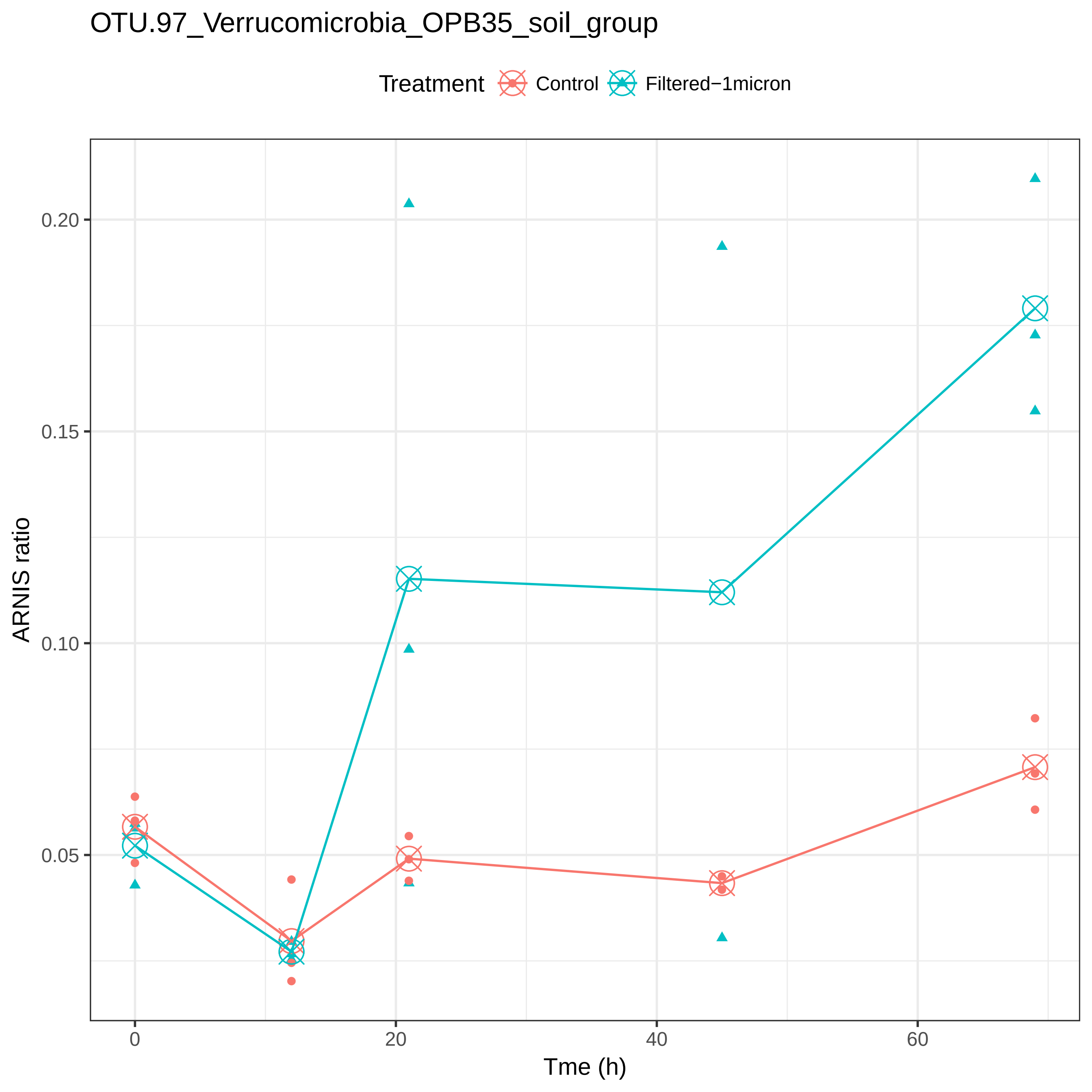

OTU.97_Verrucomicrobia_OPB35_soil_group
Treatment
Control
Filtered−1micron
ARNIS ratio
0.20
0.15
0.10
0.05
0
20
40
60
Tme (h)

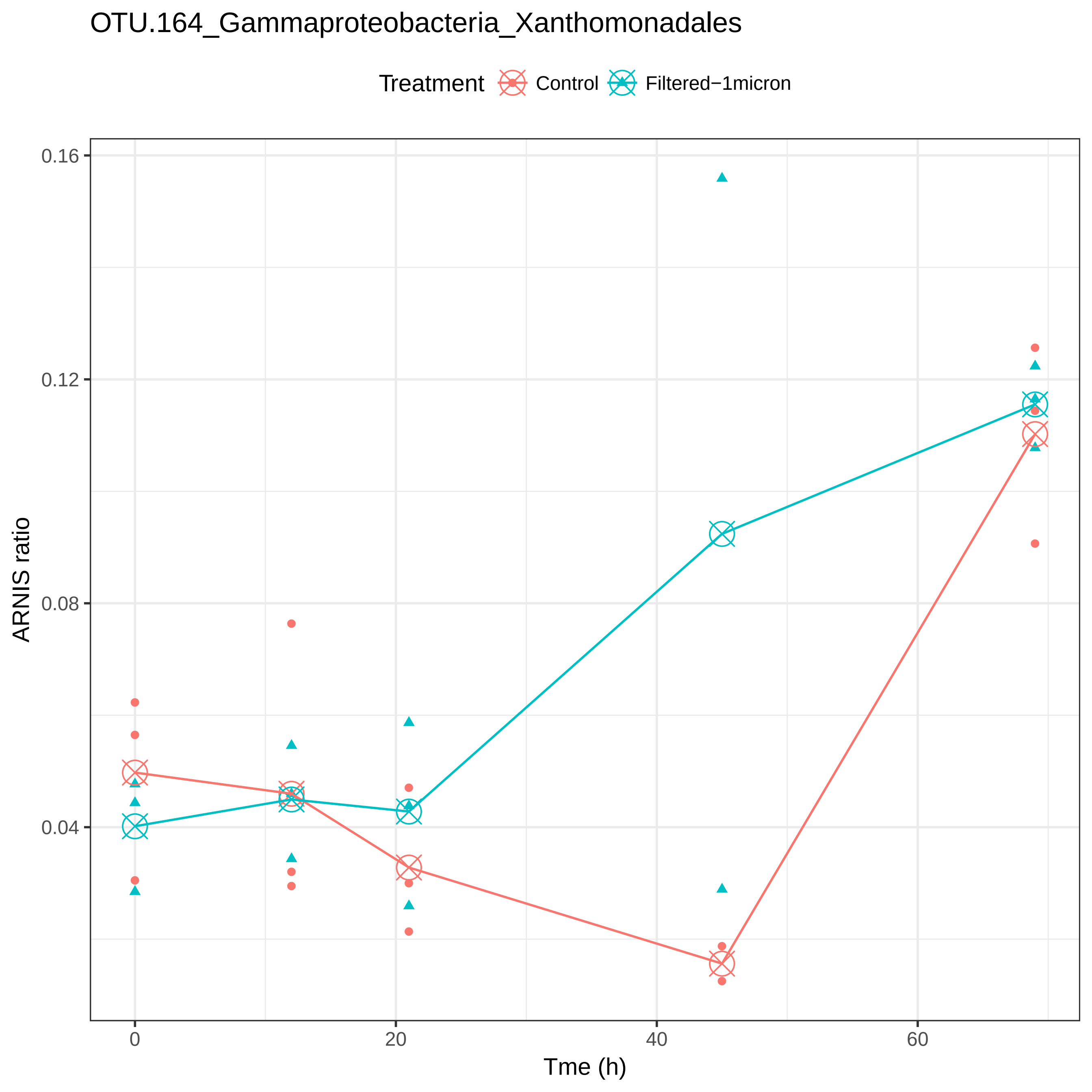

OTU.164_Gammaproteobacteria_Xanthomonadales
Treatment
Control
Filtered−1micron
0.16
0.12
0.08
0.04
ARNIS ratio
0
20
40
60
Tme (h)

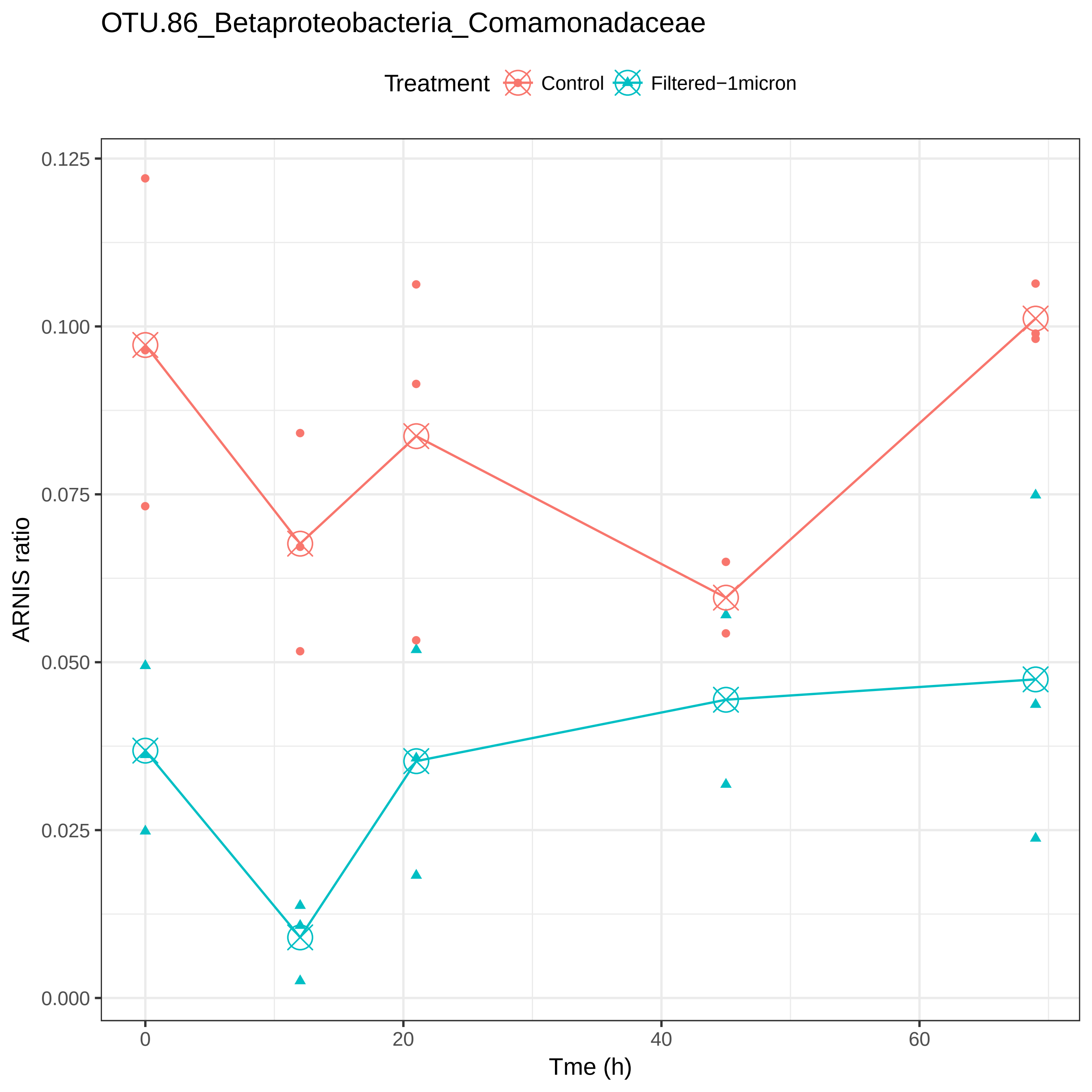
OTU.86_Betaproteobacteria_Comamonadaceae
Treatment
Control
Filtered−1micron
0.125
0.100
0.075
0.050
0.025
0.000
ARNIS ratio
0
20
40
60
Tme (h)

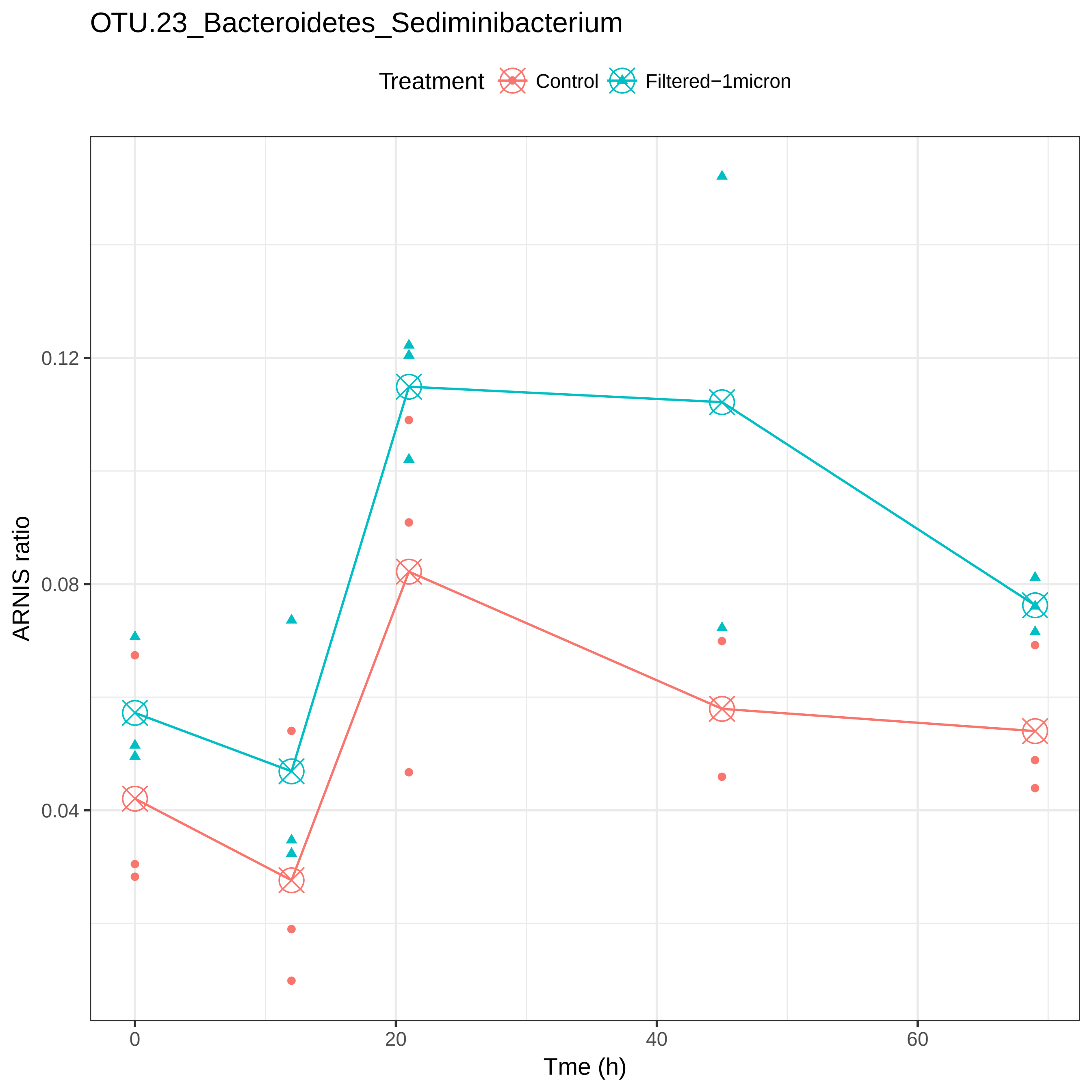
OTU.23_Bacteroidetes_Sediminibacterium
Treatment
Control
Filtered−1micron
0.12
0.08
0.04
ARNIS ratio
0
20
40
60
Tme (h)

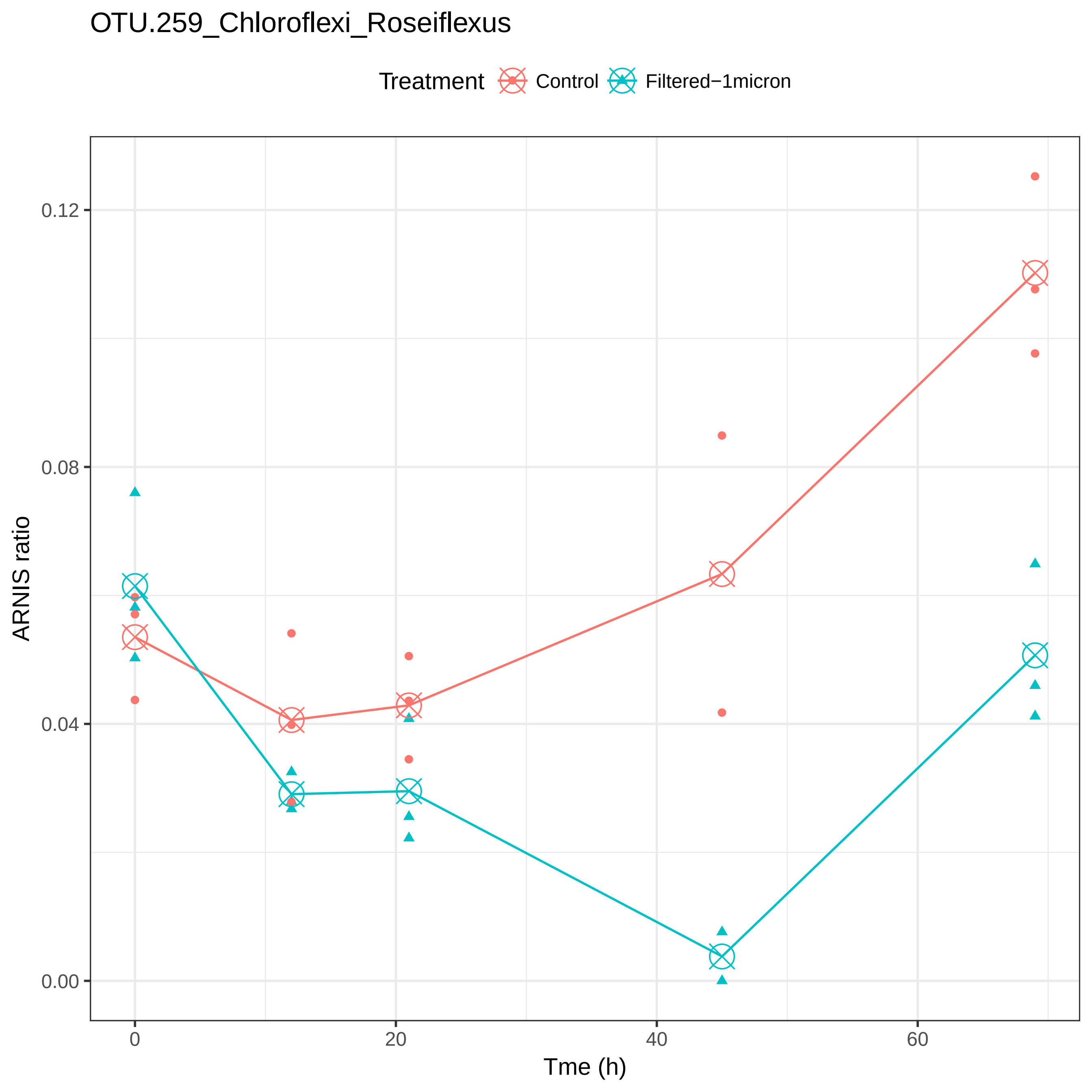

OTU.259_Chloroflexi_Roseiflexus
Treatment
Control
Filtered−1micron
ARNIS ratio
0.12
0.08
0.04
0.00
0
20
40
60
Tme (h)

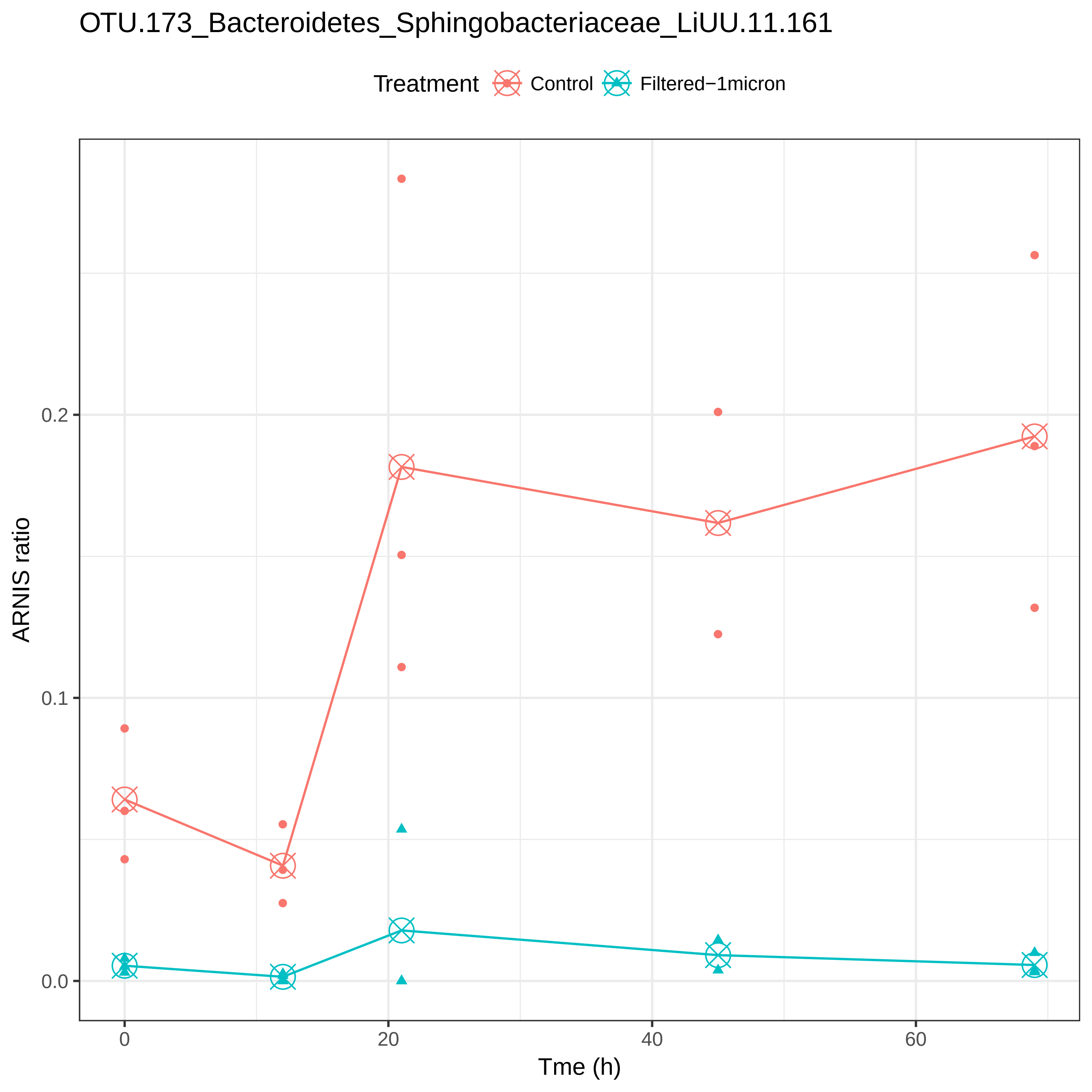

OTU.173_Bacteroidetes_Sphingobacteriaceae_LiUU.11.161
Treatment
Control
Filtered−1micron
ARNIS ratio
0.2
0.1
0.0
0
20
40
60
Tme (h)

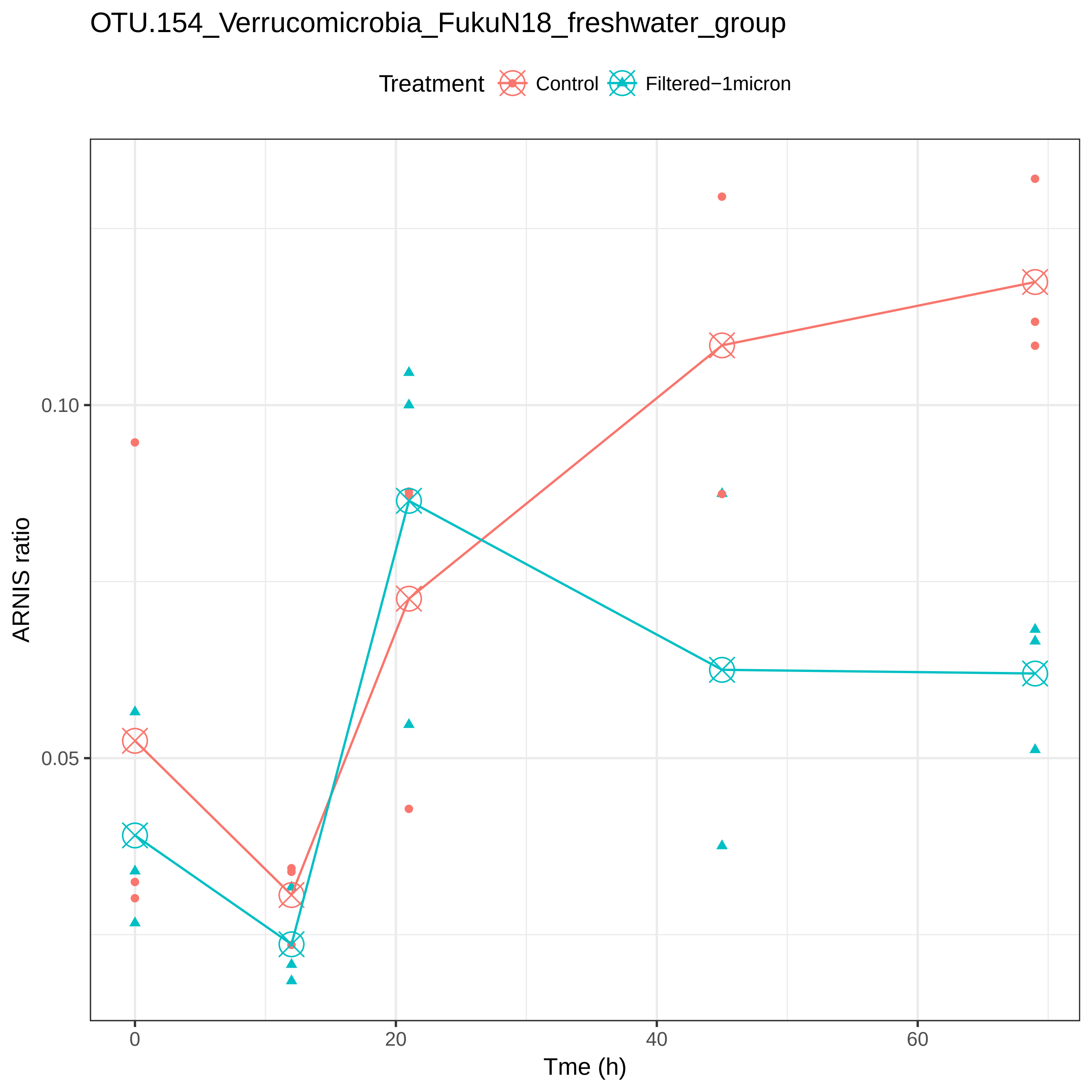

OTU.154_Verrucomicrobia_FukuN18_freshwater_group
Treatment
Control
Filtered−1micron
ARNIS ratio
0.10
0.05
0
20
40
60
Tme (h)

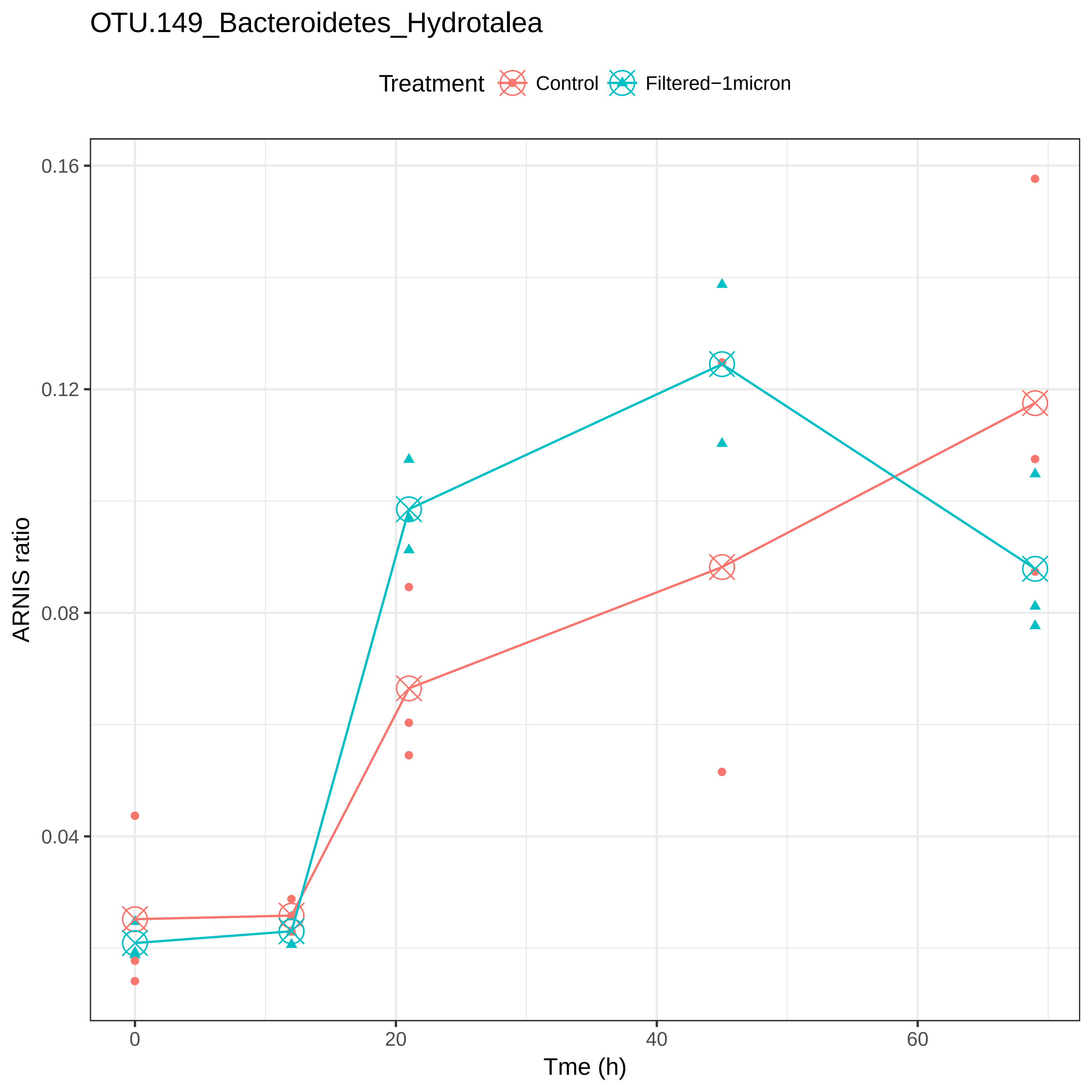
OTU.149_Bacteroidetes_Hydrotalea
Treatment
Control
Filtered−1micron
ARNIS ratio
0.16
0.12
0.08
0.04
0
20
40
60
Tme (h)

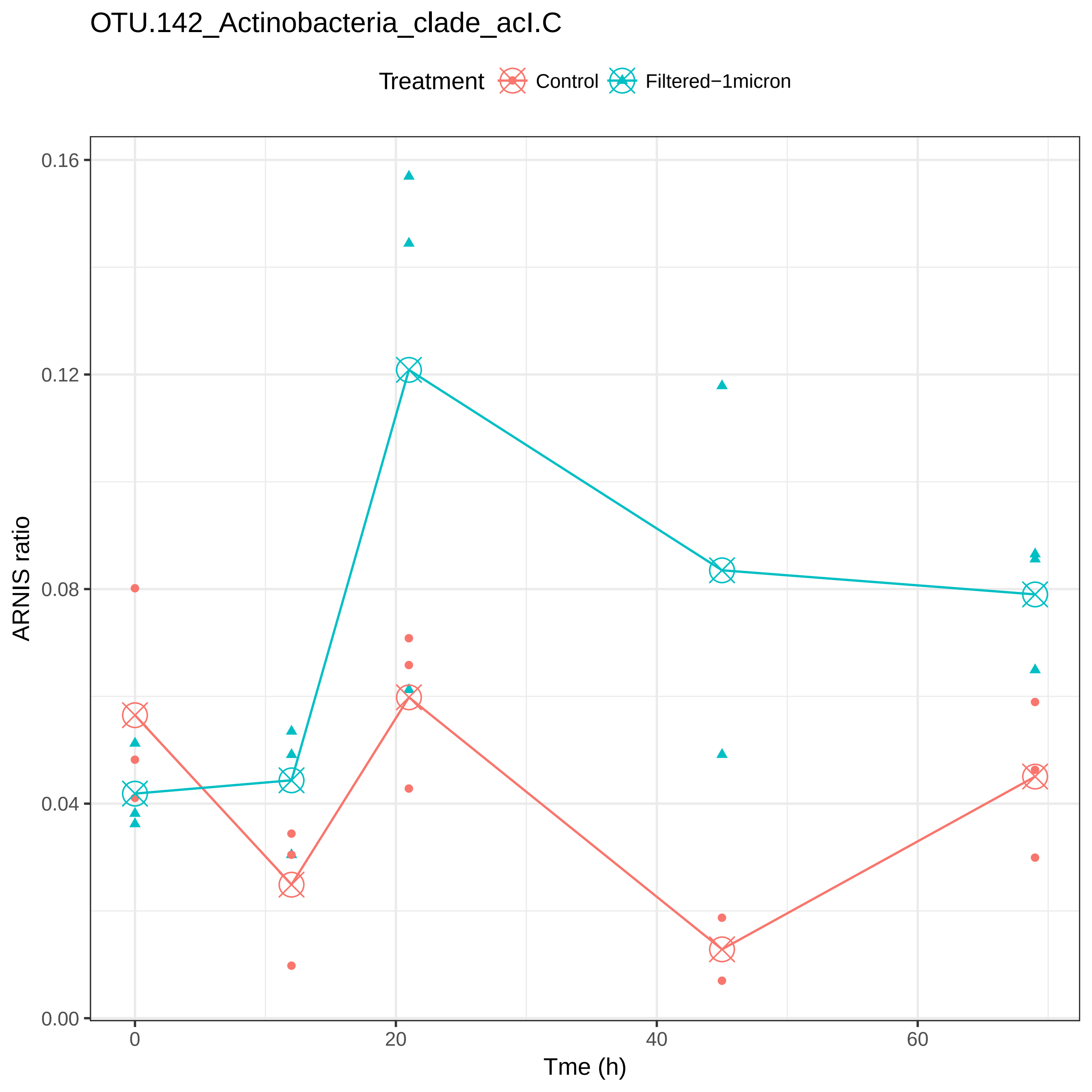

OTU.142_Actinobacteria_clade_acI.C
Treatment
Control
Filtered−1micron
ARNIS ratio
0.16
0.12
0.08
0.04
0.00
0
20
40
60
Tme (h)

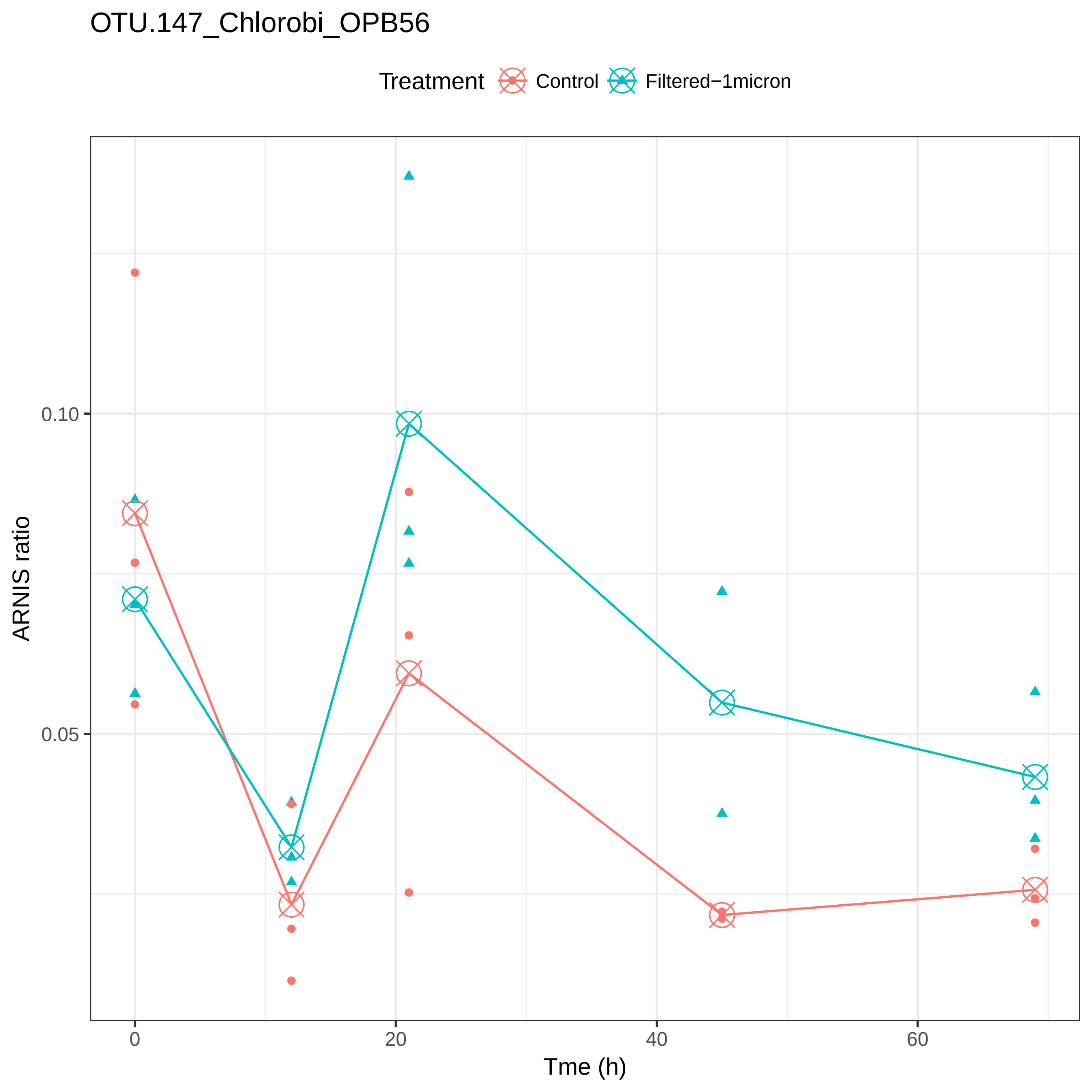

OTU.147_Chlorobi_OPB56
Treatment
Control
Filtered−1micron
0.10
0.05
ARNIS ratio
0
20
40
60
Tme (h)

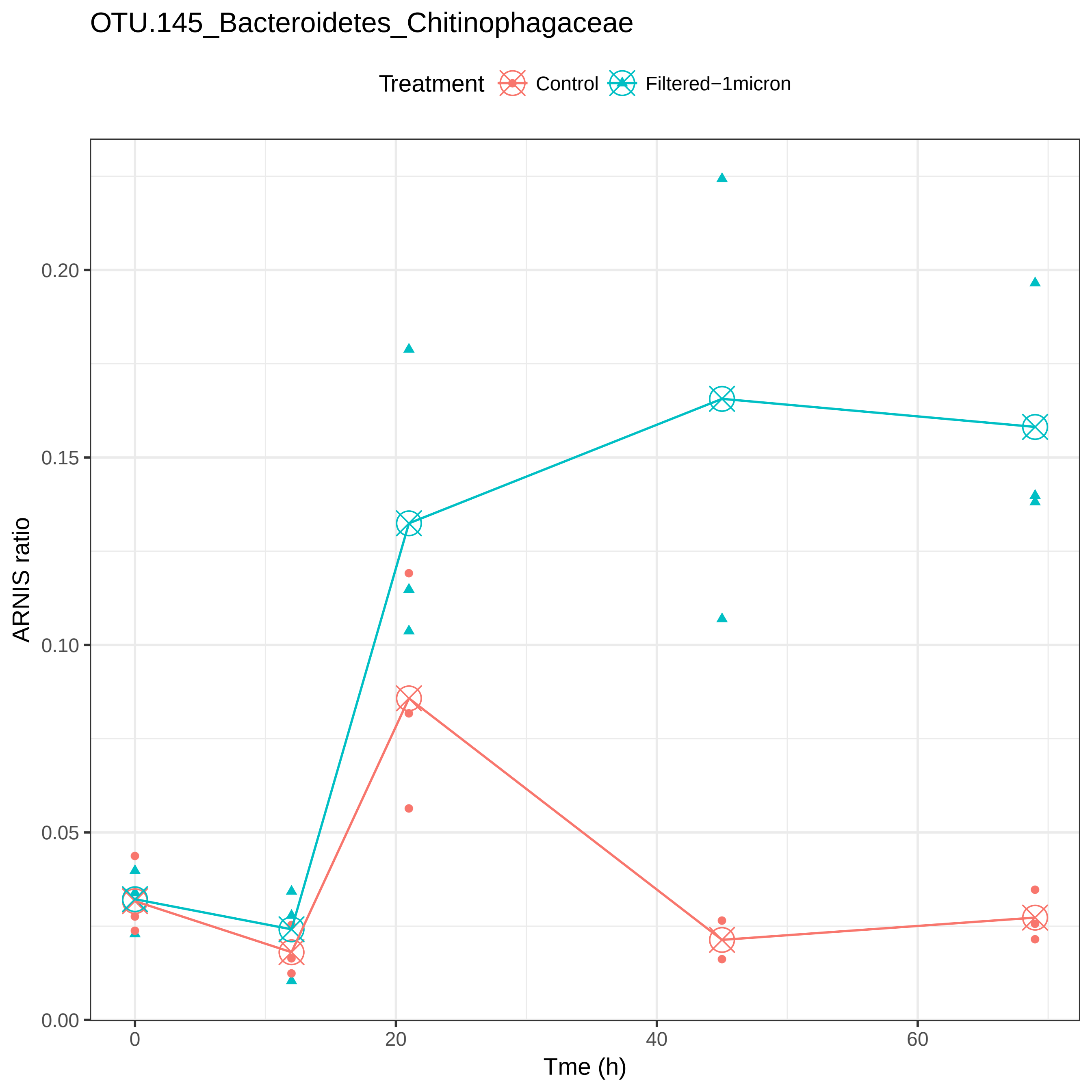

OTU.145_Bacteroidetes_Chitinophagaceae
Treatment
Control
Filtered−1micron
ARNIS ratio
0.00
0.05
0.10
0.15
0.20
0
20
40
60
Tme (h)

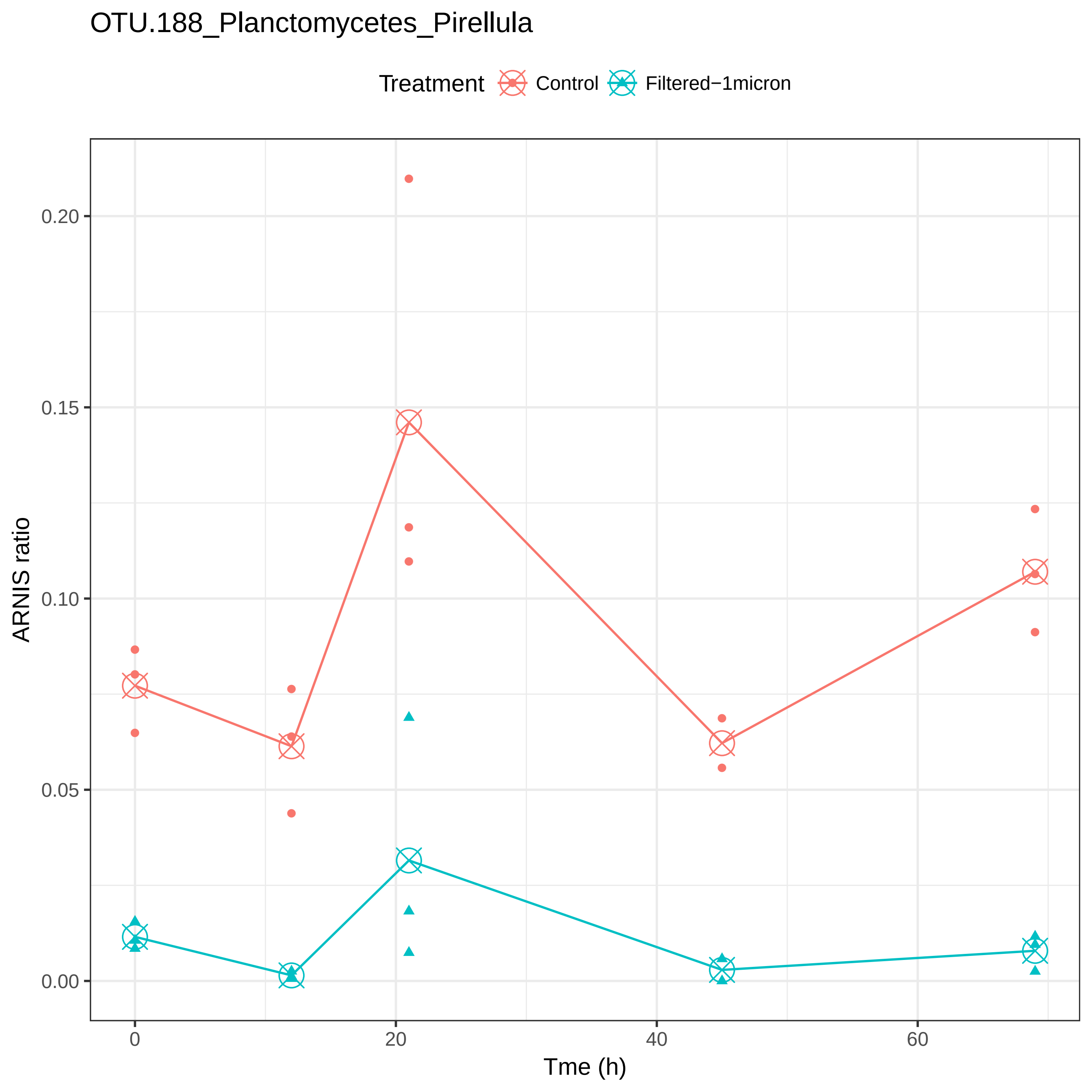

OTU.188_Planctomycetes_Pirellula
Treatment
Control
Filtered−1micron
ARNIS ratio
0.20
0.15
0.10
0.05
0.00
0
20
40
60
Tme (h)

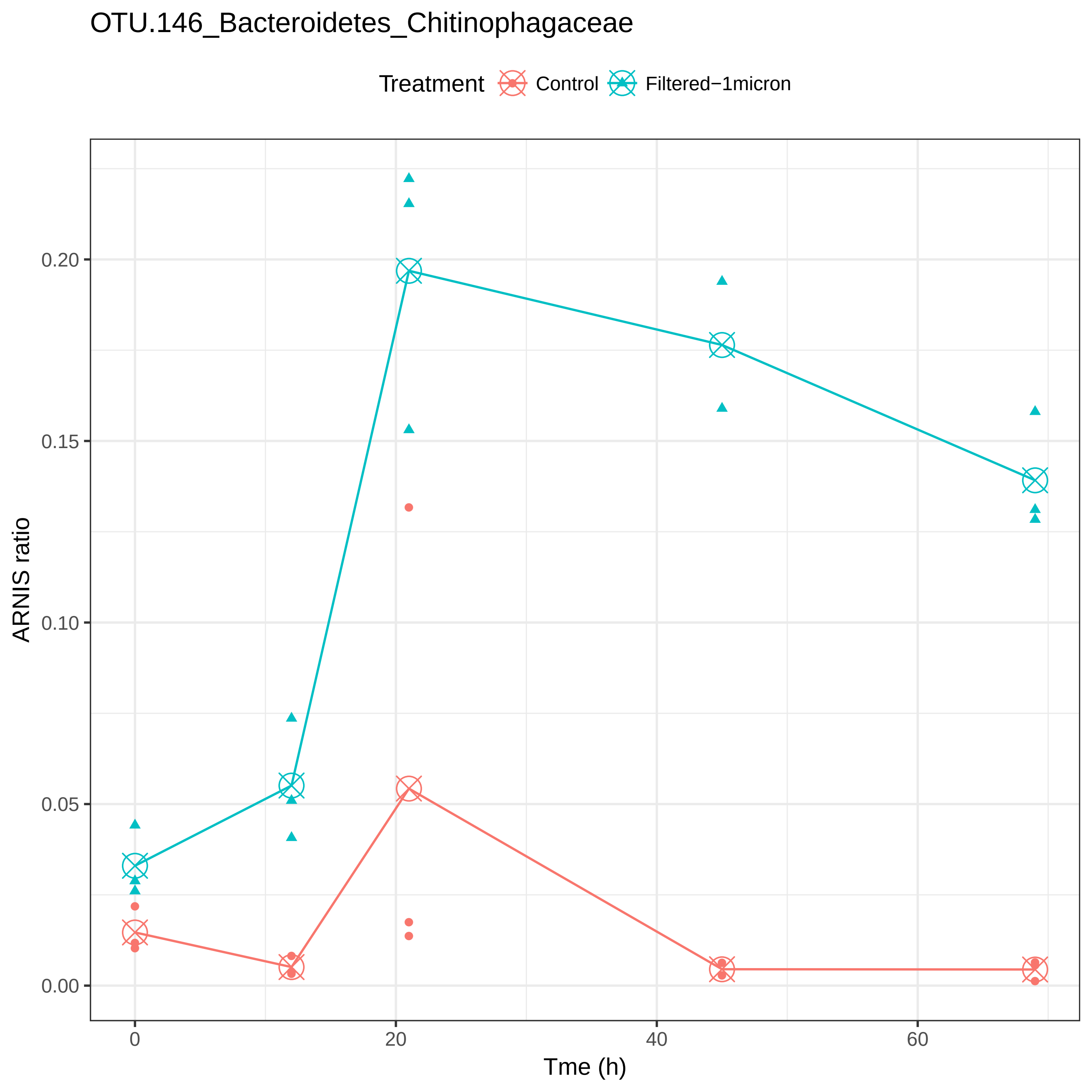

OTU.146_Bacteroidetes_Chitinophagaceae
Treatment
Control
Filtered−1micron
ARNIS ratio
0.00
0.05
0.10
0.15
0.20
0
20
40
60
Tme (h)

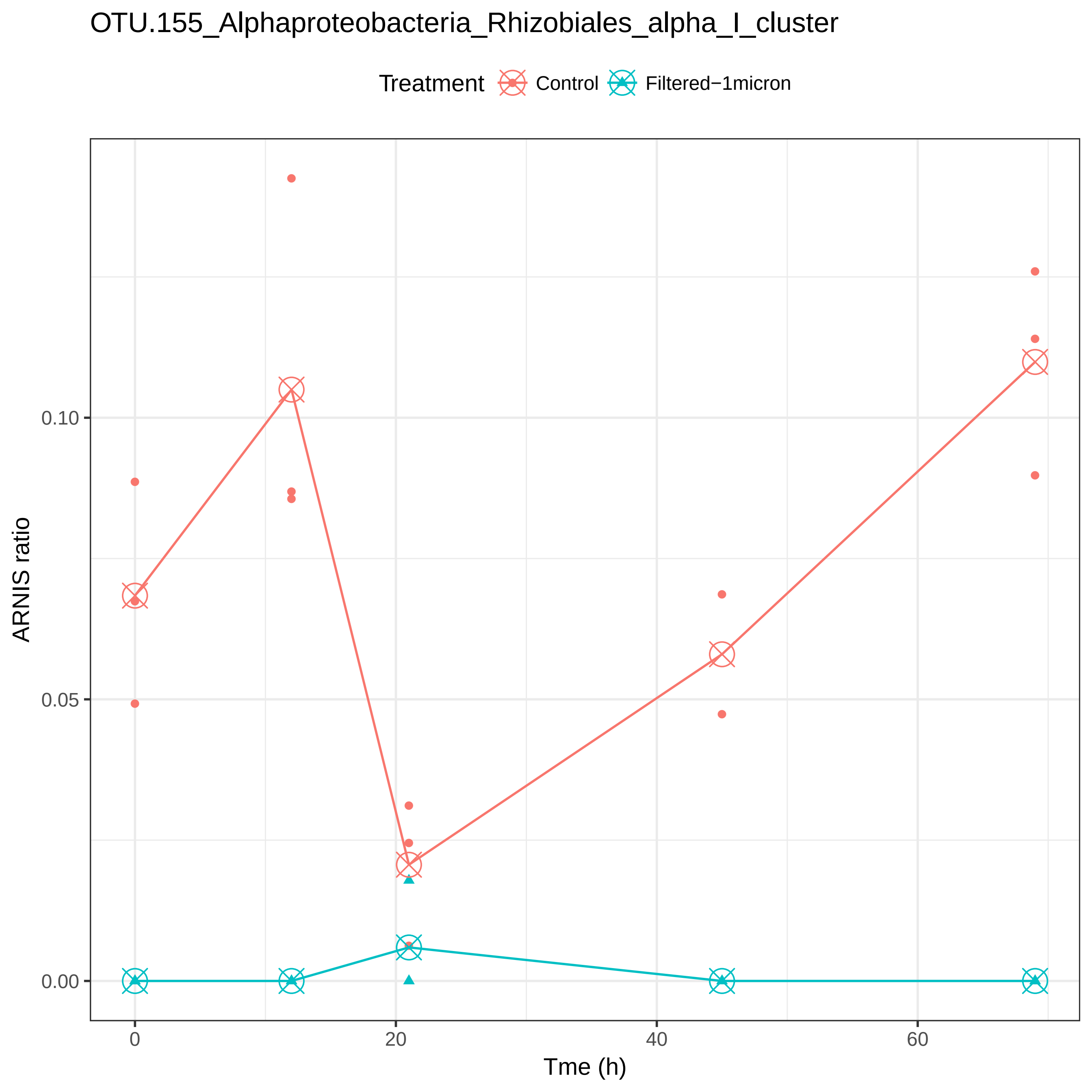

OTU.155_Alphaproteobacteria_Rhizobiales_alpha_I_cluster
Treatment
Control
Filtered−1micron
ARNIS ratio
0.10
0.05
0.00
0
20
40
60
Tme (h)

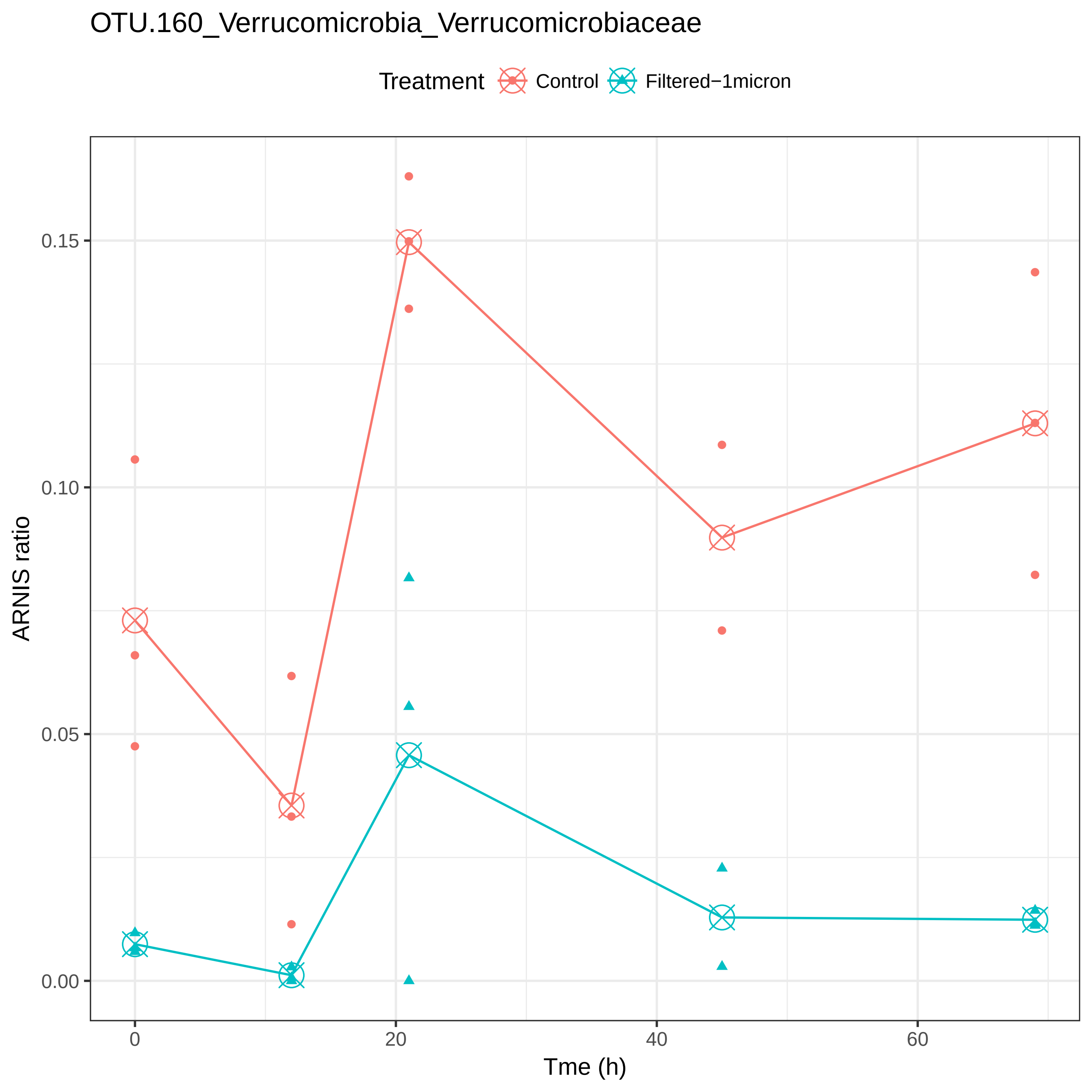

OTU.160_Verrucomicrobia_Verrucomicrobiaceae
Treatment
Control
Filtered−1micron
ARNIS ratio
0.15
0.10
0.05
0.00
0
20
40
60
Tme (h)

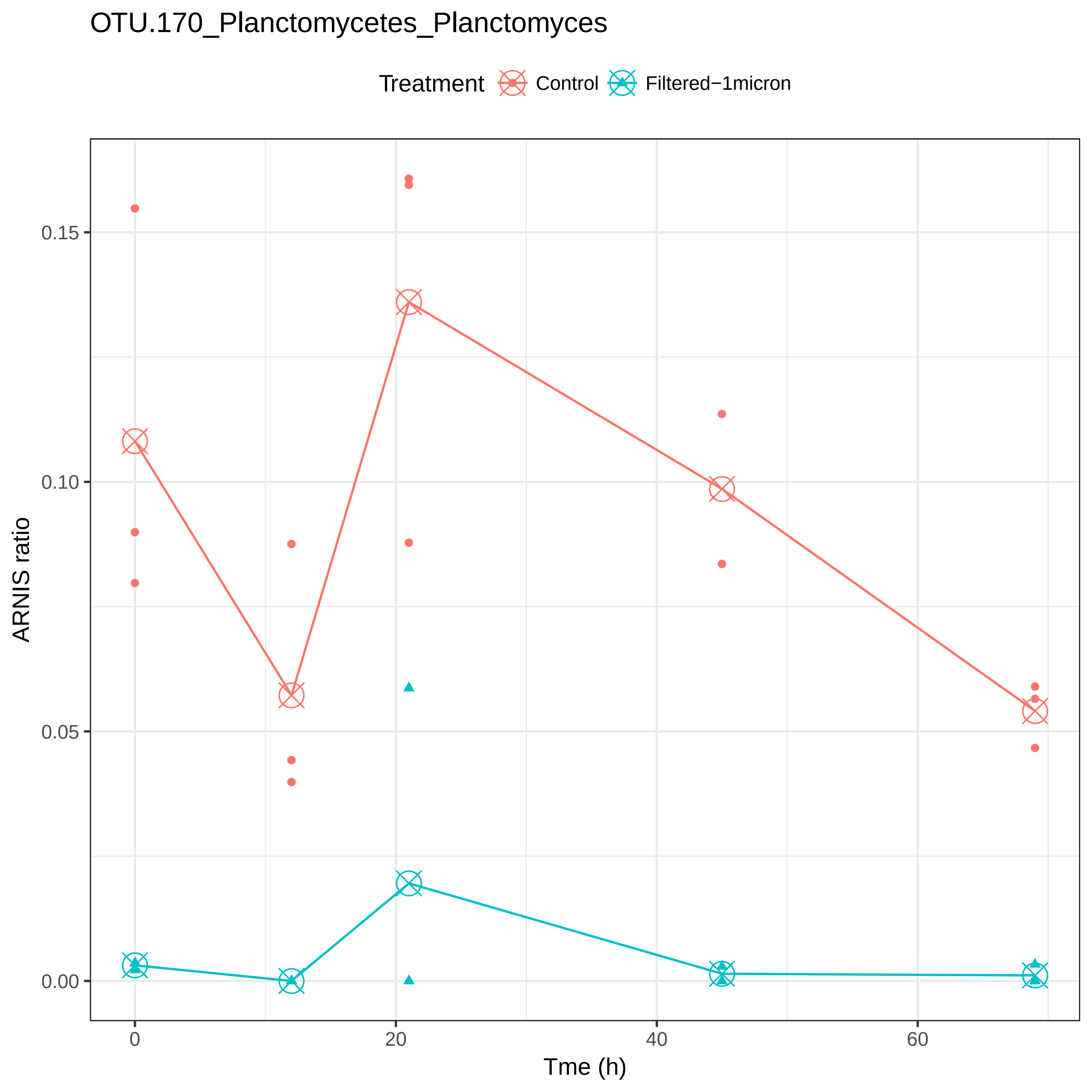

OTU.170_Planctomycetes_Planctomyces
Treatment
Control
Filtered−1micron
ARNIS ratio
0.15
0.10
0.05
0.00
0
20
40
60
Tme (h)

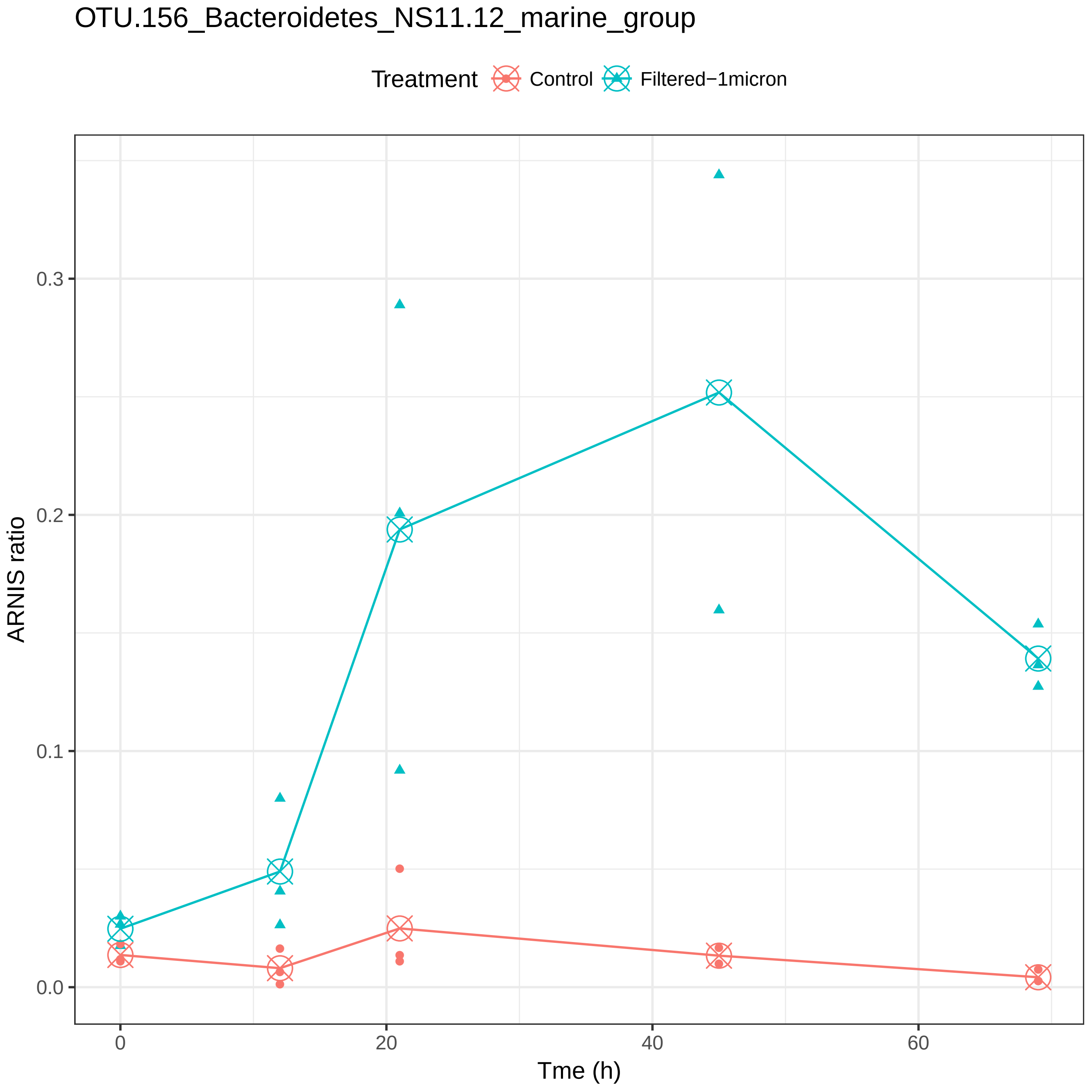

OTU.156_Bacteroidetes_NS11.12_marine_group
Treatment
Control
Filtered−1micron
ARNIS ratio
0.0
0.1
0.2
0.3
0
20
40
60
Tme (h)

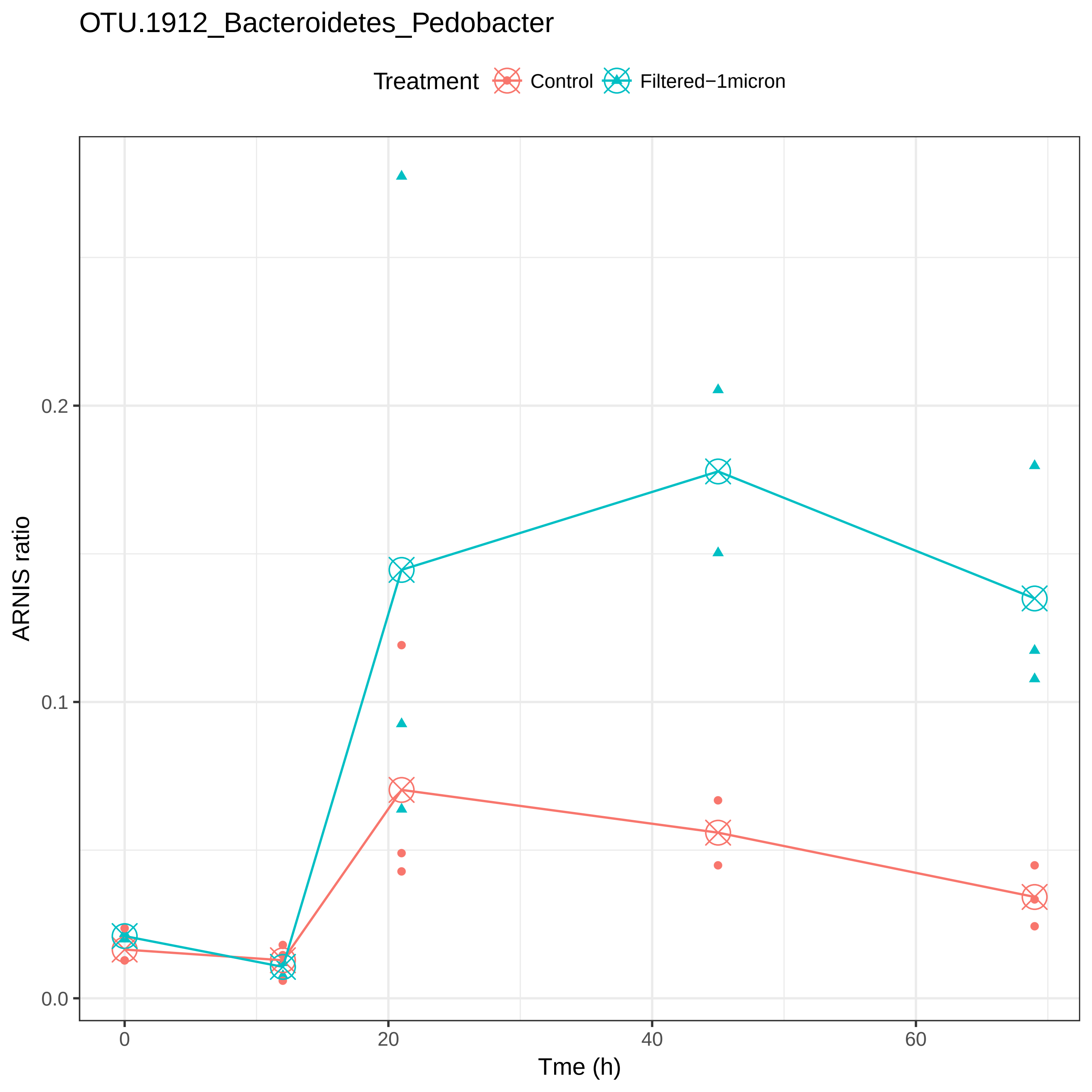

OTU.1912_Bacteroidetes_Pedobacter
Treatment
Control
Filtered−1micron
ARNIS ratio
0.0
0.1
0.2
0
20
40
60
Tme (h)

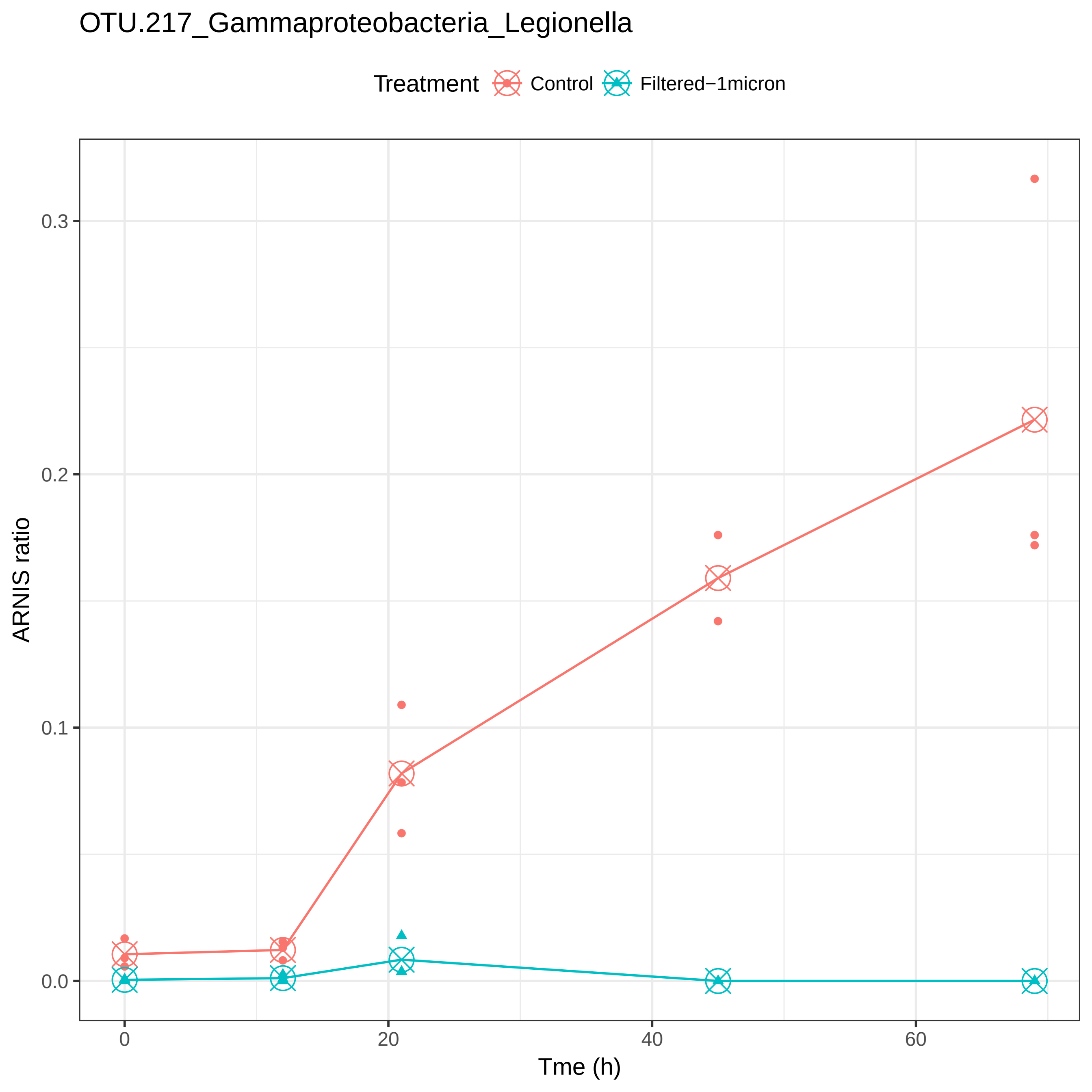

OTU.217_Gammaproteobacteria_Legionella
Treatment
Control
Filtered−1micron
ARNIS ratio
0.3
0.2
0.1
0.0
0
20
40
60
Tme (h)

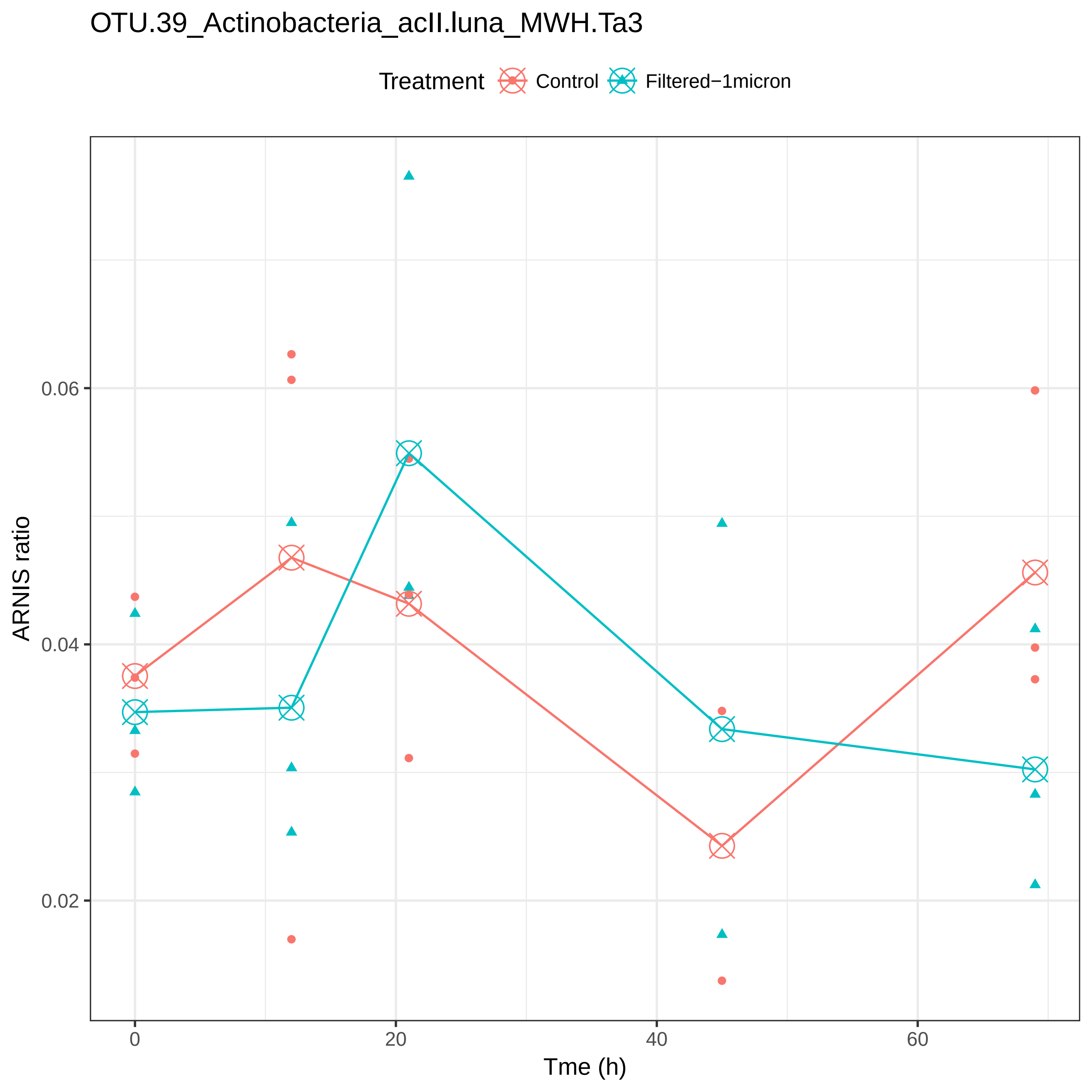

OTU.39_Actinobacteria_acII.luna_MWH.Ta3
Treatment
Control
Filtered−1micron
ARNIS ratio
0.06
0.04
0.02
0
20
40
60
Tme (h)

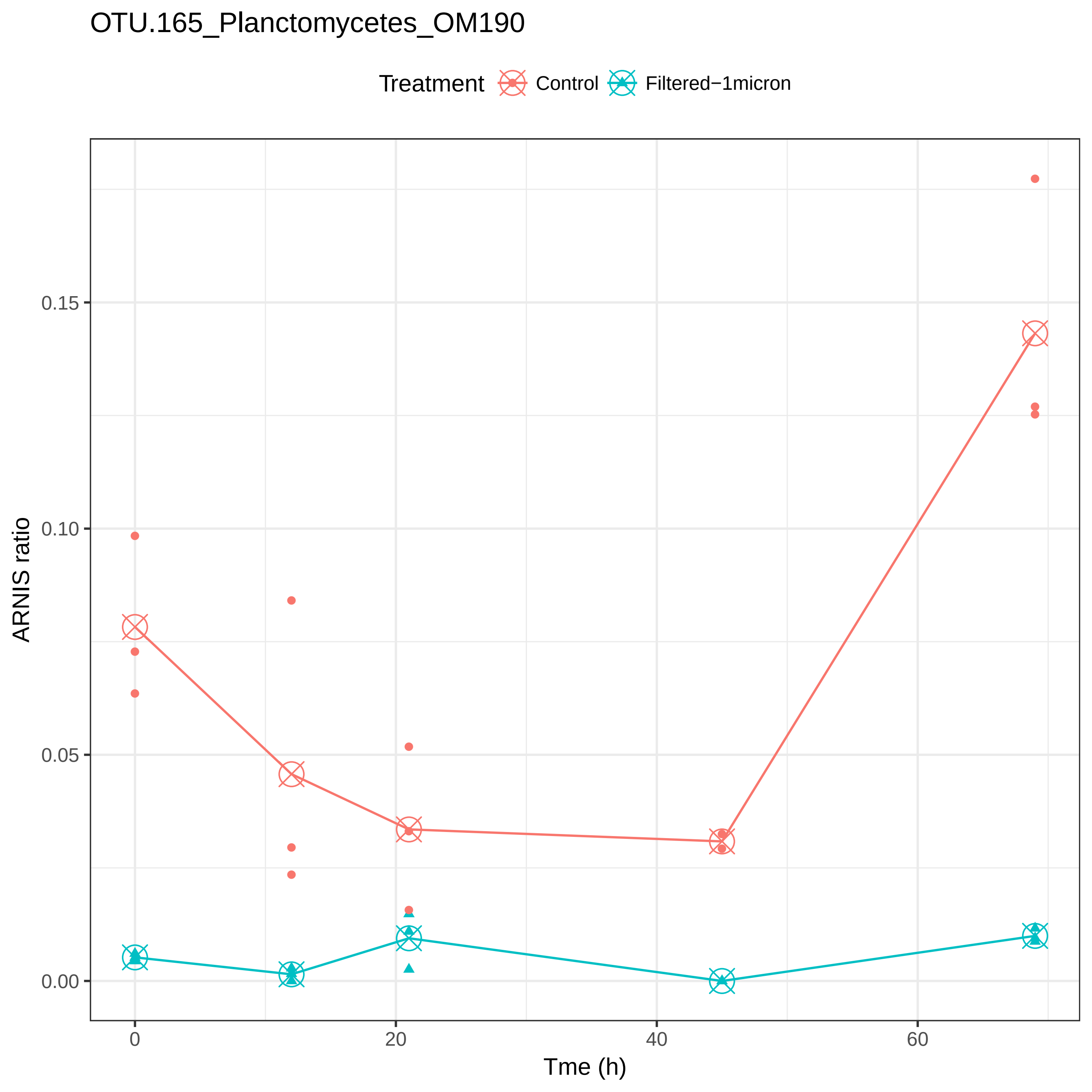
OTU.165_Planctomycetes_OM190
Treatment
Control
Filtered−1micron
ARNIS ratio
0.15
0.10
0.05
0.00
0
20
40
60
Tme (h)

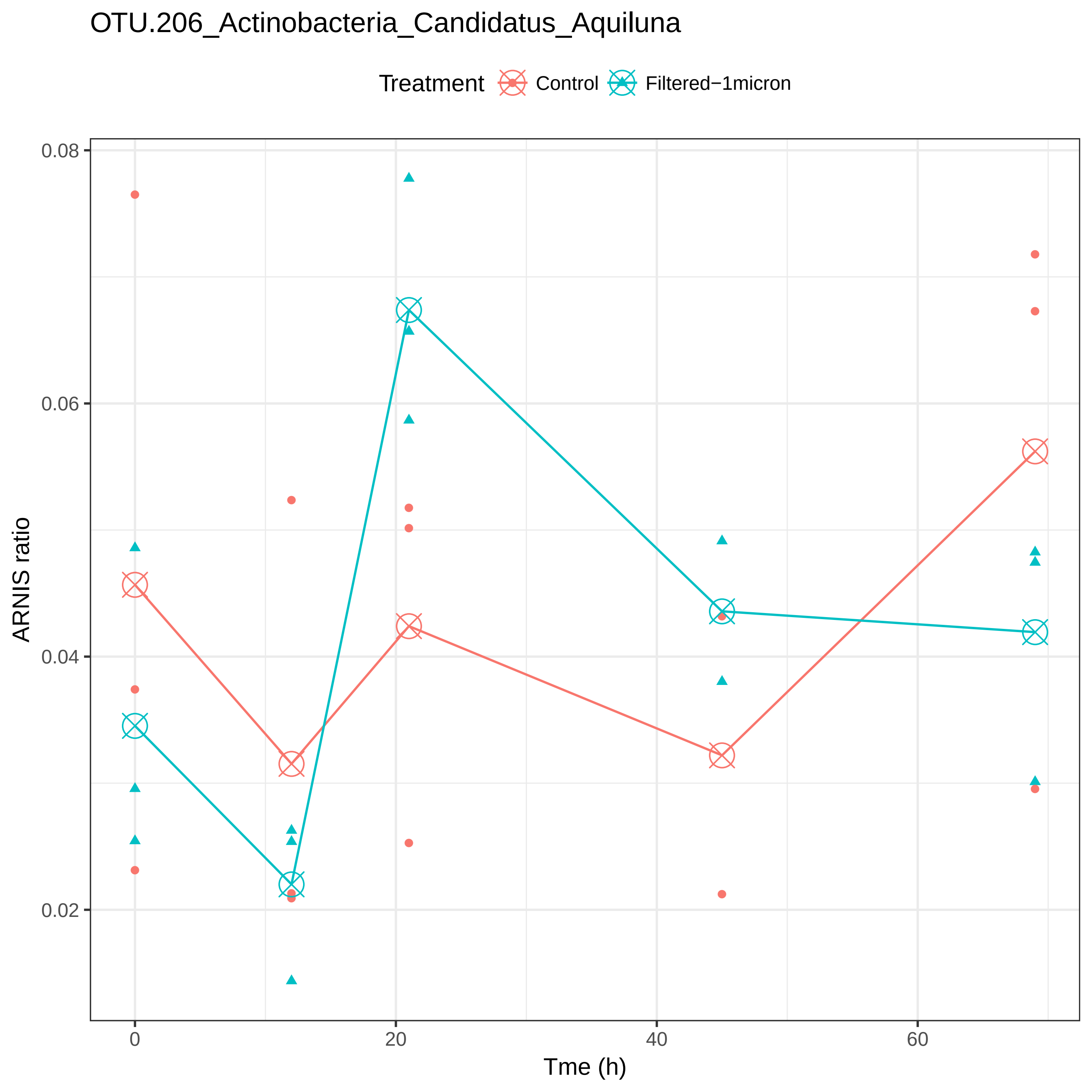
OTU.206_Actinobacteria_Candidatus_Aquiluna
Treatment
Control
Filtered−1micron
ARNIS ratio
0.08
0.06
0.04
0.02
0
20
40
60
Tme (h)

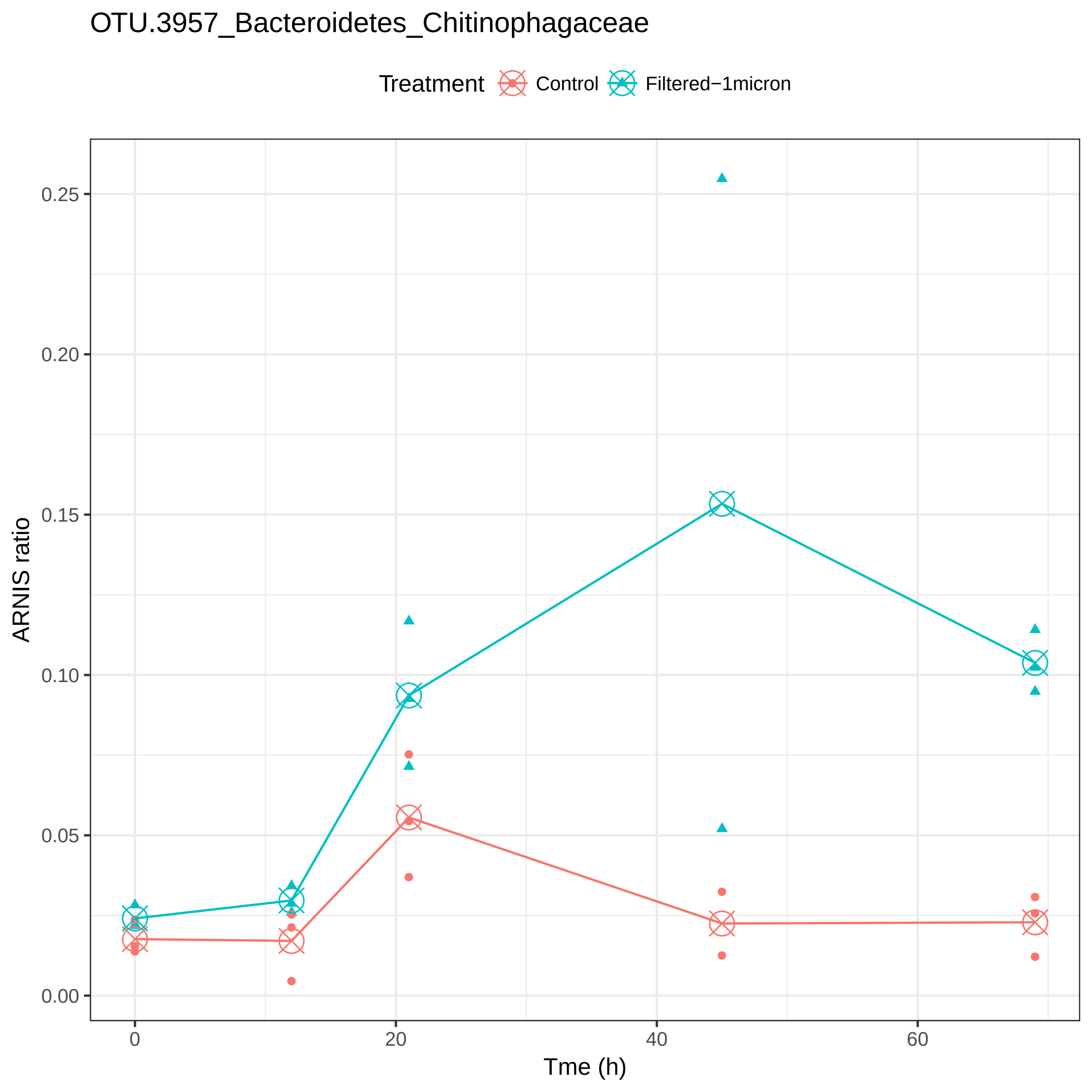

OTU.3957_Bacteroidetes_Chitinophagaceae
Treatment
Control
Filtered−1micron
ARNIS ratio
0.25
0.20
0.15
0.10
0.05
0.00
0
20
40
60
Tme (h)

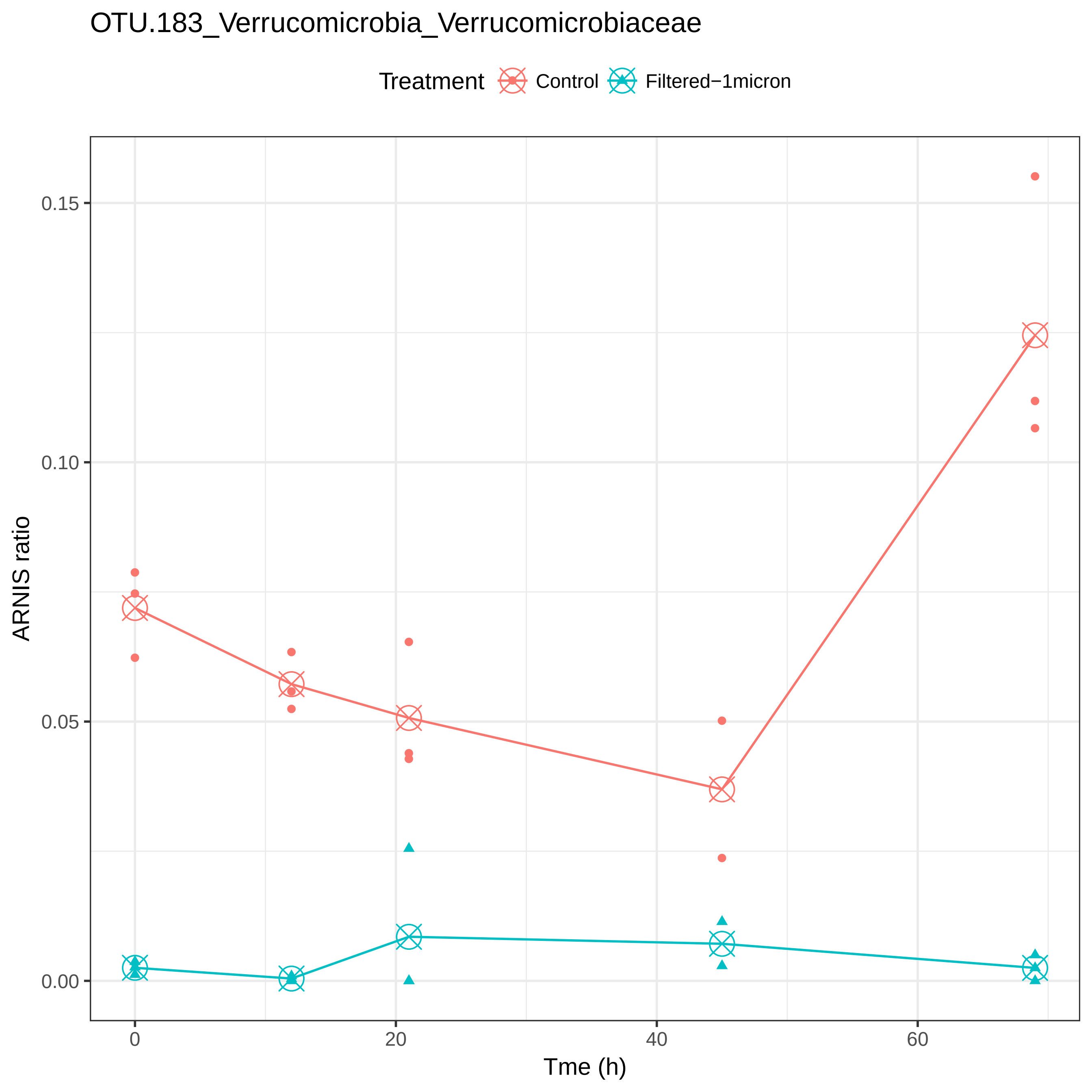

OTU.183_Verrucomicrobia_Verrucomicrobiaceae
Treatment
Control
Filtered−1micron
ARNIS ratio
0.00
0.05
0.10
0.15
0
20
40
60
Tme (h)

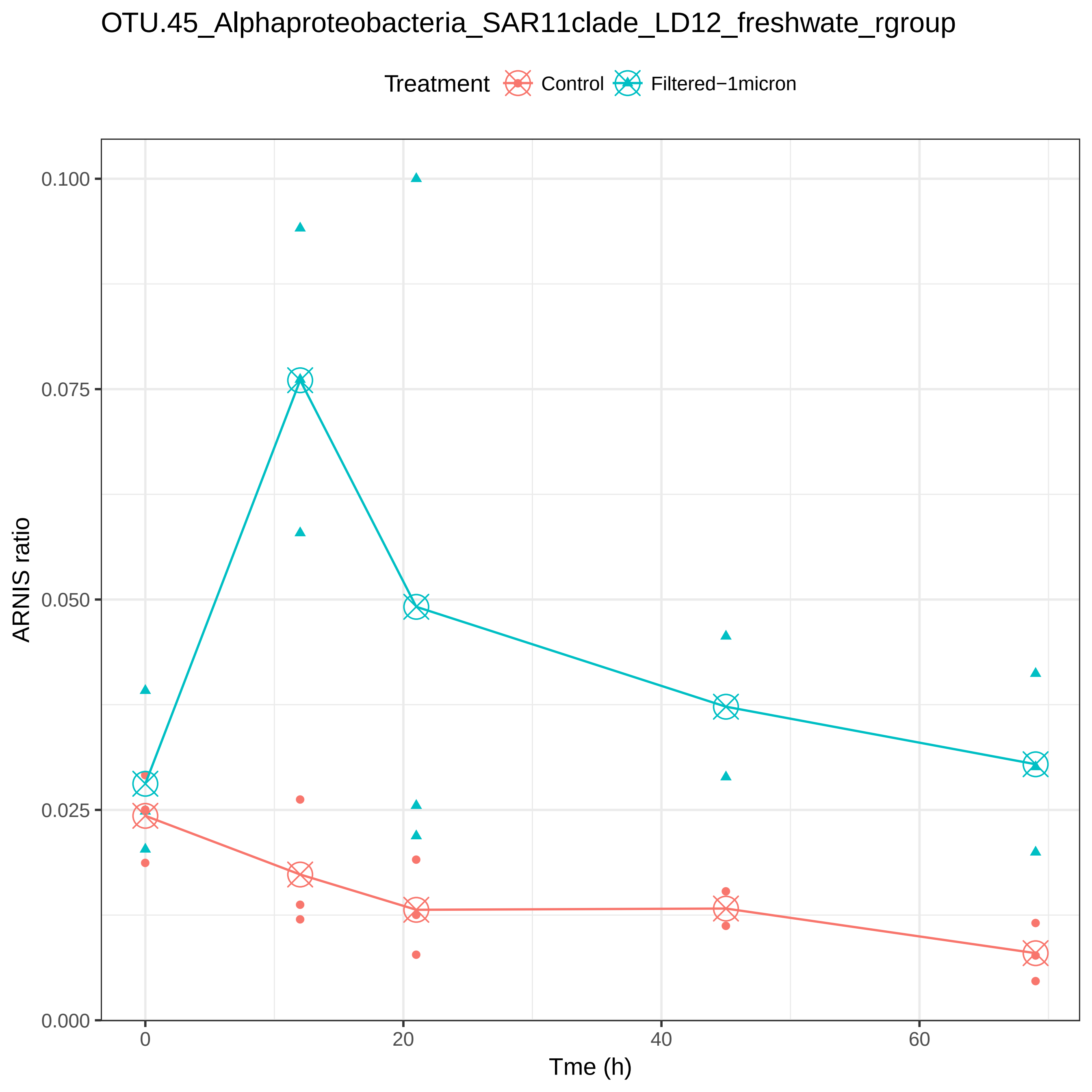
OTU.45_Alphaproteobacteria_SAR11clade_LD12_freshwate_rgroup
Treatment
Control
Filtered−1micron
ARNIS ratio
0.100
0.075
0.050
0.025
0.000
0
20
40
60
Tme (h)

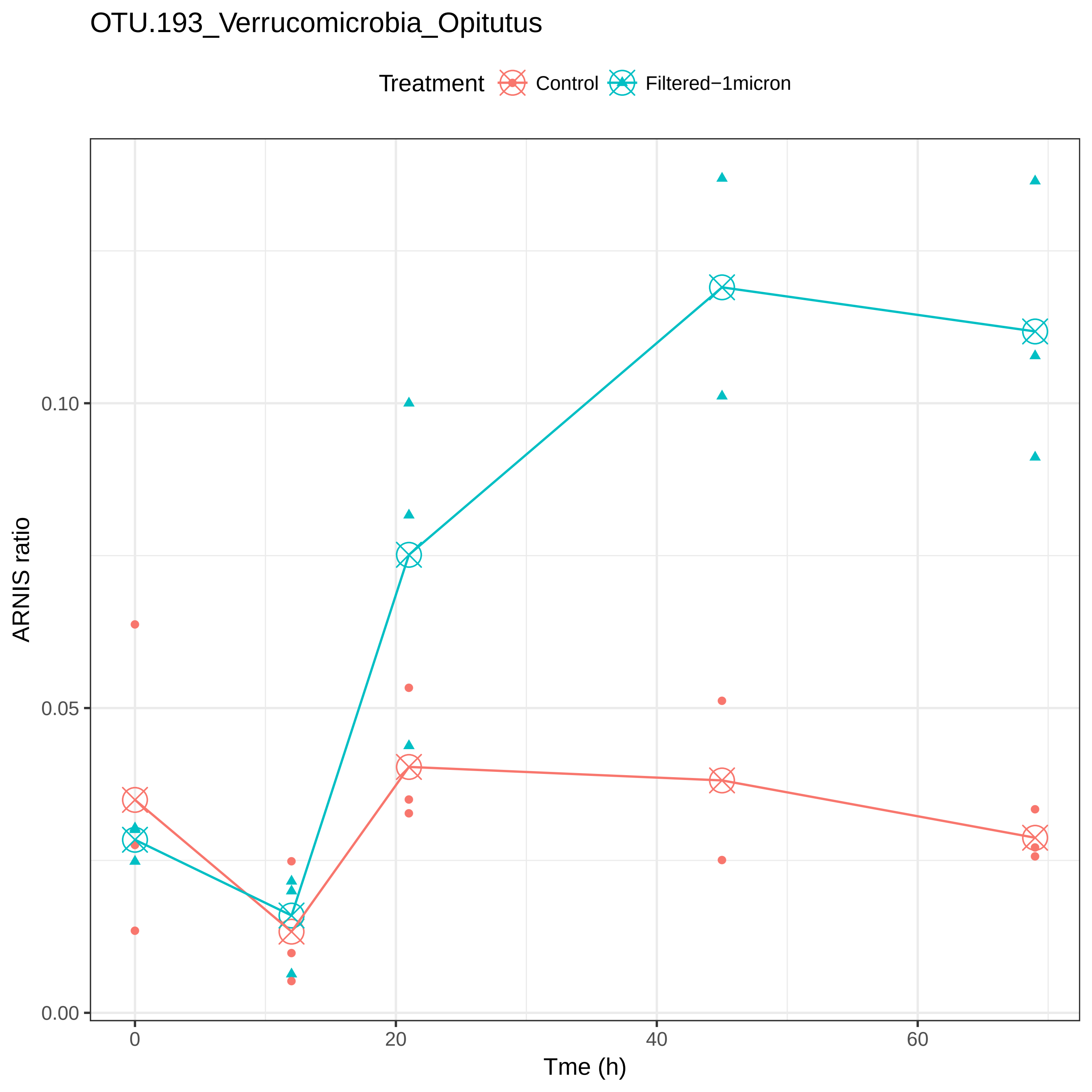

OTU.193_Verrucomicrobia_Opitutus
Treatment
Control
Filtered−1micron
ARNIS ratio
0.00
0.05
0.10
0
20
40
60
Tme (h)

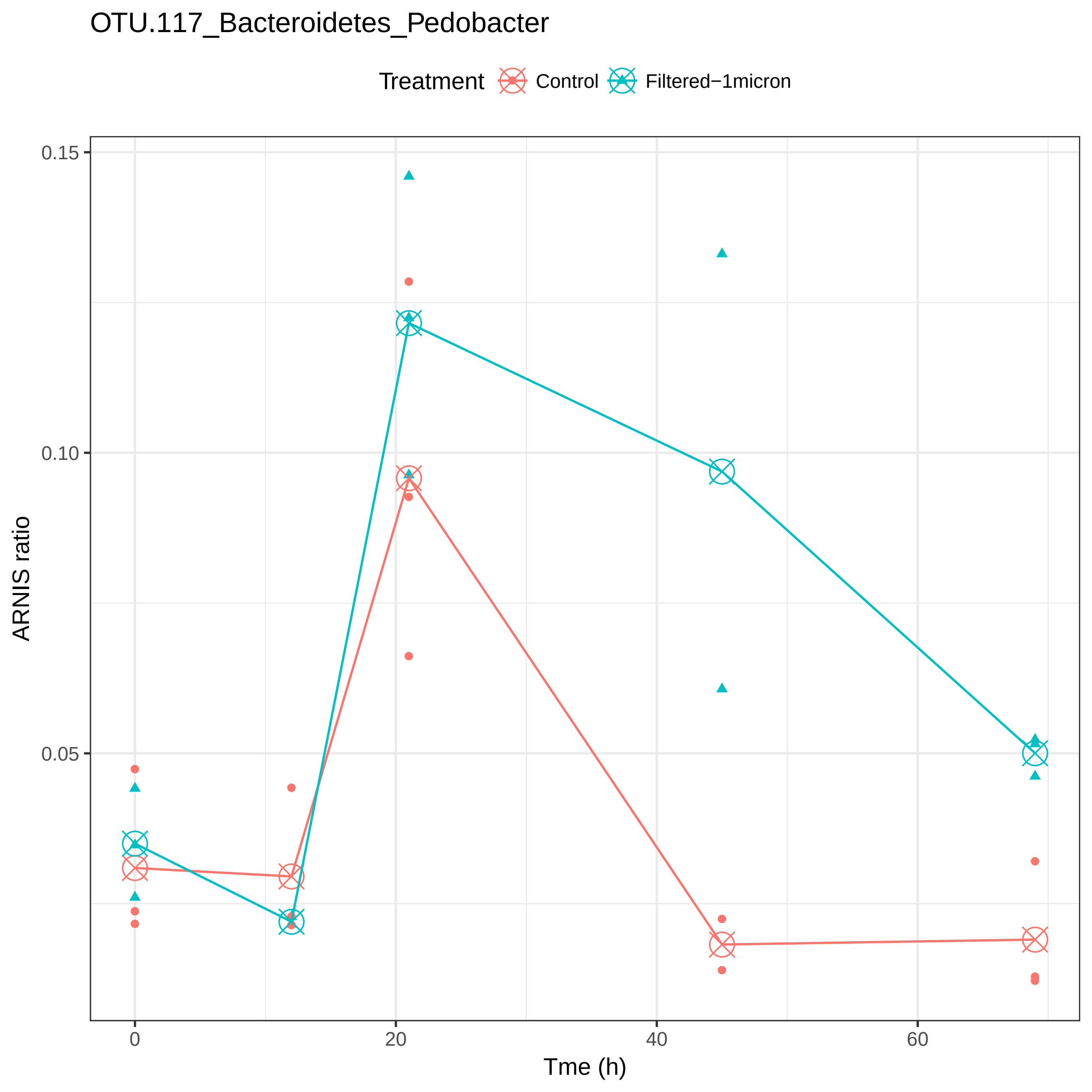
OTU.117_Bacteroidetes_Pedobacter
Treatment
Control
Filtered−1micron
0.15
0.10
0.05
ARNIS ratio
0
20
40
60
Tme (h)

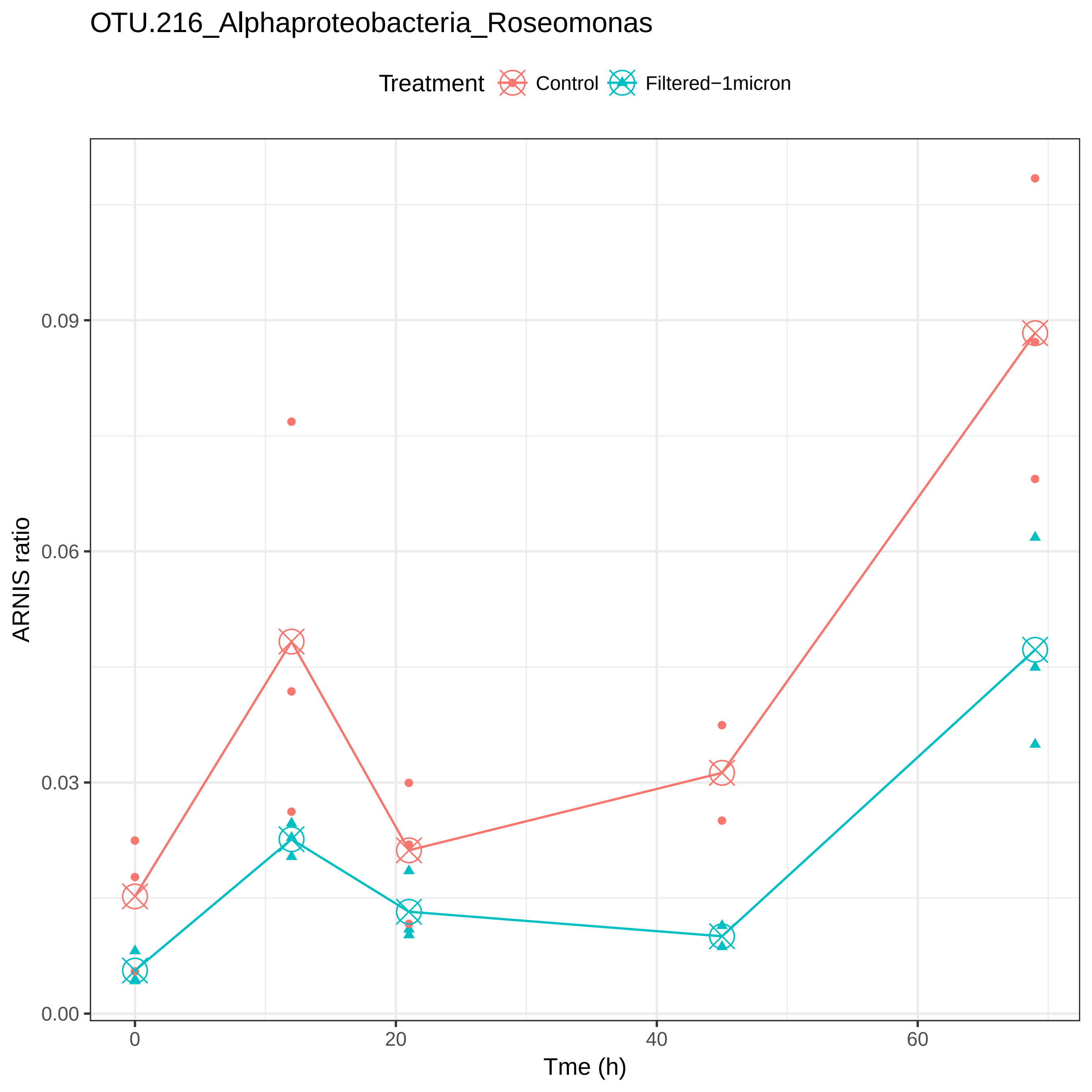
OTU.216_Alphaproteobacteria_Roseomonas
Treatment
Control
Filtered−1micron
ARNIS ratio
0.00
0.03
0.06
0.09
0
20
40
60
Tme (h)

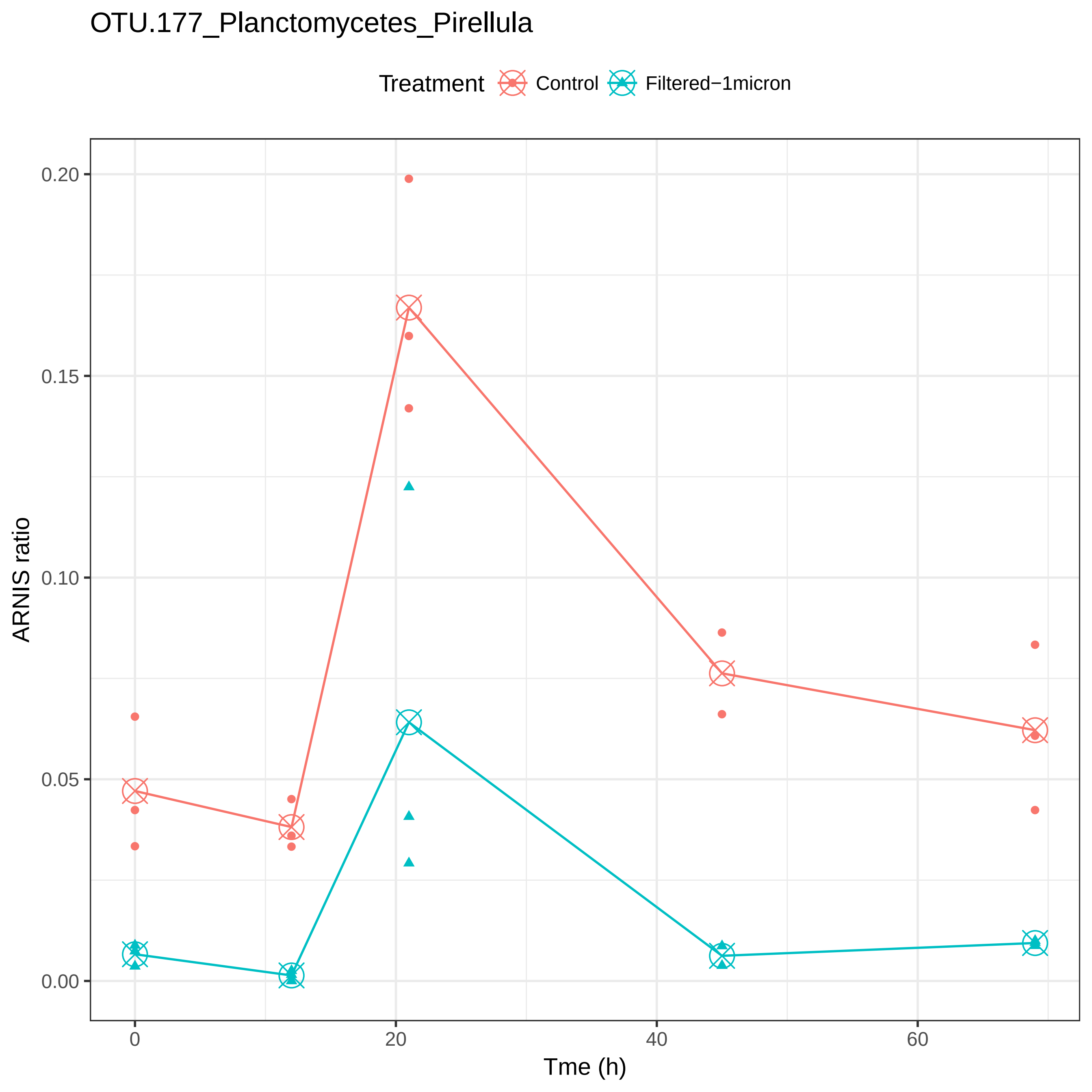
OTU.177_Planctomycetes_Pirellula
Treatment
Control
Filtered−1micron
ARNIS ratio
0.20
0.15
0.10
0.05
0.00
0
20
40
60
Tme (h)

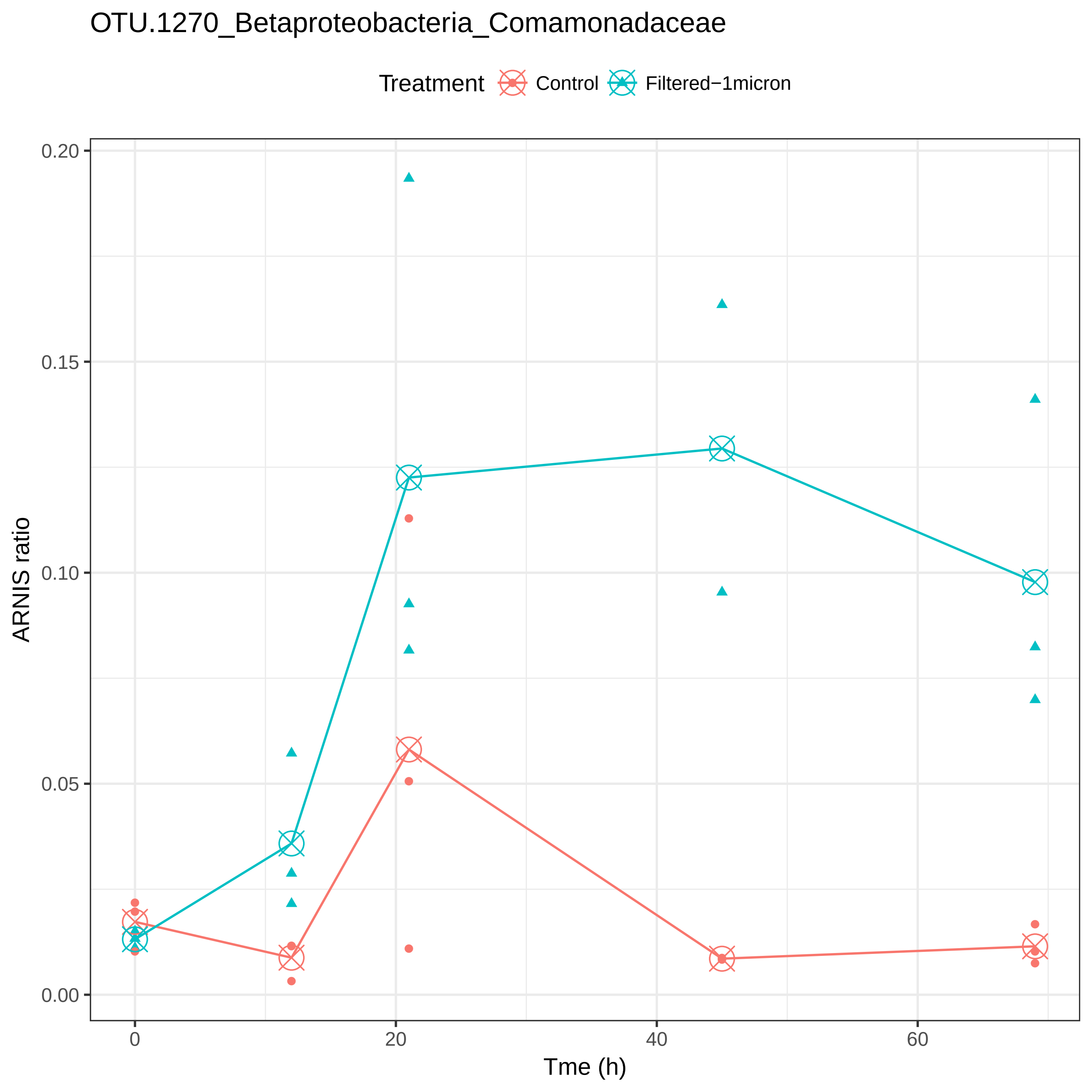

OTU.1270_Betaproteobacteria_Comamonadaceae
Treatment
Control
Filtered−1micron
0.20
0.15
0.10
0.05
0.00
ARNIS ratio
0
20
40
60
Tme (h)

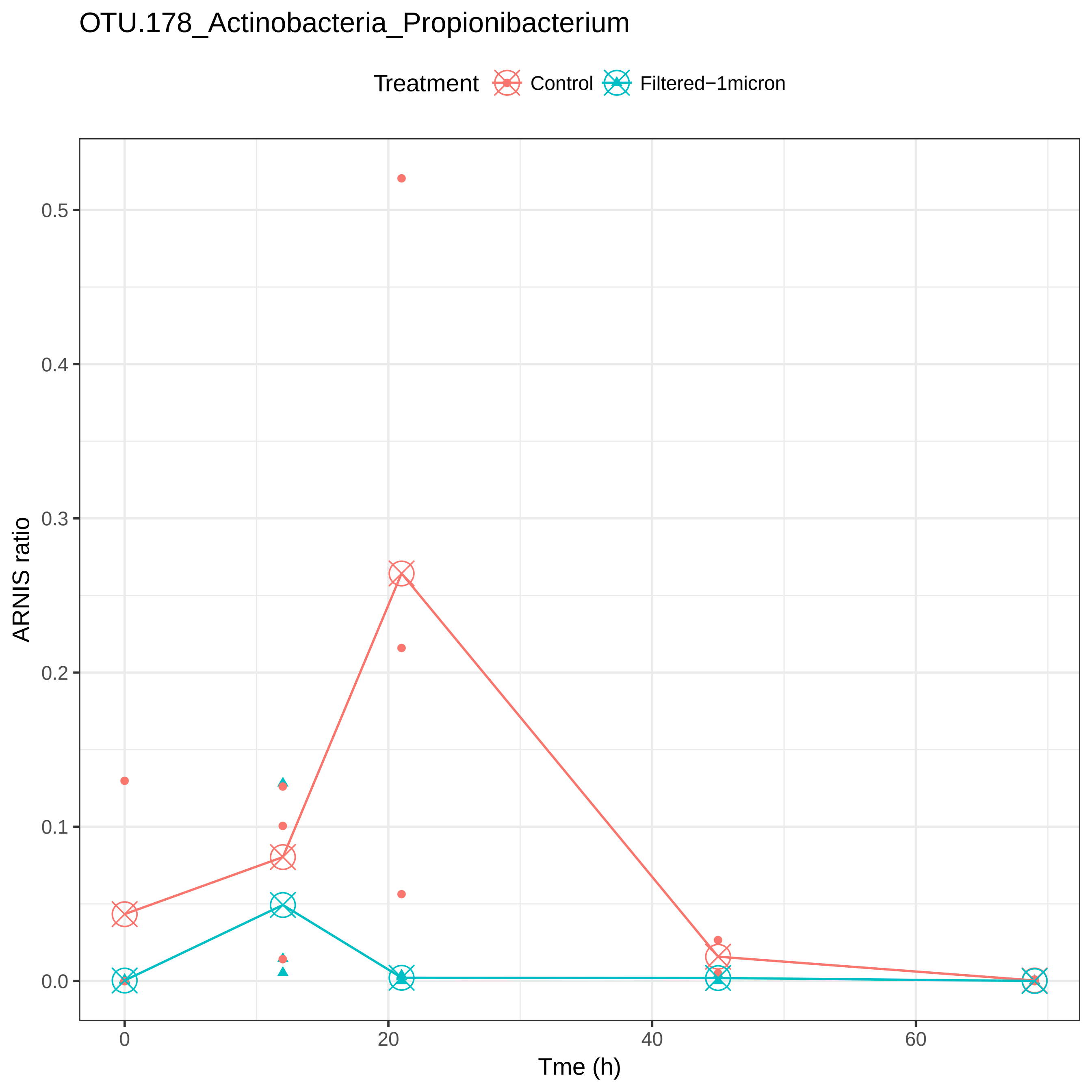

OTU.178_Actinobacteria_Propionibacterium
Treatment
Control
Filtered−1micron
ARNIS ratio
0.0
0.1
0.2
0.3
0.4
0.5
0
20
40
60
Tme (h)

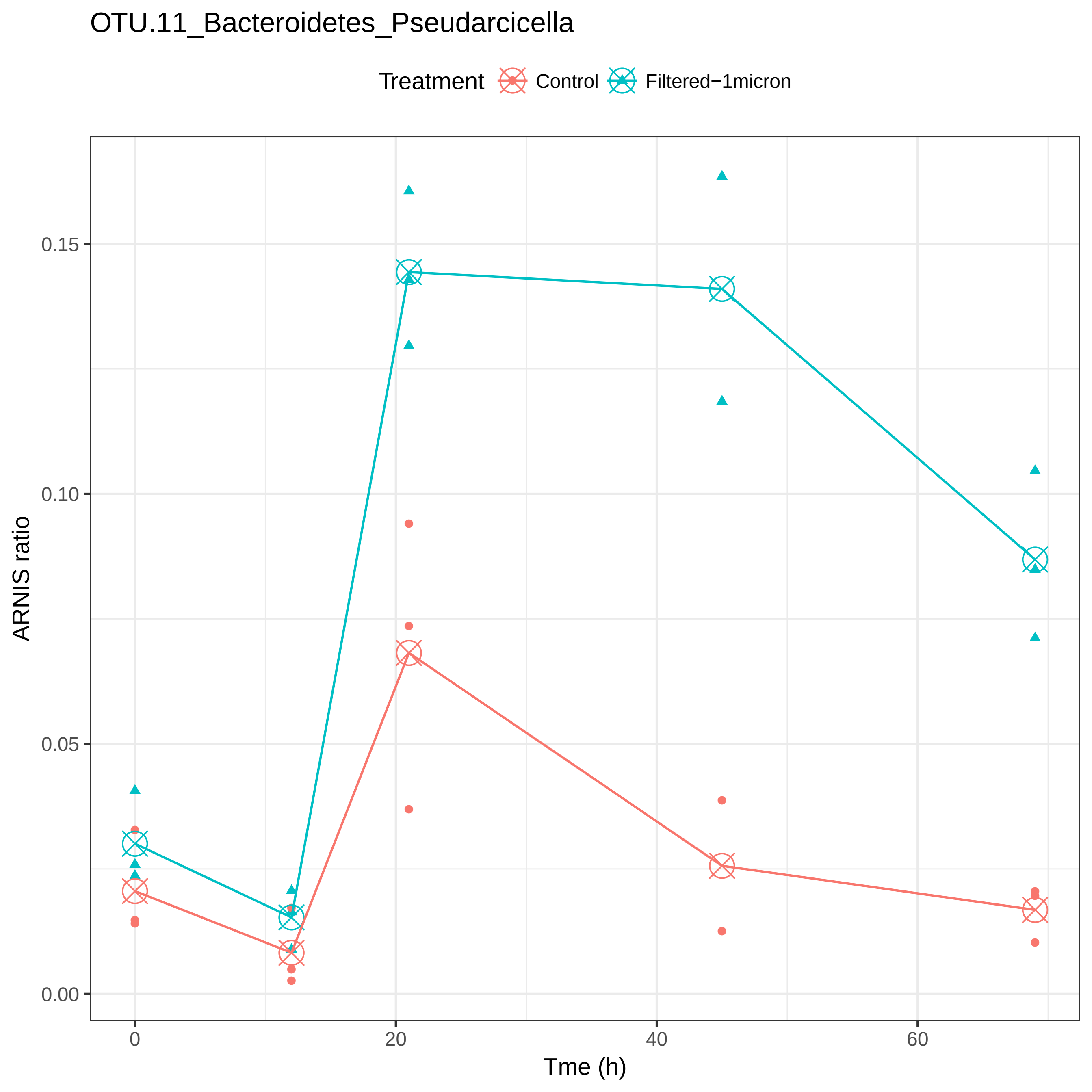
OTU.11_Bacteroidetes_Pseudarcicella
Treatment
Control
Filtered−1micron
ARNIS ratio
0.00
0.05
0.10
0.15
0
20
40
60
Tme (h)

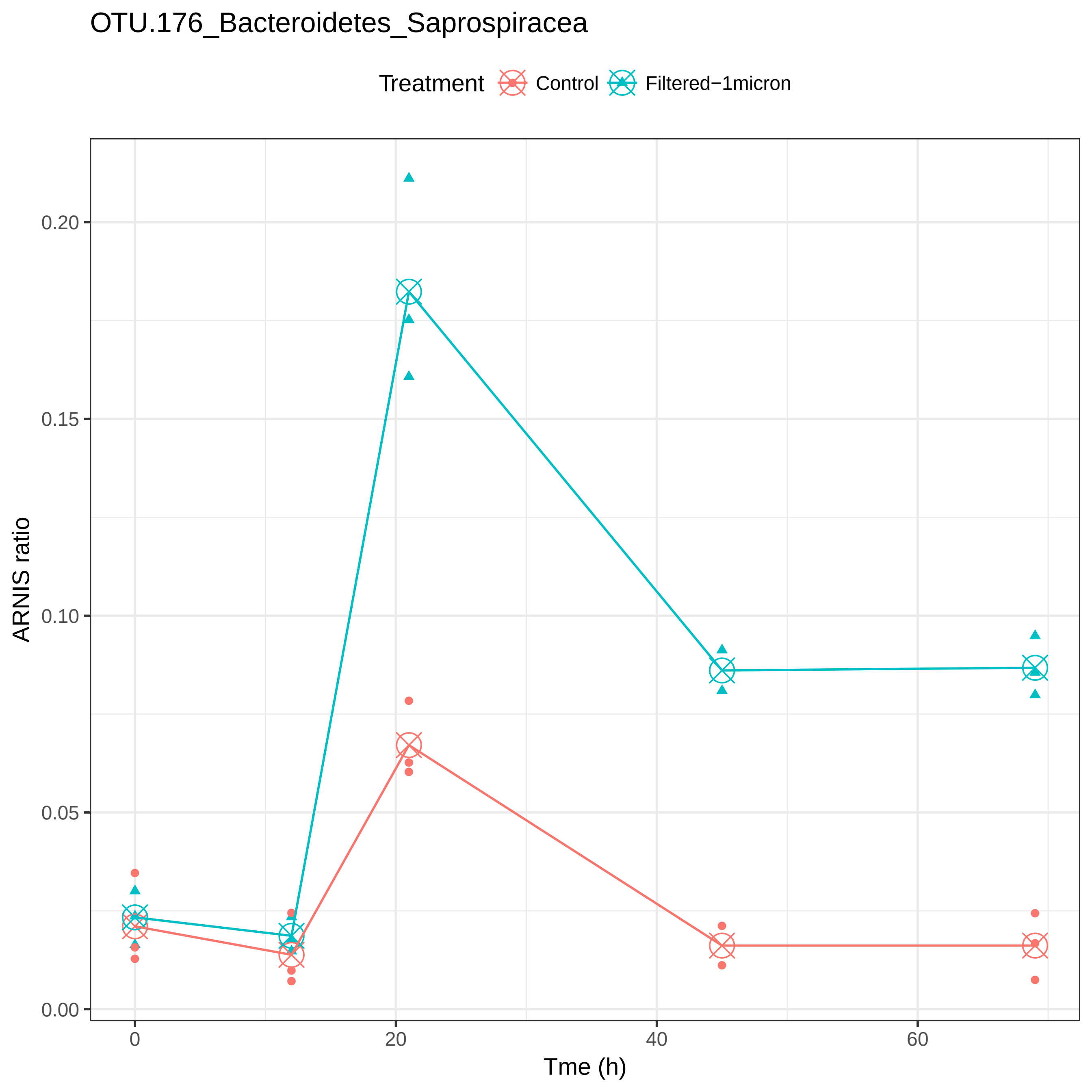
OTU.176_Bacteroidetes_Saprospiracea
Treatment
Control
Filtered−1micron
ARNIS ratio
0.00
0.05
0.10
0.15
0.20
0
20
40
60
Tme (h)

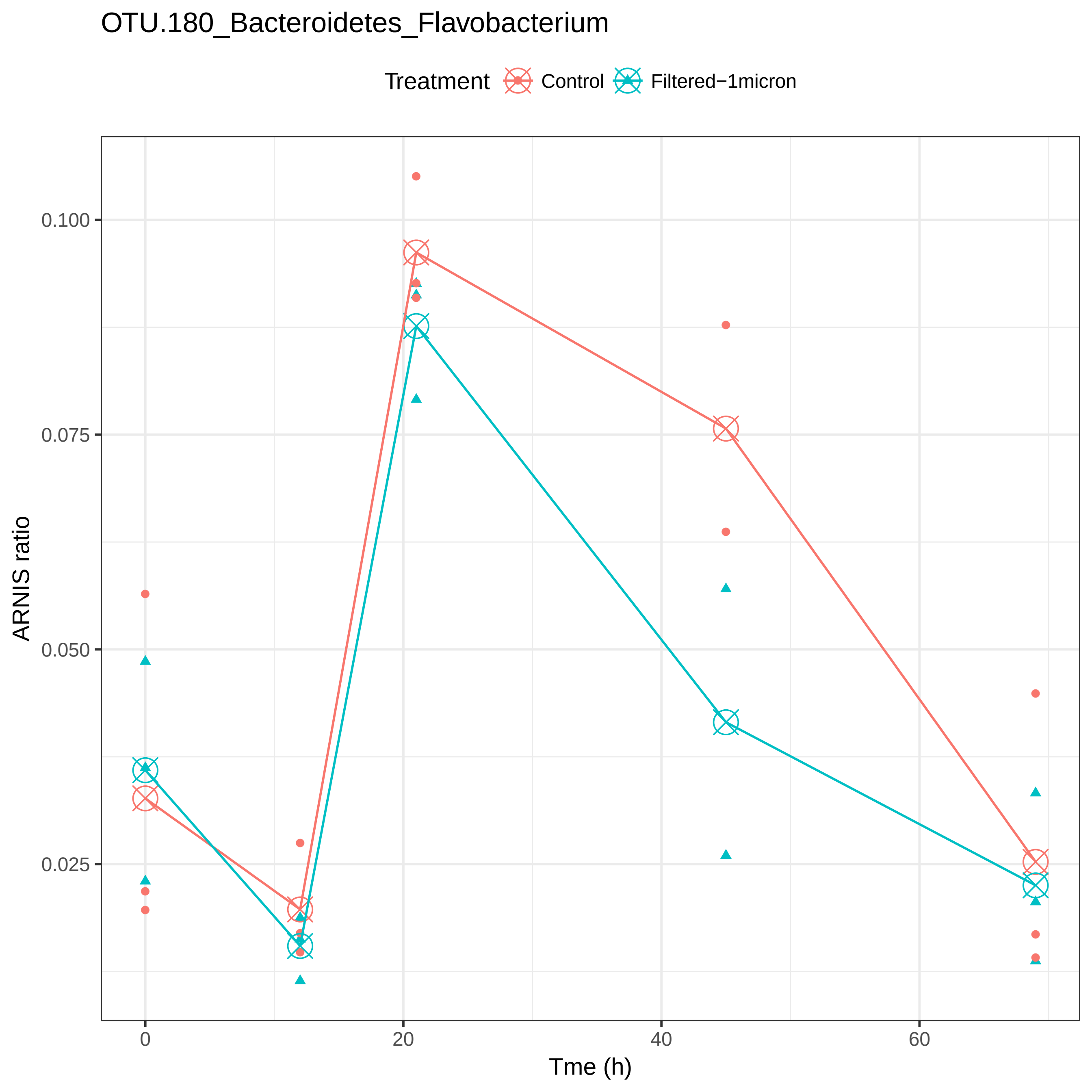
OTU.180_Bacteroidetes_Flavobacterium
Treatment
Control
Filtered−1micron
ARNIS ratio
0.100
0.075
0.050
0.025
0
20
40
60
Tme (h)

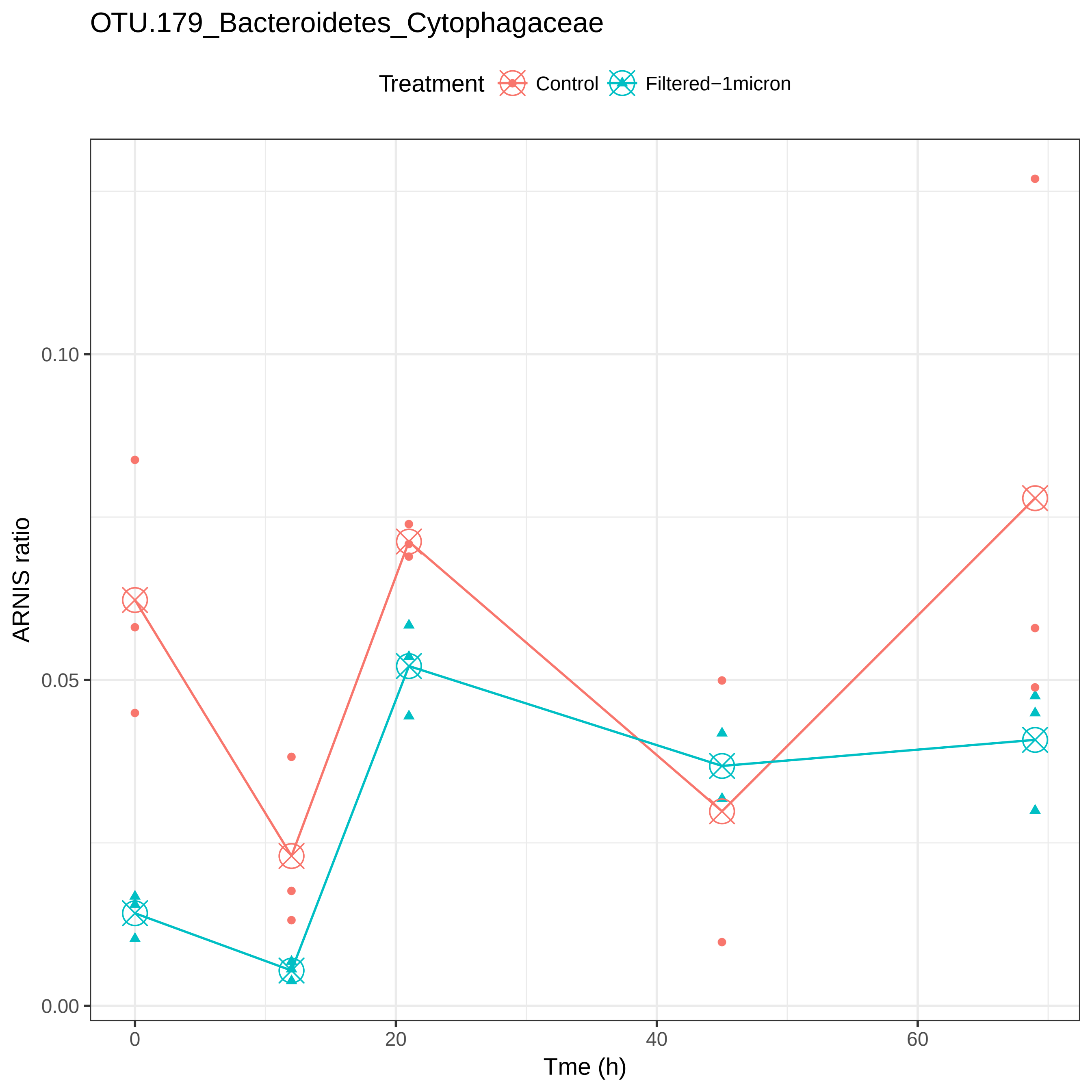

OTU.179_Bacteroidetes_Cytophagaceae
Treatment
Control
Filtered−1micron
ARNIS ratio
0.10
0.05
0.00
0
20
40
60
Tme (h)

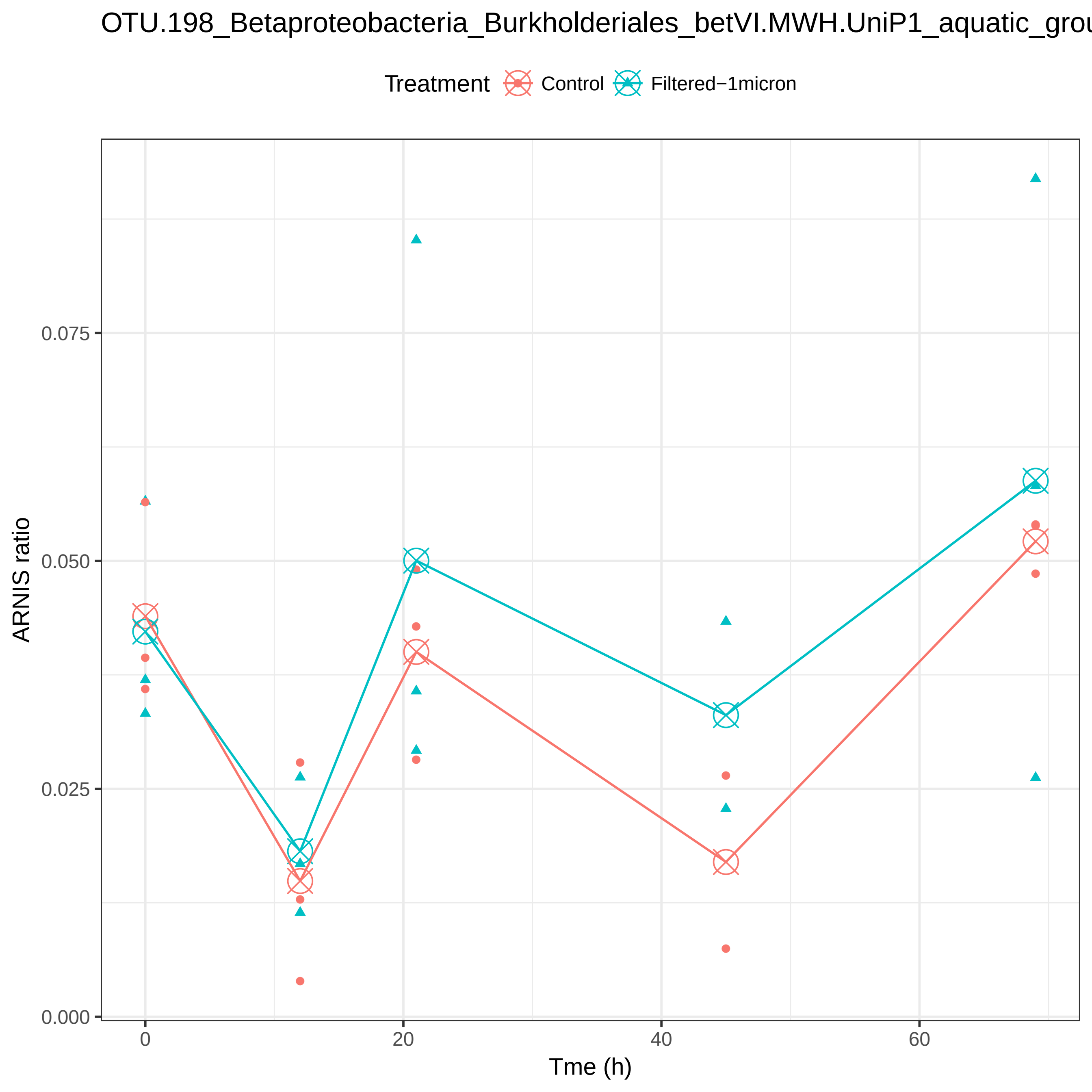

OTU.198_Betaproteobacteria_Burkholderiales_betVI.MWH.UniP1_aquatic_grou
Treatment
Control
Filtered−1micron
ARNIS ratio
0.000
0.025
0.050
0.075
0
20
40
60
Tme (h)

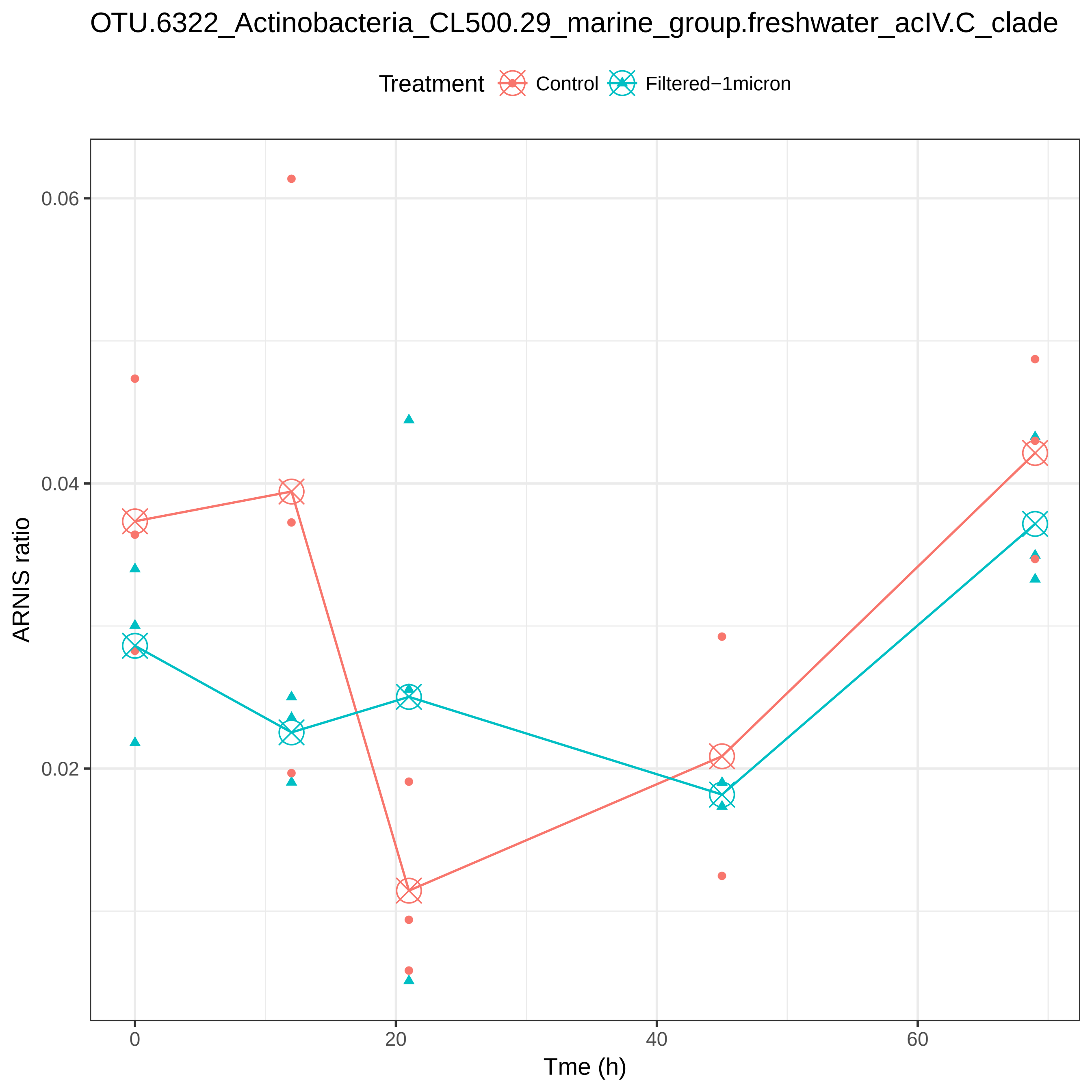

OTU.6322_Actinobacteria_CL500.29_marine_group.freshwater_acIV.C_clade
Treatment
Control
Filtered−1micron
ARNIS ratio
0.06
0.04
0.02
0
20
40
60
Tme (h)

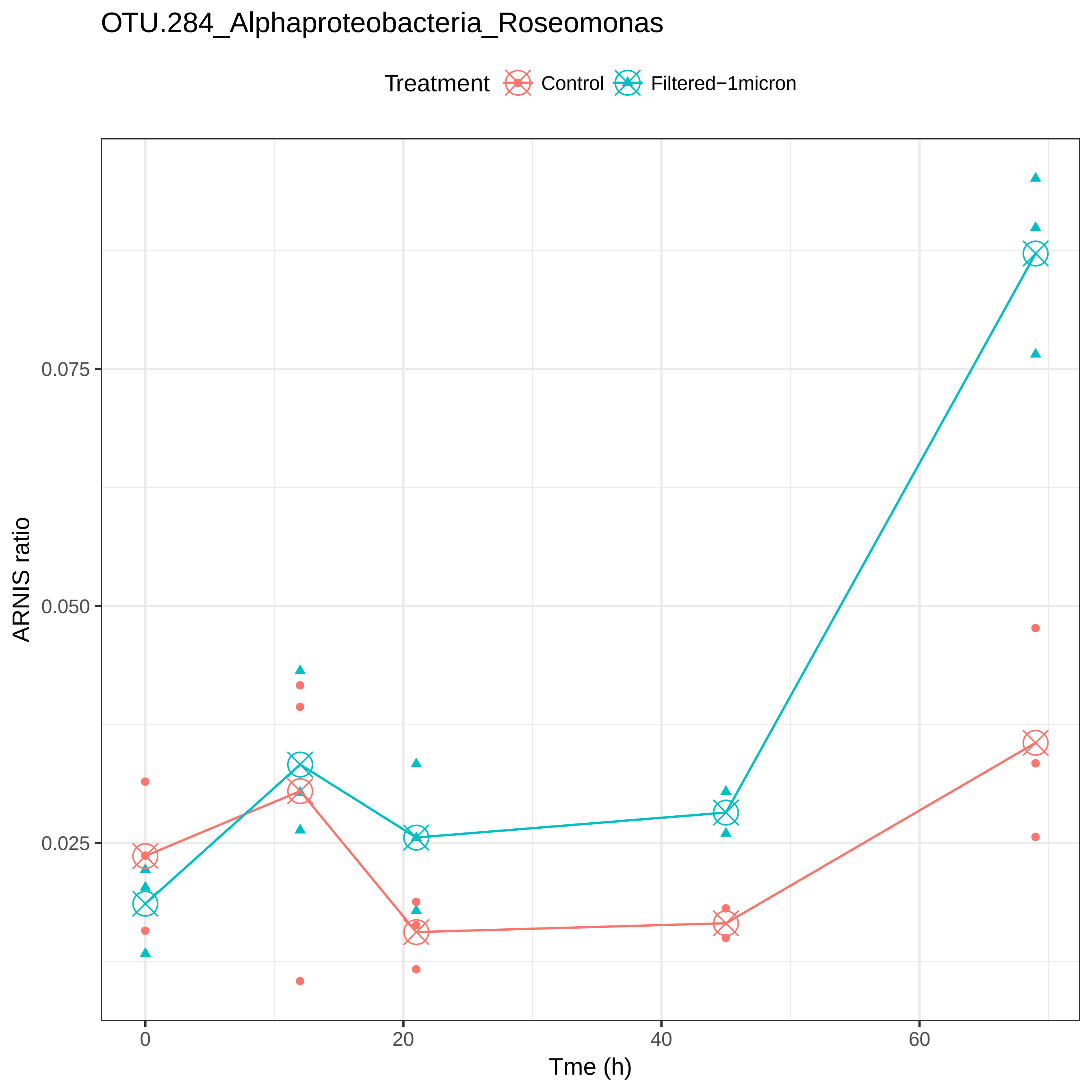

OTU.284_Alphaproteobacteria_Roseomonas
Treatment
Control
Filtered−1micron
ARNIS ratio
0.025
0.050
0.075
0
20
40
60
Tme (h)

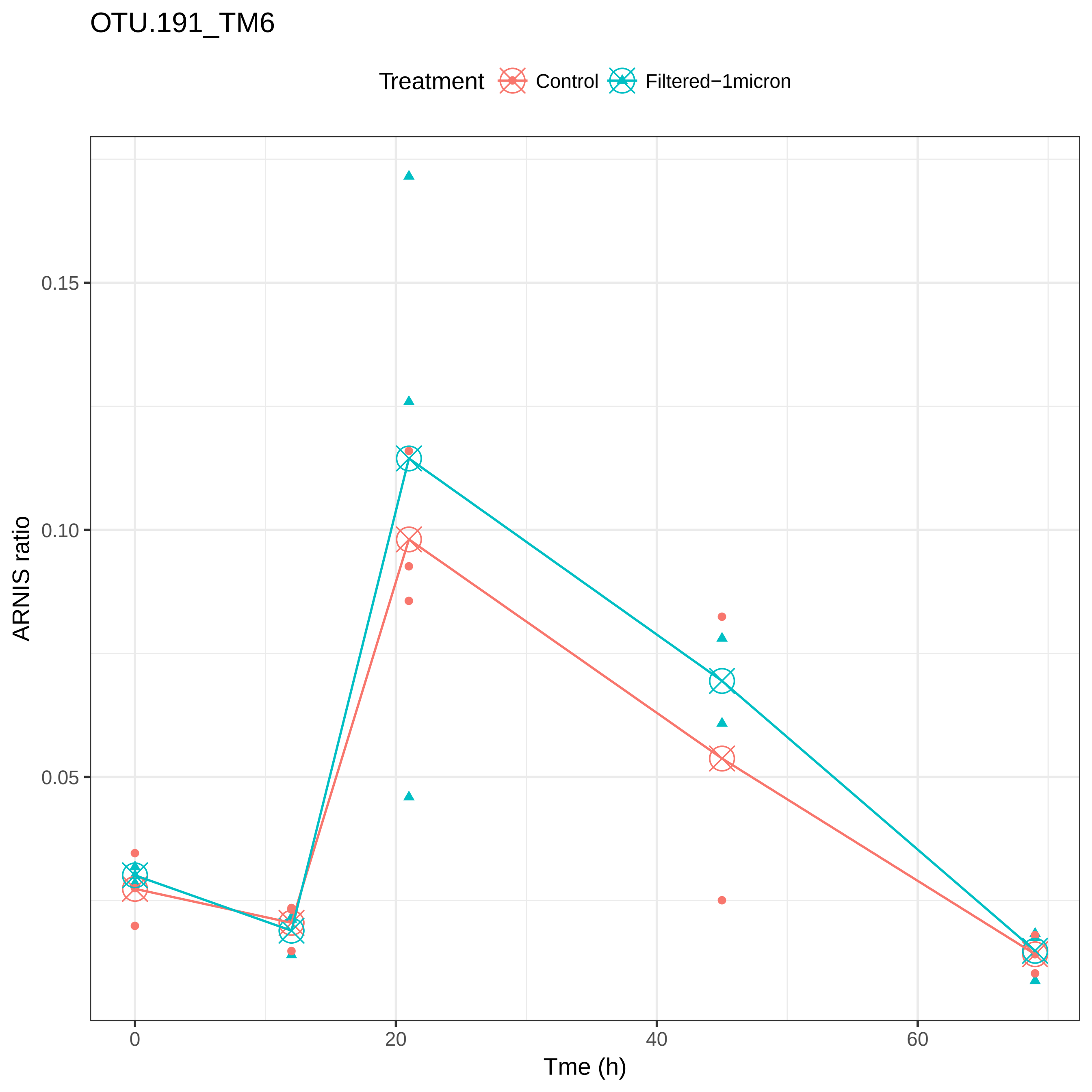
OTU.191_TM6
Treatment
Control
Filtered−1micron
ARNIS ratio
0.15
0.10
0.05
0
20
40
60
Tme (h)

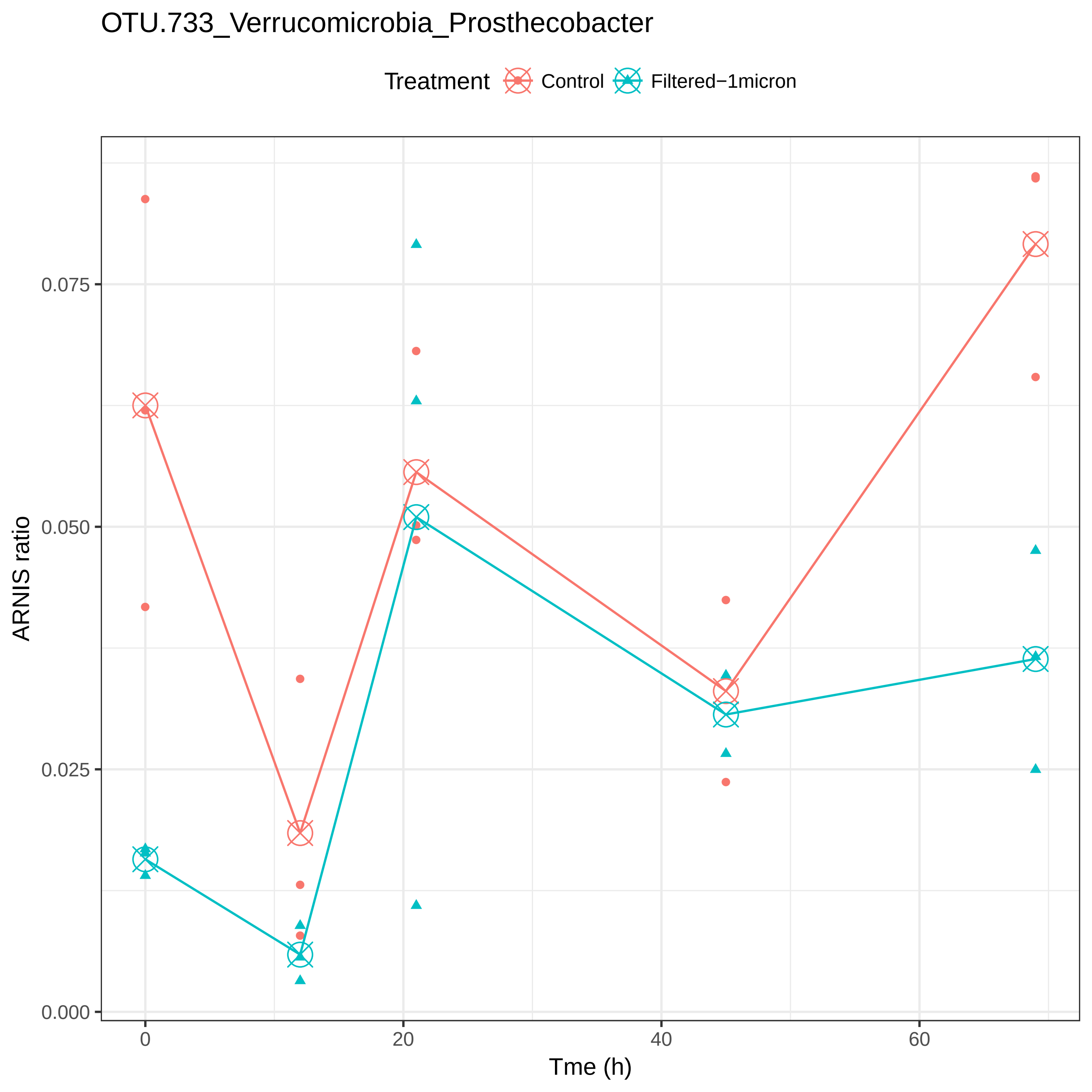
OTU.733_Verrucomicrobia_Prosthecobacter
Treatment
Control
Filtered−1micron
ARNIS ratio
0.000
0.025
0.050
0.075
0
20
40
60
Tme (h)

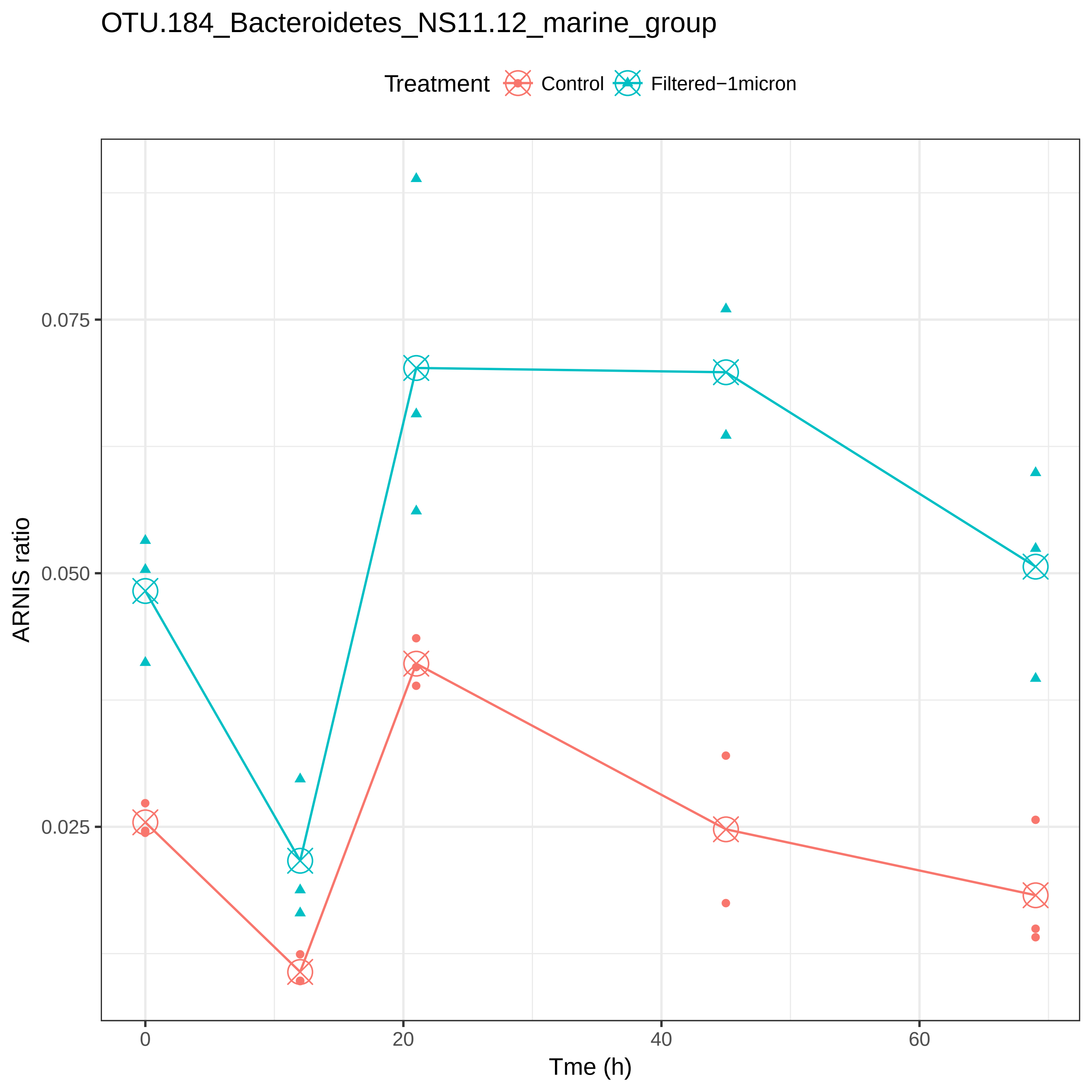

OTU.184_Bacteroidetes_NS11.12_marine_group
Treatment
Control
Filtered−1micron
ARNIS ratio
0.075
0.050
0.025
0
20
40
60
Tme (h)

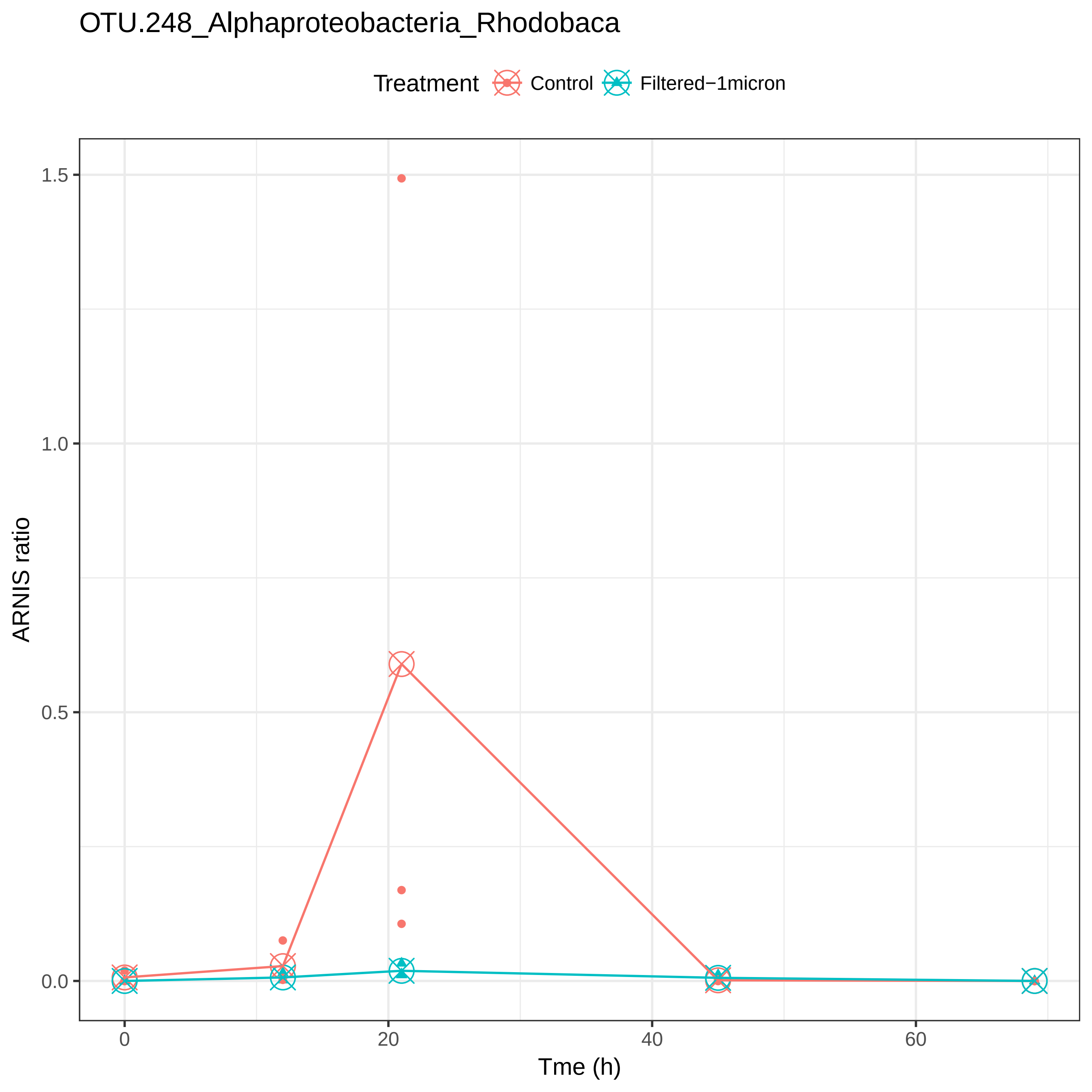

OTU.248_Alphaproteobacteria_Rhodobaca
Treatment
Control
Filtered−1micron
ARNIS ratio
1.5
1.0
0.5
0.0
0
20
40
60
Tme (h)

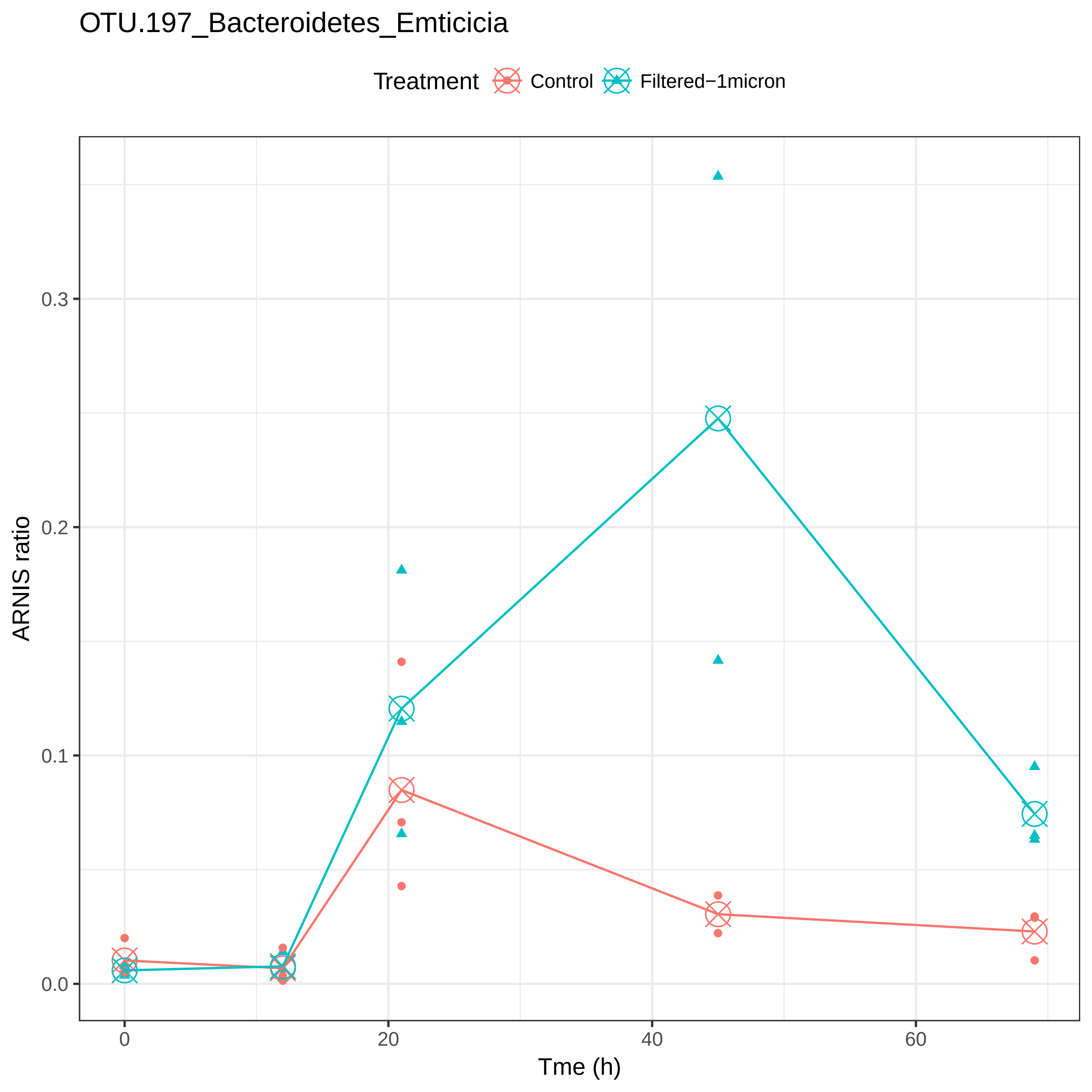

OTU.197_Bacteroidetes_Emticicia
Treatment
Control
Filtered−1micron
ARNIS ratio
0.0
0.1
0.2
0.3
0
20
40
60
Tme (h)

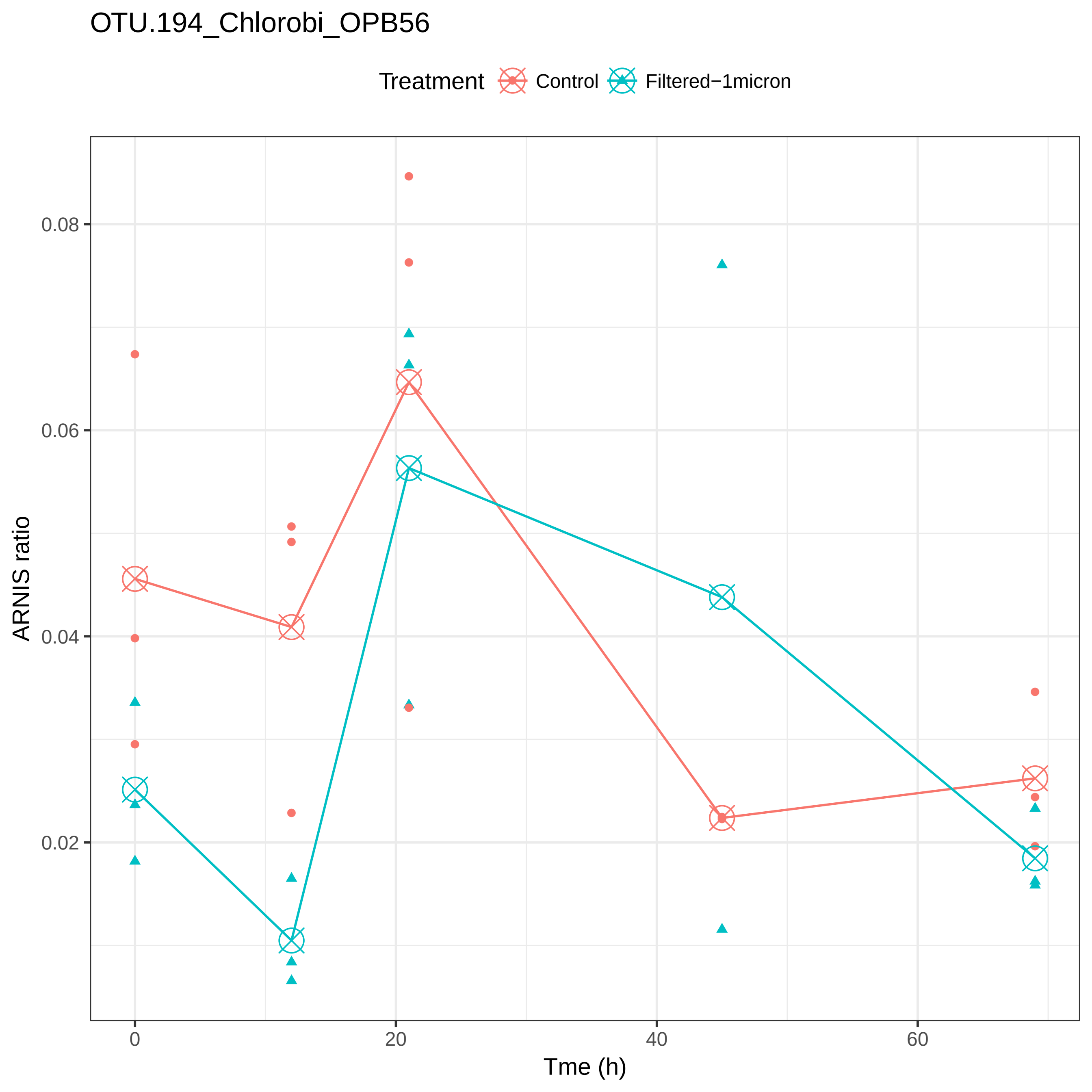

OTU.194_Chlorobi_OPB56
Treatment
Control
Filtered−1micron
ARNIS ratio
0.08
0.06
0.04
0.02
0
20
40
60
Tme (h)

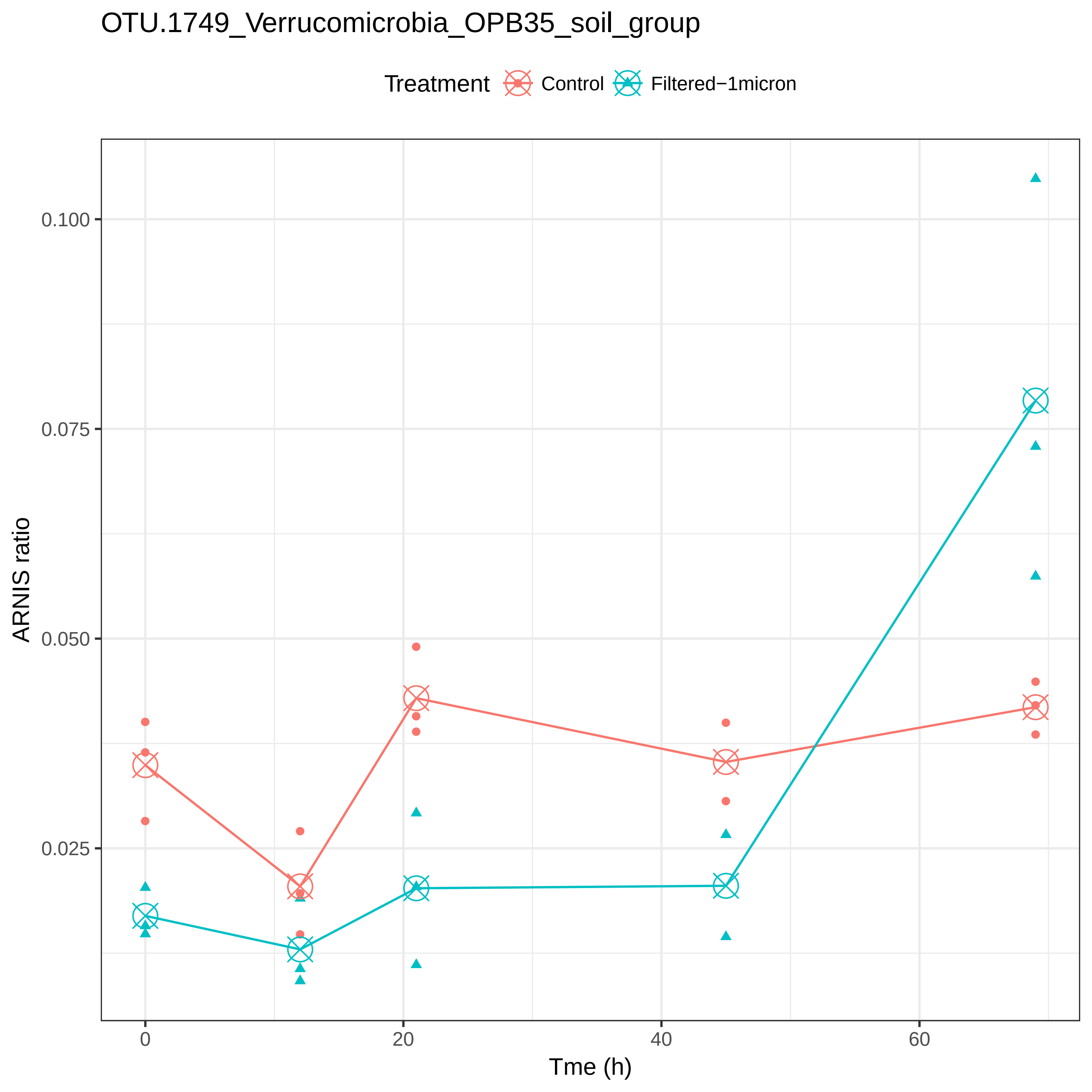

OTU.1749_Verrucomicrobia_OPB35_soil_group
Treatment
Control
Filtered−1micron
ARNIS ratio
0.100
0.075
0.050
0.025
0
20
40
60
Tme (h)

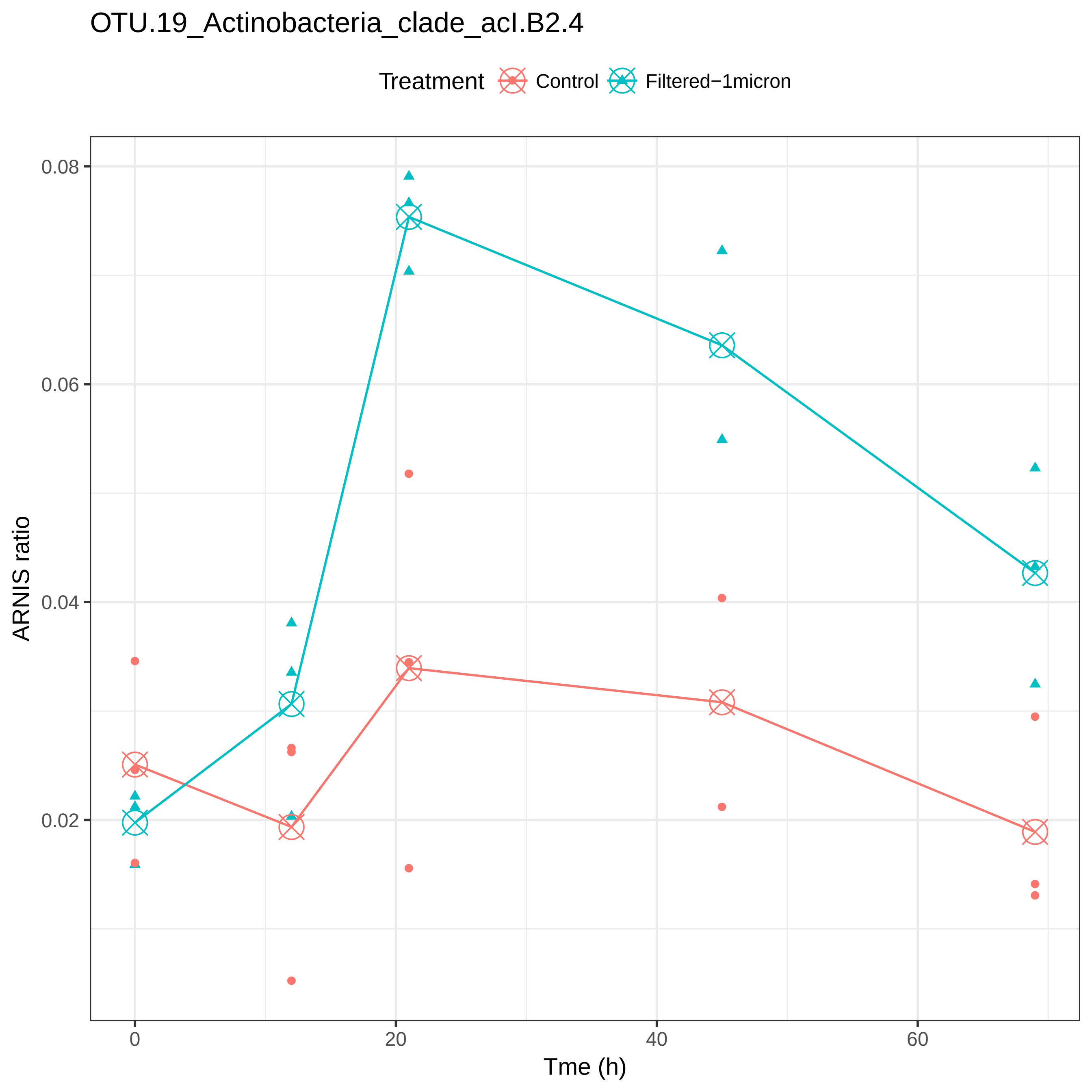
OTU.19_Actinobacteria_clade_acI.B2.4
Treatment
Control
Filtered−1micron
ARNIS ratio
0.08
0.06
0.04
0.02
Tme (h)
0
20
40
60

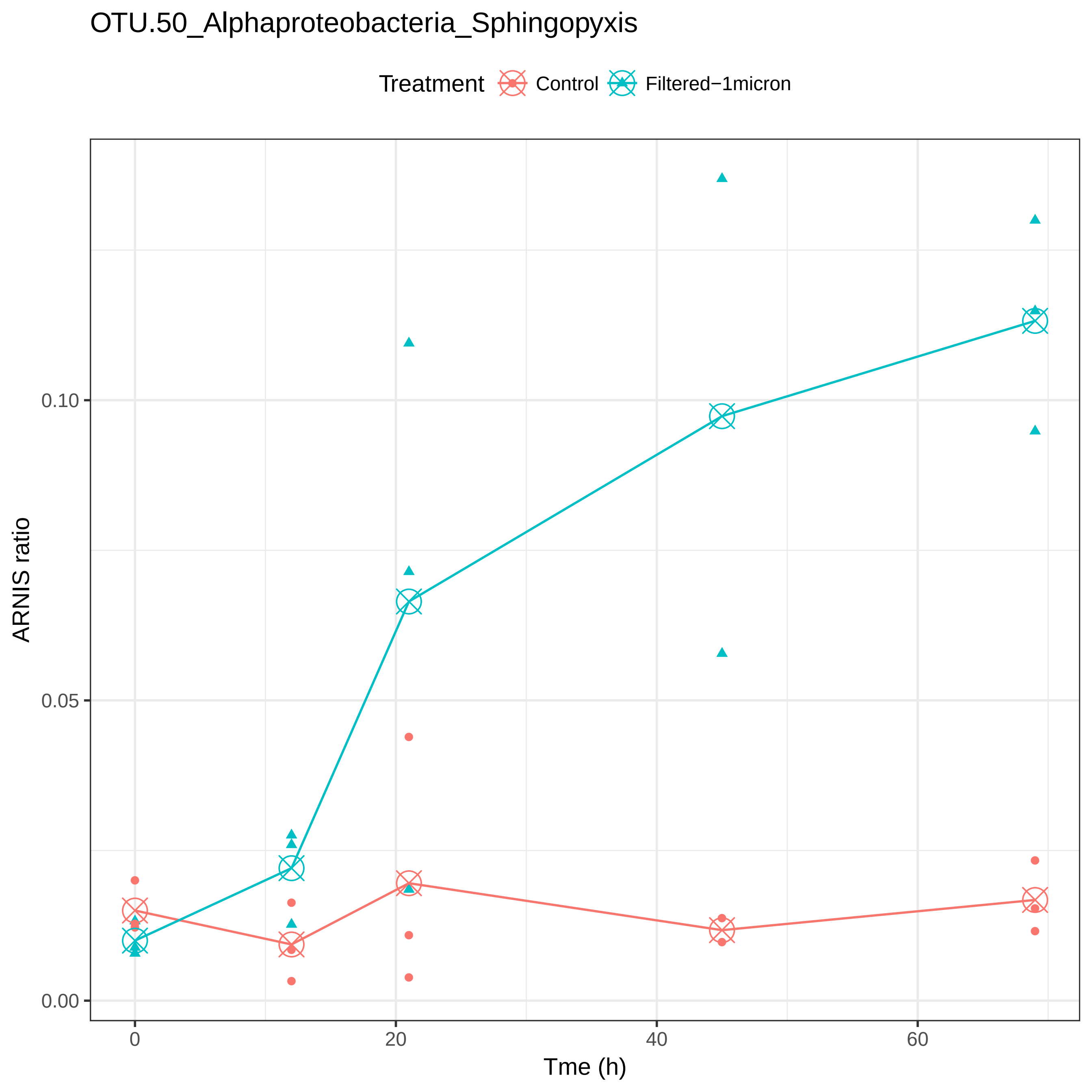

OTU.50_Alphaproteobacteria_Sphingopyxis
Treatment
Control
Filtered−1micron
ARNIS ratio
0.00
0.05
0.10
0
20
40
60
Tme (h)

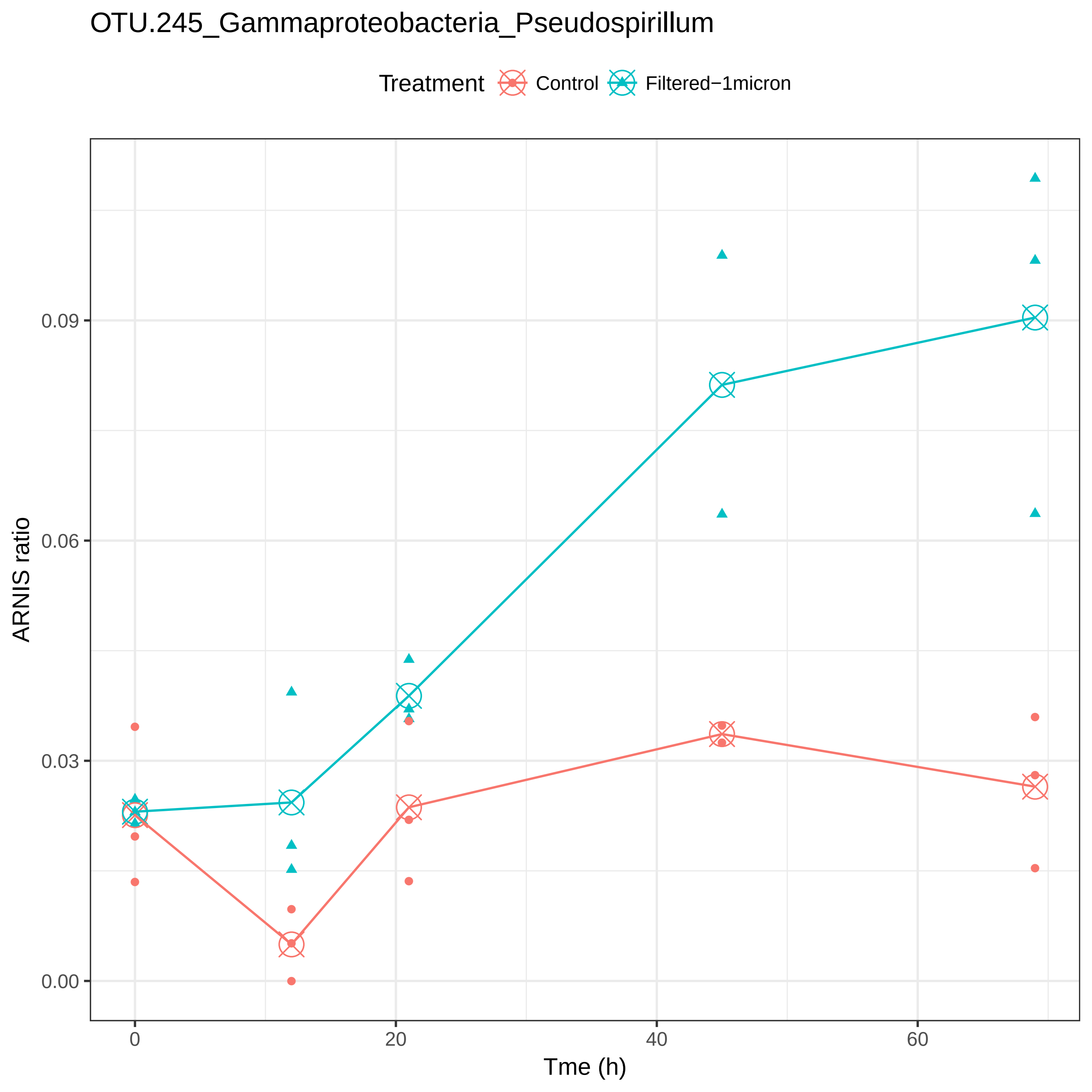

OTU.245_Gammaproteobacteria_Pseudospirillum
Treatment
Control
Filtered−1micron
ARNIS ratio
0.00
0.03
0.06
0.09
0
20
40
60
Tme (h)

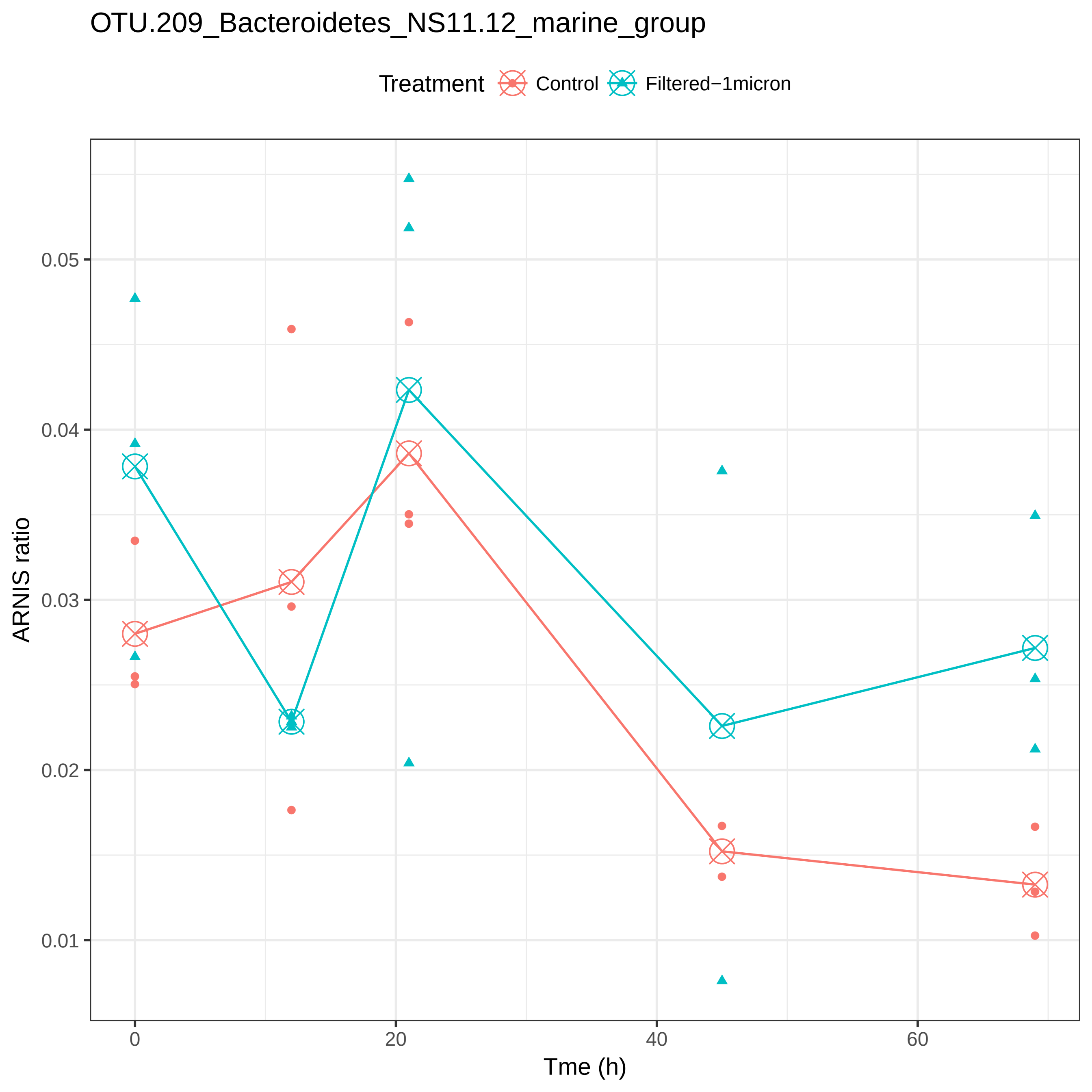
OTU.209_Bacteroidetes_NS11.12_marine_group
Treatment
Control
Filtered−1micron
ARNIS ratio
0.05
0.04
0.03
0.02
0.01
0
20
40
60
Tme (h)

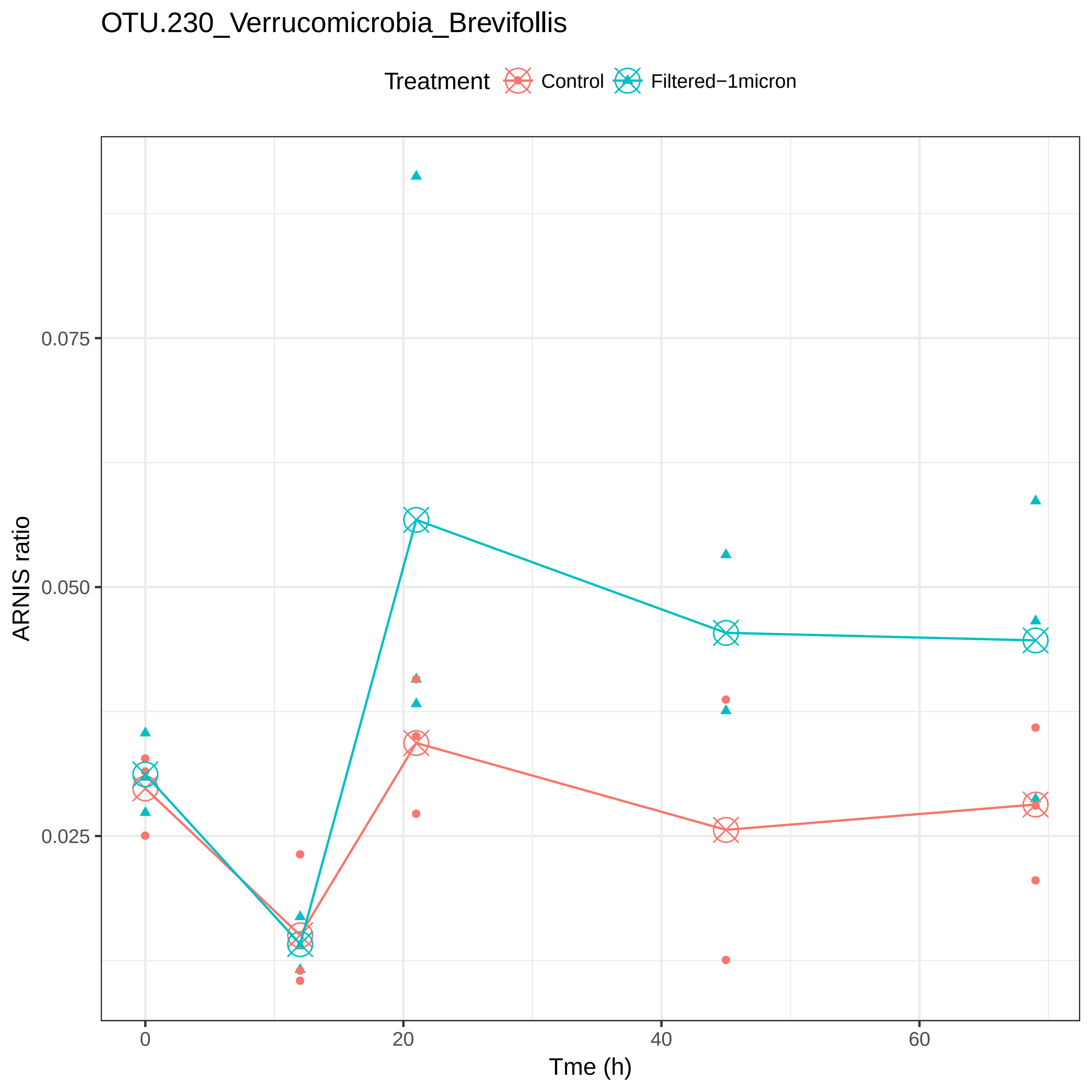

OTU.230_Verrucomicrobia_Brevifollis
Treatment
Control
Filtered−1micron
ARNIS ratio
0.075
0.050
0.025
0
20
40
60
Tme (h)

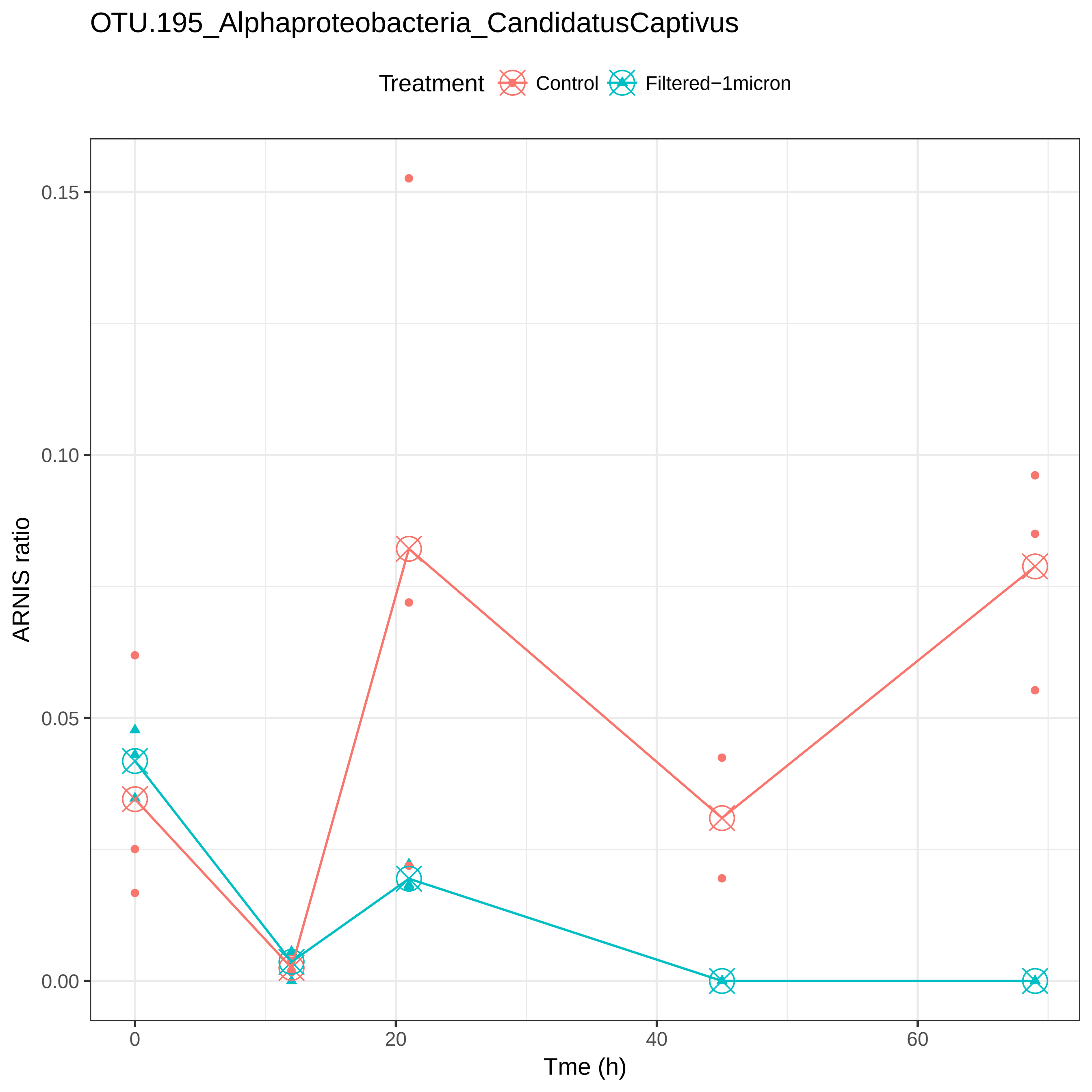

OTU.195_Alphaproteobacteria_CandidatusCaptivus
Treatment
Control
Filtered−1micron
ARNIS ratio
0.15
0.10
0.05
0.00
0
20
40
60
Tme (h)

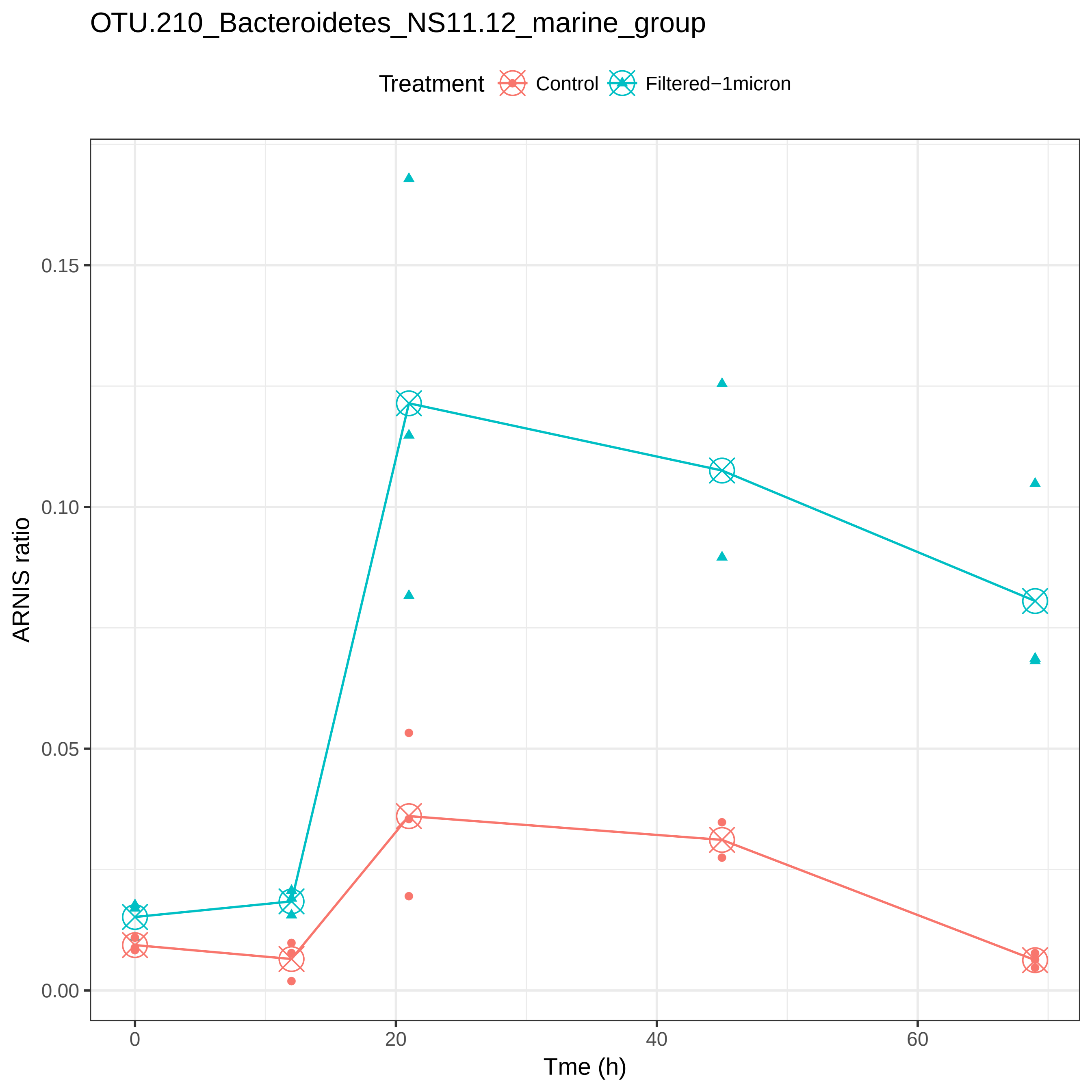
OTU.210_Bacteroidetes_NS11.12_marine_group
Treatment
Control
Filtered−1micron
0.15
0.10
0.05
0.00
ARNIS ratio
0
20
40
60
Tme (h)

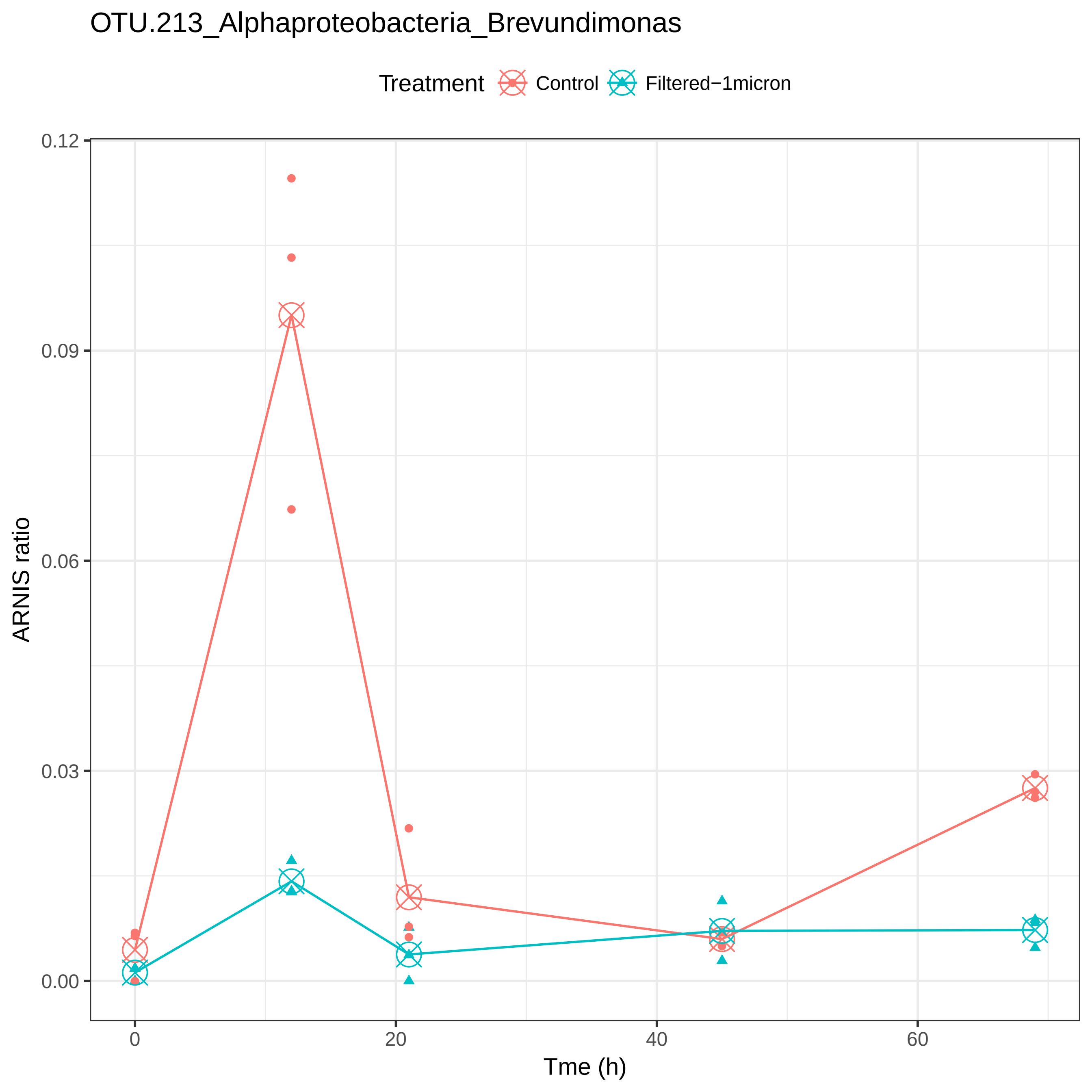
OTU.213_Alphaproteobacteria_Brevundimonas
Treatment
Control
Filtered−1micron
ARNIS ratio
0.12
0.09
0.06
0.03
0.00
0
20
40
60
Tme (h)

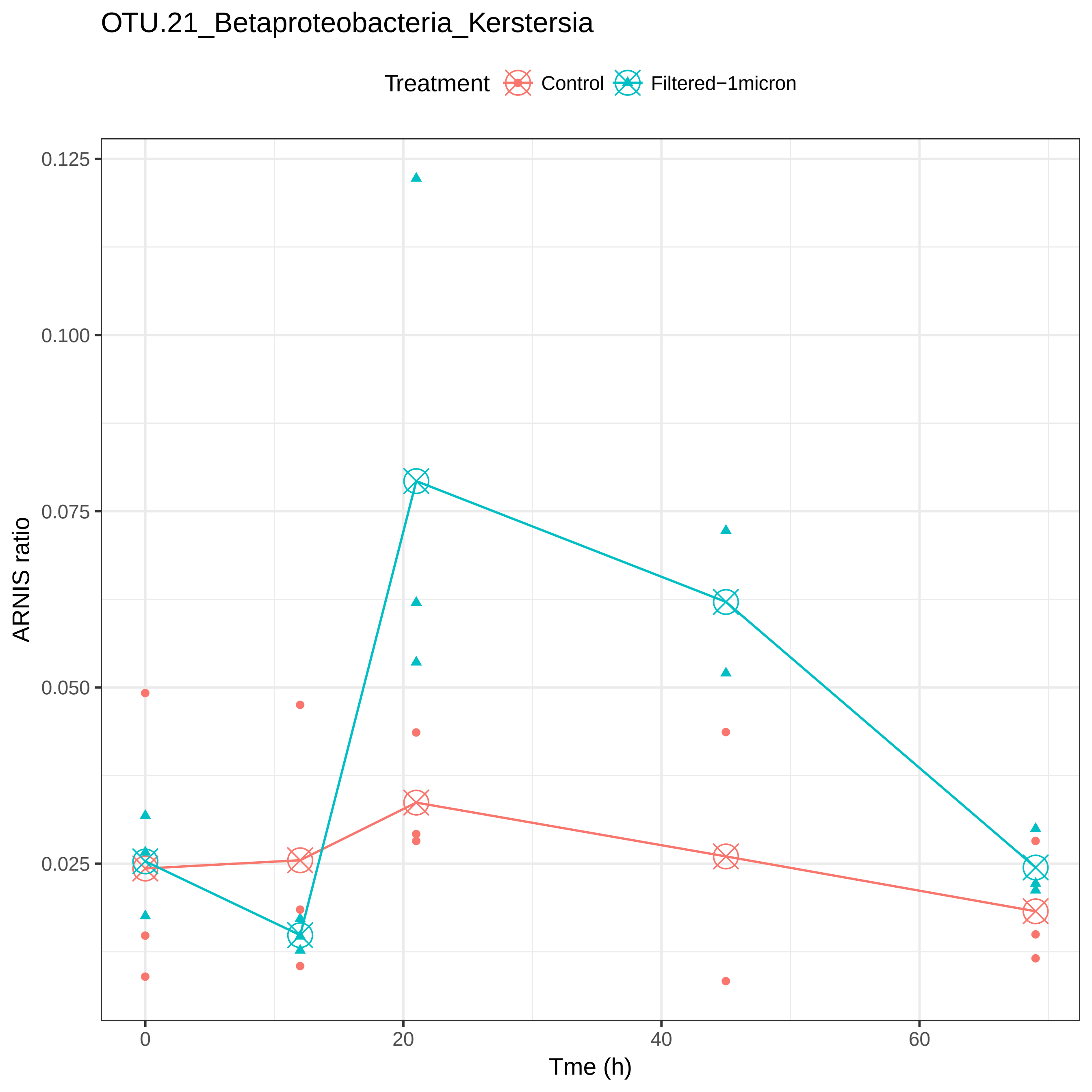
OTU.21_Betaproteobacteria_Kerstersia
Treatment
Control
Filtered−1micron
ARNIS ratio
0.125
0.100
0.075
0.050
0.025
0
20
40
60
Tme (h)

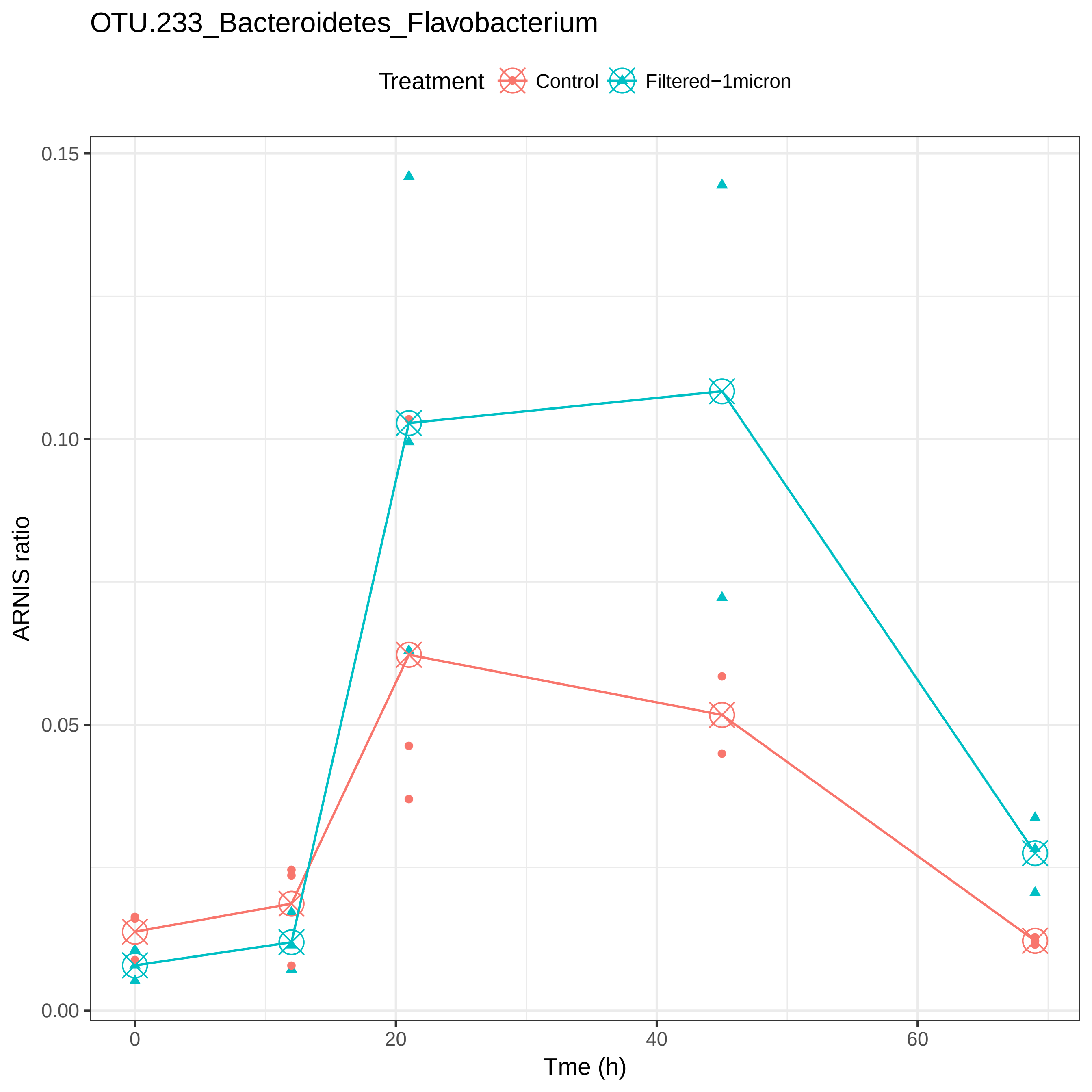

OTU.233_Bacteroidetes_Flavobacterium
Treatment
Control
Filtered−1micron
ARNIS ratio
0.15
0.10
0.05
0.00
0
20
40
60
Tme (h)

OTU.313_Alphaproteobacteria_Phenylobacterium

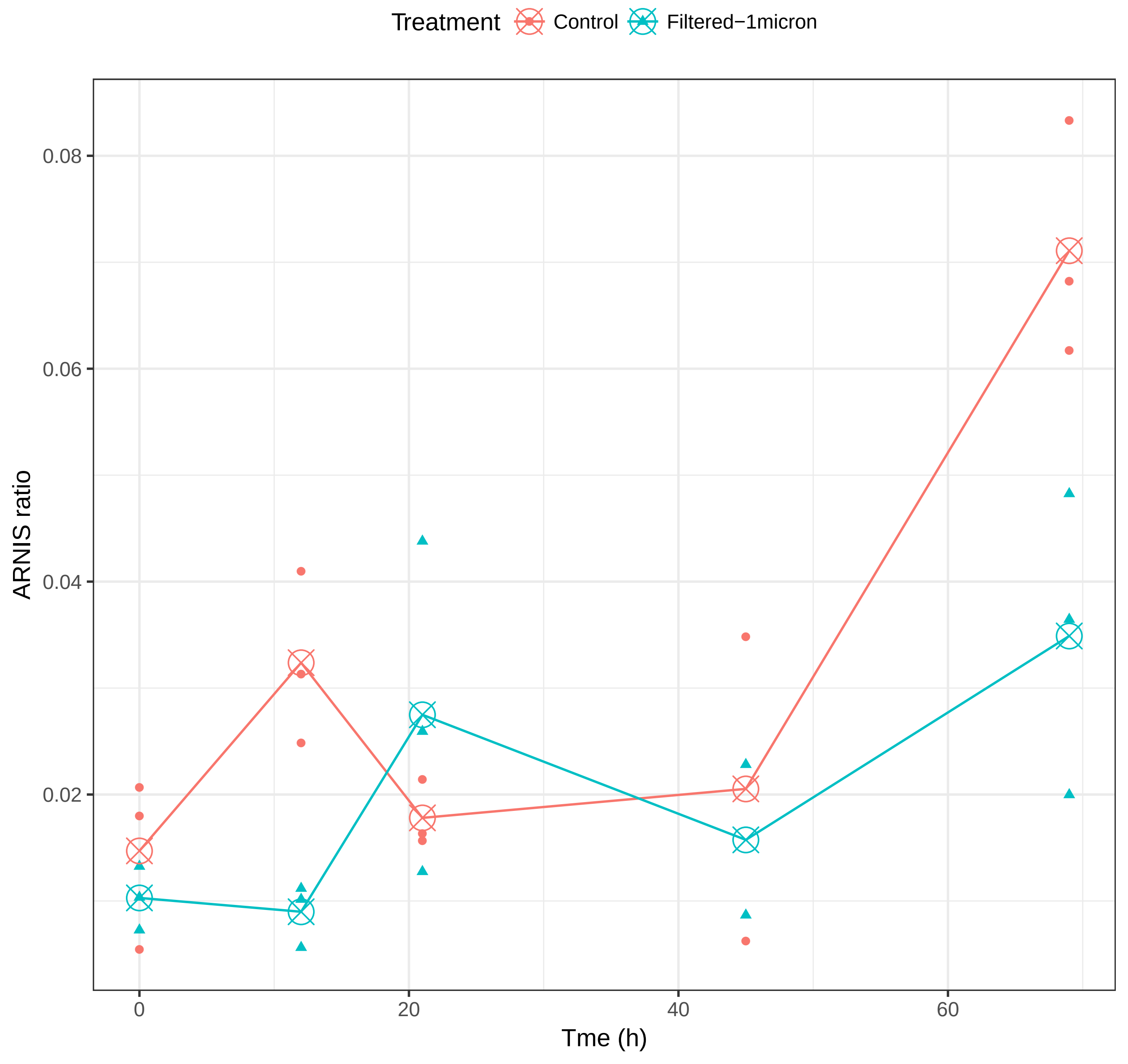

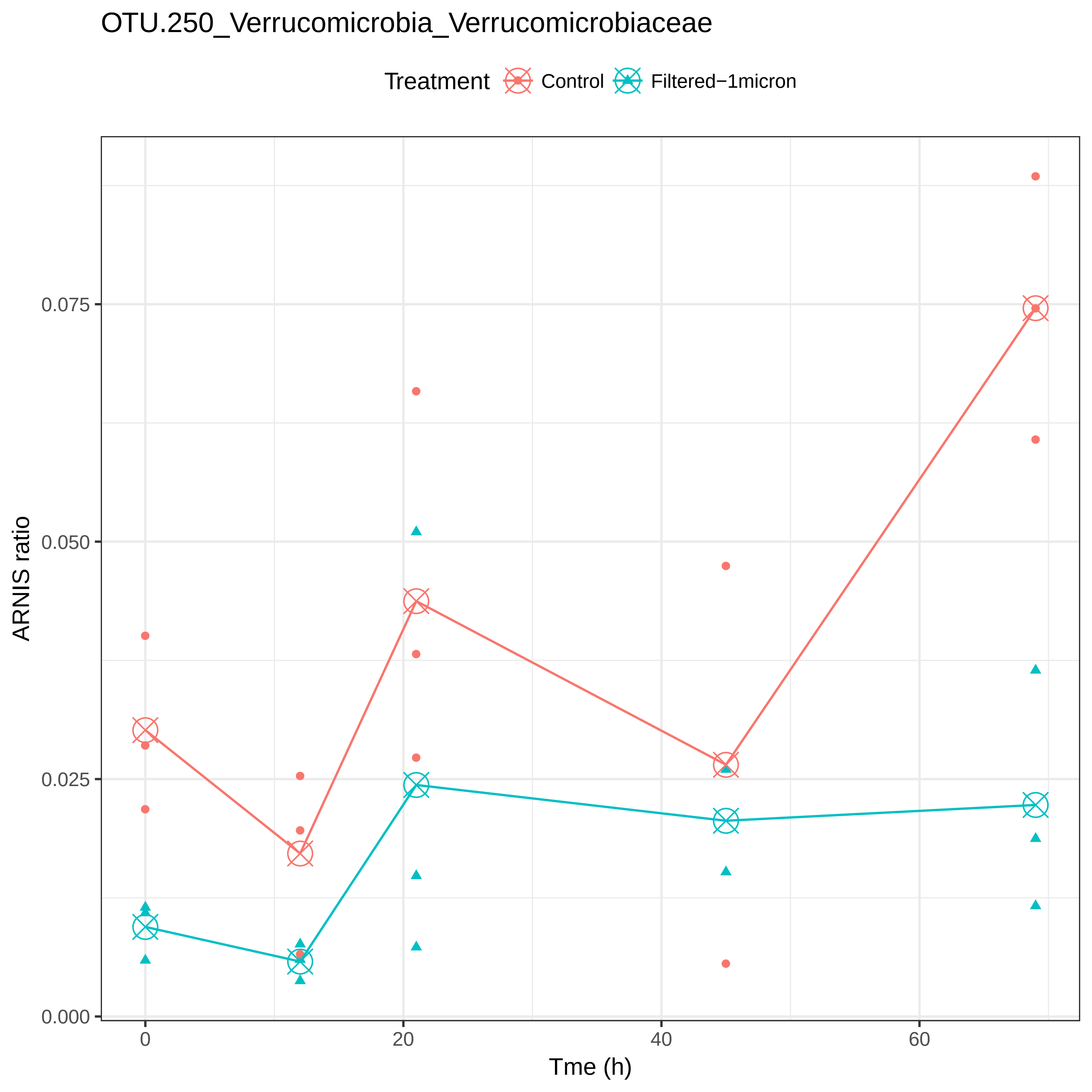

OTU.250_Verrucomicrobia_Verrucomicrobiaceae
Treatment
Control
Filtered−1micron
ARNIS ratio
0.000
0.025
0.050
0.075
0
20
40
60
Tme (h)

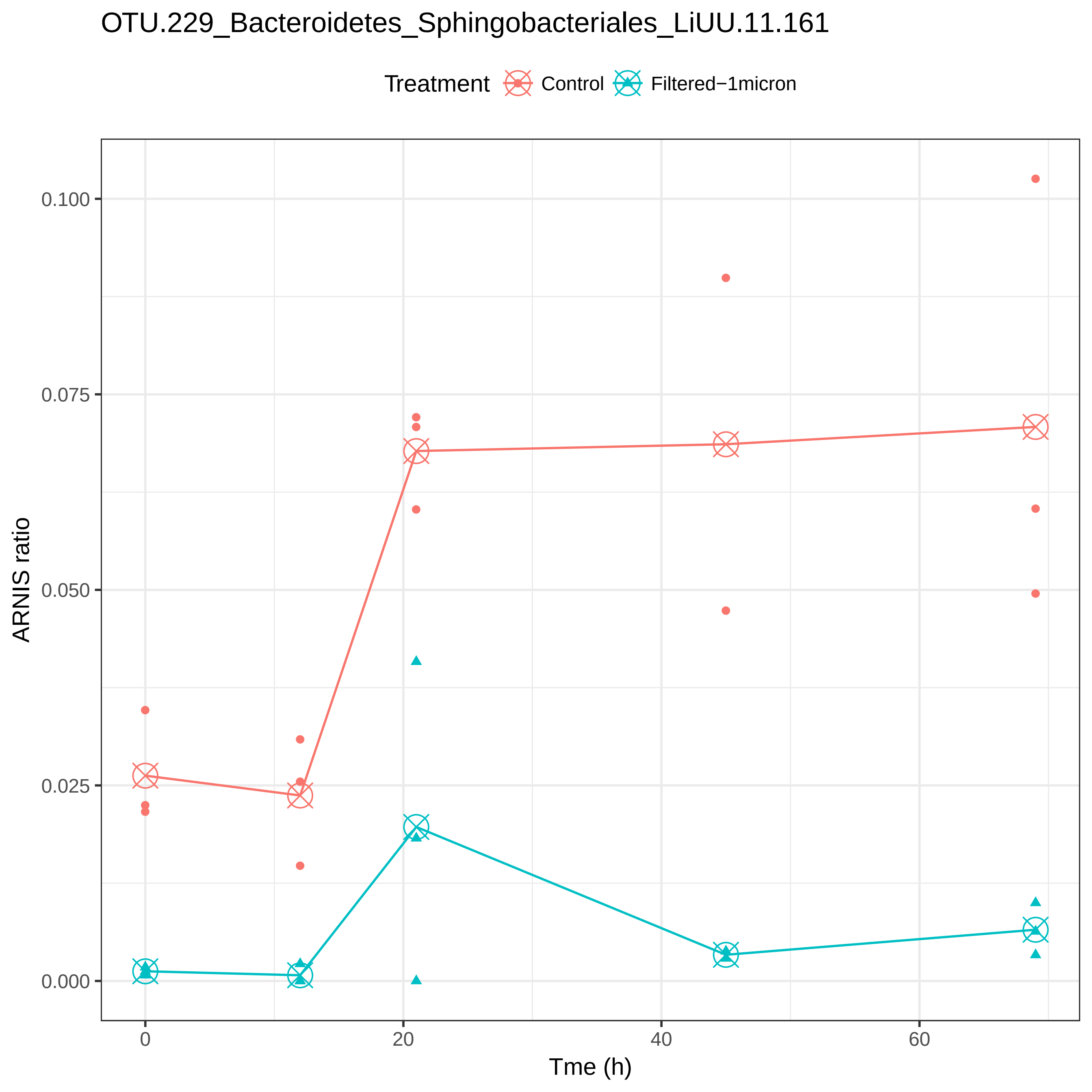

OTU.229_Bacteroidetes_Sphingobacteriales_LiUU.11.161
Treatment
Control
Filtered−1micron
ARNIS ratio
0.100
0.075
0.050
0.025
0.000
0
20
40
60
Tme (h)

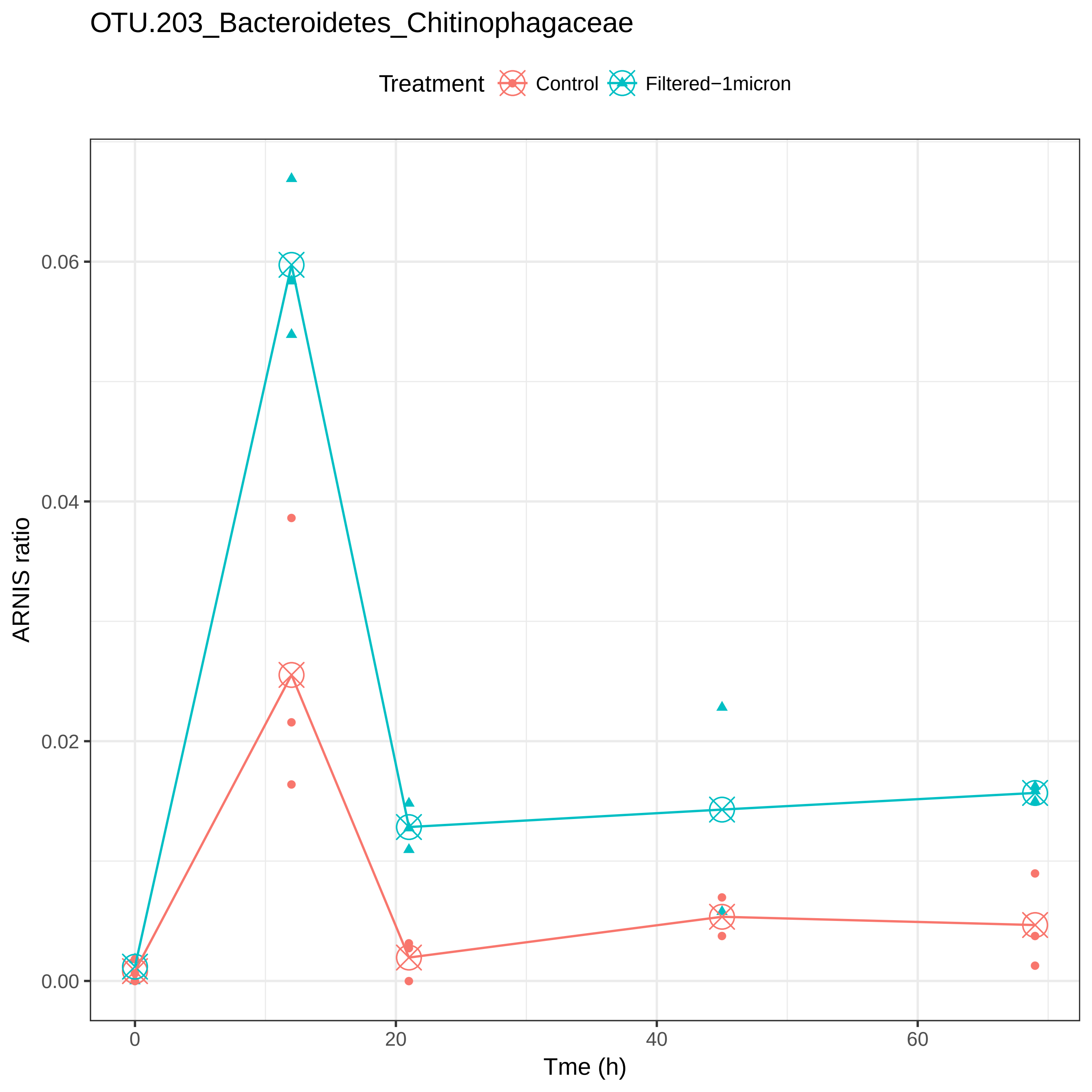
OTU.203_Bacteroidetes_Chitinophagaceae
Treatment
Control
Filtered−1micron
ARNIS ratio
0.00
0.02
0.04
0.06
0
20
40
60
Tme (h)

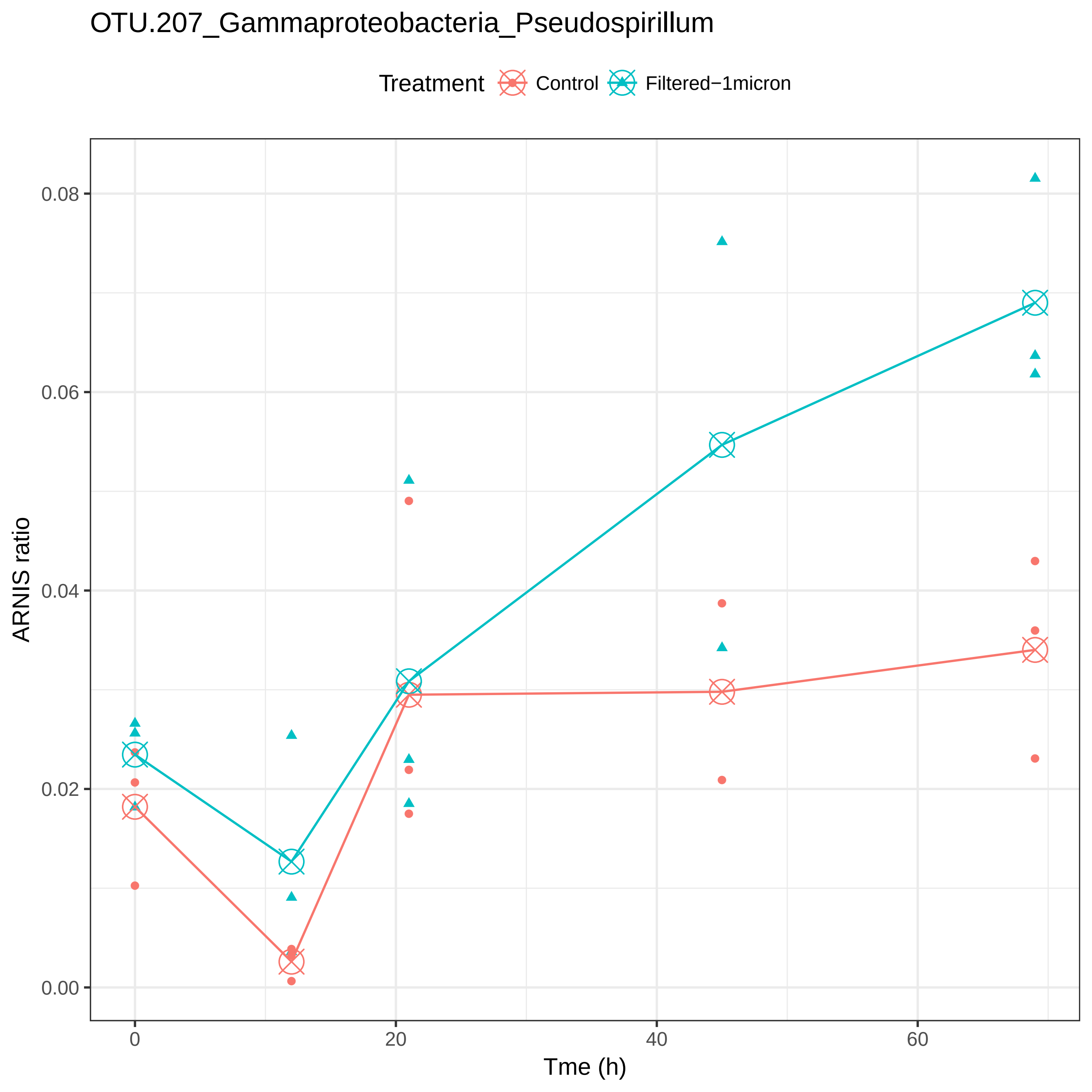
OTU.207_Gammaproteobacteria_Pseudospirillum
Treatment
Control
Filtered−1micron
ARNIS ratio
0.00
0.02
0.04
0.06
0.08
0
20
40
60
Tme (h)

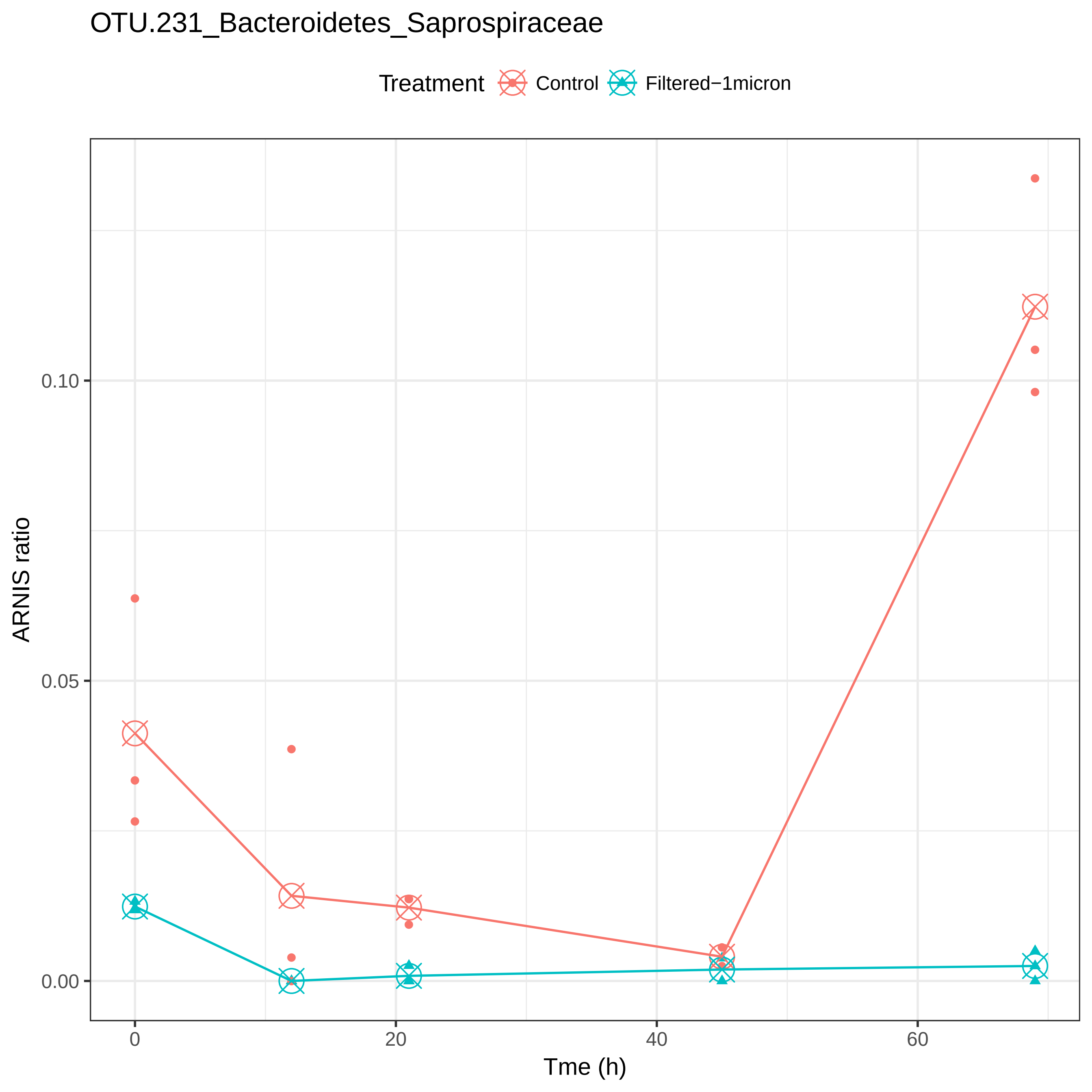
OTU.231_Bacteroidetes_Saprospiraceae
Treatment
Control
Filtered−1micron
ARNIS ratio
0.10
0.05
0.00
0
20
40
60
Tme (h)

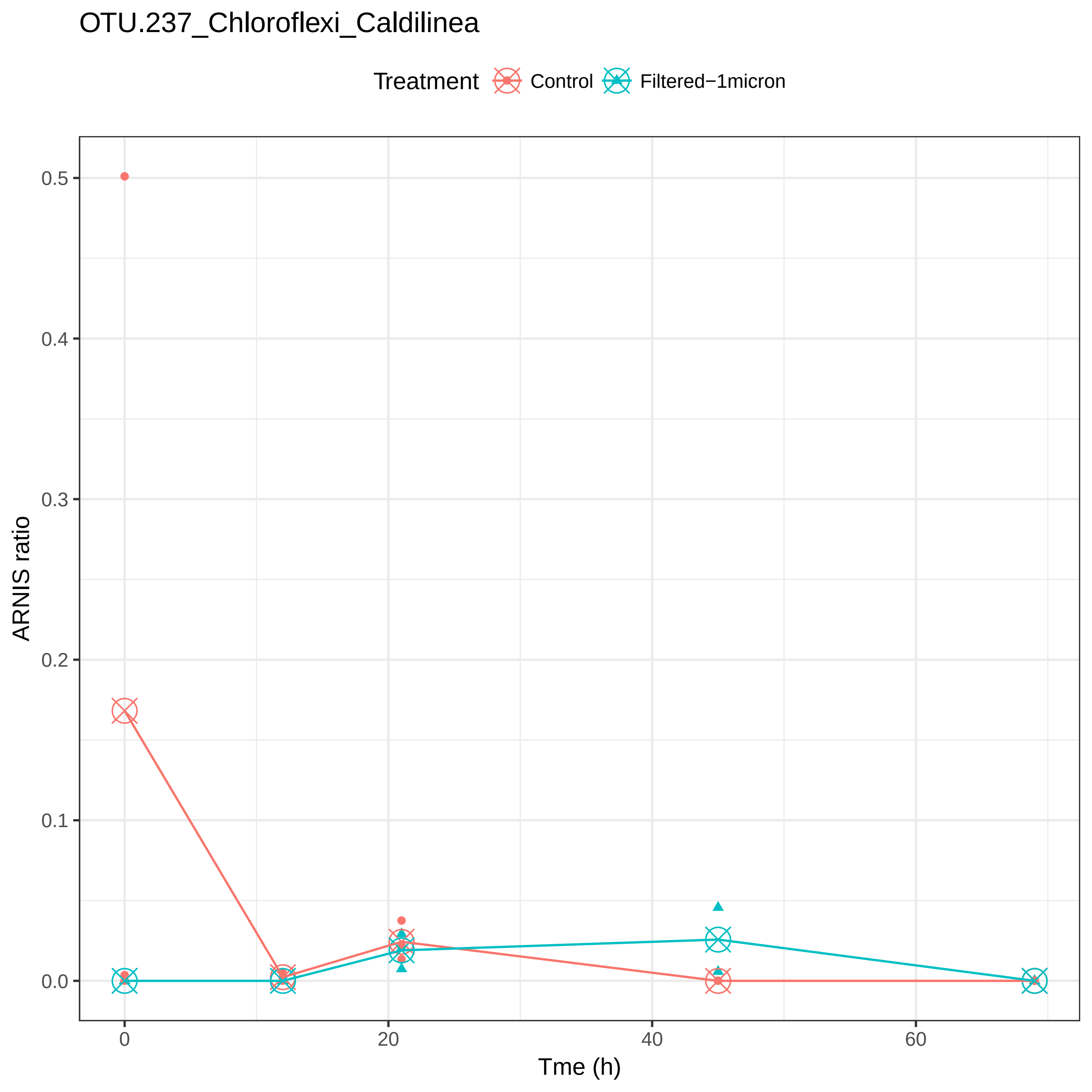

OTU.237_Chloroflexi_Caldilinea
Treatment
Control
Filtered−1micron
ARNIS ratio
0.0
0.1
0.2
0.3
0.4
0.5
0
20
40
60
Tme (h)

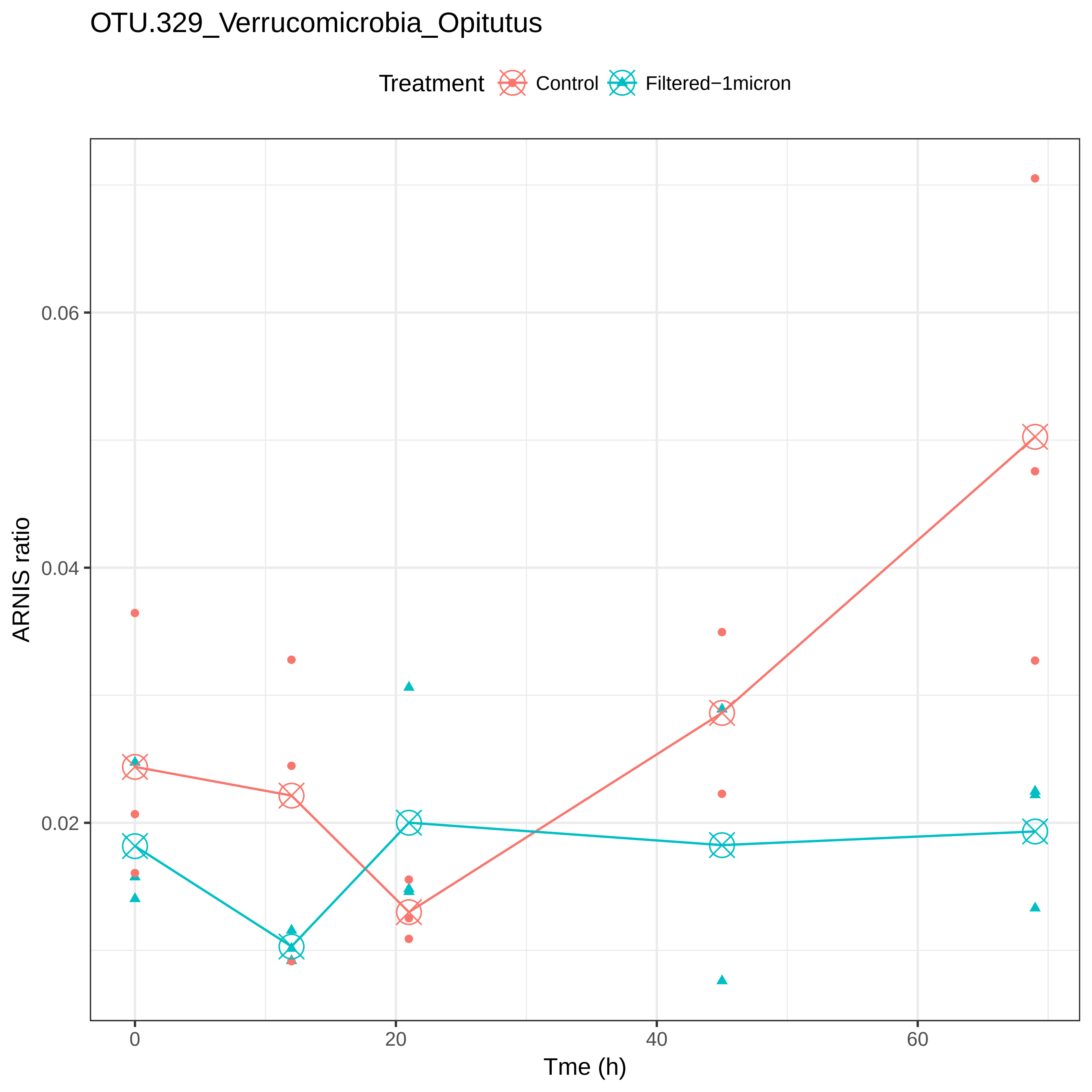

OTU.329_Verrucomicrobia_Opitutus
Treatment
Control
Filtered−1micron
ARNIS ratio
0.06
0.04
0.02
0
20
40
60
Tme (h)

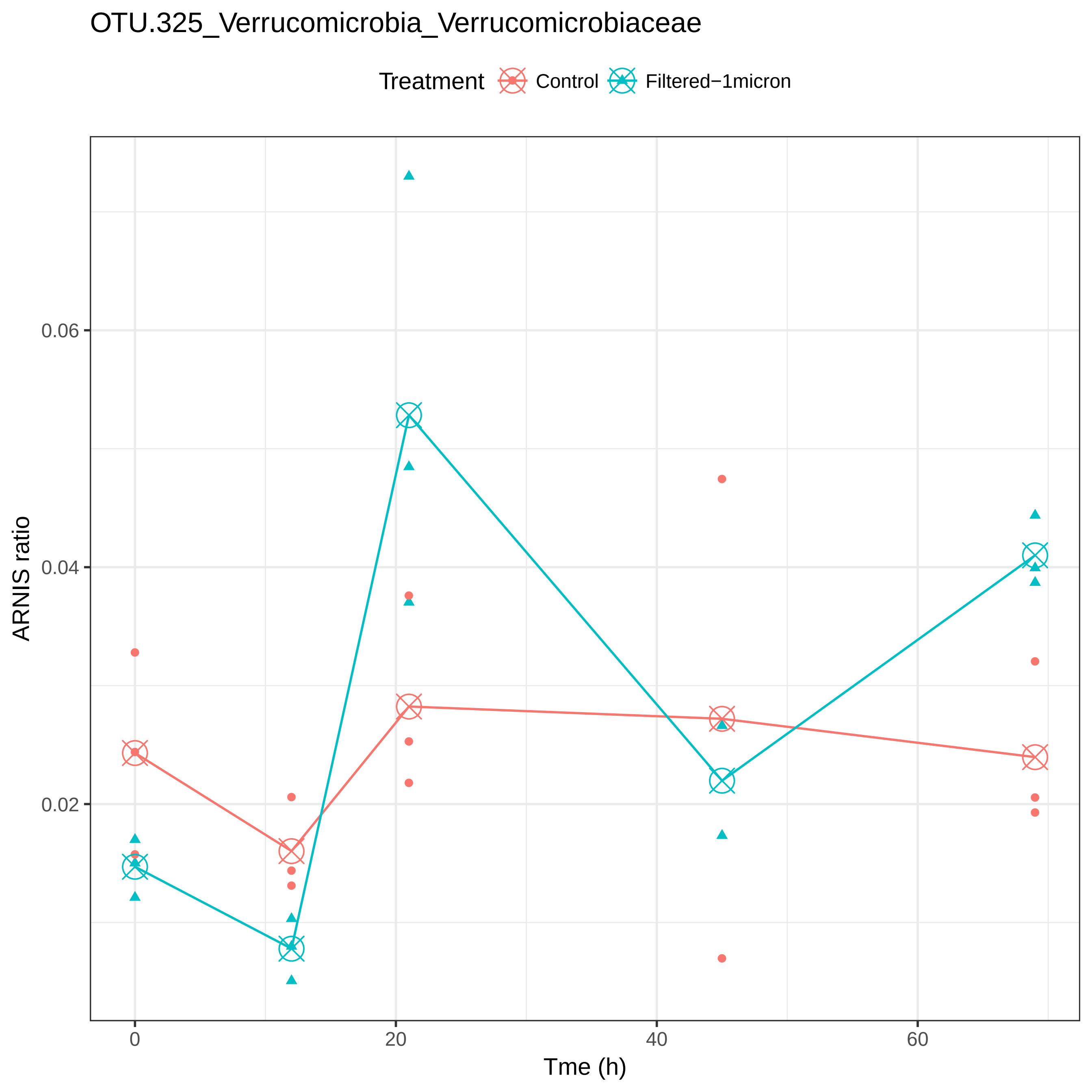

OTU.325_Verrucomicrobia_Verrucomicrobiaceae
Treatment
Control
Filtered−1micron
ARNIS ratio
0.06
0.04
0.02
0
20
40
60
Tme (h)

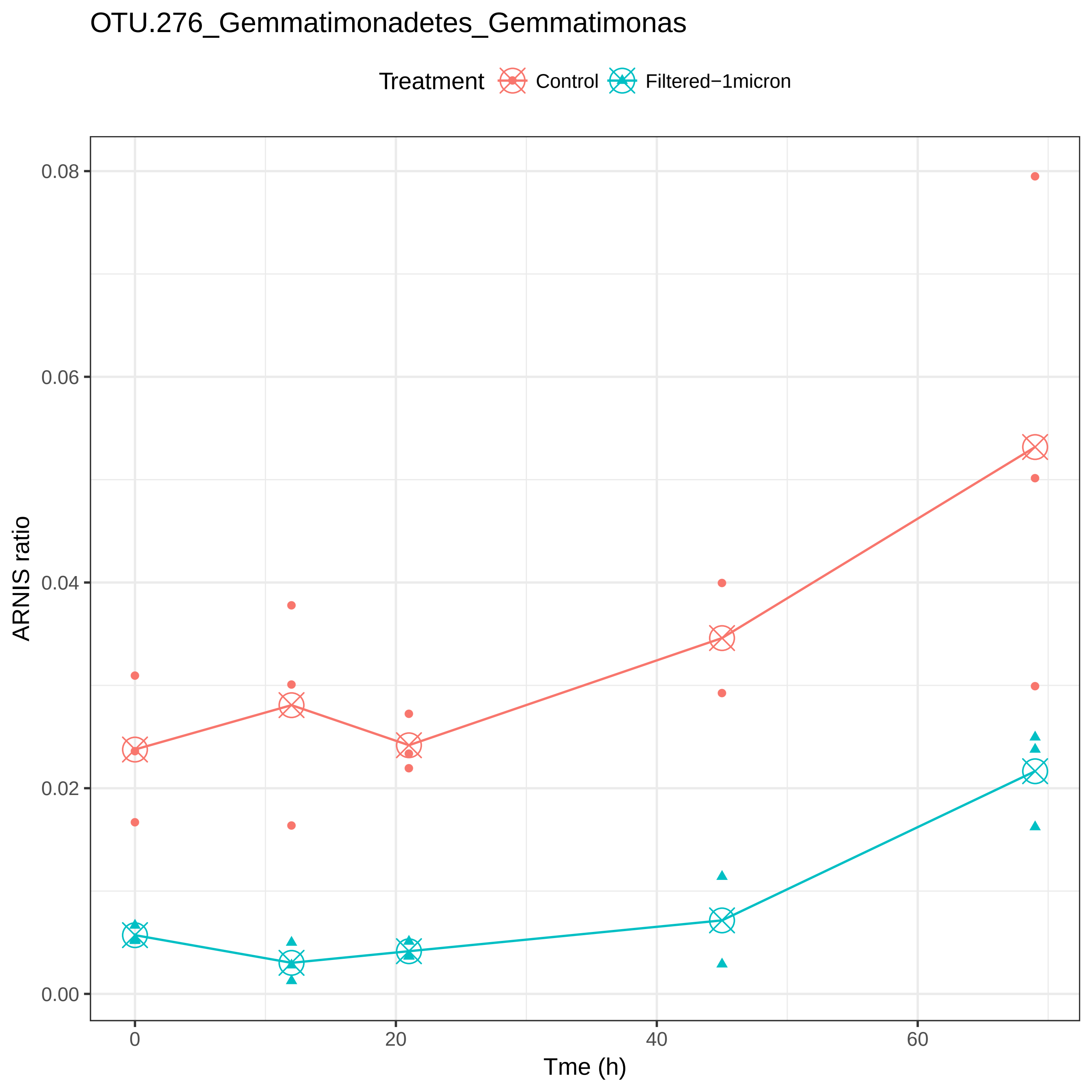

OTU.276_Gemmatimonadetes_Gemmatimonas
Treatment
Control
Filtered−1micron
0.08
0.06
0.04
0.02
0.00
ARNIS ratio
0
20
40
60
Tme (h)

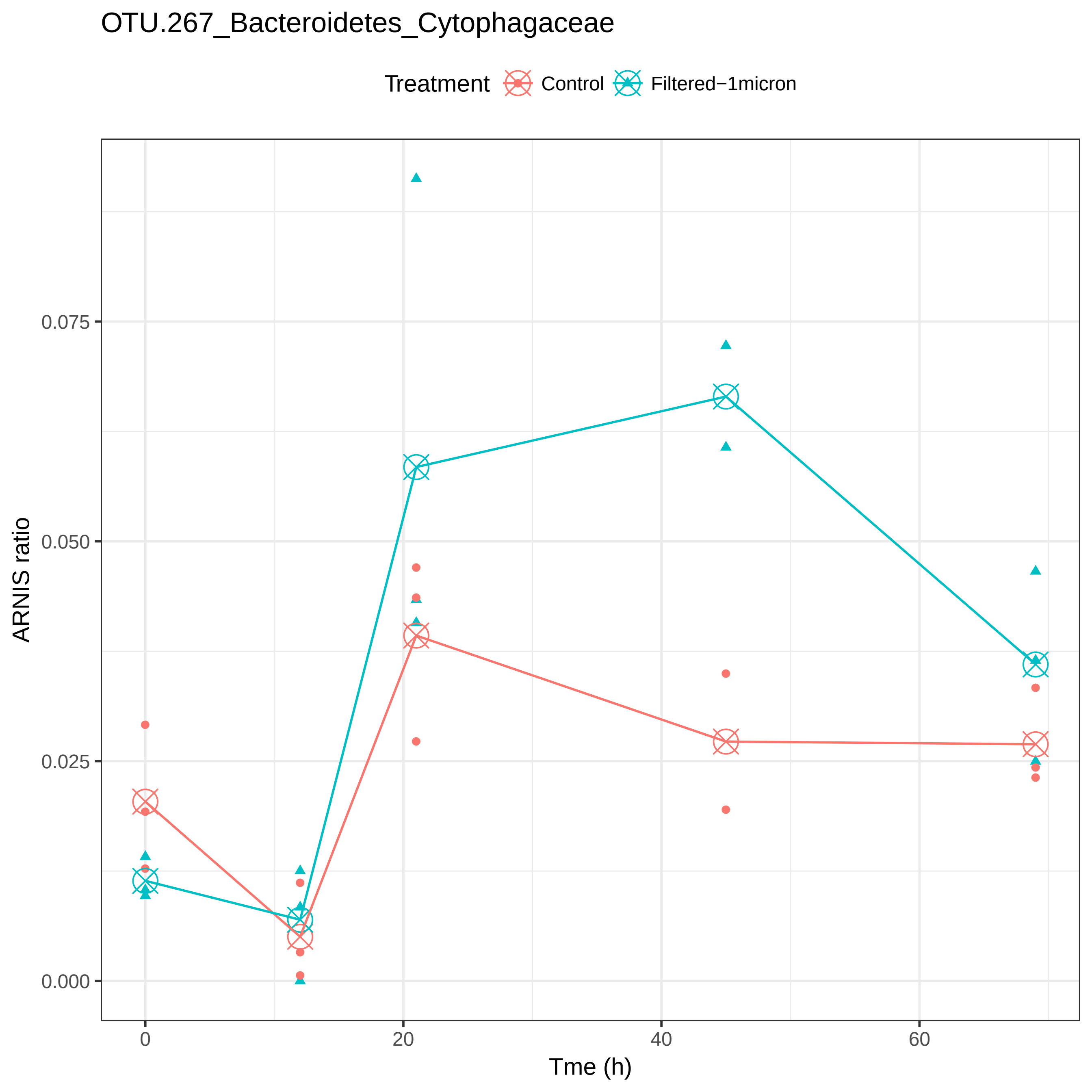

OTU.267_Bacteroidetes_Cytophagaceae
Treatment
Control
Filtered−1micron
ARNIS ratio
0.000
0.025
0.050
0.075
0
20
40
60
Tme (h)

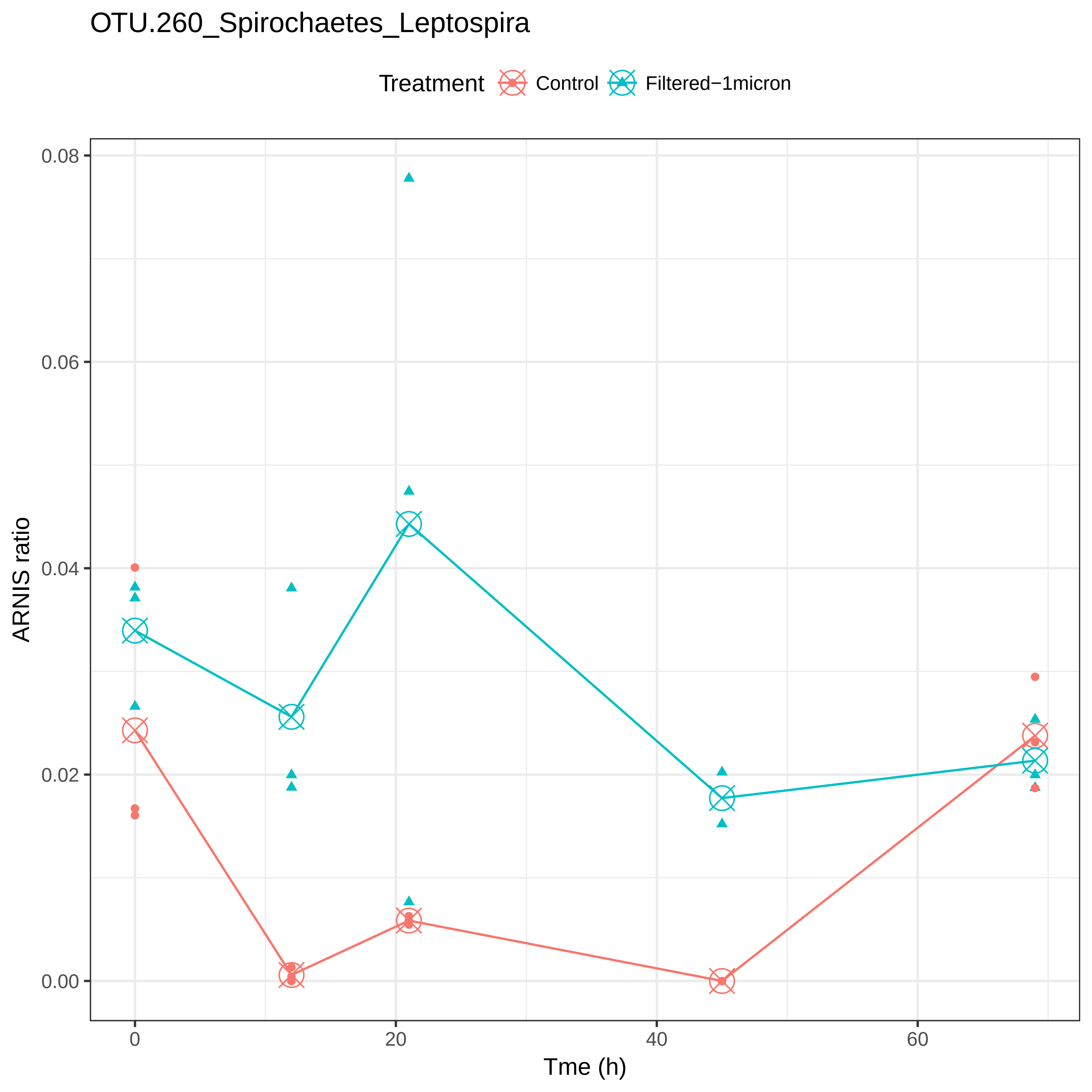

OTU.260_Spirochaetes_Leptospira
Treatment
Control
Filtered−1micron
ARNIS ratio
0.00
0.02
0.04
0.06
0.08
0
20
40
60
Tme (h)

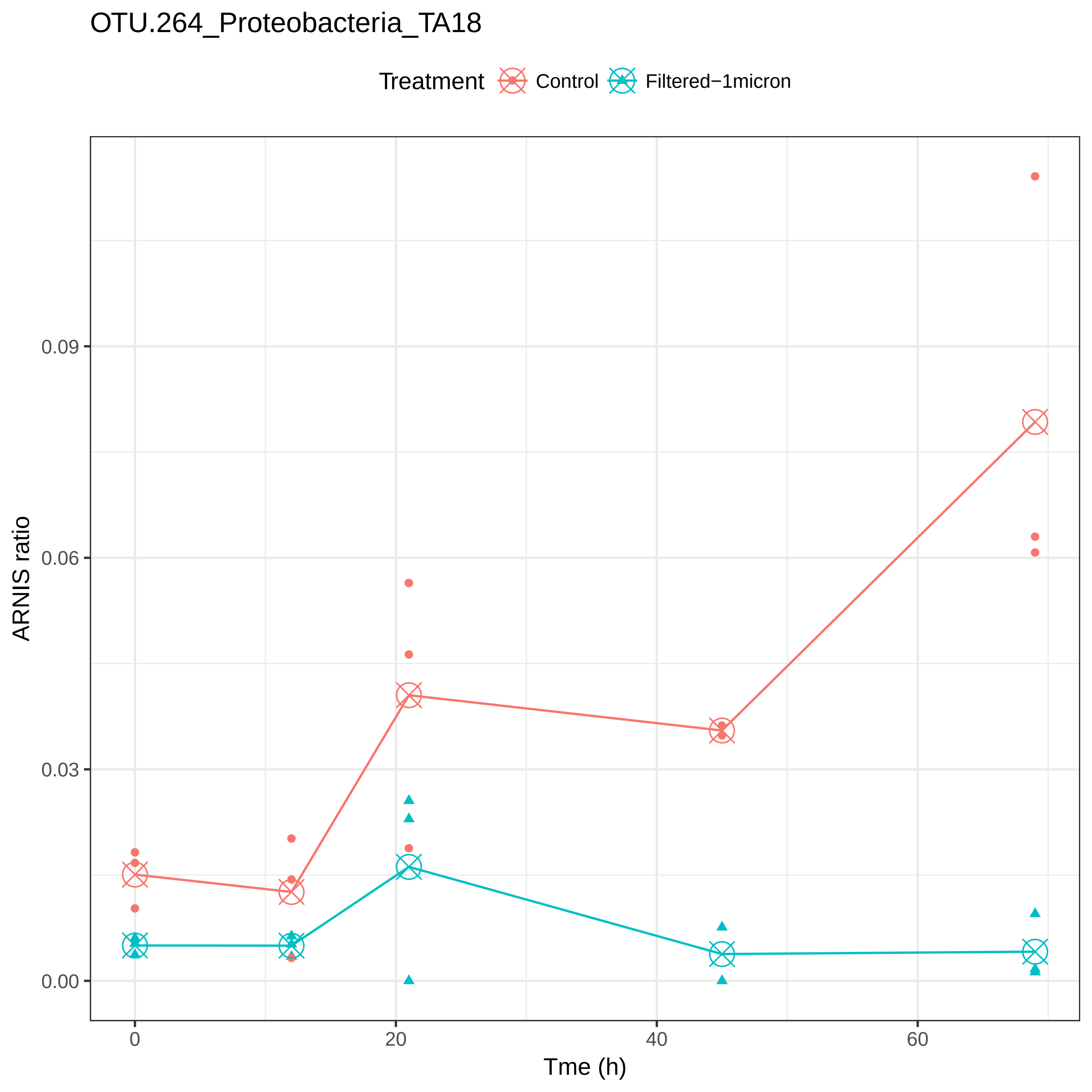
OTU.264_Proteobacteria_TA18
Treatment
Control
Filtered−1micron
ARNIS ratio
0.00
0.03
0.06
0.09
0
20
40
60
Tme (h)

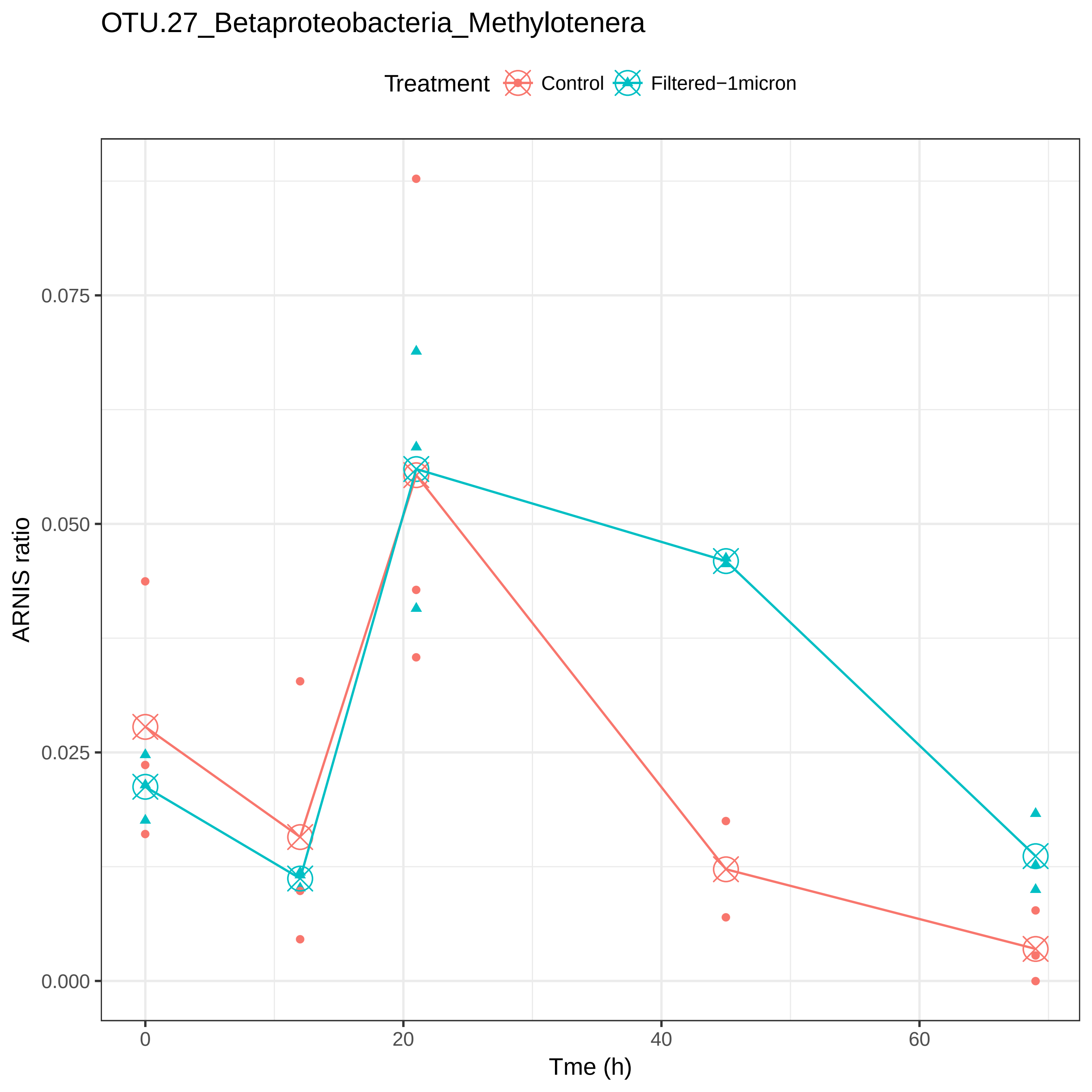

OTU.27_Betaproteobacteria_Methylotenera
Treatment
Control
Filtered−1micron
ARNIS ratio
0.075
0.050
0.025
0.000
0
20
40
60
Tme (h)

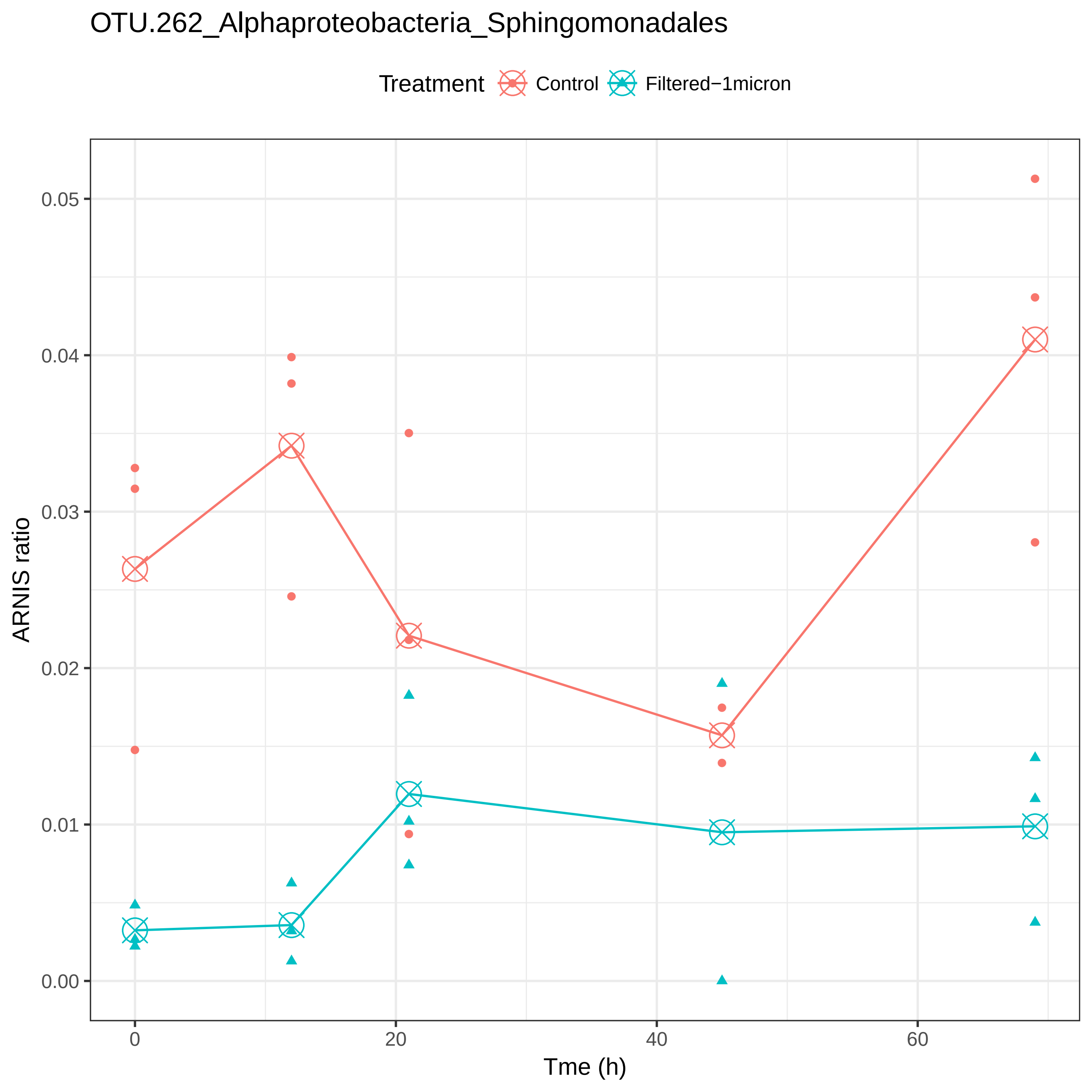
OTU.262_Alphaproteobacteria_Sphingomonadales
Treatment
Control
Filtered−1micron
ARNIS ratio
0.00
0.01
0.02
0.03
0.04
0.05
0
20
40
60
Tme (h)

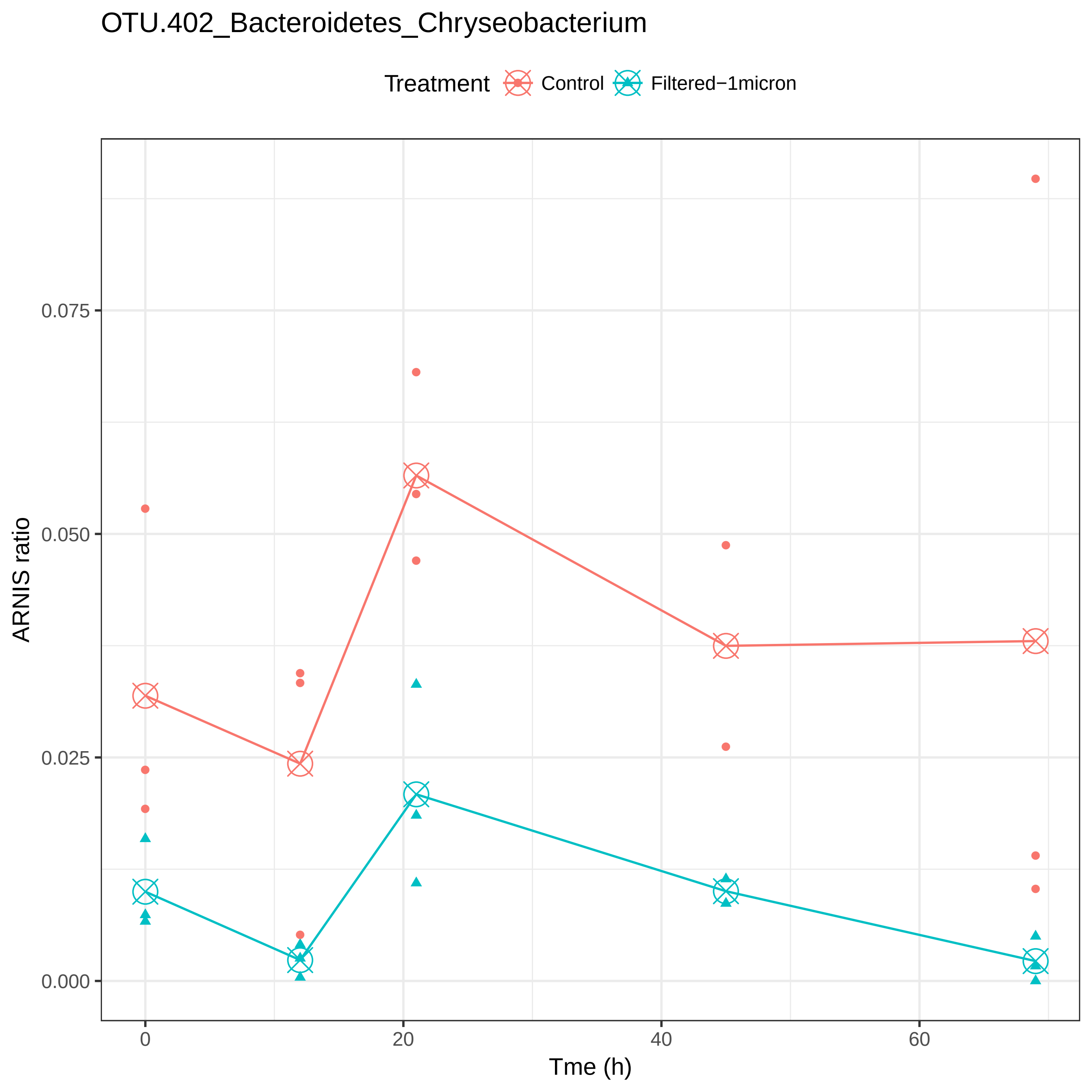
OTU.402_Bacteroidetes_Chryseobacterium
Treatment
Control
Filtered−1micron
ARNIS ratio
0.075
0.050
0.025
0.000
0
20
40
60
Tme (h)

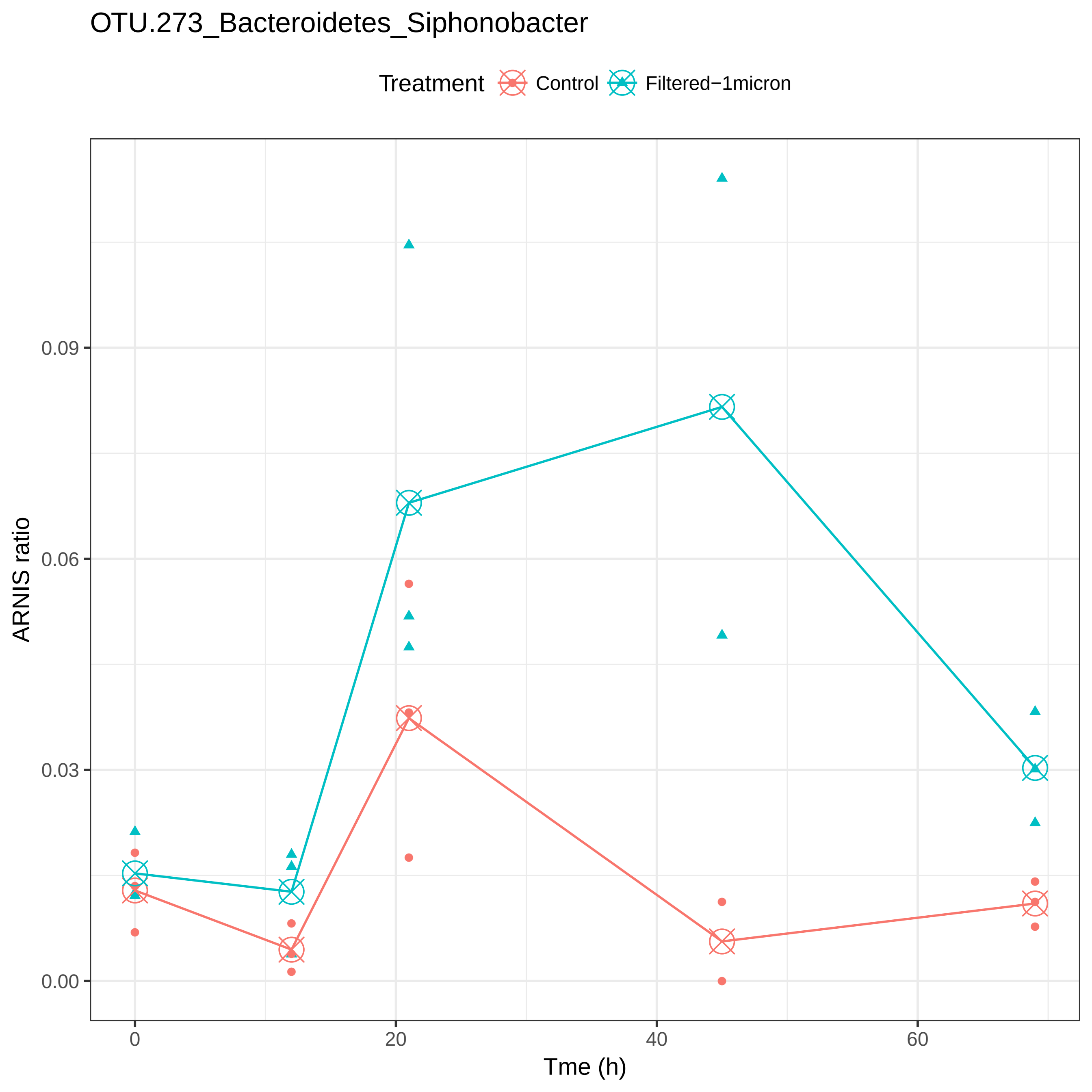

OTU.273_Bacteroidetes_Siphonobacter
Treatment
Control
Filtered−1micron
ARNIS ratio
0.00
0.03
0.06
0.09
0
20
40
60
Tme (h)

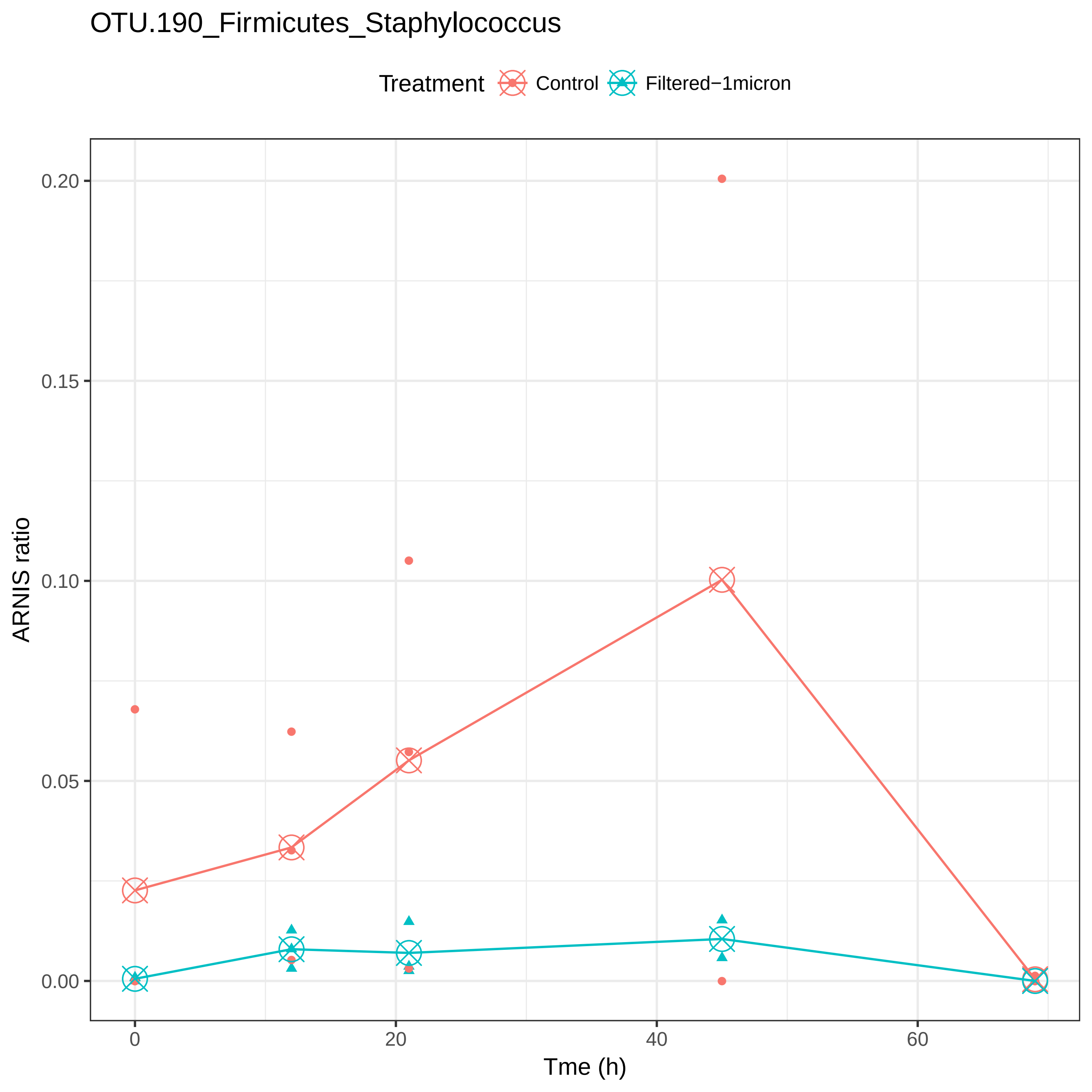
OTU.190_Firmicutes_Staphylococcus
Treatment
Control
Filtered−1micron
ARNIS ratio
0.20
0.15
0.10
0.05
0.00
0
20
40
60
Tme (h)

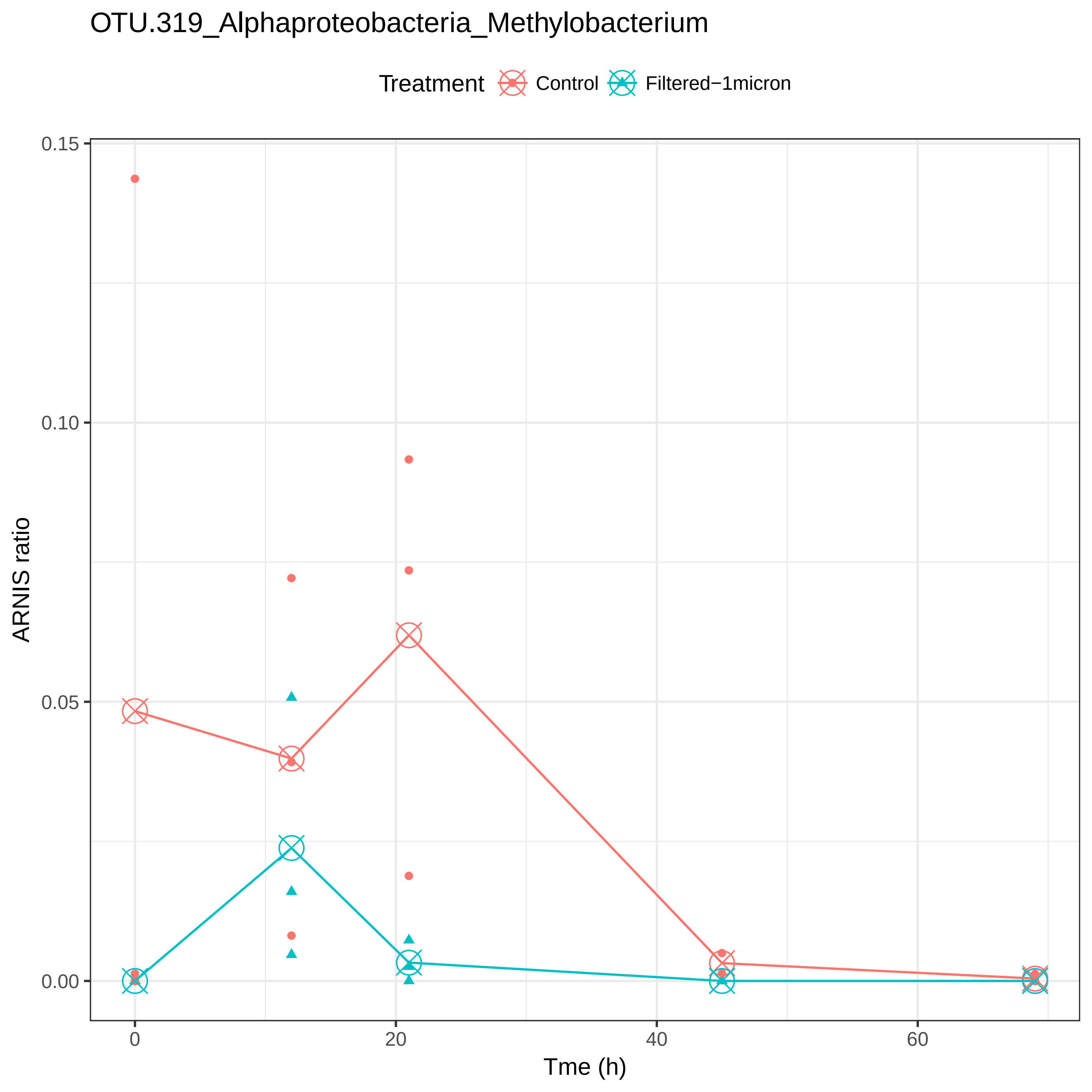
OTU.319_Alphaproteobacteria_Methylobacterium
Treatment
Control
Filtered−1micron
ARNIS ratio
0.15
0.10
0.05
0.00
0
20
40
60
Tme (h)

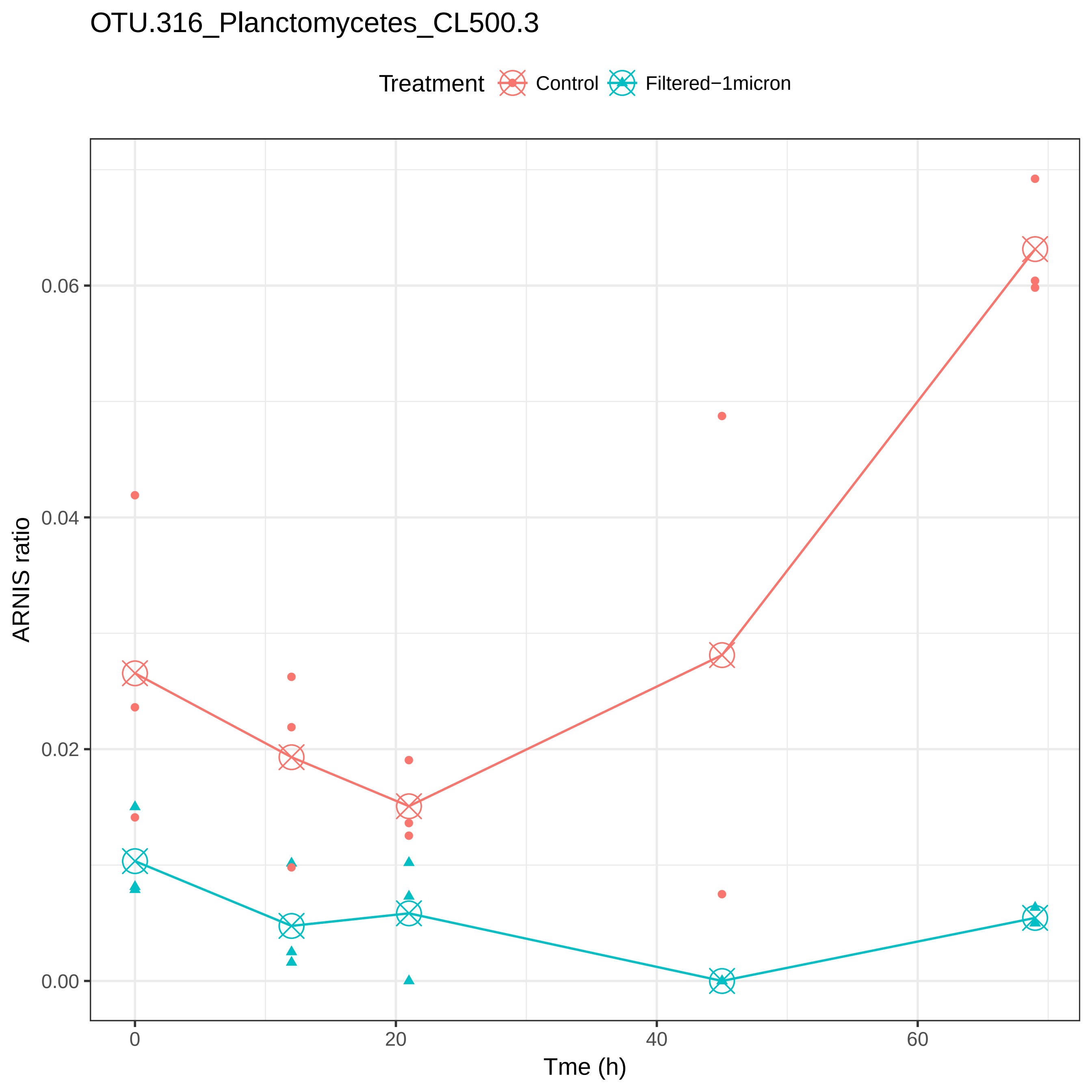
OTU.316_Planctomycetes_CL500.3
Treatment
Control
Filtered−1micron
ARNIS ratio
0.06
0.04
0.02
0.00
0
20
40
60
Tme (h)

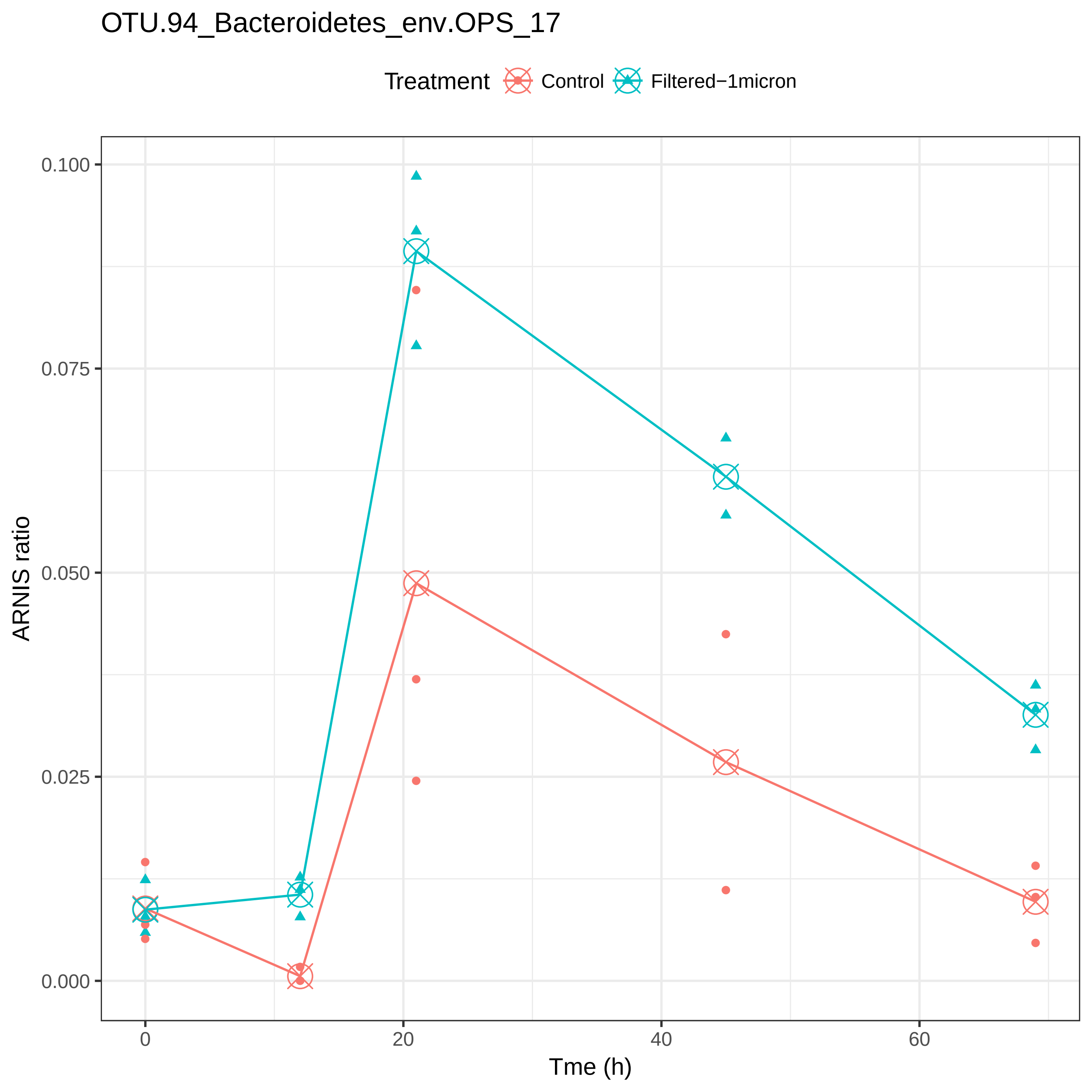
OTU.94_Bacteroidetes_env.OPS_17
Treatment
Control
Filtered−1micron
ARNIS ratio
0.100
0.075
0.050
0.025
0.000
0
20
40
60
Tme (h)

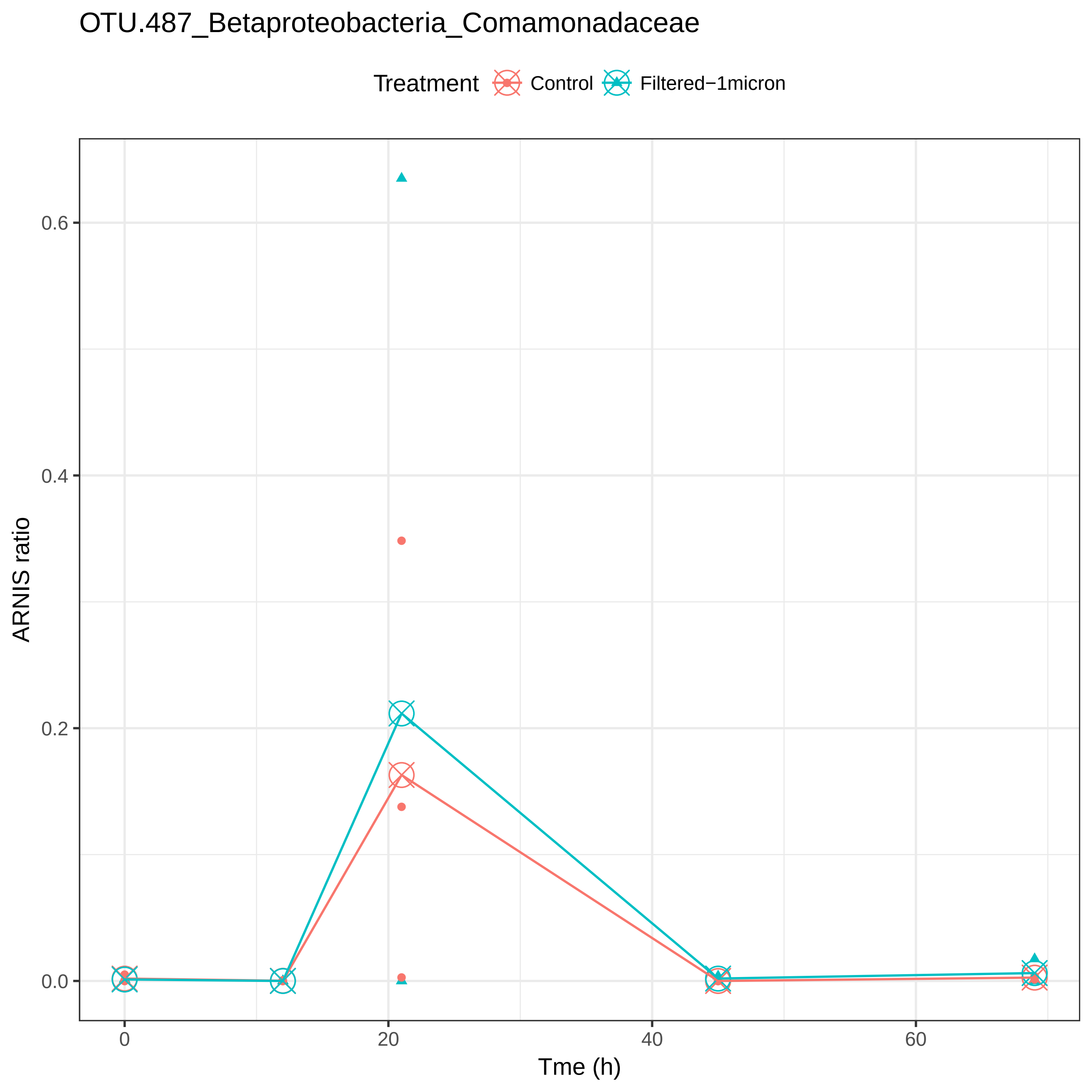
OTU.487_Betaproteobacteria_Comamonadaceae
Treatment
Control
Filtered−1micron
ARNIS ratio
0.0
0.2
0.4
0.6
0
20
40
60
Tme (h)

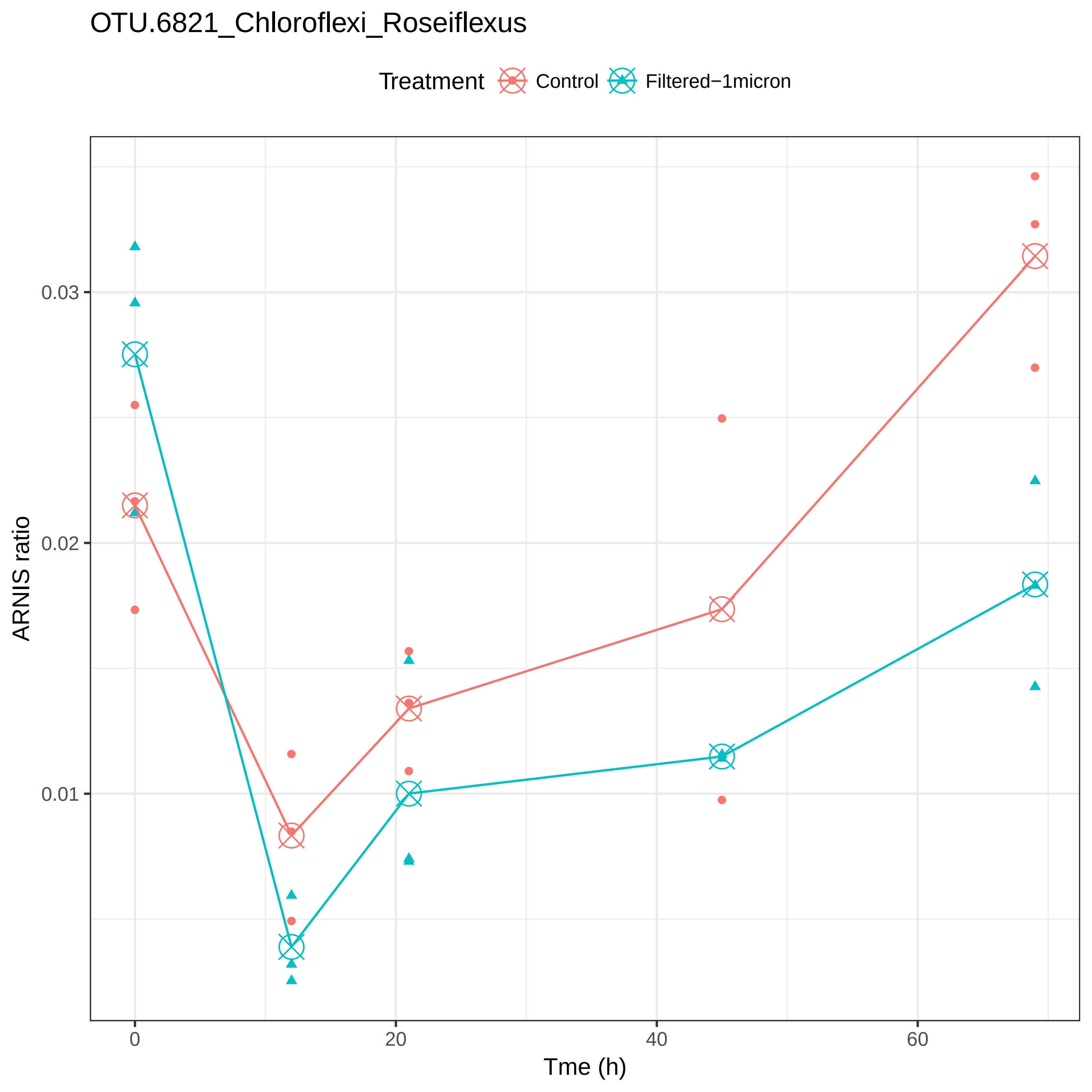

OTU.6821_Chloroflexi_Roseiflexus
Treatment
Control
Filtered−1micron
ARNIS ratio
0.03
0.02
0.01
0
20
40
60
Tme (h)

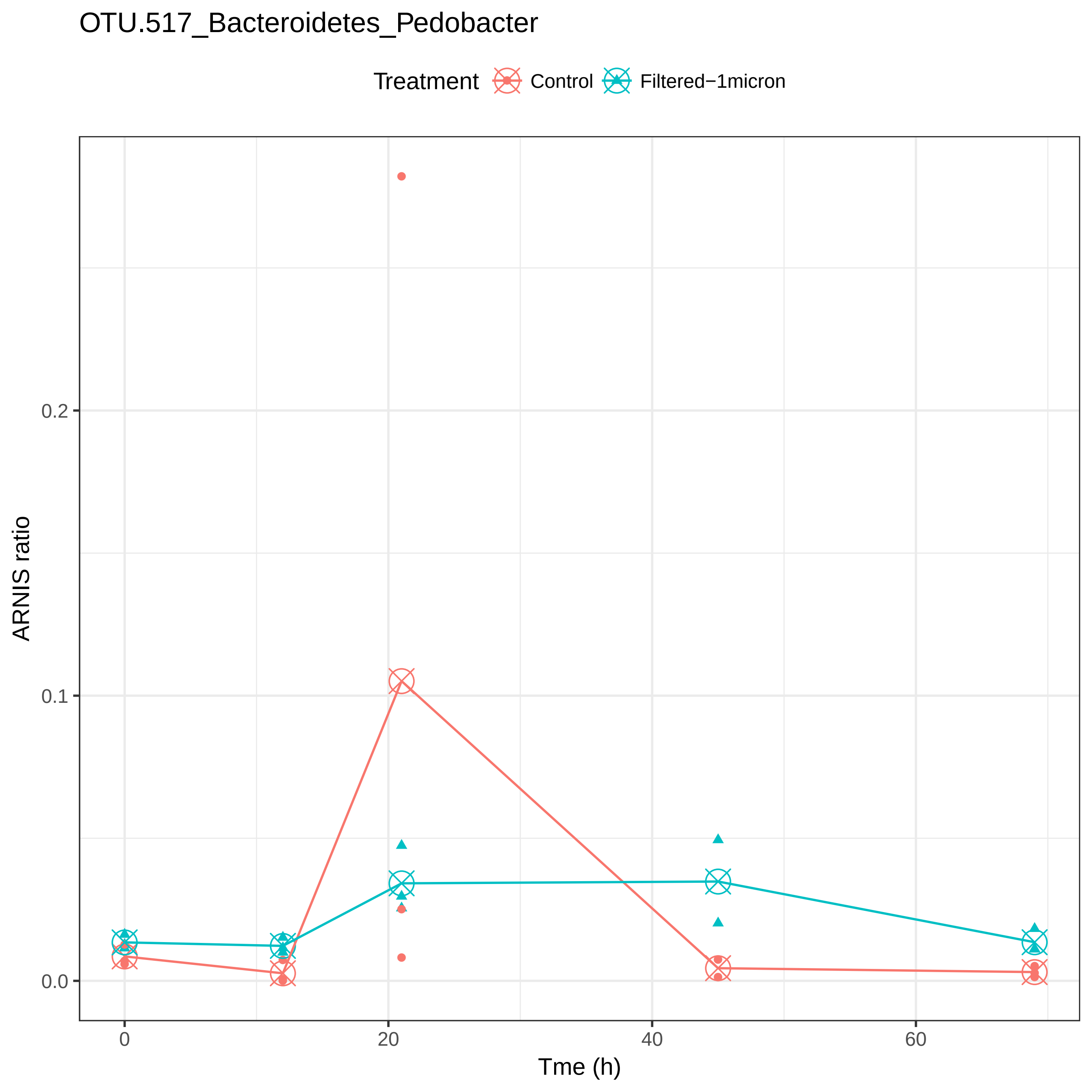

OTU.517_Bacteroidetes_Pedobacter
Treatment
Control
Filtered−1micron
ARNIS ratio
0.0
0.1
0.2
0
20
40
60
Tme (h)

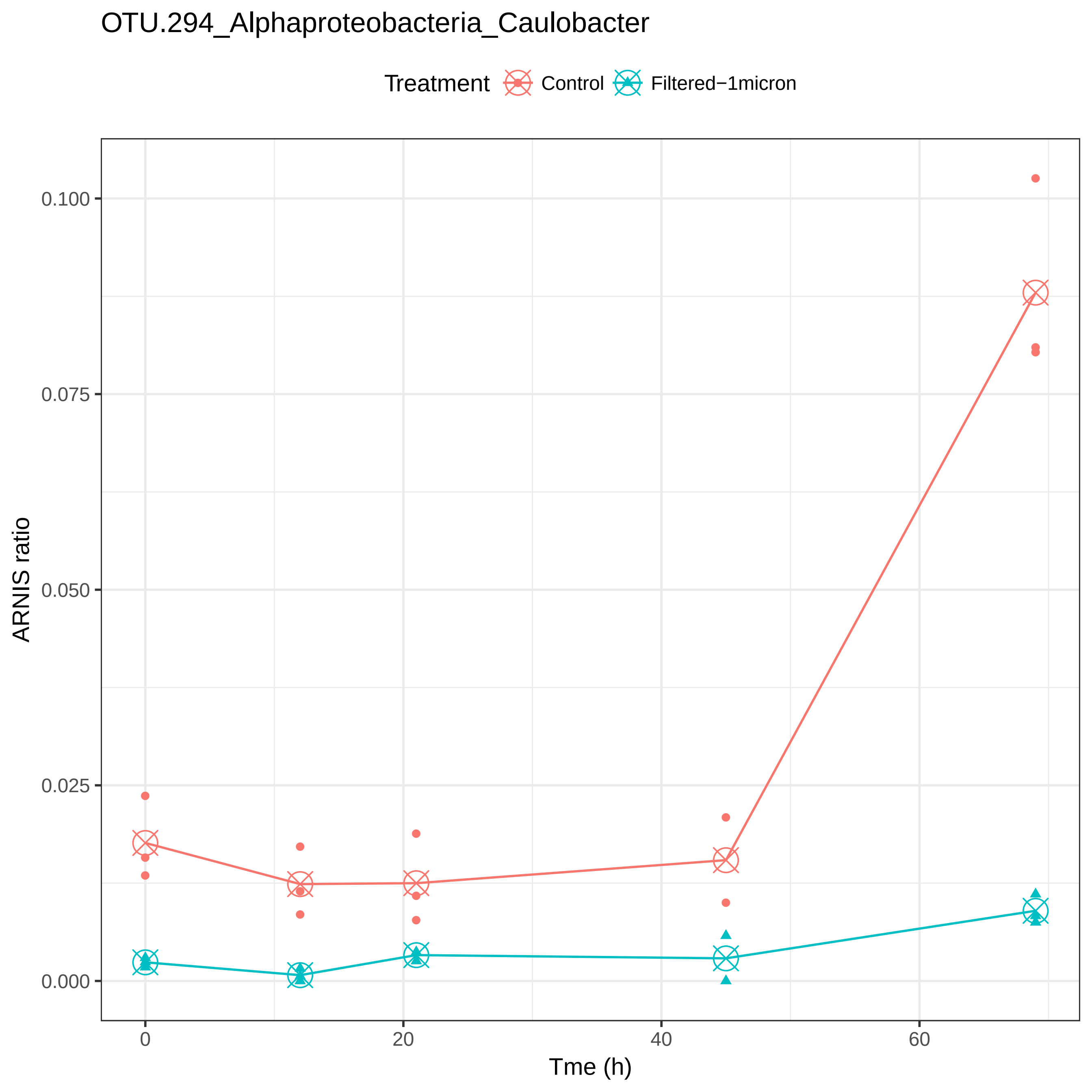

OTU.294_Alphaproteobacteria_Caulobacter
Treatment
Control
Filtered−1micron
ARNIS ratio
0.100
0.075
0.050
0.025
0.000
0
20
40
60
Tme (h)

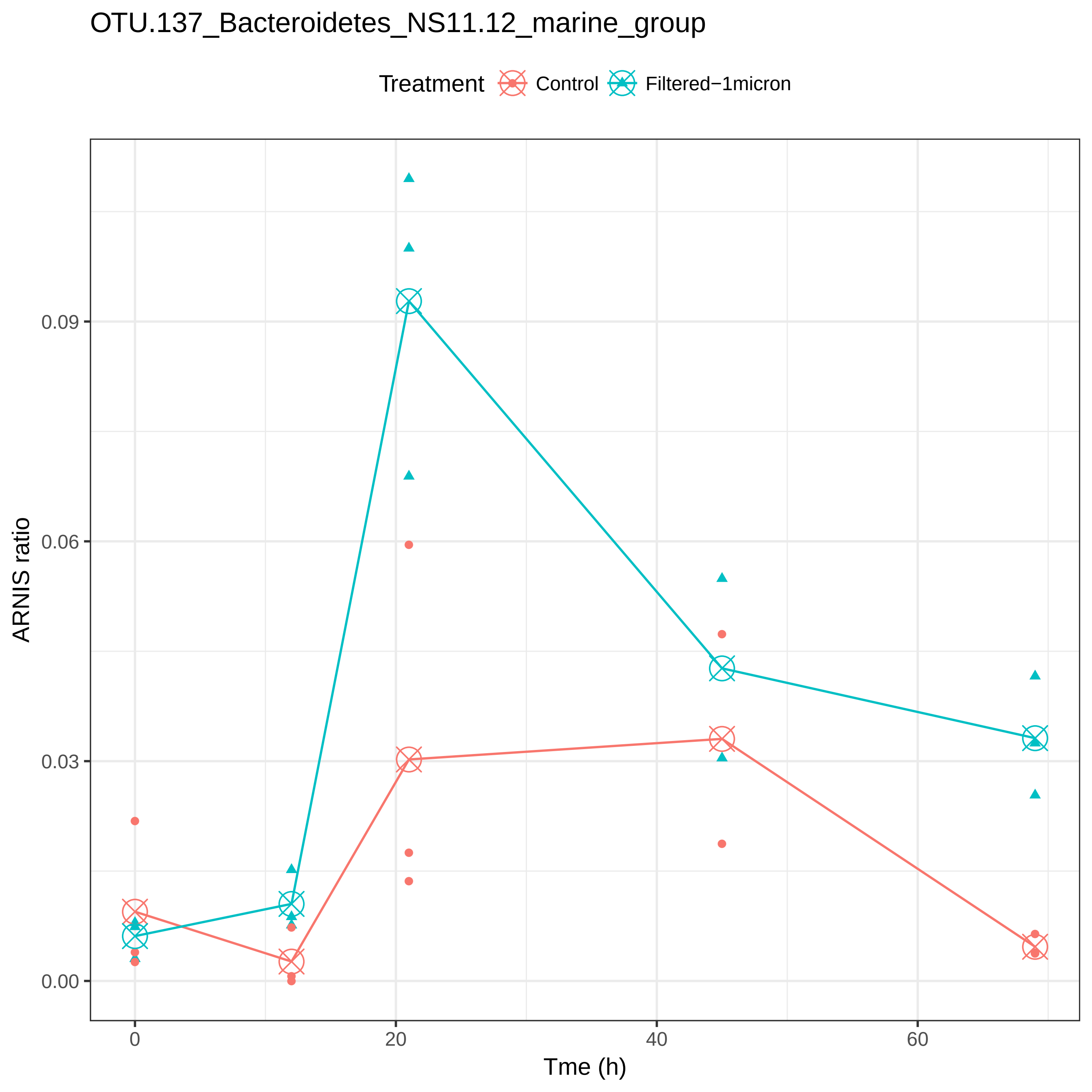

OTU.137_Bacteroidetes_NS11.12_marine_group
Treatment
Control
Filtered−1micron
ARNIS ratio
0.09
0.06
0.03
0.00
0
20
40
60
Tme (h)

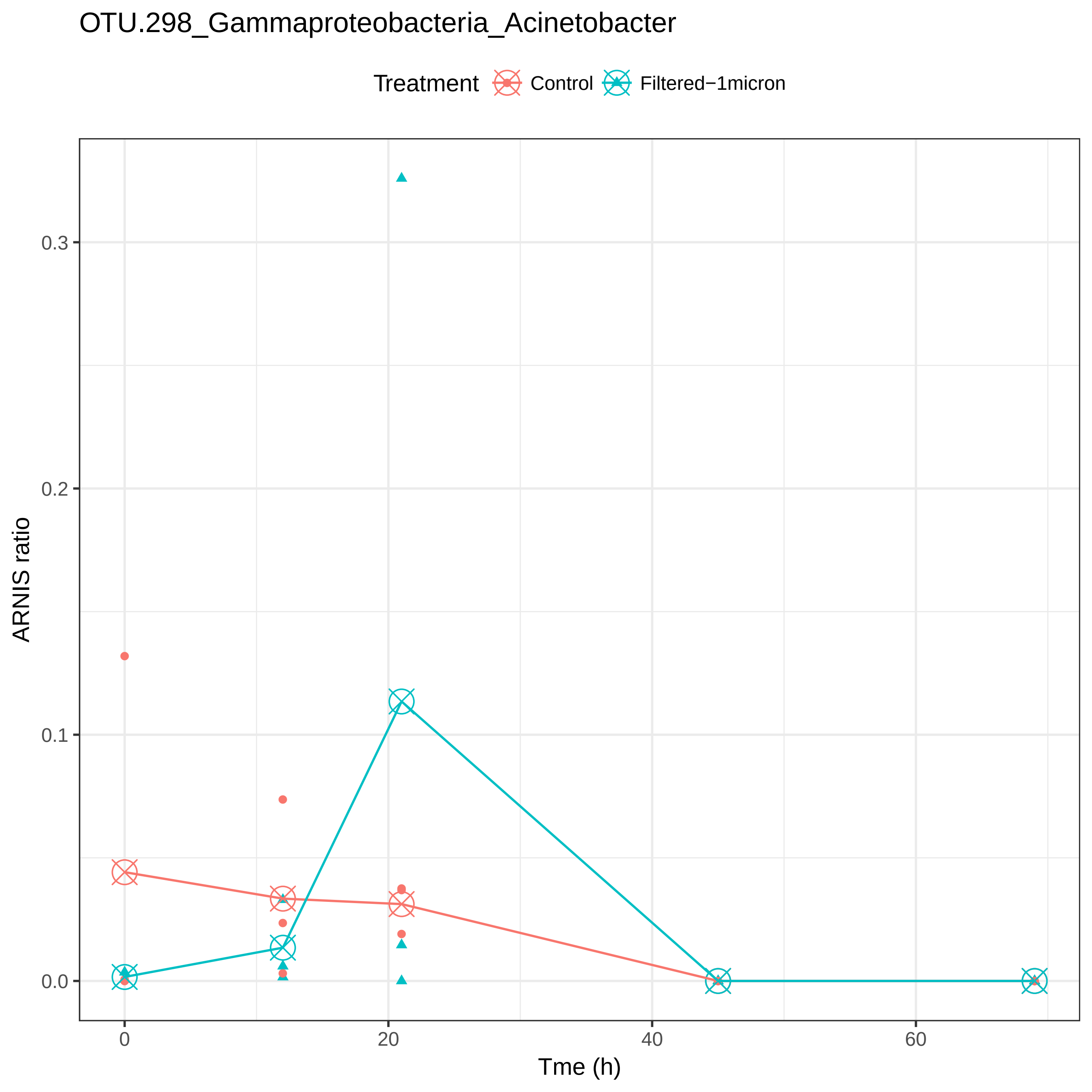
OTU.298_Gammaproteobacteria_Acinetobacter
Treatment
Control
Filtered−1micron
ARNIS ratio
0.0
0.1
0.2
0.3
0
20
40
60
Tme (h)

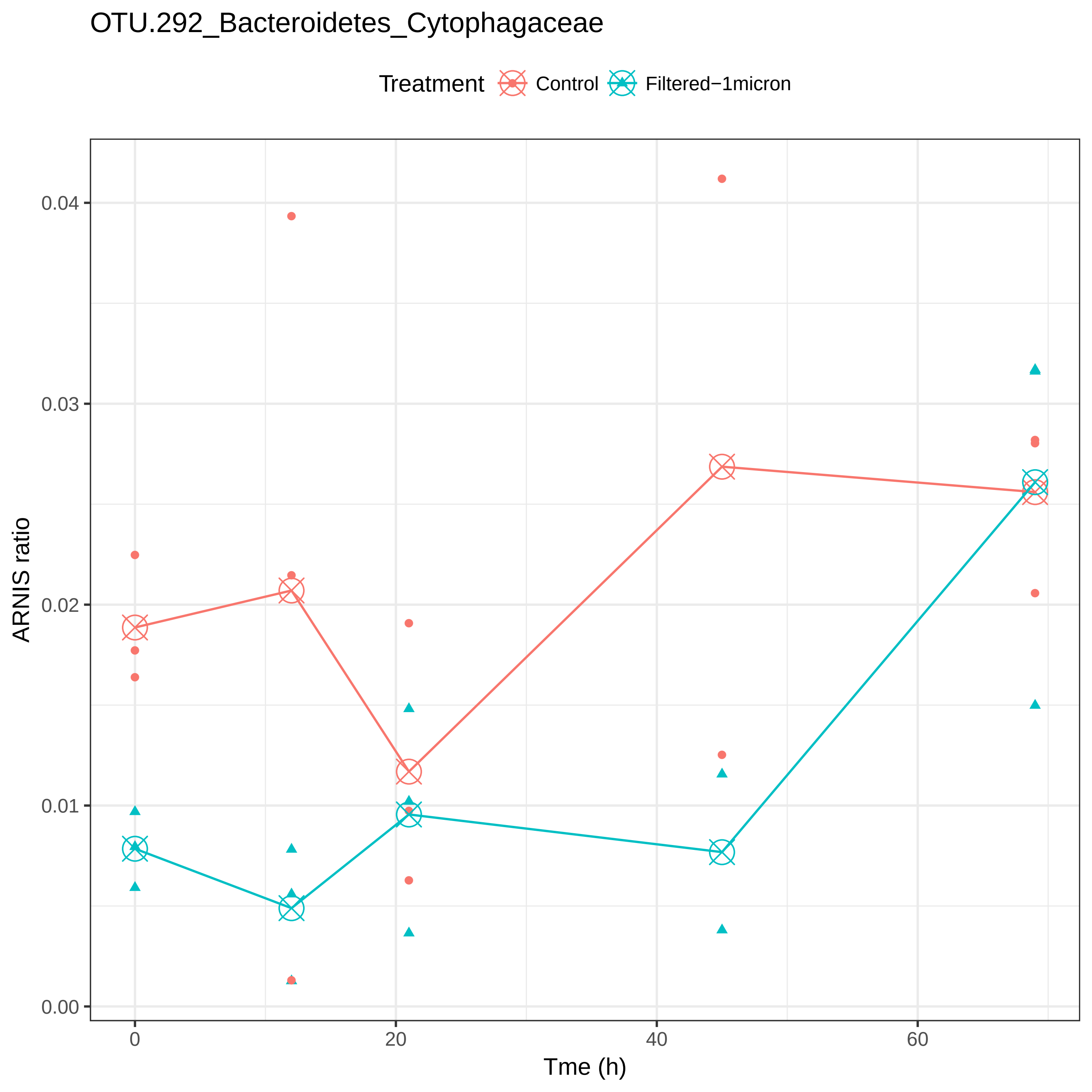

OTU.292_Bacteroidetes_Cytophagaceae
Treatment
Control
Filtered−1micron
ARNIS ratio
0.00
0.01
0.02
0.03
0.04
0
20
40
60
Tme (h)

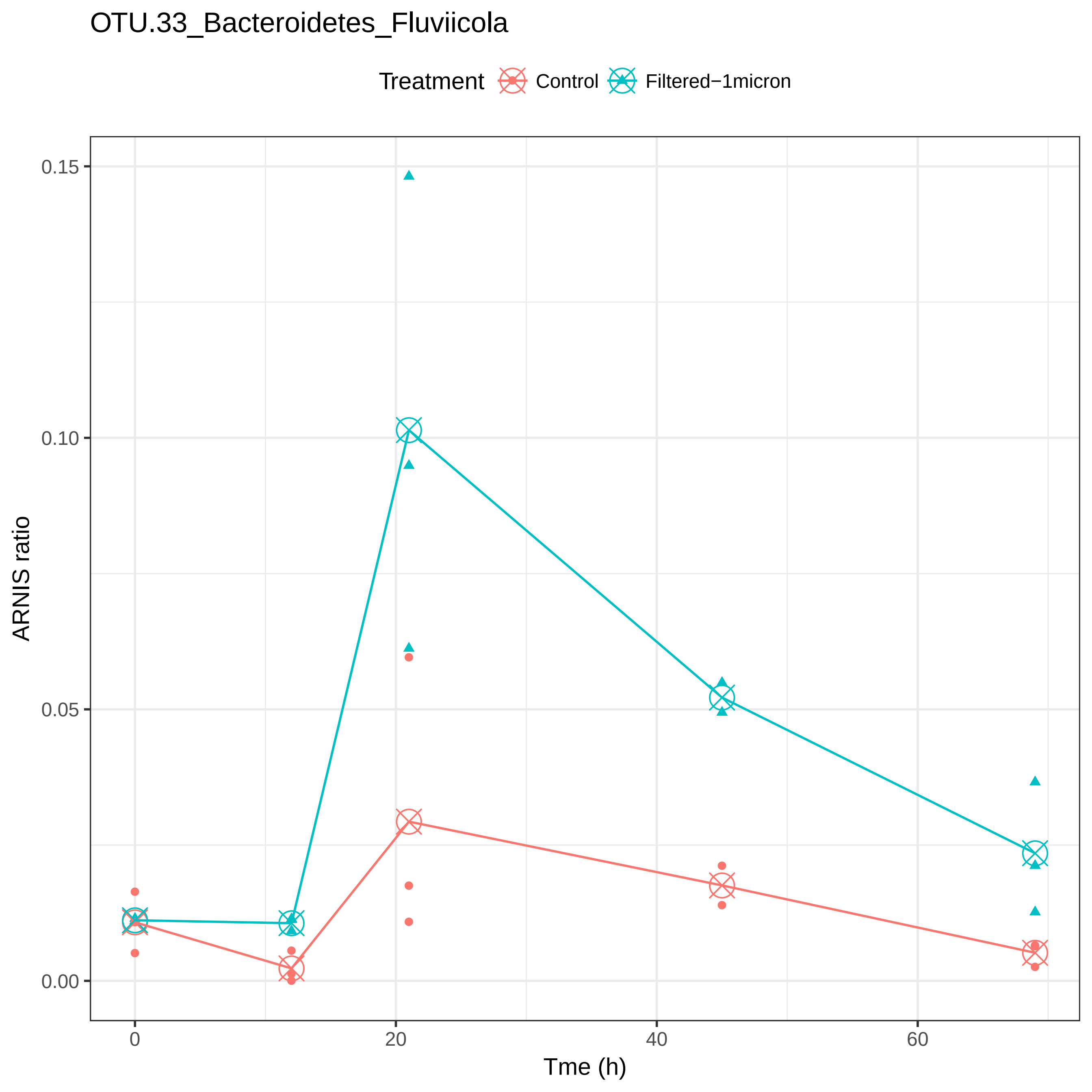

OTU.33_Bacteroidetes_Fluviicola
Treatment
Control
Filtered−1micron
ARNIS ratio
0.15
0.10
0.05
0.00
0
20
40
60
Tme (h)

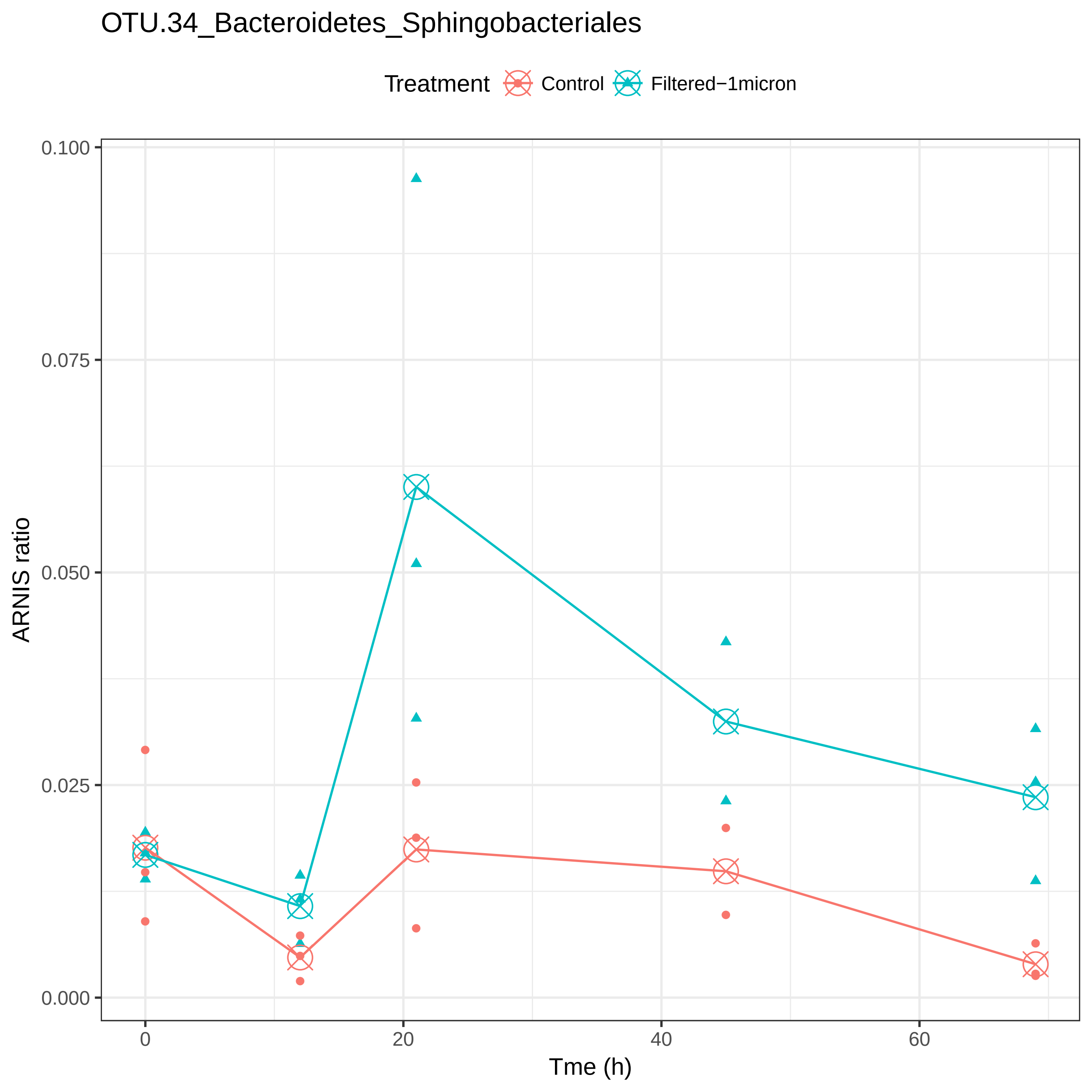

OTU.34_Bacteroidetes_Sphingobacteriales
Treatment
Control
Filtered−1micron
ARNIS ratio
0.100
0.075
0.050
0.025
0.000
0
20
40
60
Tme (h)

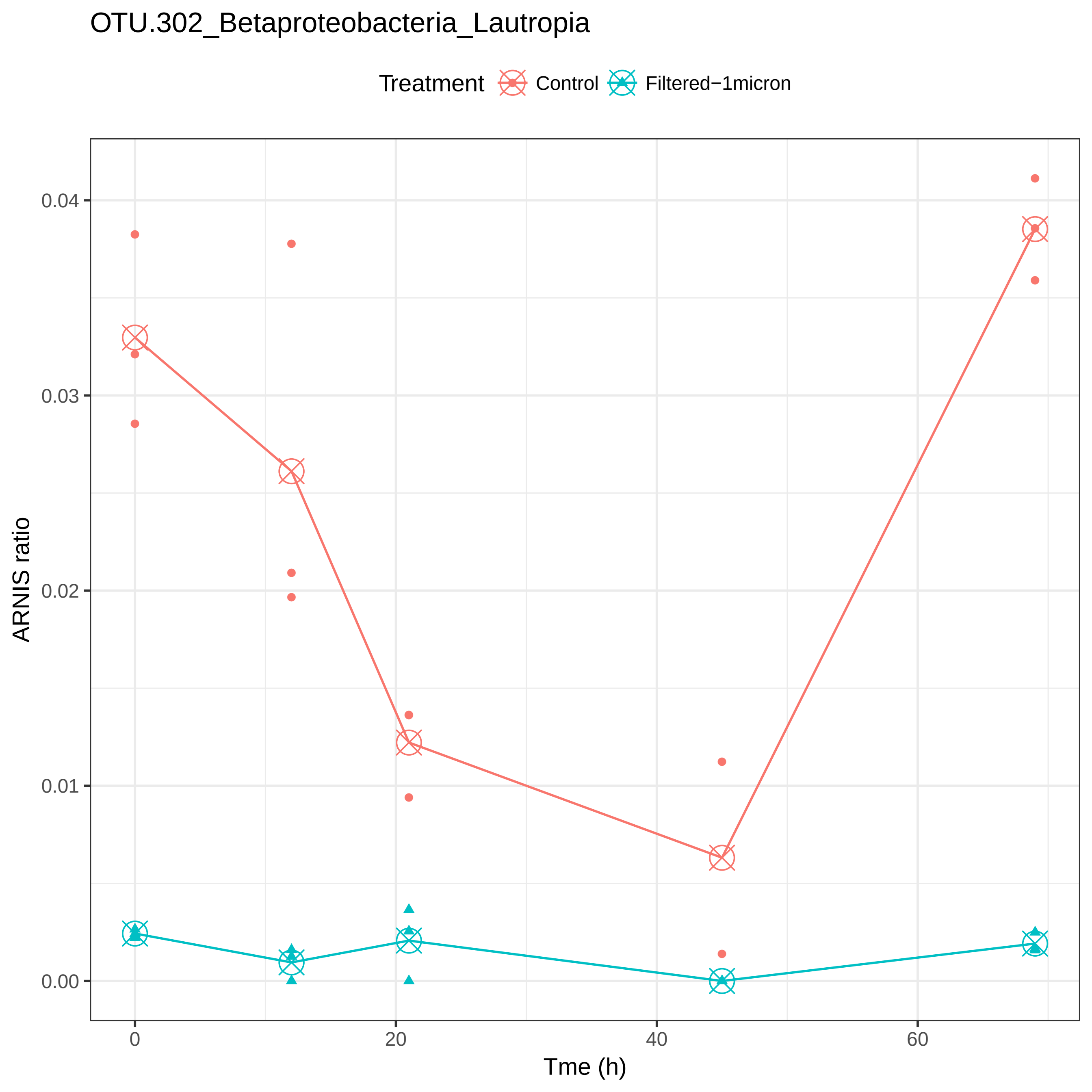
OTU.302_Betaproteobacteria_Lautropia
Treatment
Control
Filtered−1micron
ARNIS ratio
0.04
0.03
0.02
0.01
0.00
0
20
40
60
Tme (h)

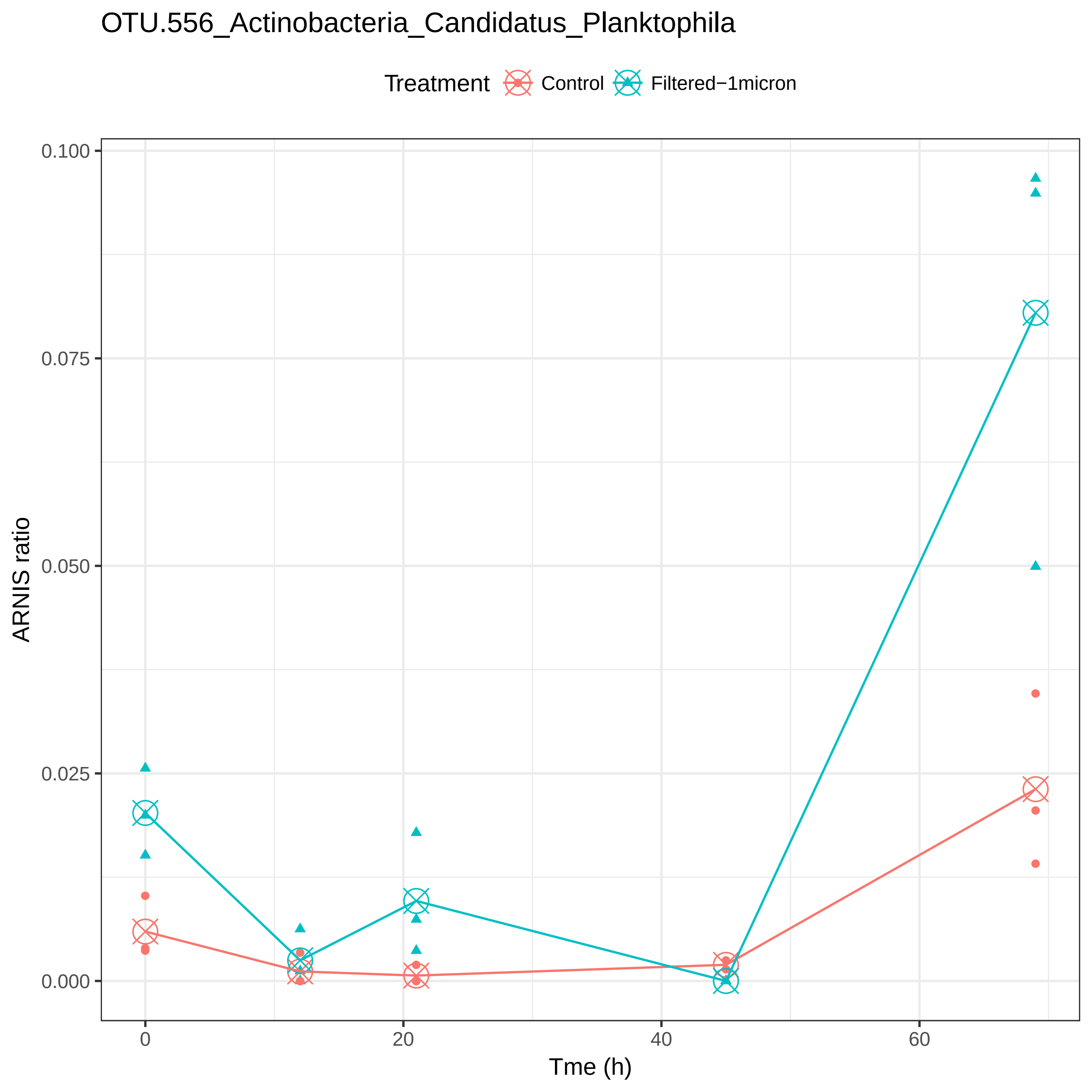

OTU.556_Actinobacteria_Candidatus_Planktophila
Treatment
Control
Filtered−1micron
ARNIS ratio
0.100
0.075
0.050
0.025
0.000
0
20
40
60
Tme (h)

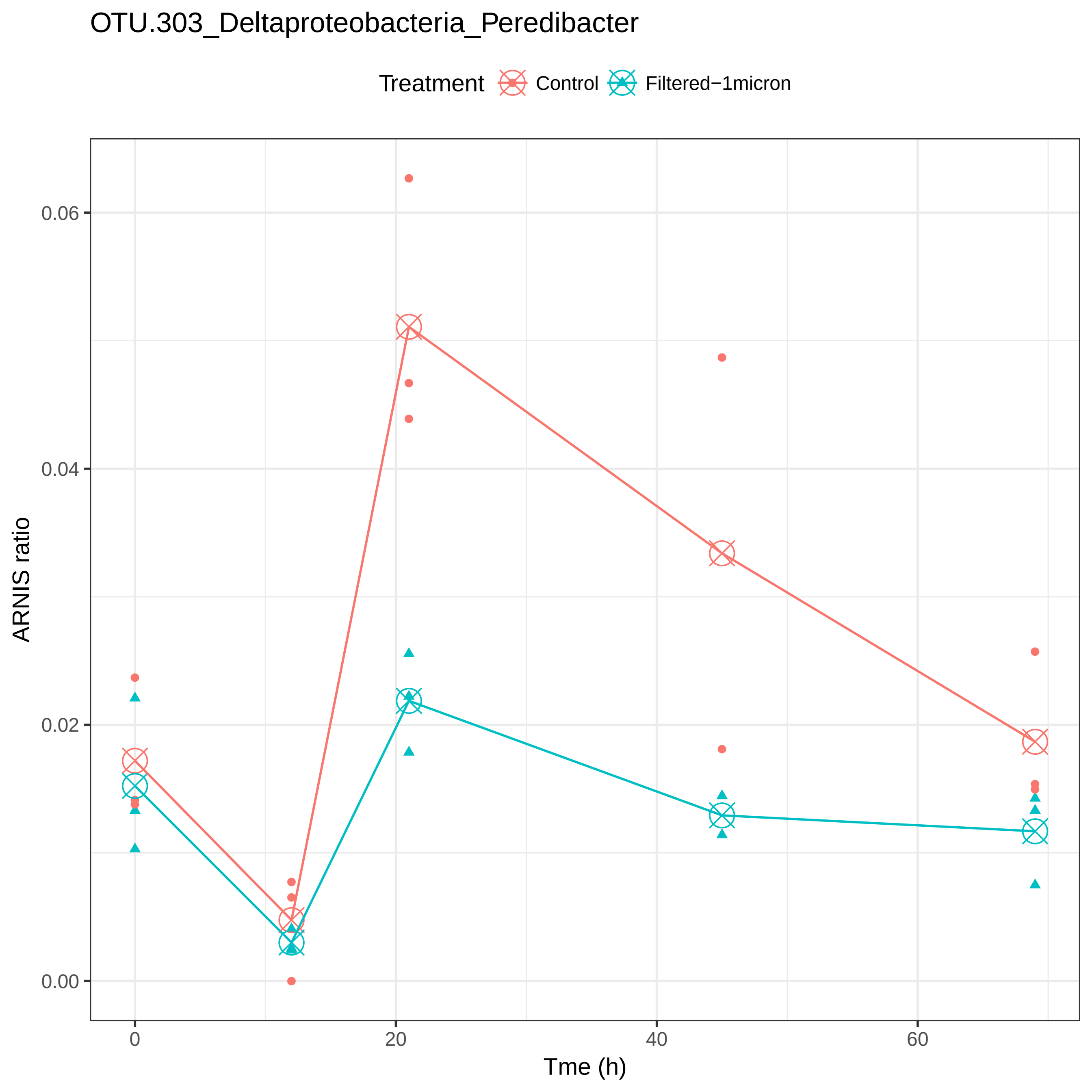
OTU.303_Deltaproteobacteria_Peredibacter
Treatment
Control
Filtered−1micron
ARNIS ratio
0.00
0.02
0.04
0.06
0
20
40
60
Tme (h)

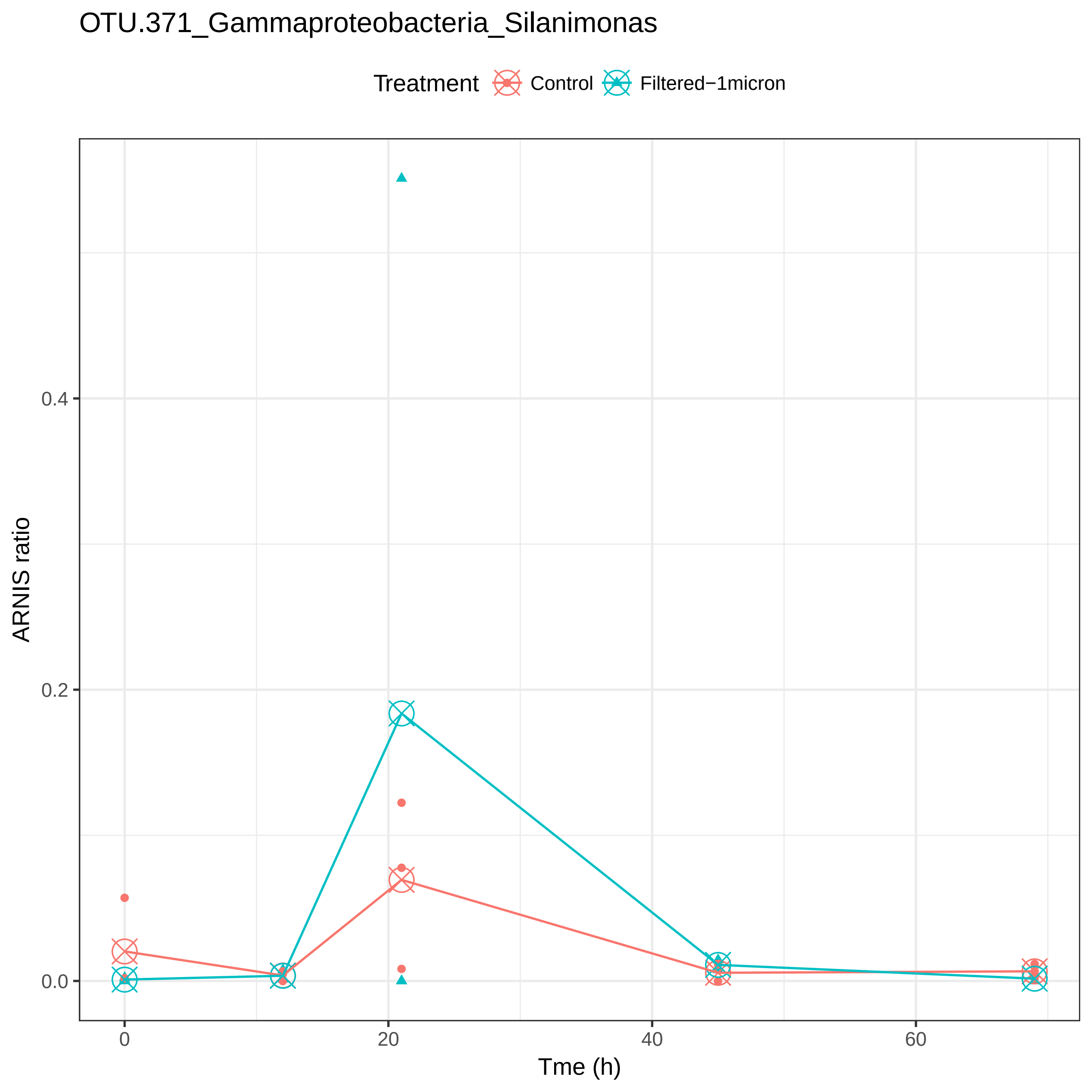

OTU.371_Gammaproteobacteria_Silanimonas
Treatment
Control
Filtered−1micron
ARNIS ratio
0.4
0.2
0.0
0
20
40
60
Tme (h)

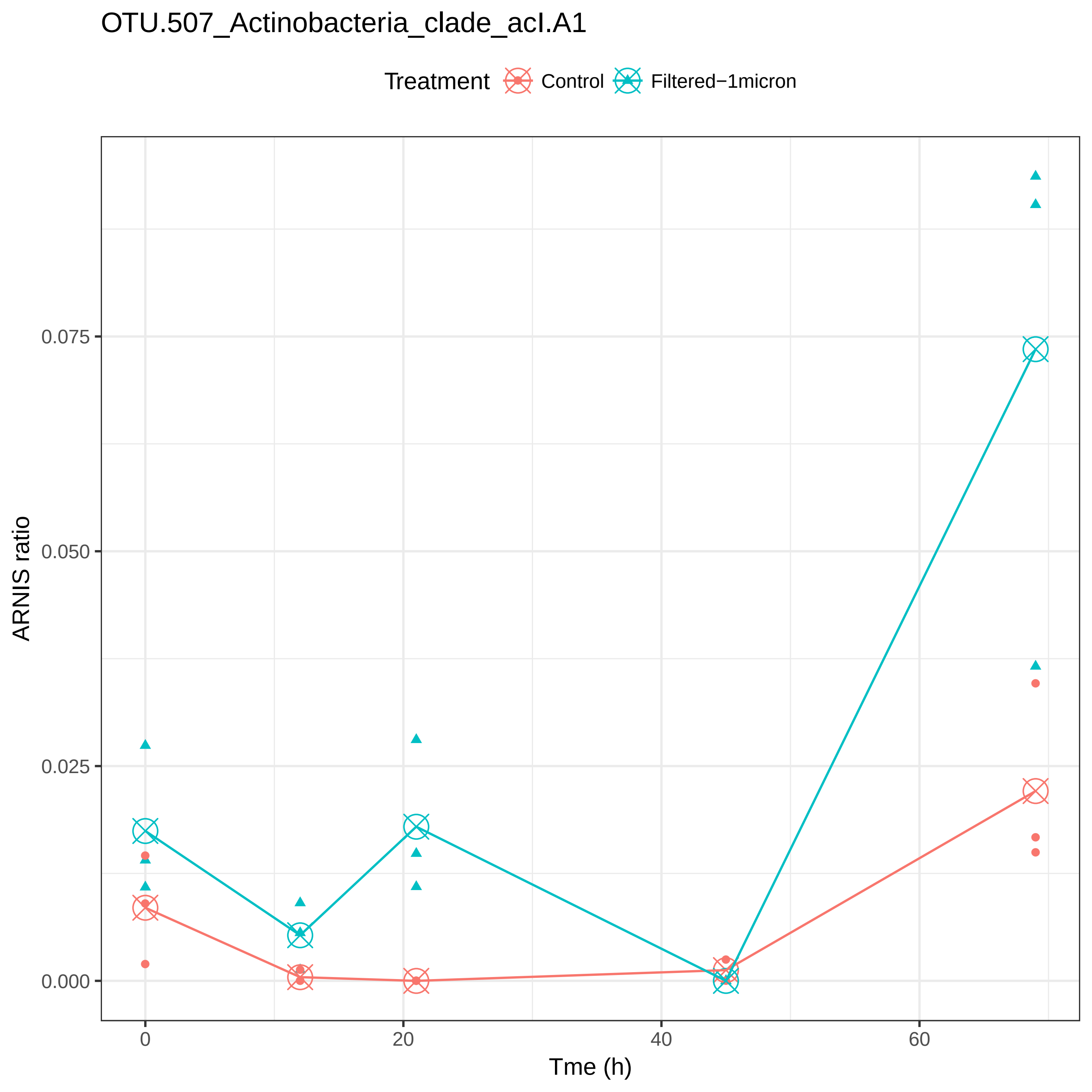

OTU.507_Actinobacteria_clade_acI.A1
Treatment
Control
Filtered−1micron
ARNIS ratio
0.000
0.025
0.050
0.075
0
20
40
60
Tme (h)

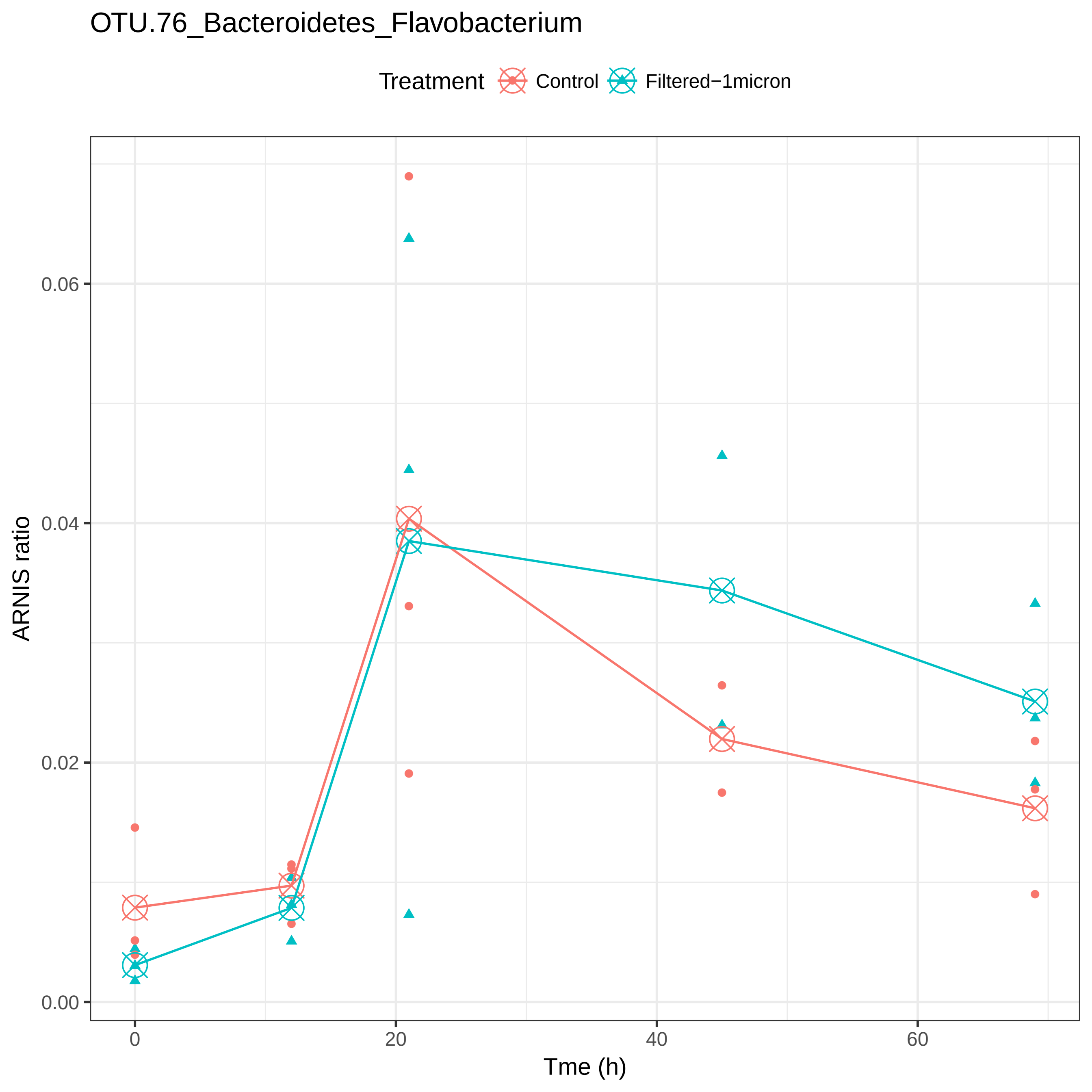
OTU.76_Bacteroidetes_Flavobacterium
Treatment
Control
Filtered−1micron
ARNIS ratio
0.00
0.02
0.04
0.06
0
20
40
60
Tme (h)

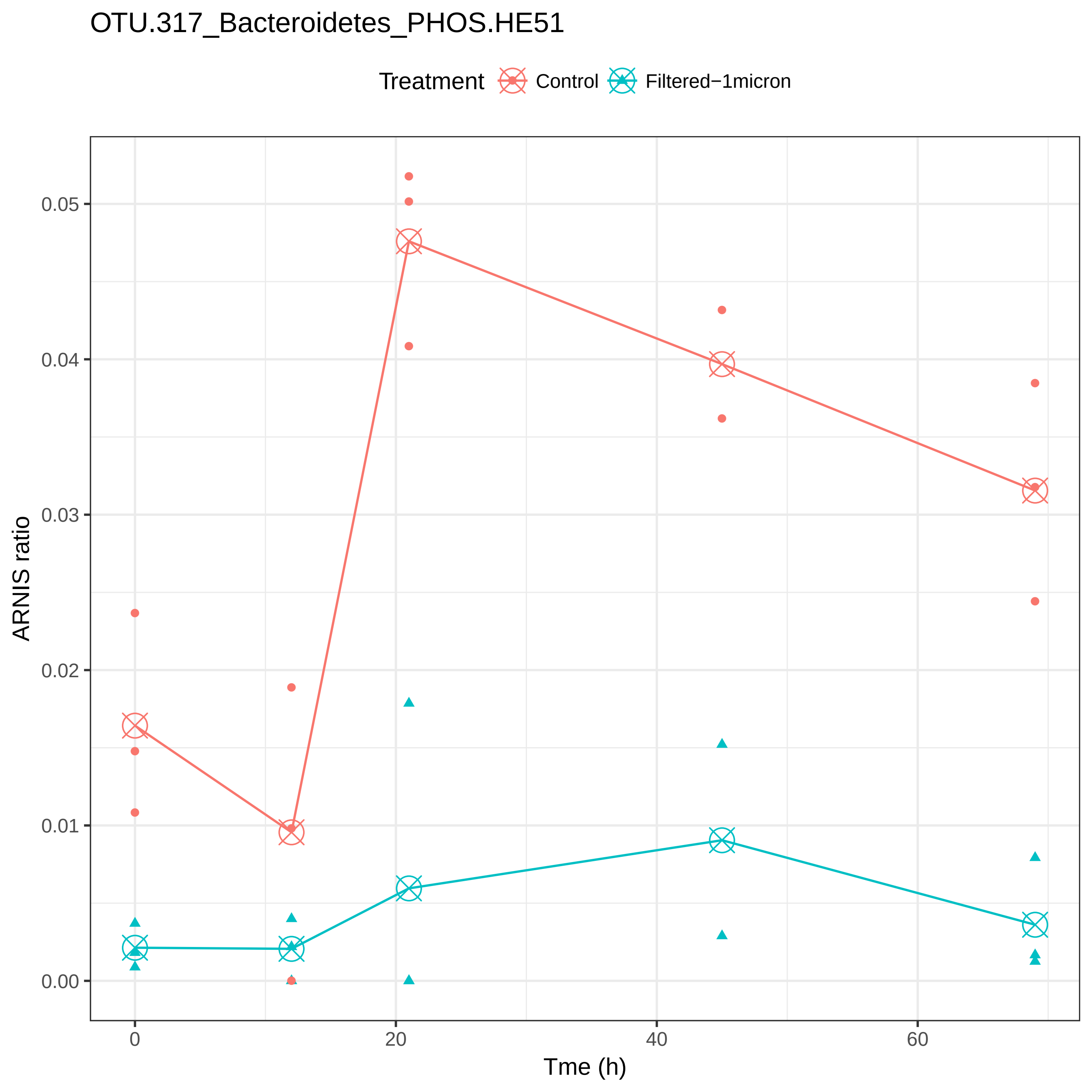

OTU.317_Bacteroidetes_PHOS.HE51
Treatment
Control
Filtered−1micron
ARNIS ratio
0.00
0.01
0.02
0.03
0.04
0.05
0
20
40
60
Tme (h)

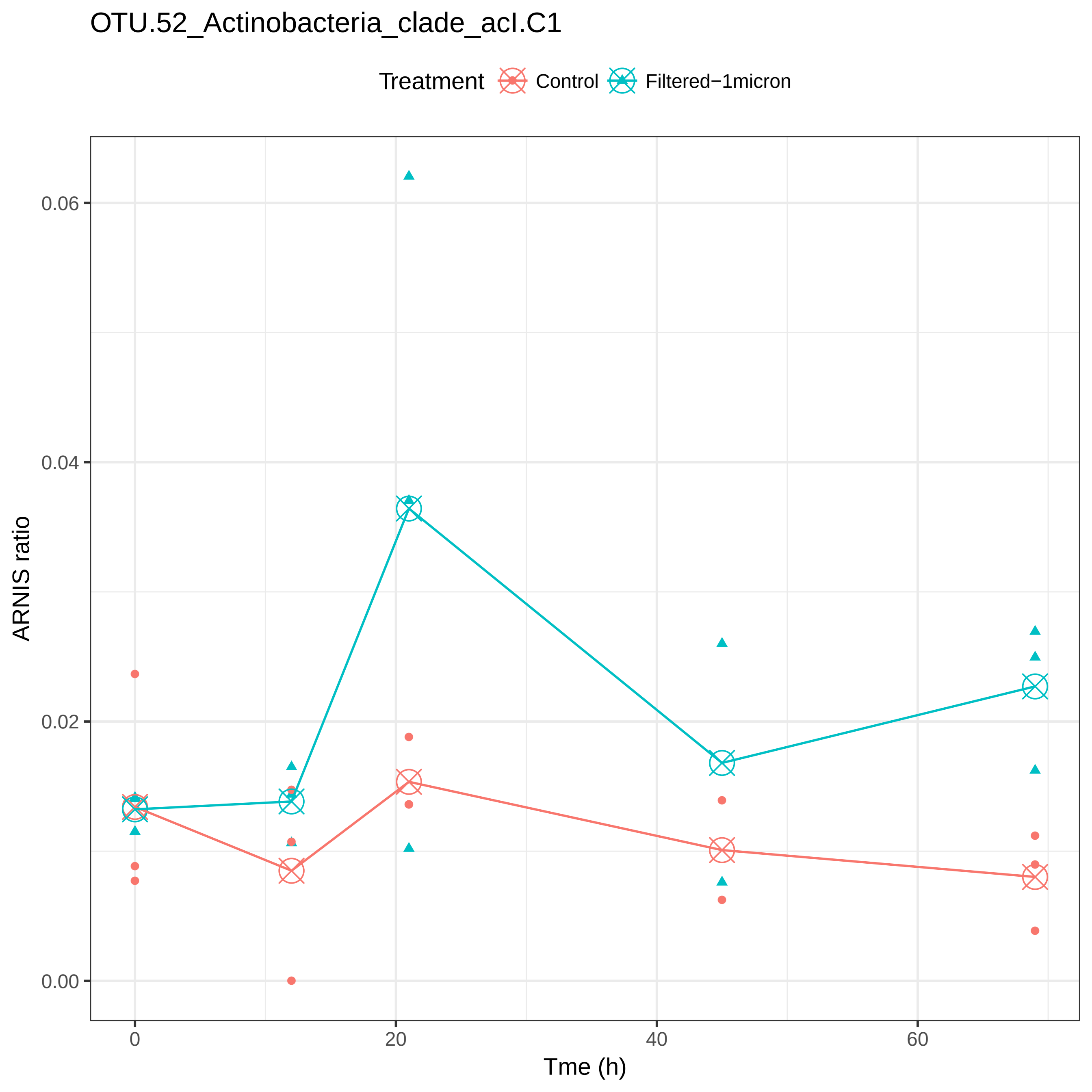

OTU.52_Actinobacteria_clade_acI.C1
Treatment
Control
Filtered−1micron
0.06
0.04
0.02
0.00
ARNIS ratio
0
20
40
60
Tme (h)

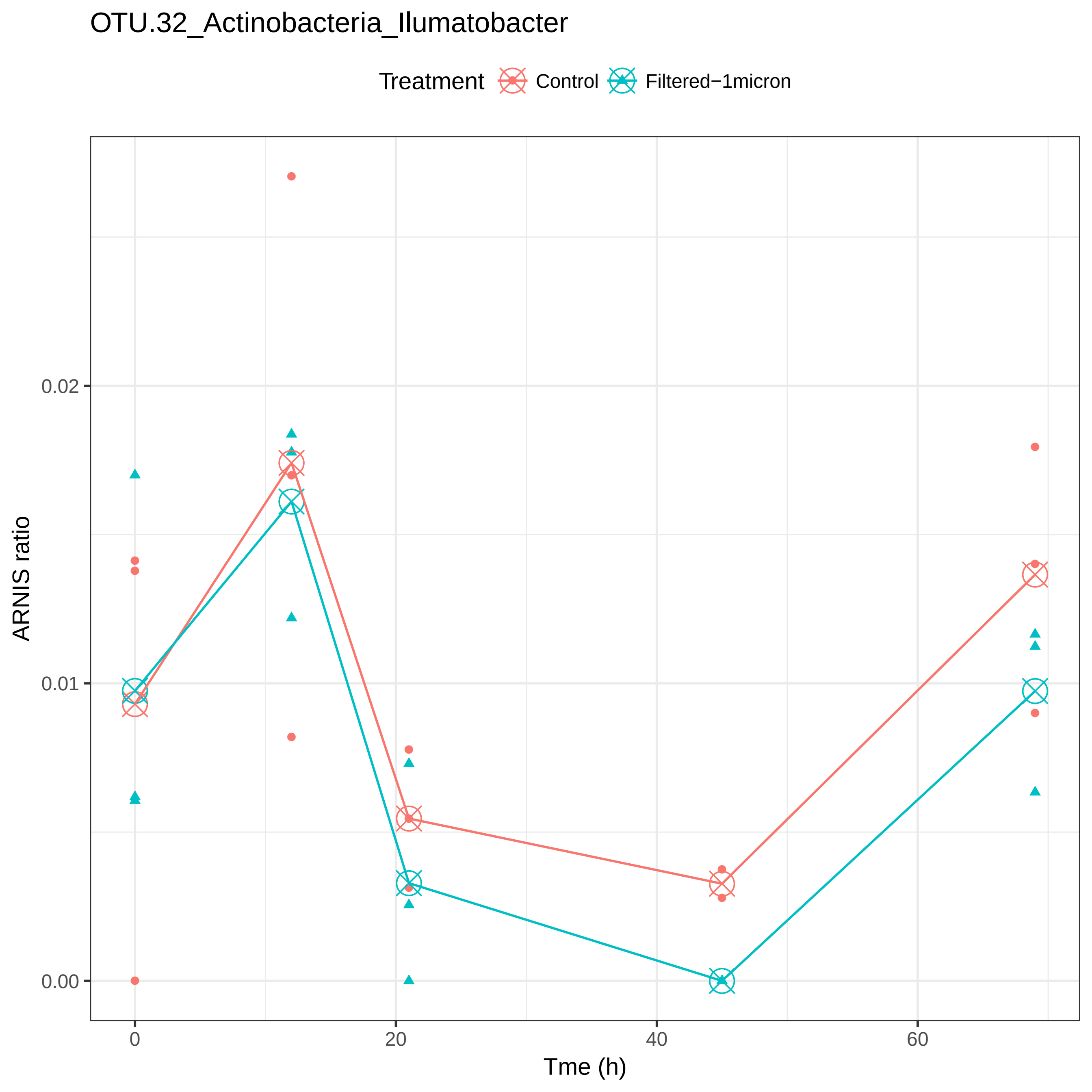
OTU.32_Actinobacteria_Ilumatobacter
Treatment
Control
Filtered−1micron
ARNIS ratio
0.00
0.01
0.02
0
20
40
60
Tme (h)

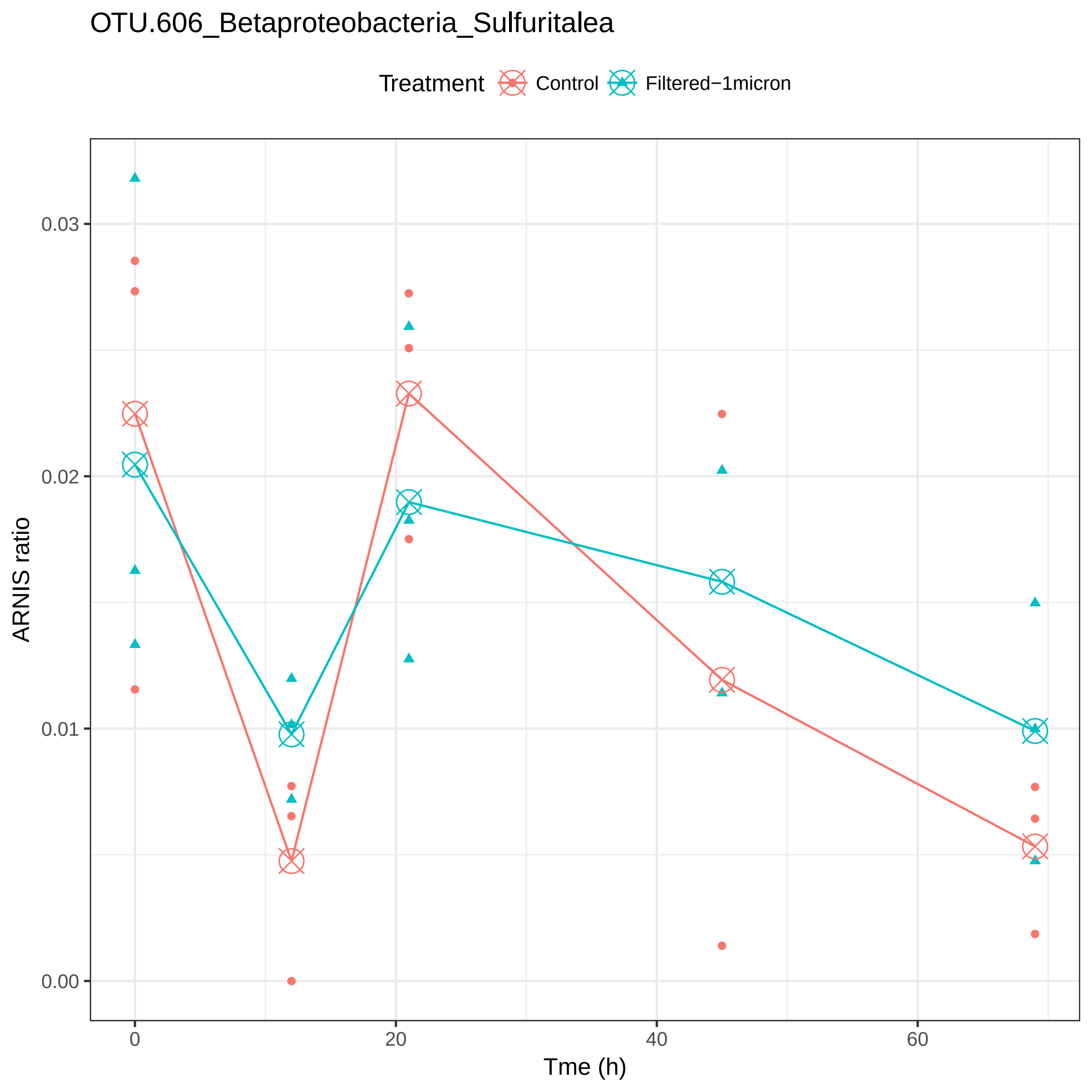

OTU.606_Betaproteobacteria_Sulfuritalea
Treatment
Control
Filtered−1micron
ARNIS ratio
0.03
0.02
0.01
0.00
0
20
40
60
Tme (h)

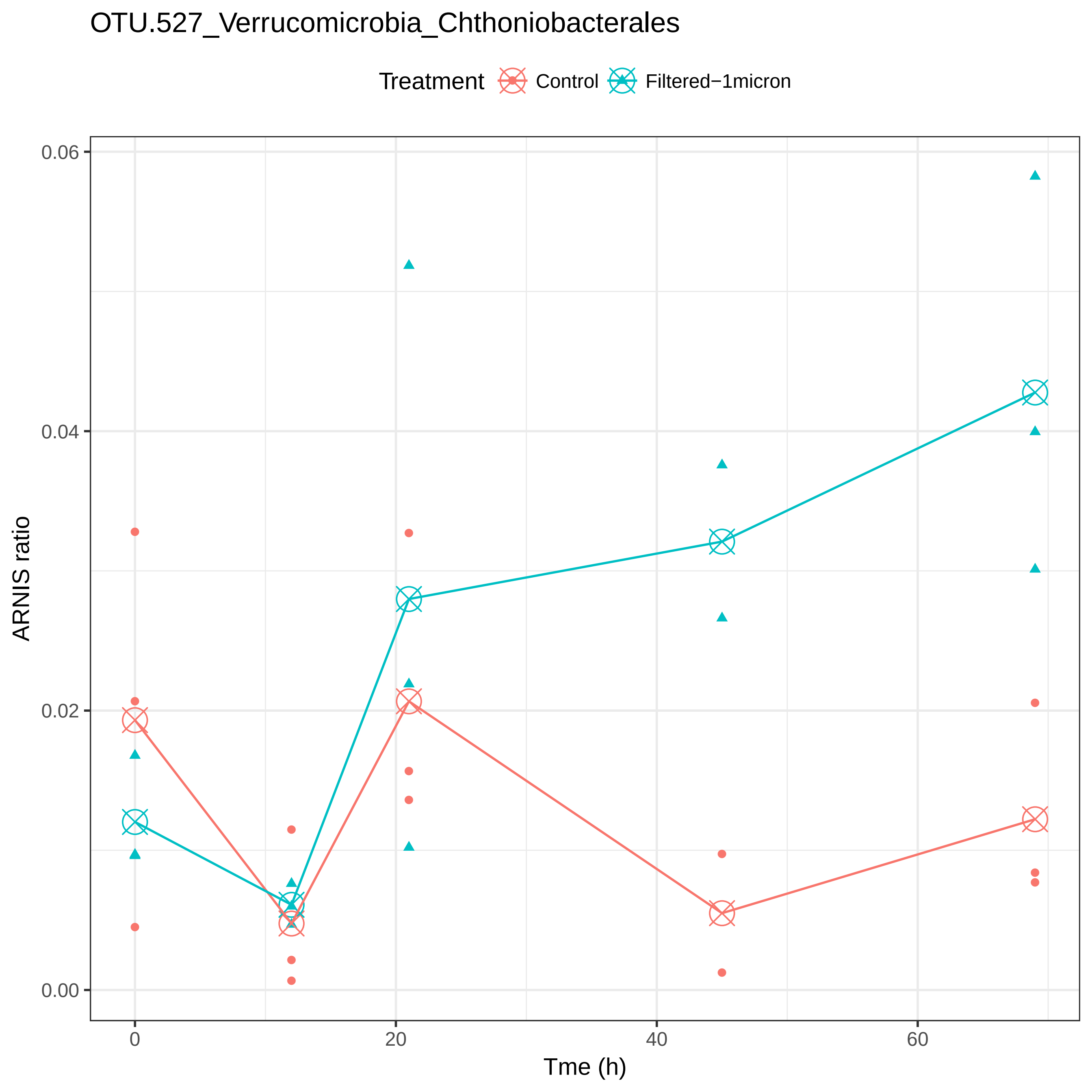

OTU.527_Verrucomicrobia_Chthoniobacterales
Treatment
Control
Filtered−1micron
ARNIS ratio
0.06
0.04
0.02
0.00
0
20
40
60
Tme (h)

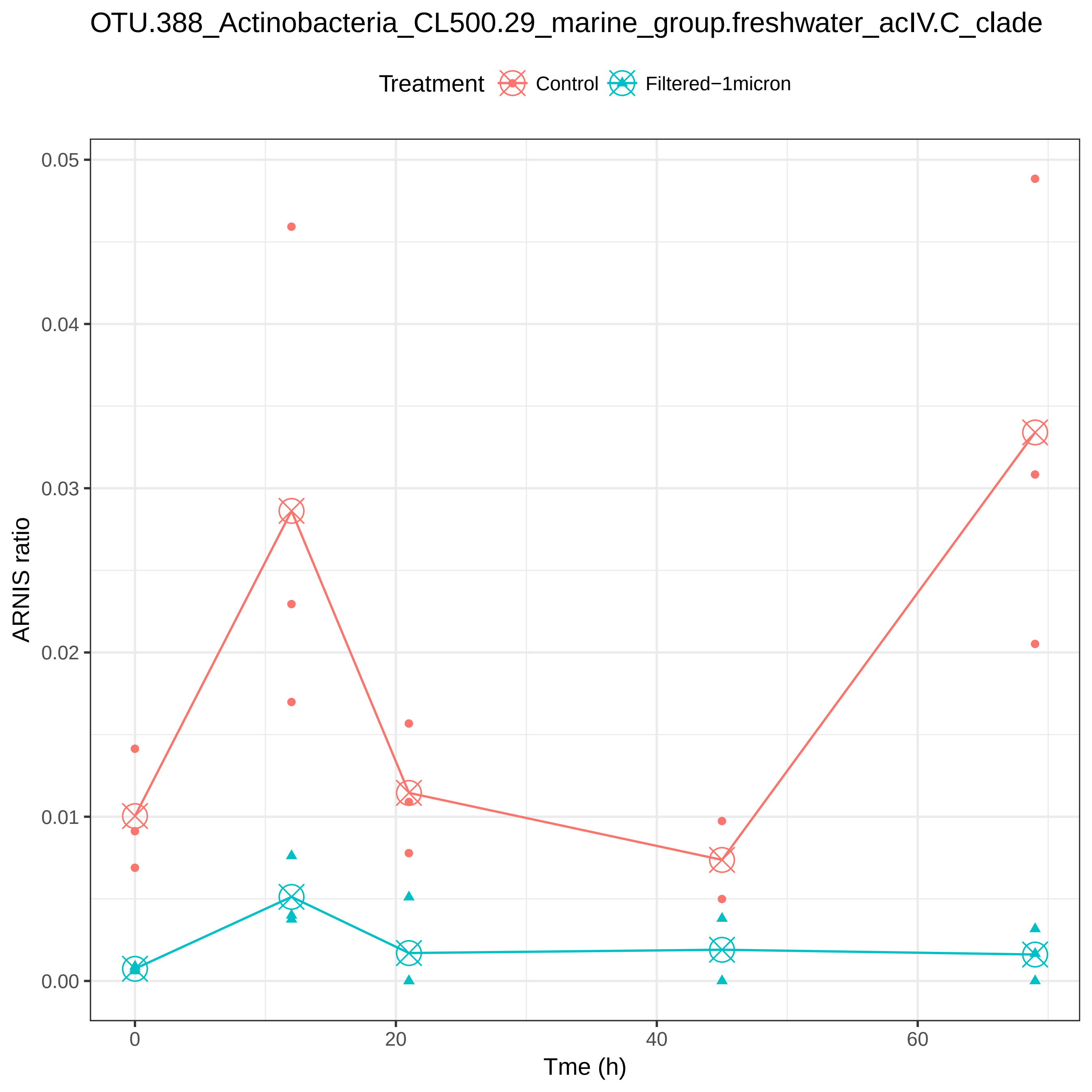

OTU.388_Actinobacteria_CL500.29_marine_group.freshwater_acIV.C_clade
Treatment
Control
Filtered−1micron
ARNIS ratio
0.05
0.04
0.03
0.02
0.01
0.00
0
20
40
60
Tme (h)

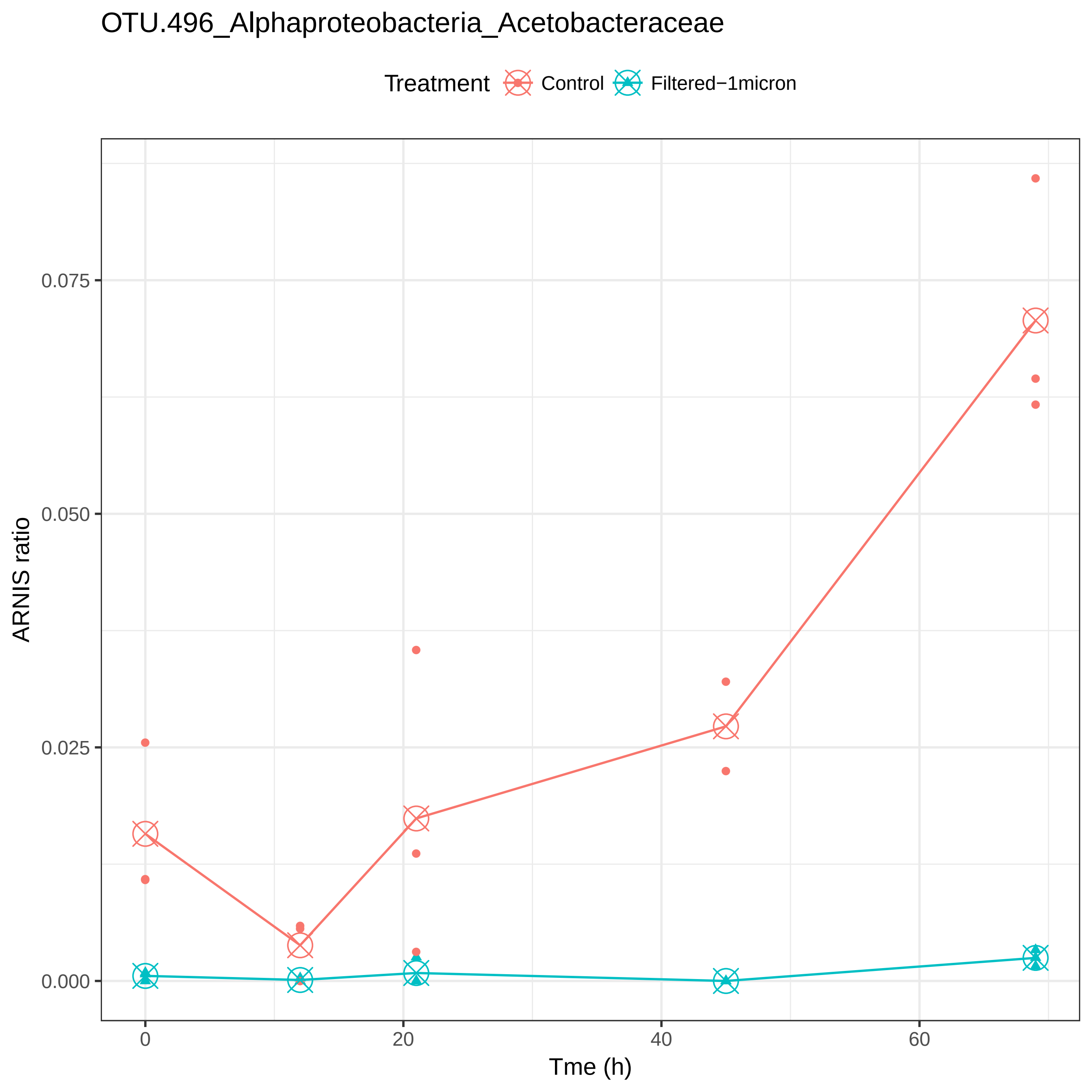

OTU.496_Alphaproteobacteria_Acetobacteraceae
Treatment
Control
Filtered−1micron
ARNIS ratio
0.075
0.050
0.025
0.000
0
20
40
60
Tme (h)

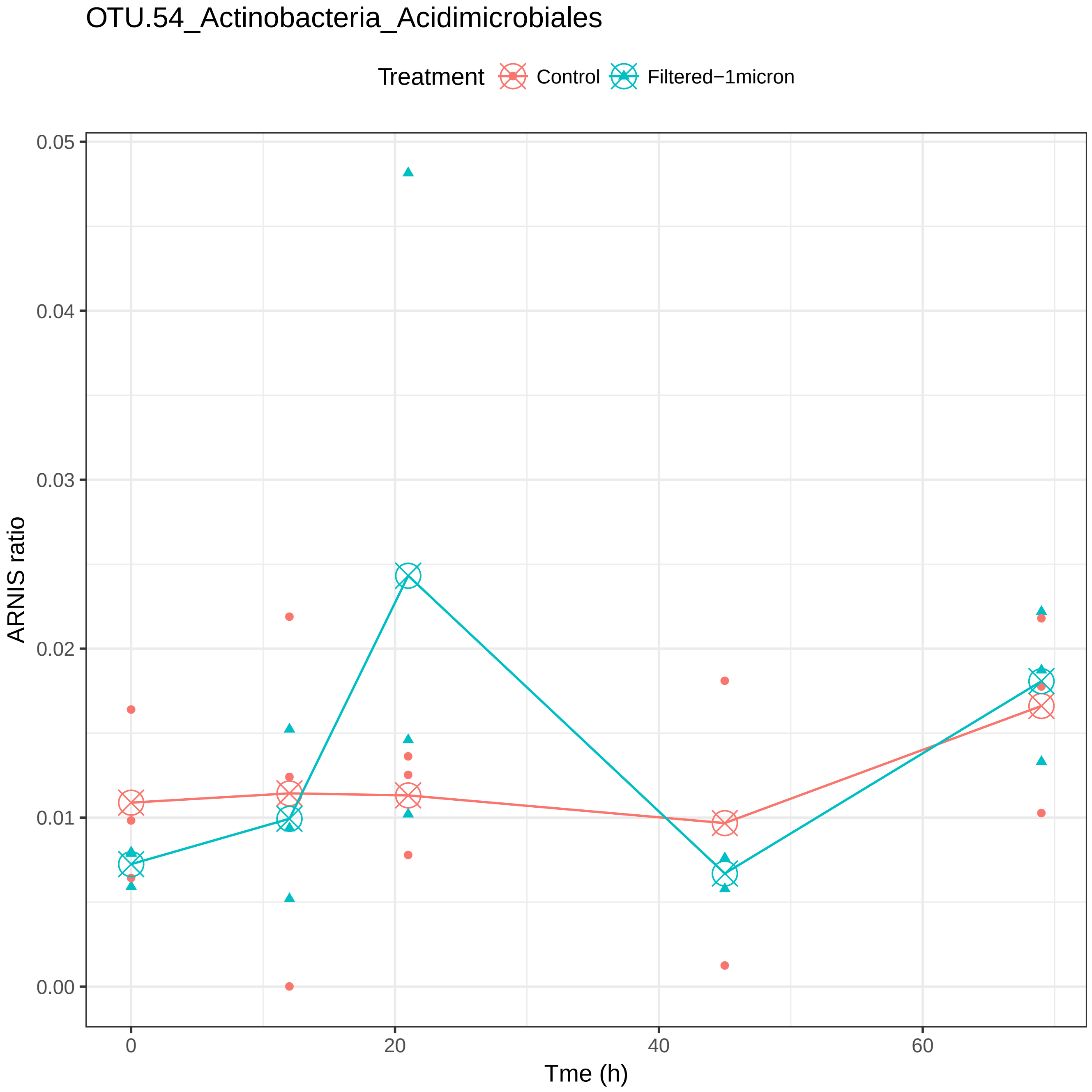
OTU.54_Actinobacteria_Acidimicrobiales
Treatment
Control
Filtered−1micron
ARNIS ratio
0.05
0.04
0.03
0.02
0.01
0.00
0
20
40
60
Tme (h)

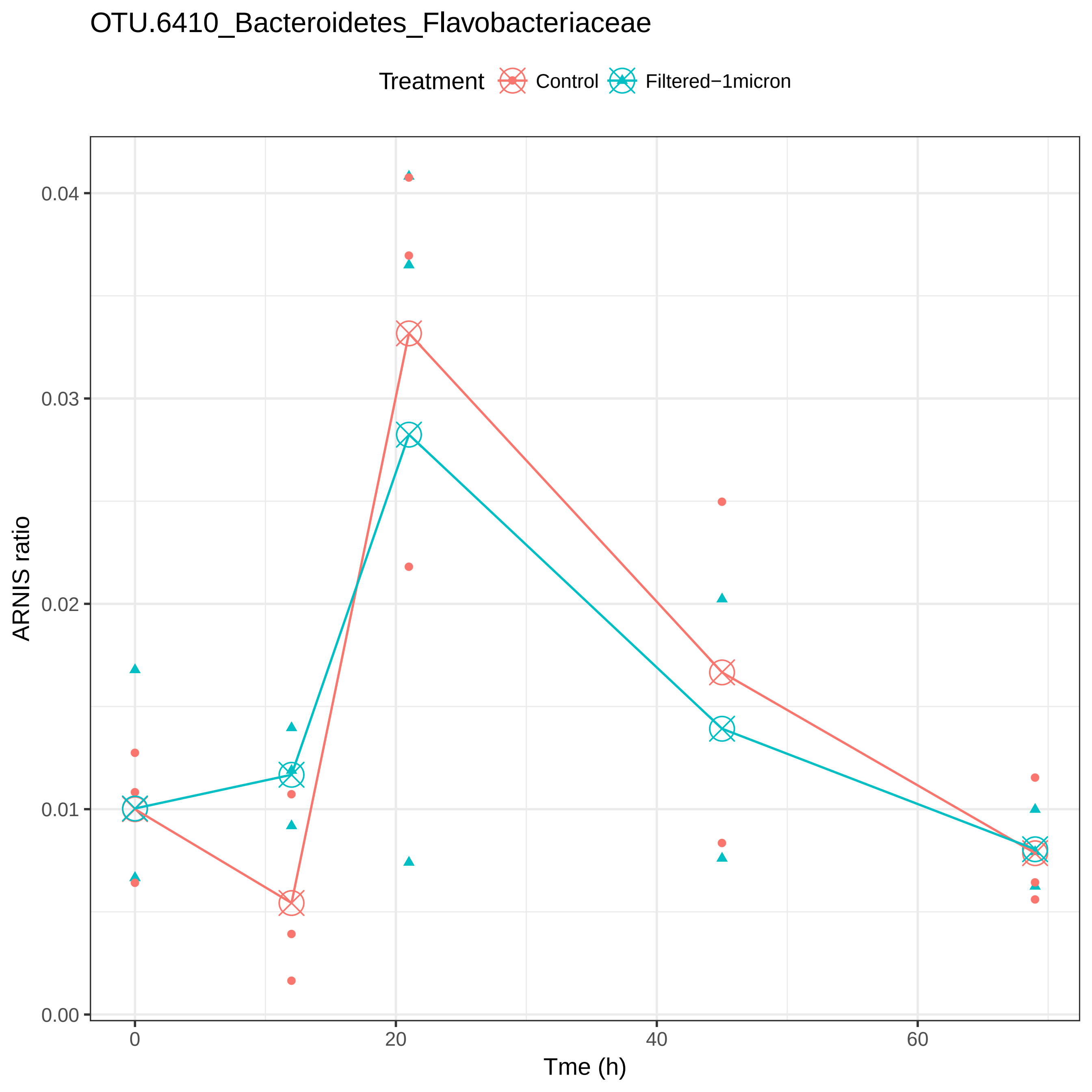
OTU.6410_Bacteroidetes_Flavobacteriaceae
Treatment
Control
Filtered−1micron
0.00
0.01
0.02
0.03
0.04
ARNIS ratio
0
20
40
60
Tme (h)

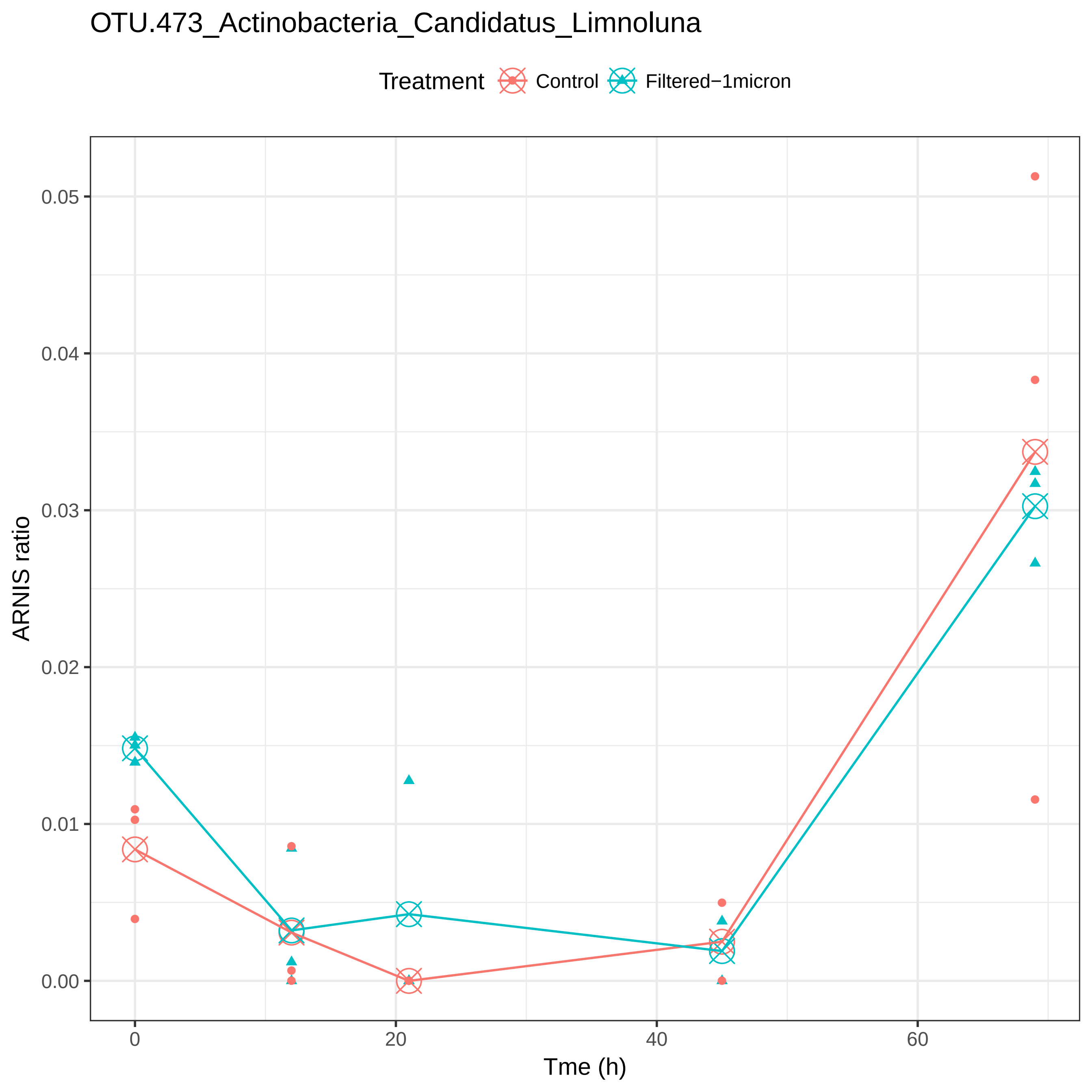
OTU.473_Actinobacteria_Candidatus_Limnoluna
Treatment
Control
Filtered−1micron
ARNIS ratio
0.00
0.01
0.02
0.03
0.04
0.05
0
20
40
60
Tme (h)

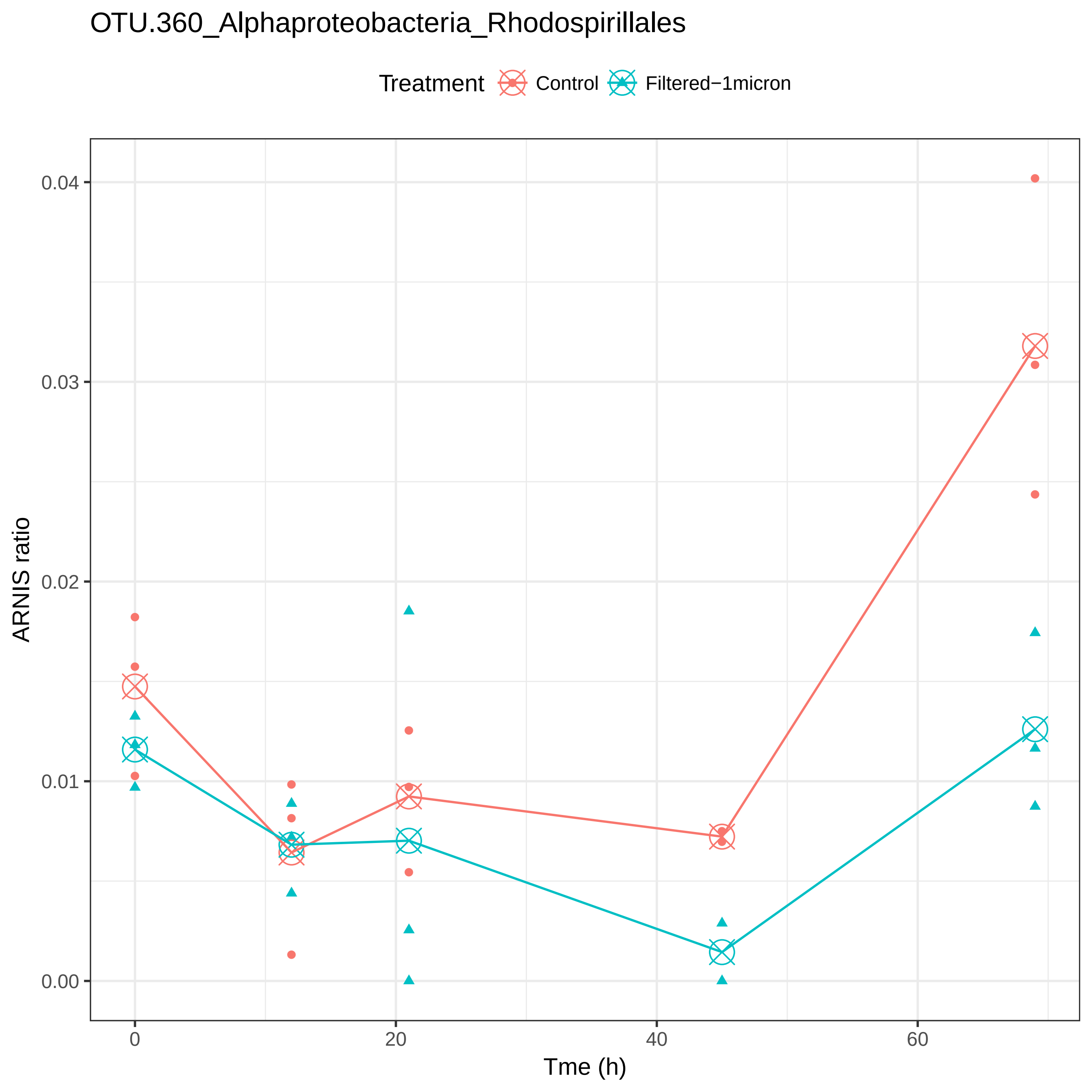

OTU.360_Alphaproteobacteria_Rhodospirillales
Treatment
Control
Filtered−1micron
ARNIS ratio
0.00
0.01
0.02
0.03
0.04
0
20
40
60
Tme (h)

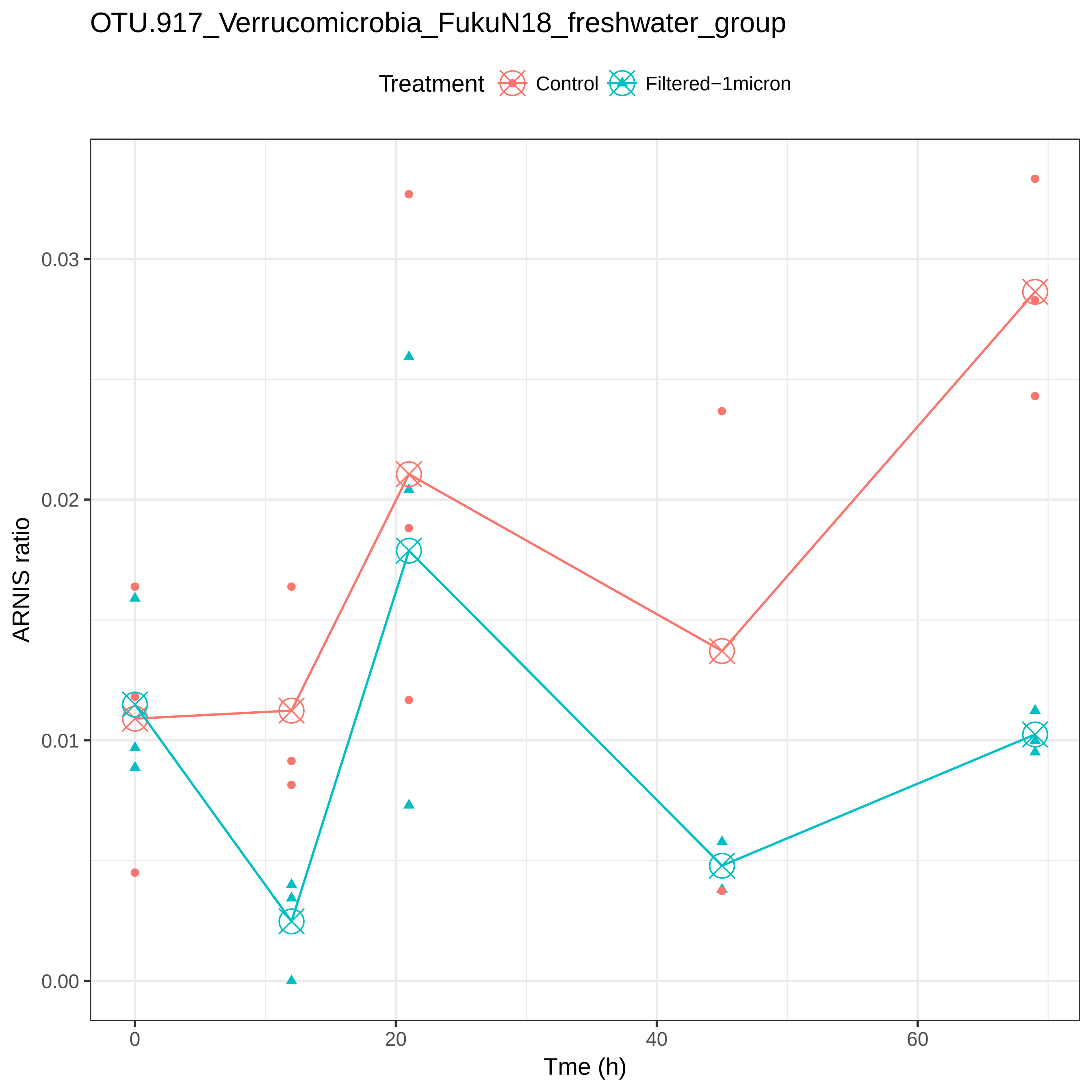

OTU.917_Verrucomicrobia_FukuN18_freshwater_group
Treatment
Control
Filtered−1micron
ARNIS ratio
0.00
0.01
0.02
0.03
0
20
40
60
Tme (h)

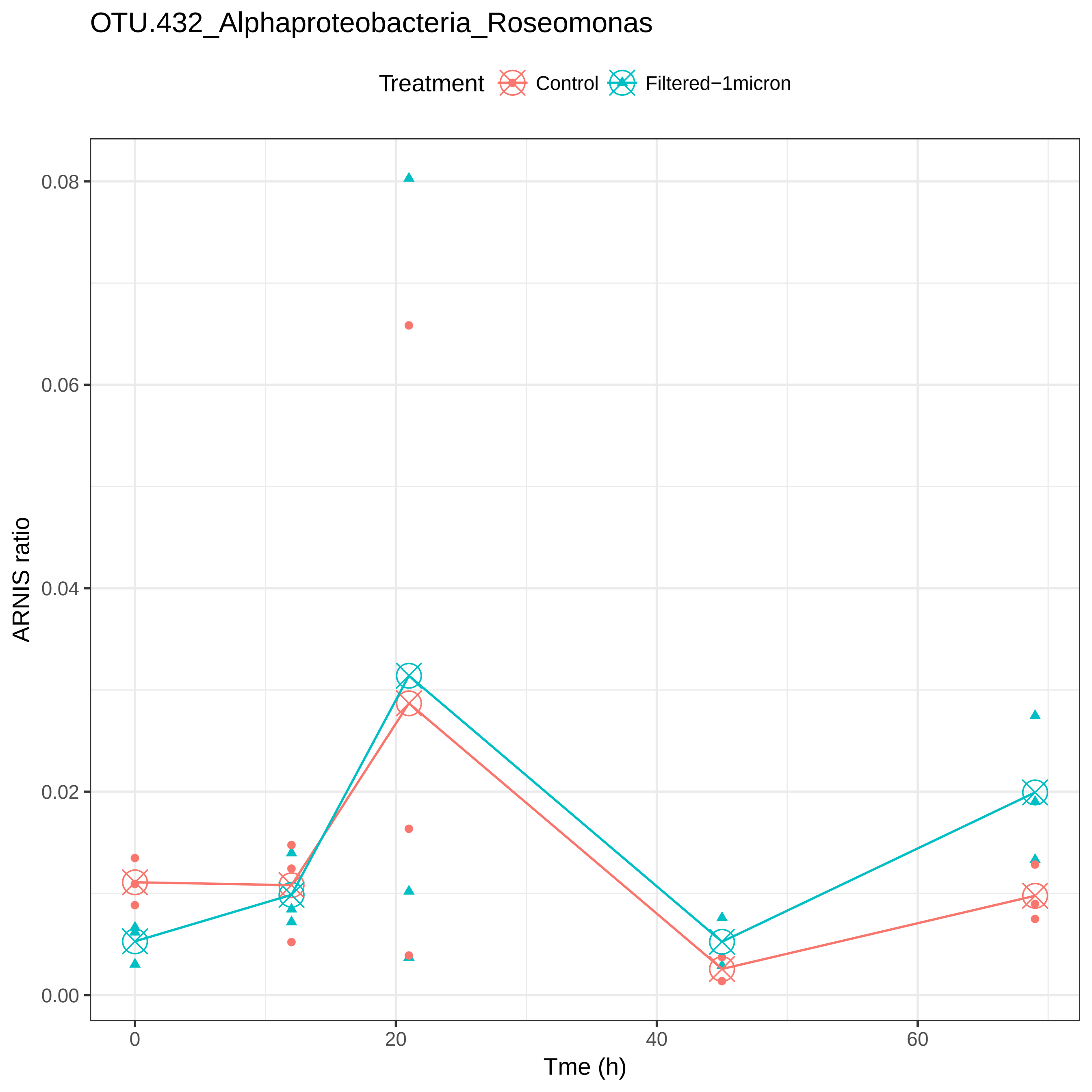
OTU.432_Alphaproteobacteria_Roseomonas
Treatment
Control
Filtered−1micron
ARNIS ratio
0.00
0.02
0.04
0.06
0.08
Tme (h)
0
20
40
60

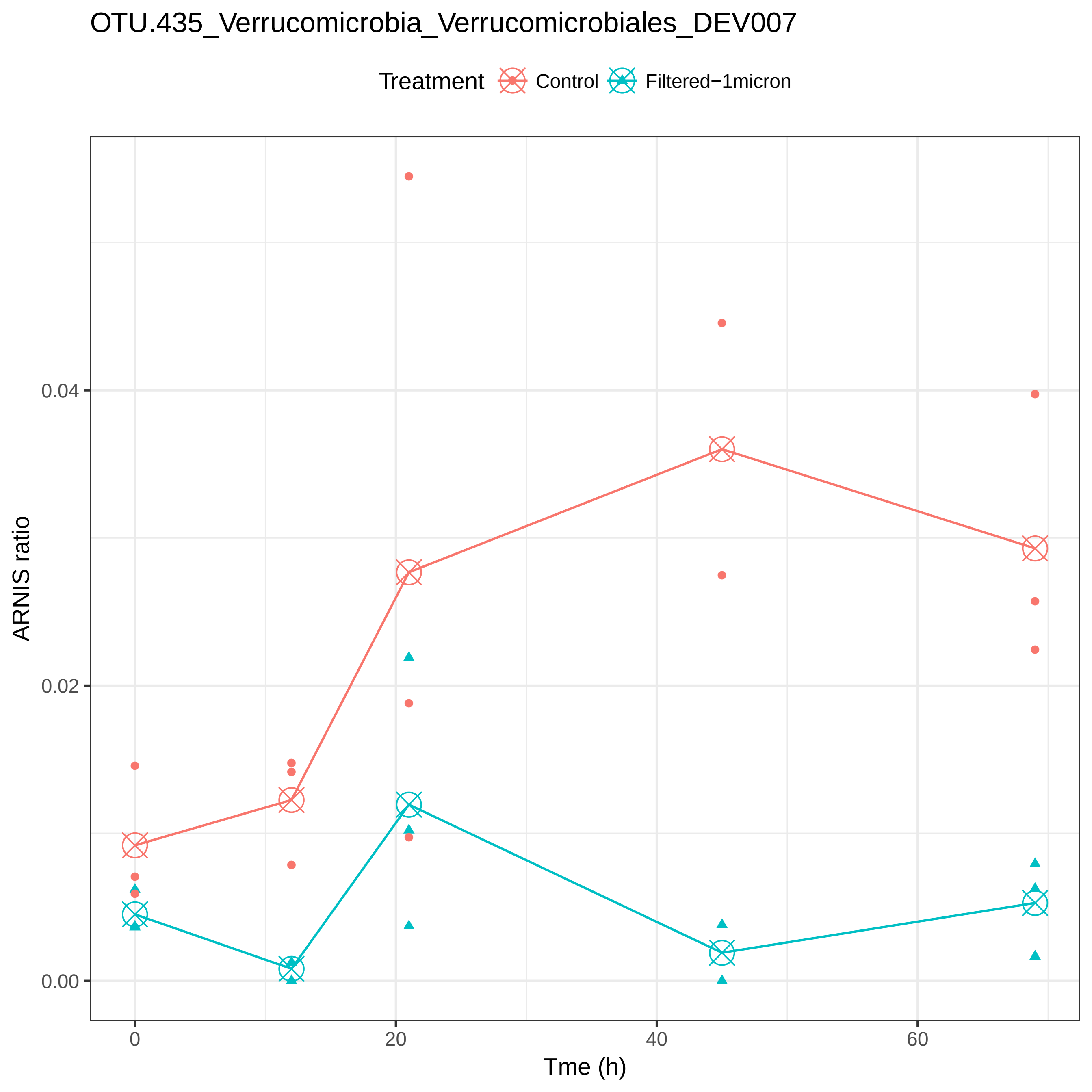

OTU.435_Verrucomicrobia_Verrucomicrobiales_DEV007
Treatment
Control
Filtered−1micron
ARNIS ratio
0.00
0.02
0.04
0
20
40
60
Tme (h)

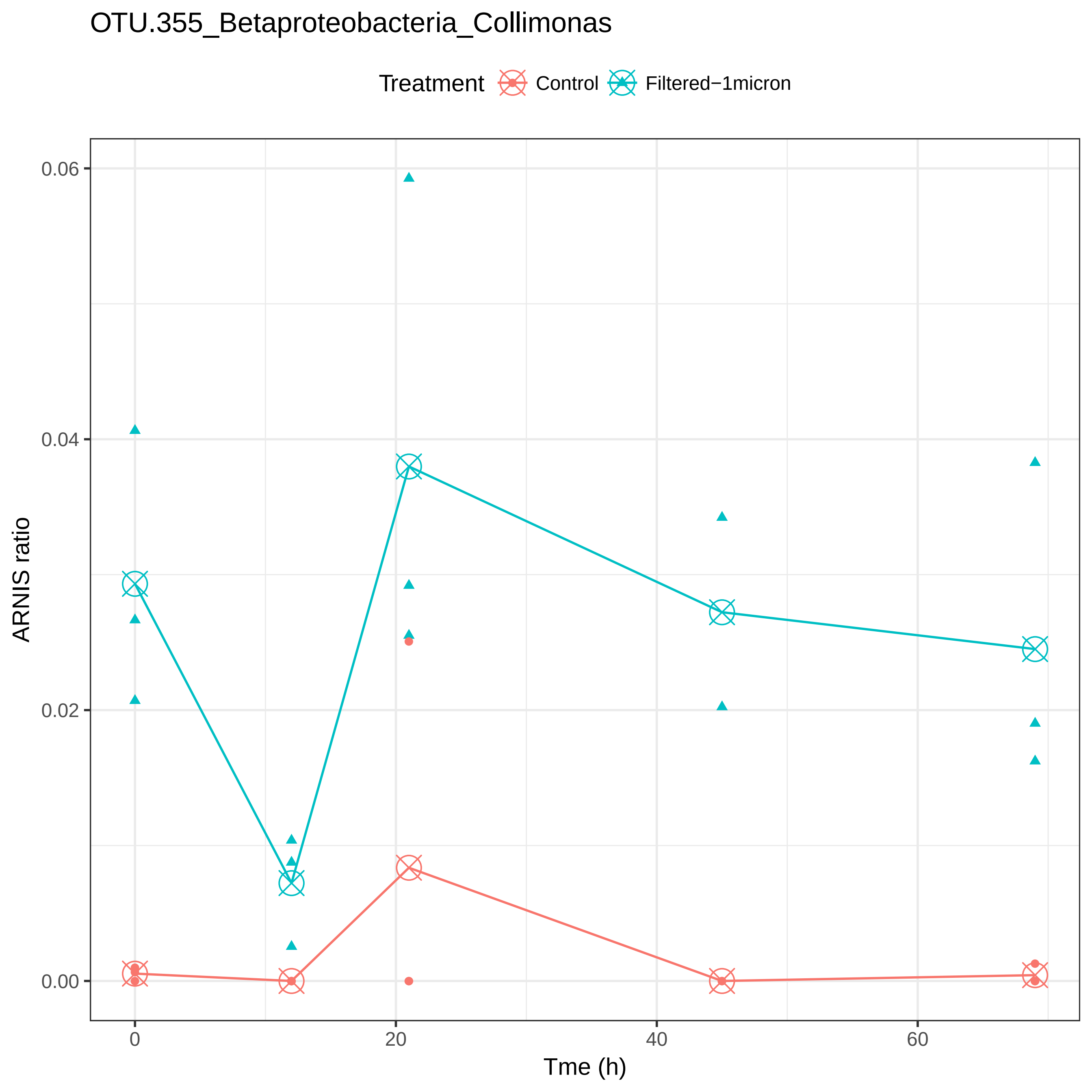

OTU.355_Betaproteobacteria_Collimonas
Treatment
Control
Filtered−1micron
ARNIS ratio
0.06
0.04
0.02
0.00
0
20
40
60
Tme (h)

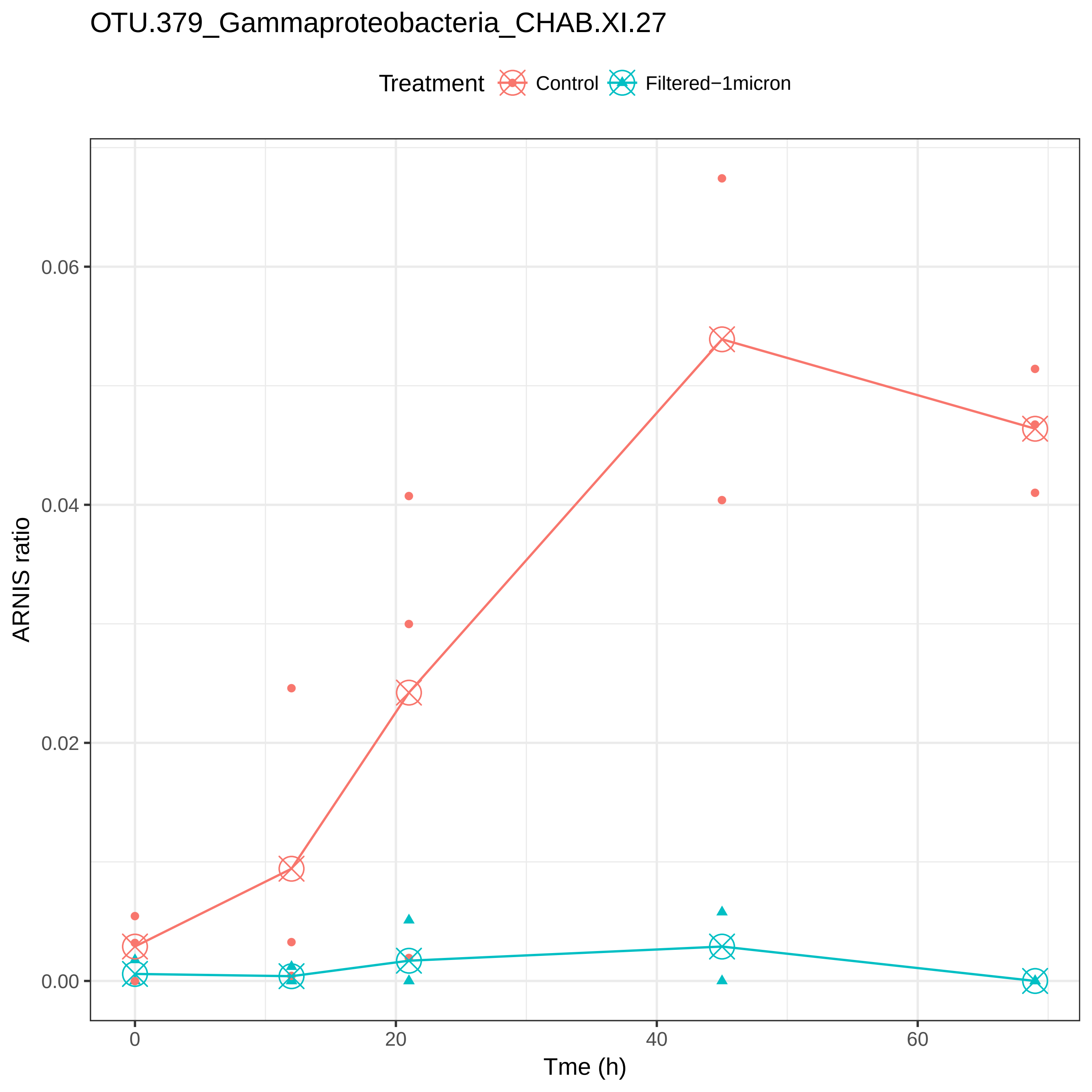

OTU.379_Gammaproteobacteria_CHAB.XI.27
Treatment
Control
Filtered−1micron
0.06
0.04
0.02
0.00
ARNIS ratio
0
20
40
60
Tme (h)

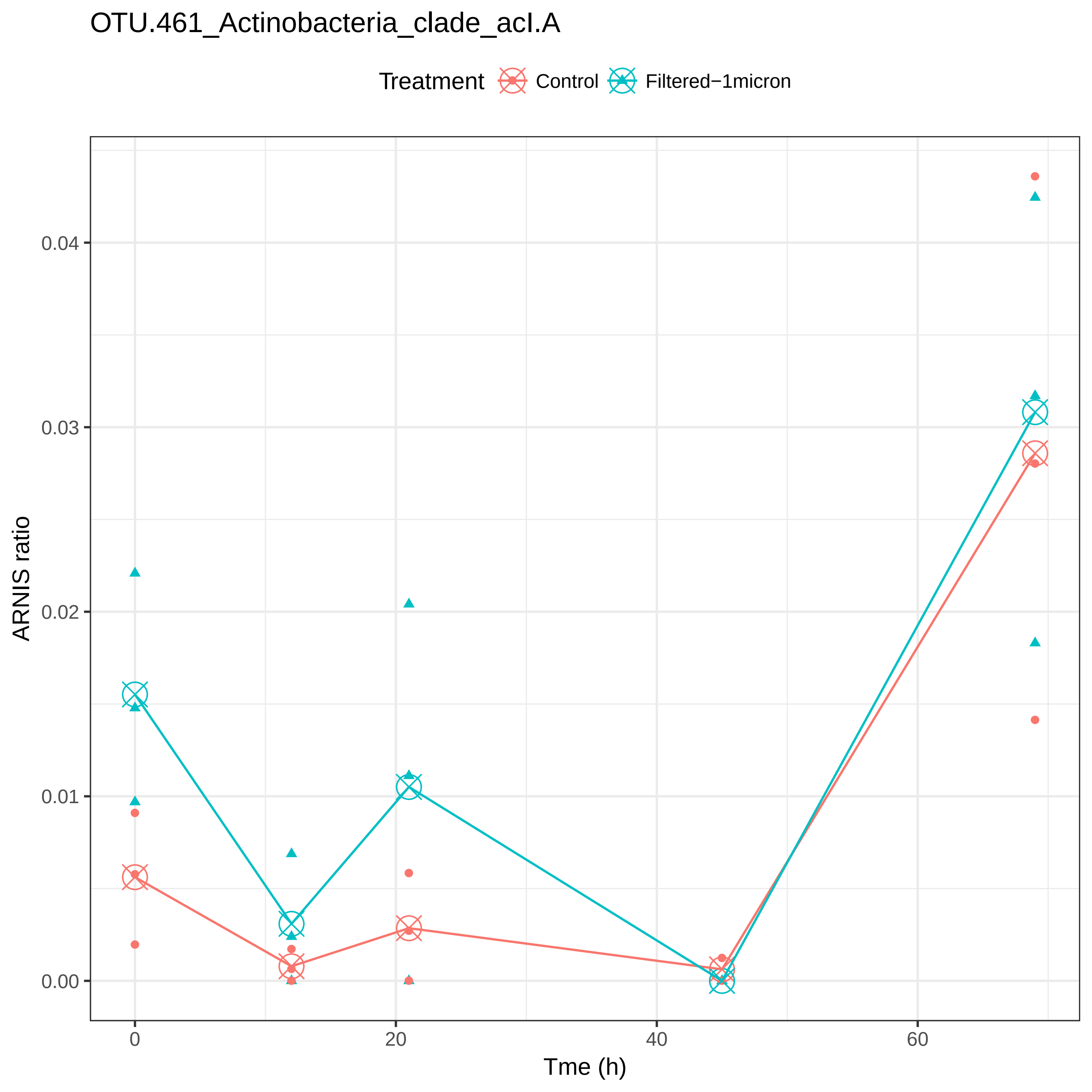
OTU.461_Actinobacteria_clade_acI.A
Treatment
Control
Filtered−1micron
0.04
0.03
0.02
0.01
0.00
ARNIS ratio
0
20
40
60
Tme (h)

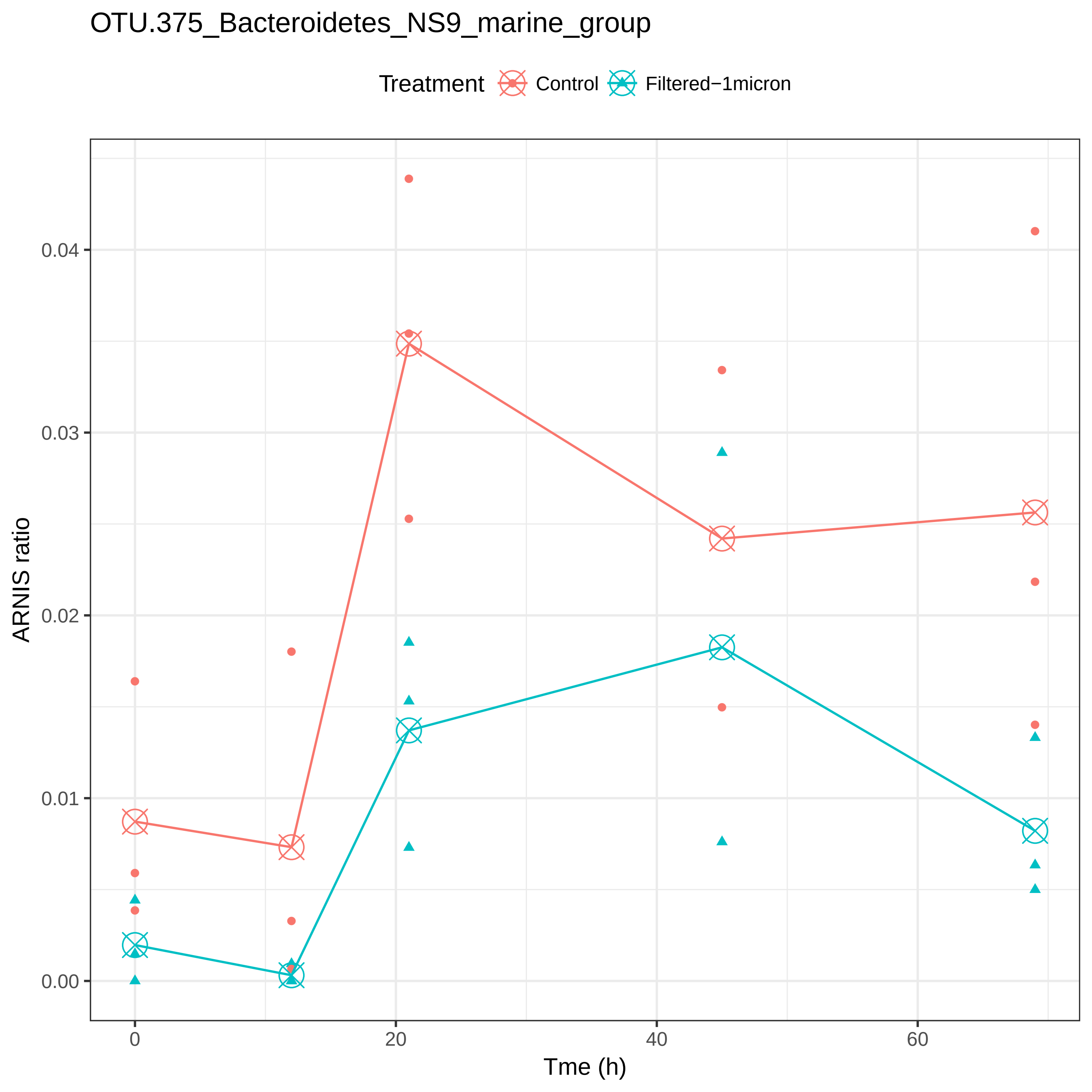

OTU.375_Bacteroidetes_NS9_marine_group
Treatment
Control
Filtered−1micron
ARNIS ratio
0.00
0.01
0.02
0.03
0.04
0
20
40
60
Tme (h)

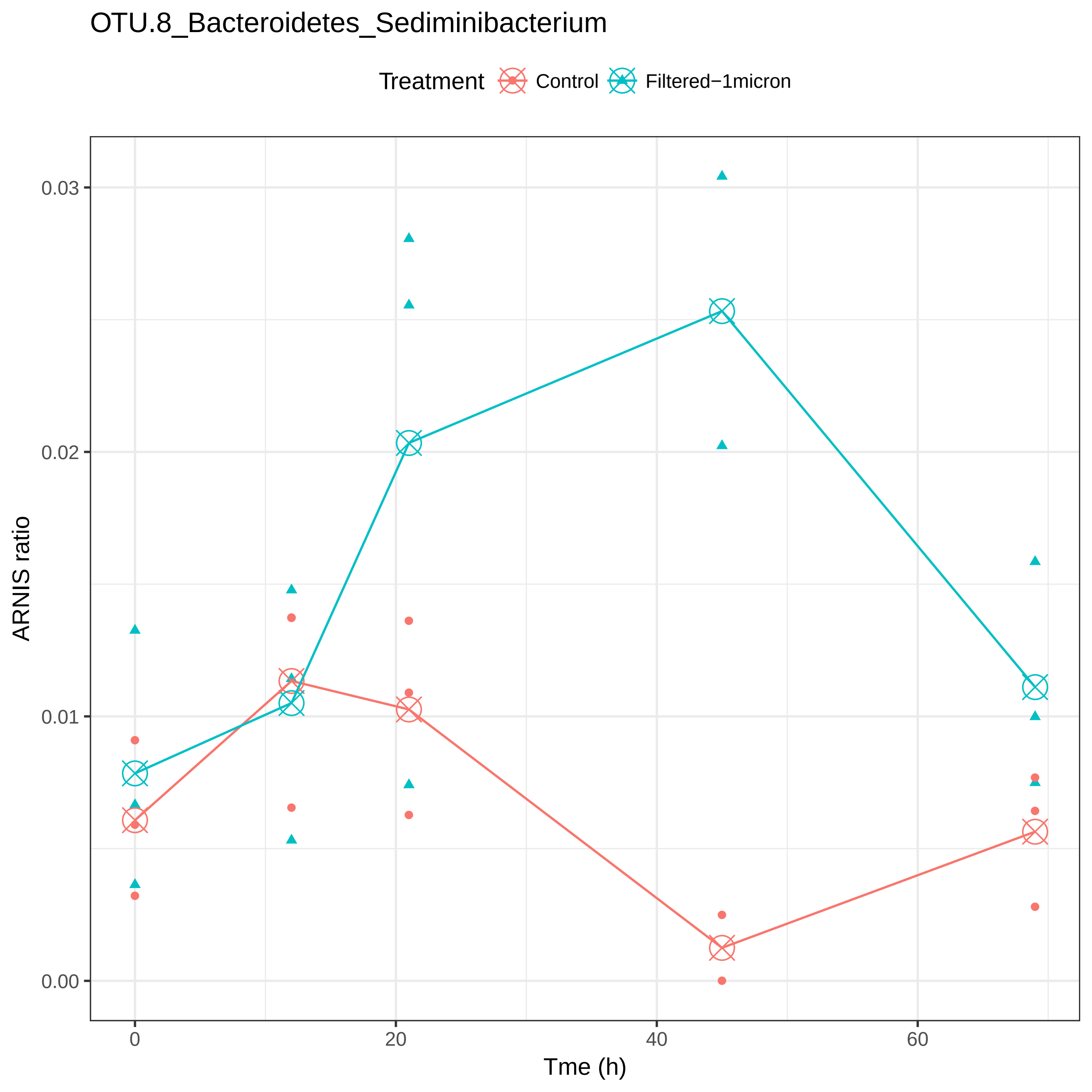

OTU.8_Bacteroidetes_Sediminibacterium
Treatment
Control
Filtered−1micron
ARNIS ratio
0.03
0.02
0.01
0.00
0
20
40
60
Tme (h)

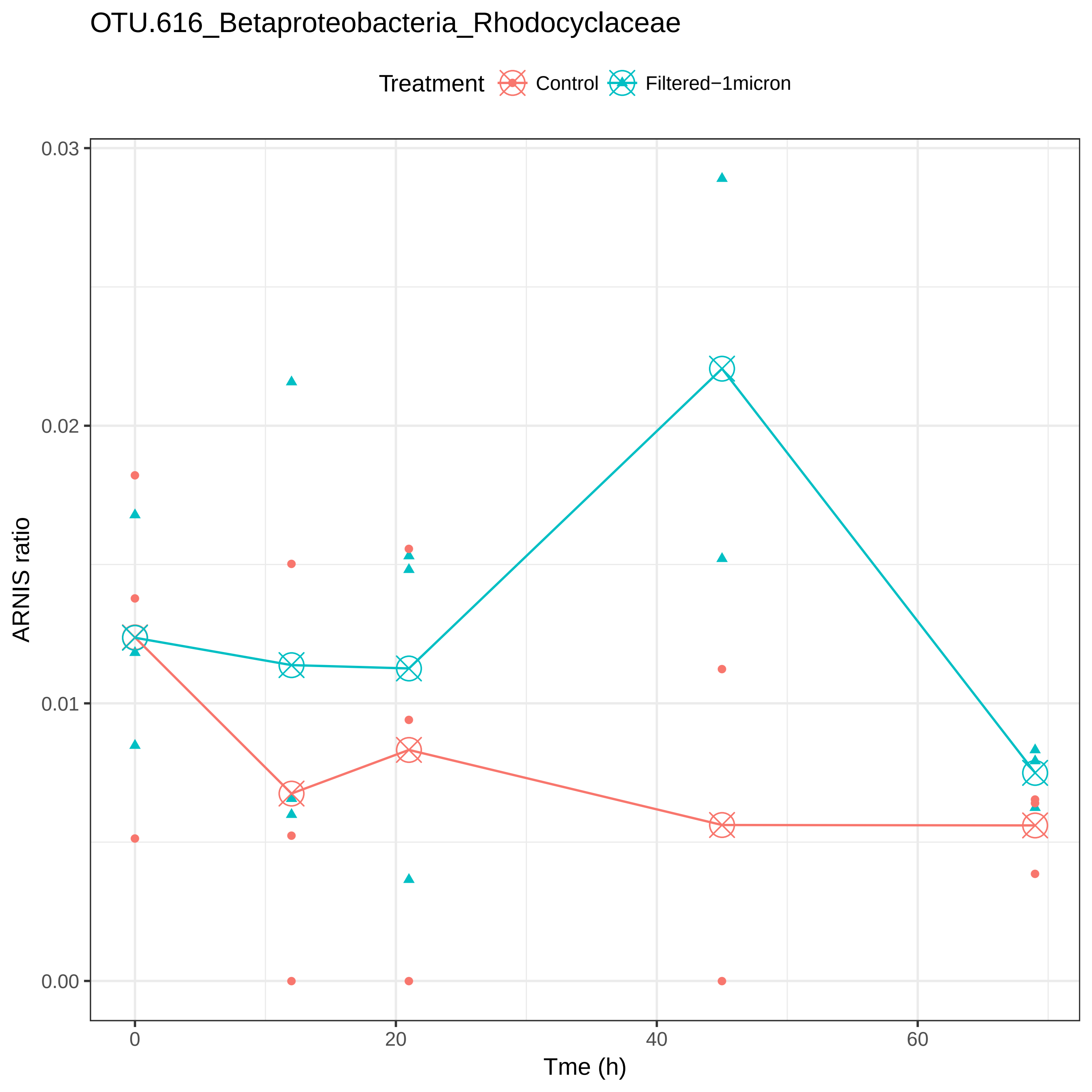

OTU.616_Betaproteobacteria_Rhodocyclaceae
Treatment
Control
Filtered−1micron
0.03
0.02
0.01
0.00
ARNIS ratio
0
20
40
60
Tme (h)

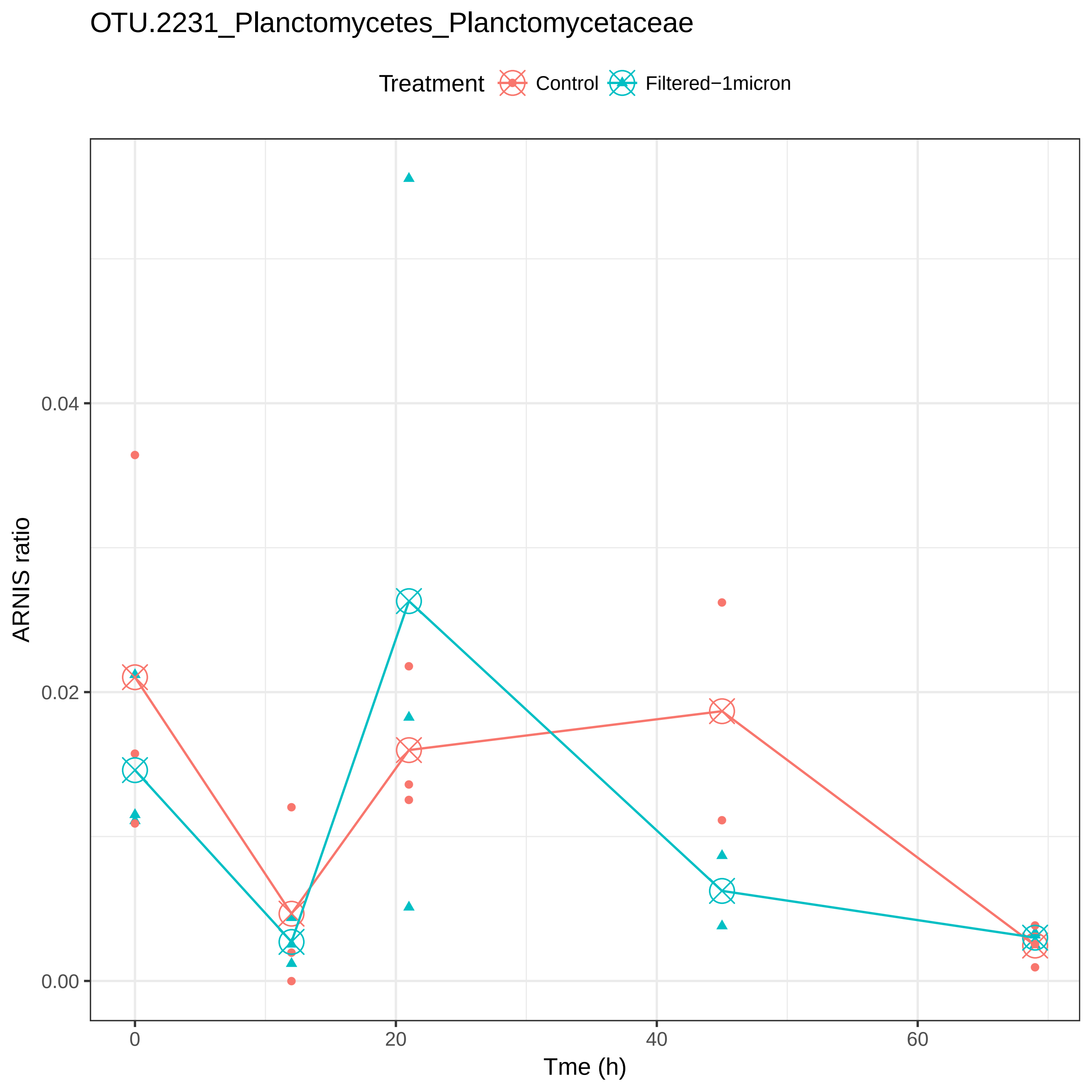

OTU.2231_Planctomycetes_Planctomycetaceae
Treatment
Control
Filtered−1micron
ARNIS ratio
0.04
0.02
0.00
0
20
40
60
Tme (h)

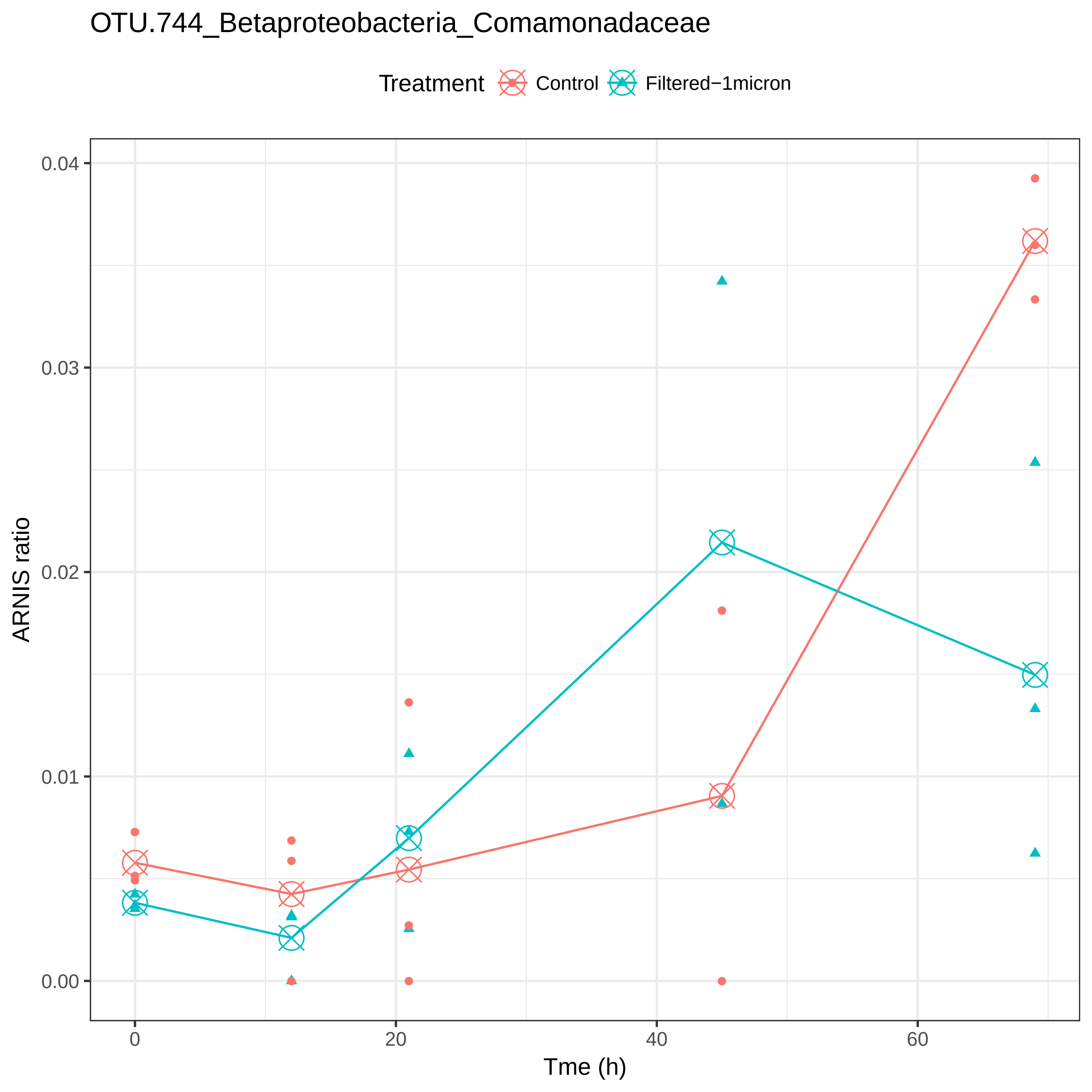
OTU.744_Betaproteobacteria_Comamonadaceae
Treatment
Control
Filtered−1micron
ARNIS ratio
0.00
0.01
0.02
0.03
0.04
0
20
40
60
Tme (h)

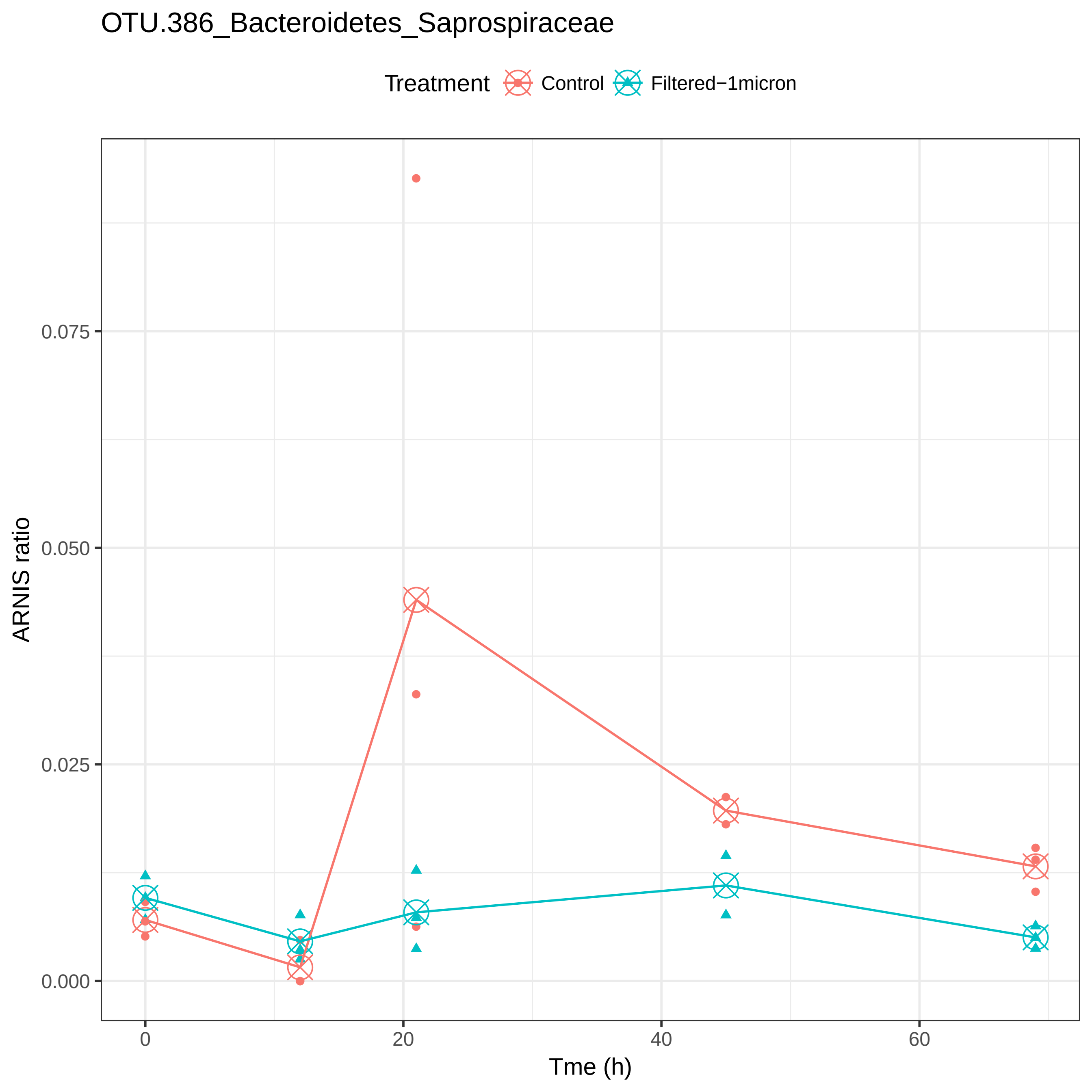
OTU.386_Bacteroidetes_Saprospiraceae
Treatment
Control
Filtered−1micron
ARNIS ratio
0.075
0.050
0.025
0.000
0
20
40
60
Tme (h)

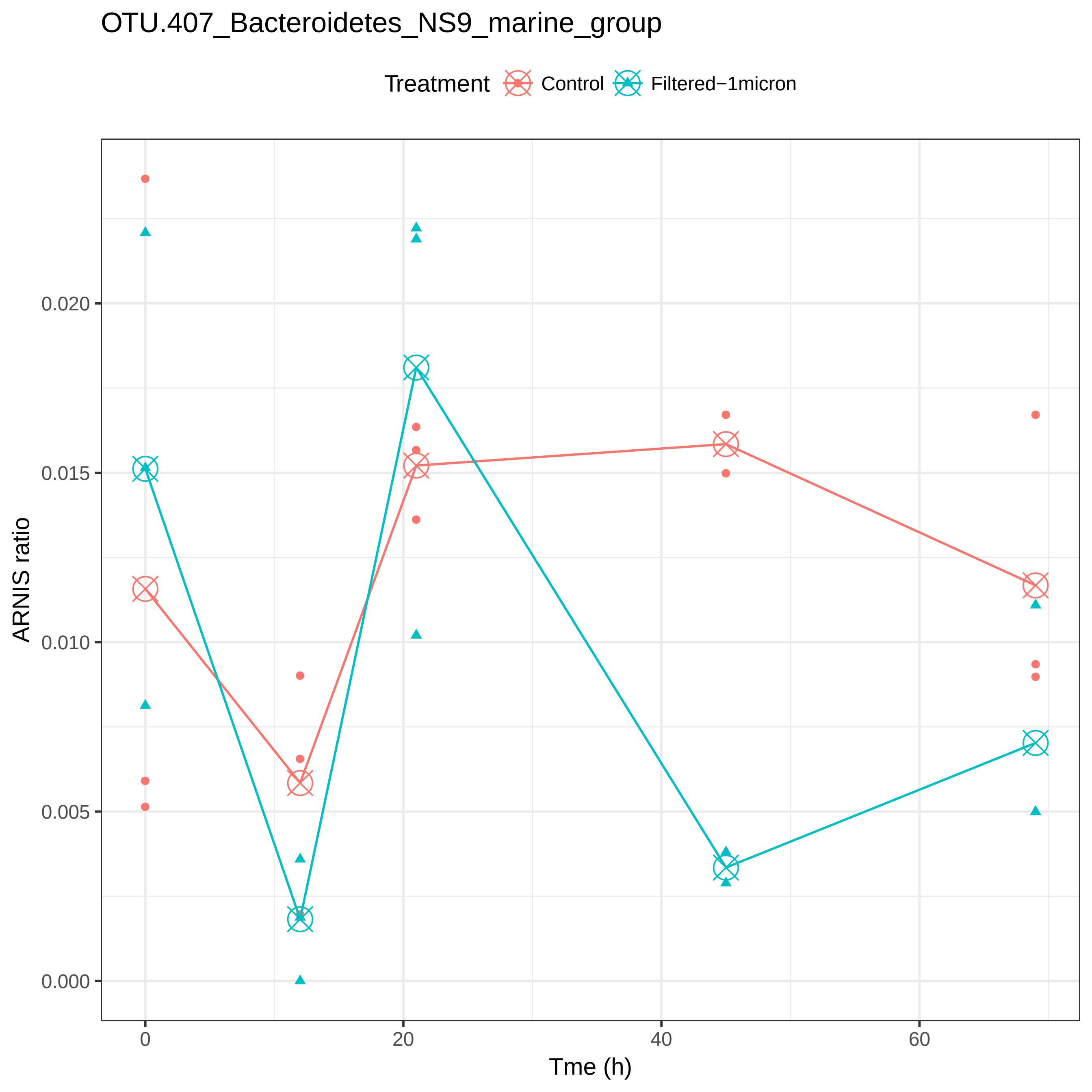
OTU.407_Bacteroidetes_NS9_marine_group
Treatment
Control
Filtered−1micron
ARNIS ratio
0.000
0.005
0.010
0.015
0.020
0
20
40
60
Tme (h)

OTU.426_Bacteroidetes_NS11.12_marine_group

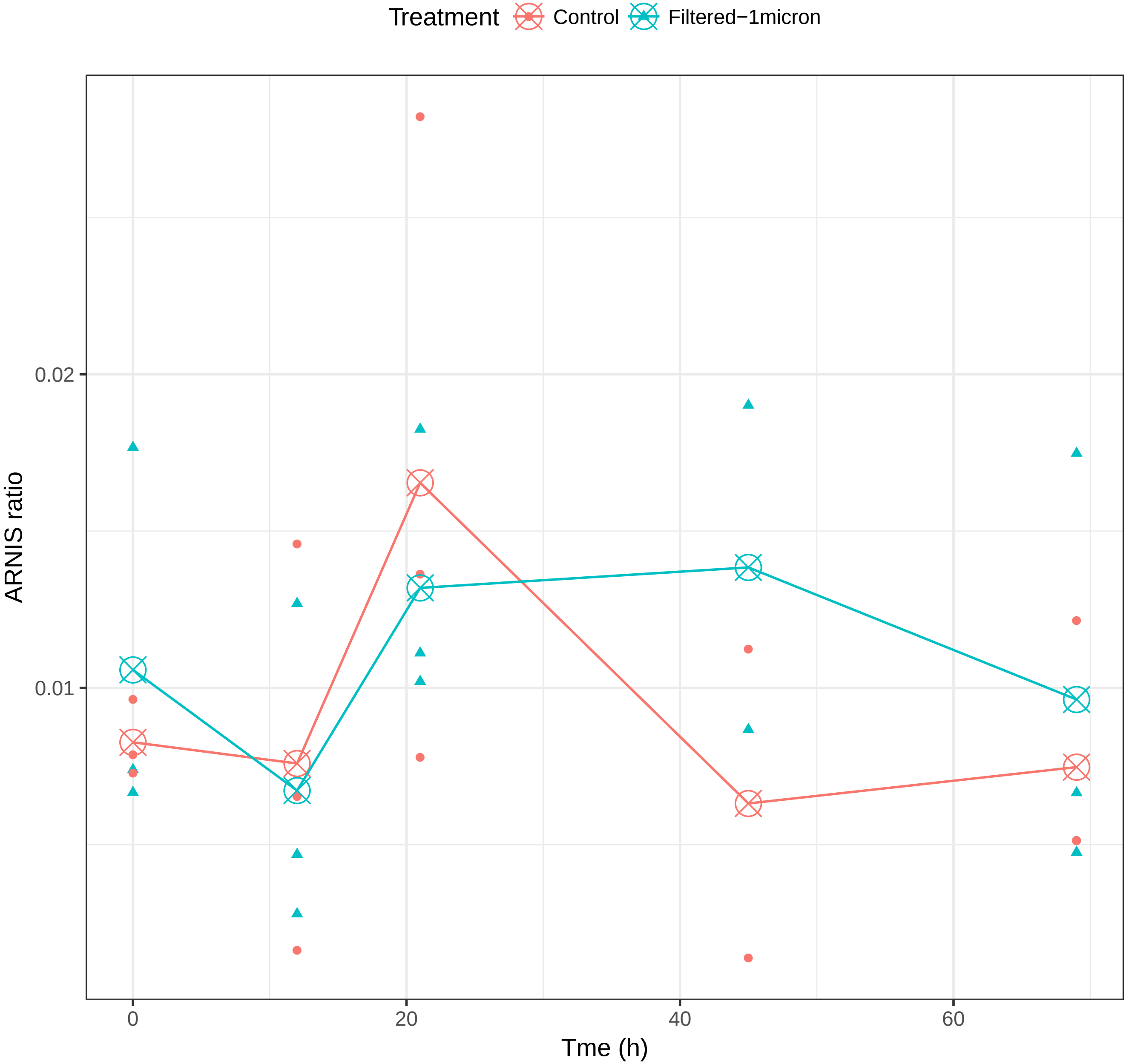

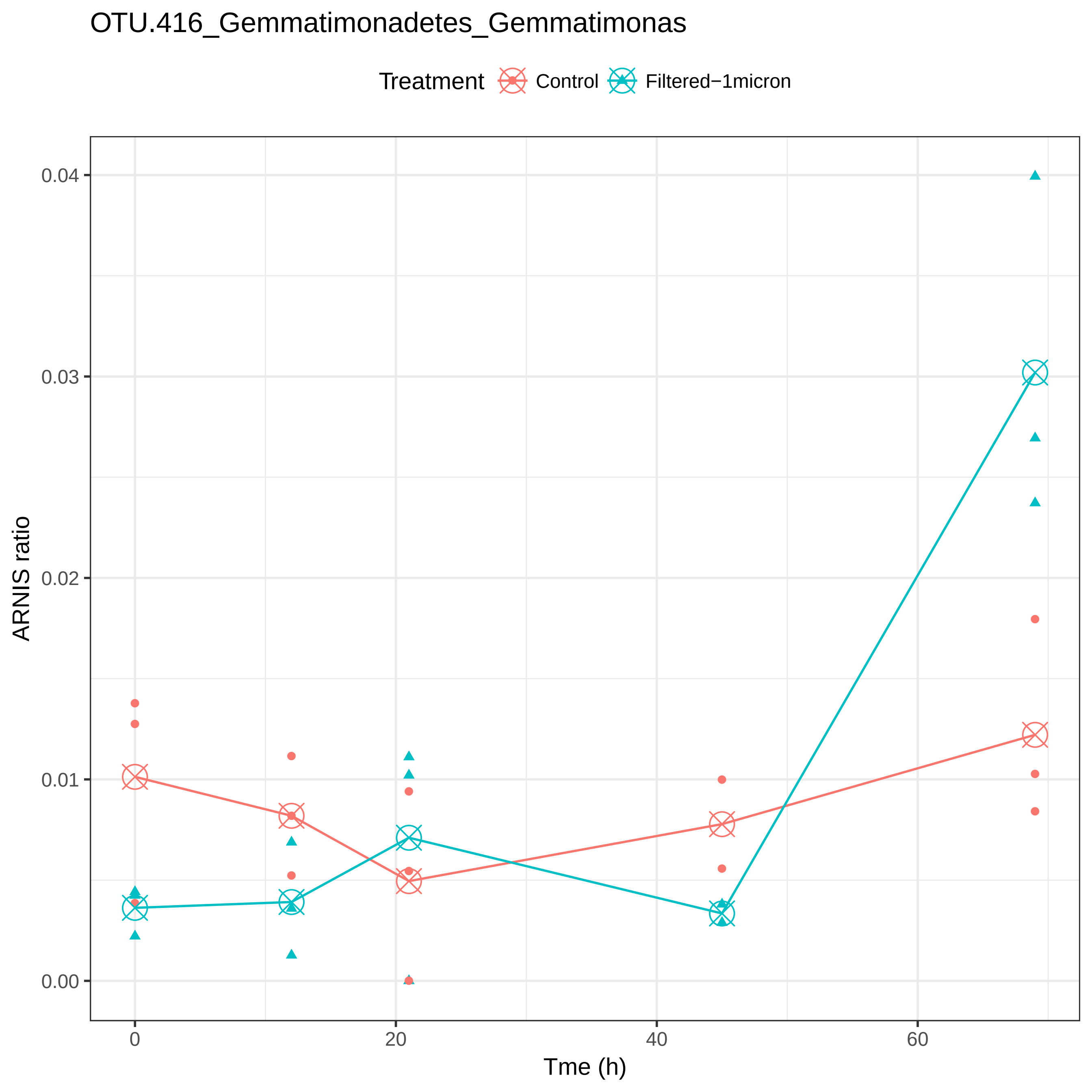
OTU.416_Gemmatimonadetes_Gemmatimonas
Treatment
Control
Filtered−1micron
ARNIS ratio
0.04
0.03
0.02
0.01
0.00
0
20
40
60
Tme (h)

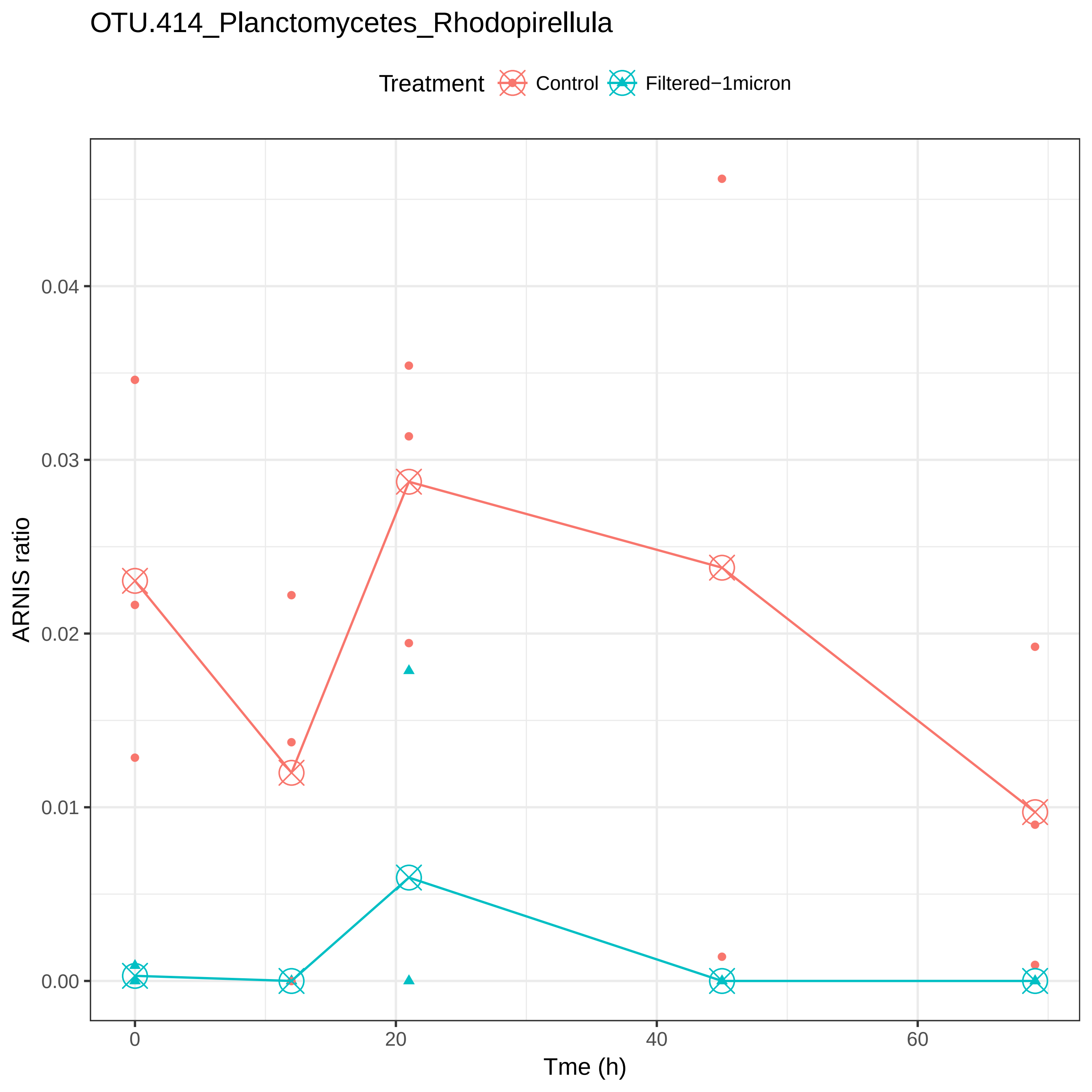

OTU.414_Planctomycetes_Rhodopirellula
Treatment
Control
Filtered−1micron
ARNIS ratio
0.00
0.01
0.02
0.03
0.04
0
20
40
60
Tme (h)

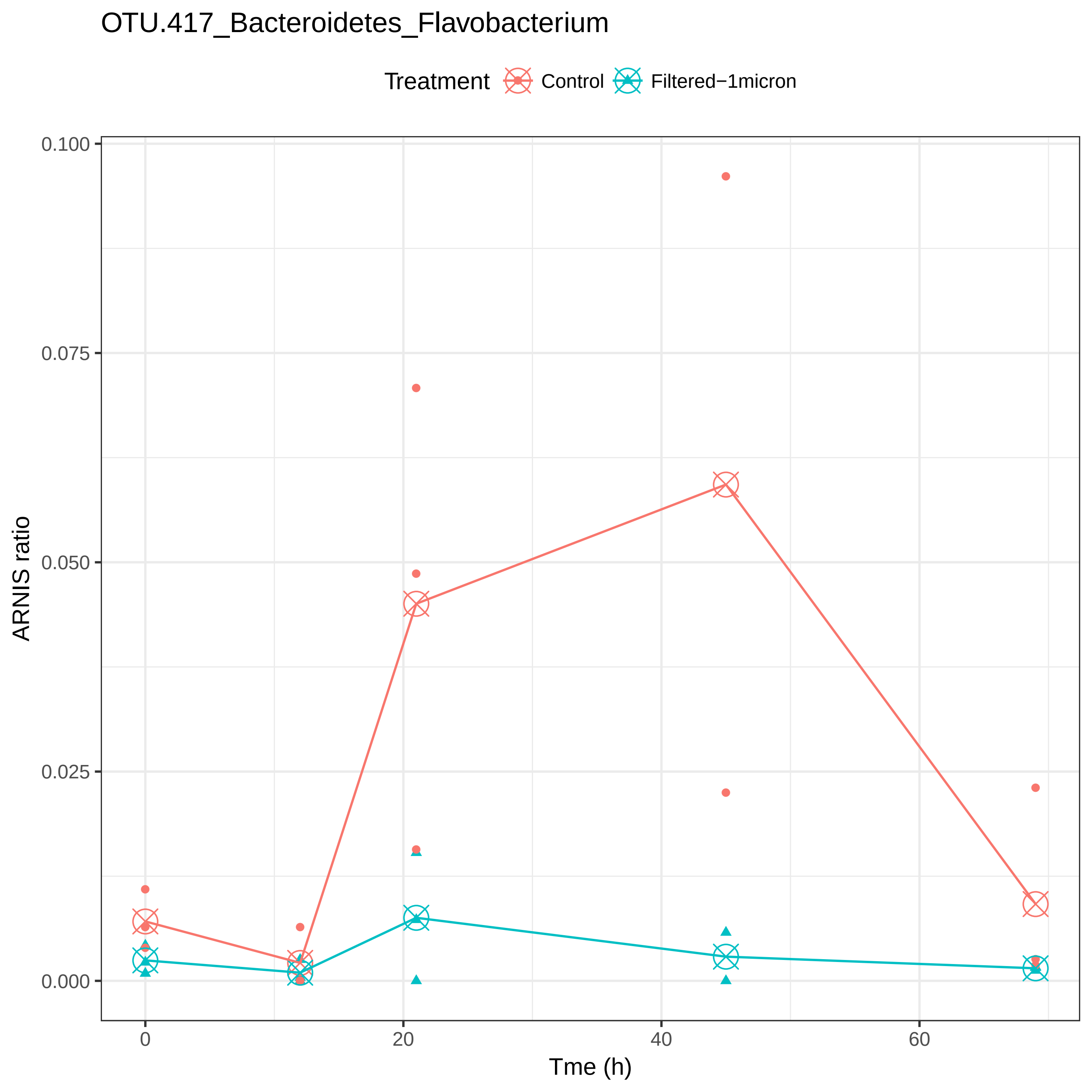

OTU.417_Bacteroidetes_Flavobacterium
Treatment
Control
Filtered−1micron
ARNIS ratio
0.100
0.075
0.050
0.025
0.000
0
20
40
60
Tme (h)

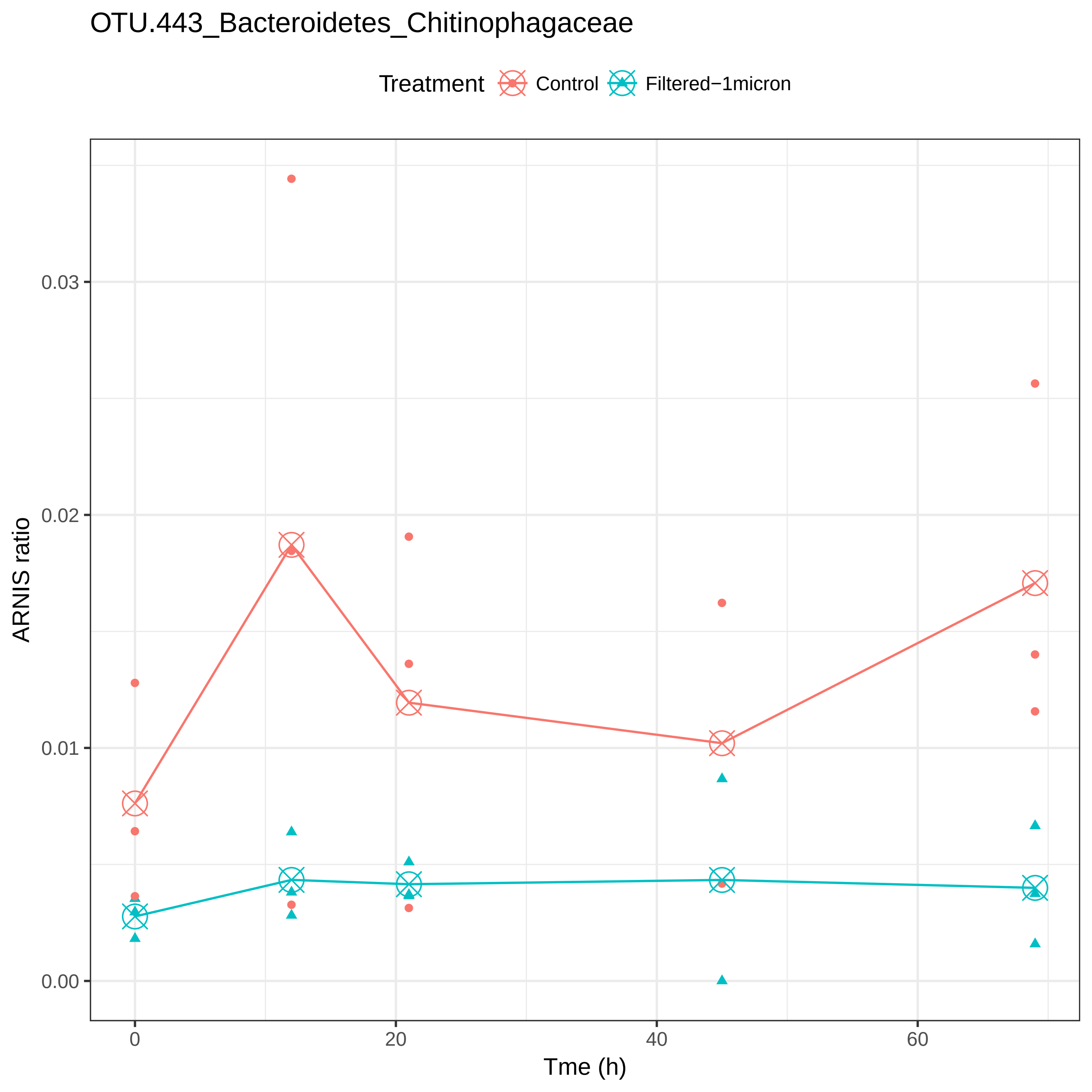
OTU.443_Bacteroidetes_Chitinophagaceae
Treatment
Control
Filtered−1micron
ARNIS ratio
0.00
0.01
0.02
0.03
0
20
40
60
Tme (h)

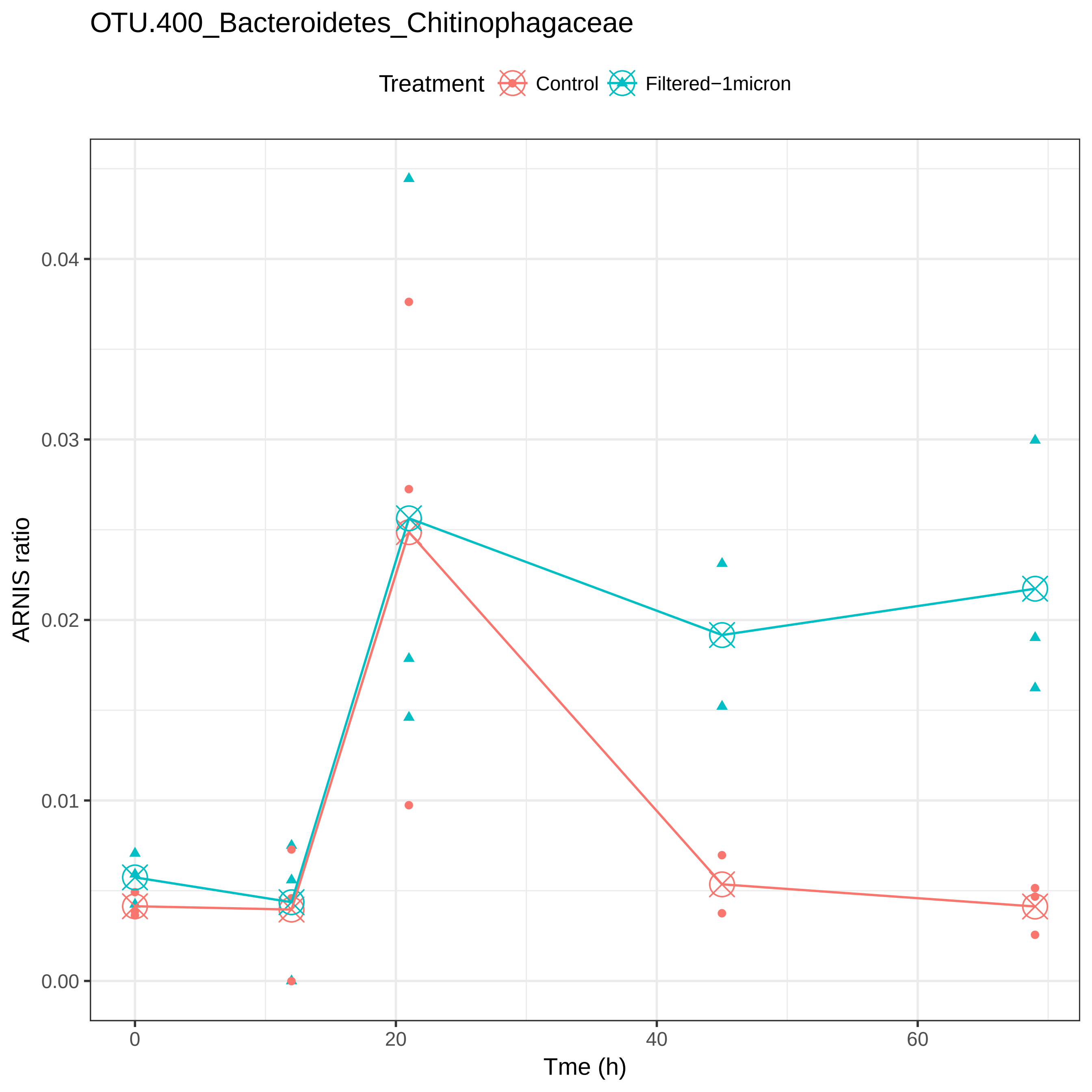

OTU.400_Bacteroidetes_Chitinophagaceae
Treatment
Control
Filtered−1micron
ARNIS ratio
0.00
0.01
0.02
0.03
0.04
0
20
40
60
Tme (h)

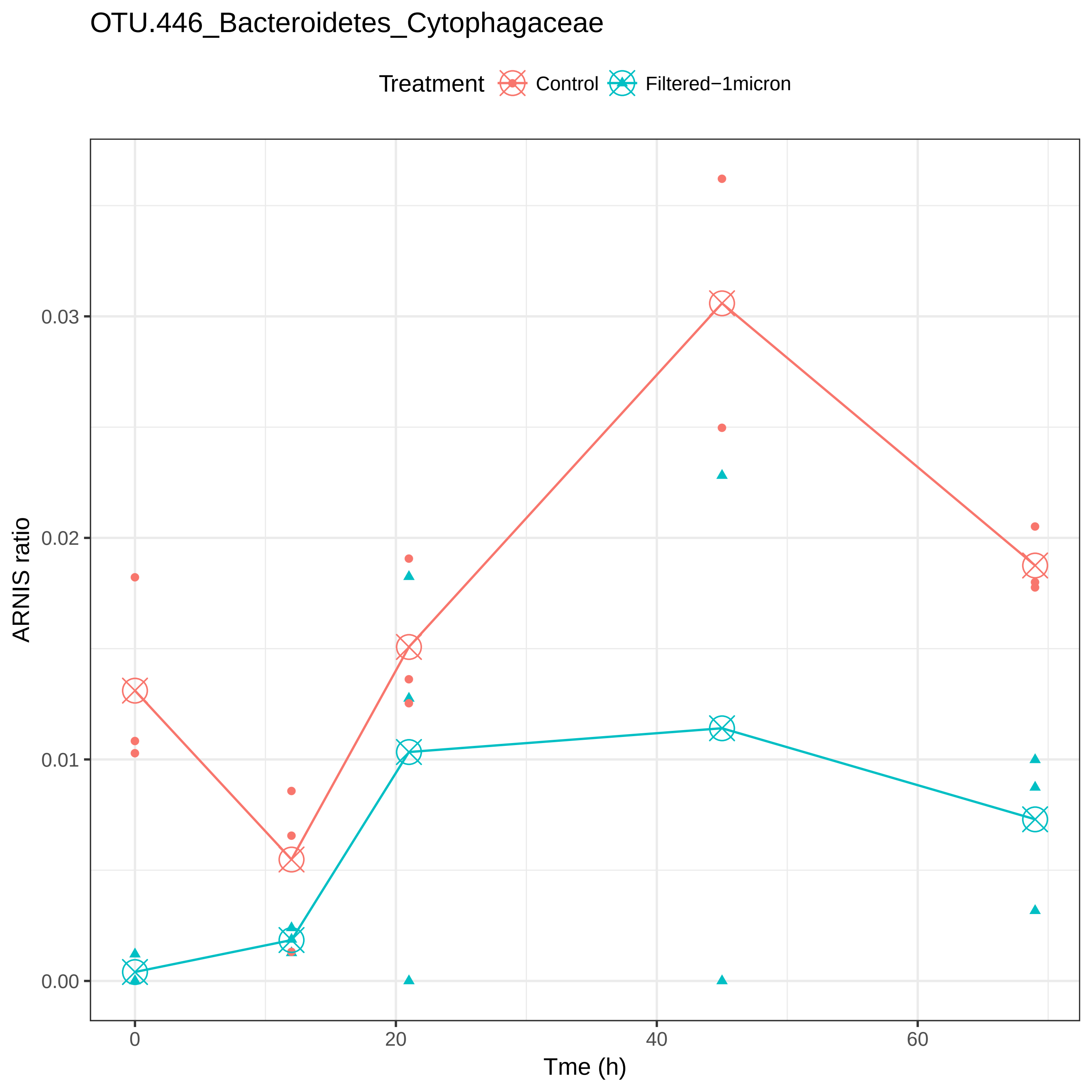
OTU.446_Bacteroidetes_Cytophagaceae
Treatment
Control
Filtered−1micron
ARNIS ratio
0.00
0.01
0.02
0.03
0
20
40
60
Tme (h)

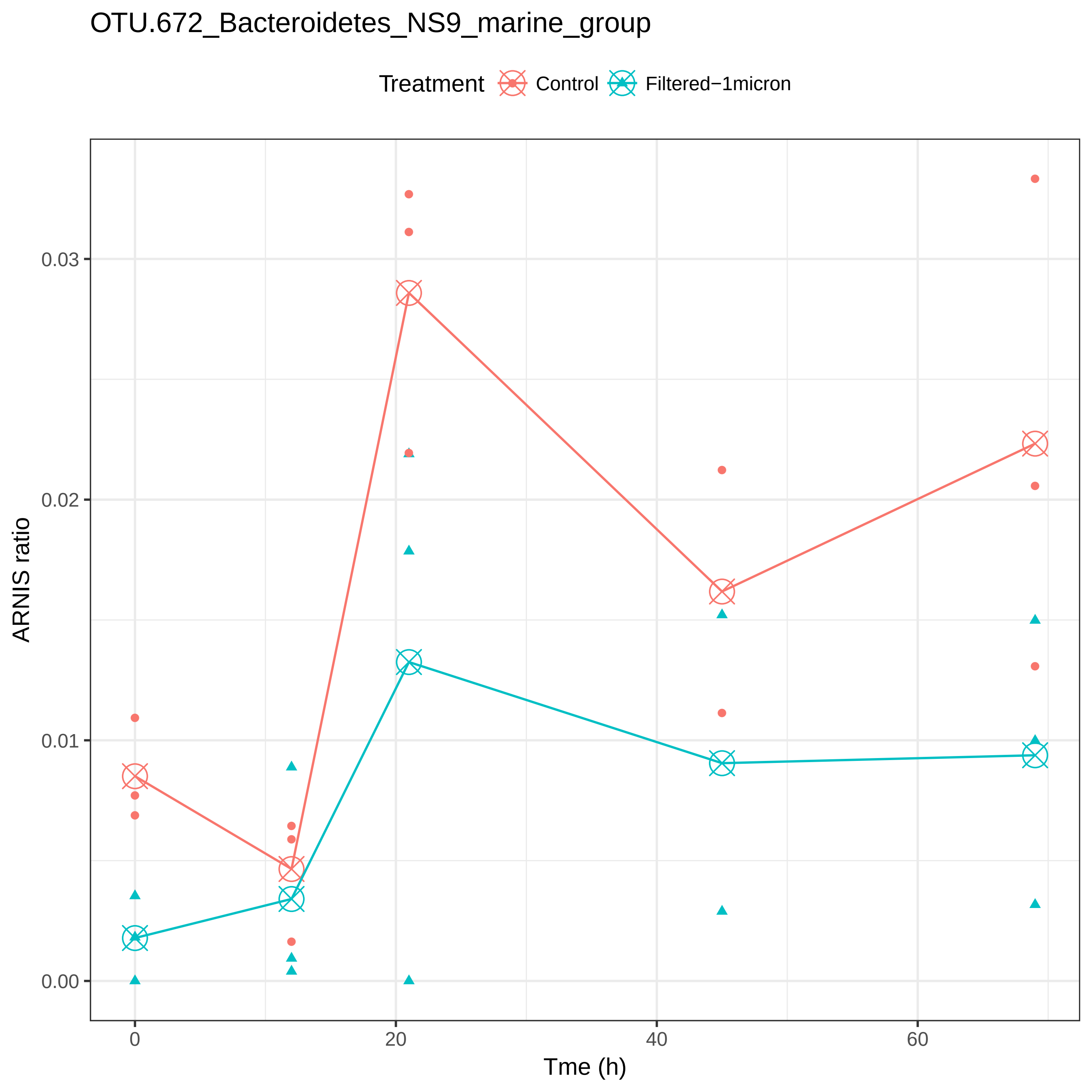

OTU.672_Bacteroidetes_NS9_marine_group
Treatment
Control
Filtered−1micron
ARNIS ratio
0.00
0.01
0.02
0.03
0
20
40
60
Tme (h)

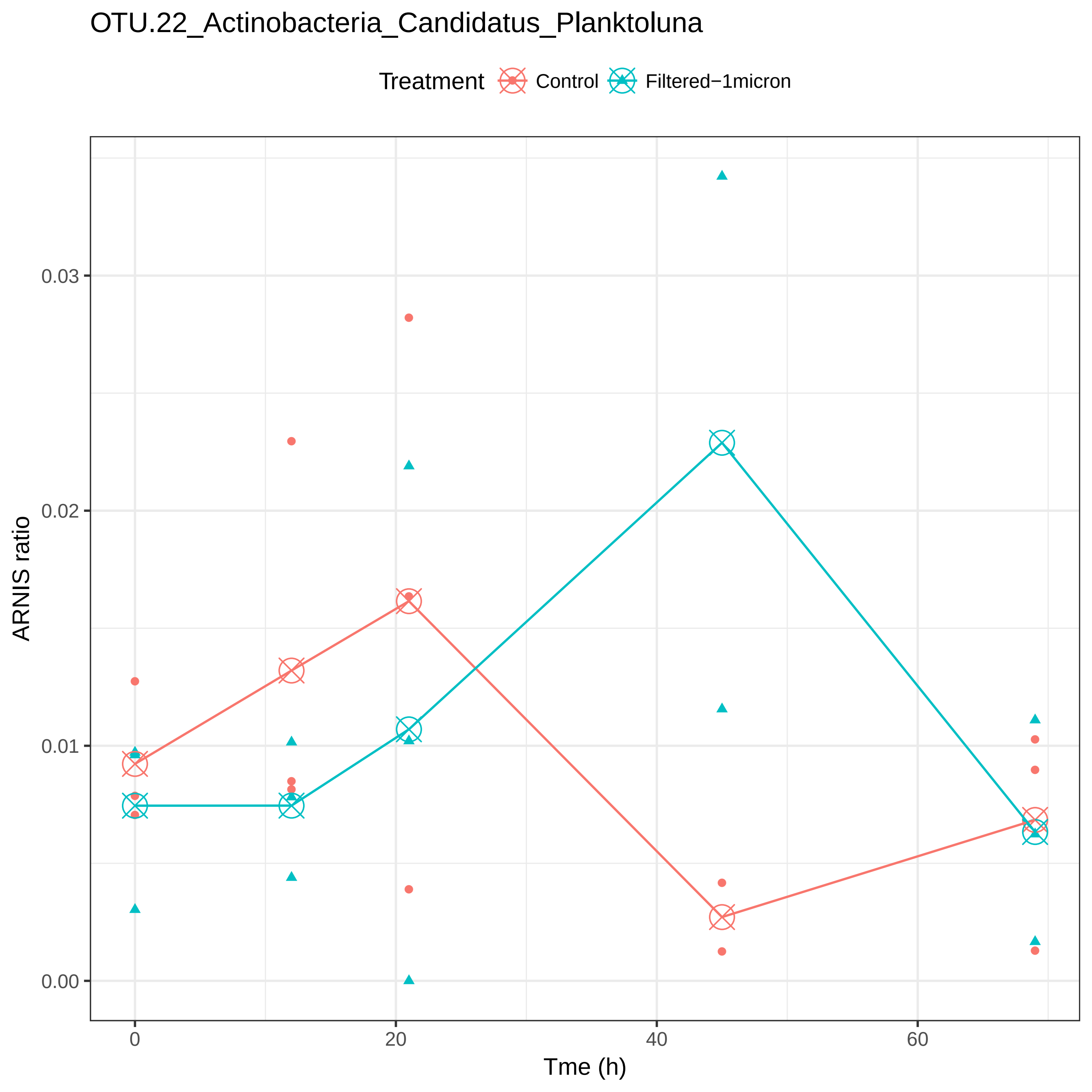

OTU.22_Actinobacteria_Candidatus_Planktoluna
Treatment
Control
Filtered−1micron
0.03
0.02
0.01
0.00
ARNIS ratio
0
20
40
60
Tme (h)

OTU.418_Firmicutes_Lactococcus

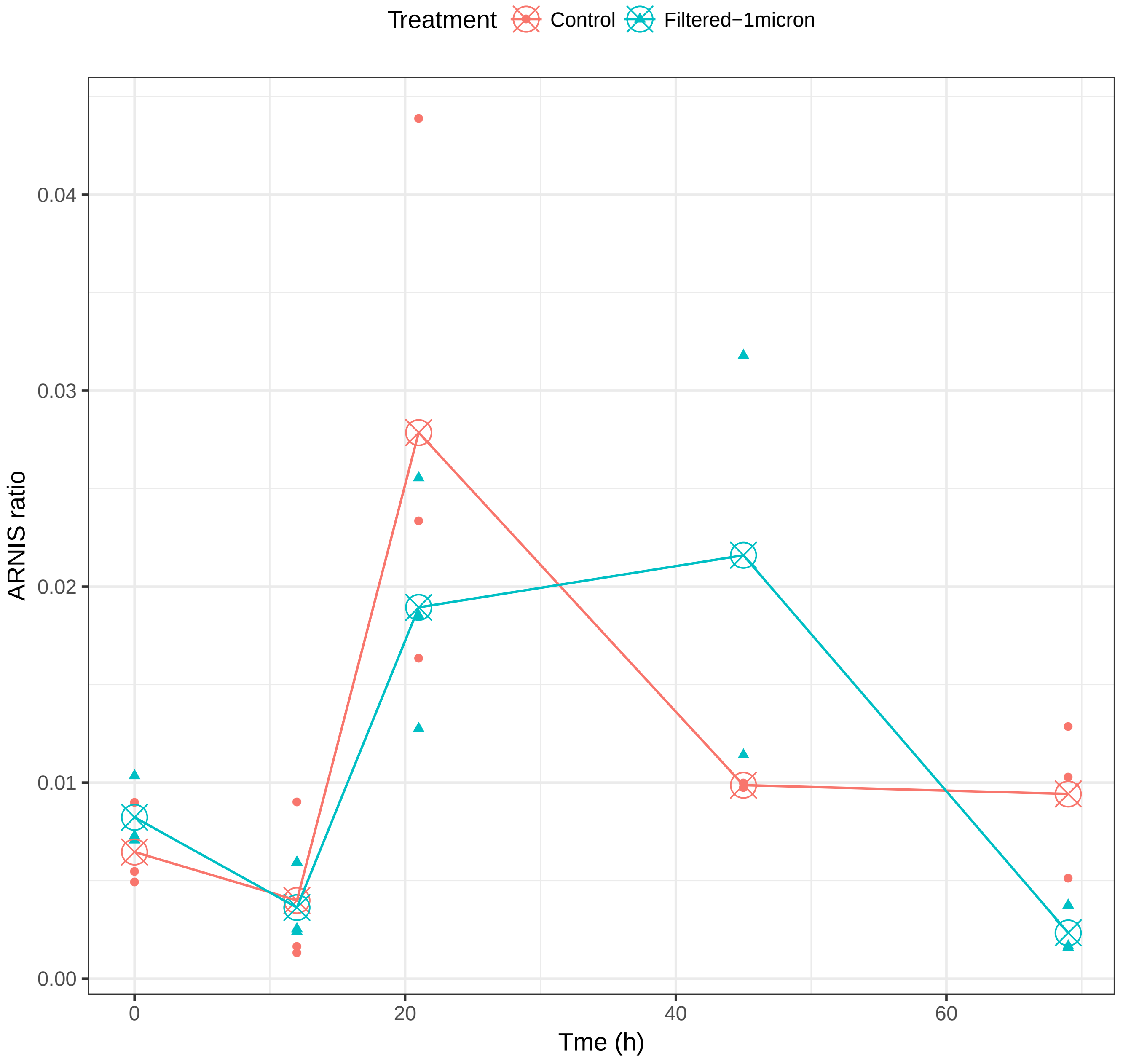

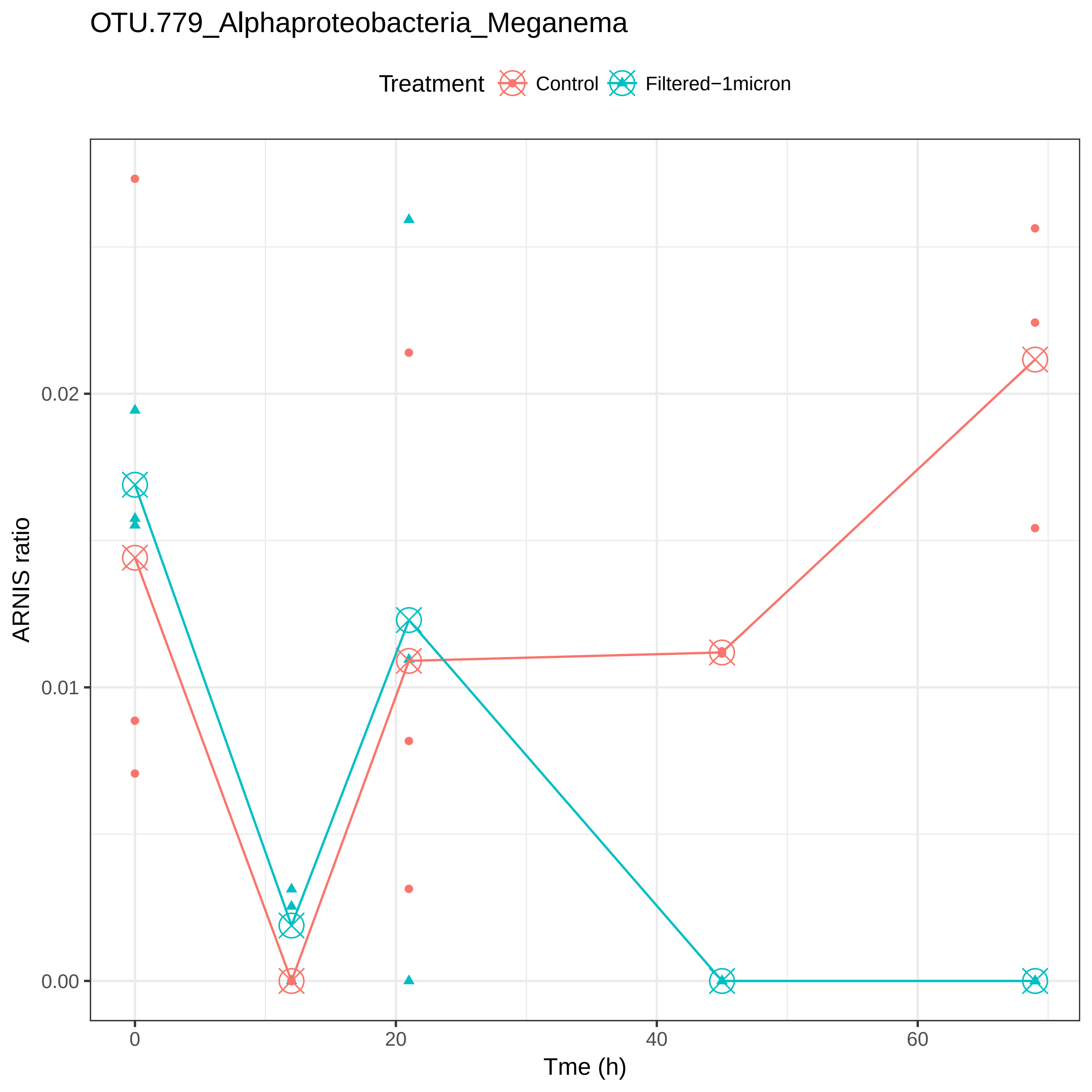
OTU.779_Alphaproteobacteria_Meganema
Treatment
Control
Filtered−1micron
ARNIS ratio
0.00
0.01
0.02
0
20
40
60
Tme (h)

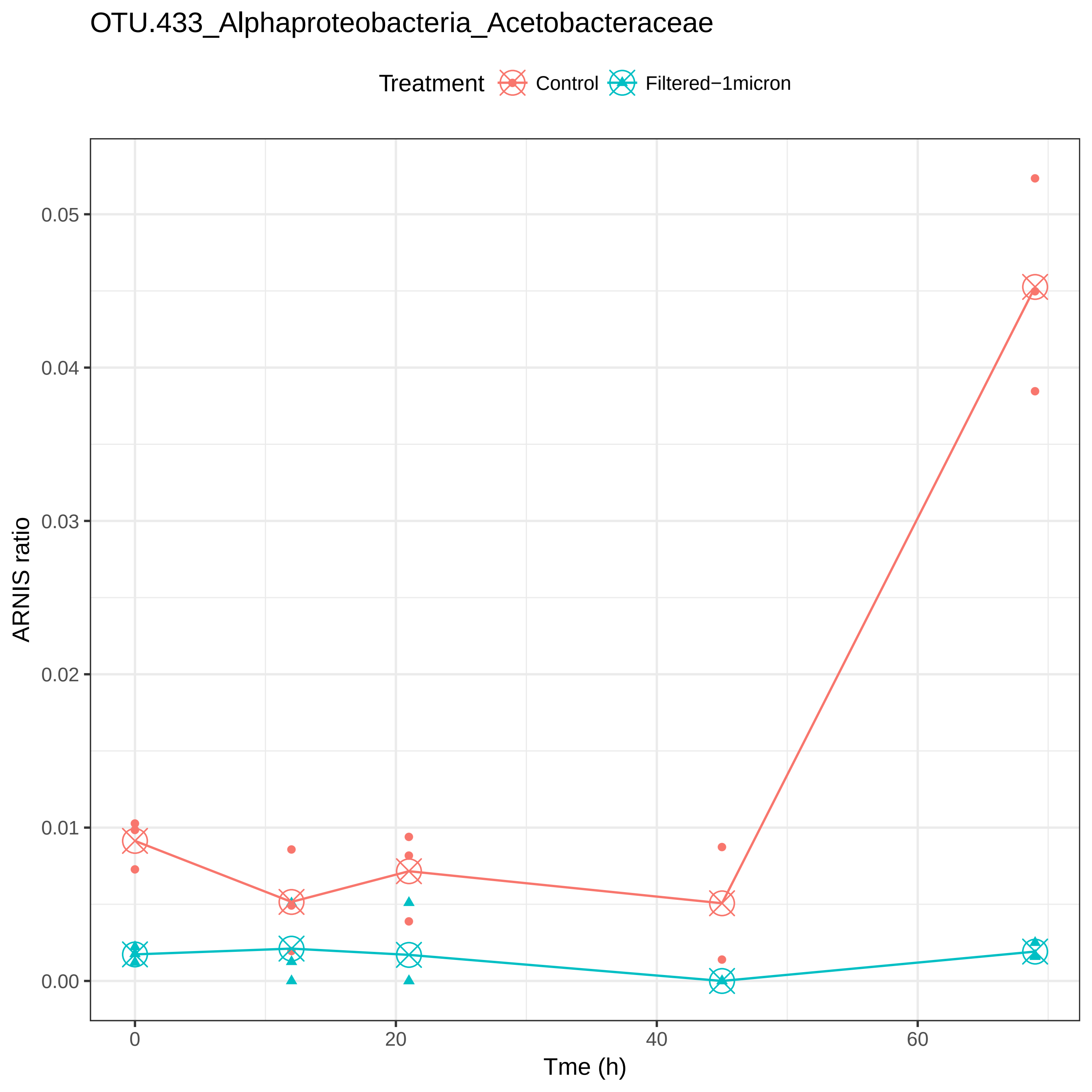
OTU.433_Alphaproteobacteria_Acetobacteraceae
Treatment
Control
Filtered−1micron
ARNIS ratio
0.05
0.04
0.03
0.02
0.01
0.00
0
20
40
60
Tme (h)

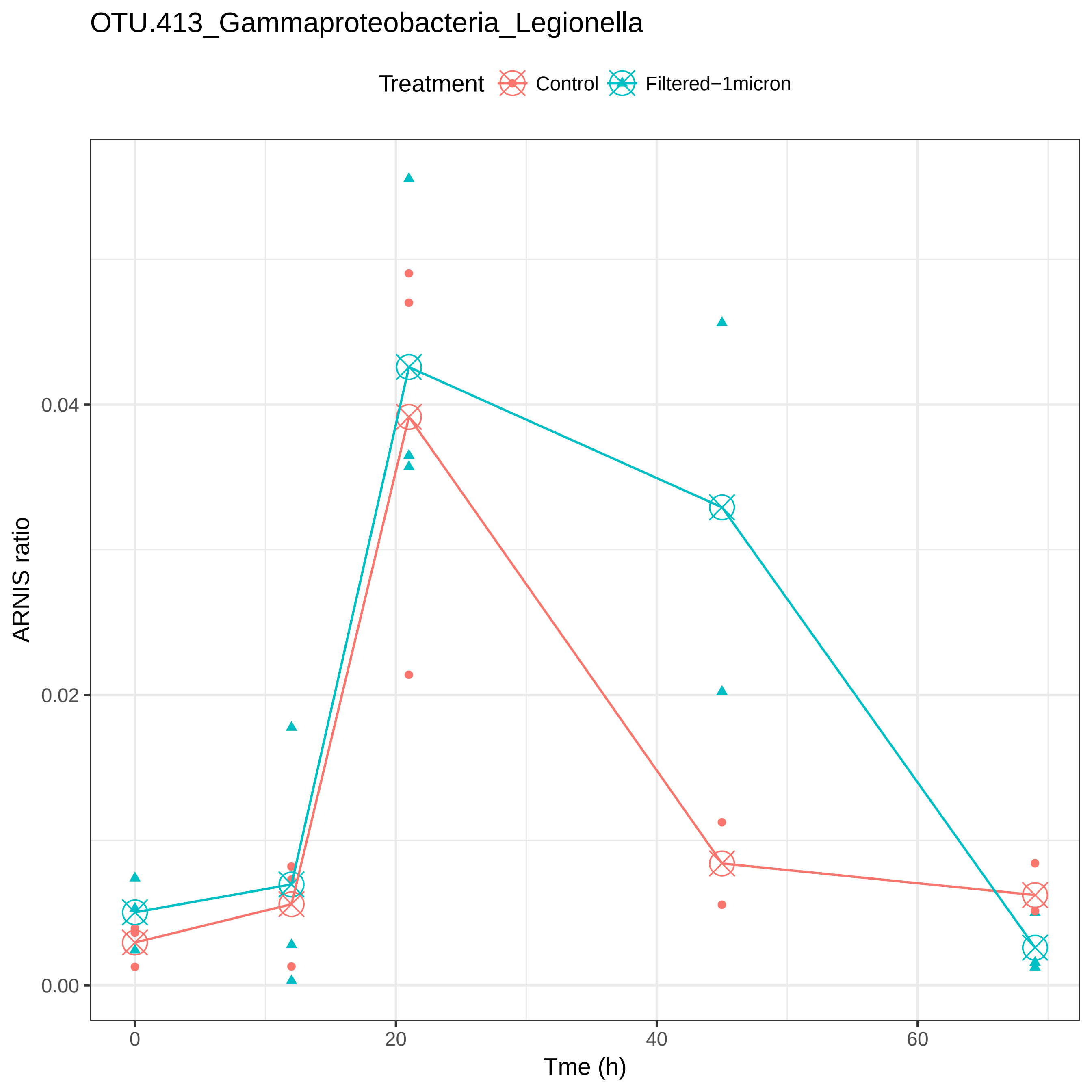

OTU.413_Gammaproteobacteria_Legionella
Treatment
Control
Filtered−1micron
ARNIS ratio
0.00
0.02
0.04
0
20
40
60
Tme (h)

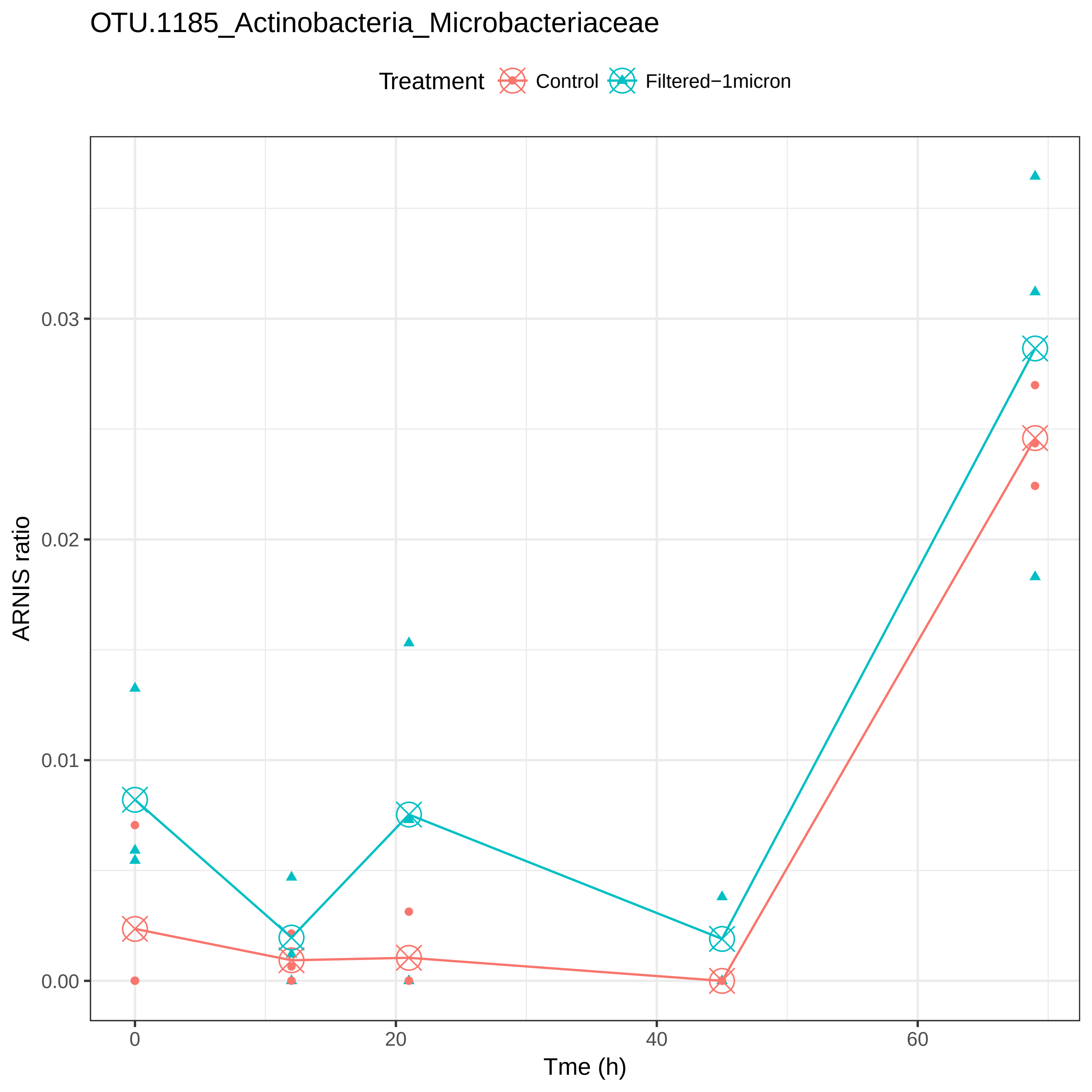

OTU.1185_Actinobacteria_Microbacteriaceae
Treatment
Control
Filtered−1micron
ARNIS ratio
0.00
0.01
0.02
0.03
0
20
40
60
Tme (h)

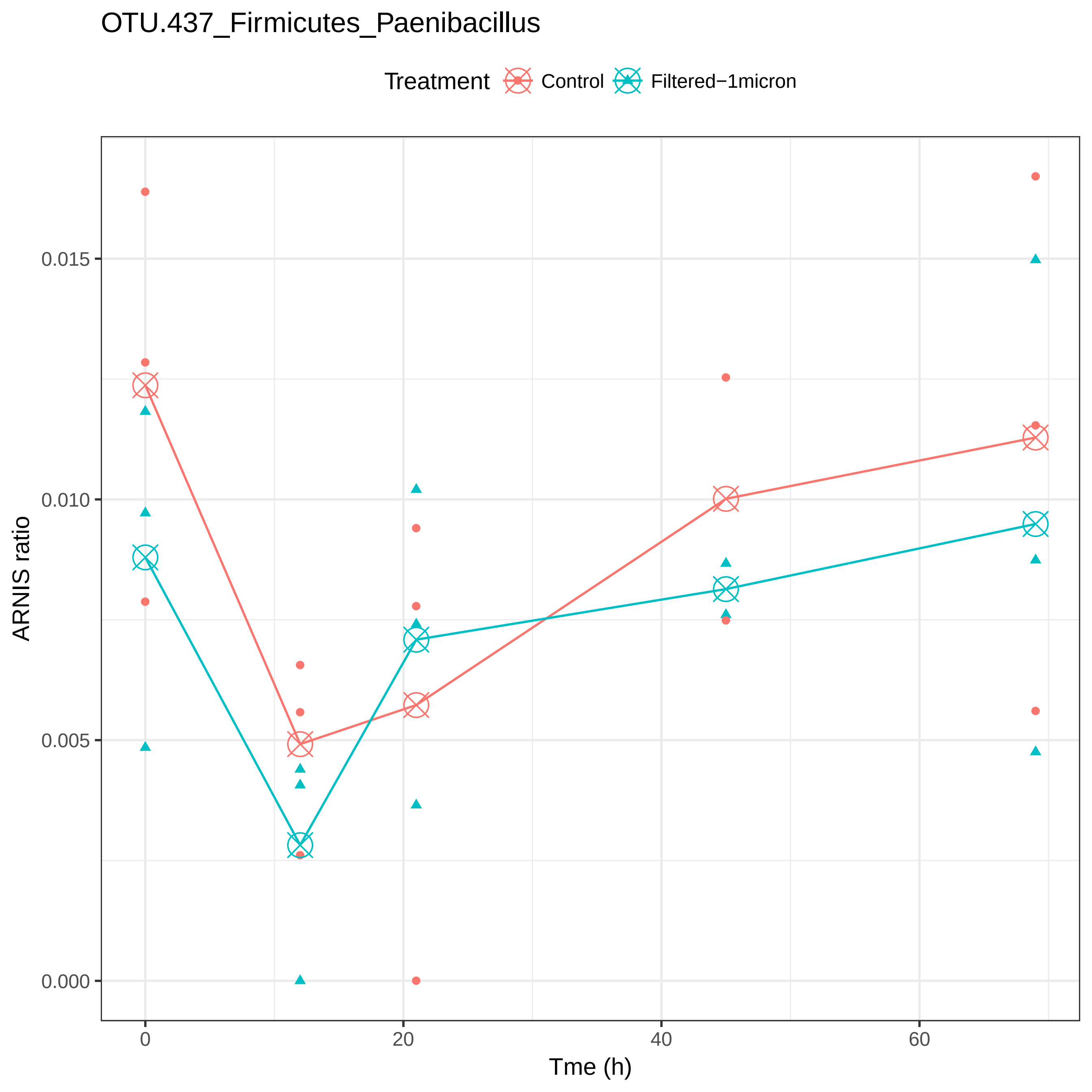
OTU.437_Firmicutes_Paenibacillus
Treatment
Control
Filtered−1micron
ARNIS ratio
0.000
0.005
0.010
0.015
0
20
40
60
Tme (h)

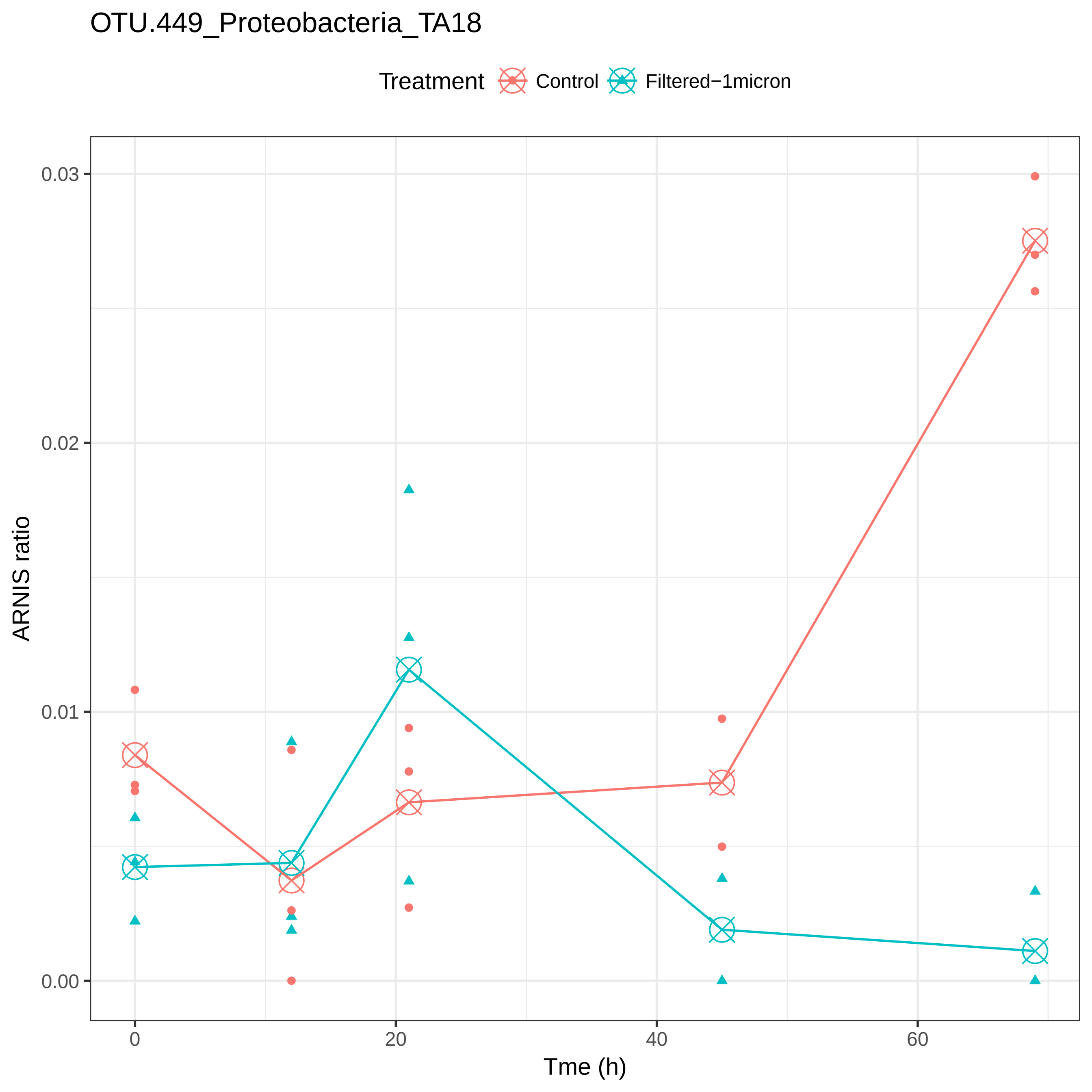

OTU.449_Proteobacteria_TA18
Treatment
Control
Filtered−1micron
0.03
0.02
0.01
0.00
0
20
40
60
ARNIS ratio
Tme (h)

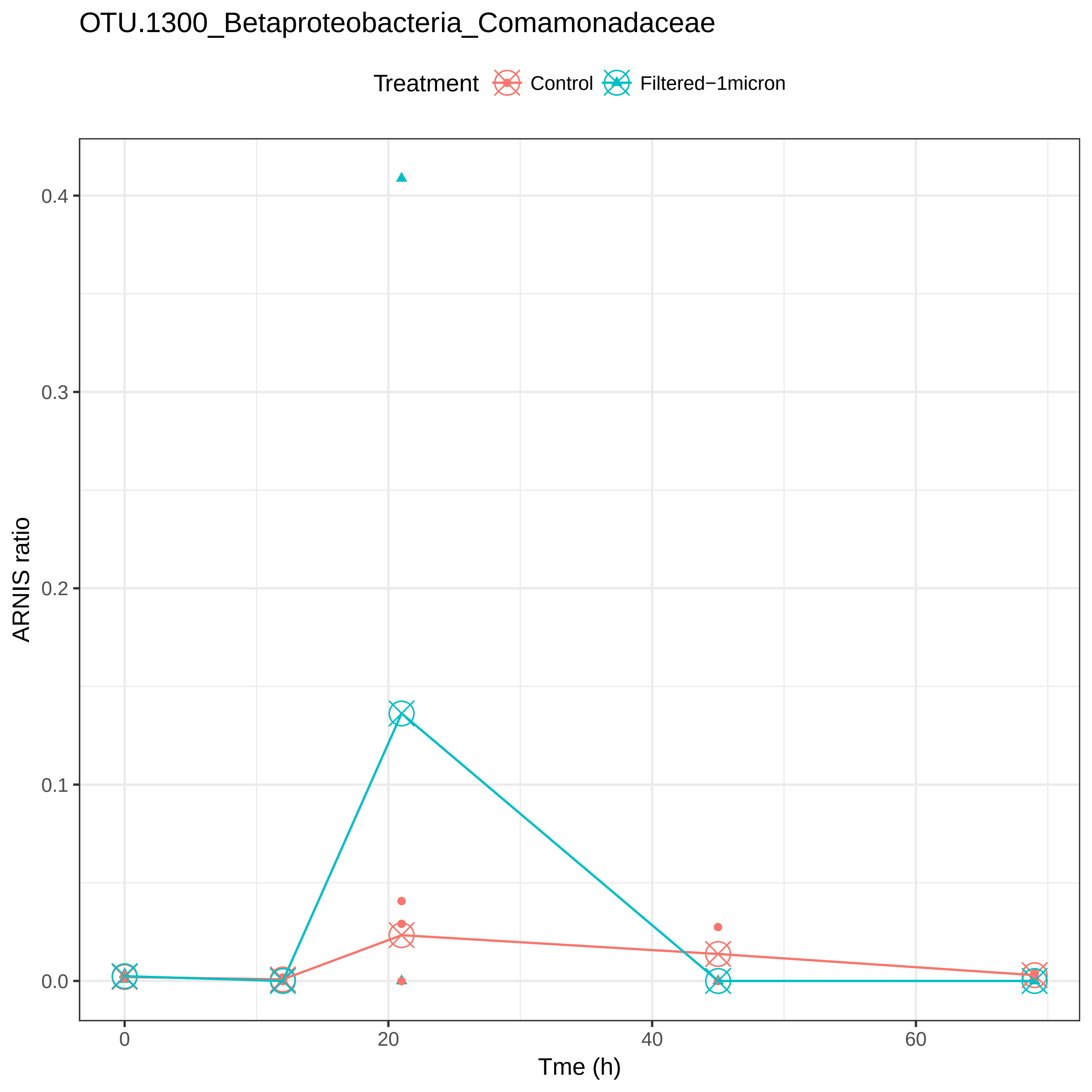

OTU.1300_Betaproteobacteria_Comamonadaceae
Treatment
Control
Filtered−1micron
ARNIS ratio
0.0
0.1
0.2
0.3
0.4
0
20
40
60
Tme (h)

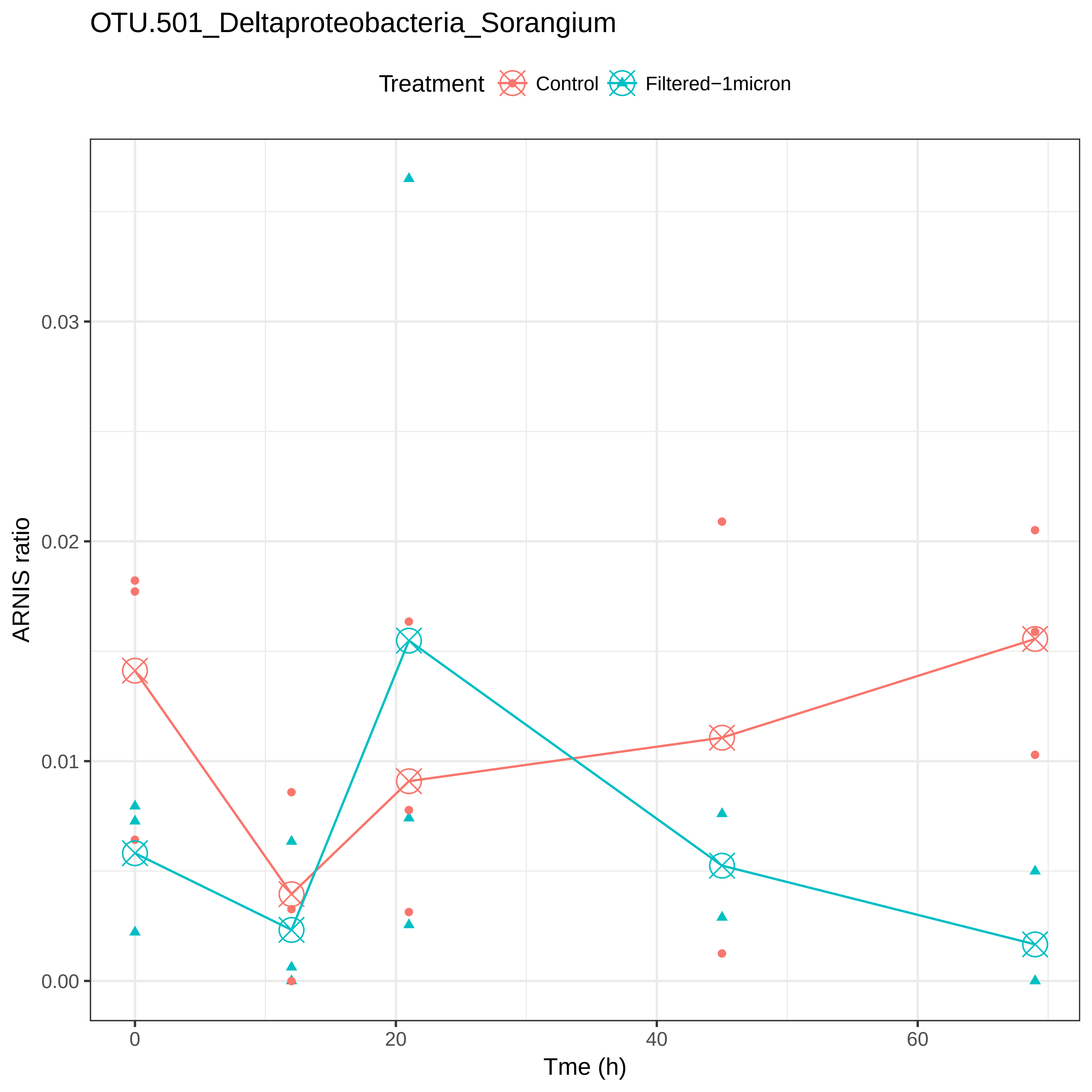

OTU.501_Deltaproteobacteria_Sorangium
Treatment
Control
Filtered−1micron
ARNIS ratio
0.00
0.01
0.02
0.03
0
20
40
60
Tme (h)

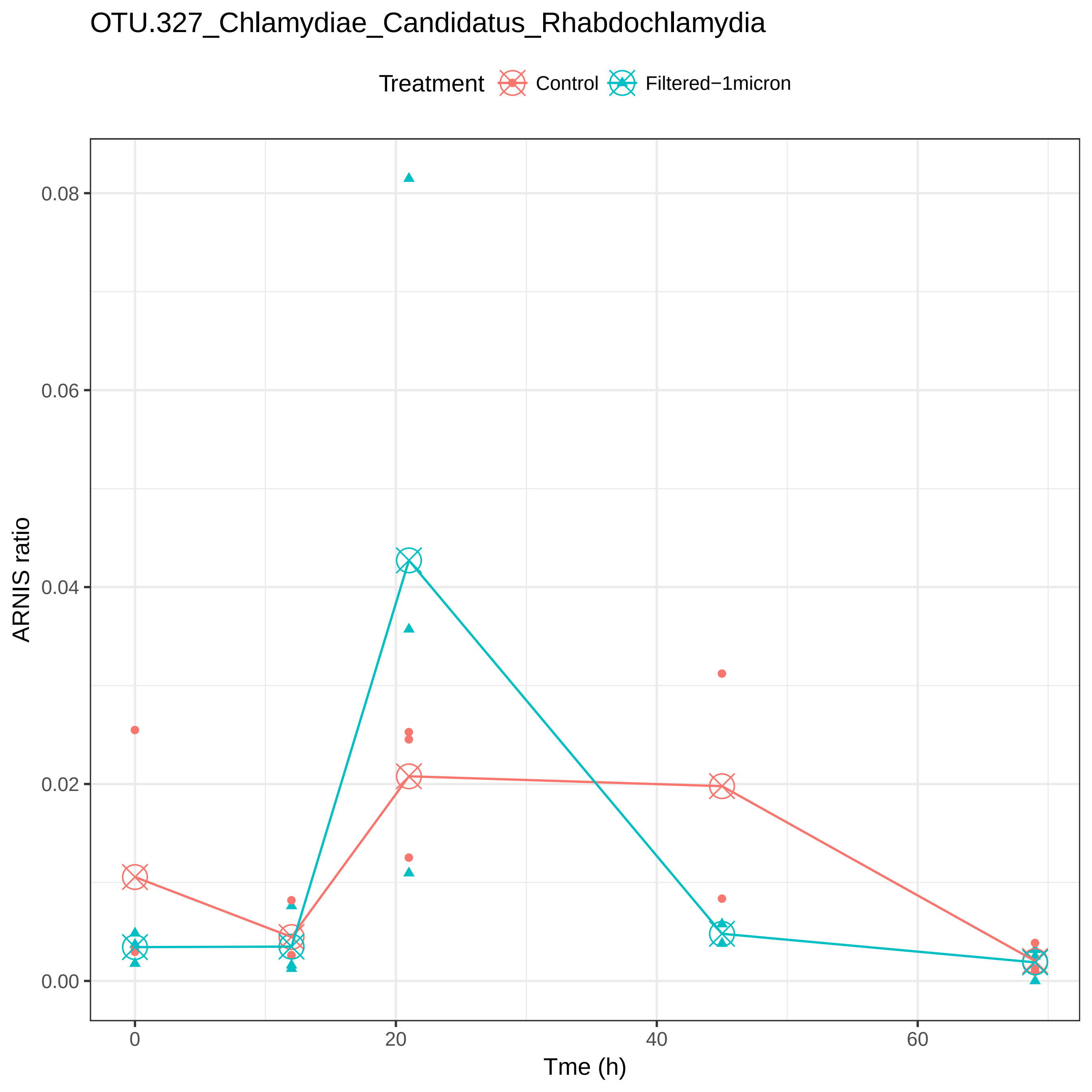
OTU.327_Chlamydiae_Candidatus_Rhabdochlamydia
Treatment
Control
Filtered−1micron
ARNIS ratio
0.08
0.06
0.04
0.02
0.00
0
20
40
60
Tme (h)

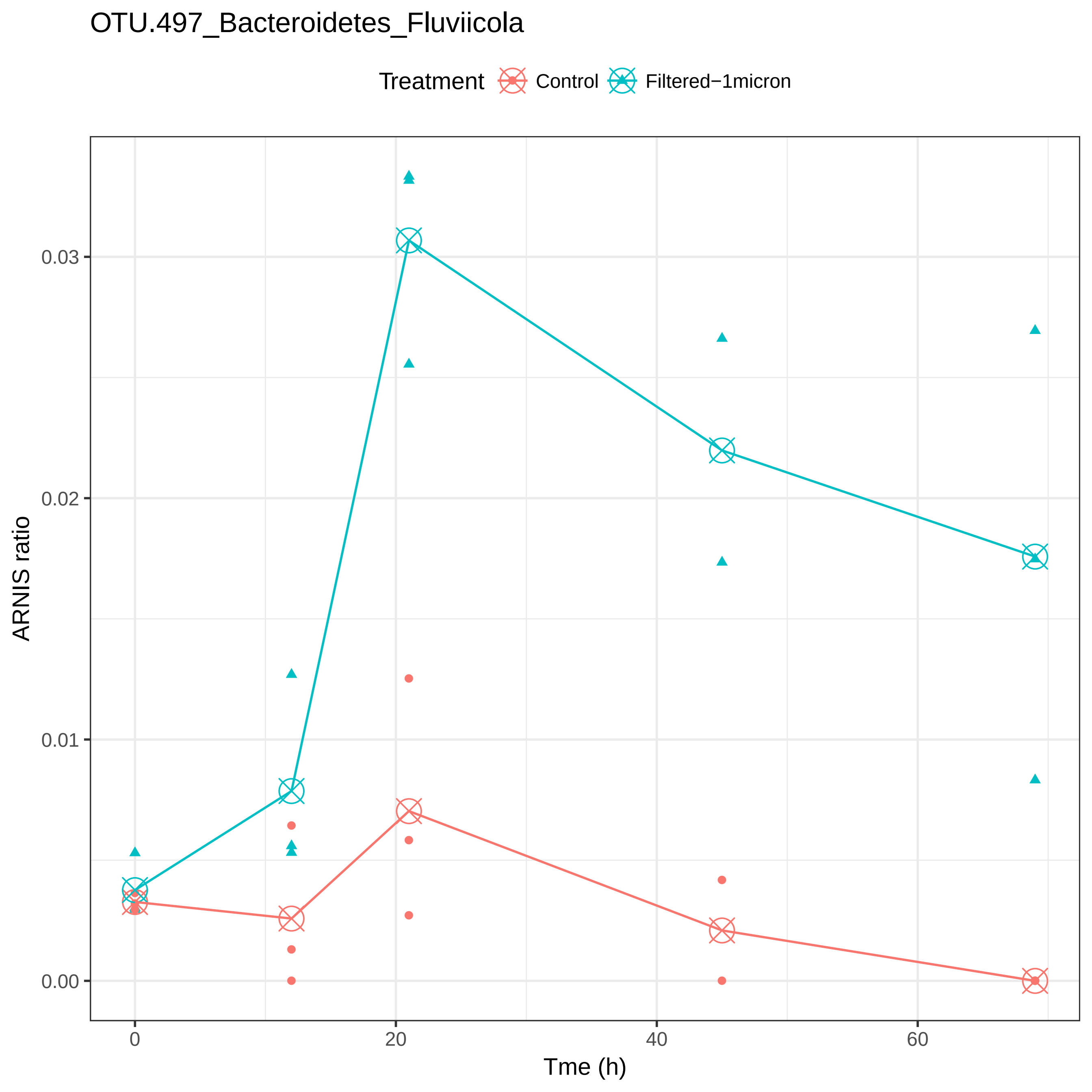

OTU.497_Bacteroidetes_Fluviicola
Treatment
Control
Filtered−1micron
ARNIS ratio
0.00
0.01
0.02
0.03
0
20
40
60
Tme (h)

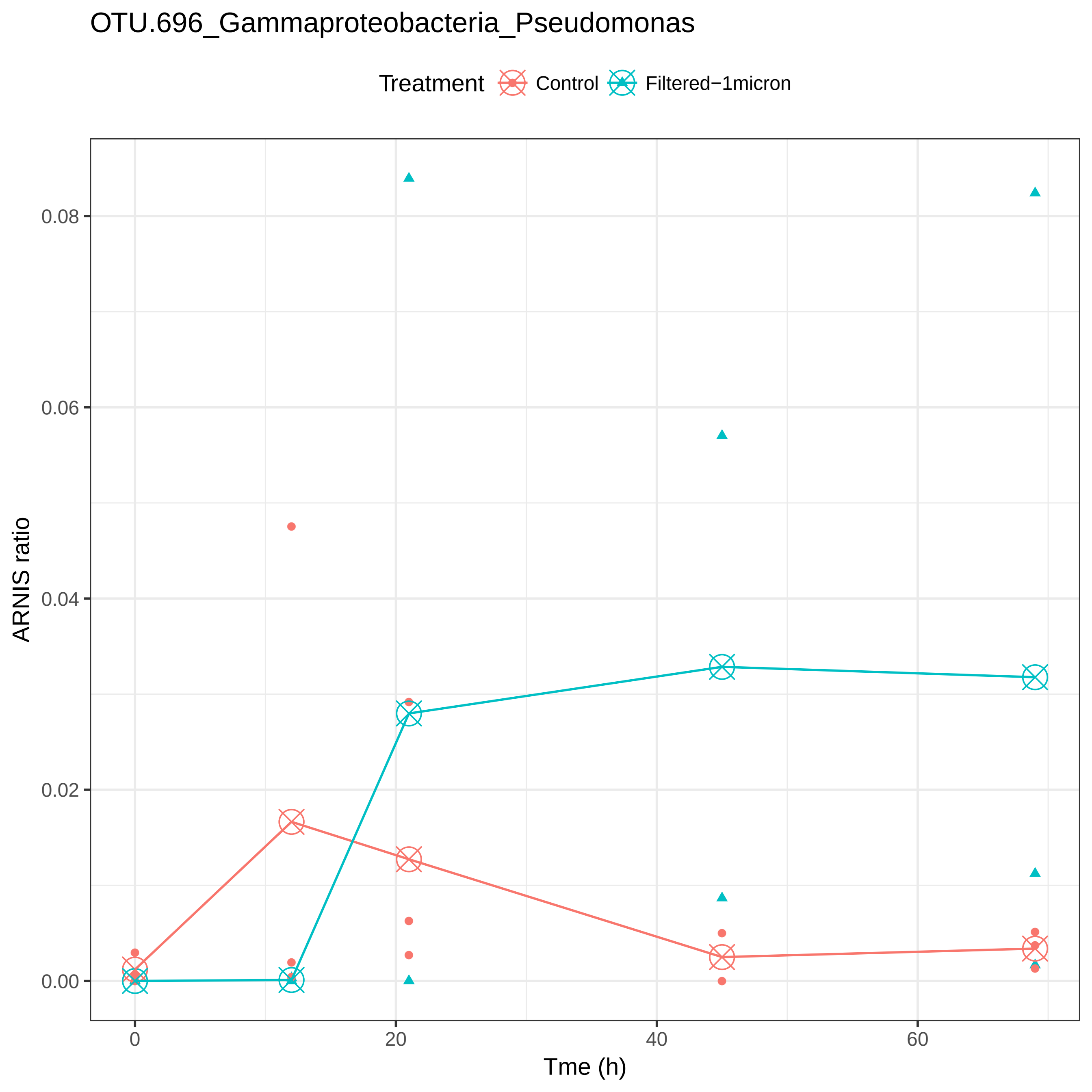
OTU.696_Gammaproteobacteria_Pseudomonas
Treatment
Control
Filtered−1micron
ARNIS ratio
0.00
0.02
0.04
0.06
0.08
0
20
40
60
Tme (h)

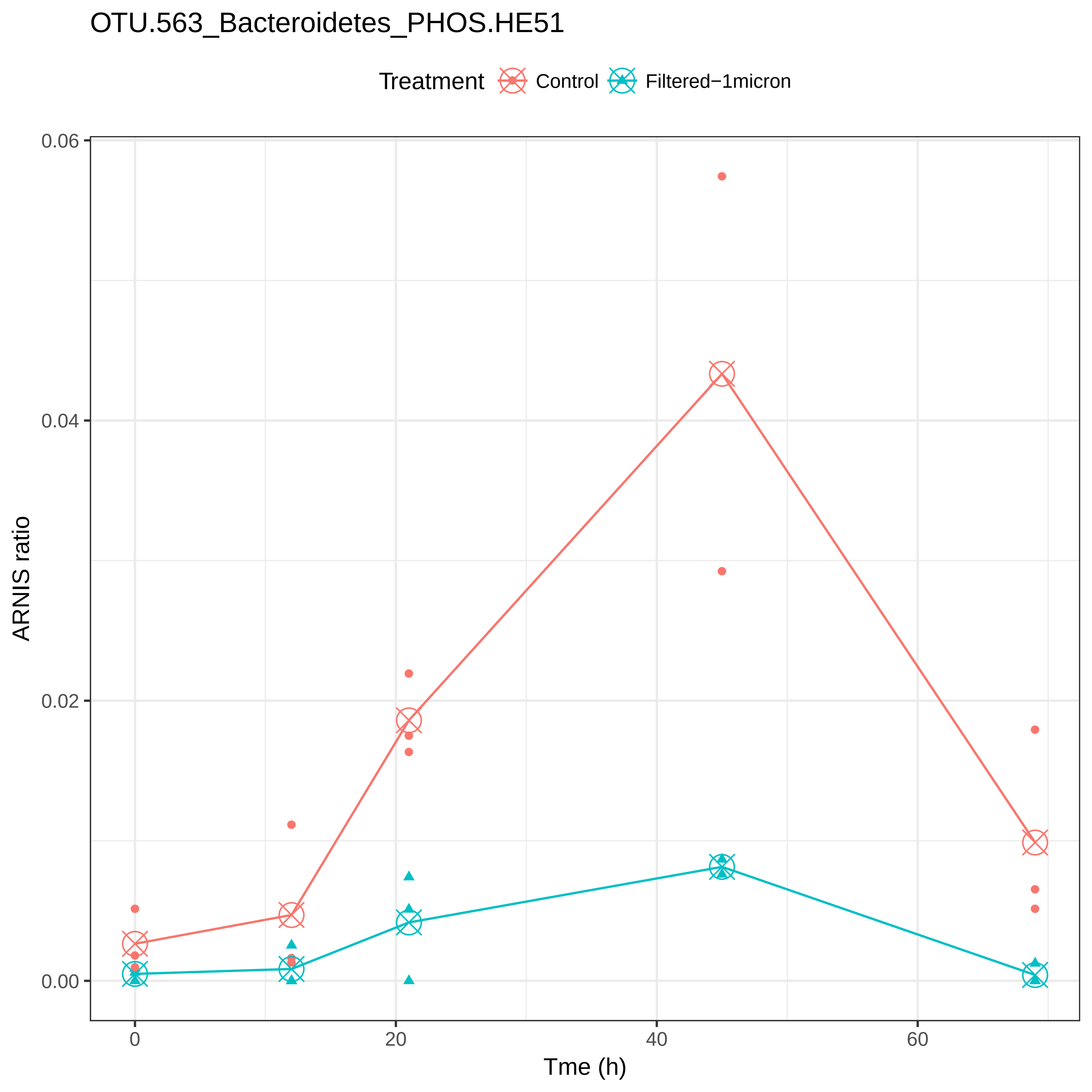

OTU.563_Bacteroidetes_PHOS.HE51
Treatment
Control
Filtered−1micron
0.06
0.04
0.02
0.00
ARNIS ratio
0
20
40
60
Tme (h)

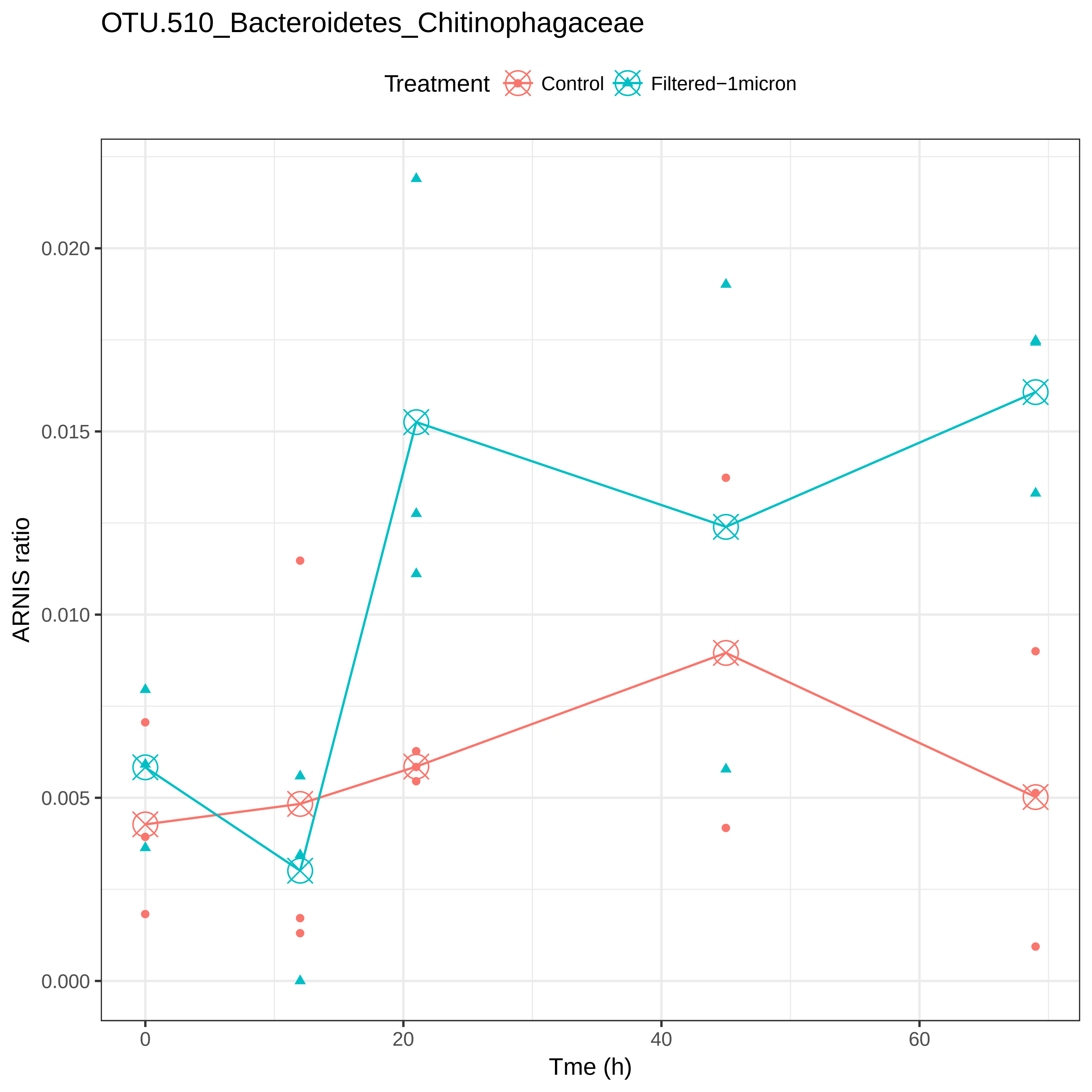

OTU.510_Bacteroidetes_Chitinophagaceae
Treatment
Control
Filtered−1micron
ARNIS ratio
0.000
0.005
0.010
0.015
0.020
0
20
40
60
Tme (h)

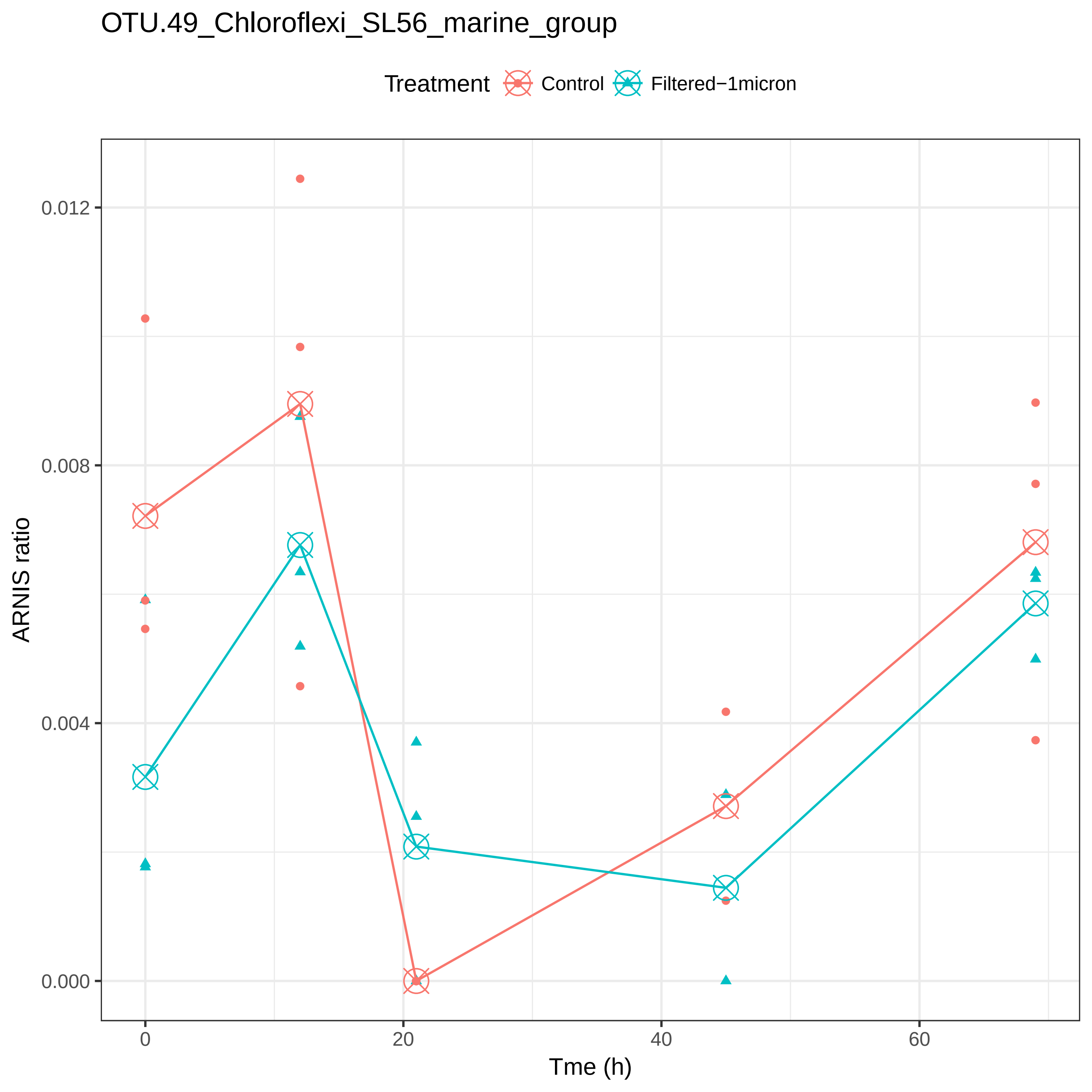
OTU.49_Chloroflexi_SL56_marine_group
Treatment
Control
Filtered−1micron
ARNIS ratio
0.012
0.008
0.004
0.000
0
20
40
60
Tme (h)

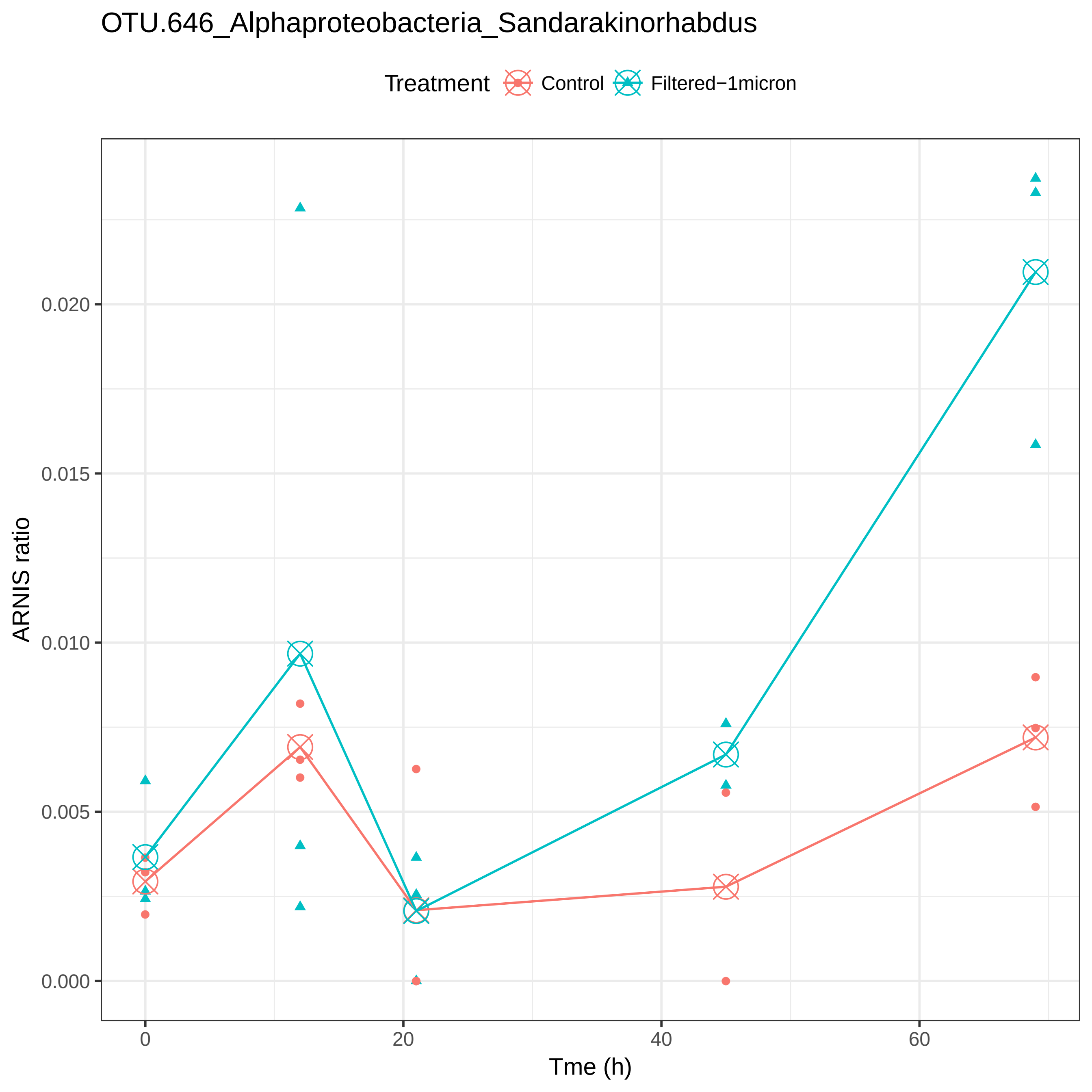
OTU.646_Alphaproteobacteria_Sandarakinorhabdus
Treatment
Control
Filtered−1micron
ARNIS ratio
0.000
0.005
0.010
0.015
0.020
0
20
40
60
Tme (h)

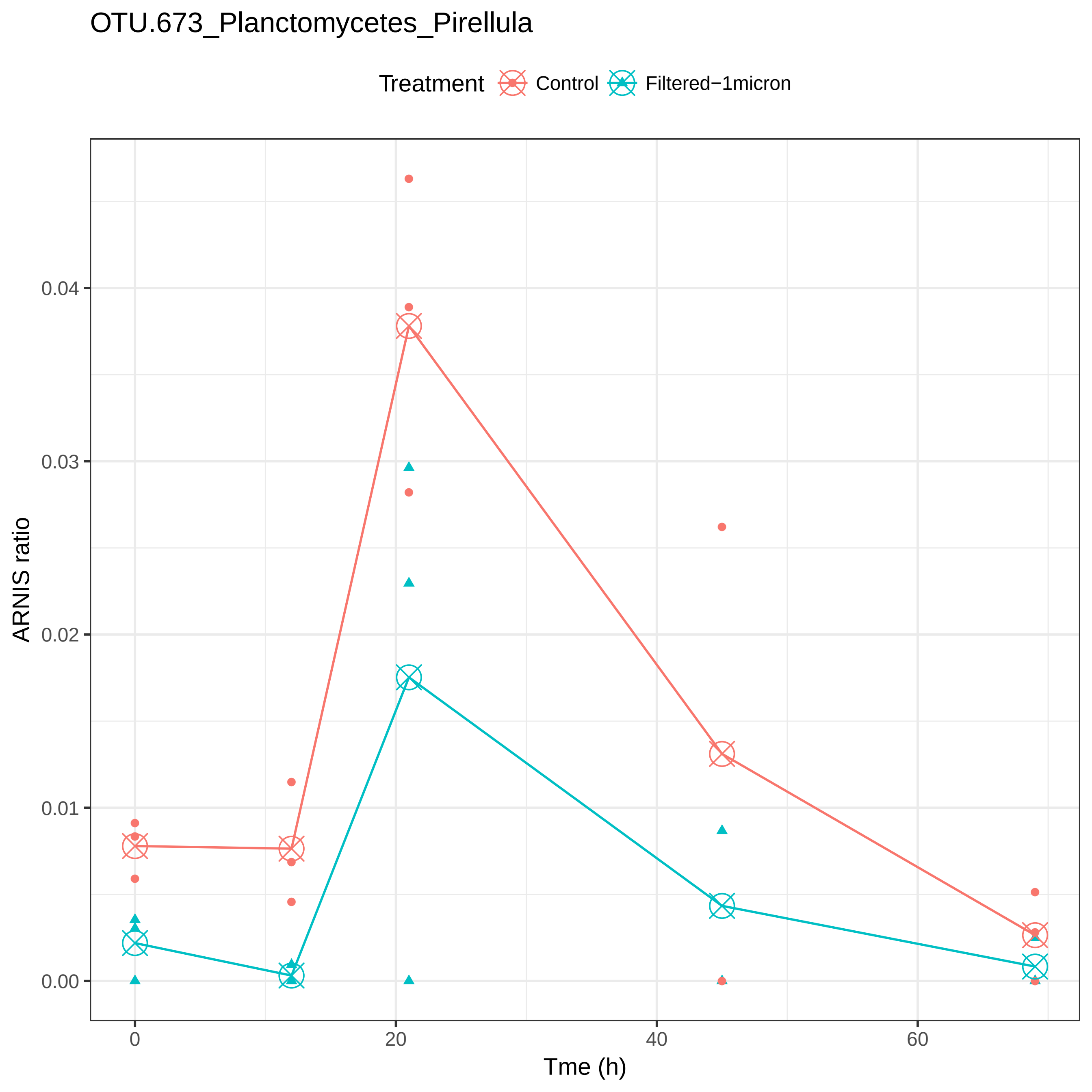

OTU.673_Planctomycetes_Pirellula
Treatment
Control
Filtered−1micron
ARNIS ratio
0.00
0.01
0.02
0.03
0.04
0
20
40
60
Tme (h)

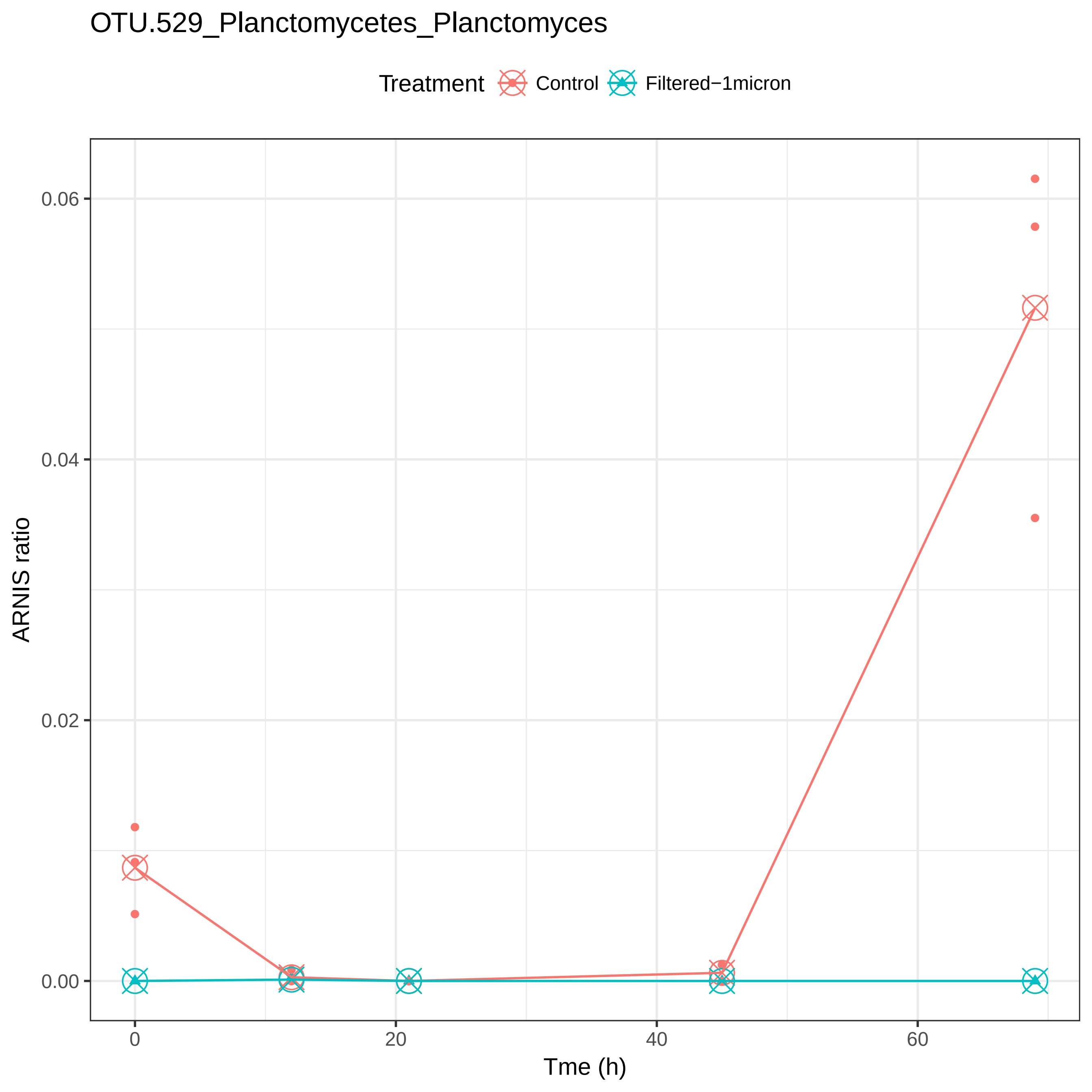

OTU.529_Planctomycetes_Planctomyces
Treatment
Control
Filtered−1micron
ARNIS ratio
0.06
0.04
0.02
0.00
0
20
40
60
Tme (h)

OTU.249_Bacteroidetes_Chitinophagaceae

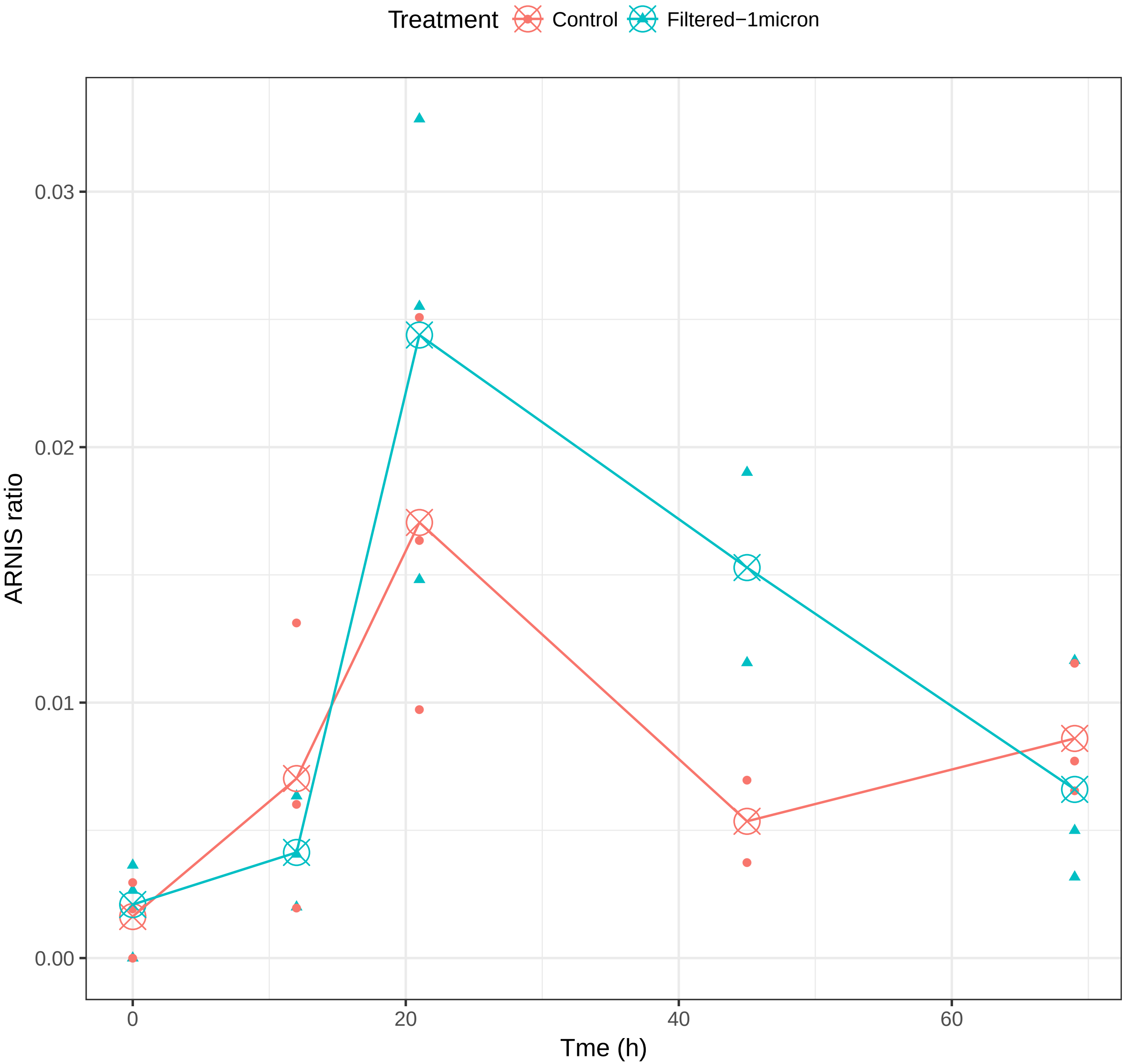

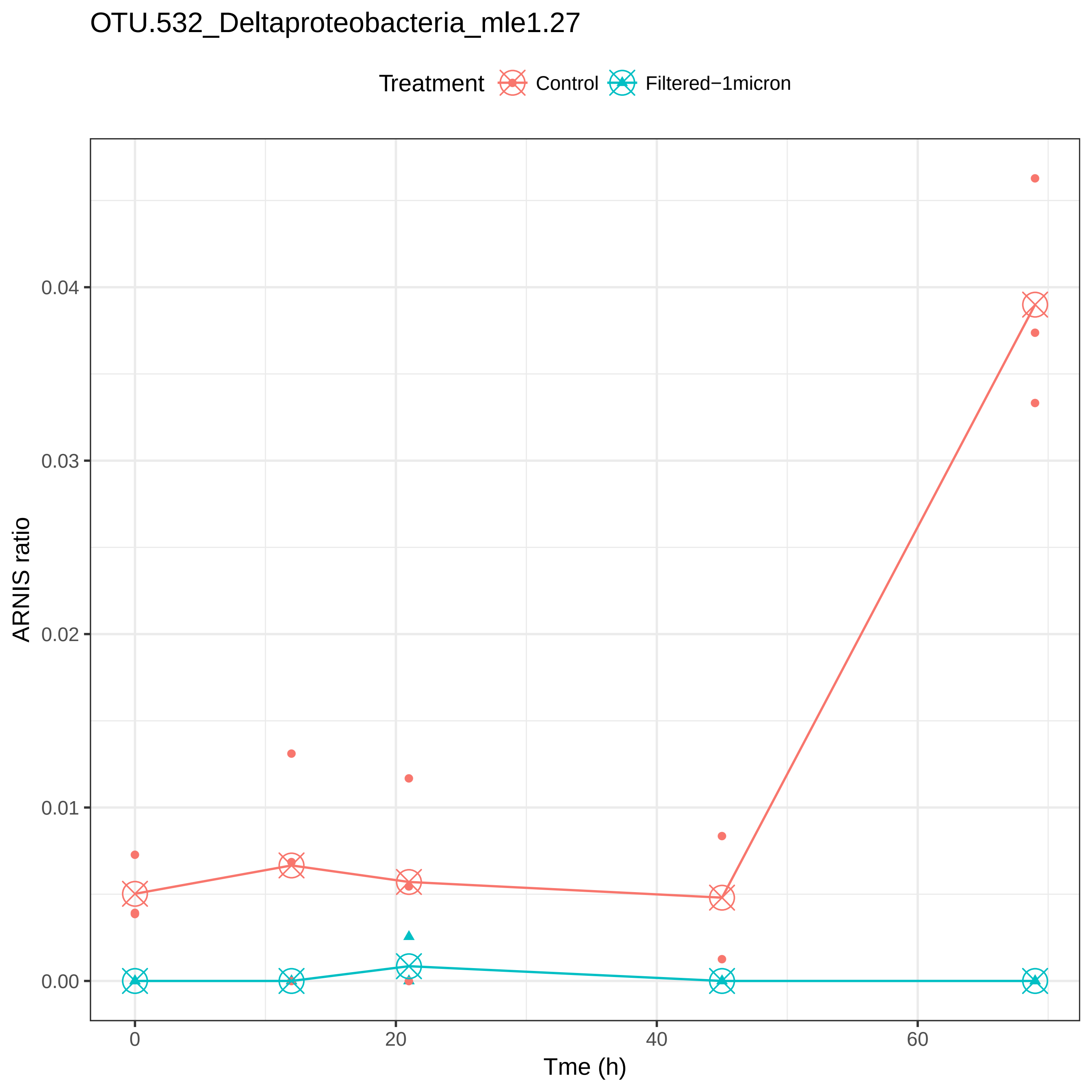

OTU.532_Deltaproteobacteria_mle1.27
Treatment
Control
Filtered−1micron
ARNIS ratio
0.00
0.01
0.02
0.03
0.04
0
20
40
60
Tme (h)

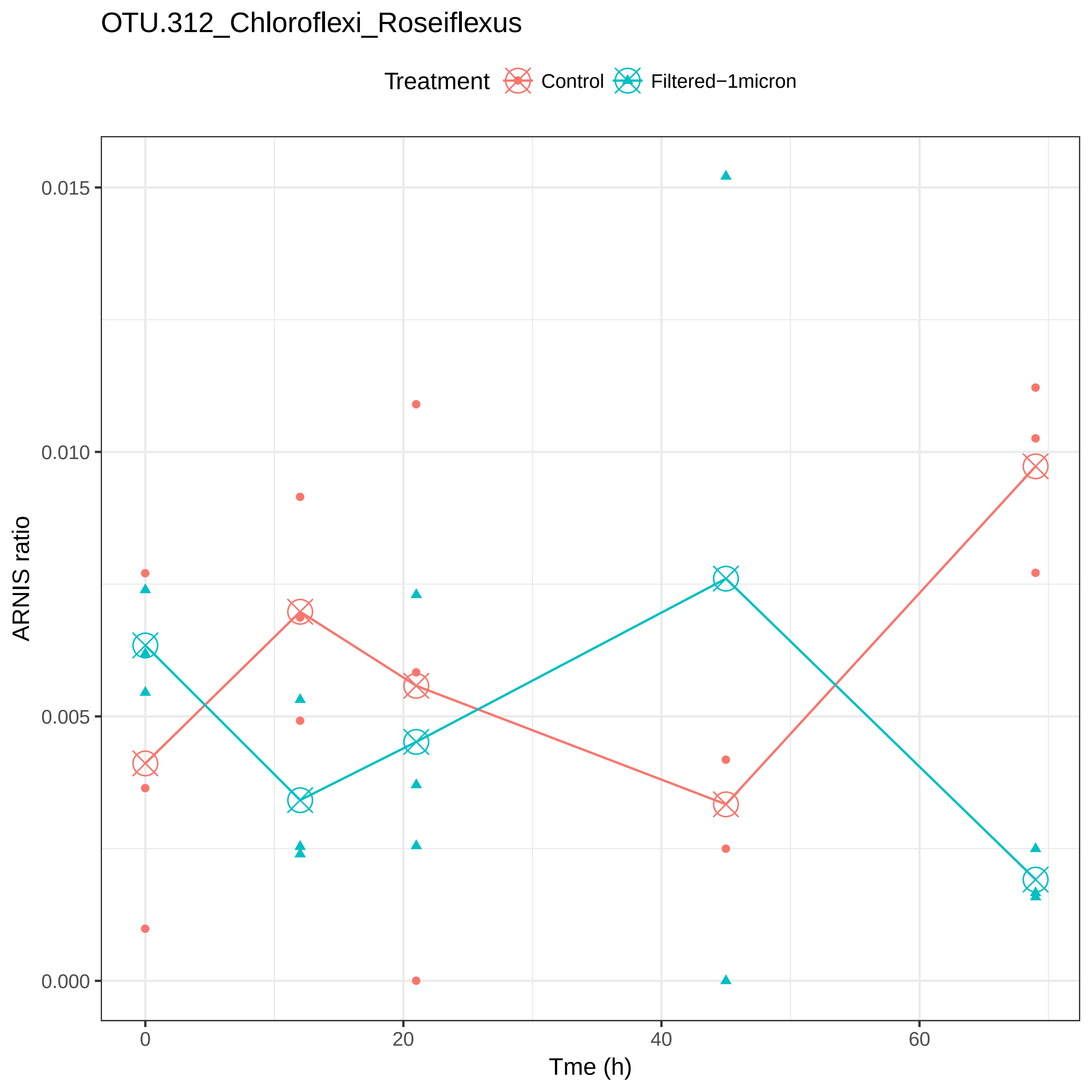
OTU.312_Chloroflexi_Roseiflexus
Treatment
Control
Filtered−1micron
ARNIS ratio
0.015
0.010
0.005
0.000
0
20
40
60
Tme (h)

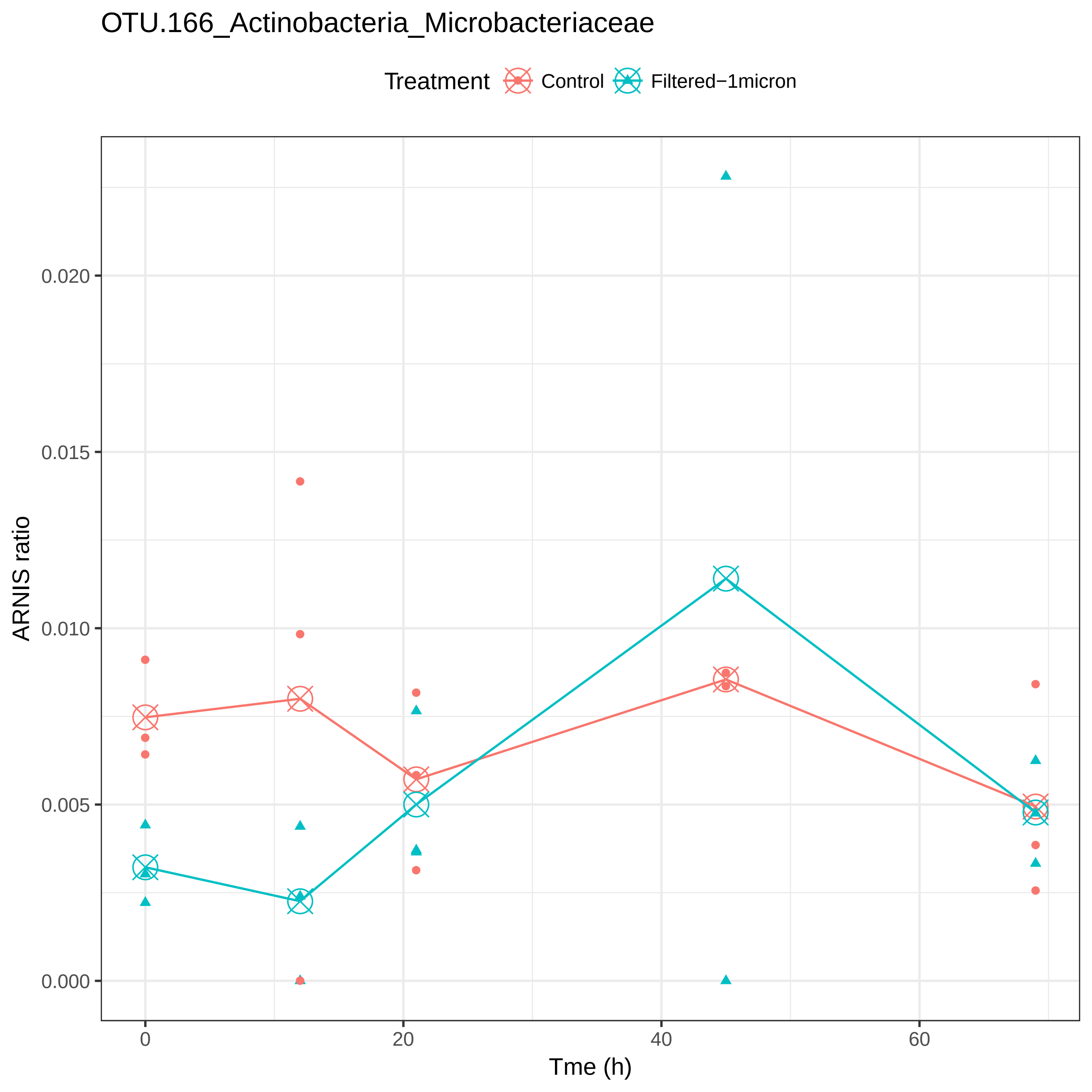
OTU.166_Actinobacteria_Microbacteriaceae
Treatment
Control
Filtered−1micron
ARNIS ratio
0.000
0.005
0.010
0.015
0.020
0
20
40
60
Tme (h)

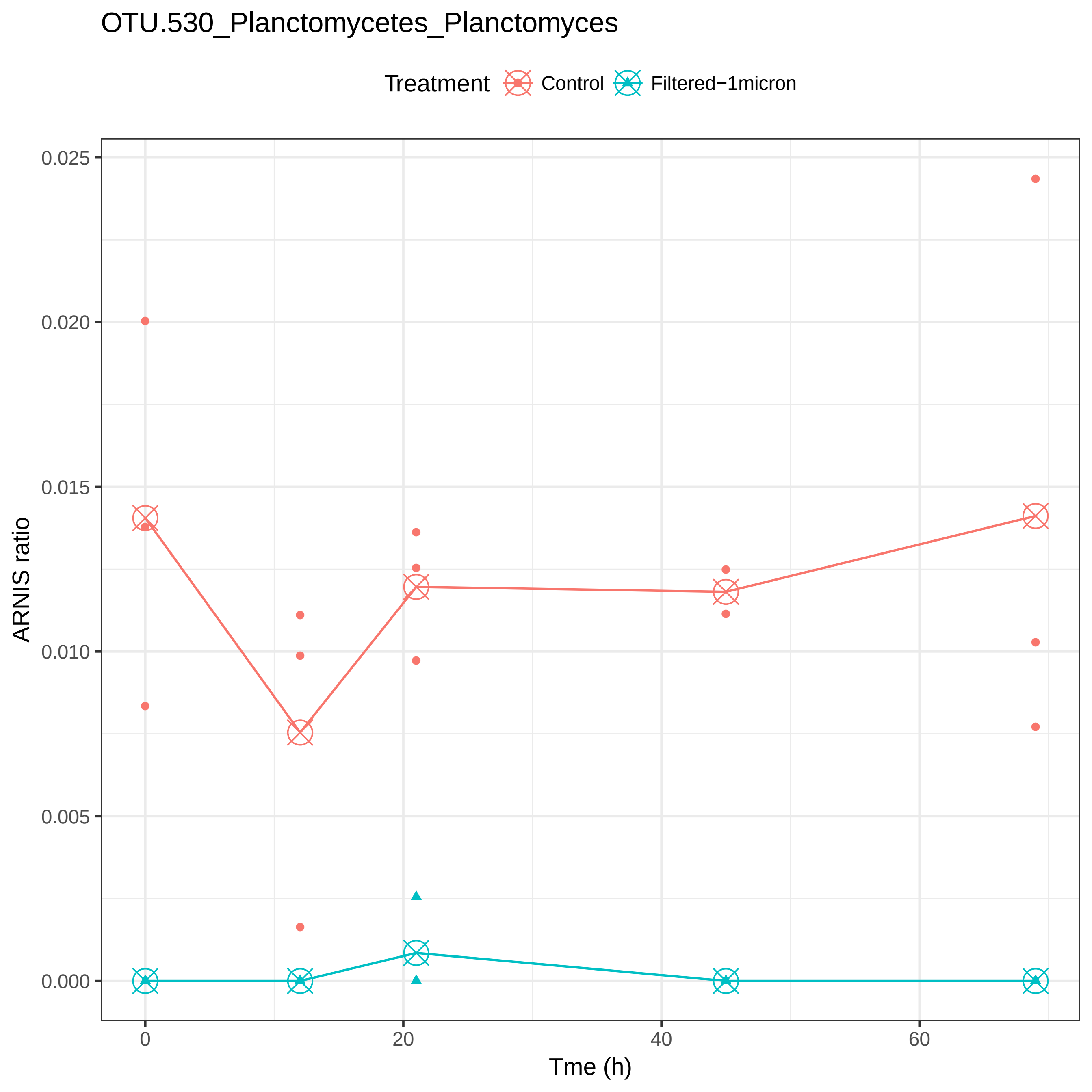
OTU.530_Planctomycetes_Planctomyces
Treatment
Control
Filtered−1micron
0.025
0.020
0.015
0.010
0.005
0.000
ARNIS ratio
0
20
40
60
Tme (h)

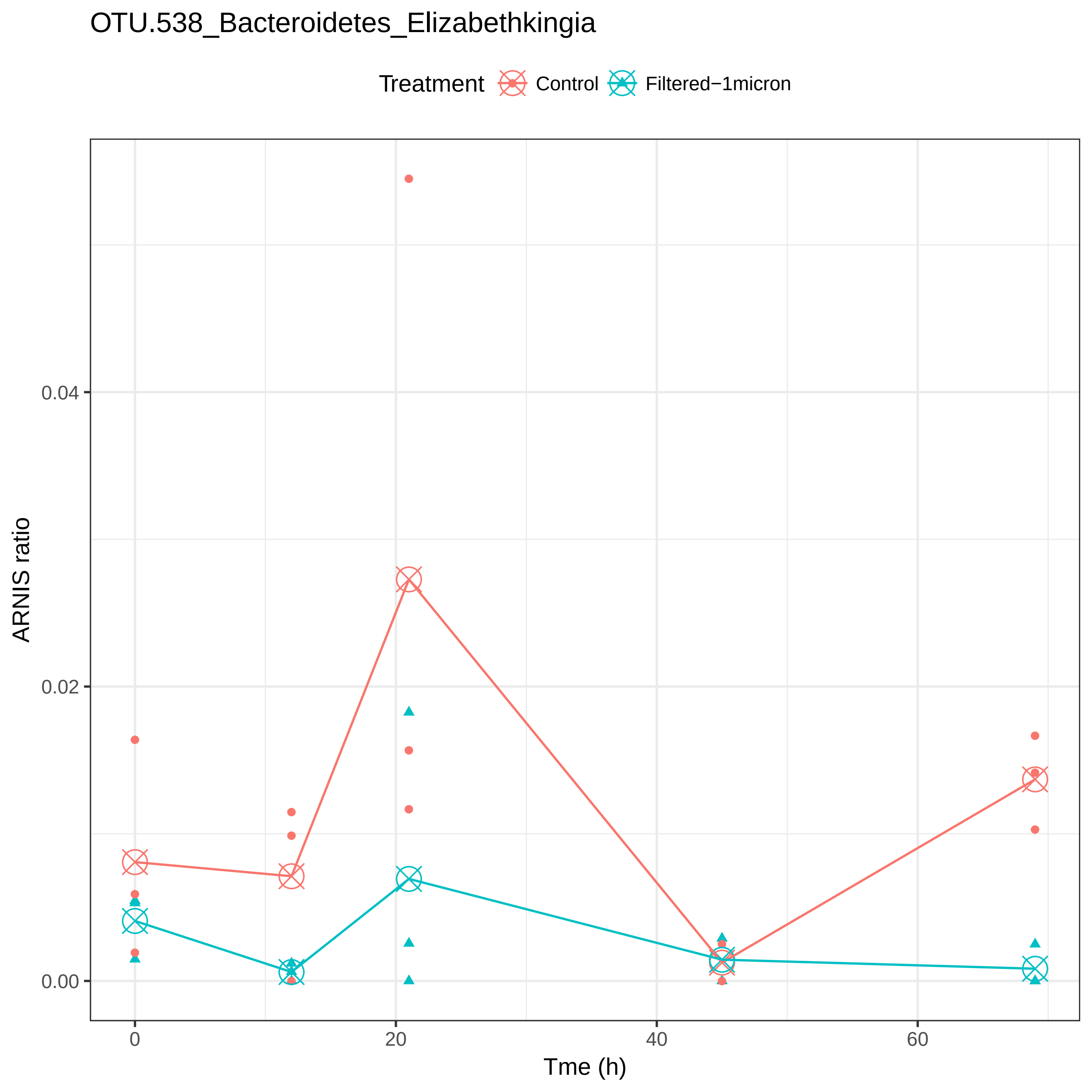

OTU.538_Bacteroidetes_Elizabethkingia
Treatment
Control
Filtered−1micron
ARNIS ratio
0.00
0.02
0.04
0
20
40
60
Tme (h)

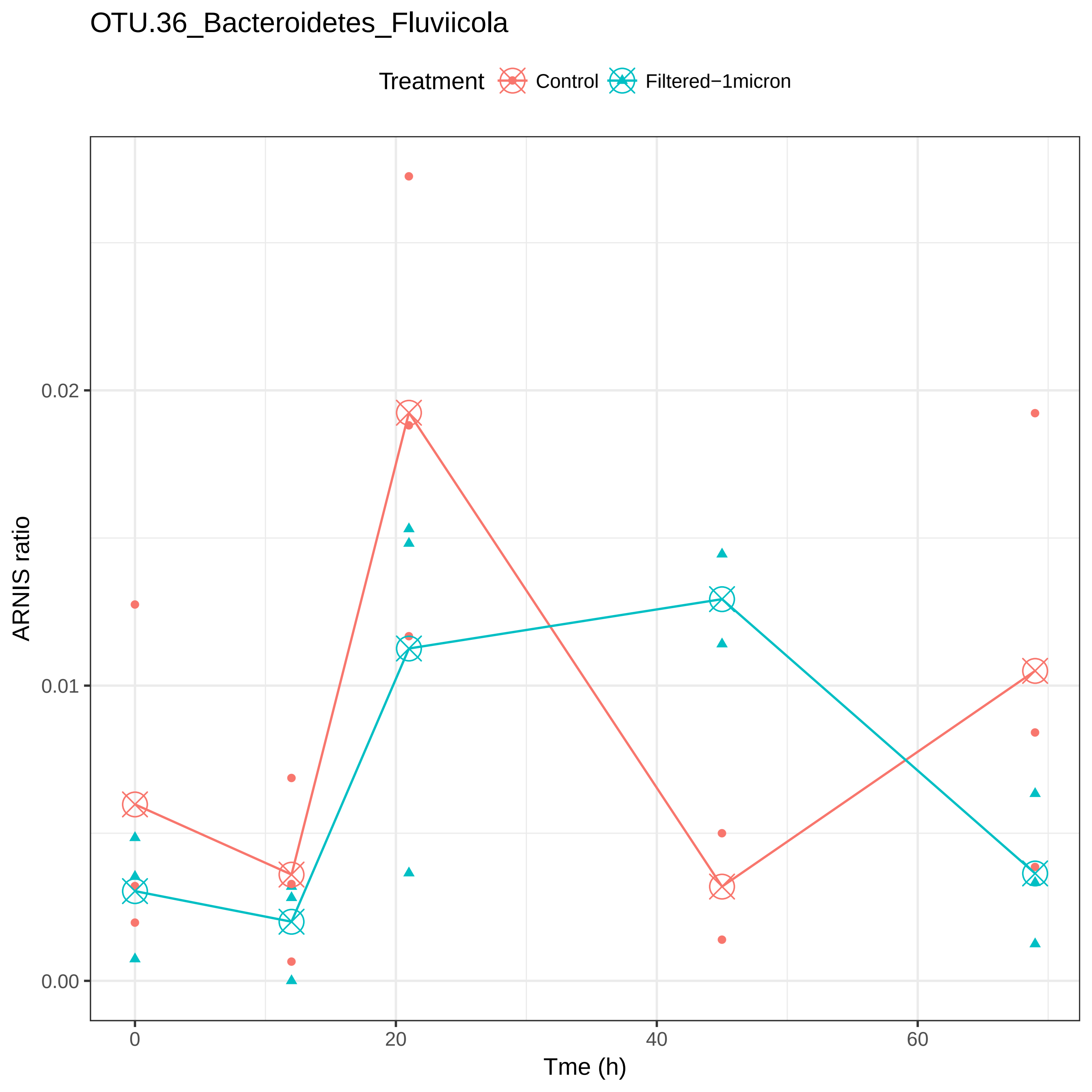
OTU.36_Bacteroidetes_Fluviicola
Treatment
Control
Filtered−1micron
ARNIS ratio
0.00
0.01
0.02
0
20
40
60
Tme (h)

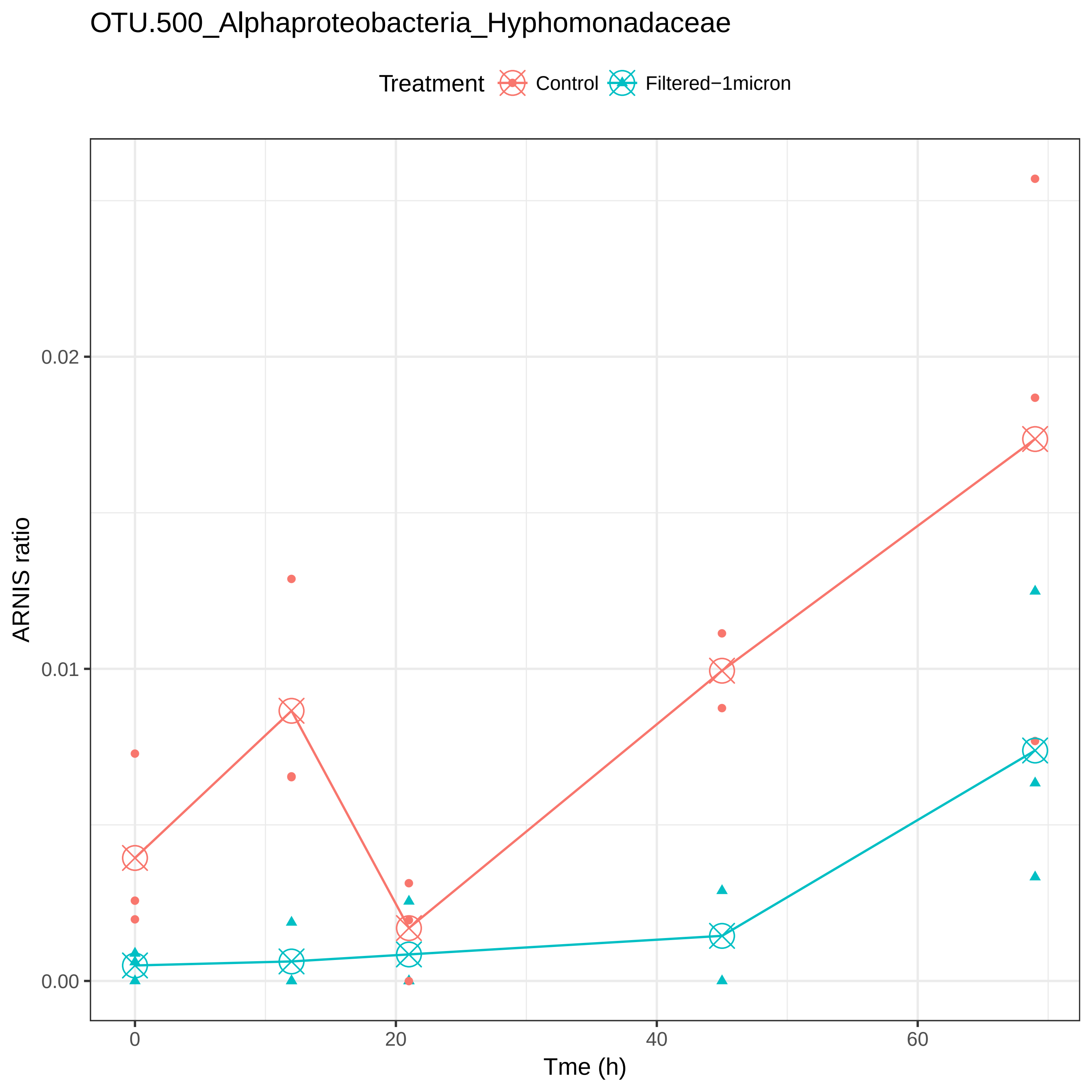
OTU.500_Alphaproteobacteria_Hyphomonadaceae
Treatment
Control
Filtered−1micron
ARNIS ratio
0.02
0.01
0.00
0
20
40
60
Tme (h)

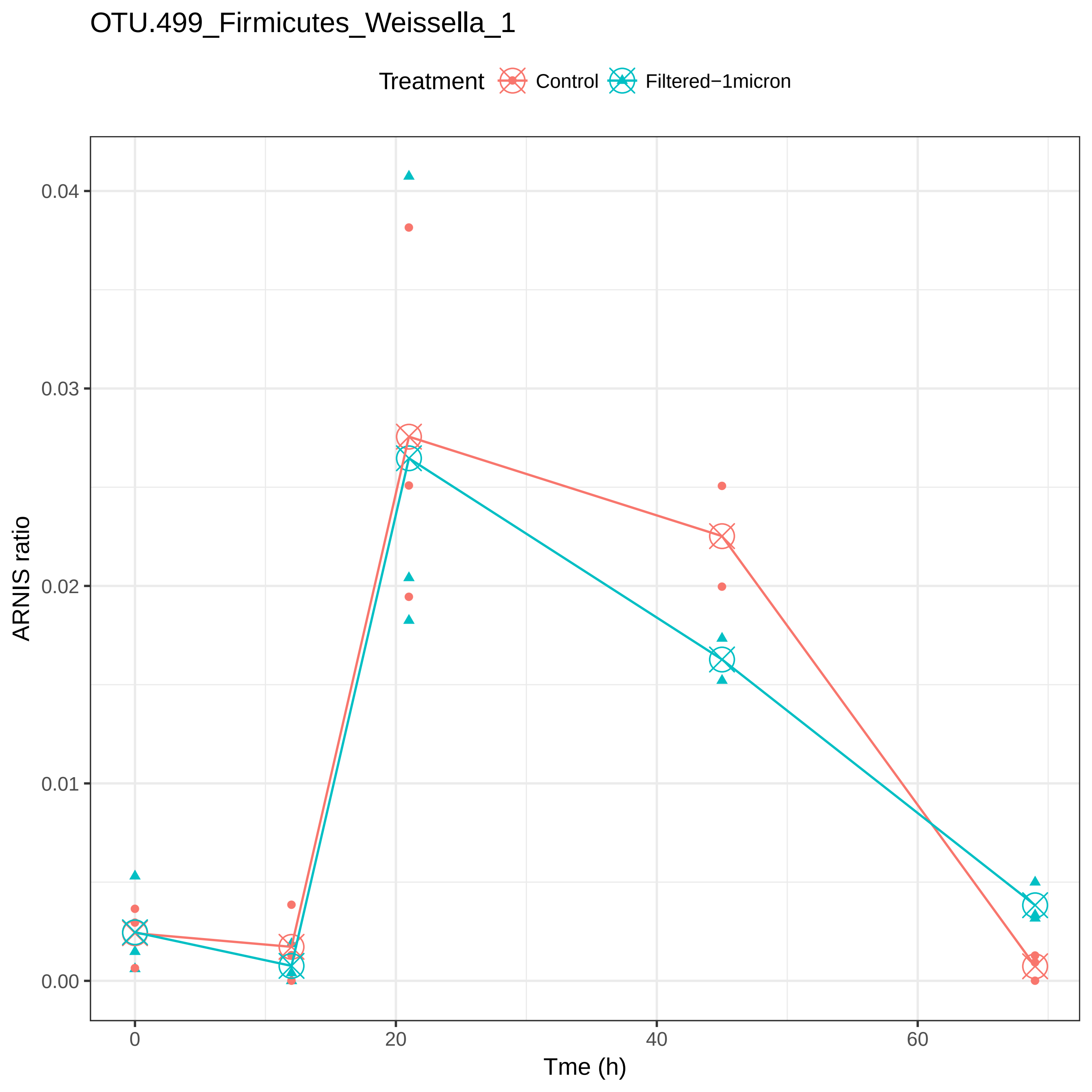

OTU.499_Firmicutes_Weissella_1
Treatment
Control
Filtered−1micron
ARNIS ratio
0.00
0.01
0.02
0.03
0.04
0
20
40
60
Tme (h)

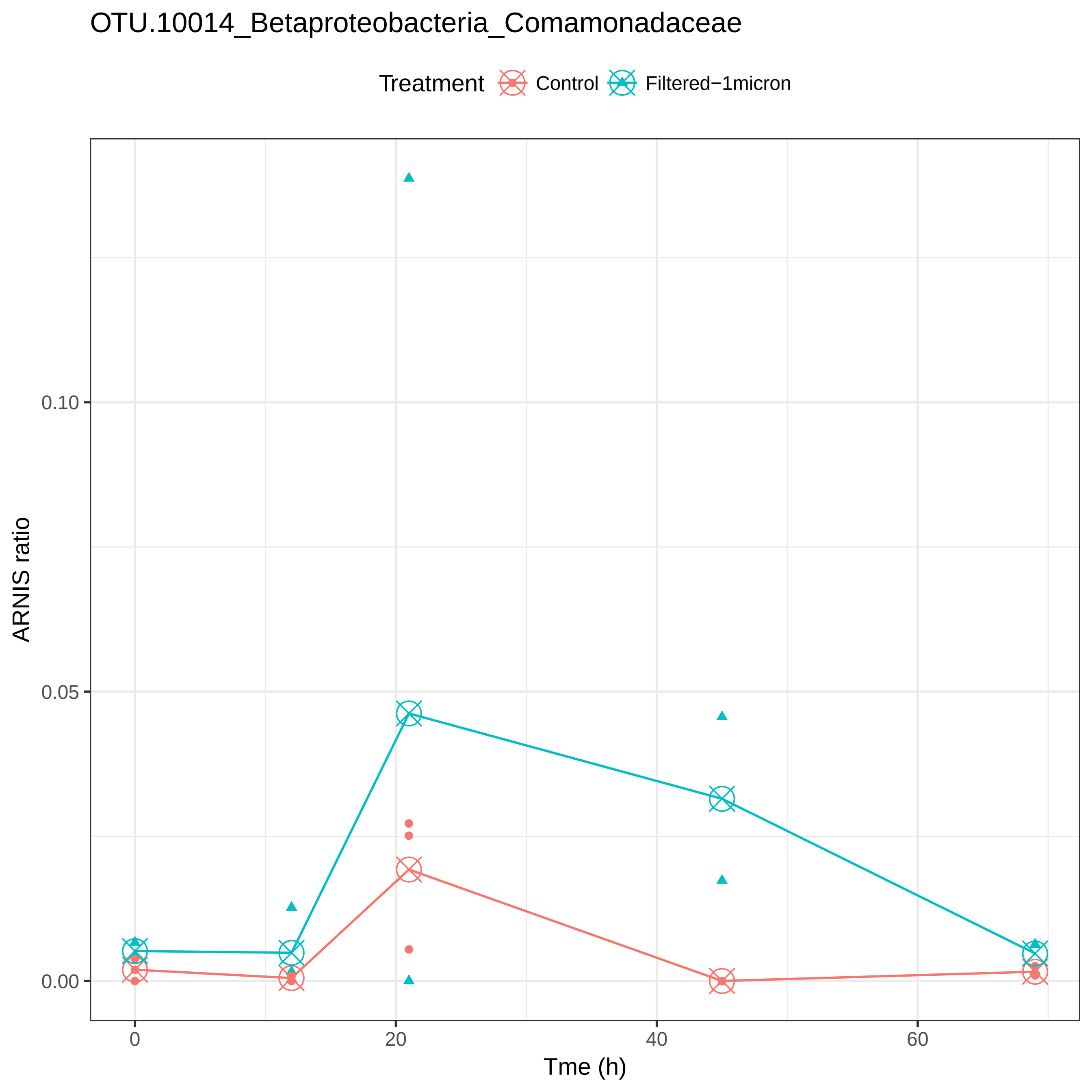

OTU.10014_Betaproteobacteria_Comamonadaceae
Treatment
Control
Filtered−1micron
ARNIS ratio
0.10
0.05
0.00
0
20
40
60
Tme (h)

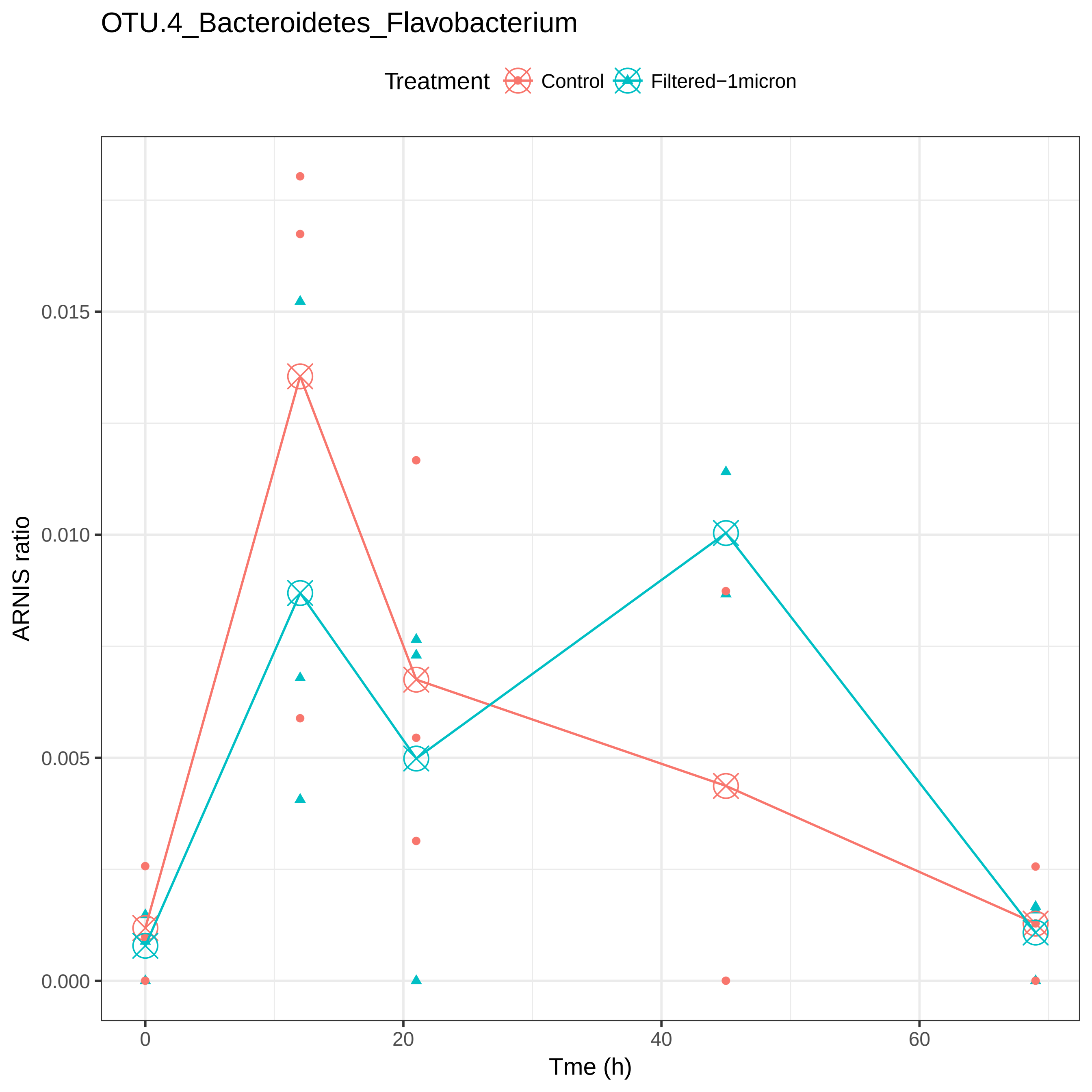

OTU.4_Bacteroidetes_Flavobacterium
Treatment
Control
Filtered−1micron
ARNIS ratio
0.000
0.005
0.010
0.015
0
20
40
60
Tme (h)

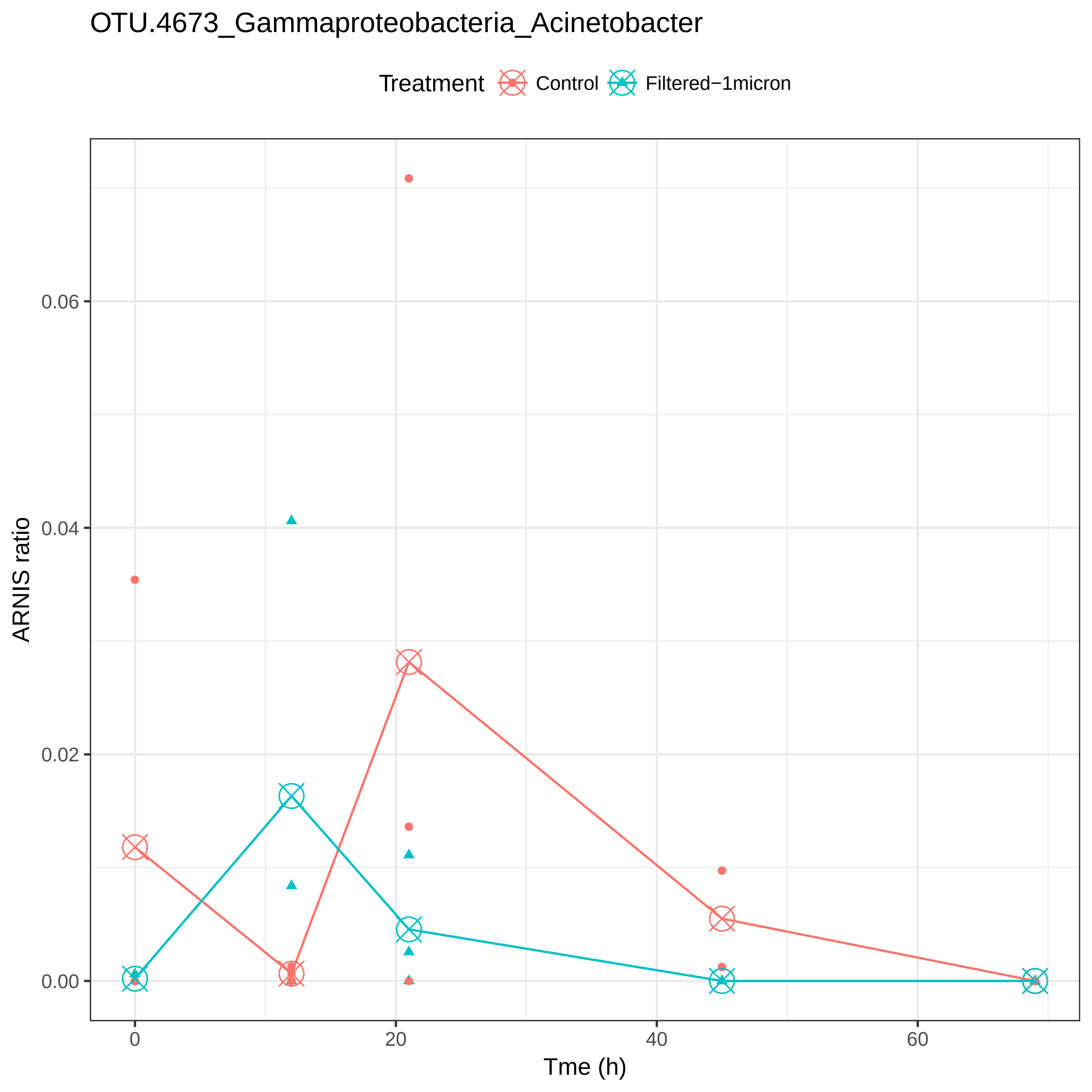
OTU.4673_Gammaproteobacteria_Acinetobacter
Treatment
Control
Filtered−1micron
ARNIS ratio
0.06
0.04
0.02
0.00
0
20
40
60
Tme (h)

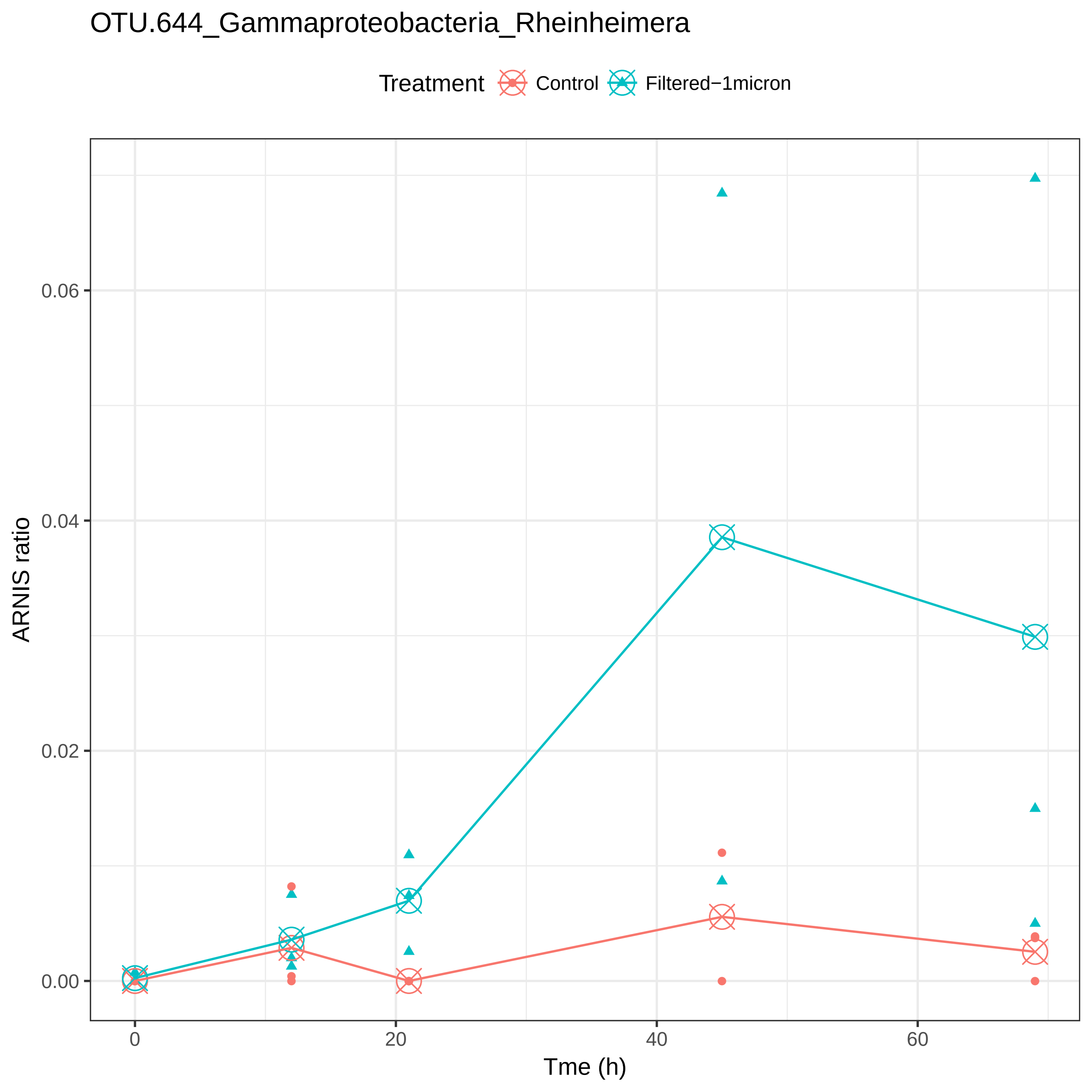
OTU.644_Gammaproteobacteria_Rheinheimera
Treatment
Control
Filtered−1micron
ARNIS ratio
0.06
0.04
0.02
0.00
0
20
40
60
Tme (h)

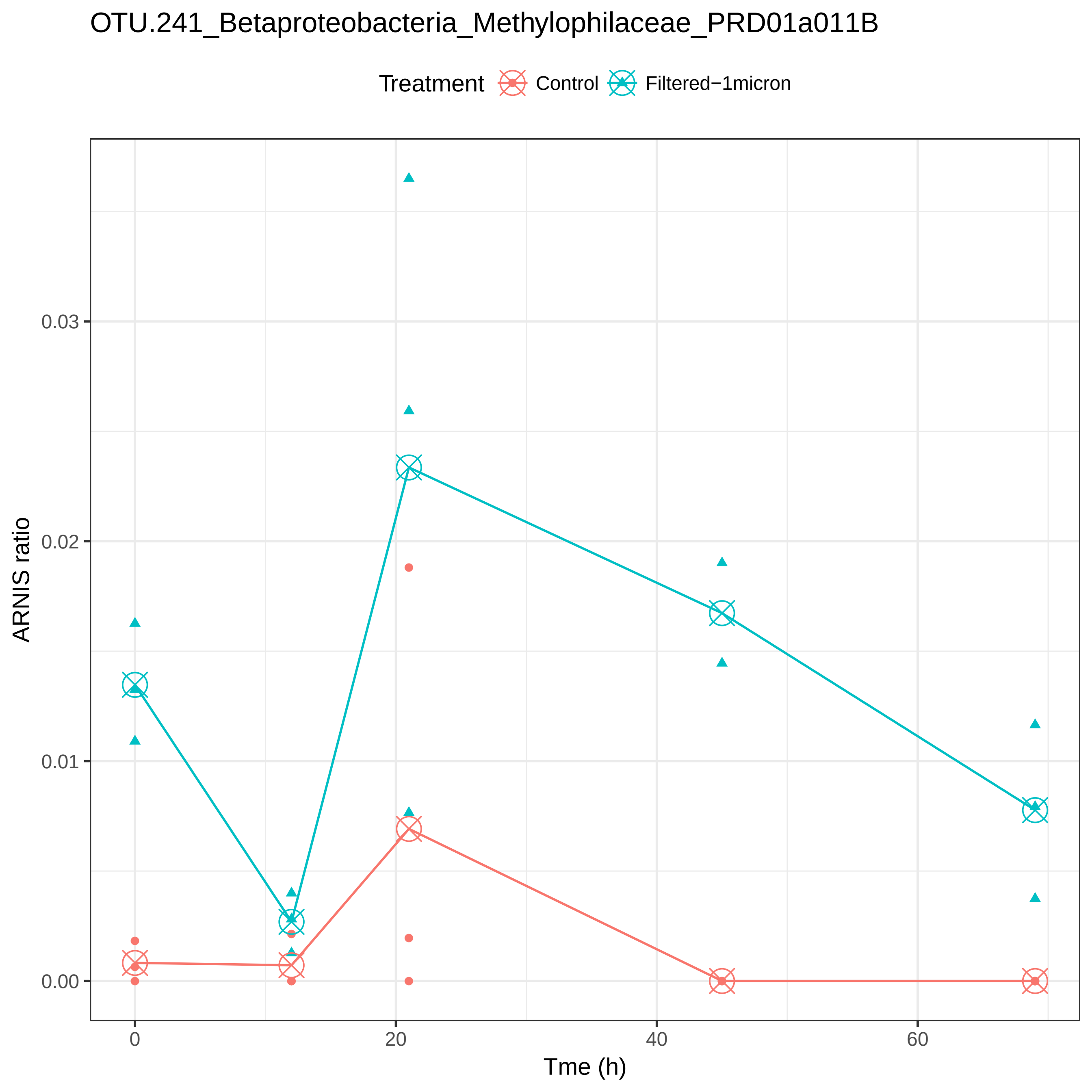

OTU.241_Betaproteobacteria_Methylophilaceae_PRD01a011B
Treatment
Control
Filtered−1micron
ARNIS ratio
0.03
0.02
0.01
0.00
0
20
40
60
Tme (h)

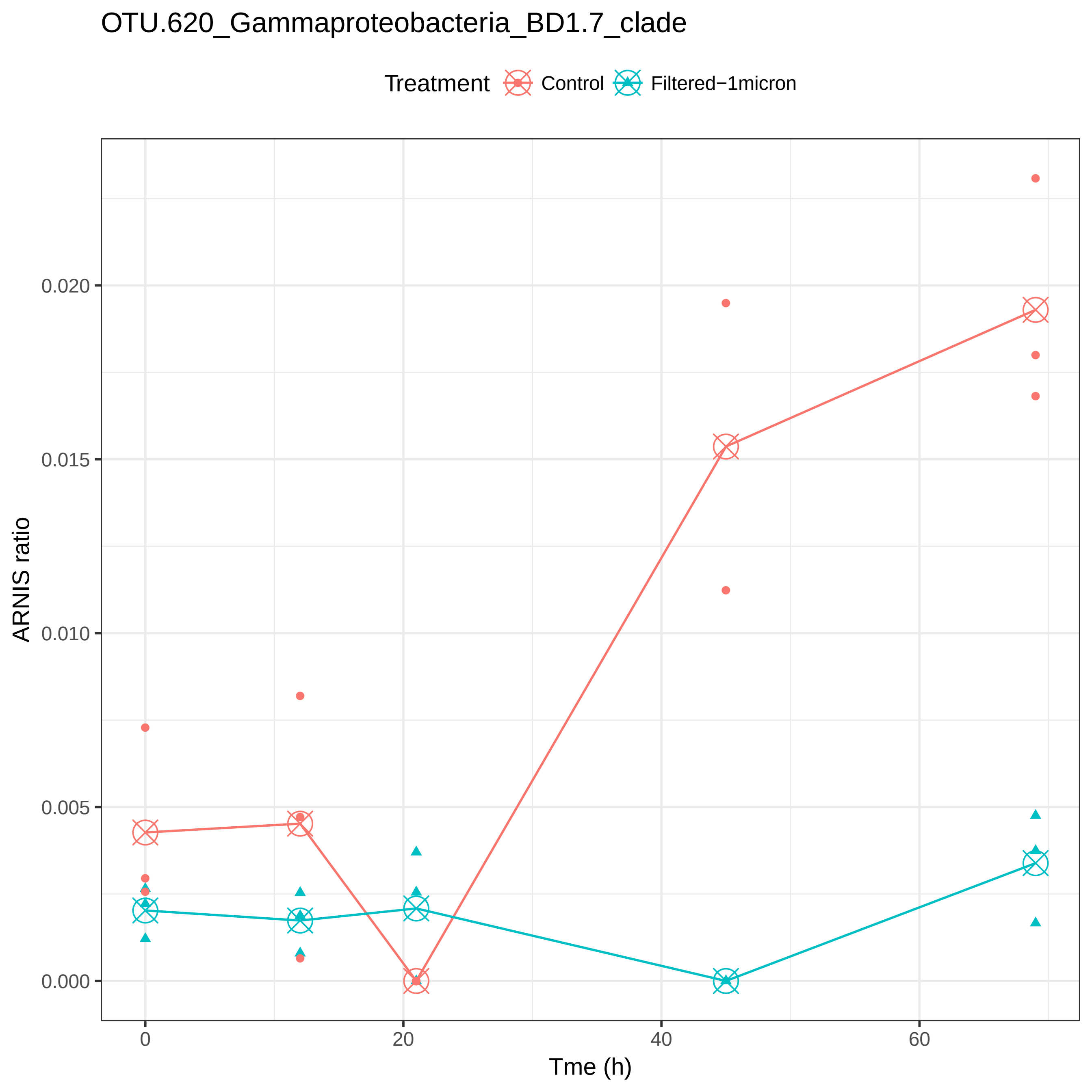

OTU.620_Gammaproteobacteria_BD1.7_clade
Treatment
Control
Filtered−1micron
ARNIS ratio
0.000
0.005
0.010
0.015
0.020
0
20
40
60
Tme (h)

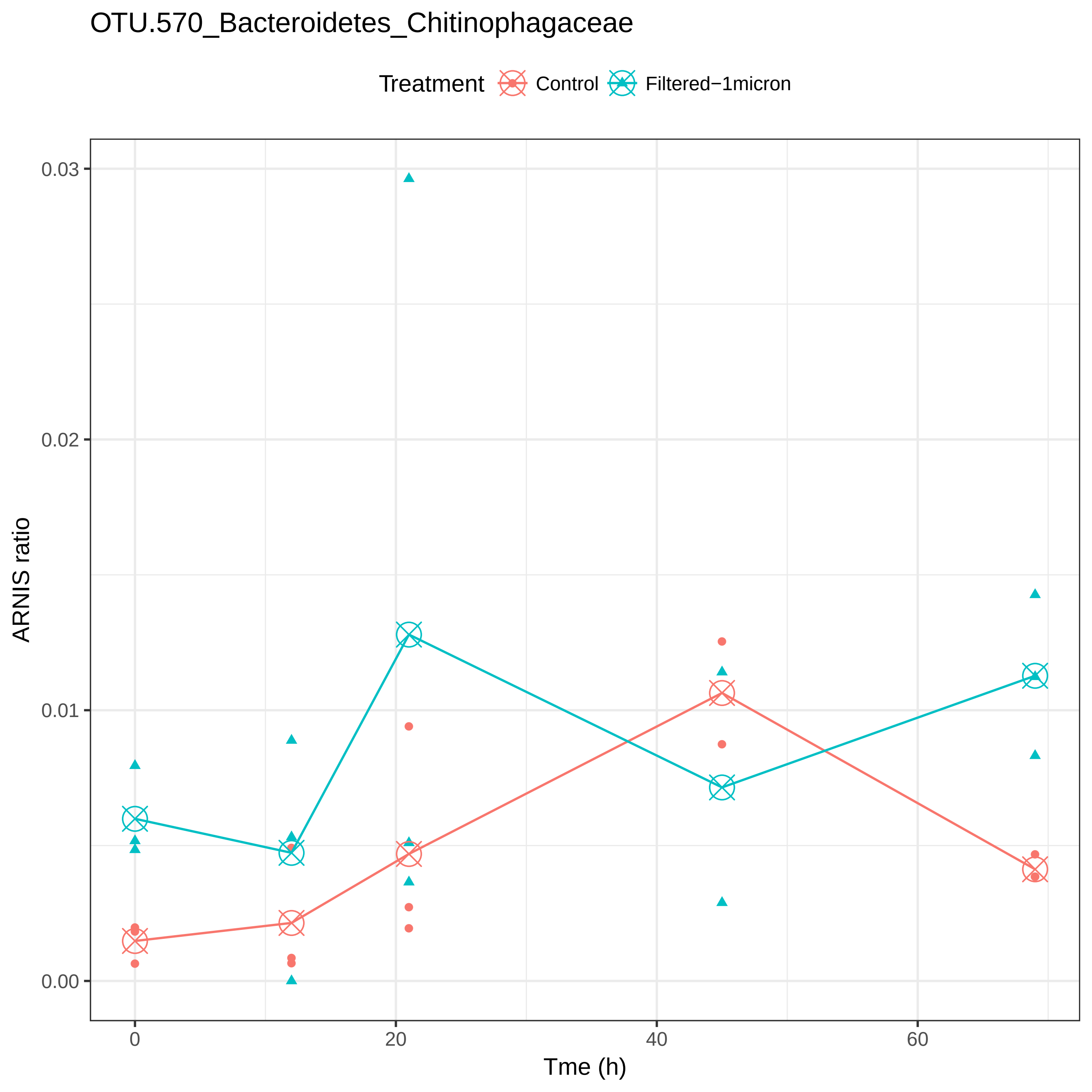

OTU.570_Bacteroidetes_Chitinophagaceae
Treatment
Control
Filtered−1micron
ARNIS ratio
0.03
0.02
0.01
0.00
0
20
40
60
Tme (h)

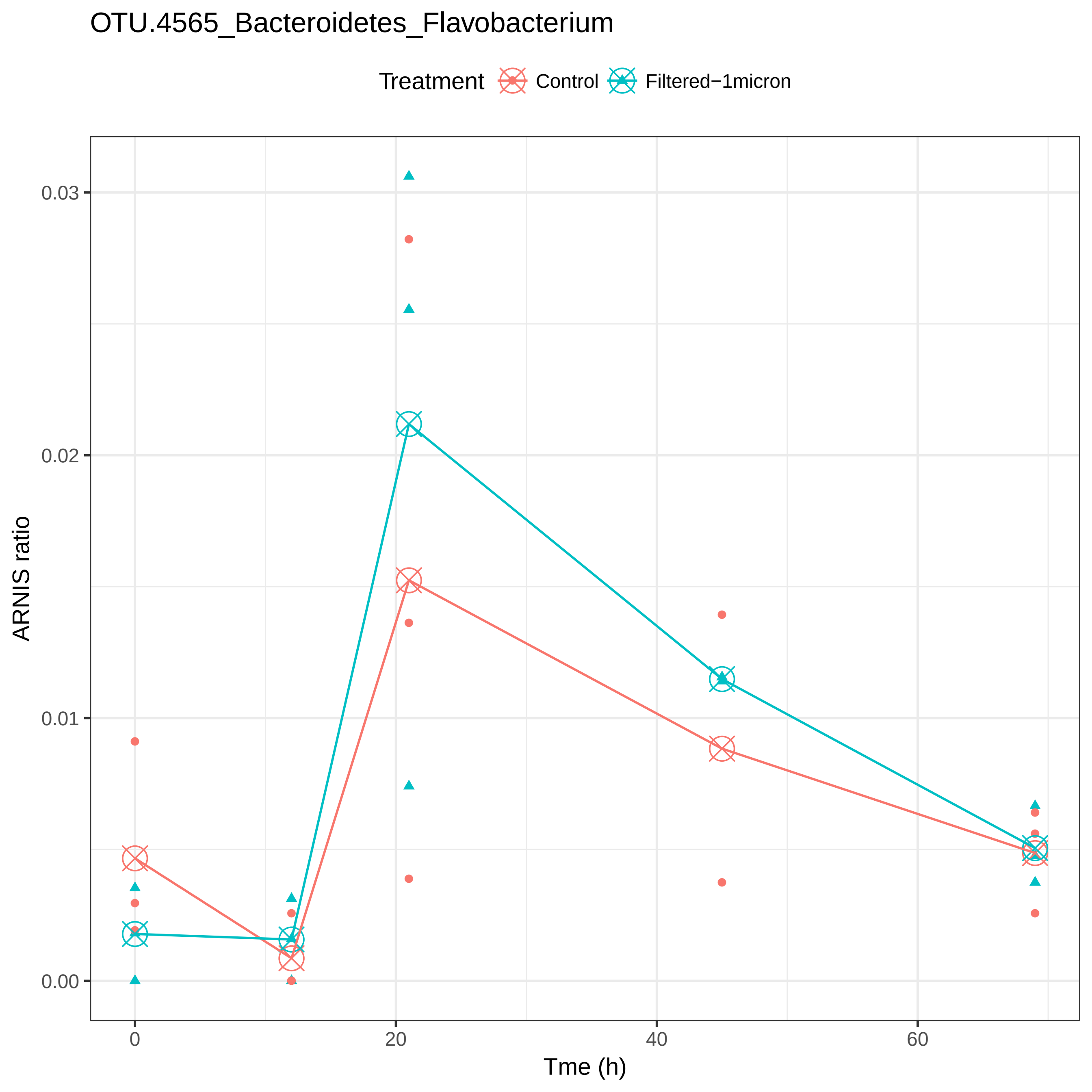
OTU.4565_Bacteroidetes_Flavobacterium
Treatment
Control
Filtered−1micron
ARNIS ratio
0.03
0.02
0.01
0.00
0
20
40
60
Tme (h)

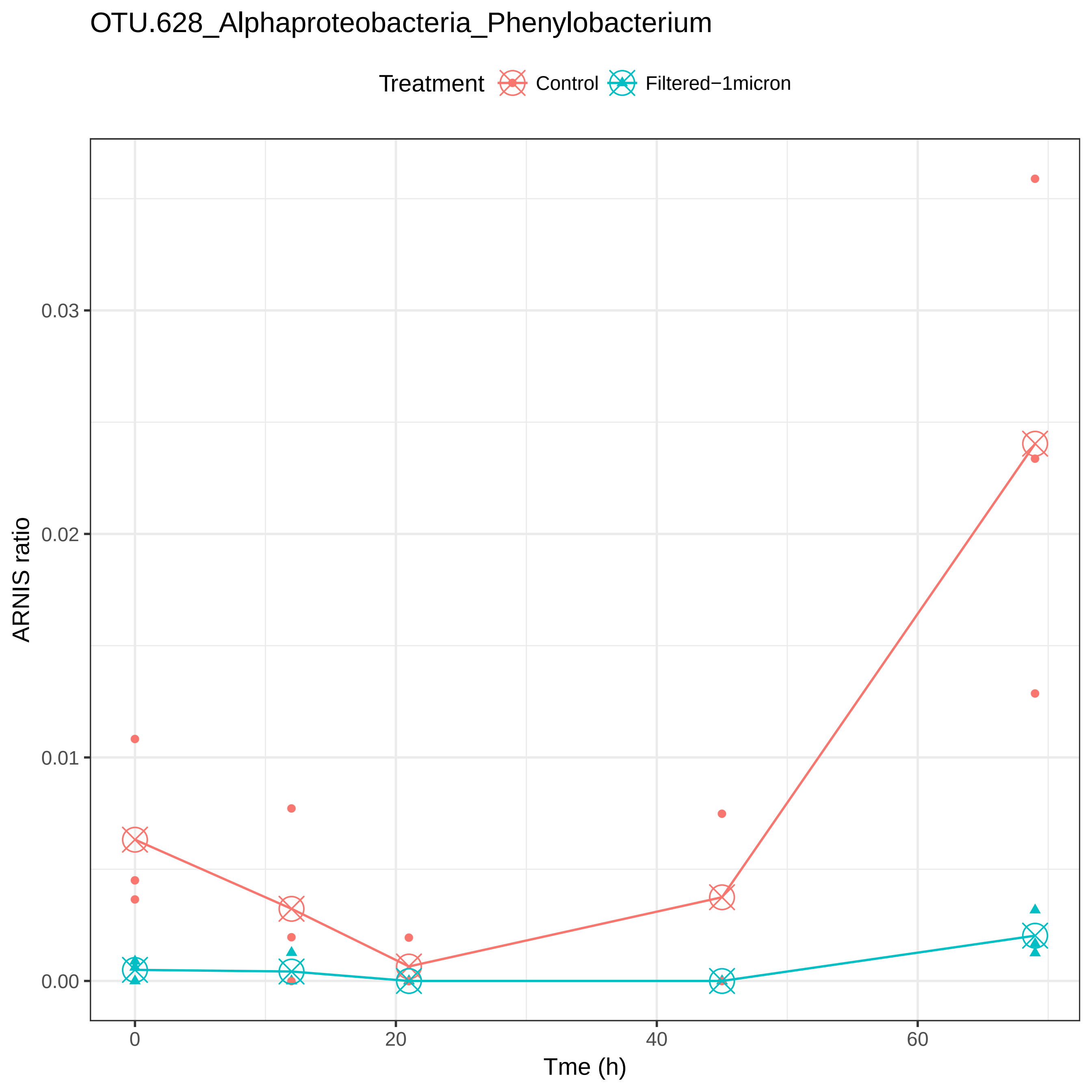

OTU.628_Alphaproteobacteria_Phenylobacterium
Treatment
Control
Filtered−1micron
ARNIS ratio
0.00
0.01
0.02
0.03
0
20
40
60
Tme (h)

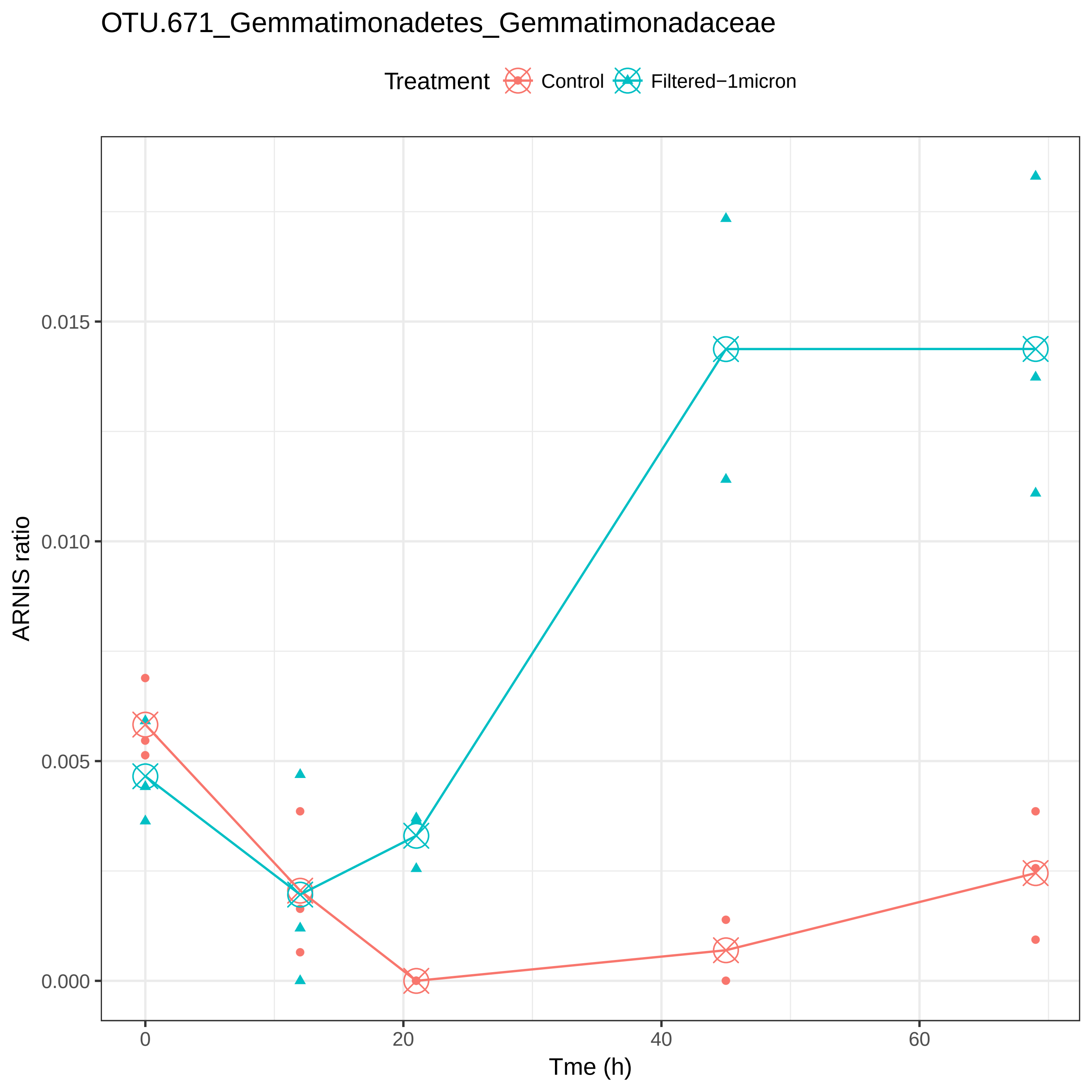

OTU.671_Gemmatimonadetes_Gemmatimonadaceae
Treatment
Control
Filtered−1micron
ARNIS ratio
0.015
0.010
0.005
0.000
0
20
40
60
Tme (h)

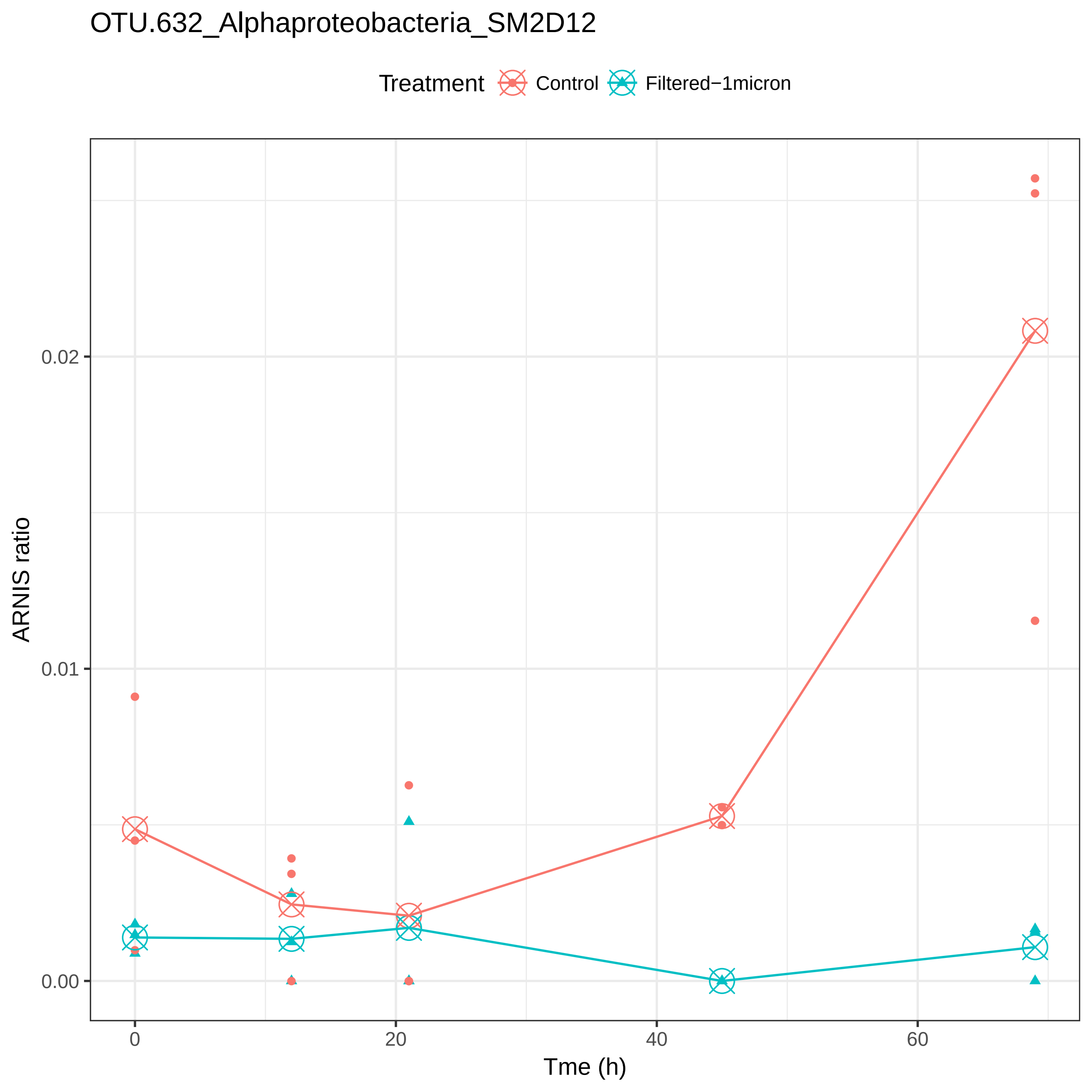

OTU.632_Alphaproteobacteria_SM2D12
Treatment
Control
Filtered−1micron
ARNIS ratio
0.00
0.01
0.02
0
20
40
60
Tme (h)

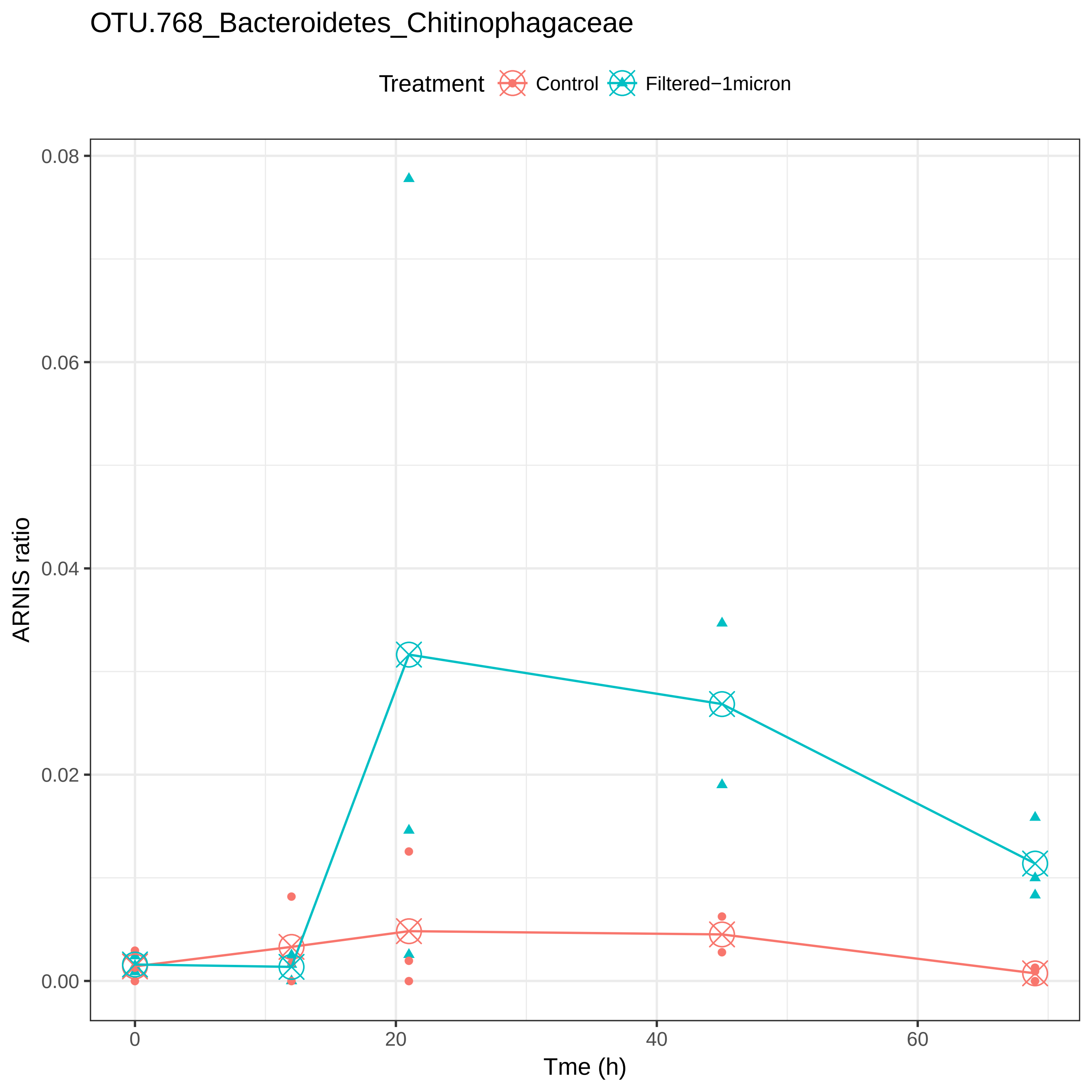
OTU.768_Bacteroidetes_Chitinophagaceae
Treatment
Control
Filtered−1micron
ARNIS ratio
0.08
0.06
0.04
0.02
0.00
0
20
40
60
Tme (h)

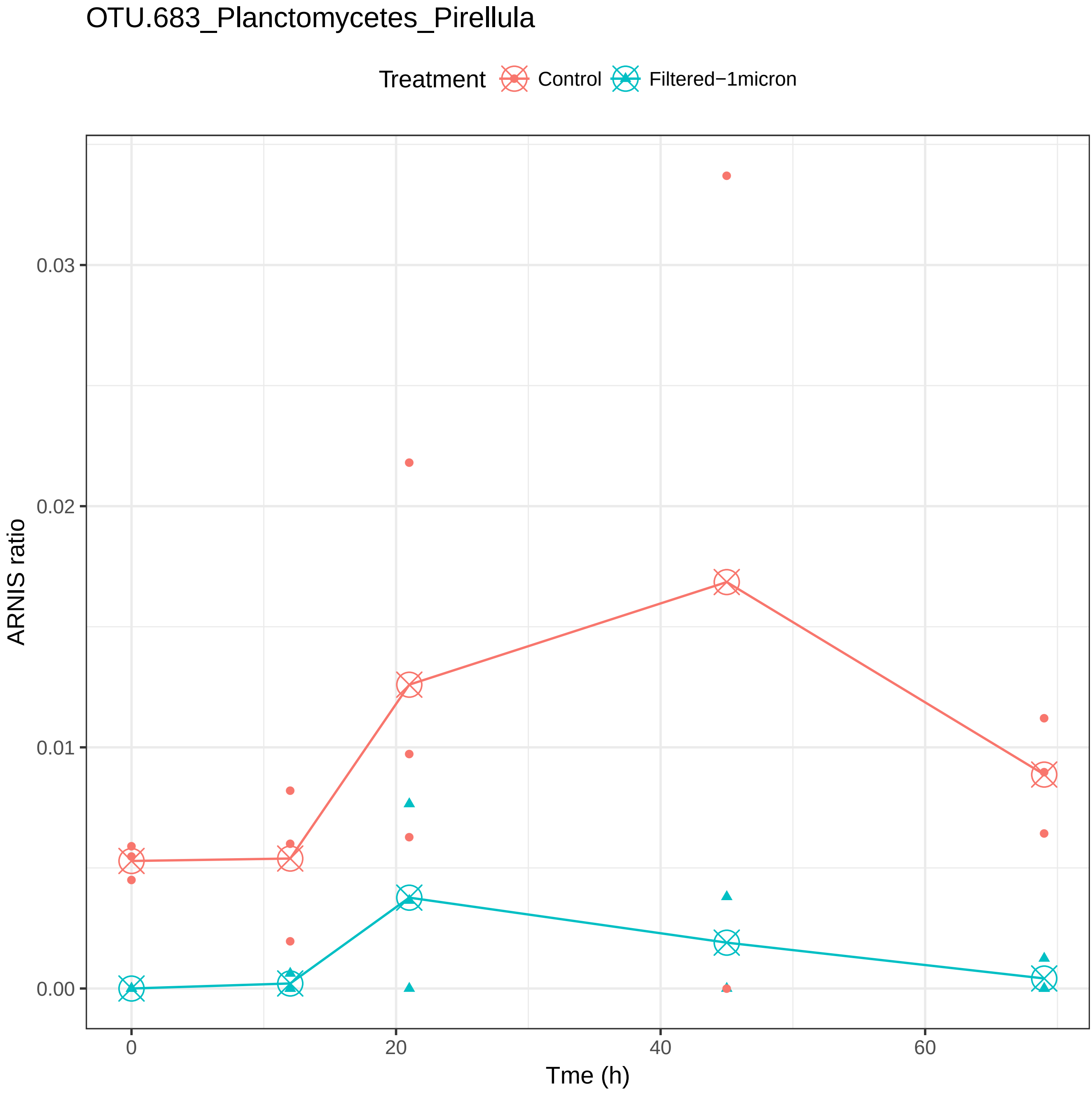

OTU.683_Planctomycetes_Pirellula
Treatment
Control
Filtered−1micron
ARNIS ratio
0.03
0.02
0.01
0.00
0
20
40
60
Tme (h)

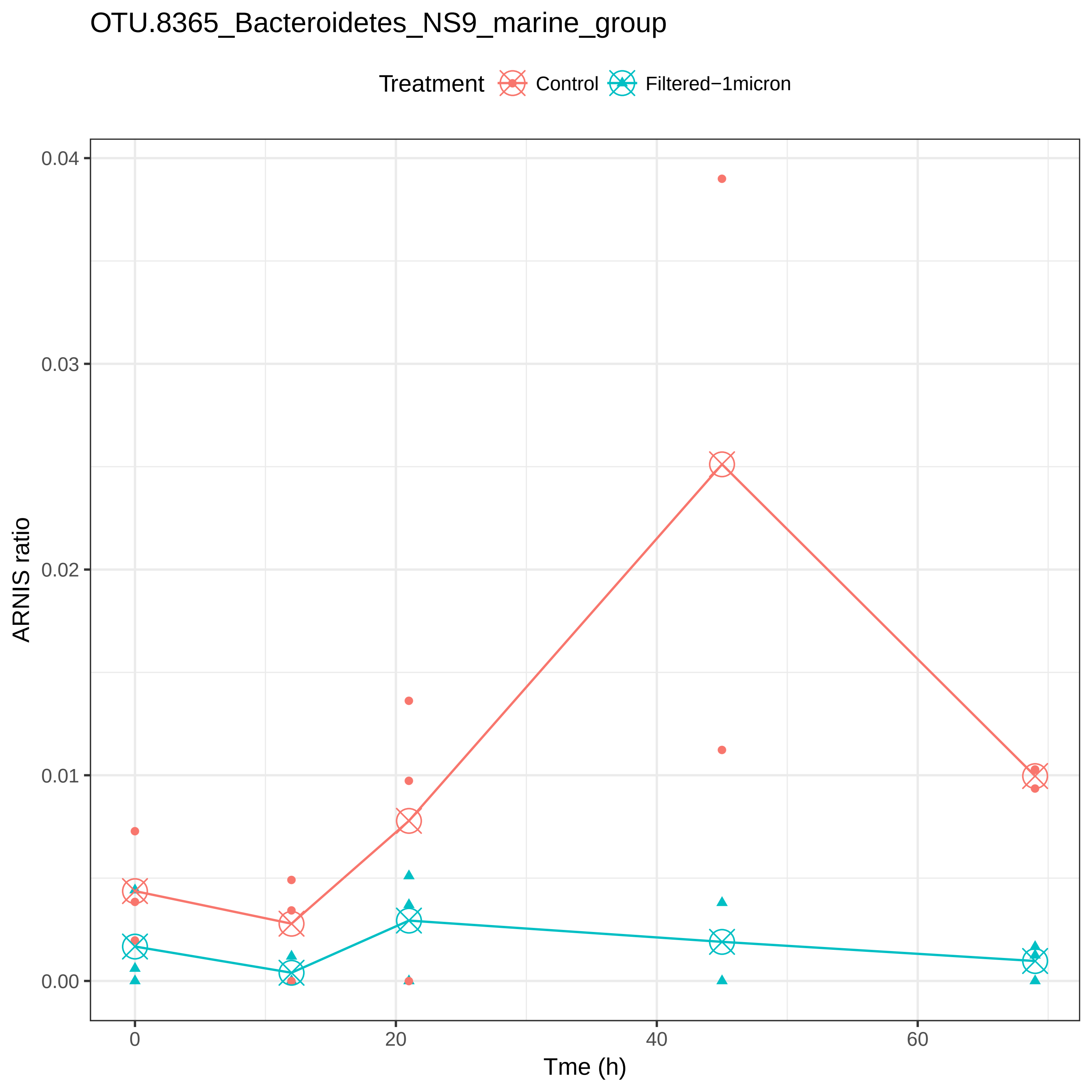

OTU.8365_Bacteroidetes_NS9_marine_group
Treatment
Control
Filtered−1micron
ARNIS ratio
0.00
0.01
0.02
0.03
0.04
0
20
40
60
Tme (h)

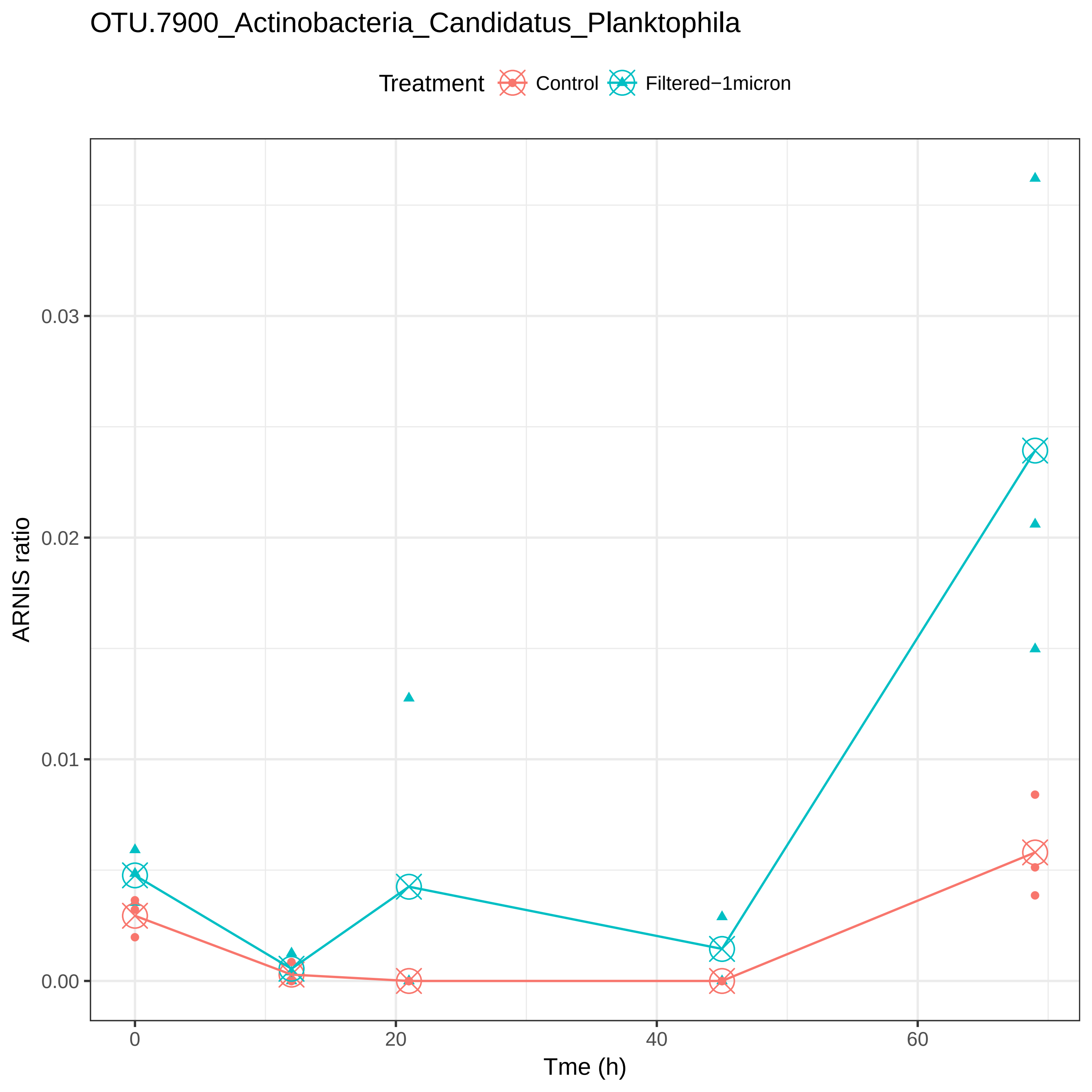

OTU.7900_Actinobacteria_Candidatus_Planktophila
Treatment
Control
Filtered−1micron
ARNIS ratio
0.00
0.01
0.02
0.03
0
20
40
60
Tme (h)

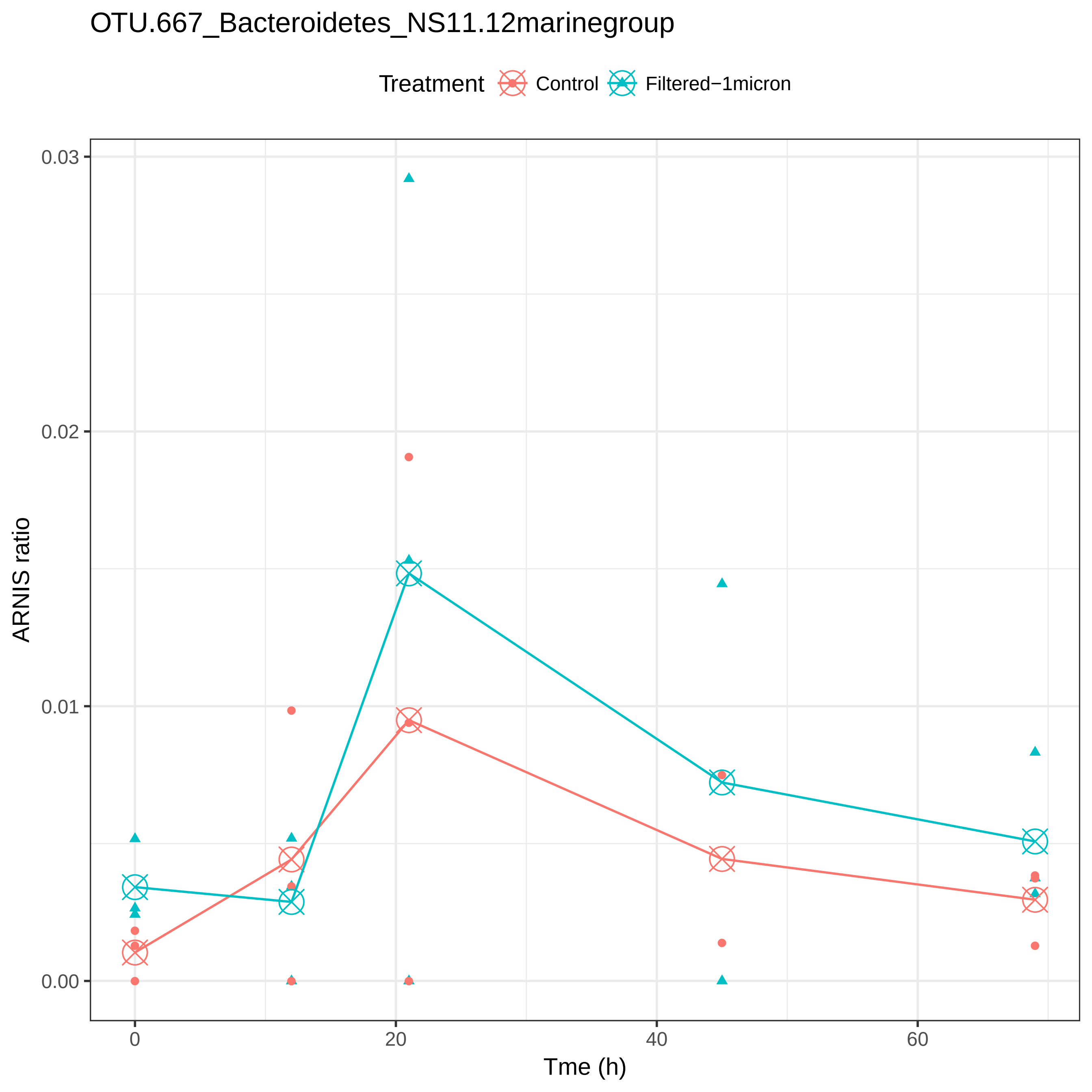
OTU.667_Bacteroidetes_NS11.12marinegroup
Treatment
Control
Filtered−1micron
ARNIS ratio
0.03
0.02
0.01
0.00
0
20
40
60
Tme (h)

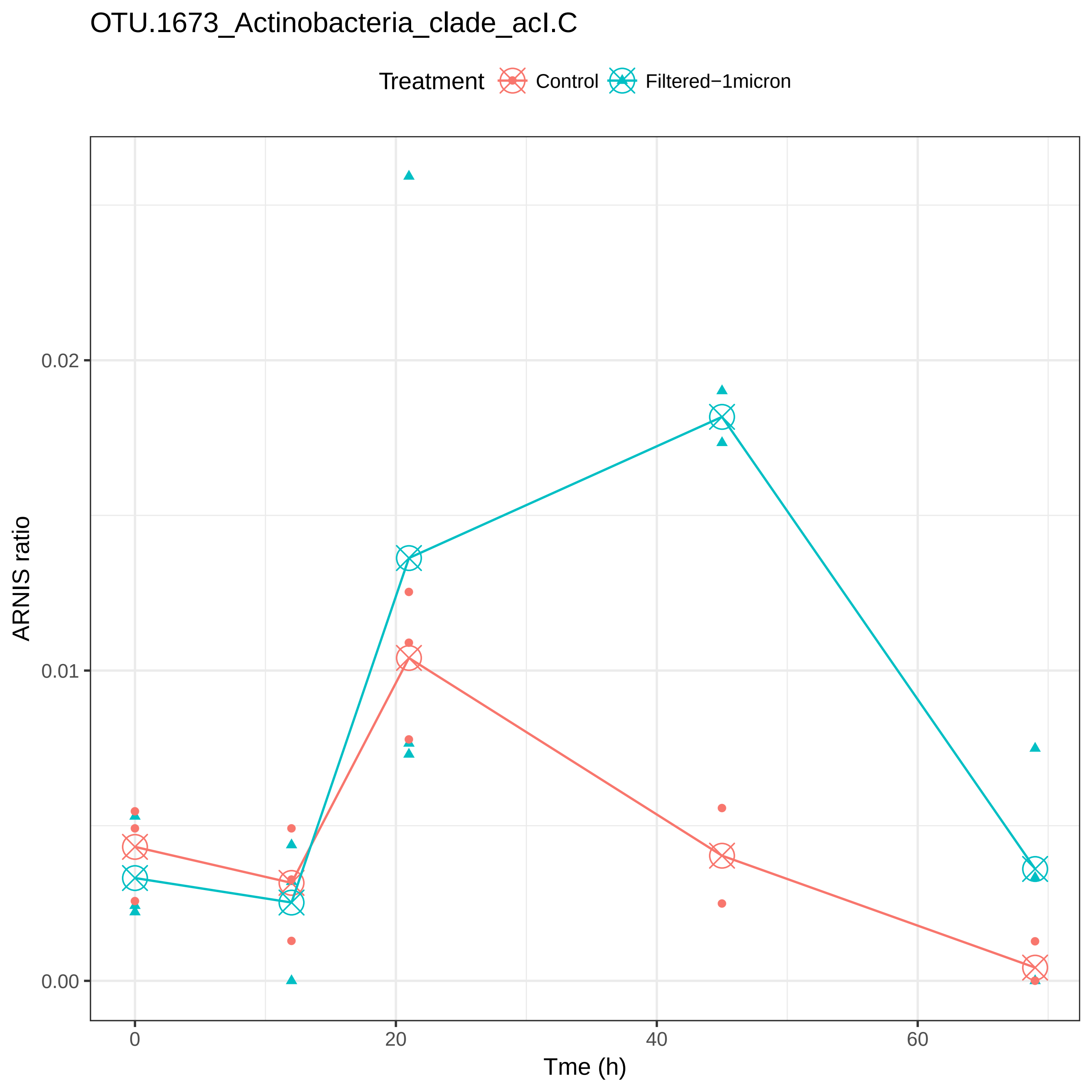

OTU.1673_Actinobacteria_clade_acI.C
Treatment
Control
Filtered−1micron
ARNIS ratio
0.00
0.01
0.02
0
20
40
60
Tme (h)

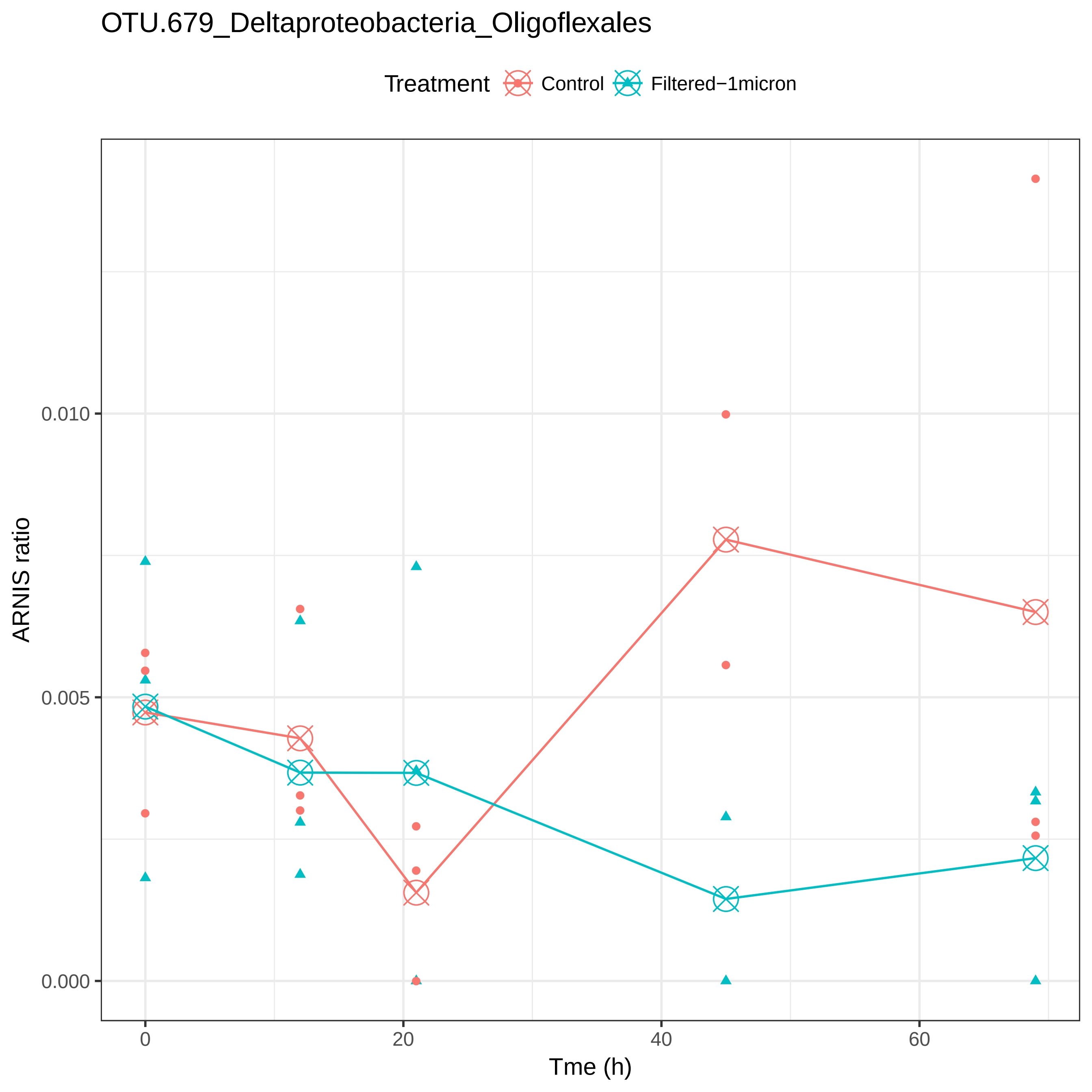

OTU.679_Deltaproteobacteria_Oligoflexales
Treatment
Control
Filtered−1micron
ARNIS ratio
0.010
0.005
0.000
0
20
40
60
Tme (h)

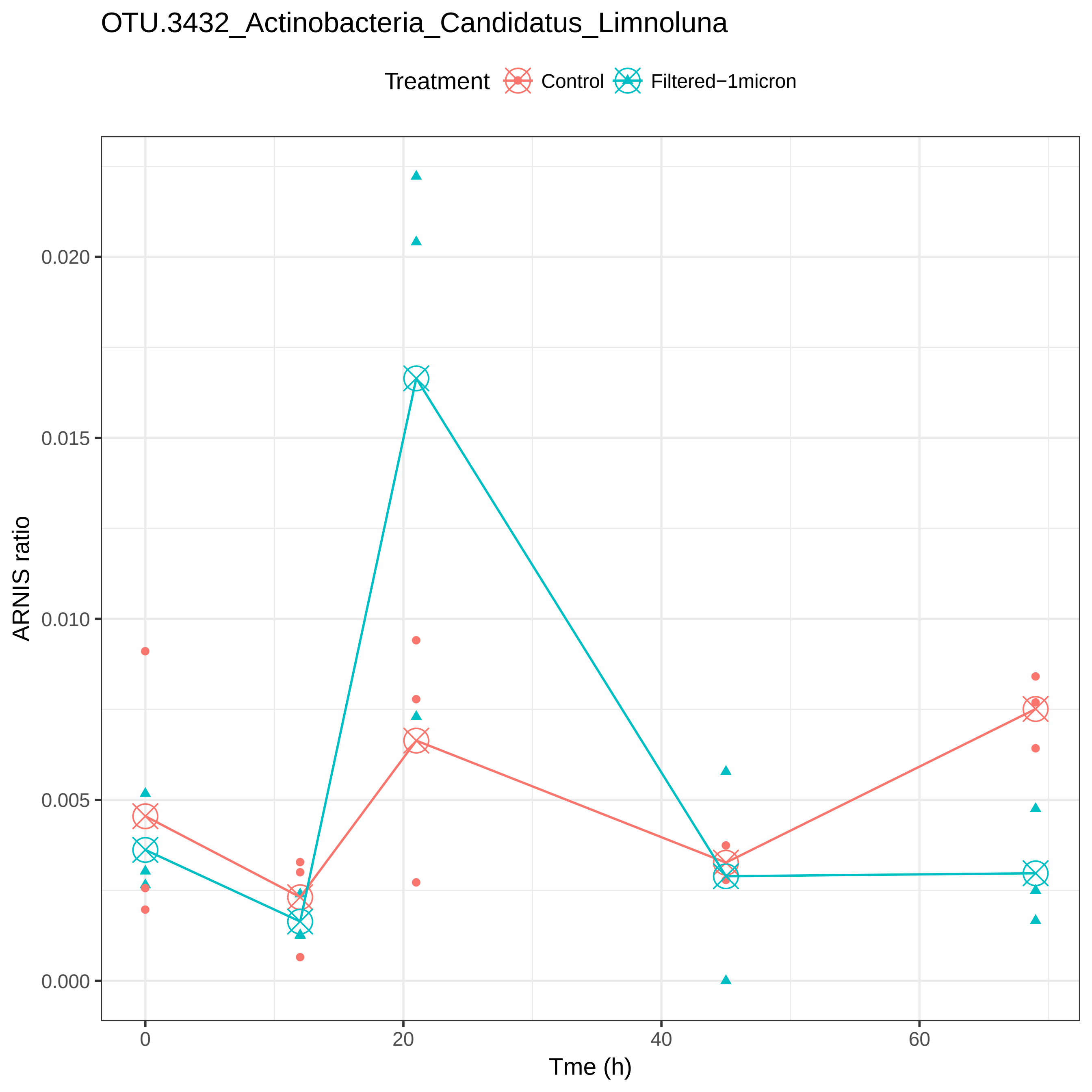

OTU.3432_Actinobacteria_Candidatus_Limnoluna
Treatment
Control
Filtered−1micron
ARNIS ratio
0.000
0.005
0.010
0.015
0.020
0
20
40
60
Tme (h)

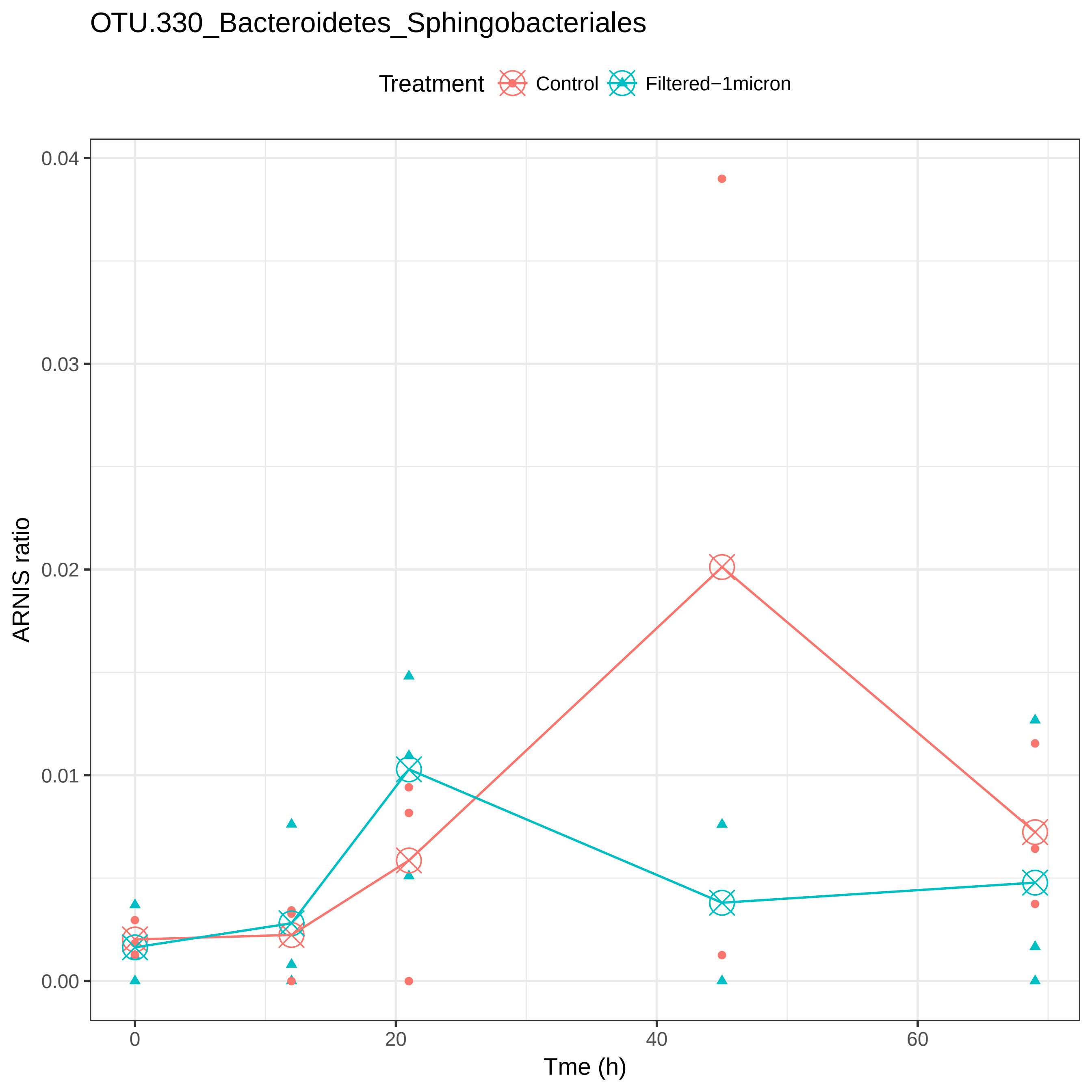

OTU.330_Bacteroidetes_Sphingobacteriales
Treatment
Control
Filtered−1micron
ARNIS ratio
0.04
0.03
0.02
0.01
0.00
0
20
40
60
Tme (h)

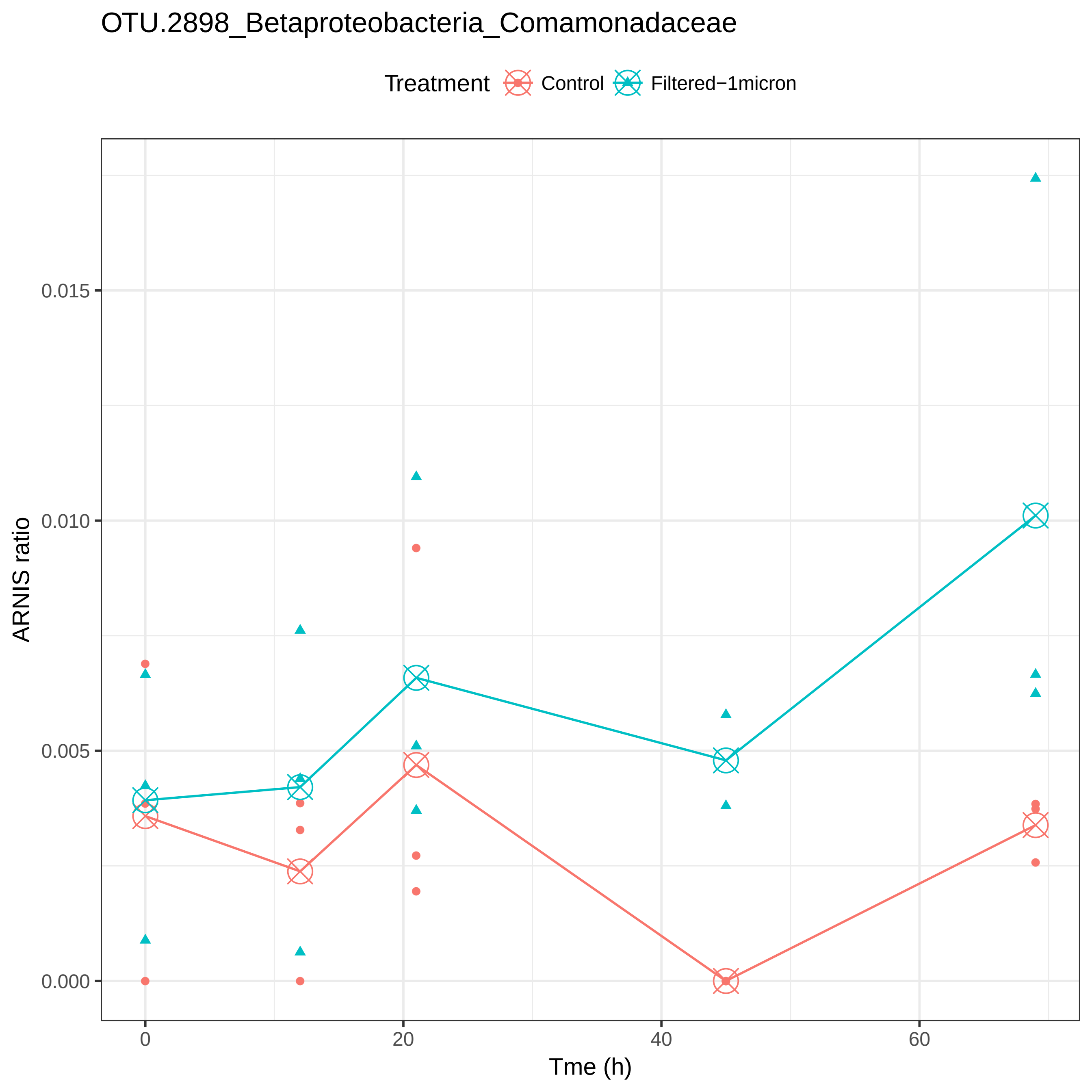
OTU.2898_Betaproteobacteria_Comamonadaceae
Treatment
Control
Filtered−1micron
ARNIS ratio
0.000
0.005
0.010
0.015
0
20
40
60
Tme (h)

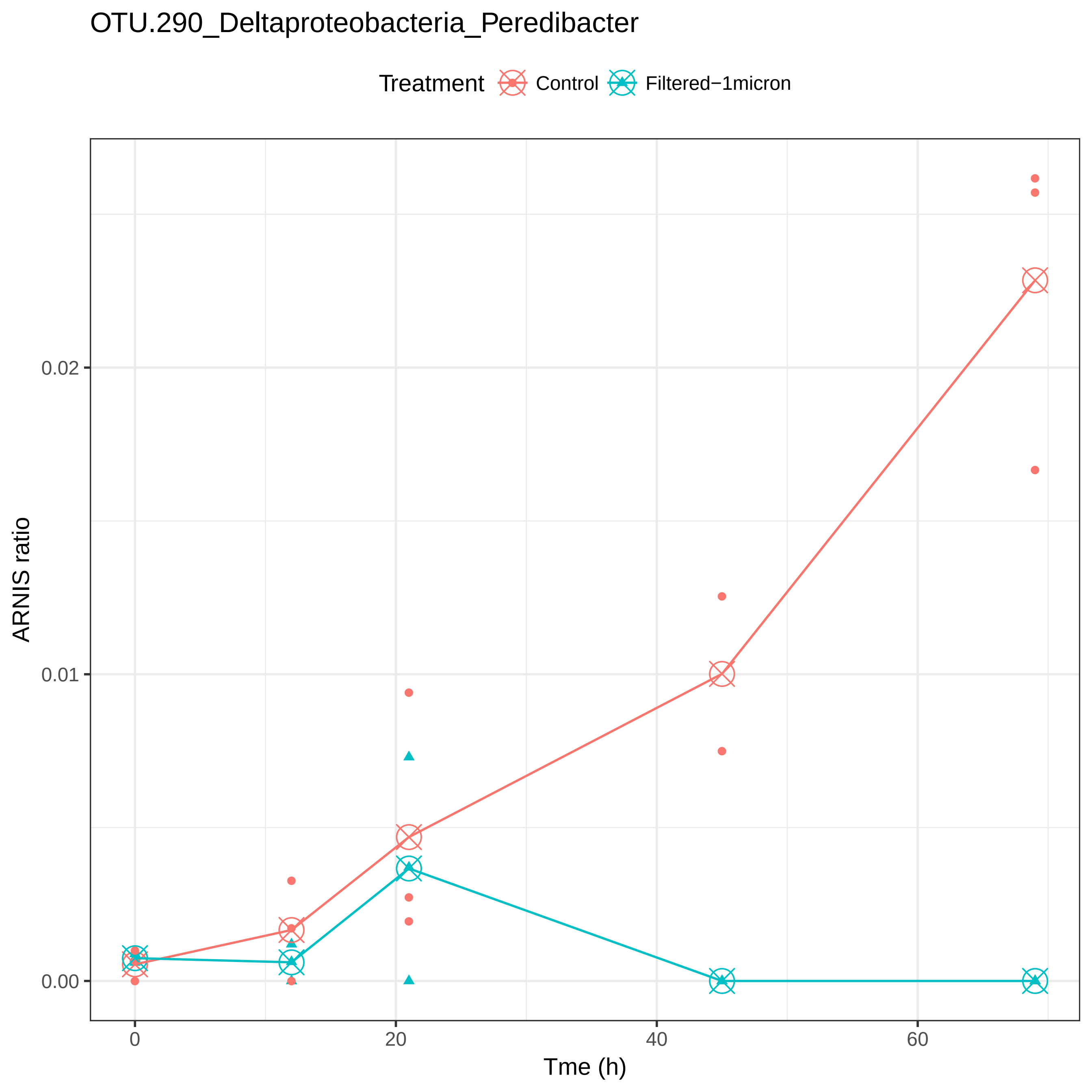

OTU.290_Deltaproteobacteria_Peredibacter
Treatment
Control
Filtered−1micron
ARNIS ratio
0.00
0.01
0.02
0
20
40
60
Tme (h)

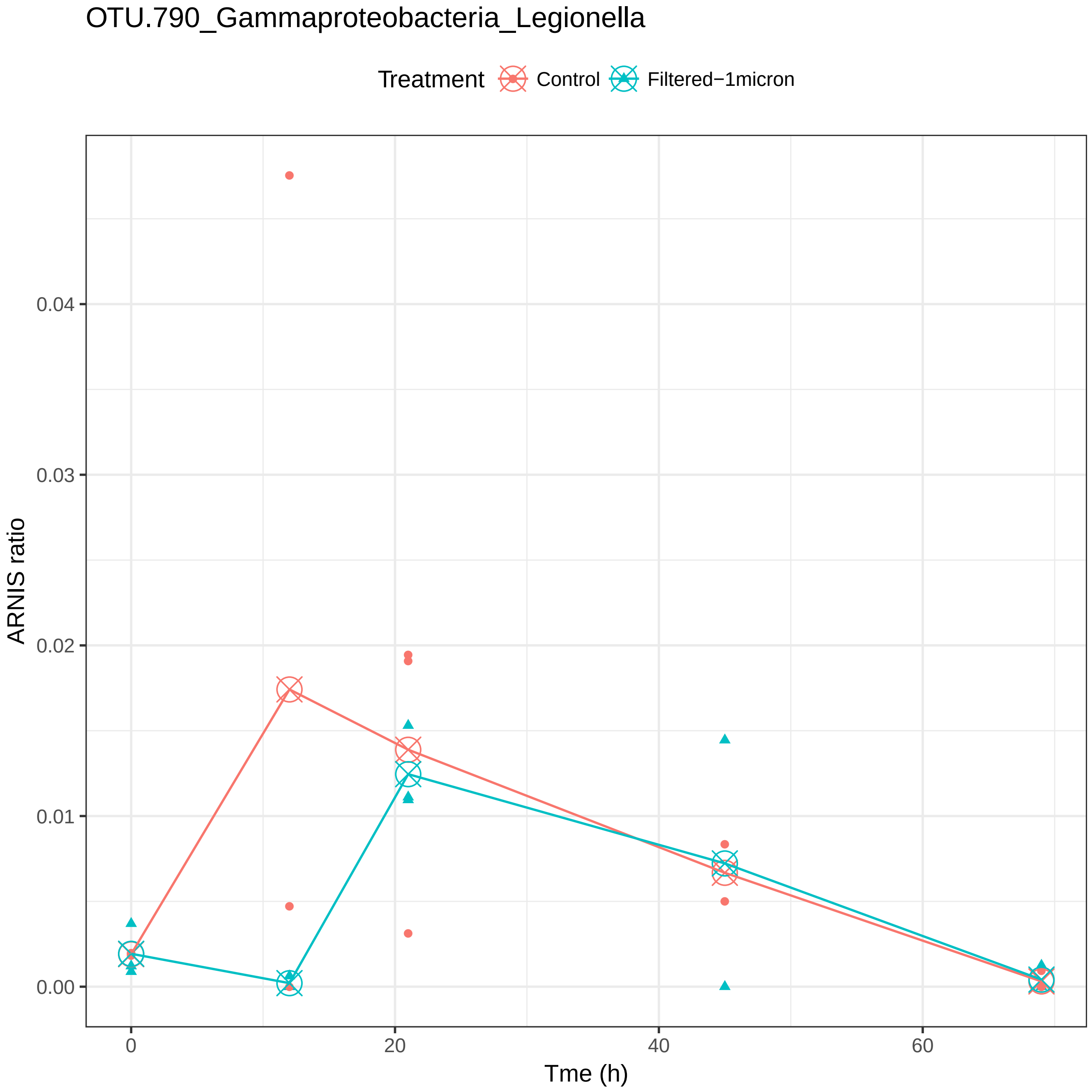

OTU.790_Gammaproteobacteria_Legionella
Treatment
Control
Filtered−1micron
0.04
0.03
0.02
0.01
0.00
ARNIS ratio
0
20
40
60
Tme (h)

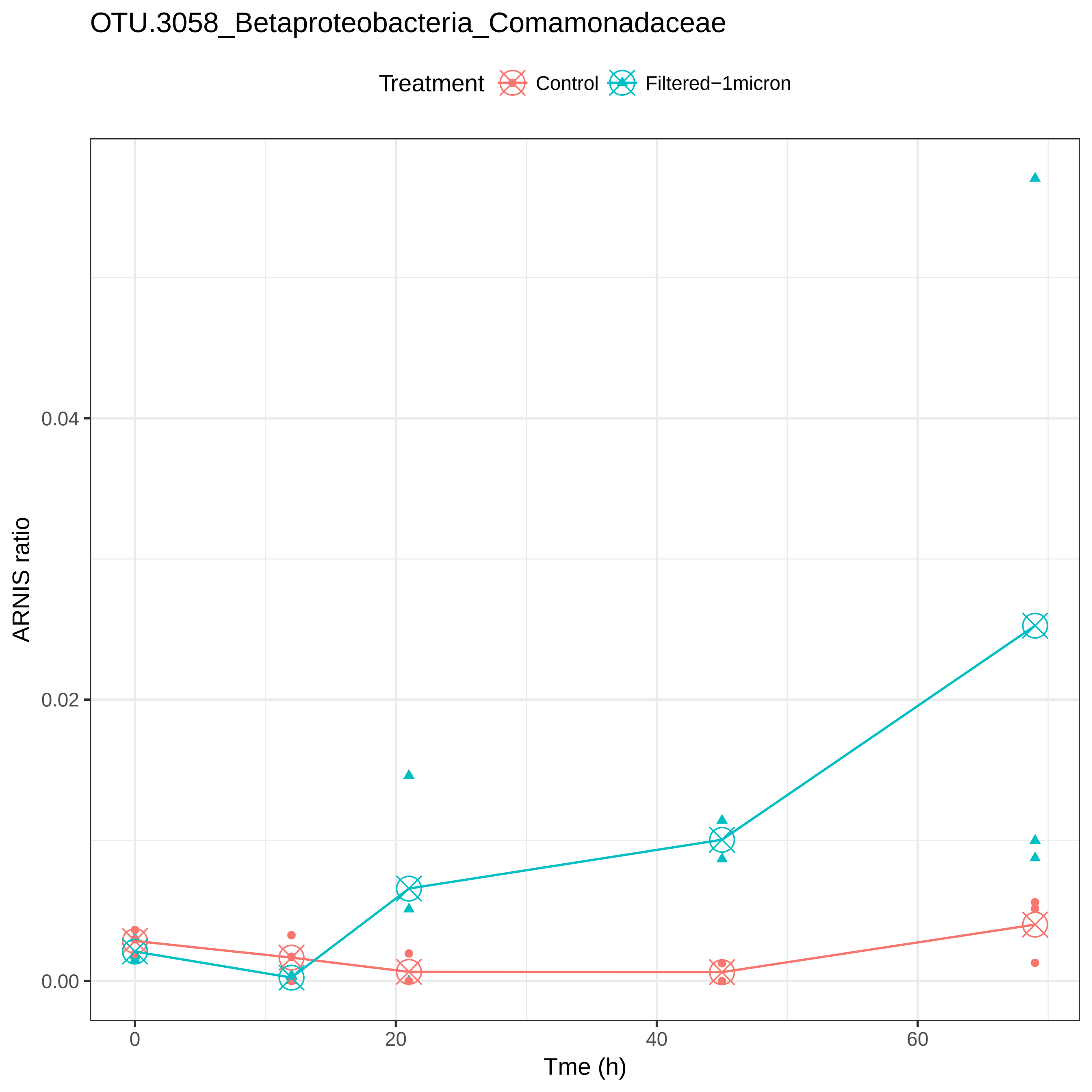
OTU.3058_Betaproteobacteria_Comamonadaceae
Treatment
Control
Filtered−1micron
ARNIS ratio
0.04
0.02
0.00
0
20
40
60
Tme (h)

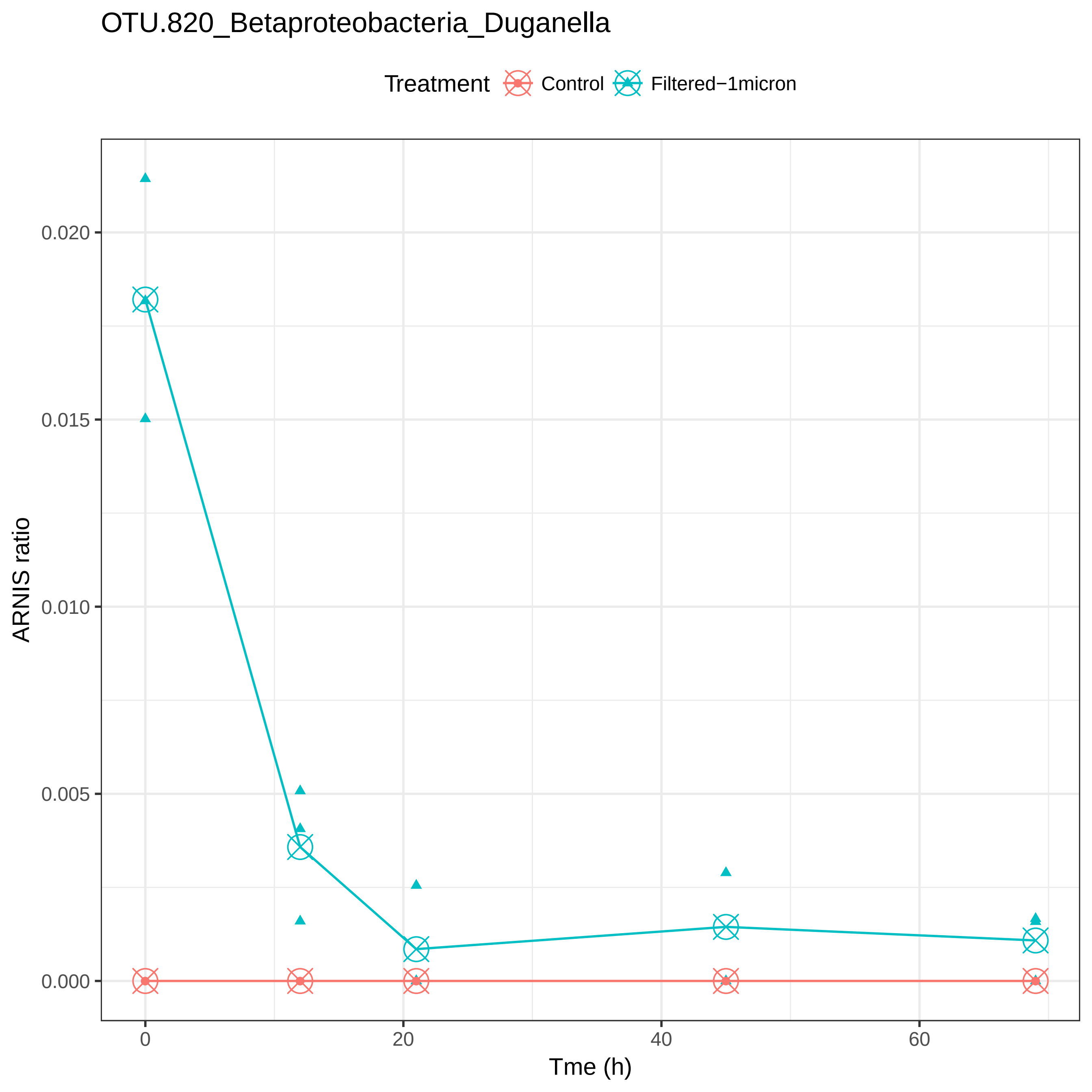

OTU.820_Betaproteobacteria_Duganella
Treatment
Control
Filtered−1micron
ARNIS ratio
0.020
0.015
0.010
0.005
0.000
0
20
40
60
Tme (h)

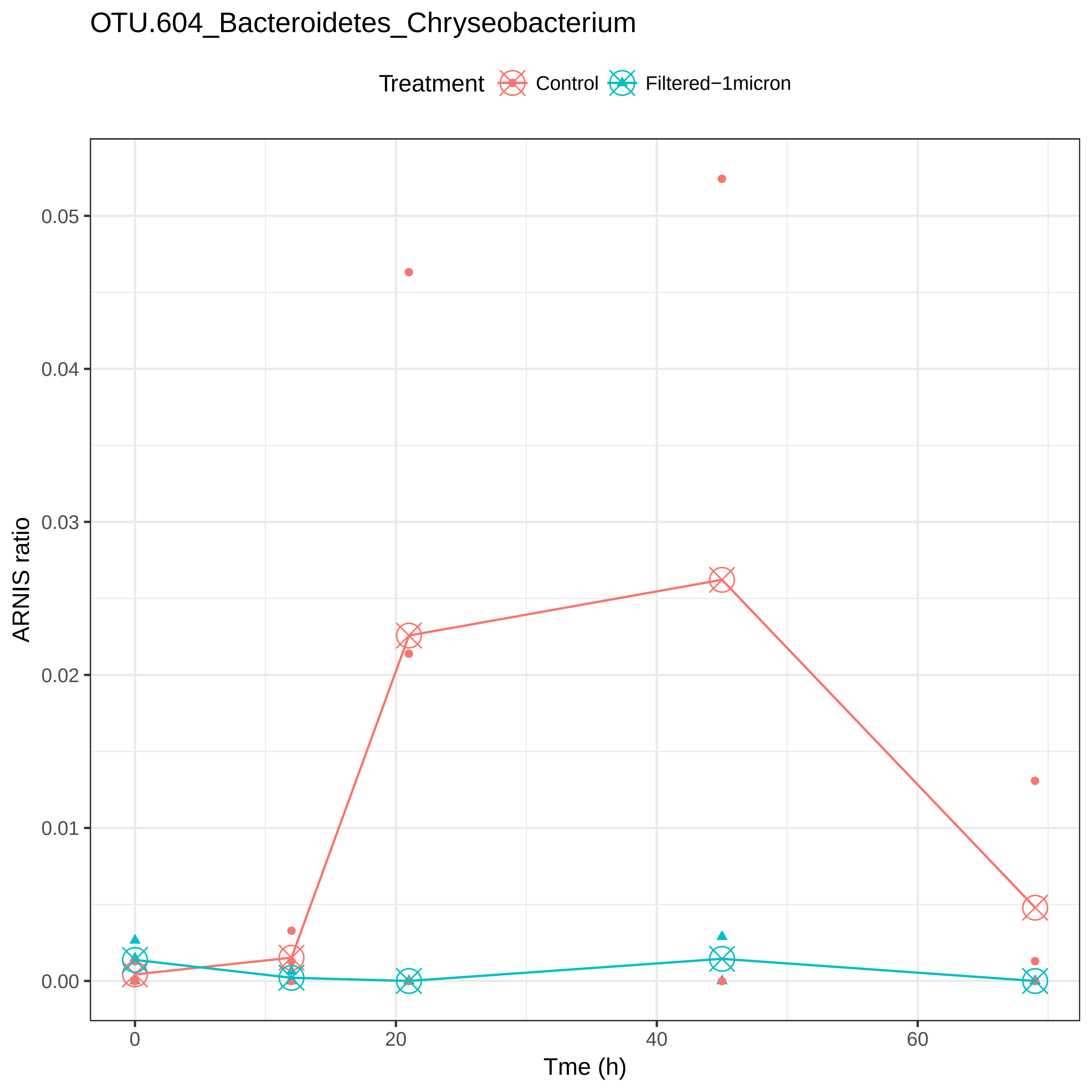

OTU.604_Bacteroidetes_Chryseobacterium
Treatment
Control
Filtered−1micron
ARNIS ratio
0.00
0.01
0.02
0.03
0.04
0.05
0
20
40
60
Tme (h)

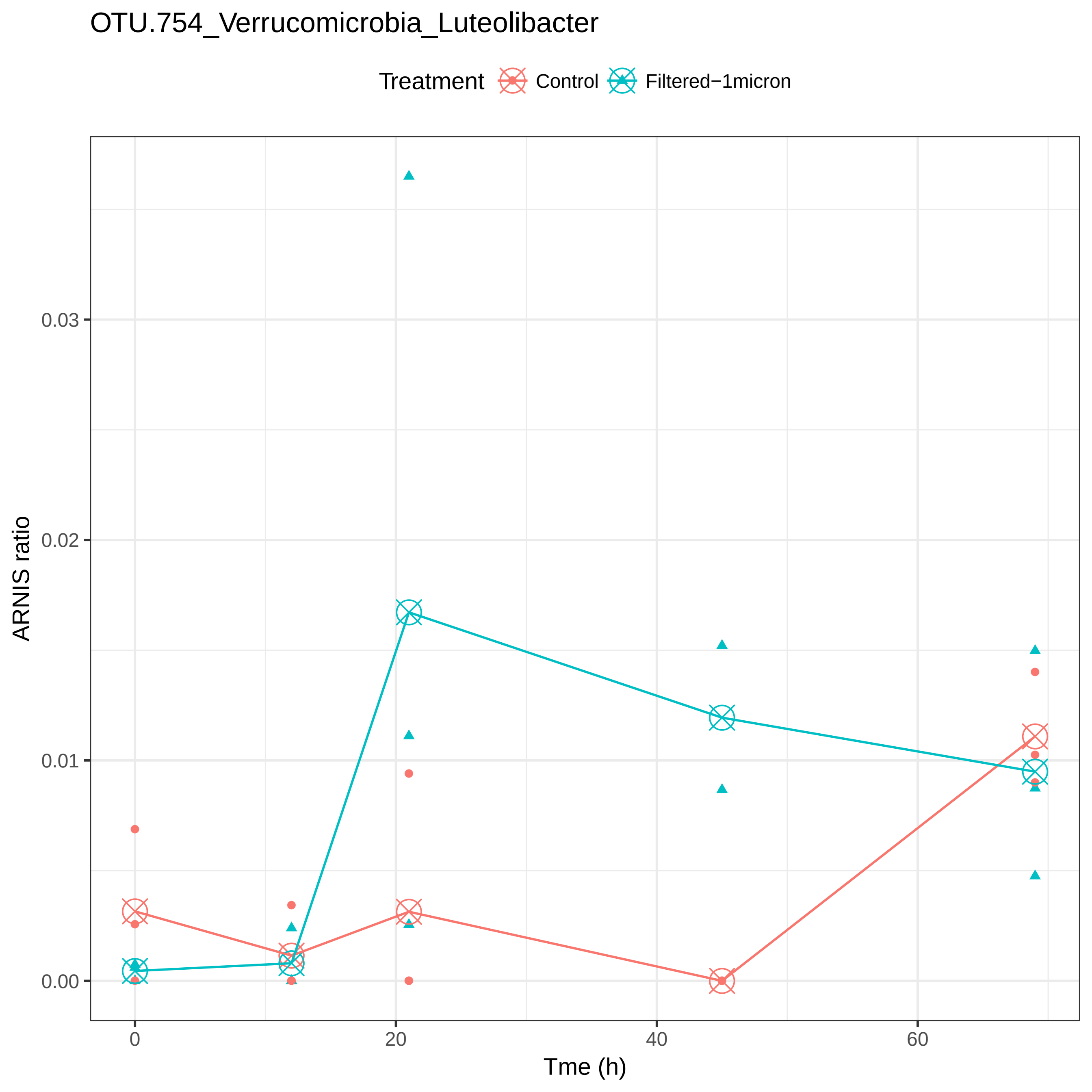
OTU.754_Verrucomicrobia_Luteolibacter
Treatment
Control
Filtered−1micron
ARNIS ratio
0.00
0.01
0.02
0.03
0
20
40
60
Tme (h)

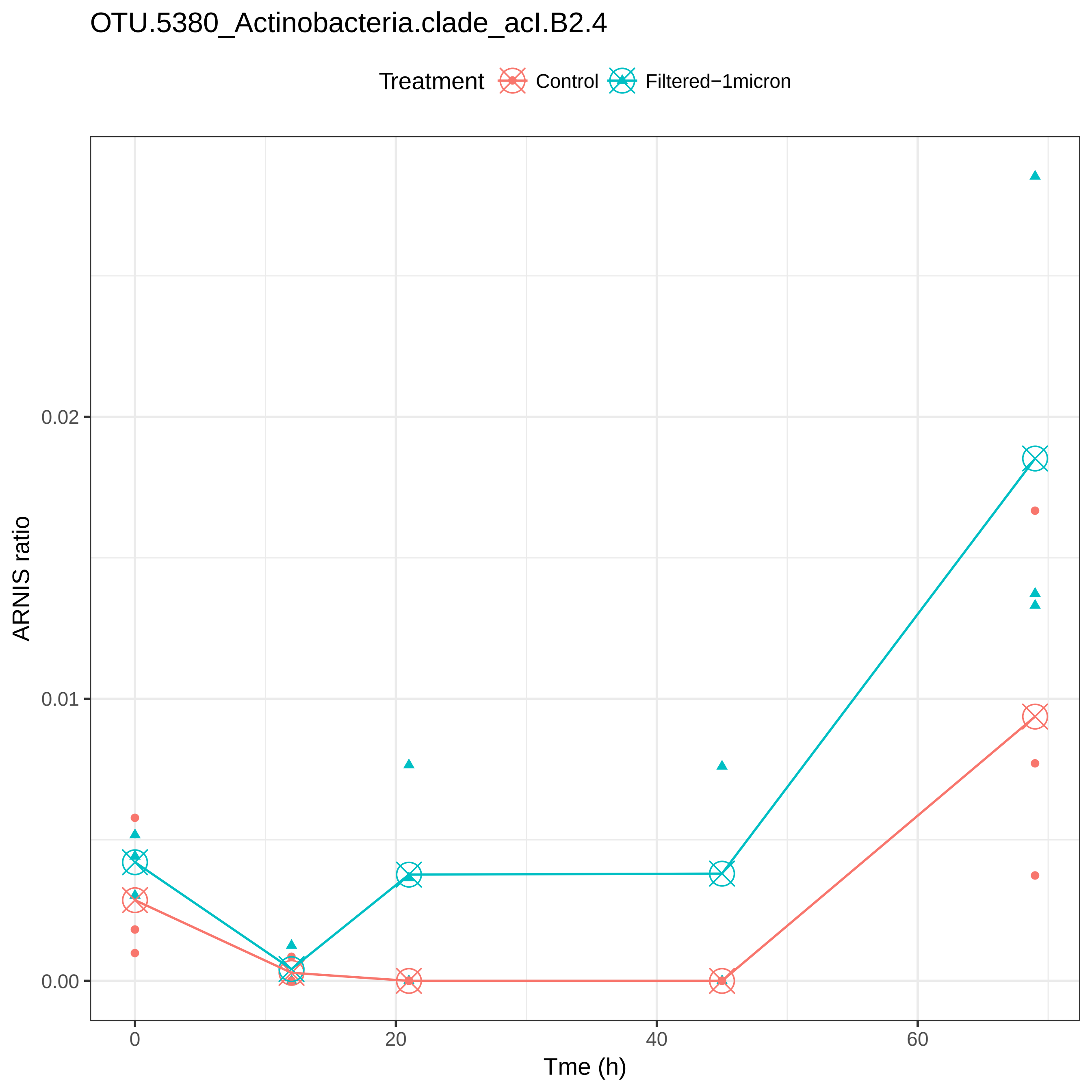

OTU.5380_Actinobacteria.clade_acI.B2.4
Treatment
Control
Filtered−1micron
ARNIS ratio
0.02
0.01
0.00
0
20
40
60
Tme (h)

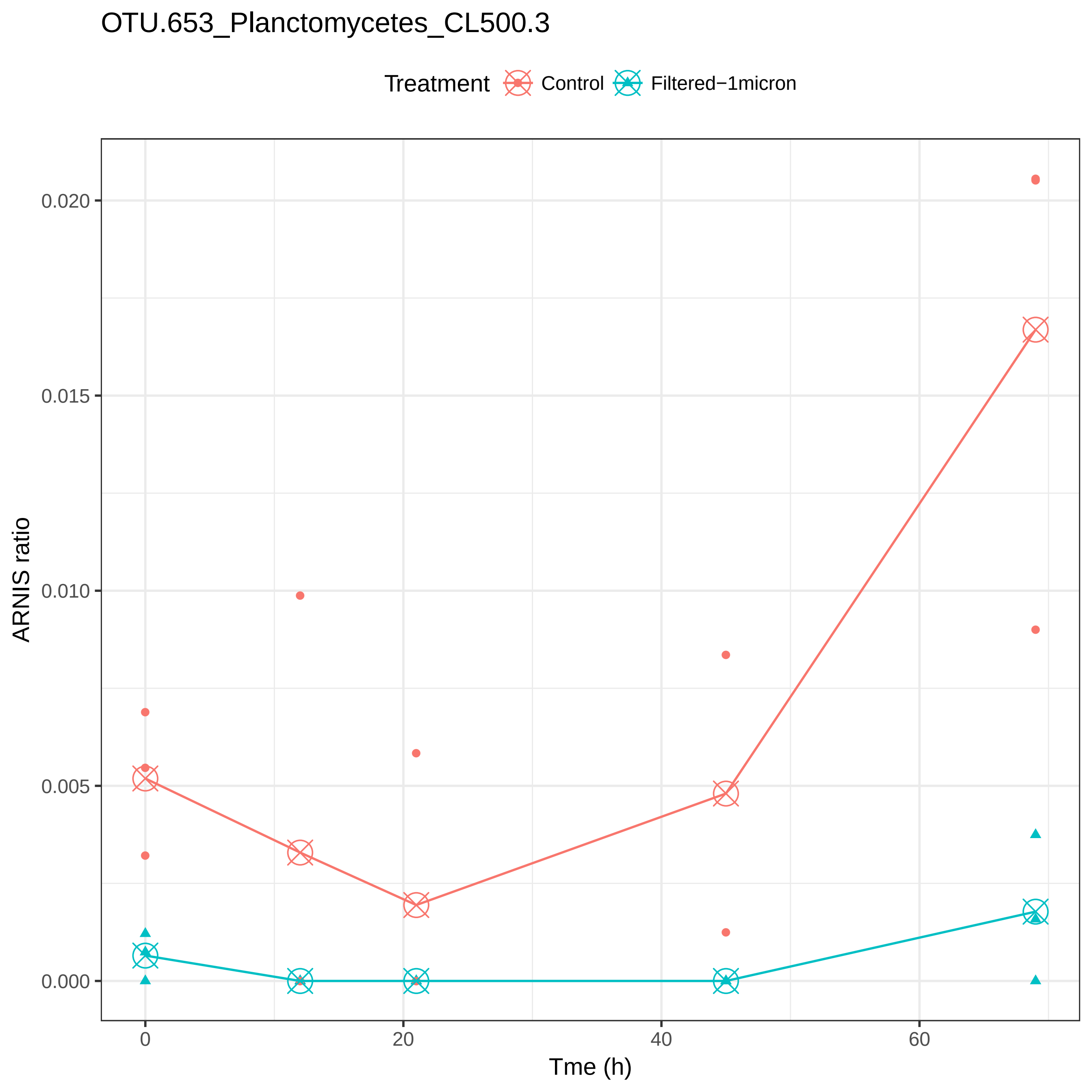

OTU.653_Planctomycetes_CL500.3
Treatment
Control
Filtered−1micron
0.020
0.015
0.010
0.005
0.000
ARNIS ratio
0
20
40
60
Tme (h)

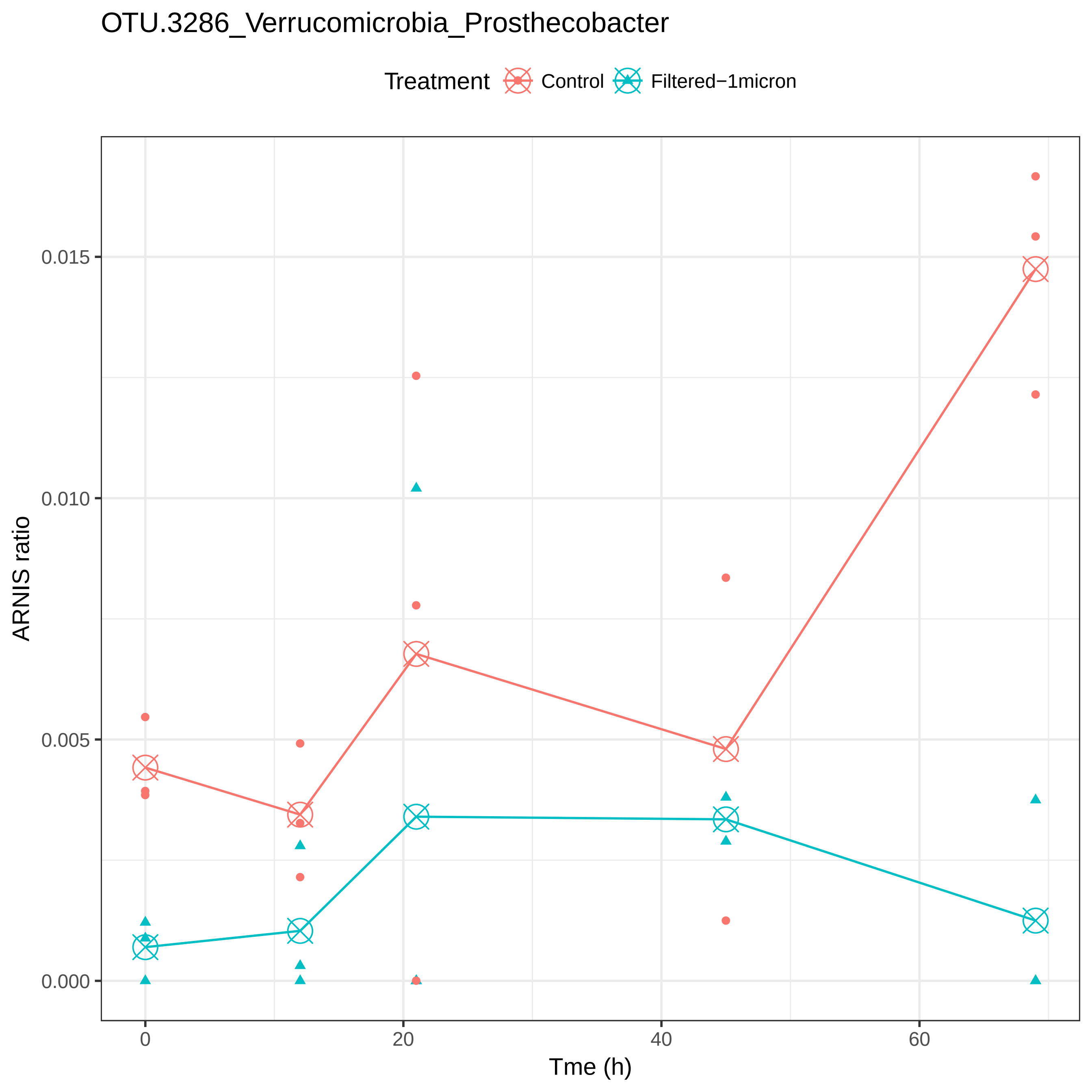

OTU.3286_Verrucomicrobia_Prosthecobacter
Treatment
Control
Filtered−1micron
ARNIS ratio
0.015
0.010
0.005
0.000
0
20
40
60
Tme (h)

OTU.726_Bacteroidetes_Sphingobacteriaceae

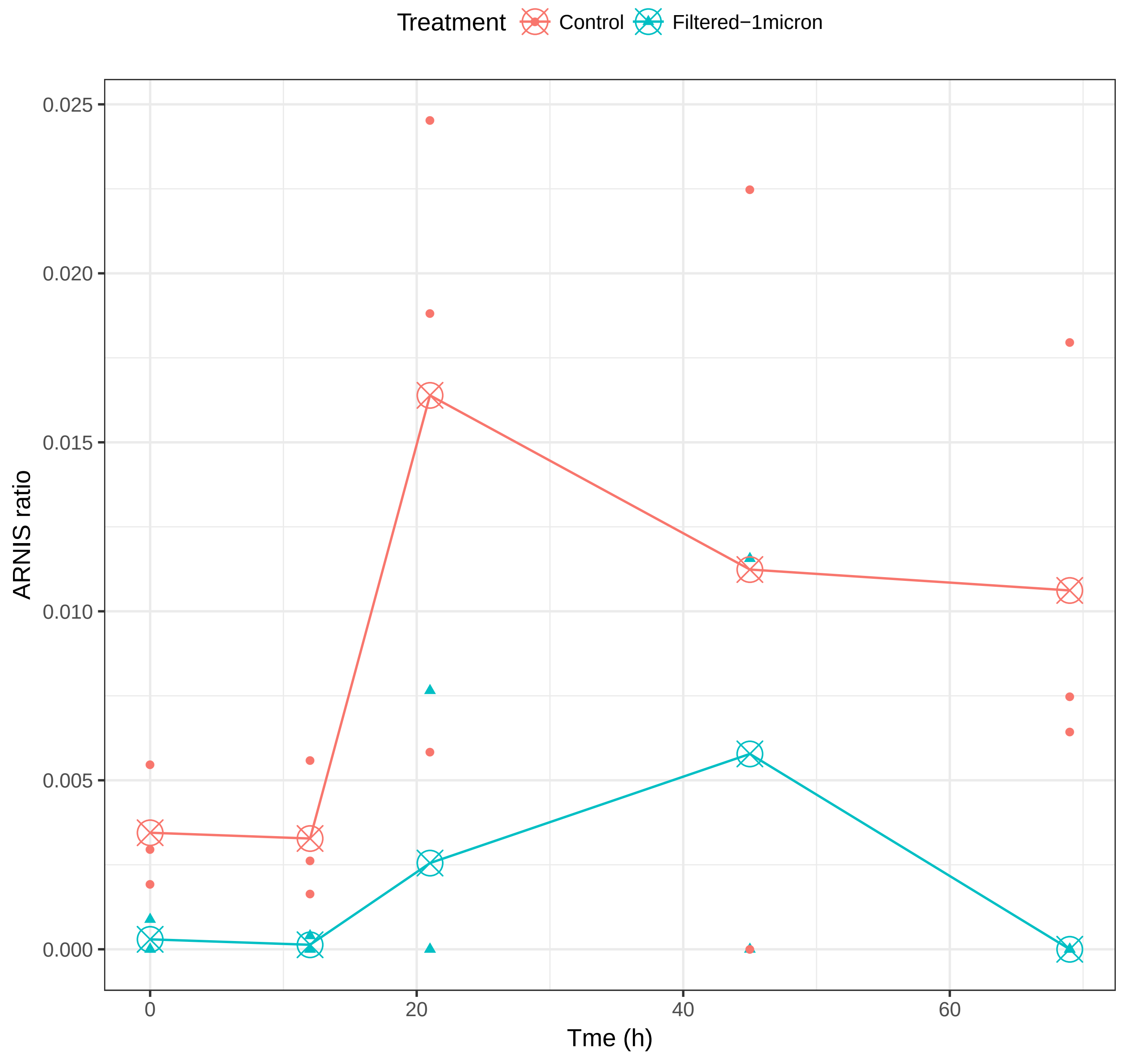

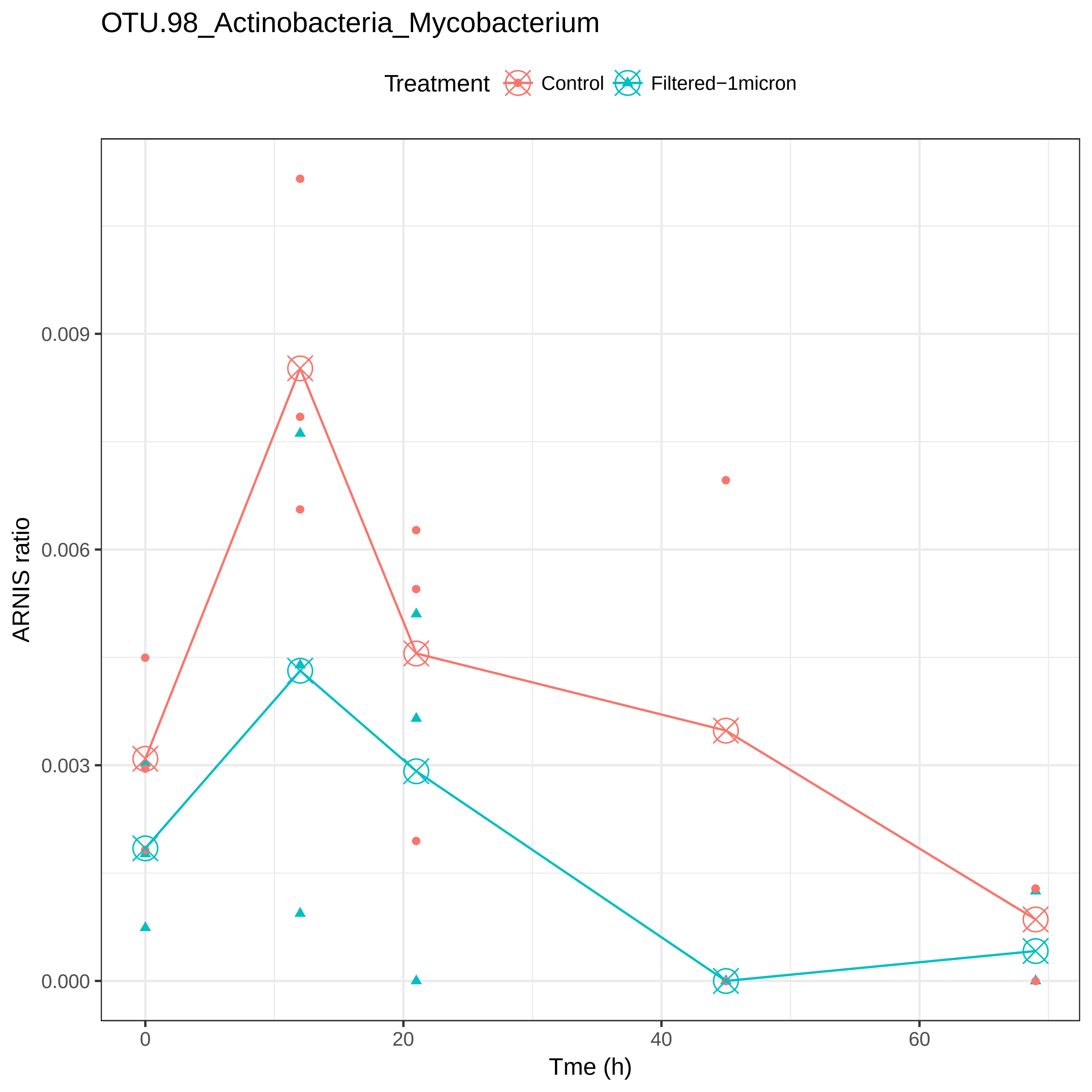
OTU.98_Actinobacteria_Mycobacterium
Treatment
Control
Filtered−1micron
ARNIS ratio
0.009
0.006
0.003
0.000
0
20
40
60
Tme (h)

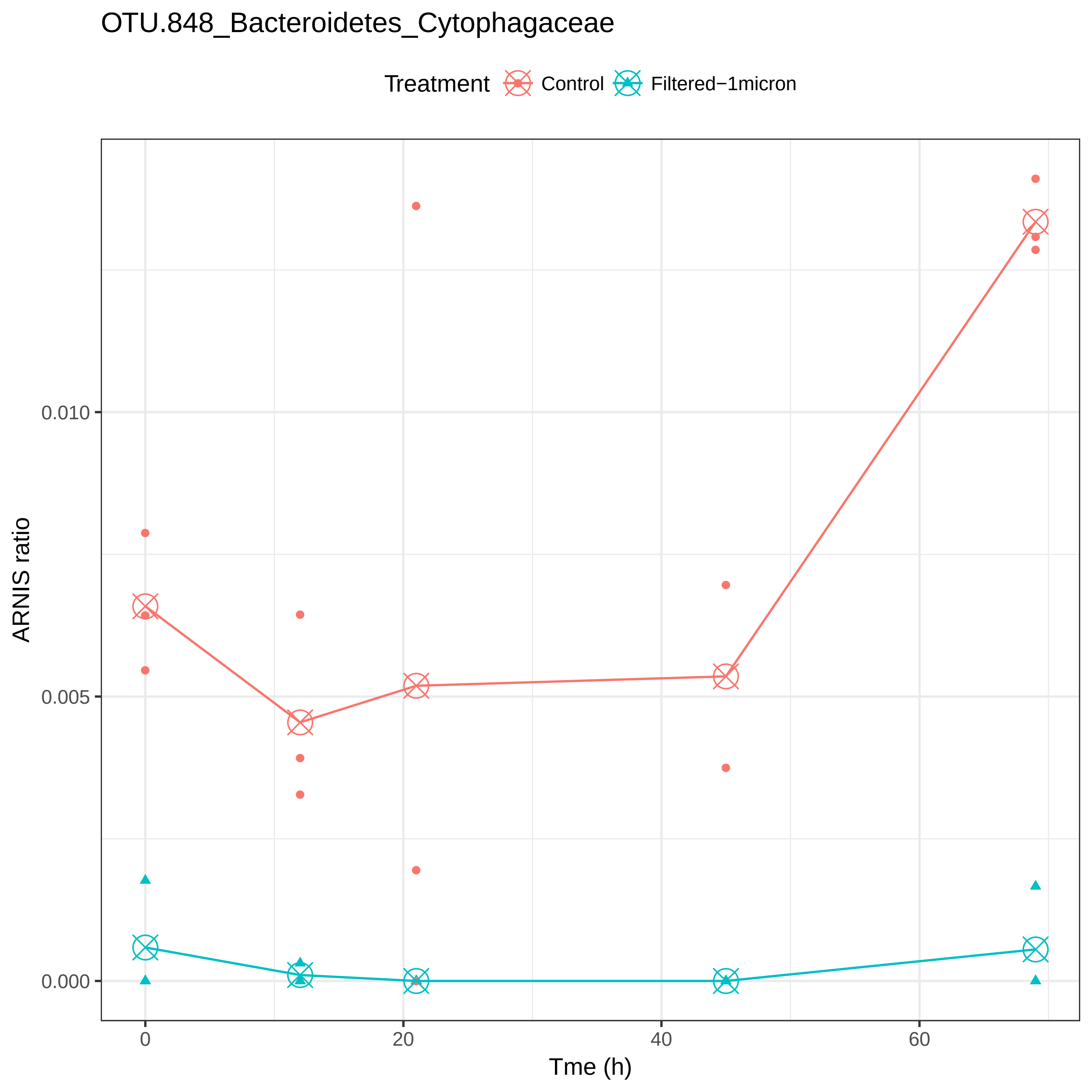

OTU.848_Bacteroidetes_Cytophagaceae
Treatment
Control
Filtered−1micron
ARNIS ratio
0.010
0.005
0.000
0
20
40
60
Tme (h)

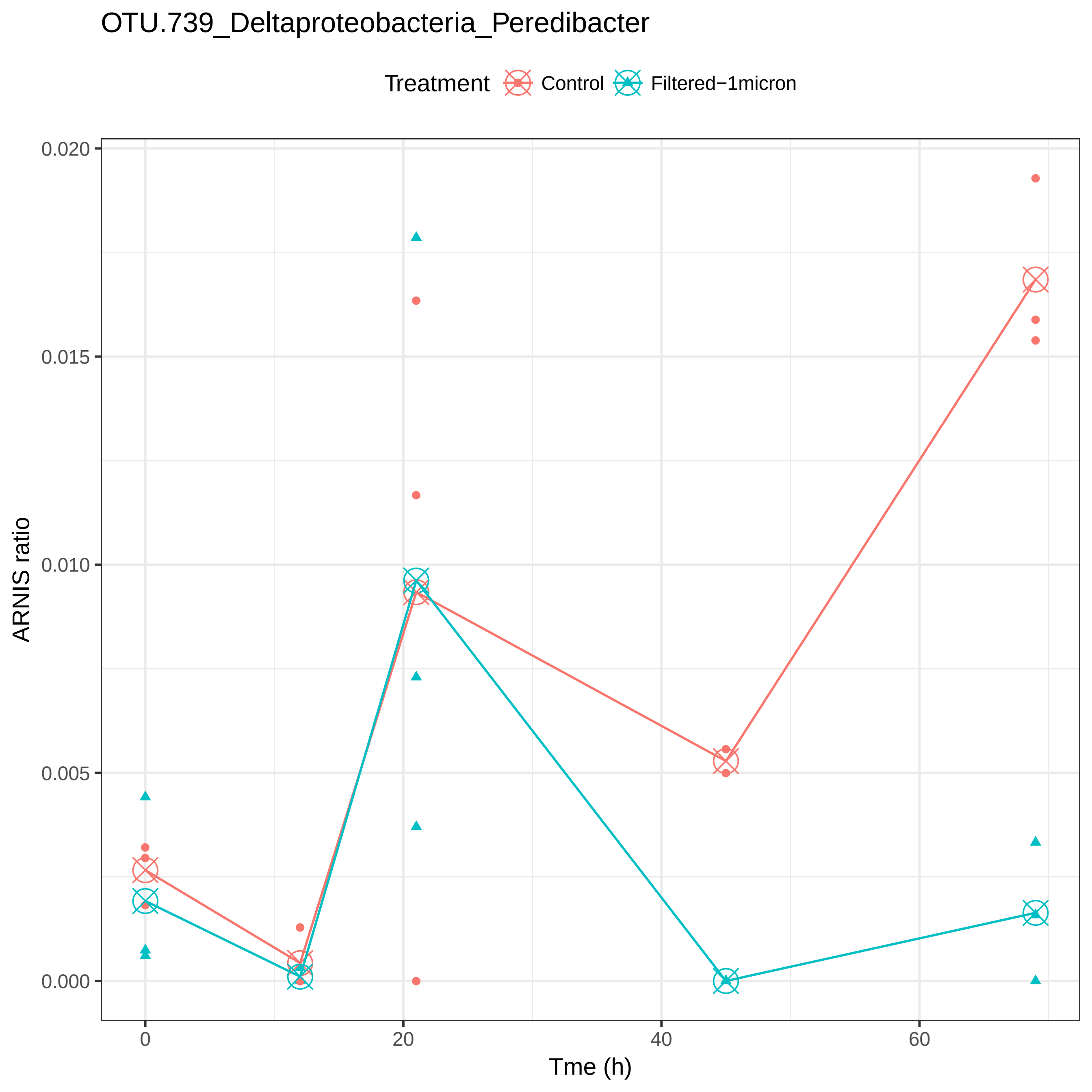
OTU.739_Deltaproteobacteria_Peredibacter
Treatment
Control
Filtered−1micron
ARNIS ratio
0.020
0.015
0.010
0.005
0.000
0
20
40
60
Tme (h)

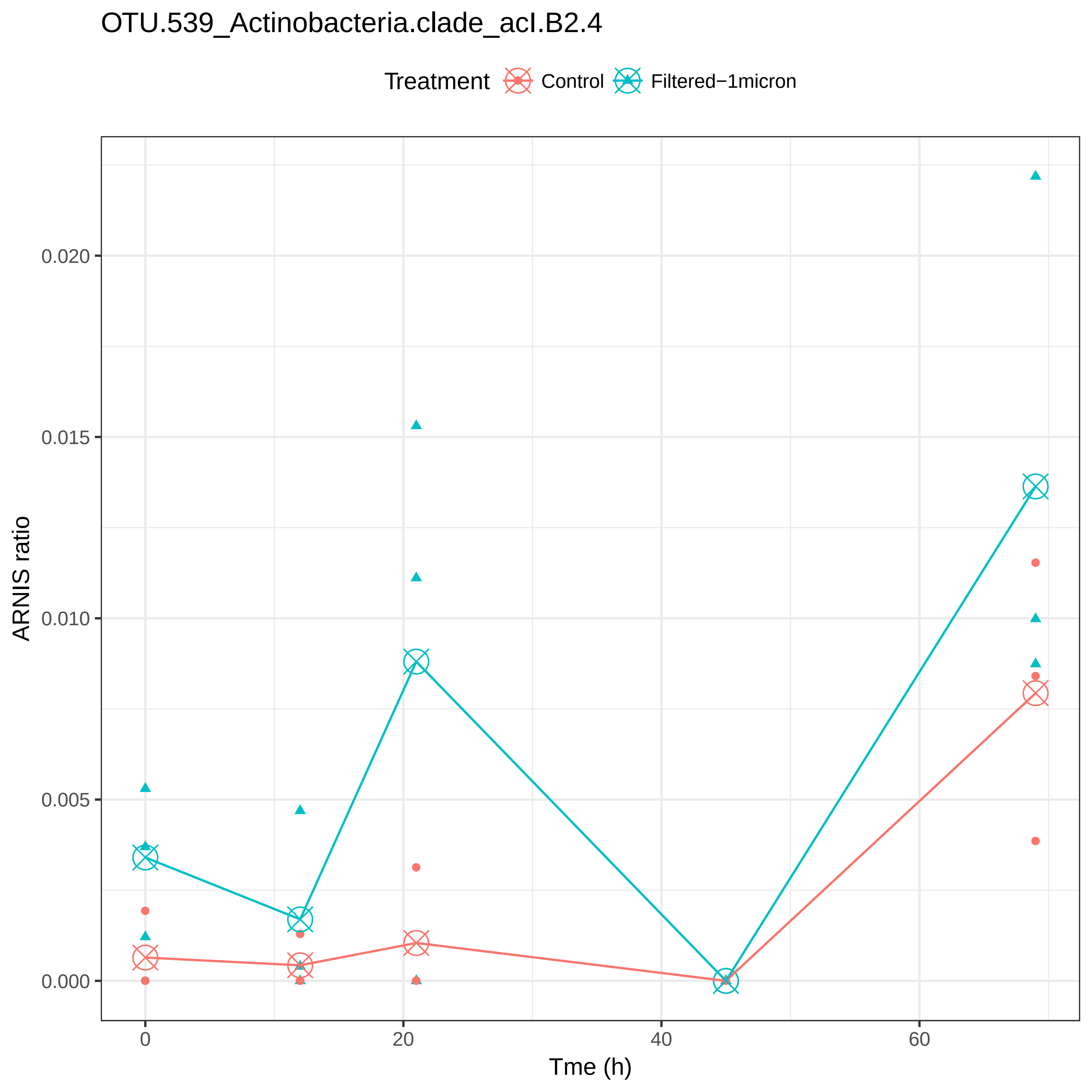

OTU.539_Actinobacteria.clade_acI.B2.4
Treatment
Control
Filtered−1micron
ARNIS ratio
0.000
0.005
0.010
0.015
0.020
0
20
40
60
Tme (h)

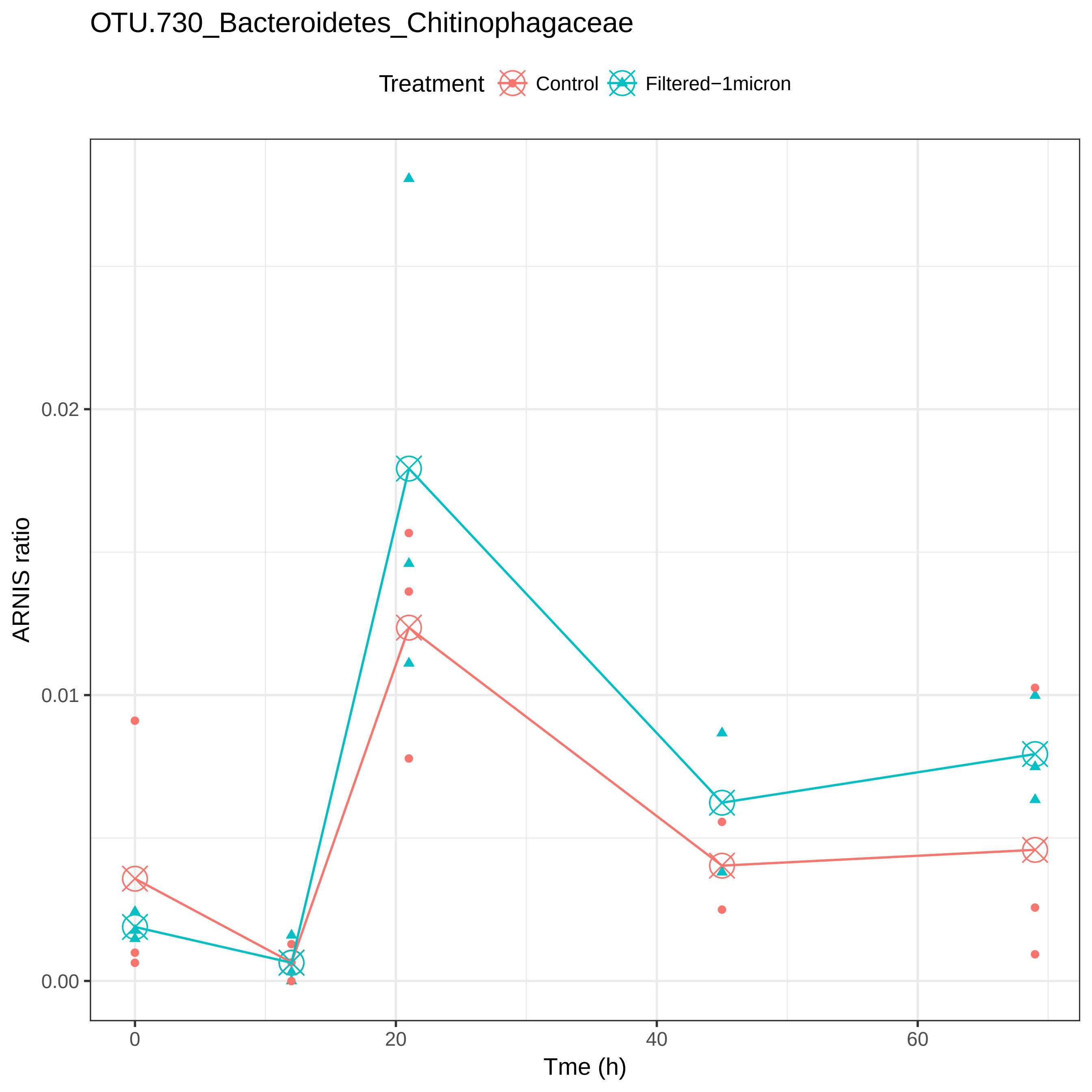

OTU.730_Bacteroidetes_Chitinophagaceae
Treatment
Control
Filtered−1micron
ARNIS ratio
0.02
0.01
0.00
0
20
40
60
Tme (h)

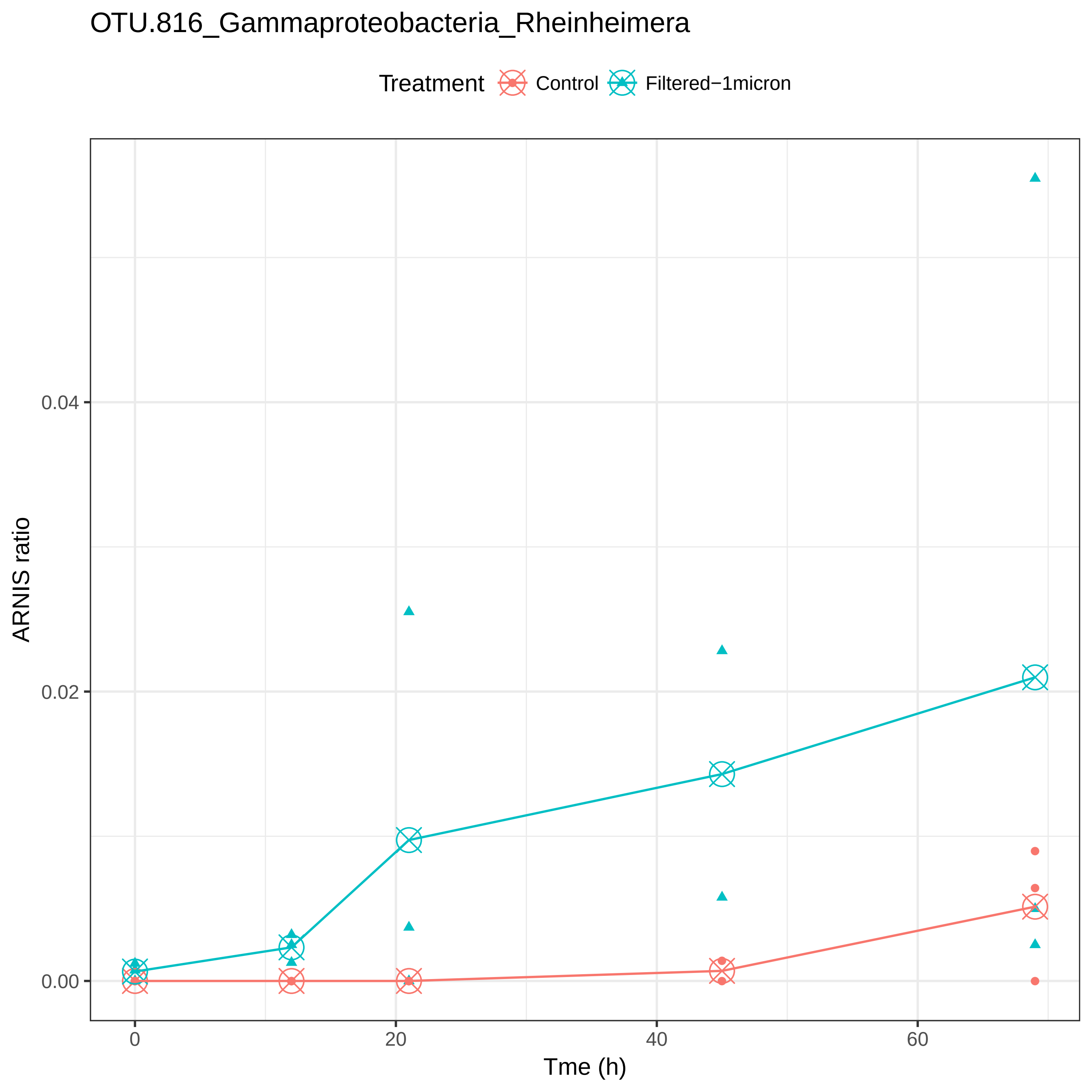

OTU.816_Gammaproteobacteria_Rheinheimera
Treatment
Control
Filtered−1micron
ARNIS ratio
0.04
0.02
0.00
0
20
40
60
Tme (h)

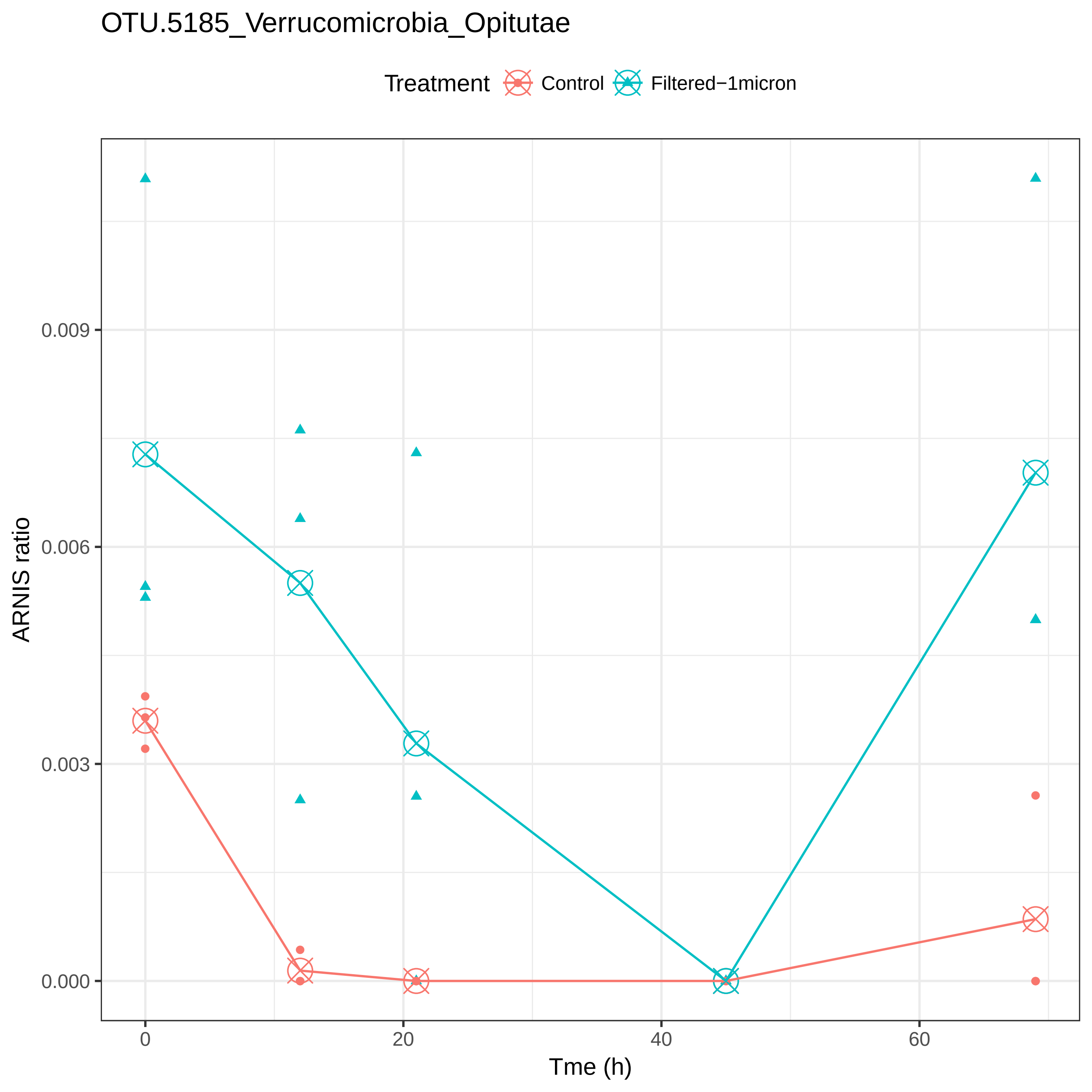
OTU.5185_Verrucomicrobia_Opitutae
Treatment
Control
Filtered−1micron
ARNIS ratio
0.009
0.006
0.003
0.000
0
20
40
60
Tme (h)

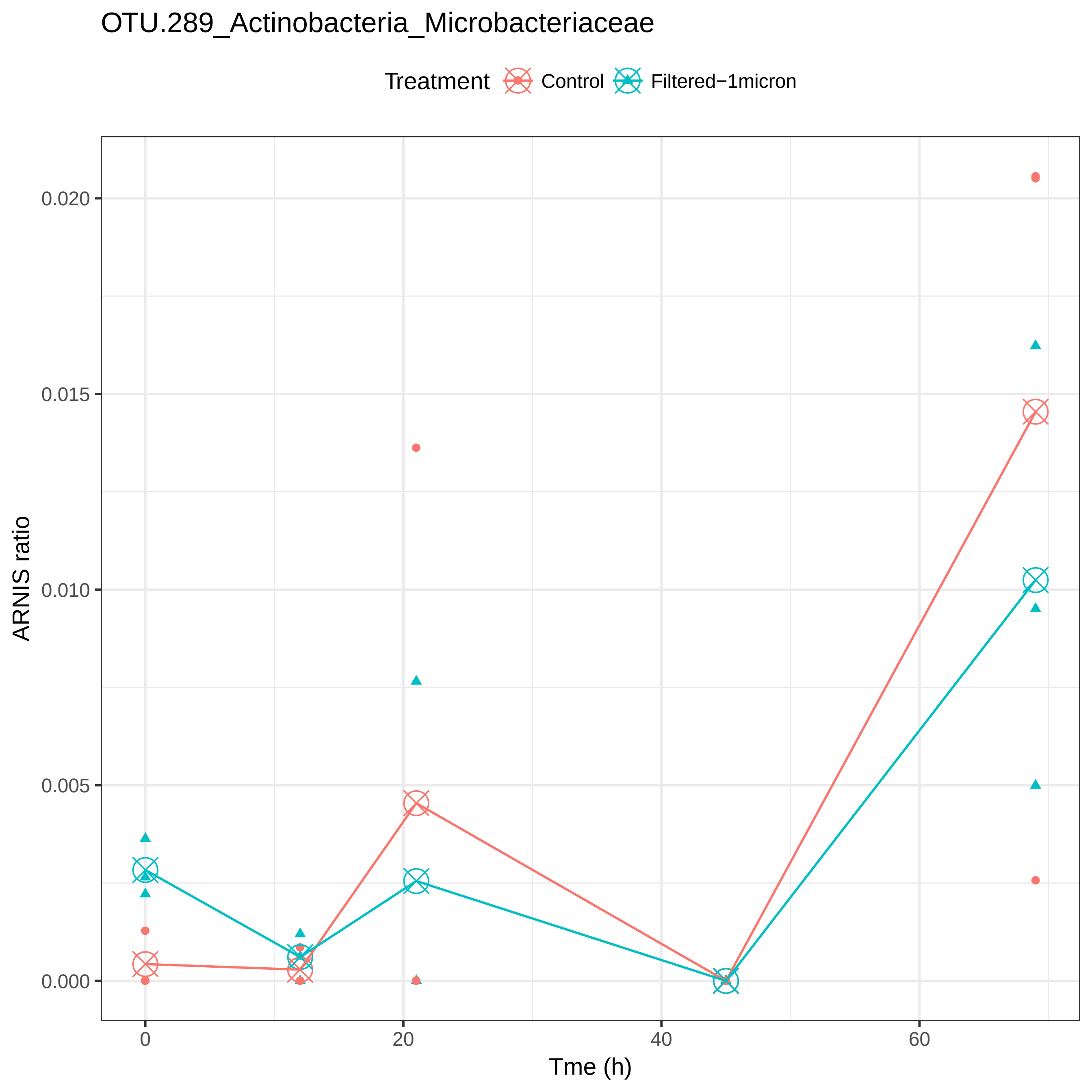
OTU.289_Actinobacteria_Microbacteriaceae
Treatment
Control
Filtered−1micron
ARNIS ratio
0.000
0.005
0.010
0.015
0.020
0
20
40
60
Tme (h)

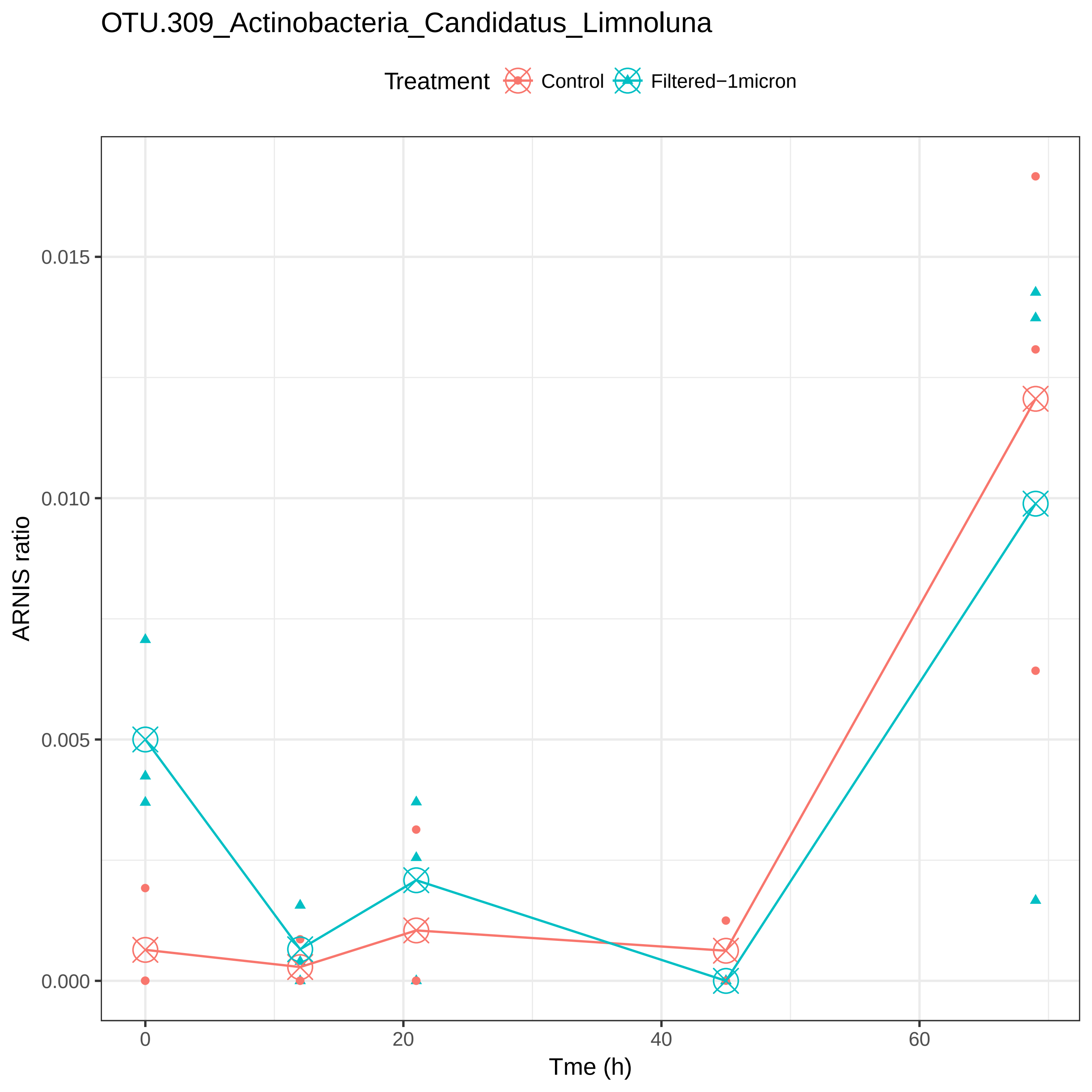

OTU.309_Actinobacteria_Candidatus_Limnoluna
Treatment
Control
Filtered−1micron
0.015
0.010
0.005
0.000
ARNIS ratio
0
20
40
60
Tme (h)

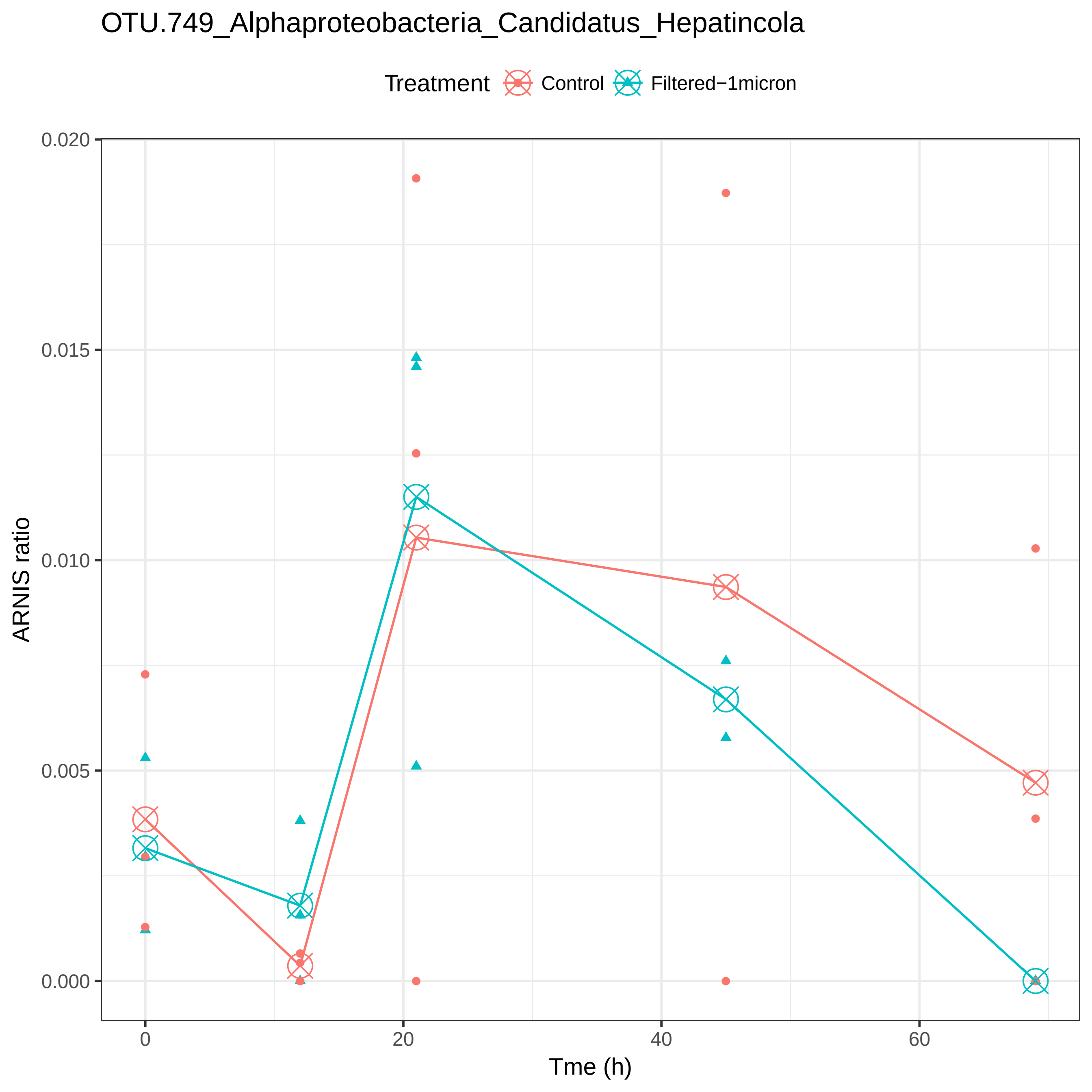

OTU.749_Alphaproteobacteria_Candidatus_Hepatincola
Treatment
Control
Filtered−1micron
ARNIS ratio
0.020
0.015
0.010
0.005
0.000
0
20
40
60
Tme (h)

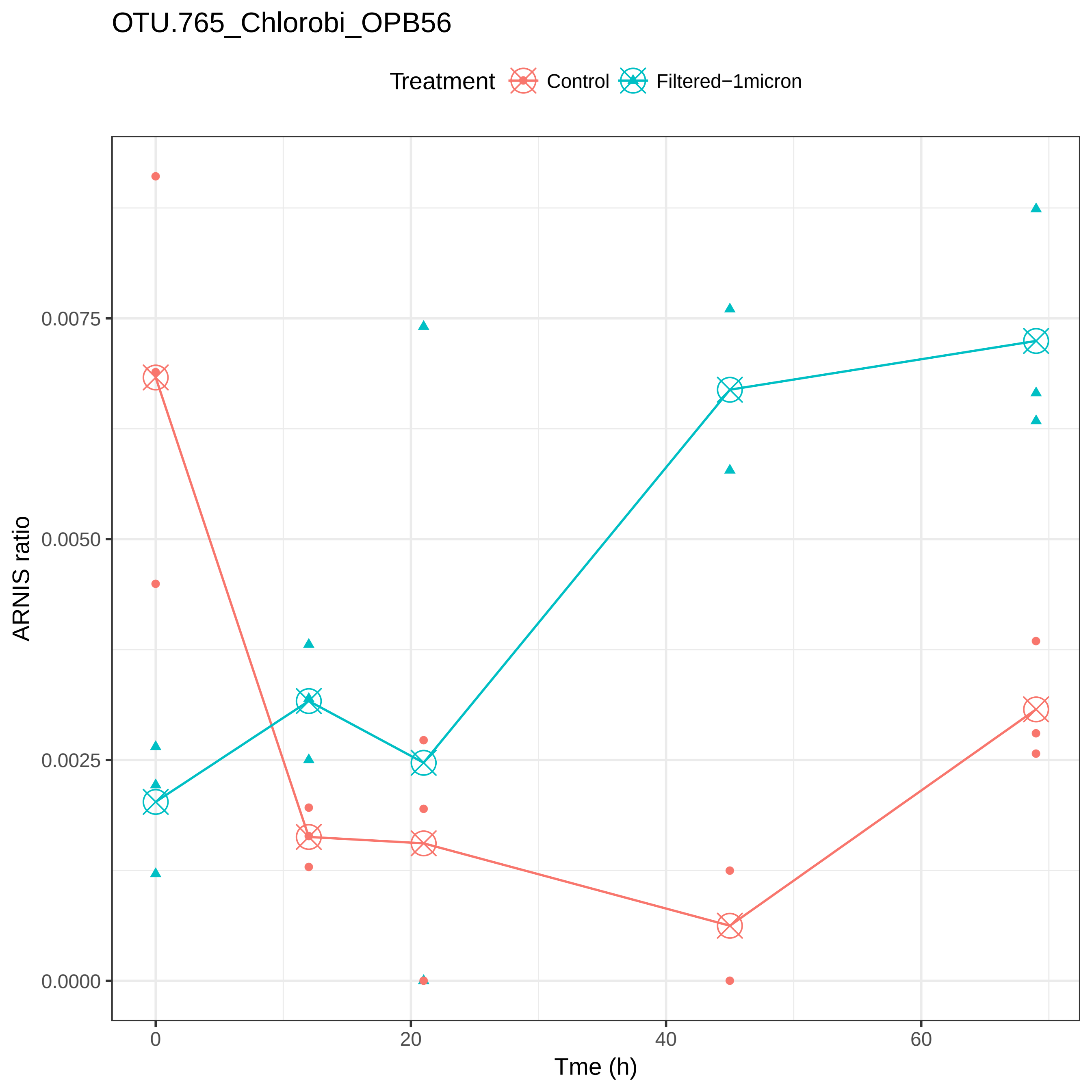

OTU.765_Chlorobi_OPB56
Treatment
Control
Filtered−1micron
ARNIS ratio
0.0000
0.0025
0.0050
0.0075
0
20
40
60
Tme (h)

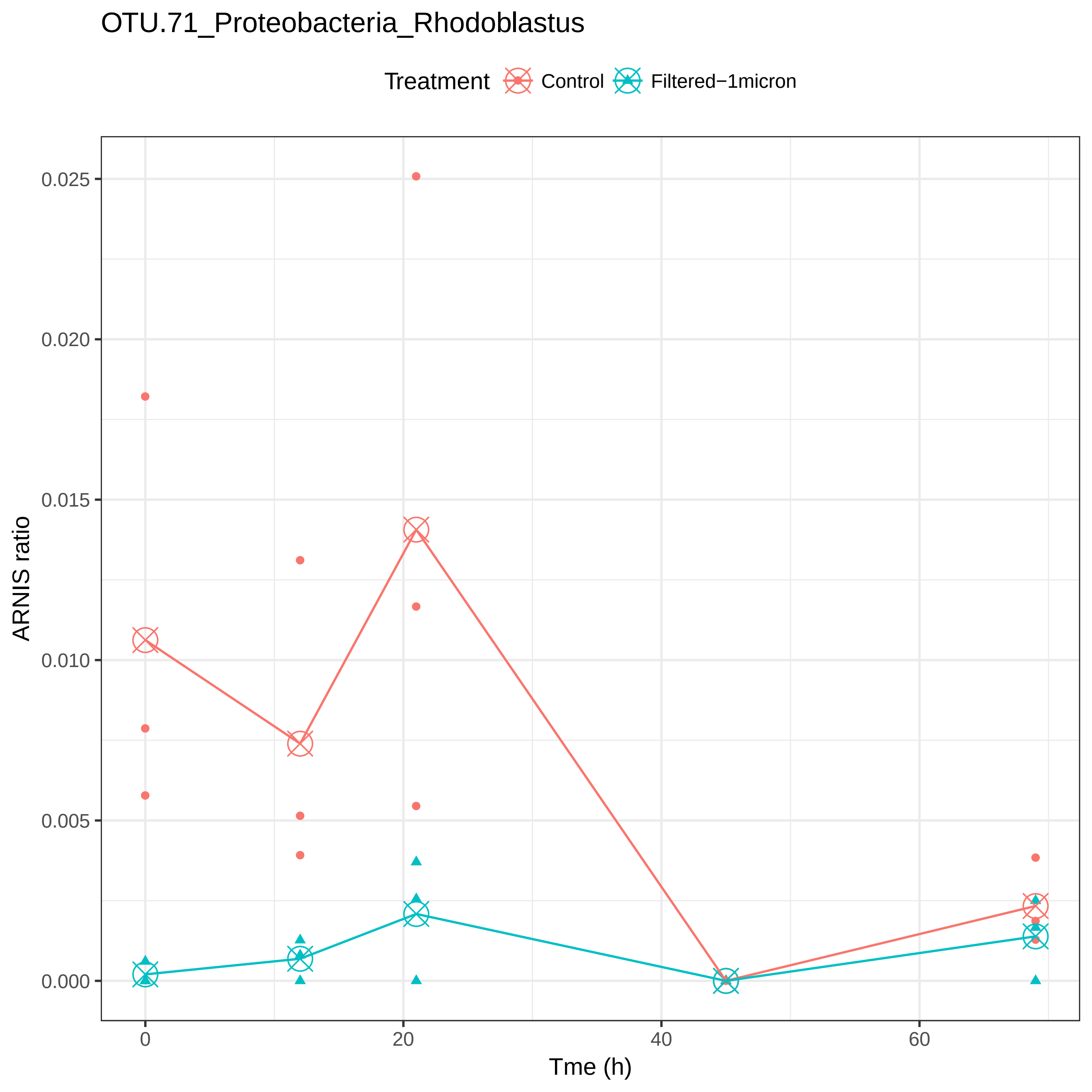
OTU.71_Proteobacteria_Rhodoblastus
Treatment
Control
Filtered−1micron
ARNIS ratio
0.025
0.020
0.015
0.010
0.005
0.000
0
20
40
60
Tme (h)

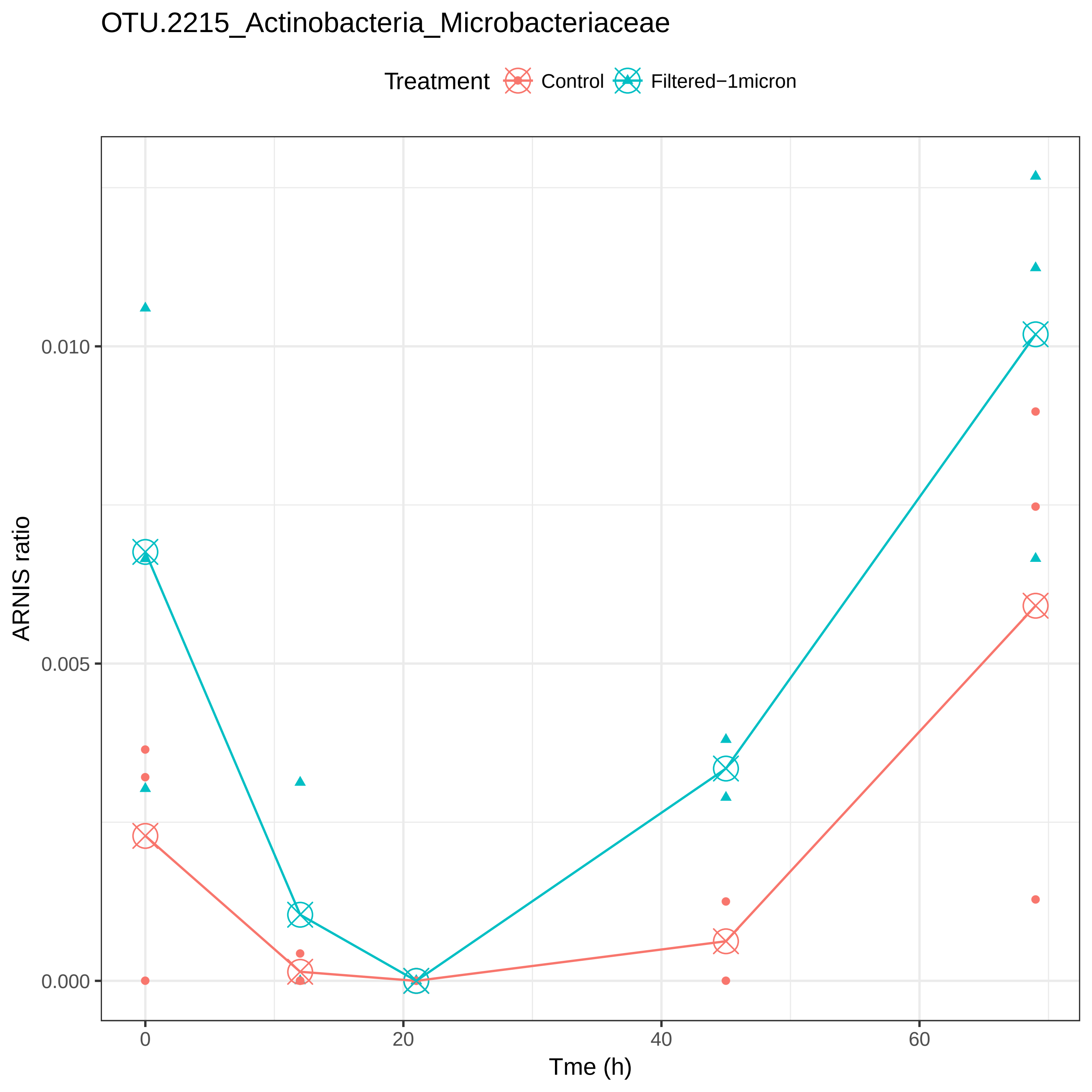
OTU.2215_Actinobacteria_Microbacteriaceae
Treatment
Control
Filtered−1micron
ARNIS ratio
0.010
0.005
0.000
0
20
40
60
Tme (h)

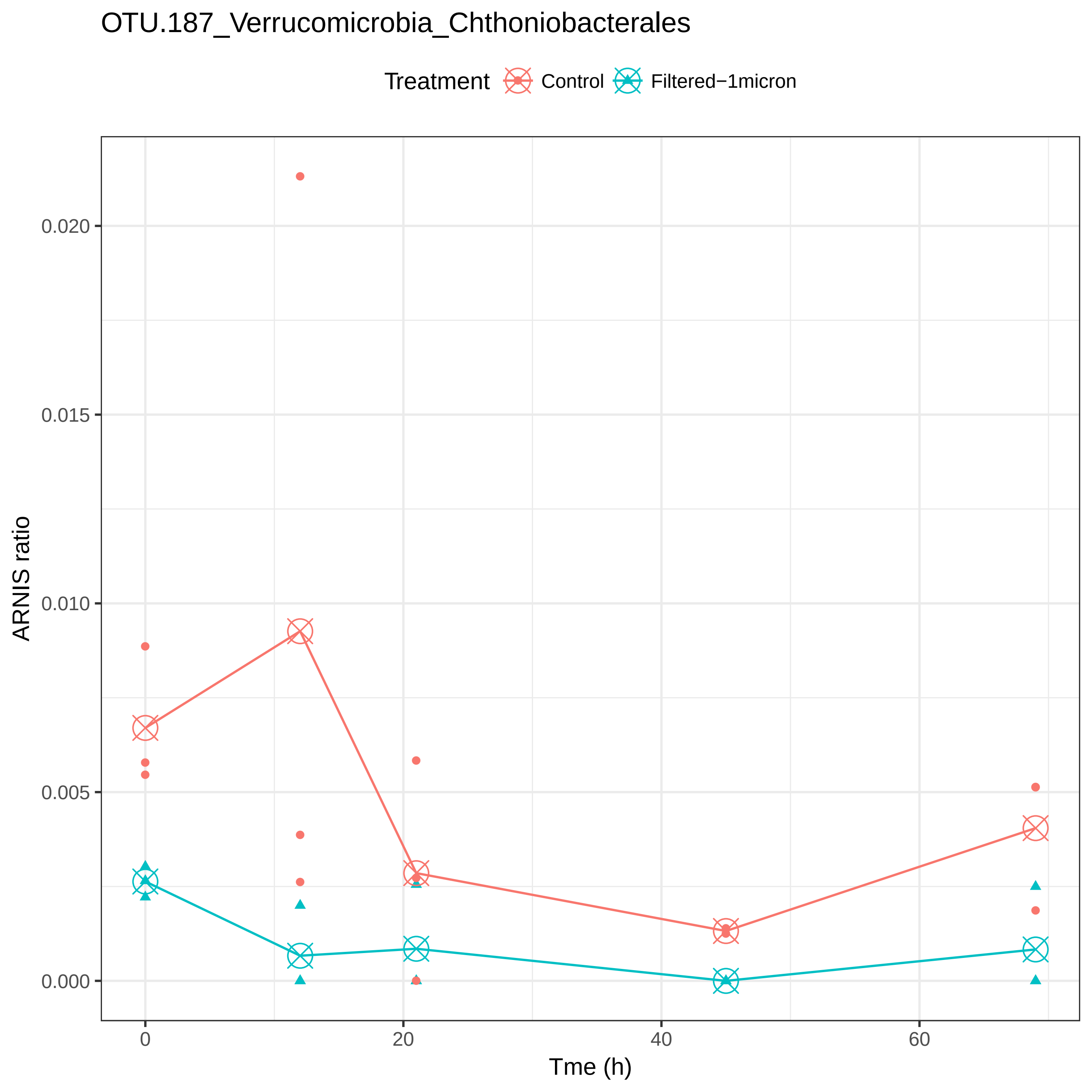

OTU.187_Verrucomicrobia_Chthoniobacterales
Treatment
Control
Filtered−1micron
ARNIS ratio
0.020
0.015
0.010
0.005
0.000
0
20
40
60
Tme (h)

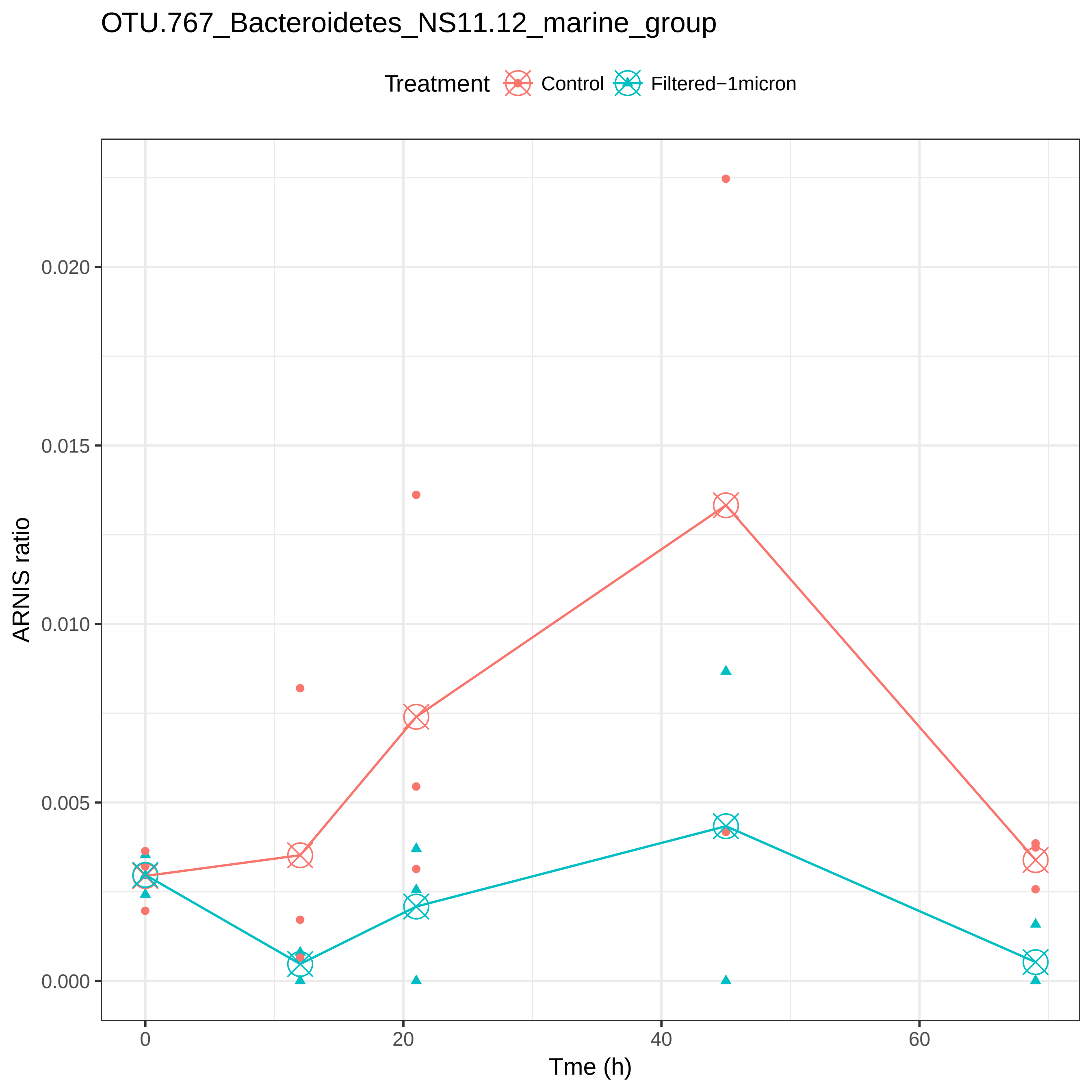

OTU.767_Bacteroidetes_NS11.12_marine_group
Treatment
Control
Filtered−1micron
ARNIS ratio
0.000
0.005
0.010
0.015
0.020
0
20
40
60
Tme (h)

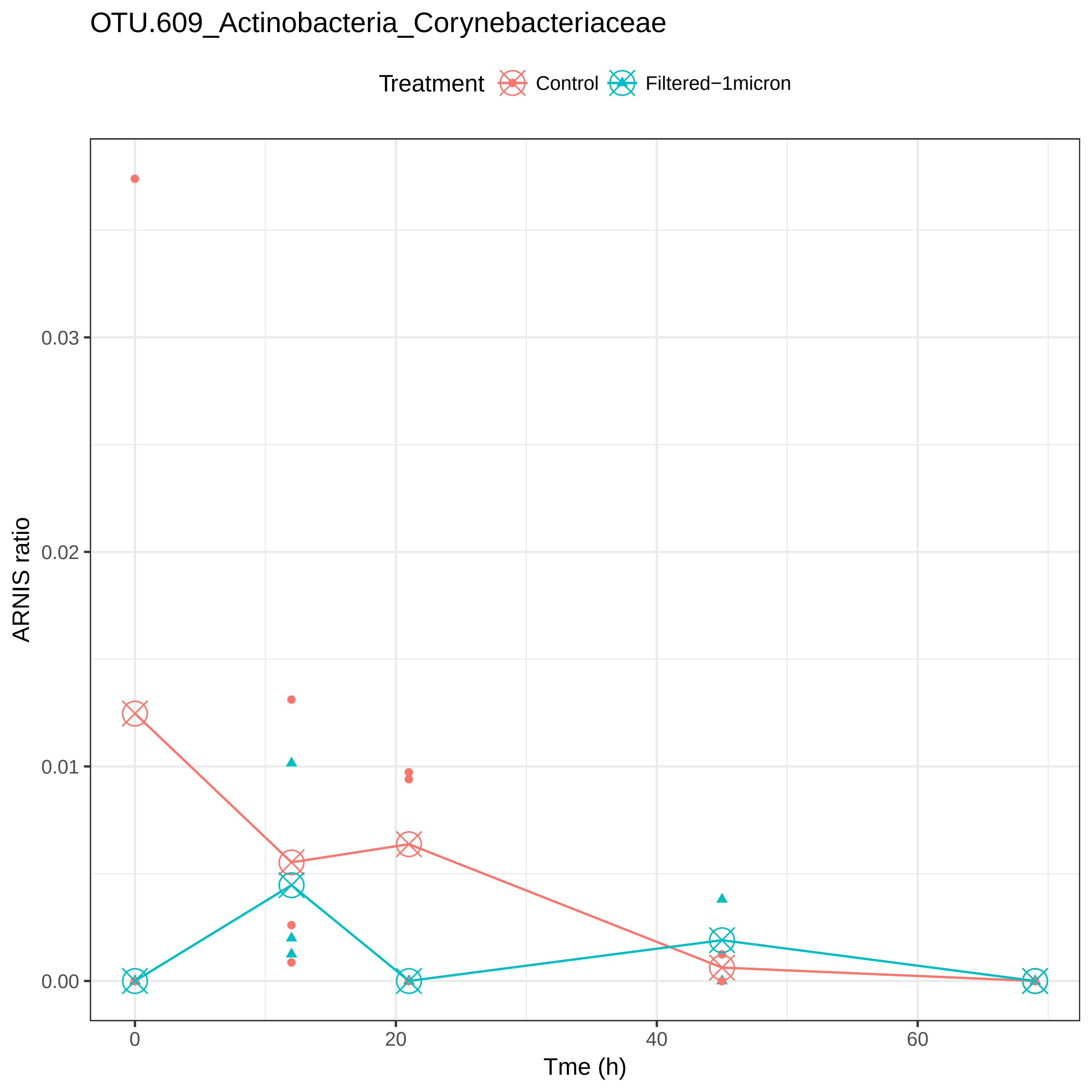

OTU.609_Actinobacteria_Corynebacteriaceae
Treatment
Control
Filtered−1micron
ARNIS ratio
0.03
0.02
0.01
0.00
0
20
40
60
Tme (h)

OTU.425_Bacteroidetes_Chitinophagaceae

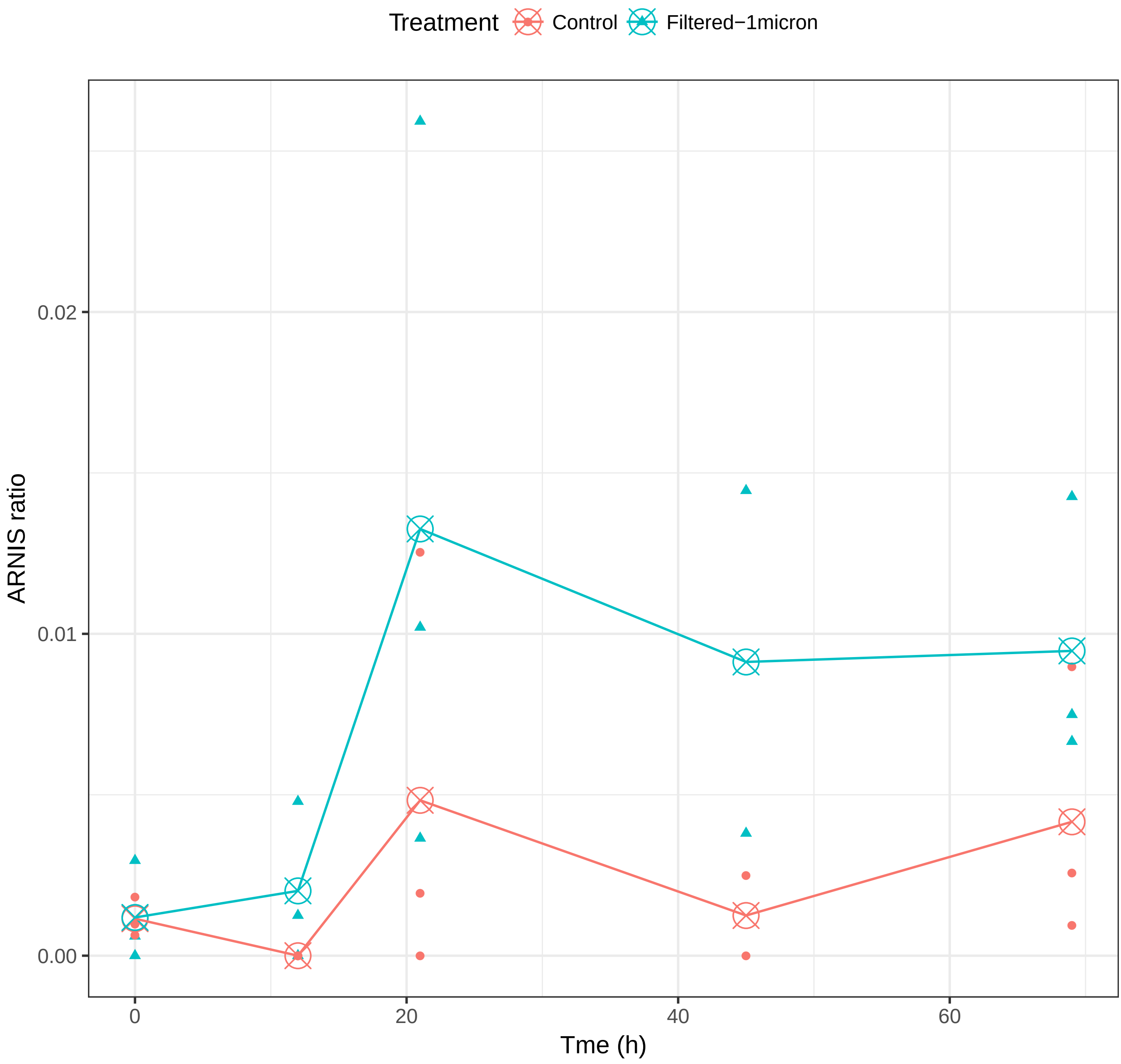

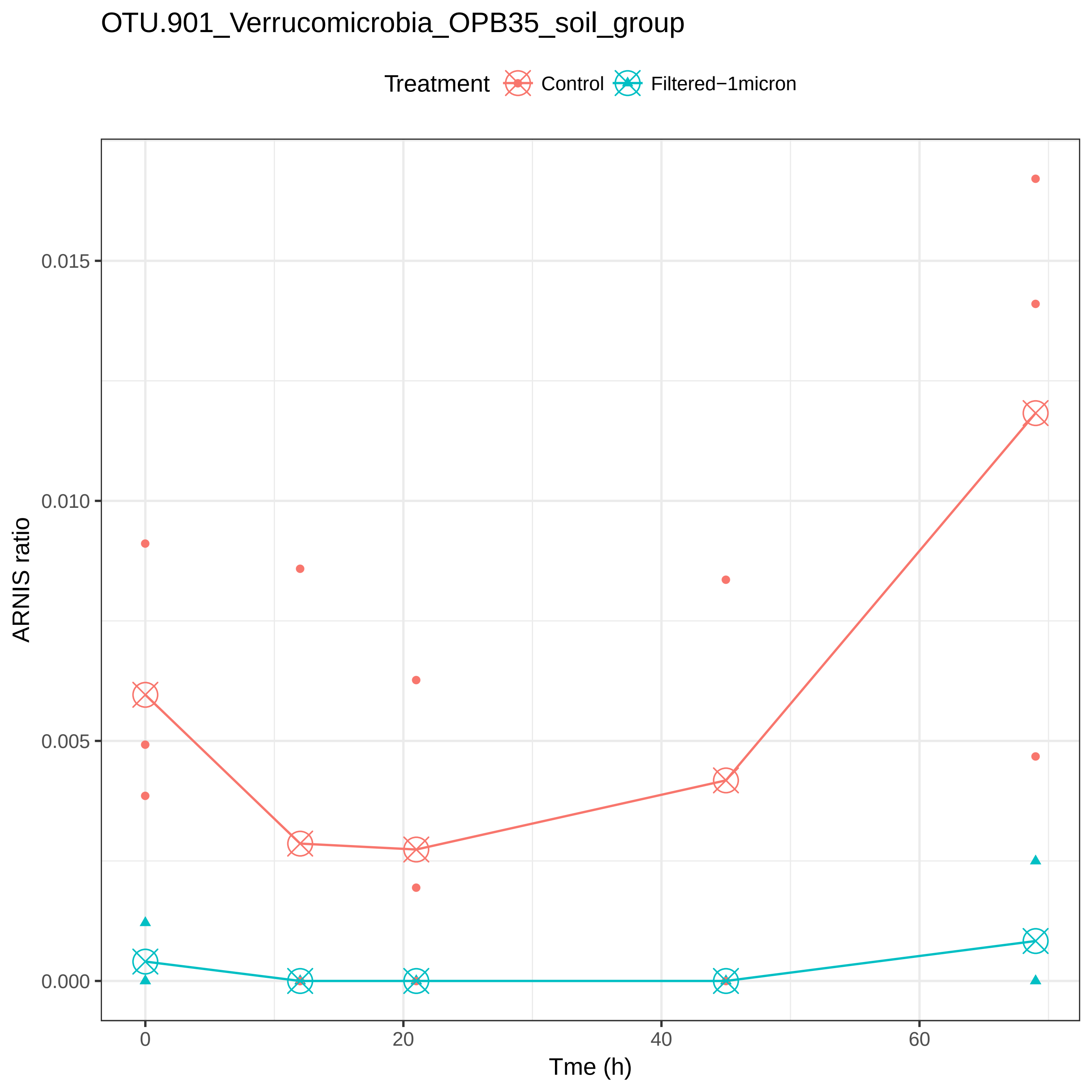
OTU.901_Verrucomicrobia_OPB35_soil_group
Treatment
Control
Filtered−1micron
ARNIS ratio
0.015
0.010
0.005
0.000
0
20
40
60
Tme (h)

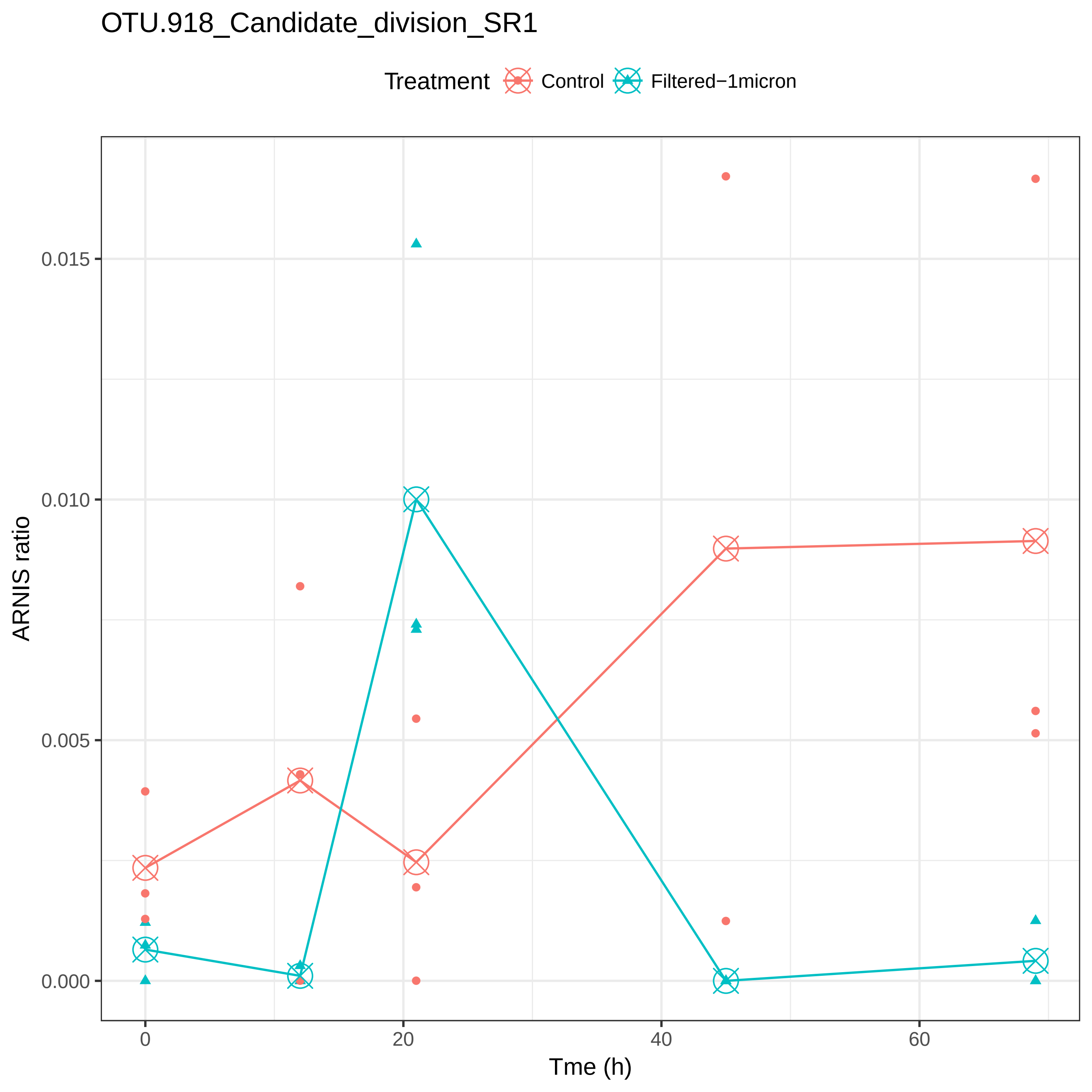

OTU.918_Candidate_division_SR1
Treatment
Control
Filtered−1micron
ARNIS ratio
0.015
0.010
0.005
0.000
0
20
40
60
Tme (h)

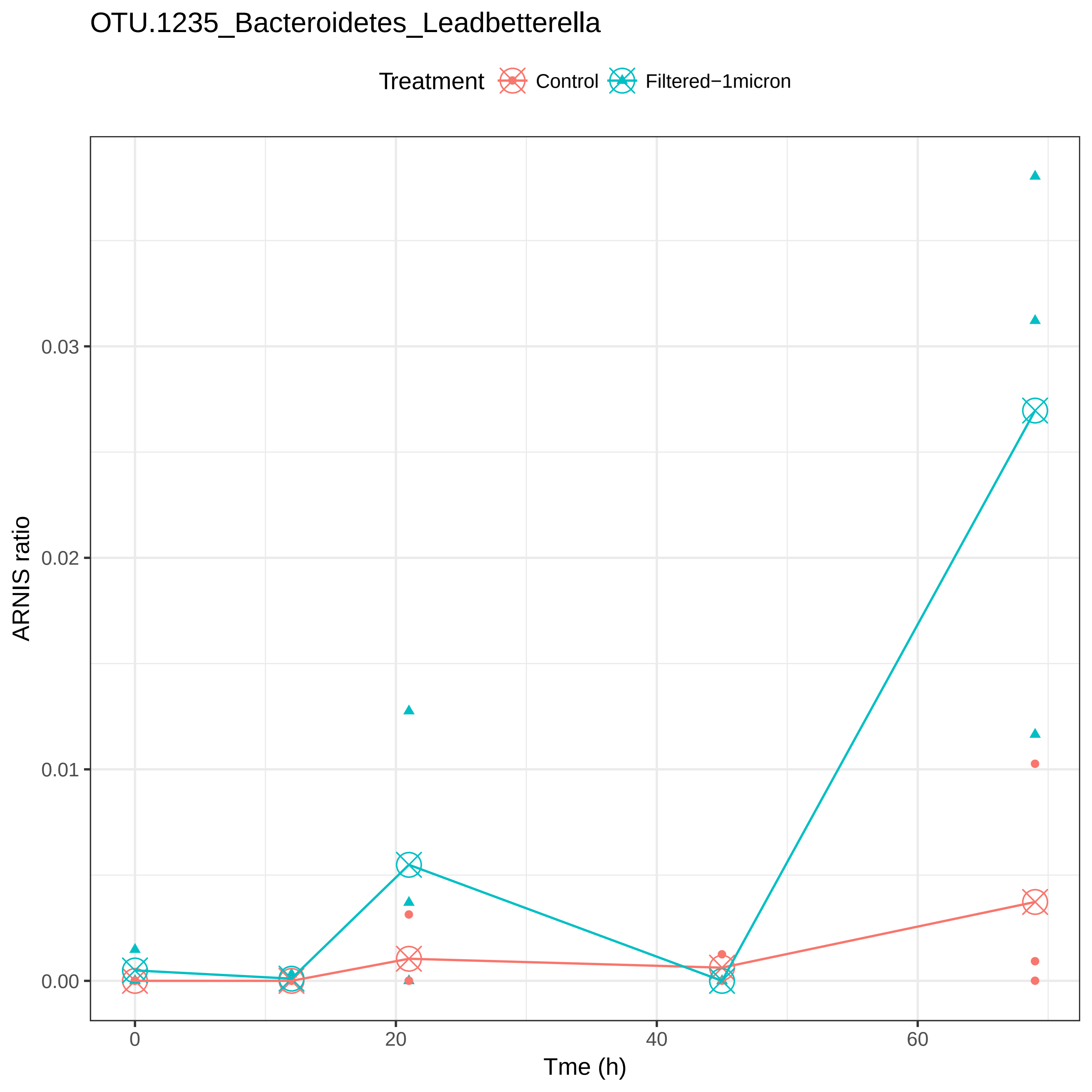

OTU.1235_Bacteroidetes_Leadbetterella
Treatment
Control
Filtered−1micron
ARNIS ratio
0.00
0.01
0.02
0.03
Tme (h)
0
20
40
60

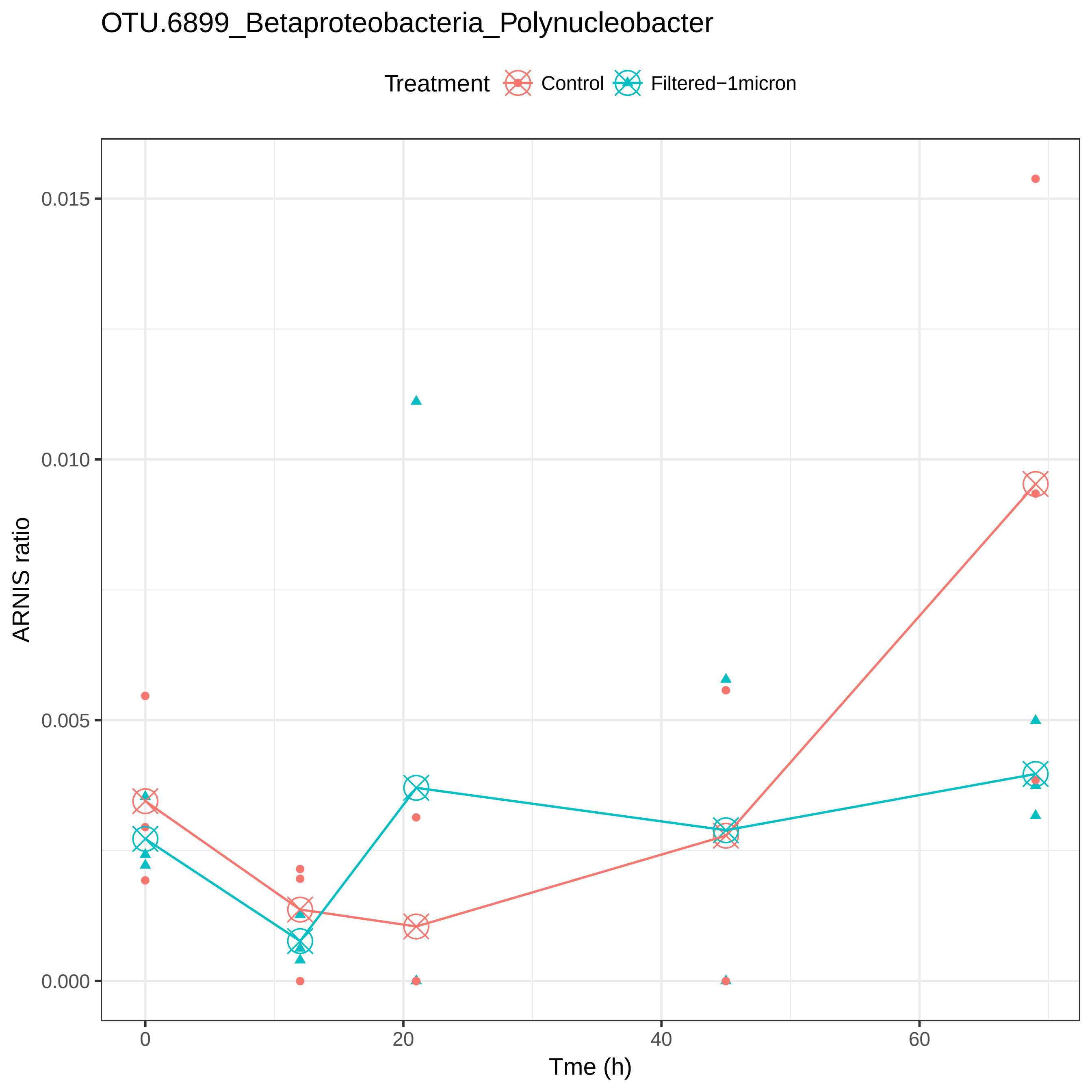

OTU.6899_Betaproteobacteria_Polynucleobacter
Treatment
Control
Filtered−1micron
ARNIS ratio
0.015
0.010
0.005
0.000
0
20
40
60
Tme (h)

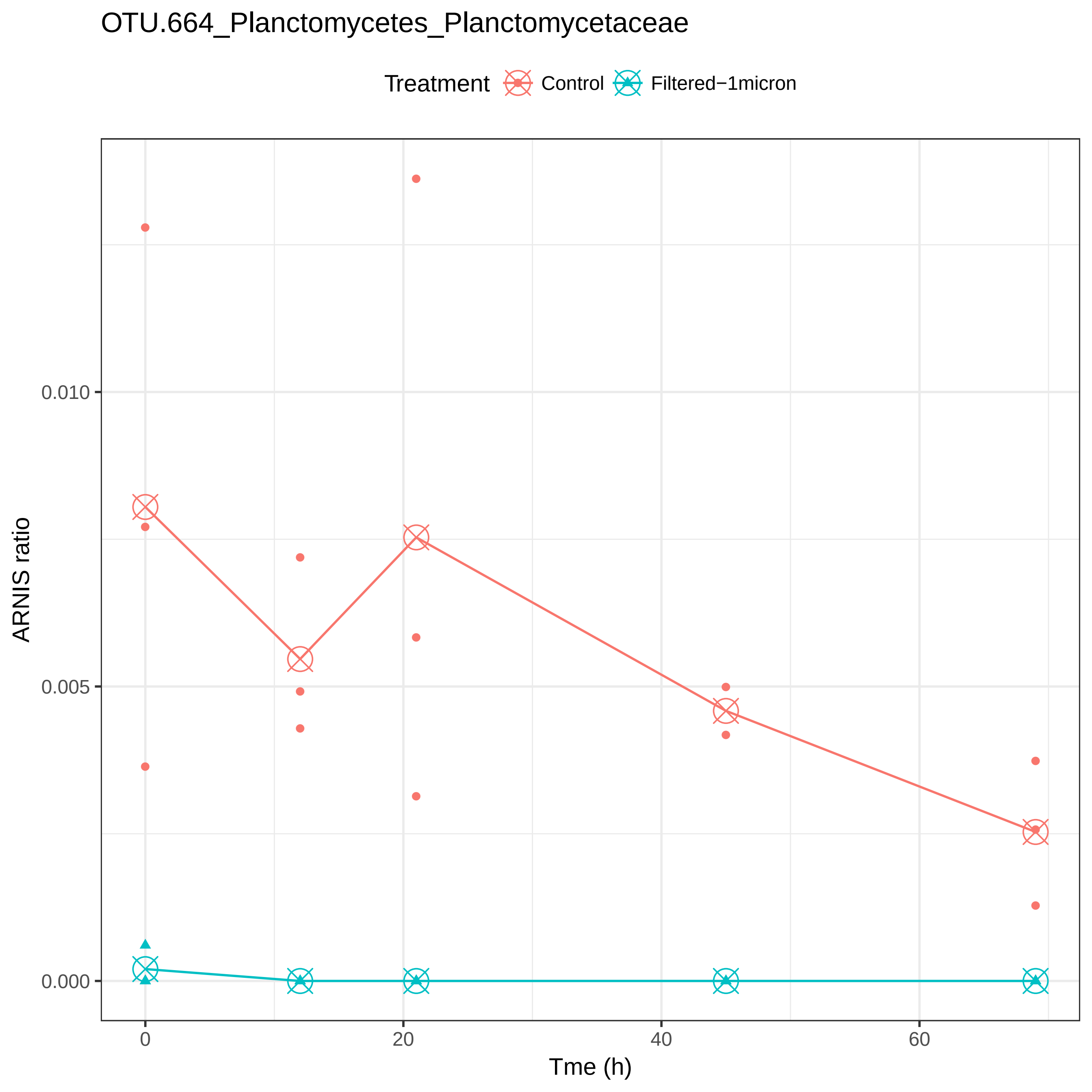

OTU.664_Planctomycetes_Planctomycetaceae
Treatment
Control
Filtered−1micron
ARNIS ratio
0.010
0.005
0.000
0
20
40
60
Tme (h)

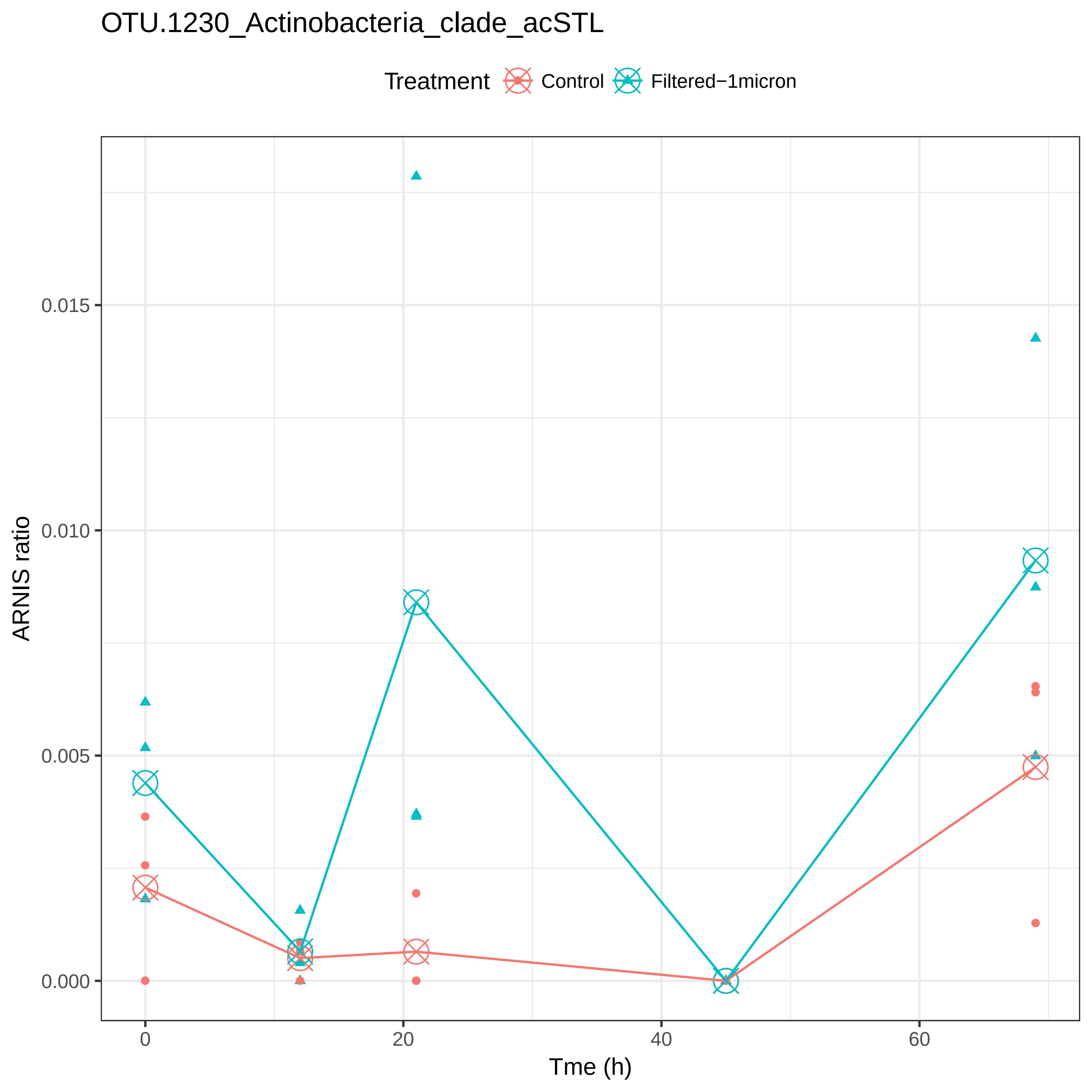

OTU.1230_Actinobacteria_clade_acSTL
Treatment
Control
Filtered−1micron
ARNIS ratio
0.000
0.005
0.010
0.015
0
20
40
60
Tme (h)

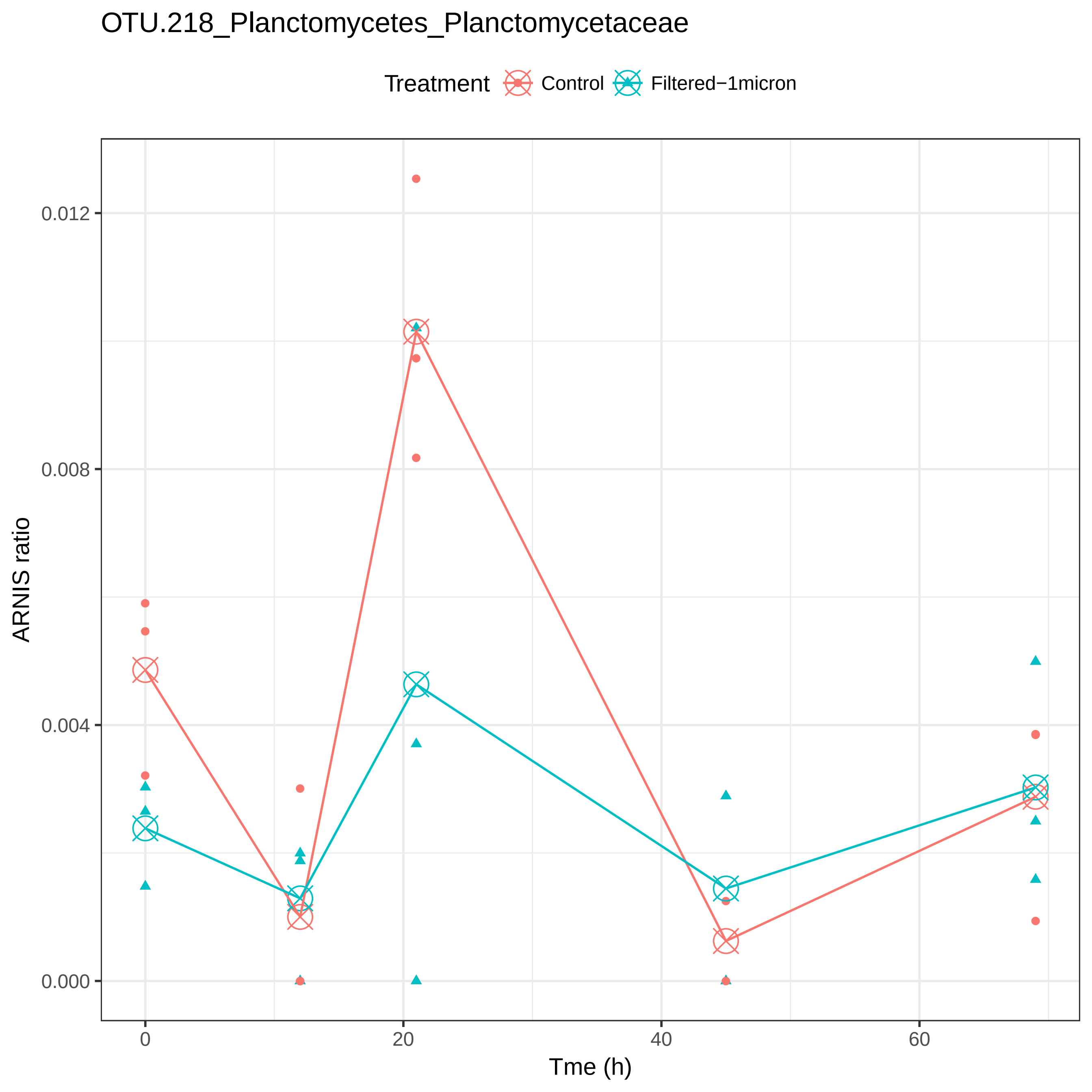

OTU.218_Planctomycetes_Planctomycetaceae
Treatment
Control
Filtered−1micron
0.012
0.008
0.004
0.000
ARNIS ratio
0
20
40
60
Tme (h)

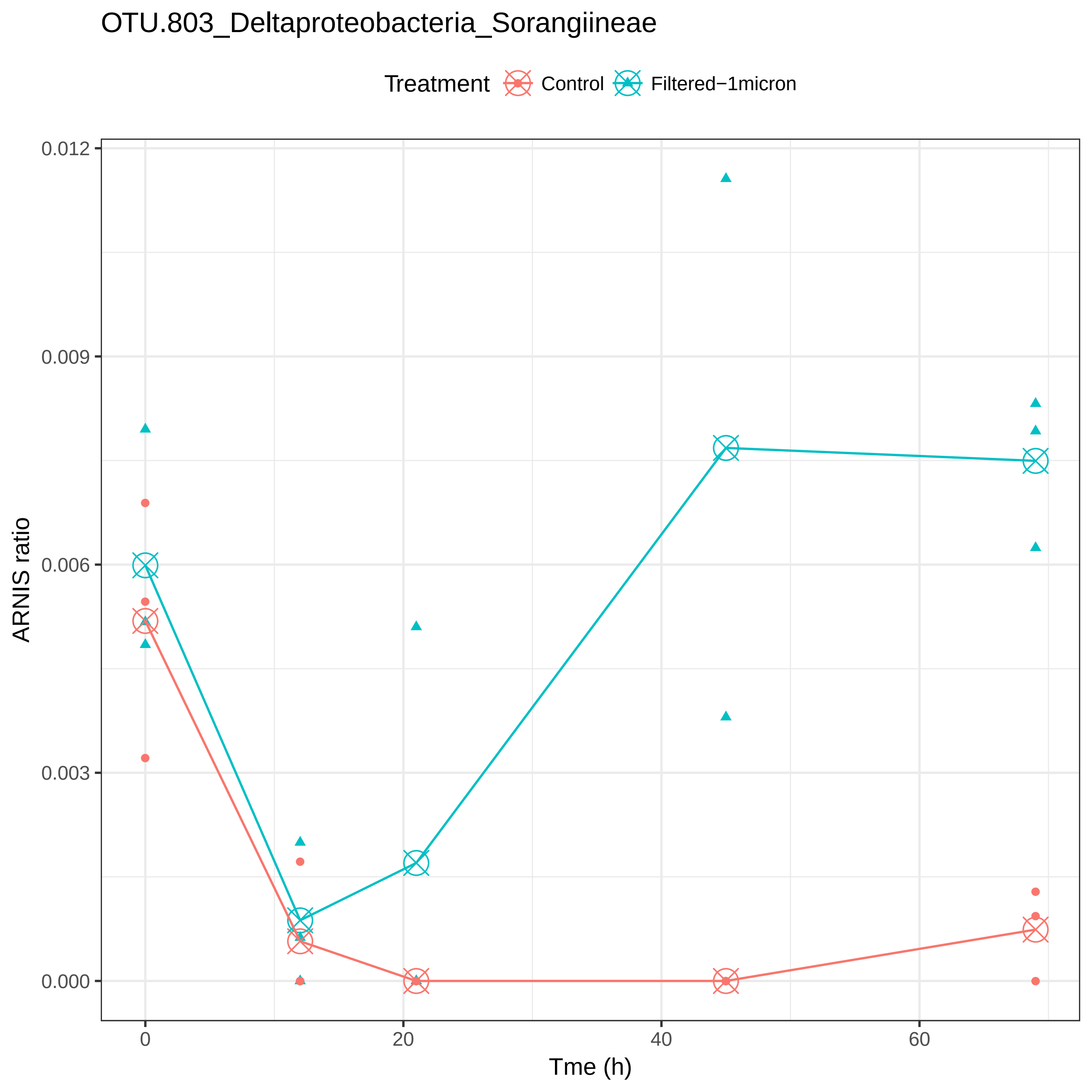

OTU.803_Deltaproteobacteria_Sorangiineae
Treatment
Control
Filtered−1micron
ARNIS ratio
0.012
0.009
0.006
0.003
0.000
0
20
40
60
Tme (h)

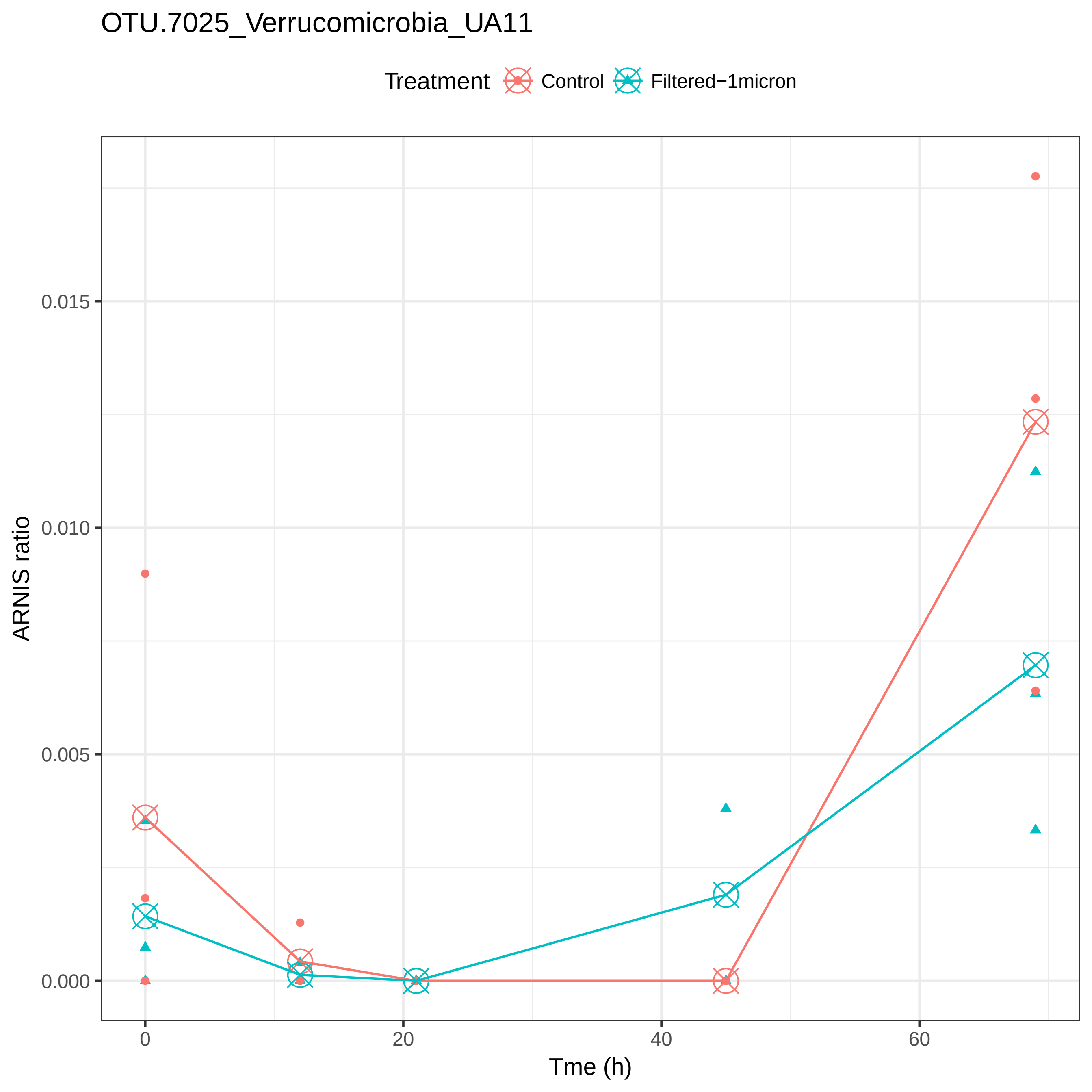
OTU.7025_Verrucomicrobia_UA11
Treatment
Control
Filtered−1micron
ARNIS ratio
0.000
0.005
0.010
0.015
0
20
40
60
Tme (h)

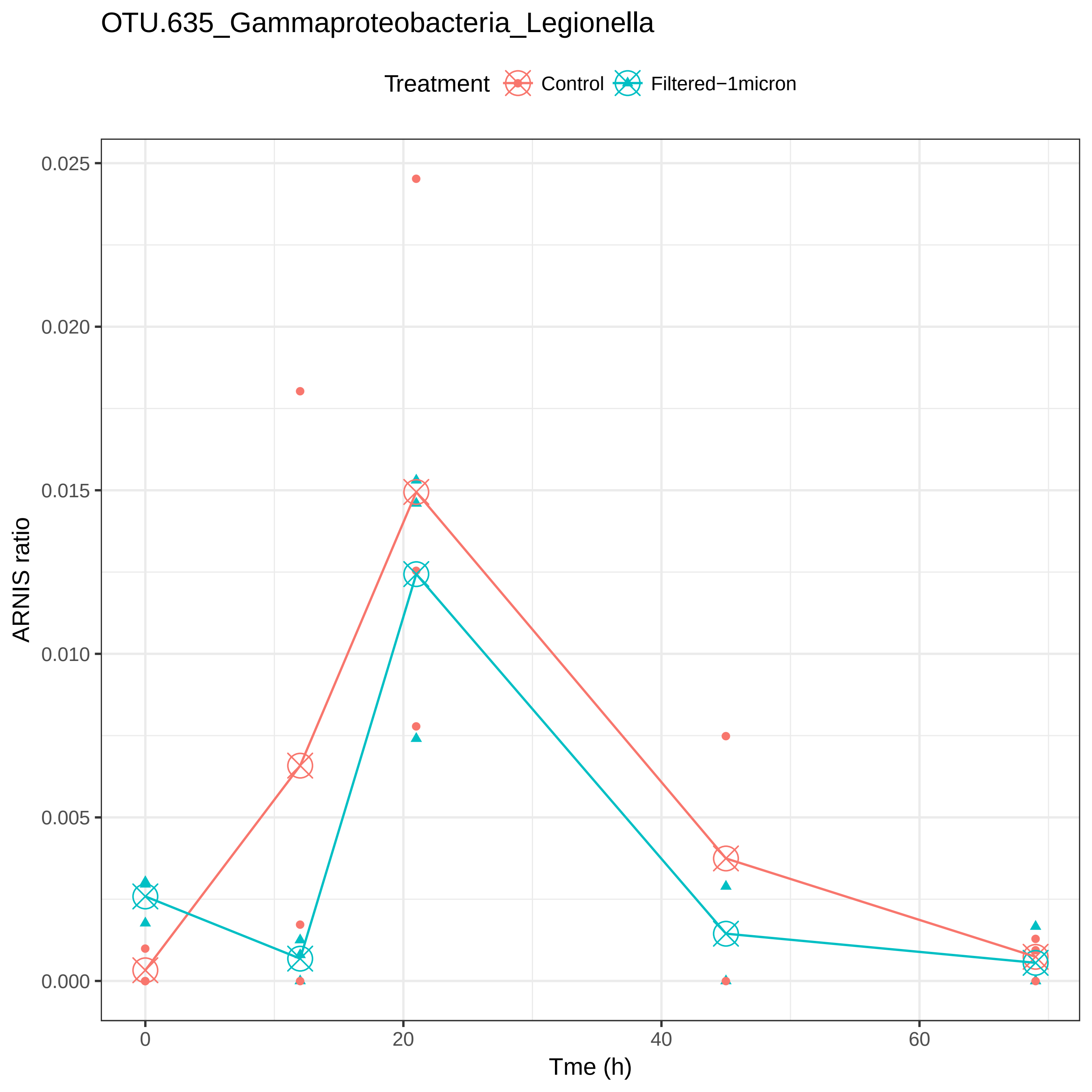

OTU.635_Gammaproteobacteria_Legionella
Treatment
Control
Filtered−1micron
ARNIS ratio
0.025
0.020
0.015
0.010
0.005
0.000
0
20
40
60
Tme (h)

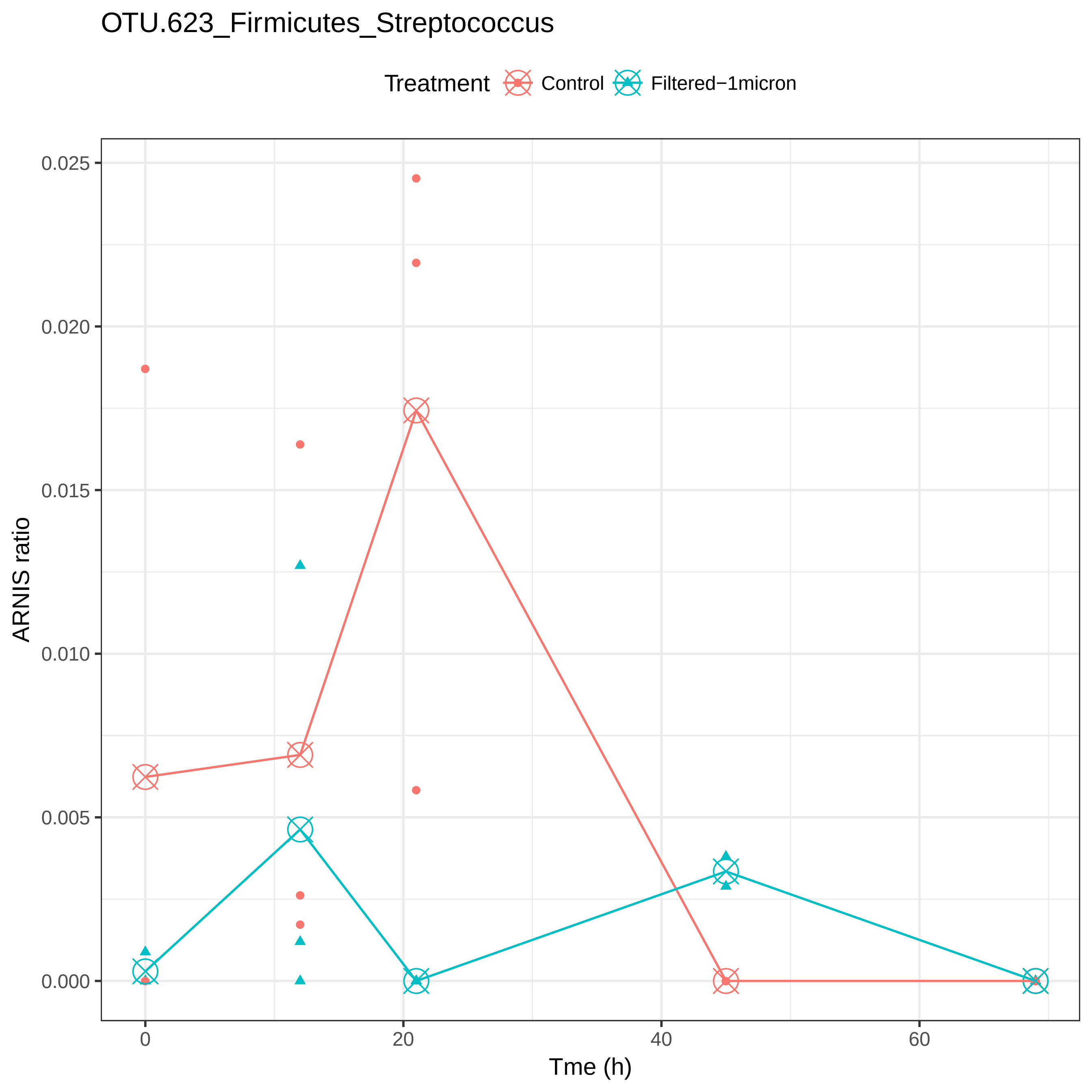

OTU.623_Firmicutes_Streptococcus
Treatment
Control
Filtered−1micron
ARNIS ratio
0.025
0.020
0.015
0.010
0.005
0.000
0
20
40
60
Tme (h)

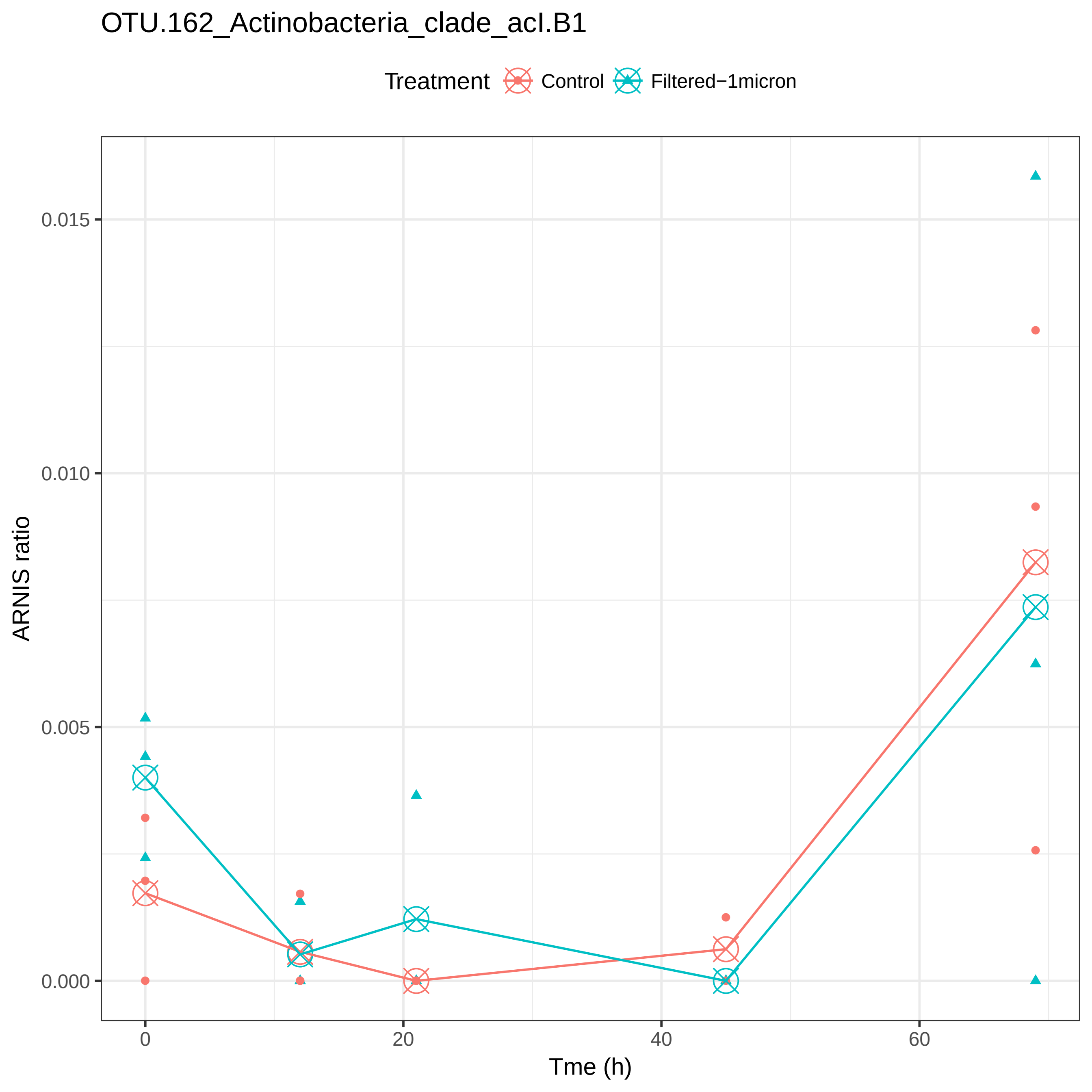

OTU.162_Actinobacteria_clade_acI.B1
Treatment
Control
Filtered−1micron
ARNIS ratio
0.015
0.010
0.005
0.000
0
20
40
60
Tme (h)

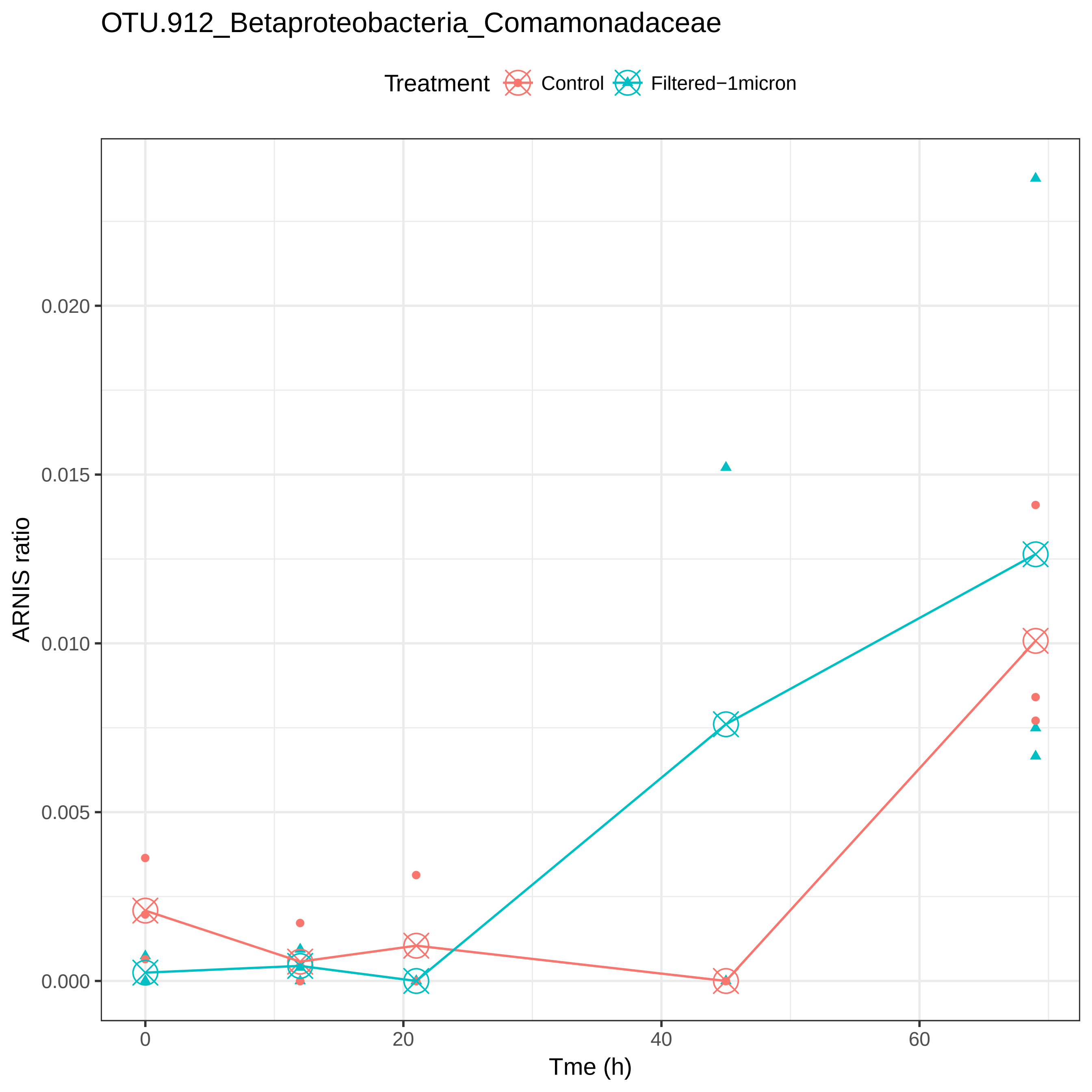

OTU.912_Betaproteobacteria_Comamonadaceae
Treatment
Control
Filtered−1micron
ARNIS ratio
0.020
0.015
0.010
0.005
0.000
0
20
40
60
Tme (h)

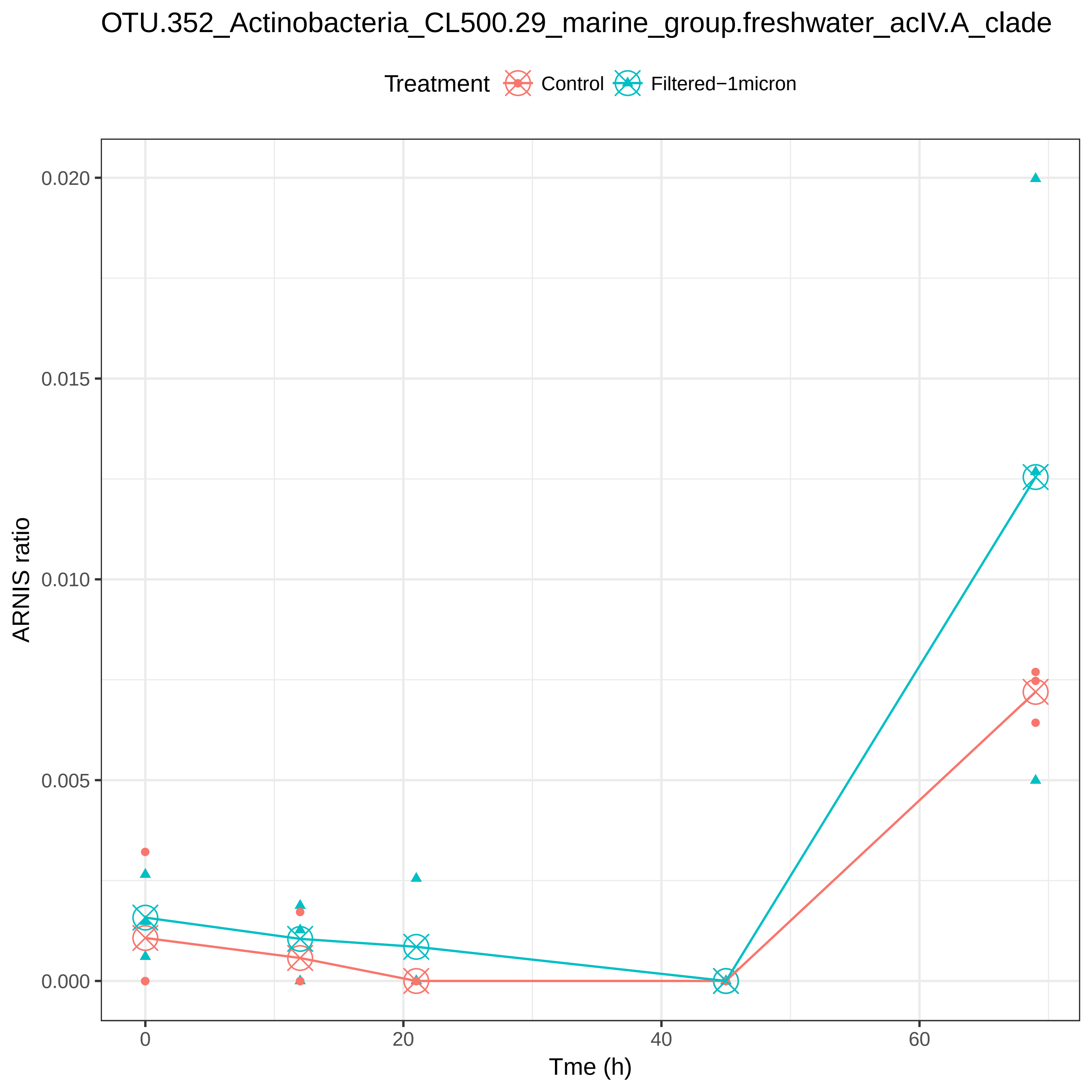

OTU.352_Actinobacteria_CL500.29_marine_group.freshwater_acIV.A_clade
Treatment
Control
Filtered−1micron
ARNIS ratio
0.000
0.005
0.010
0.015
0.020
0
20
40
60
Tme (h)

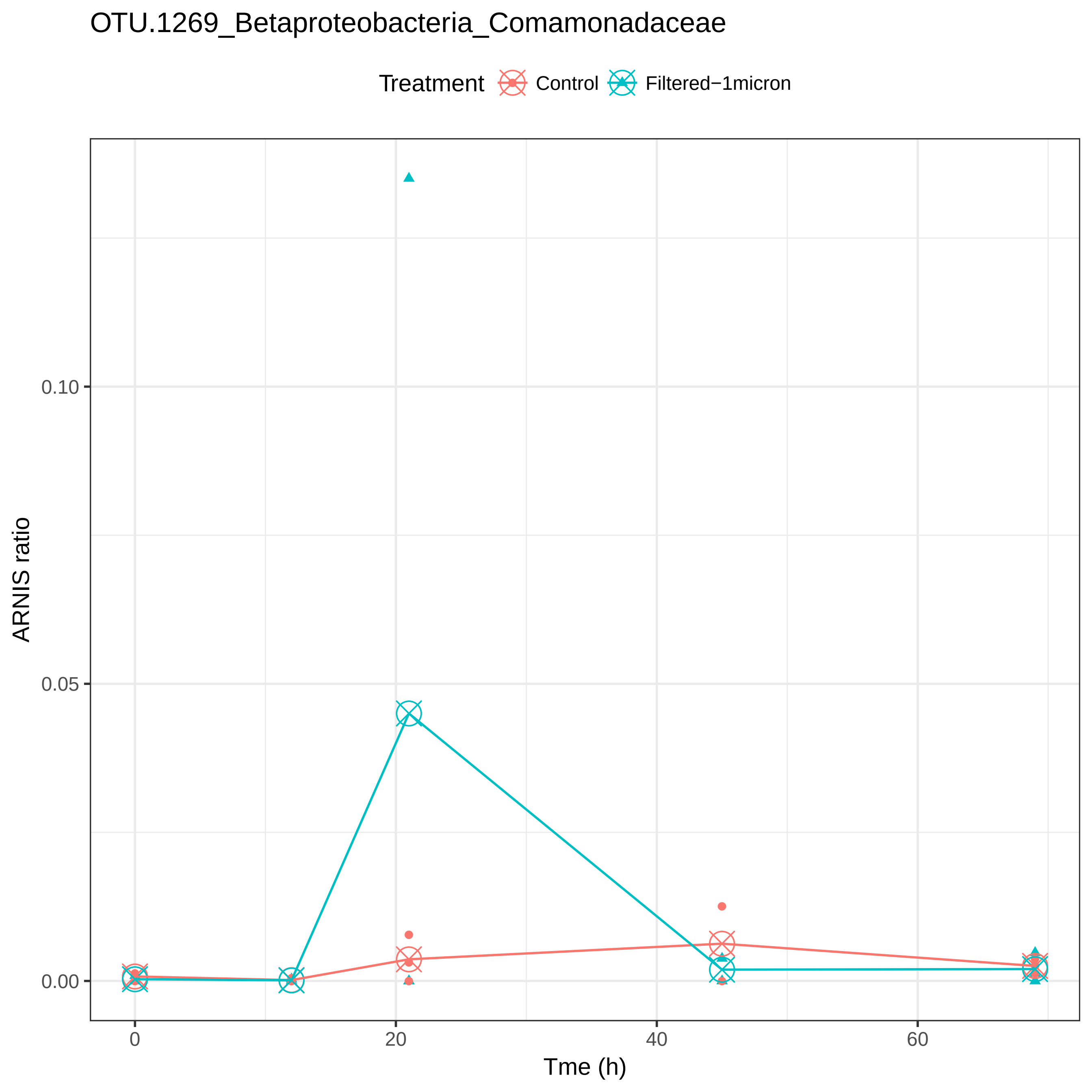
OTU.1269_Betaproteobacteria_Comamonadaceae
Treatment
Control
Filtered−1micron
ARNIS ratio
0.10
0.05
0.00
0
20
40
60
Tme (h)

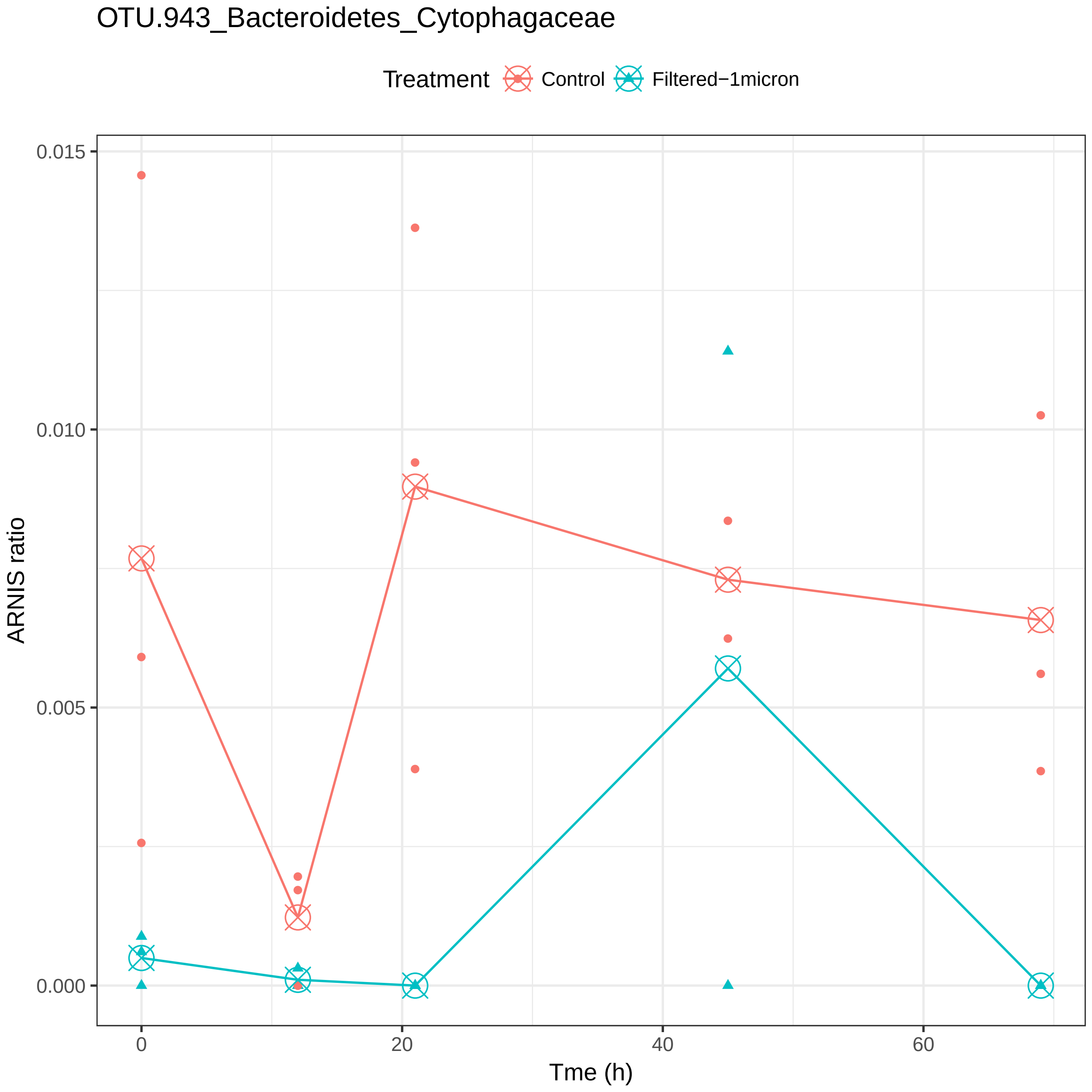
OTU.943_Bacteroidetes_Cytophagaceae
Treatment
Control
Filtered−1micron
ARNIS ratio
0.015
0.010
0.005
0.000
0
20
40
60
Tme (h)

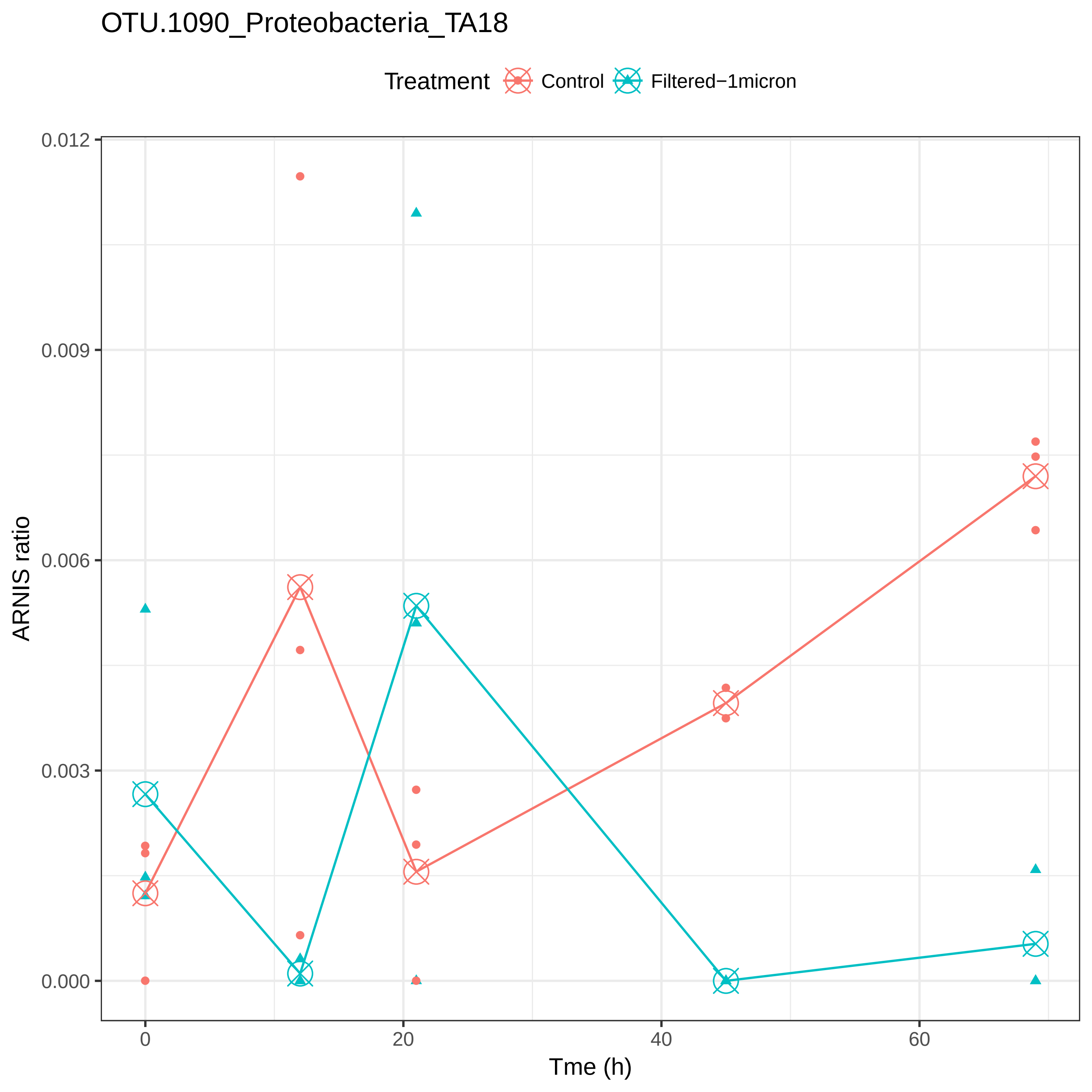

OTU.1090_Proteobacteria_TA18
Treatment
Control
Filtered−1micron
ARNIS ratio
0.012
0.009
0.006
0.003
0.000
0
20
40
60
Tme (h)

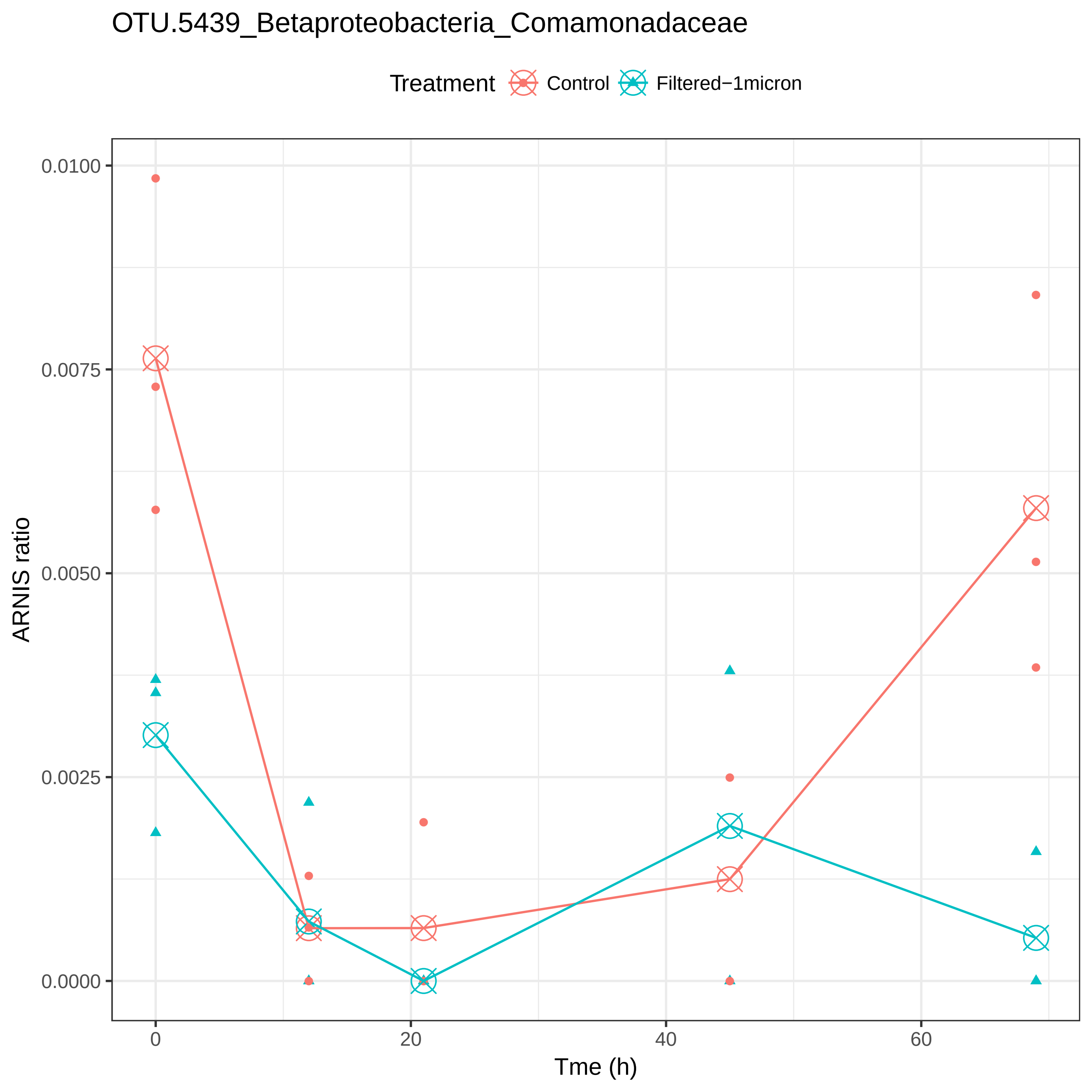
OTU.5439_Betaproteobacteria_Comamonadaceae
Treatment
Control
Filtered−1micron
ARNIS ratio
0.0100
0.0075
0.0050
0.0025
0.0000
0
20
40
60
Tme (h)

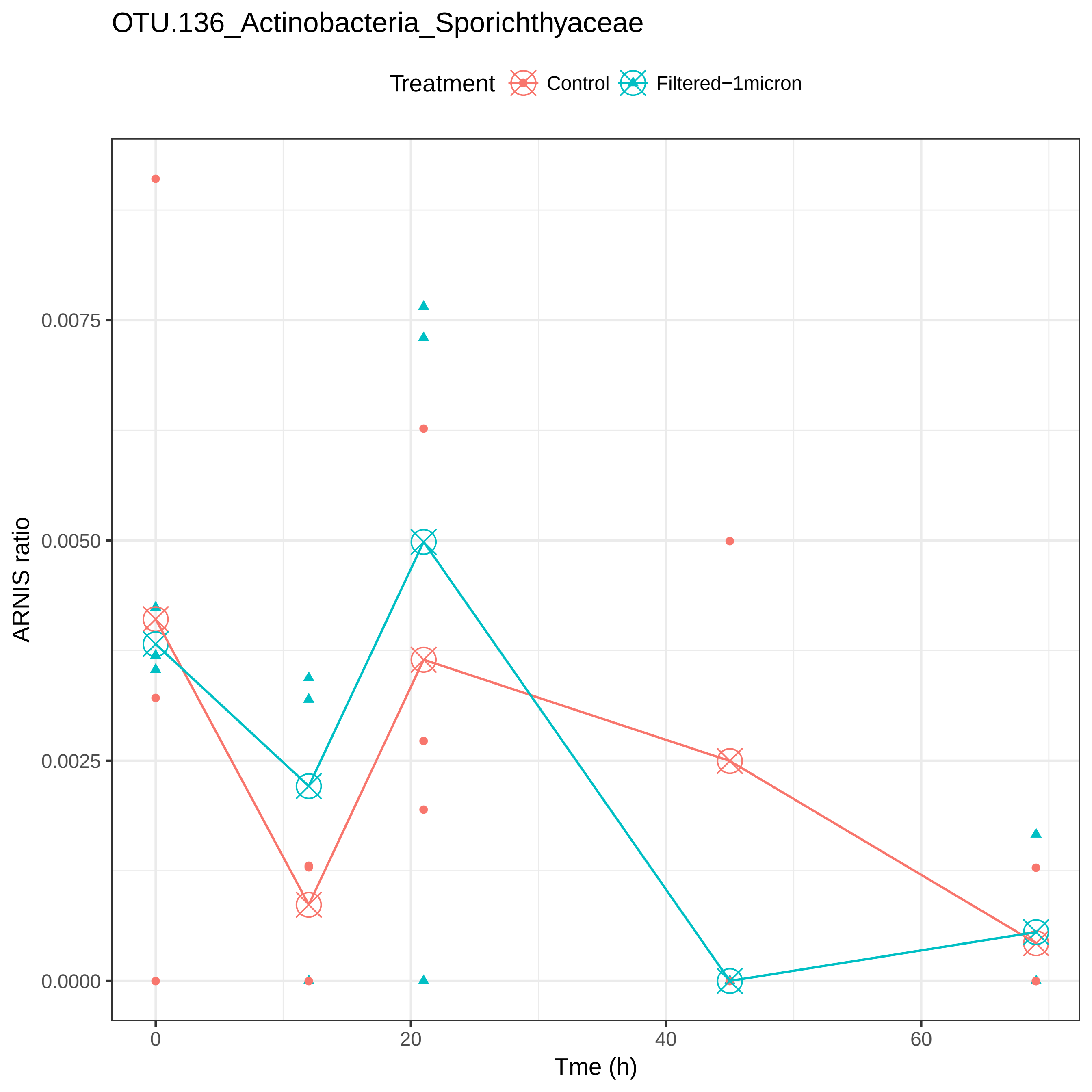
OTU.136_Actinobacteria_Sporichthyaceae
Treatment
Control
Filtered−1micron
ARNIS ratio
0.0000
0.0025
0.0050
0.0075
0
20
40
60
Tme (h)

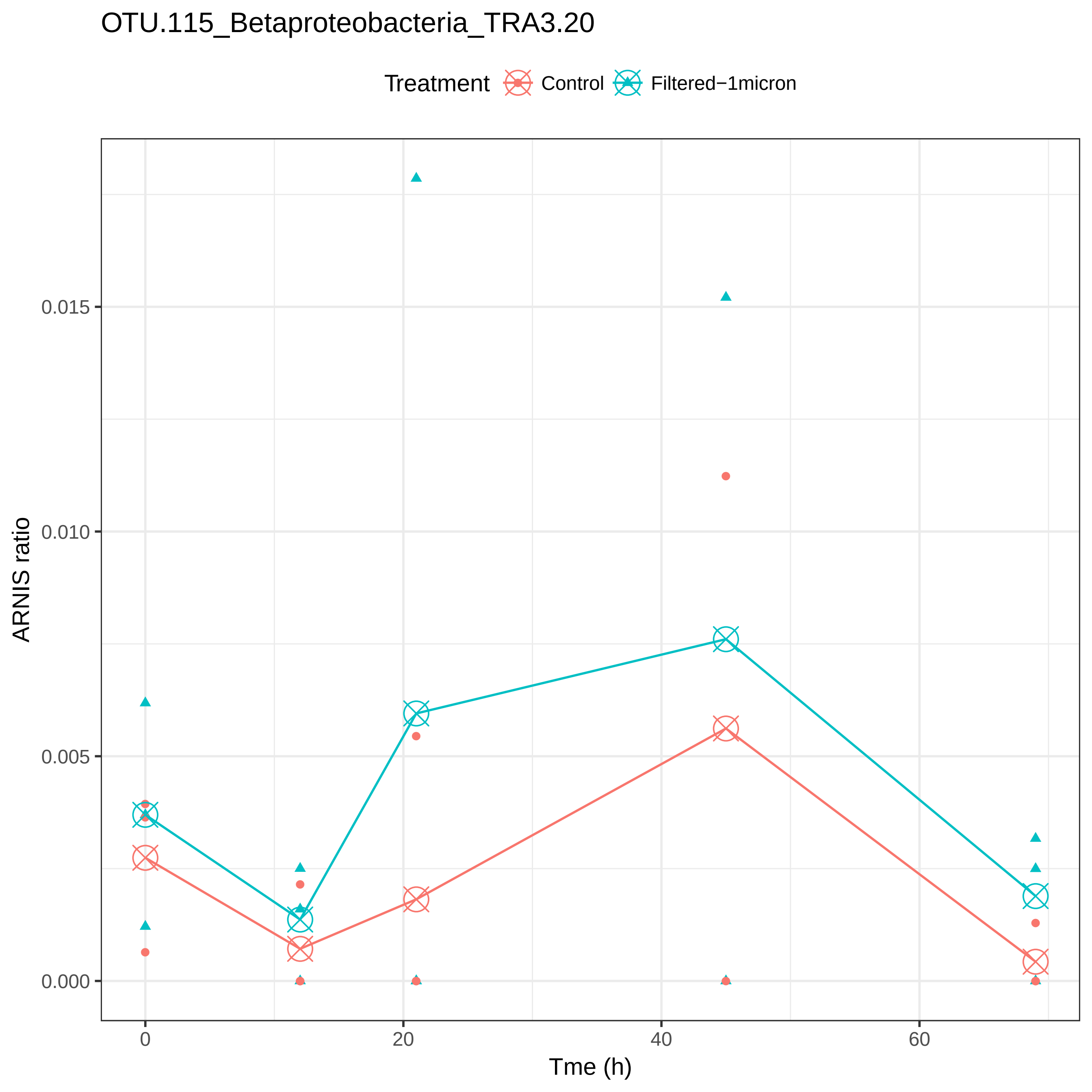

OTU.115_Betaproteobacteria_TRA3.20
Treatment
Control
Filtered−1micron
ARNIS ratio
0.015
0.010
0.005
0.000
0
20
40
60
Tme (h)

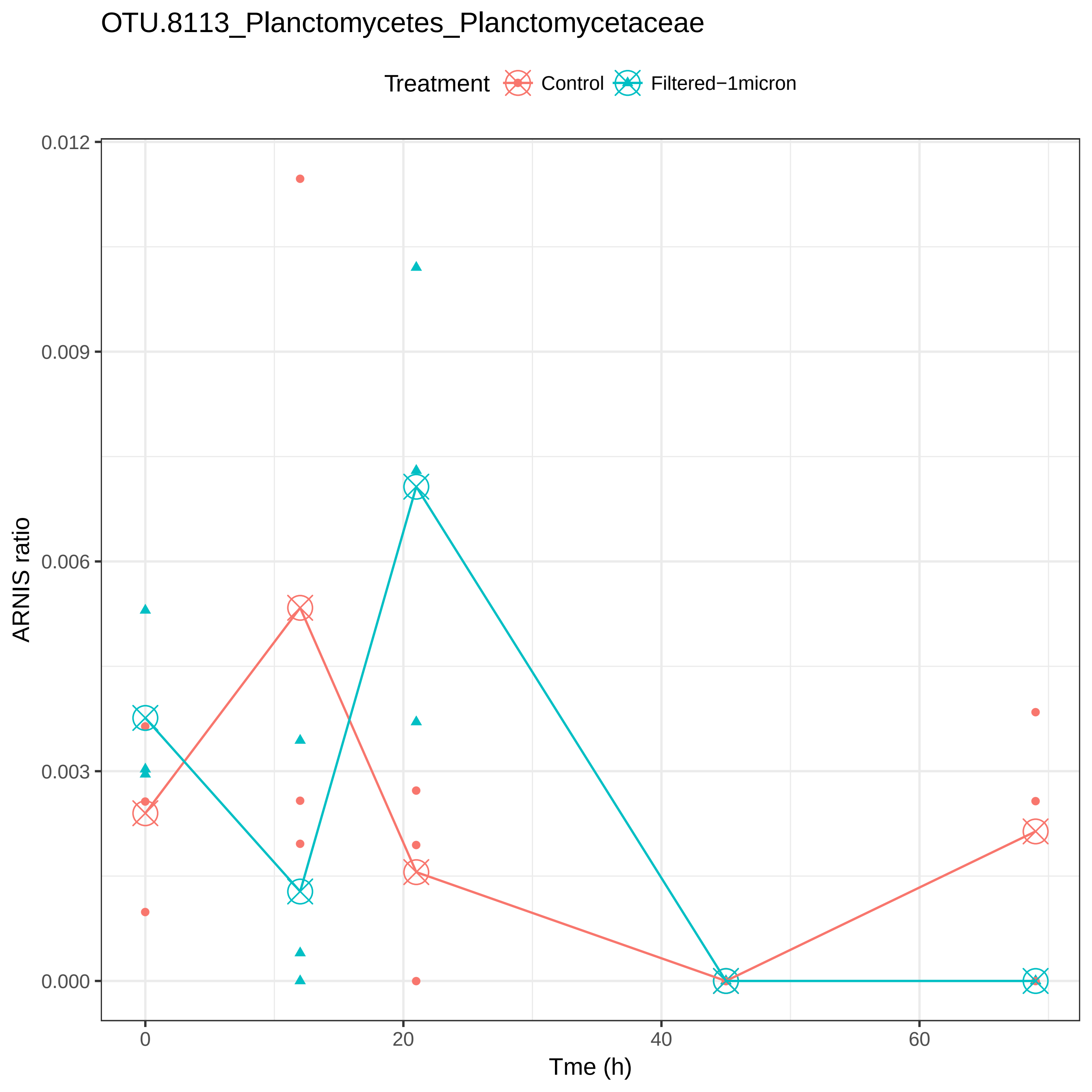

OTU.8113_Planctomycetes_Planctomycetaceae
Treatment
Control
Filtered−1micron
ARNIS ratio
0.012
0.009
0.006
0.003
0.000
0
20
40
60
Tme (h)

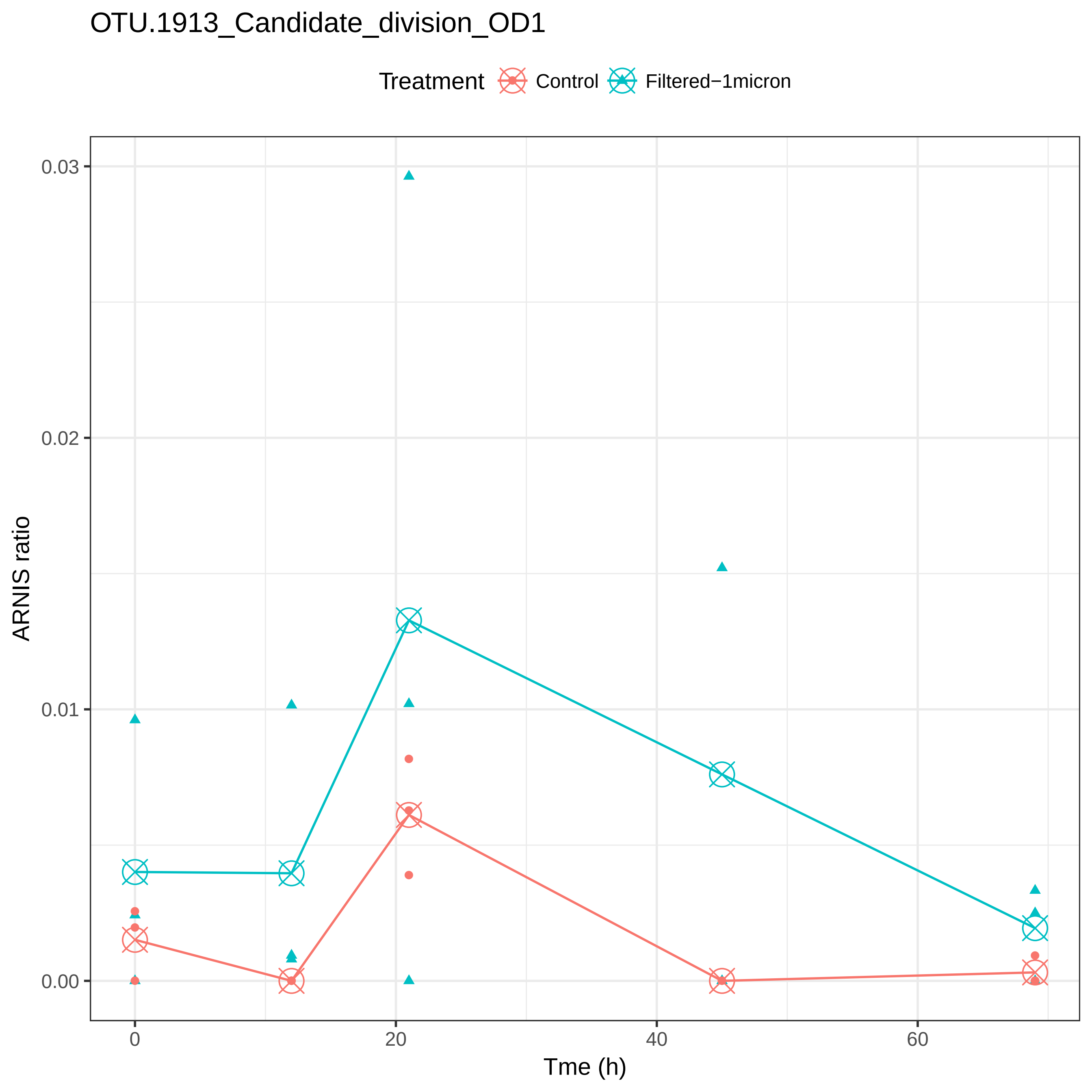
OTU.1913_Candidate_division_OD1
Treatment
Control
Filtered−1micron
ARNIS ratio
0.03
0.02
0.01
0.00
0
20
40
60
Tme (h)

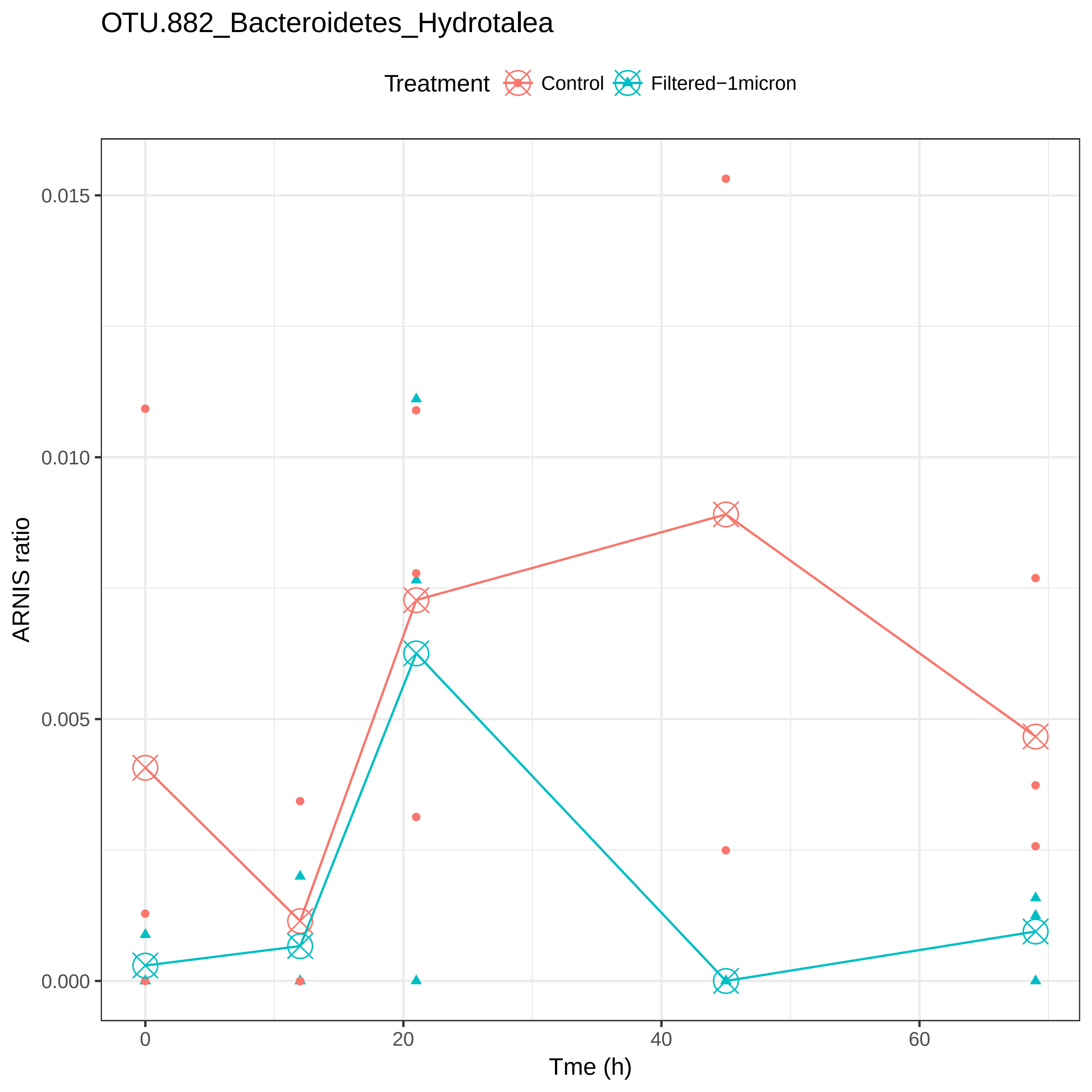

OTU.882_Bacteroidetes_Hydrotalea
Treatment
Control
Filtered−1micron
ARNIS ratio
0.015
0.010
0.005
0.000
0
20
40
60
Tme (h)

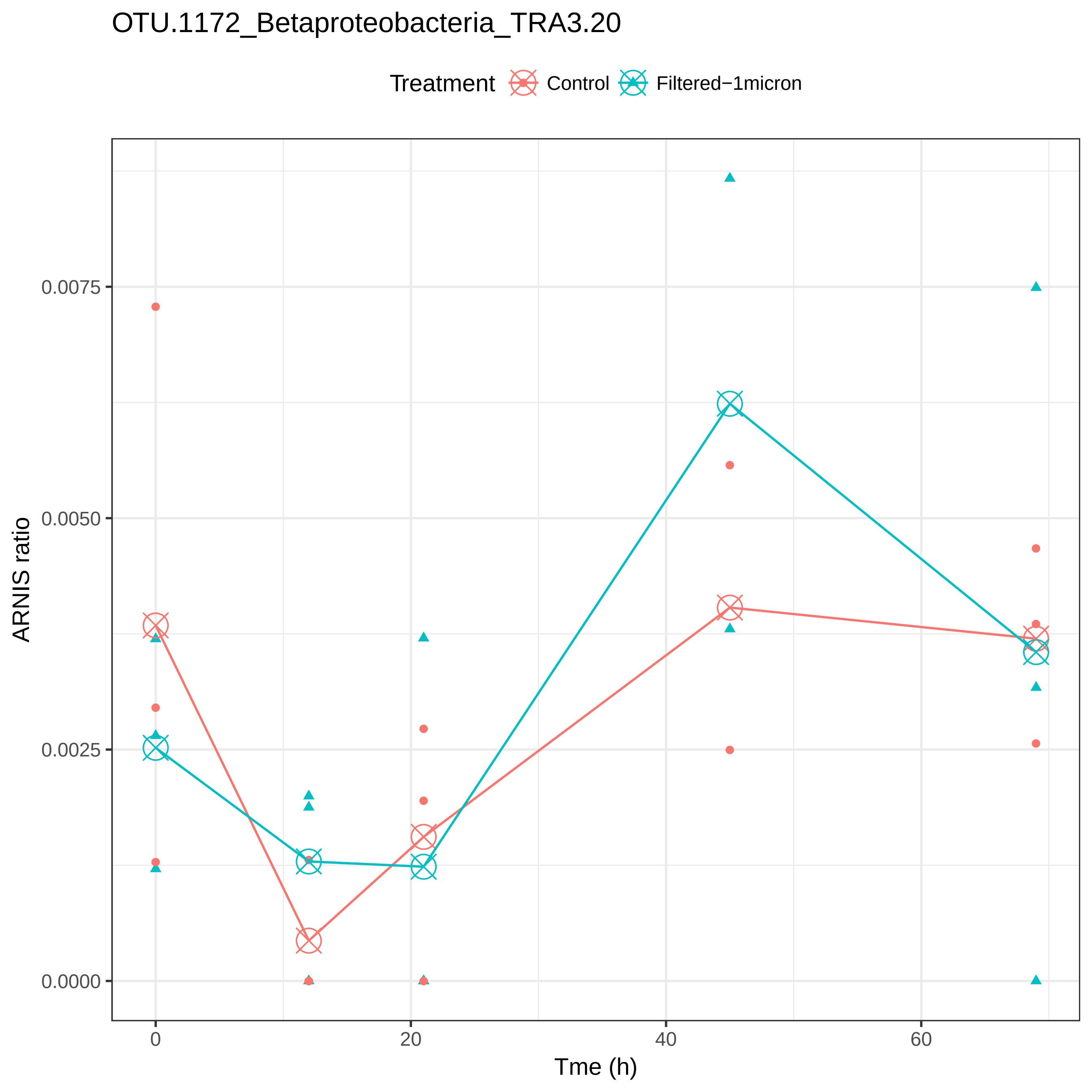

OTU.1172_Betaproteobacteria_TRA3.20
Treatment
Control
Filtered−1micron
ARNIS ratio
0.0075
0.0050
0.0025
0.0000
0
20
40
60
Tme (h)

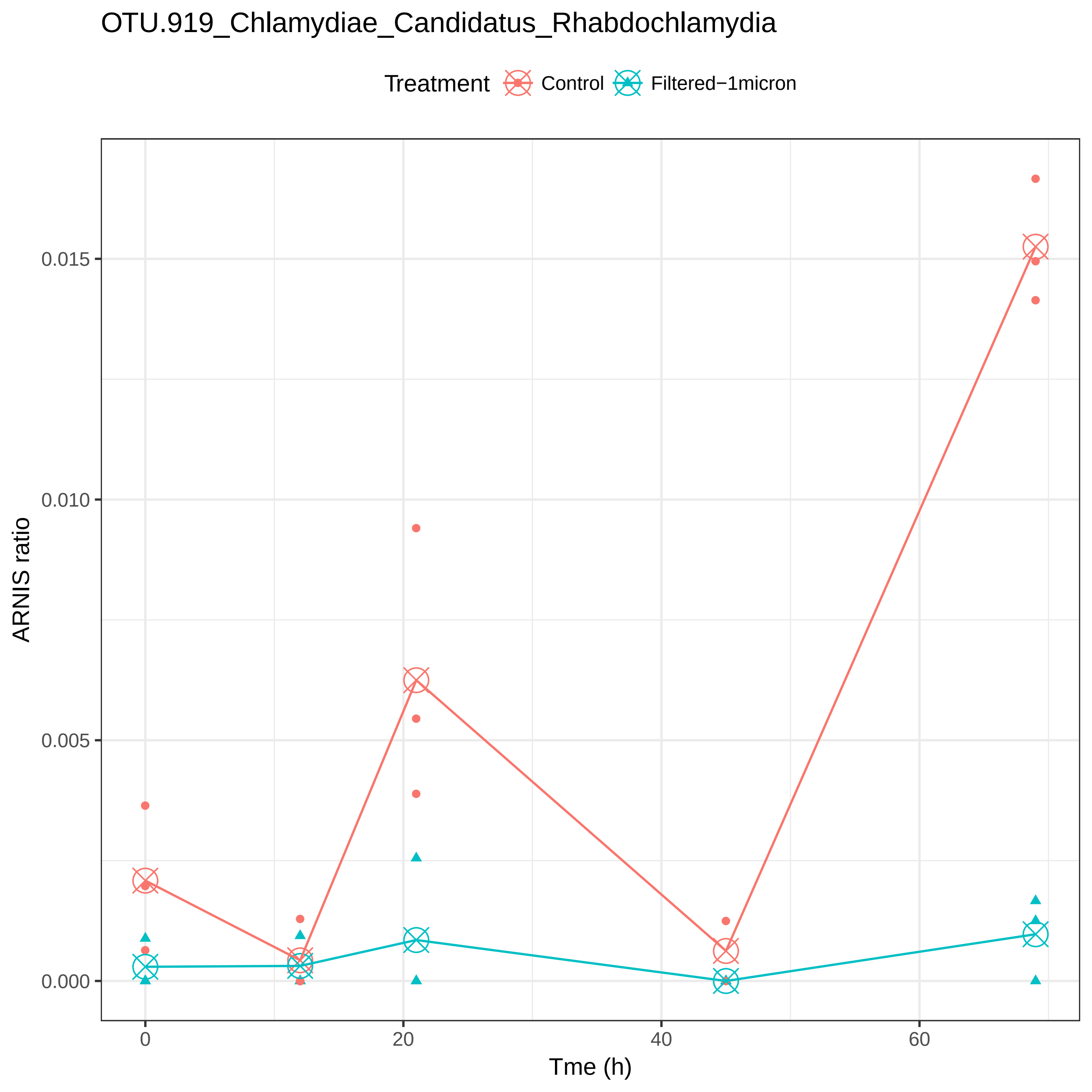
OTU.919_Chlamydiae_Candidatus_Rhabdochlamydia
Treatment
Control
Filtered−1micron
ARNIS ratio
0.015
0.010
0.005
0.000
0
20
40
60
Tme (h)

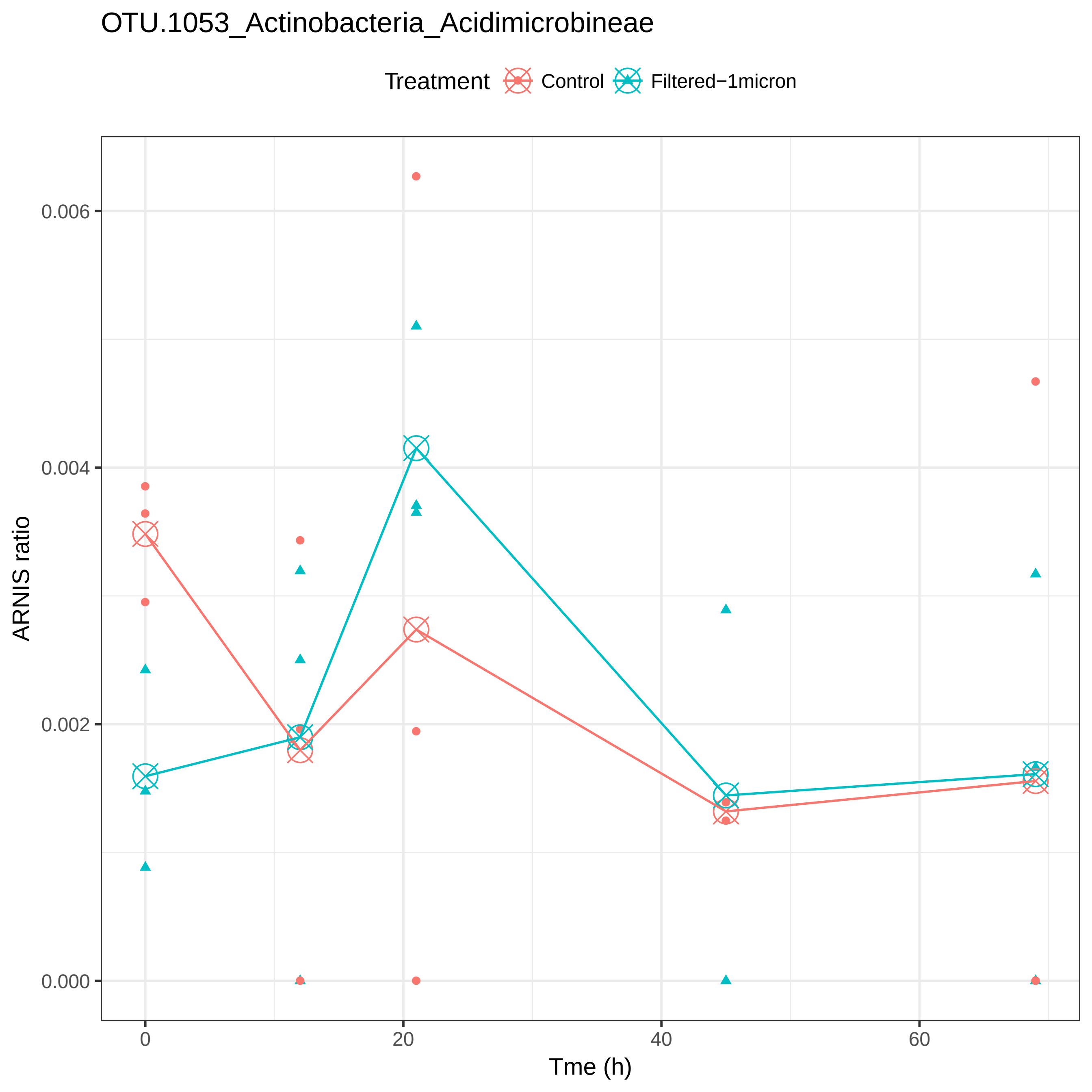
OTU.1053_Actinobacteria_Acidimicrobineae
Treatment
Control
Filtered−1micron
ARNIS ratio
0.006
0.004
0.002
0.000
0
20
40
60
Tme (h)

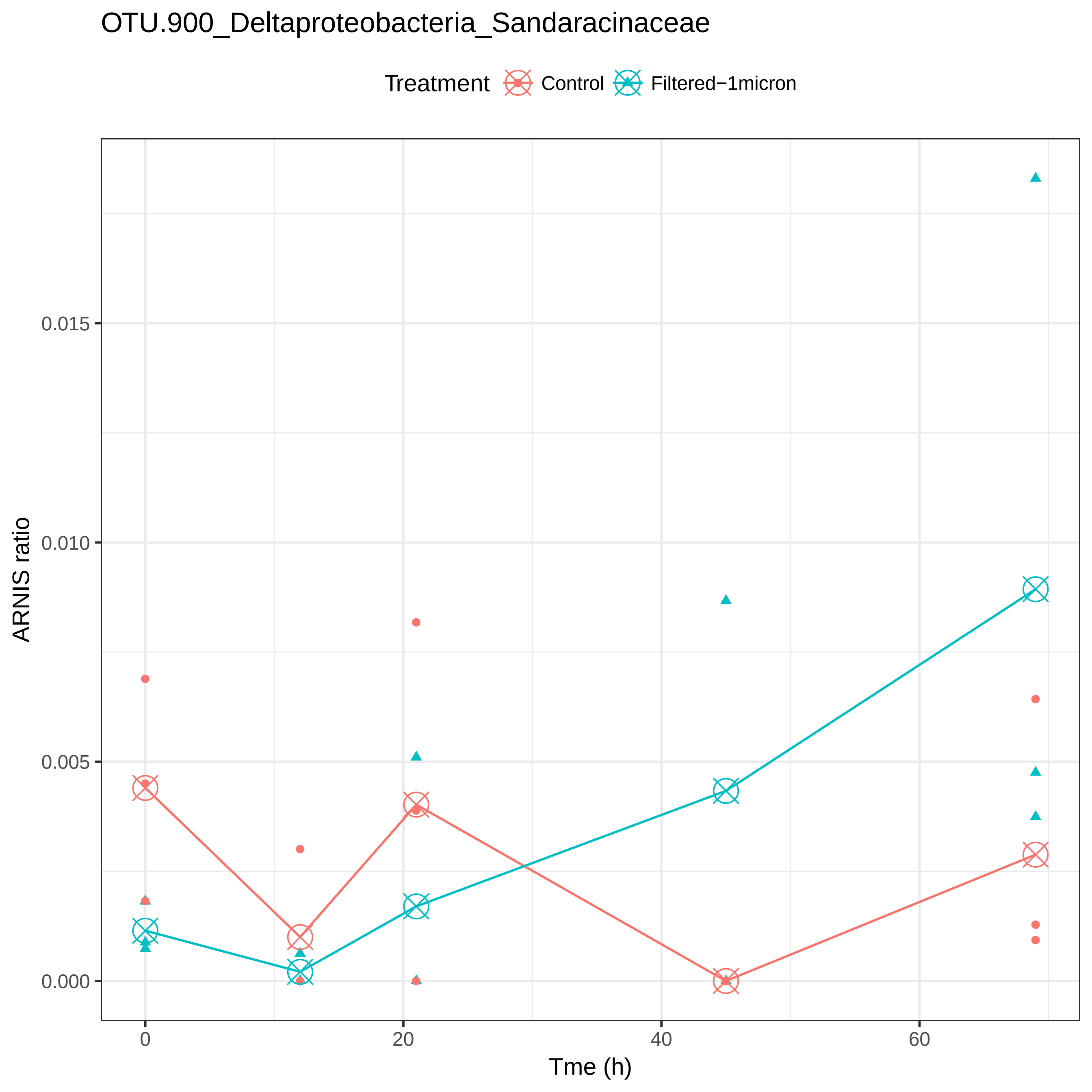

OTU.900_Deltaproteobacteria_Sandaracinaceae
Treatment
Control
Filtered−1micron
ARNIS ratio
0.015
0.010
0.005
0.000
0
20
40
60
Tme (h)

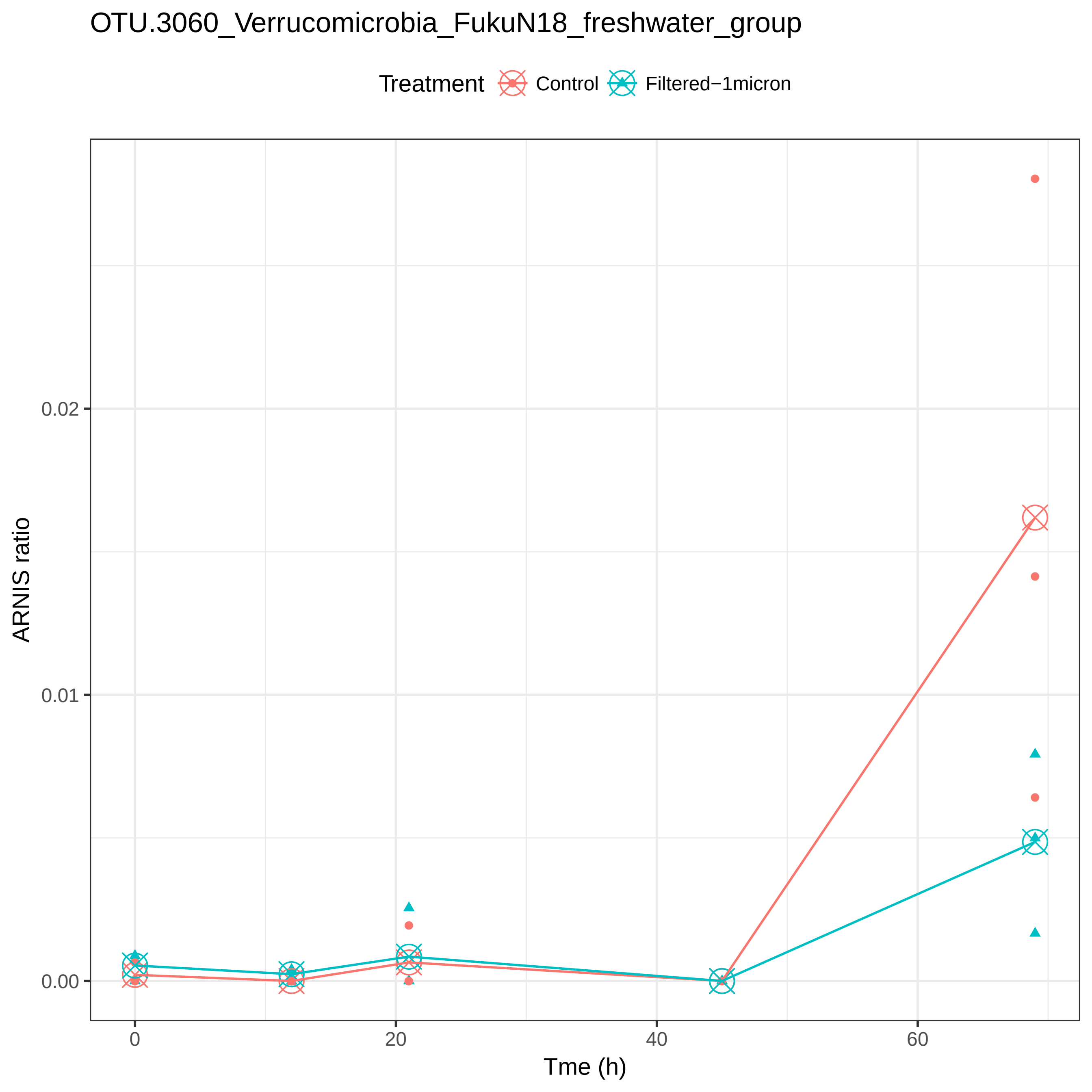

OTU.3060_Verrucomicrobia_FukuN18_freshwater_group
Treatment
Control
Filtered−1micron
ARNIS ratio
0.00
0.01
0.02
0
20
40
60
Tme (h)

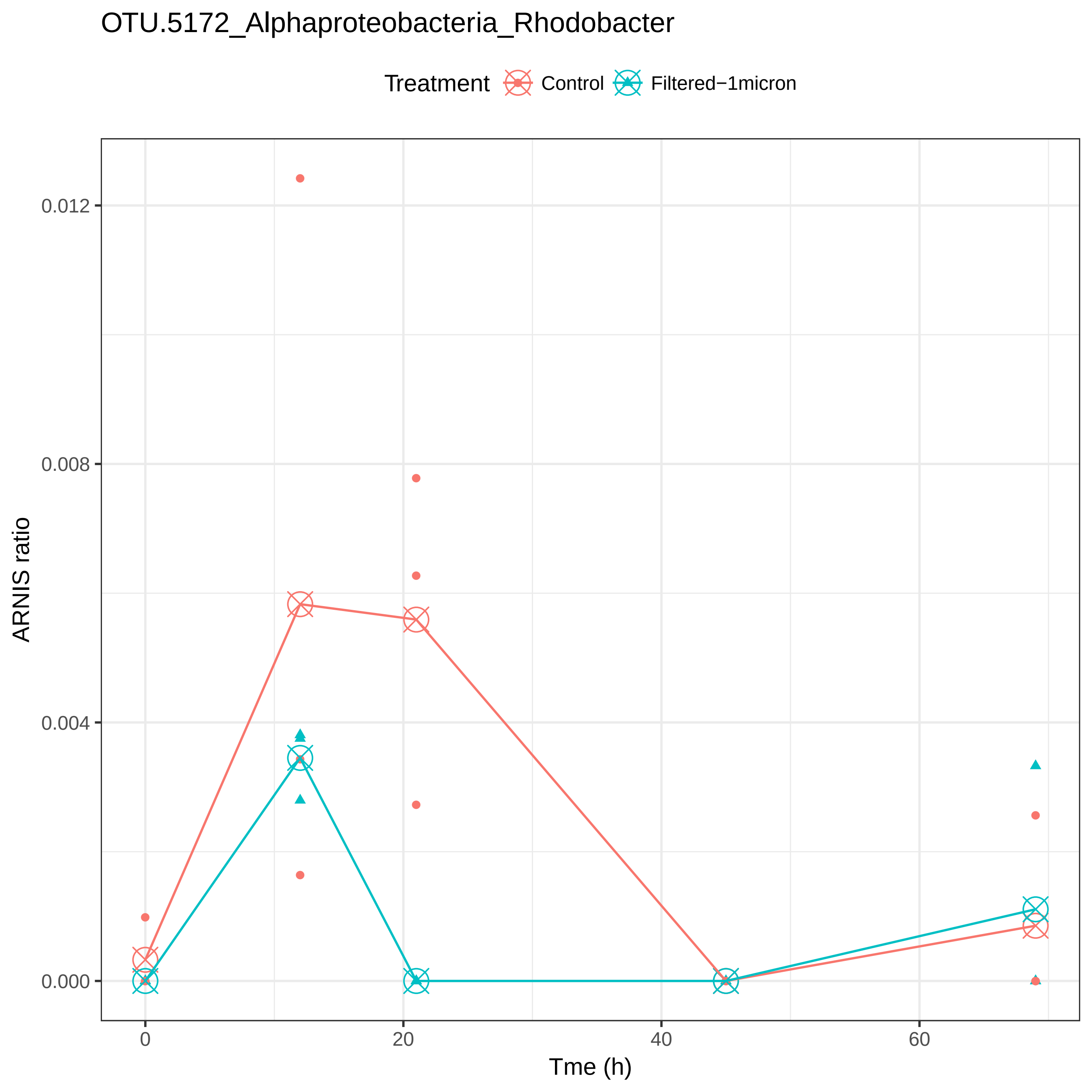
OTU.5172_Alphaproteobacteria_Rhodobacter
Treatment
Control
Filtered−1micron
ARNIS ratio
0.012
0.008
0.004
0.000
0
20
40
60
Tme (h)

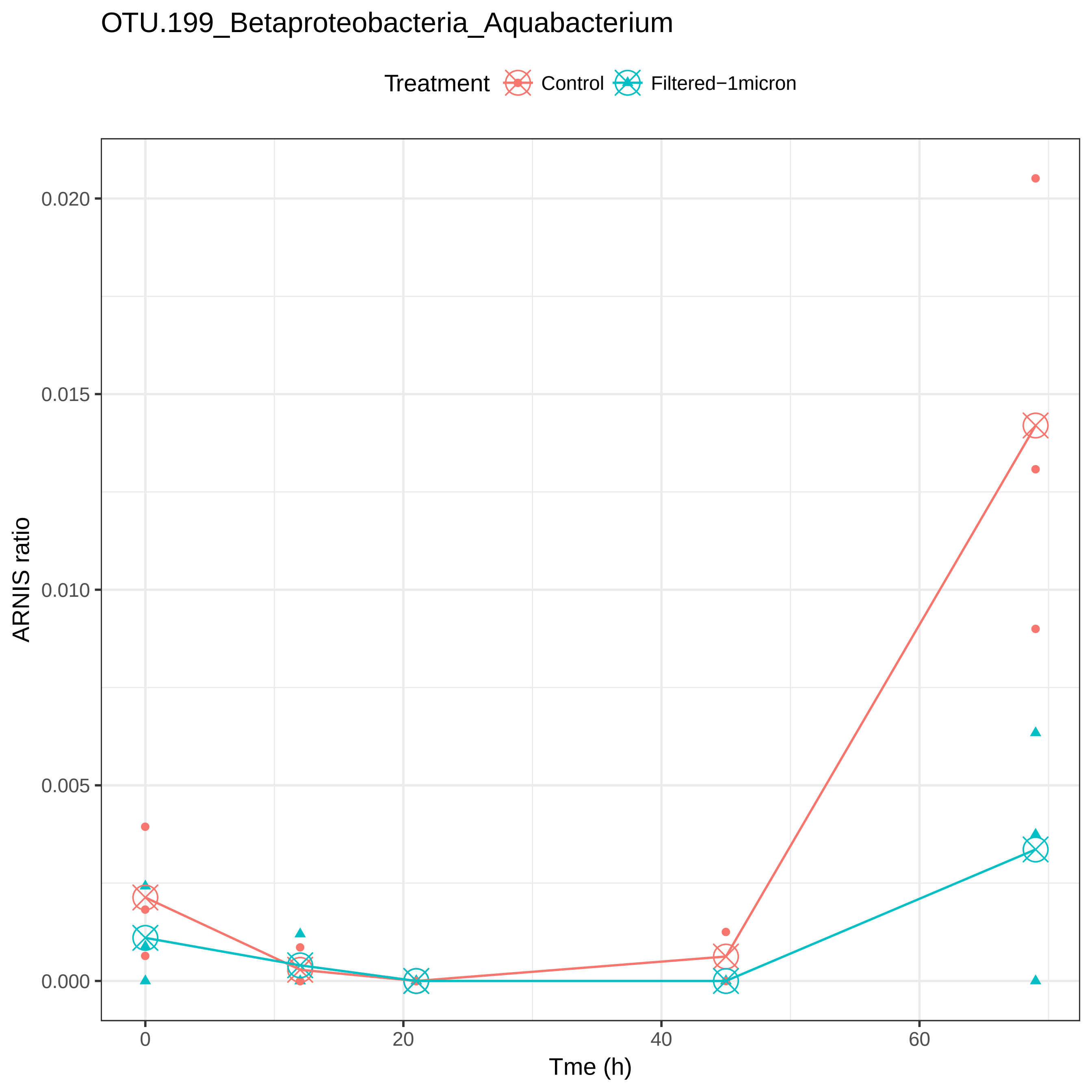

OTU.199_Betaproteobacteria_Aquabacterium
Treatment
Control
Filtered−1micron
ARNIS ratio
0.020
0.015
0.010
0.005
0.000
0
20
40
60
Tme (h)

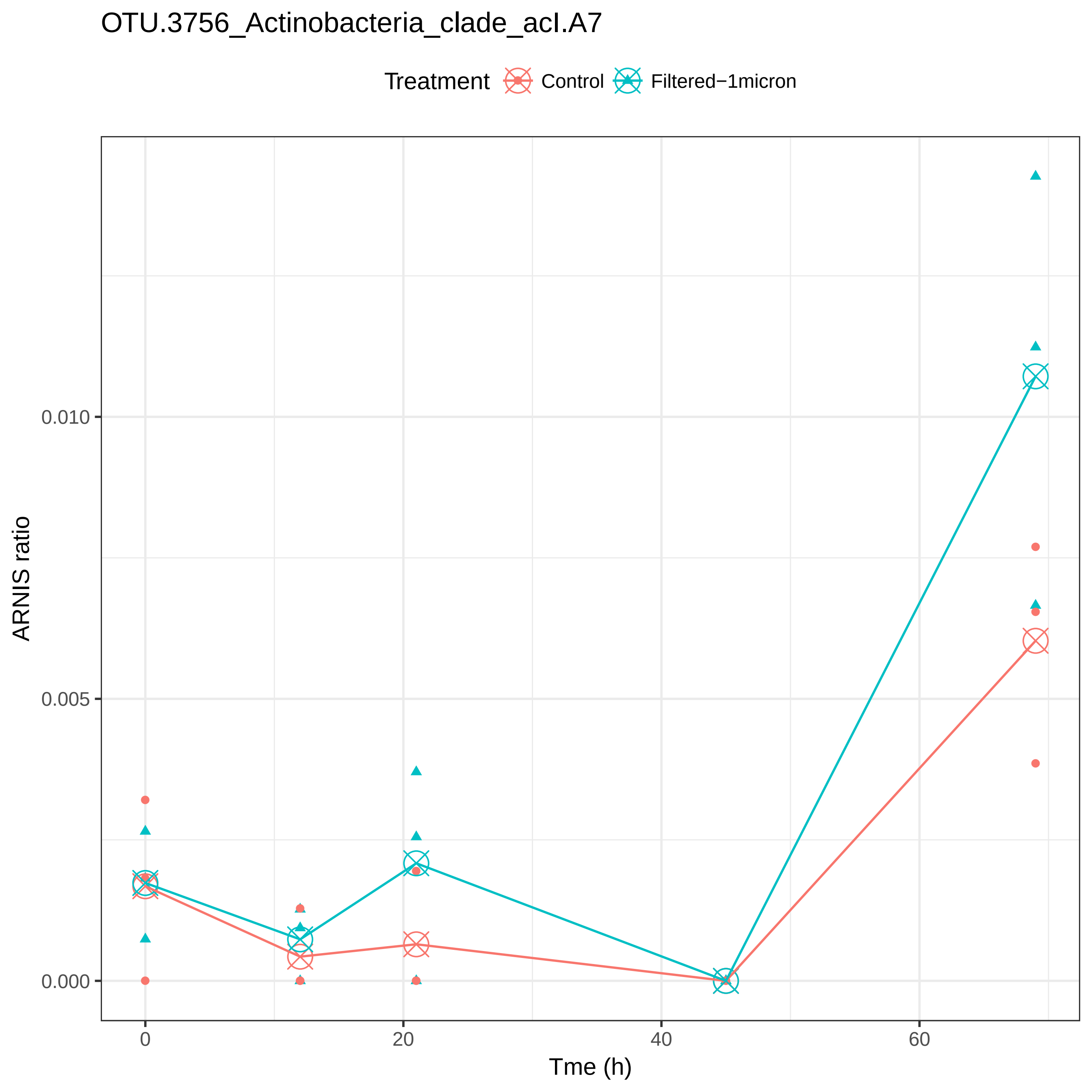

OTU.3756_Actinobacteria_clade_acI.A7
Treatment
Control
Filtered−1micron
ARNIS ratio
0.010
0.005
0.000
0
20
40
60
Tme (h)

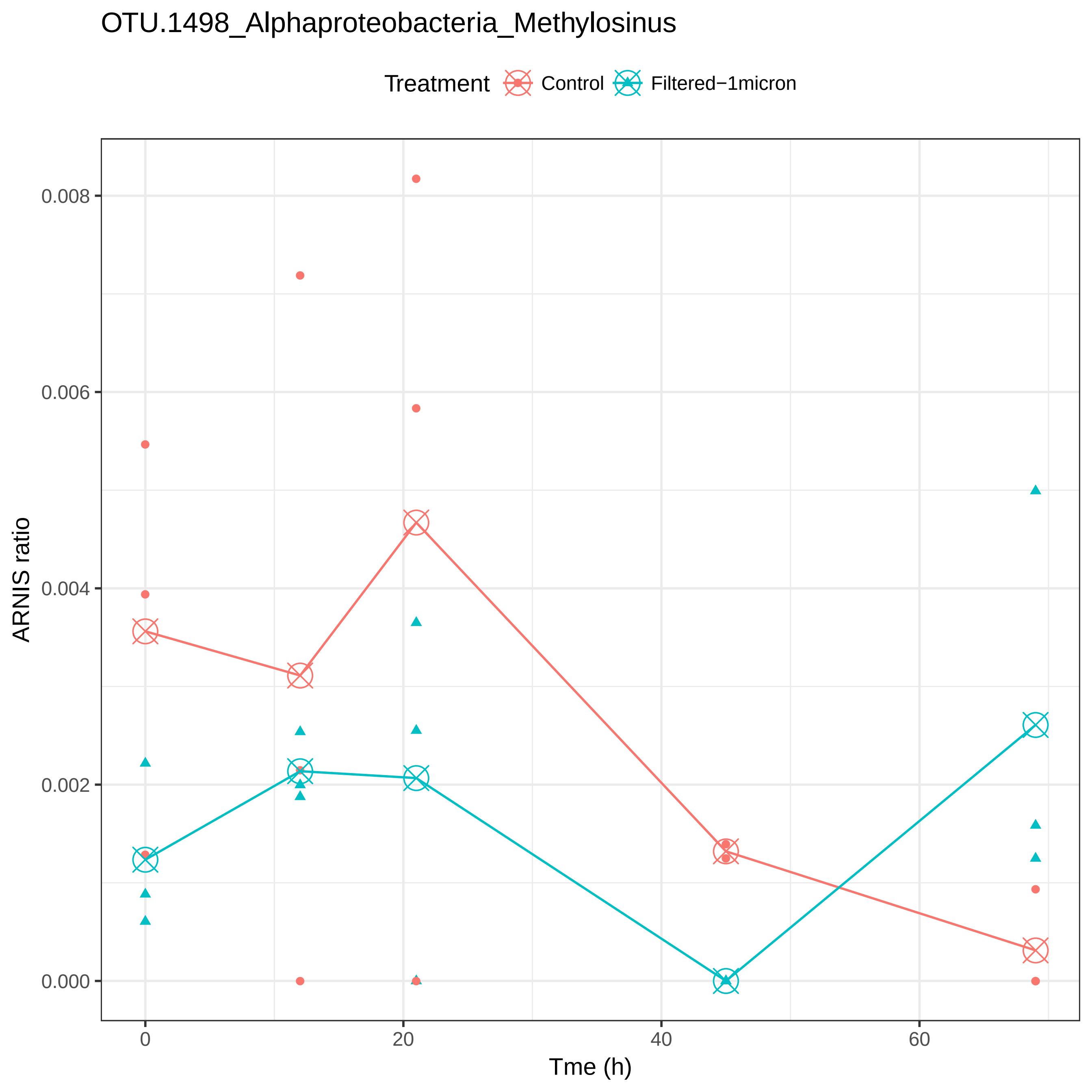

OTU.1498_Alphaproteobacteria_Methylosinus
Treatment
Control
Filtered−1micron
ARNIS ratio
0.000
0.002
0.004
0.006
0.008
0
20
40
60
Tme (h)

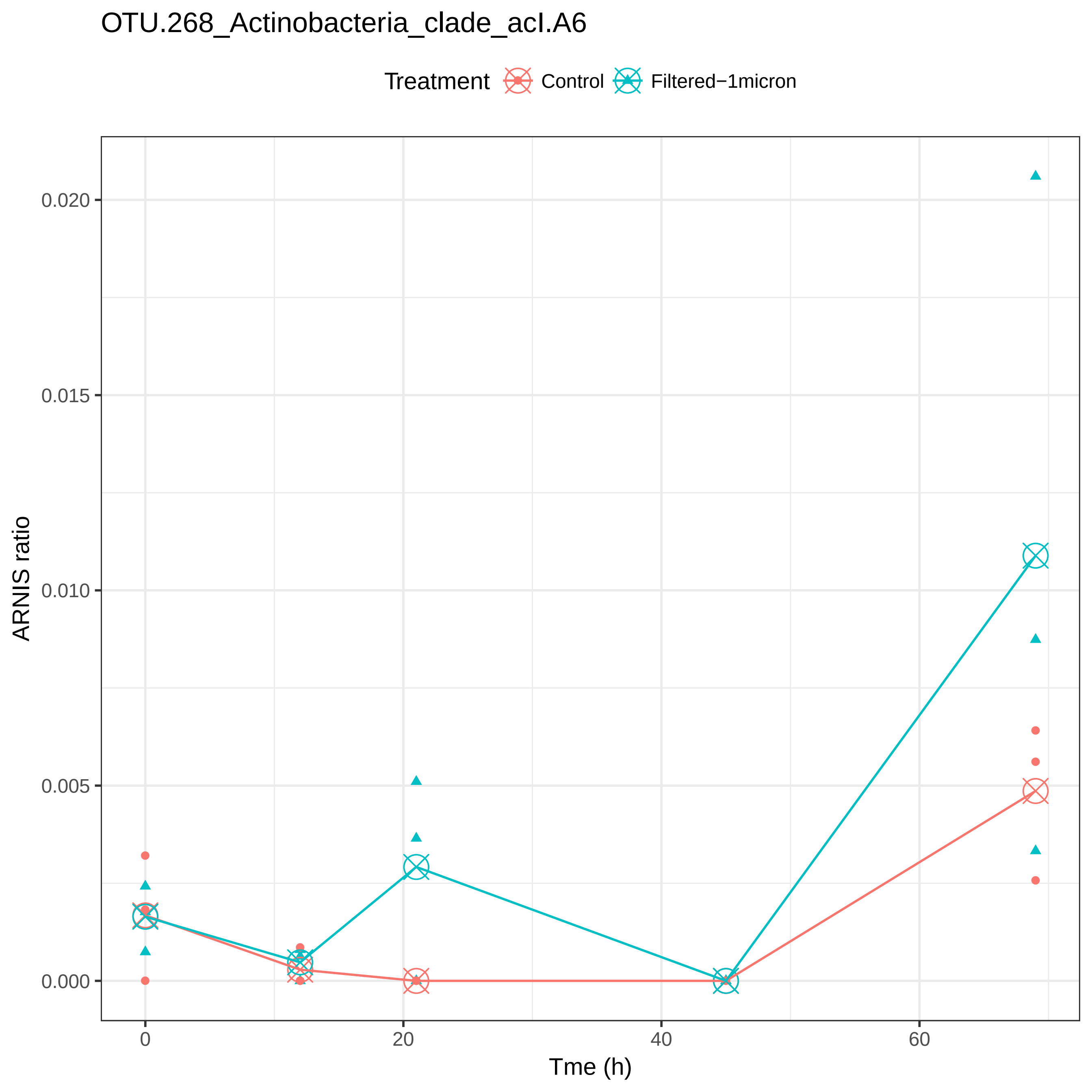

OTU.268_Actinobacteria_clade_acI.A6
Treatment
Control
Filtered−1micron
ARNIS ratio
0.020
0.015
0.010
0.005
0.000
0
20
40
60
Tme (h)

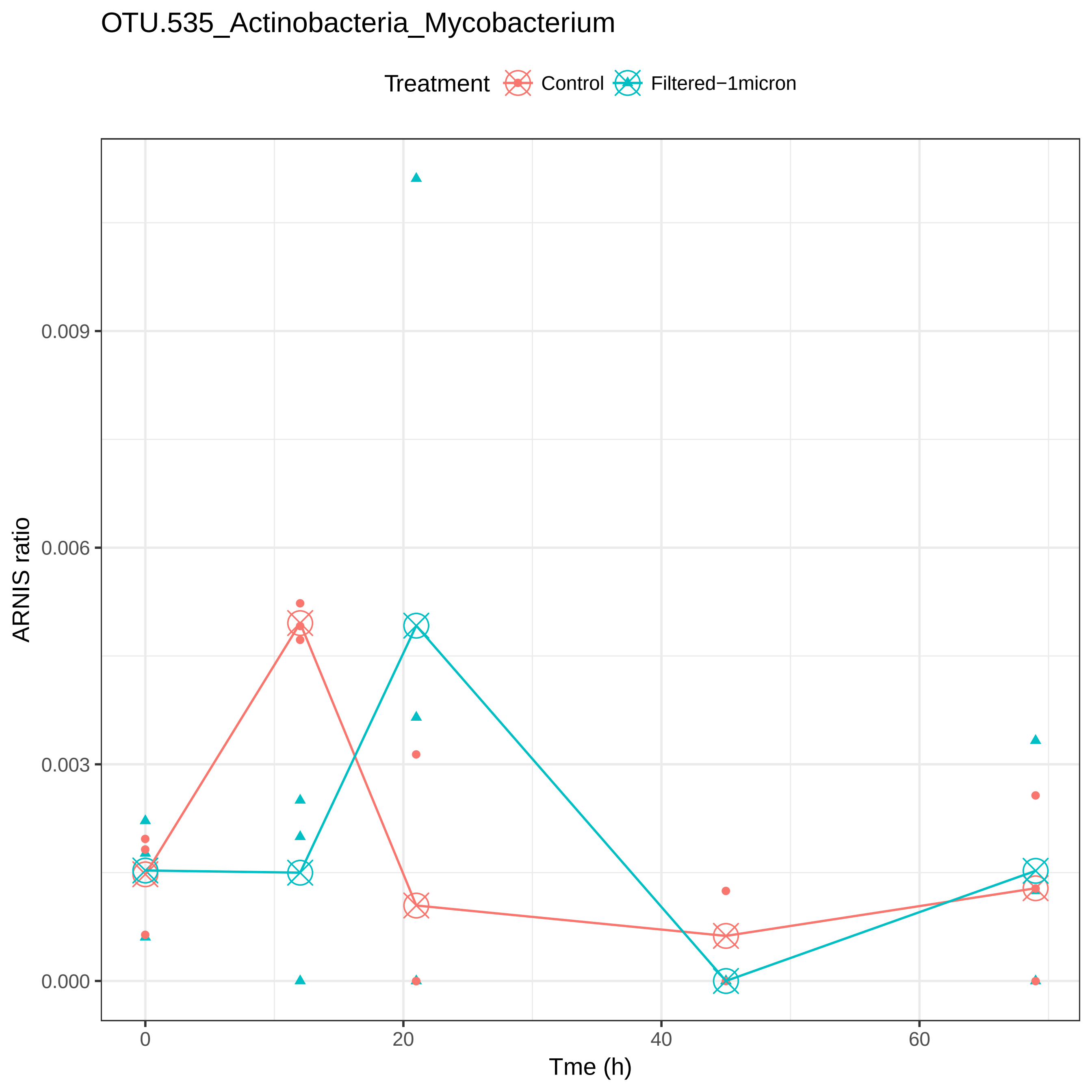

OTU.535_Actinobacteria_Mycobacterium
Treatment
Control
Filtered−1micron
ARNIS ratio
0.009
0.006
0.003
0.000
0
20
40
60
Tme (h)

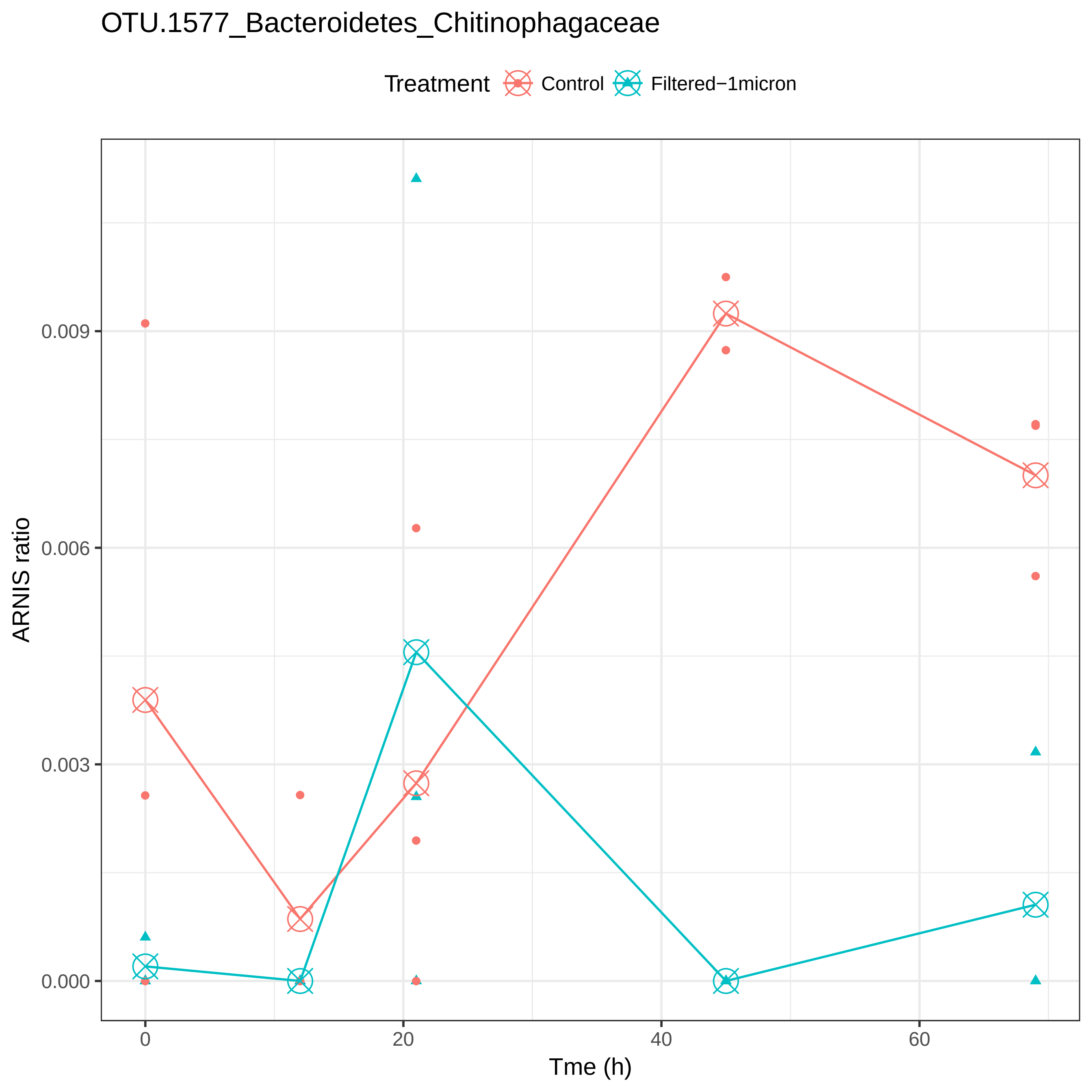
OTU.1577_Bacteroidetes_Chitinophagaceae
Treatment
Control
Filtered−1micron
ARNIS ratio
0.009
0.006
0.003
0.000
0
20
40
60
Tme (h)

OTU.1168_Chloroflexi

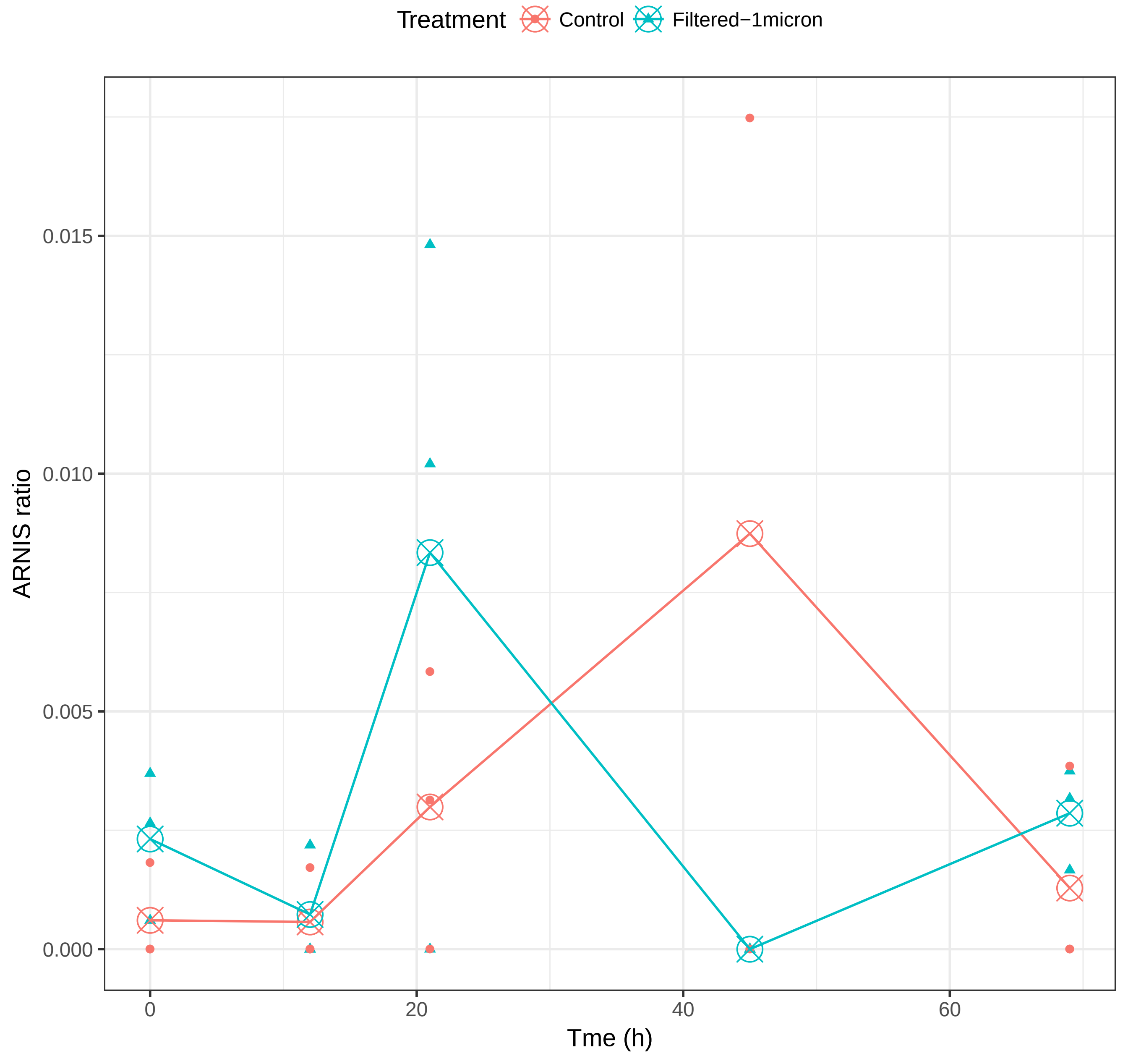

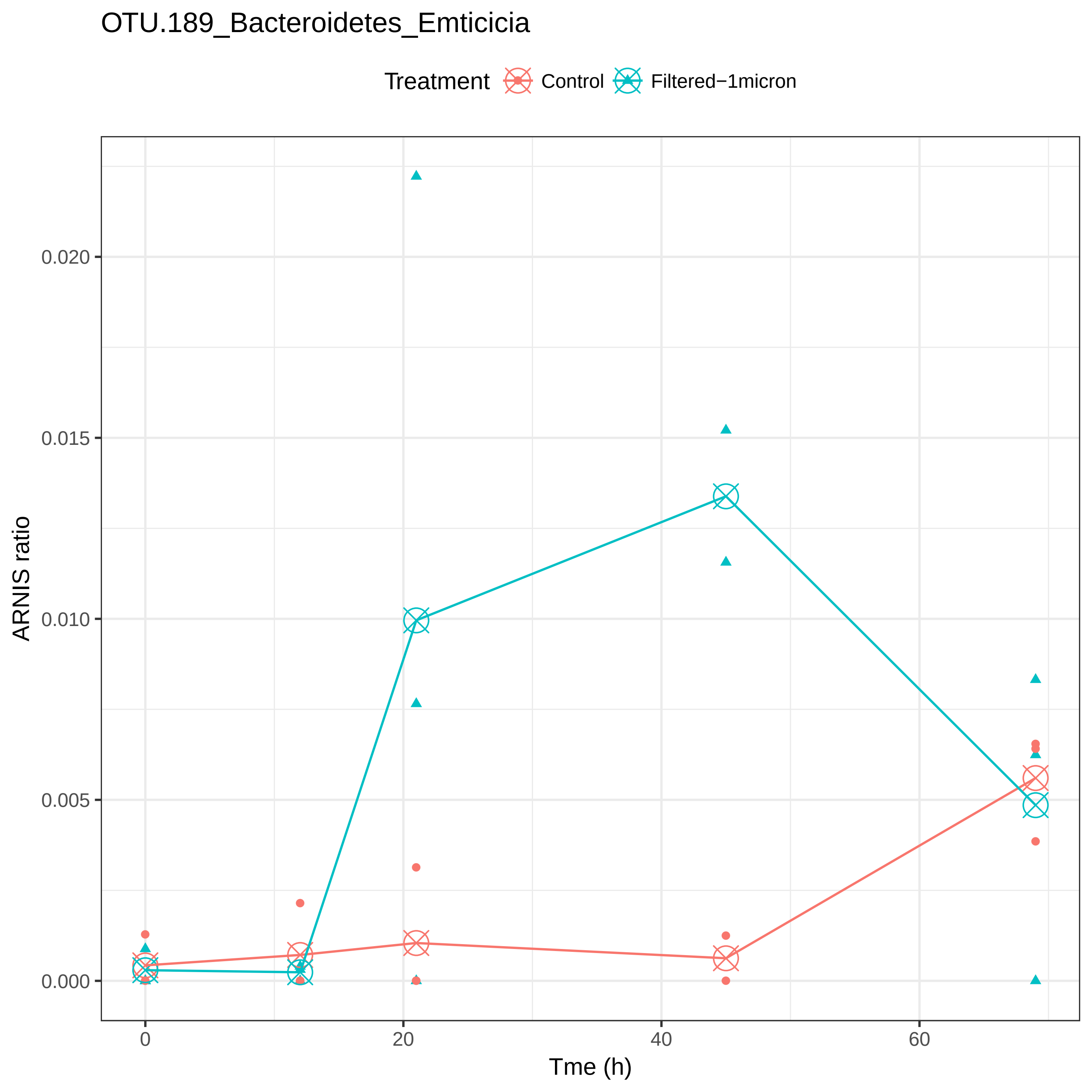

OTU.189_Bacteroidetes_Emticicia
Treatment
Control
Filtered−1micron
ARNIS ratio
0.000
0.005
0.010
0.015
0.020
0
20
40
60
Tme (h)

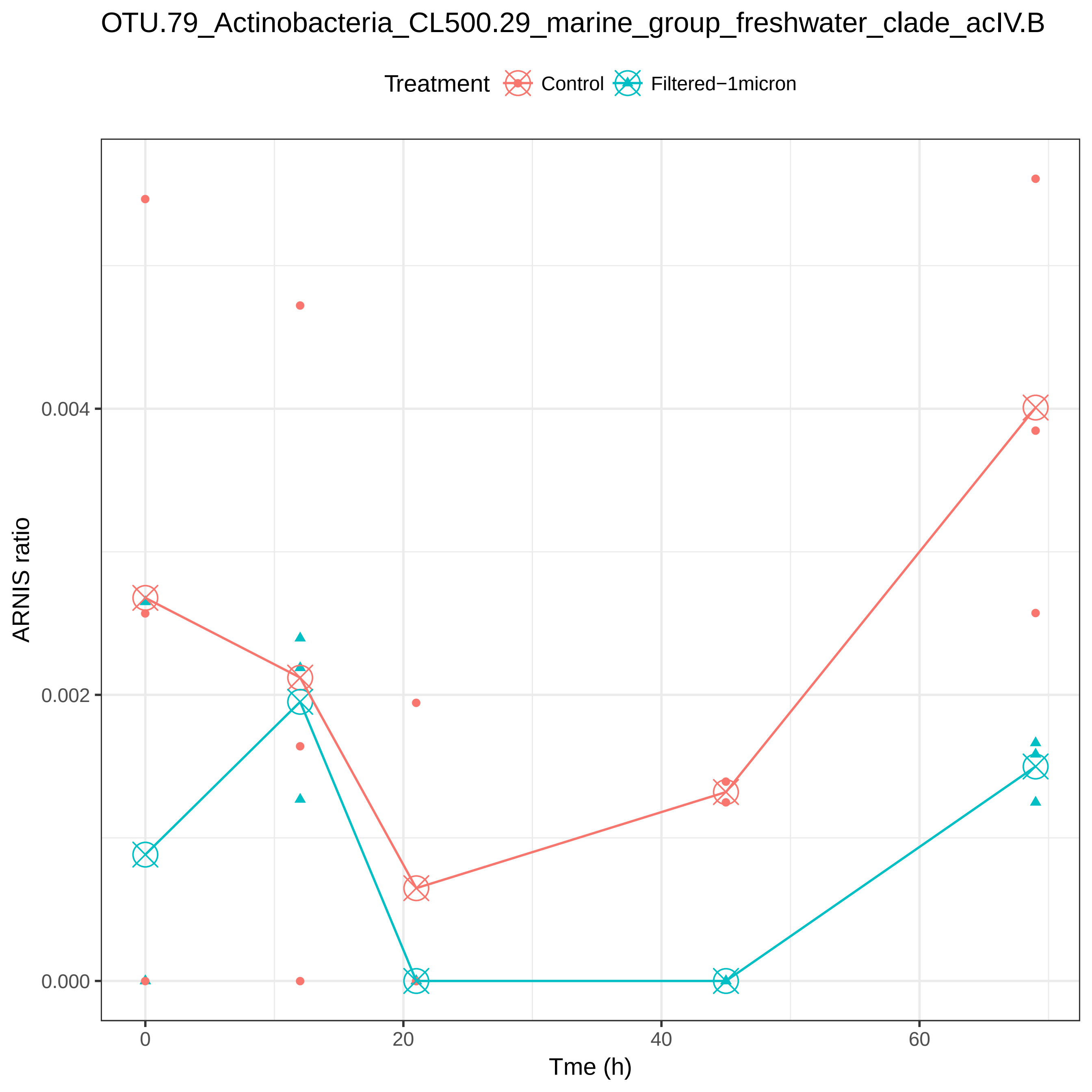
OTU.79_Actinobacteria_CL500.29_marine_group_freshwater_clade_acIV.B
Treatment
Control
Filtered−1micron
ARNIS ratio
0.000
0.002
0.004
0
20
40
60
Tme (h)

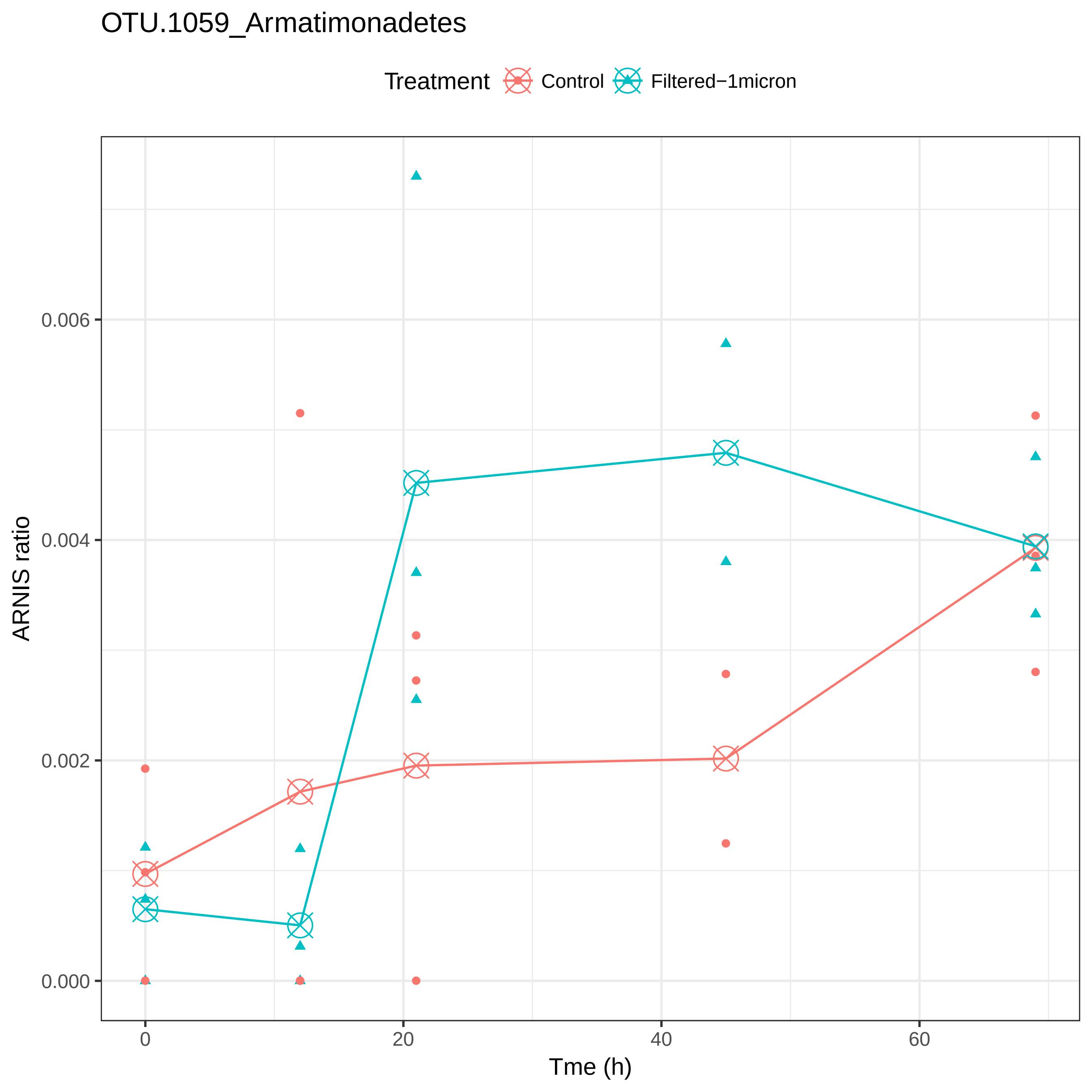

OTU.1059_Armatimonadetes
Treatment
Control
Filtered−1micron
ARNIS ratio
0.000
0.002
0.004
0.006
0
20
40
60
Tme (h)

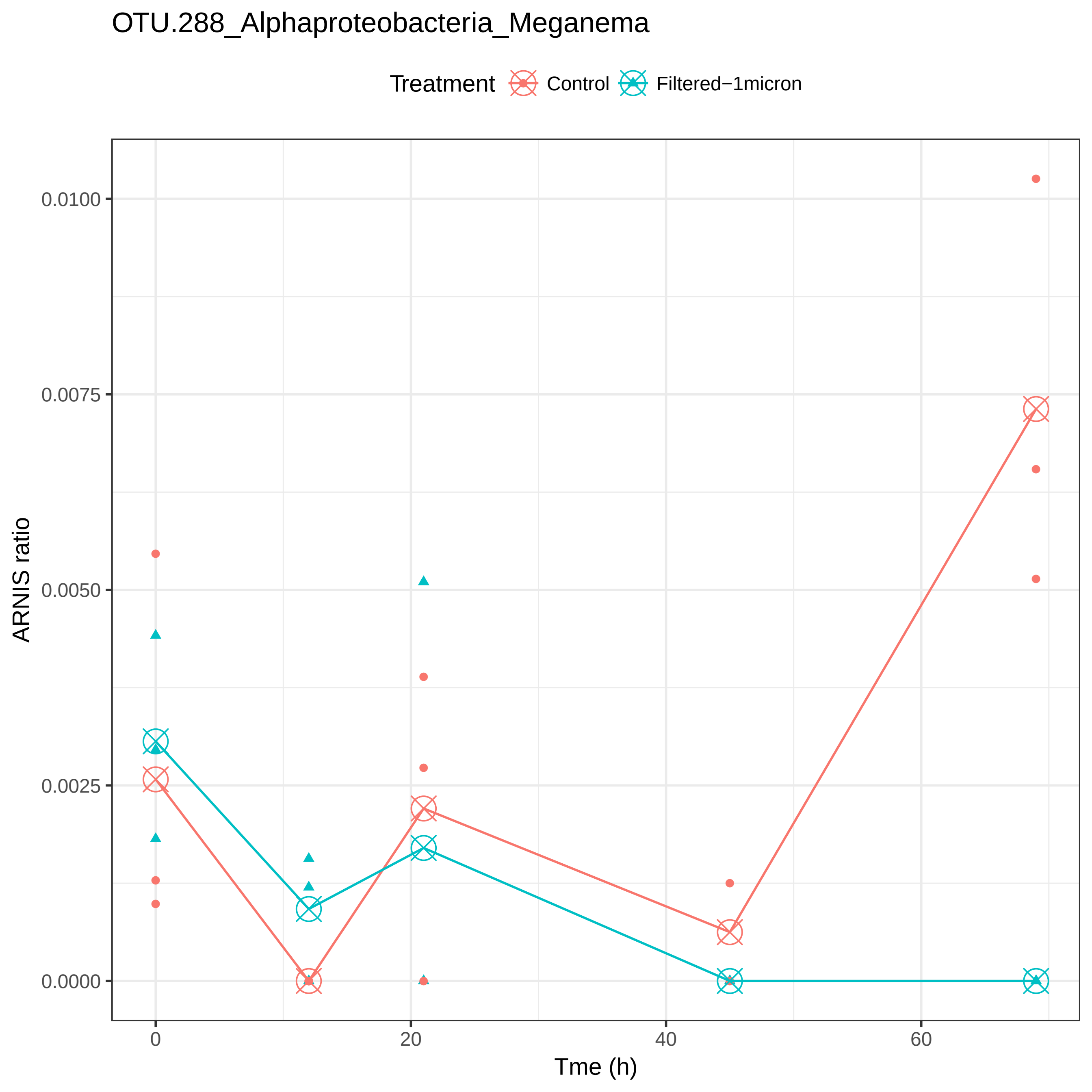
OTU.288_Alphaproteobacteria_Meganema
Treatment
Control
Filtered−1micron
ARNIS ratio
0.0100
0.0075
0.0050
0.0025
0.0000
0
20
40
60
Tme (h)

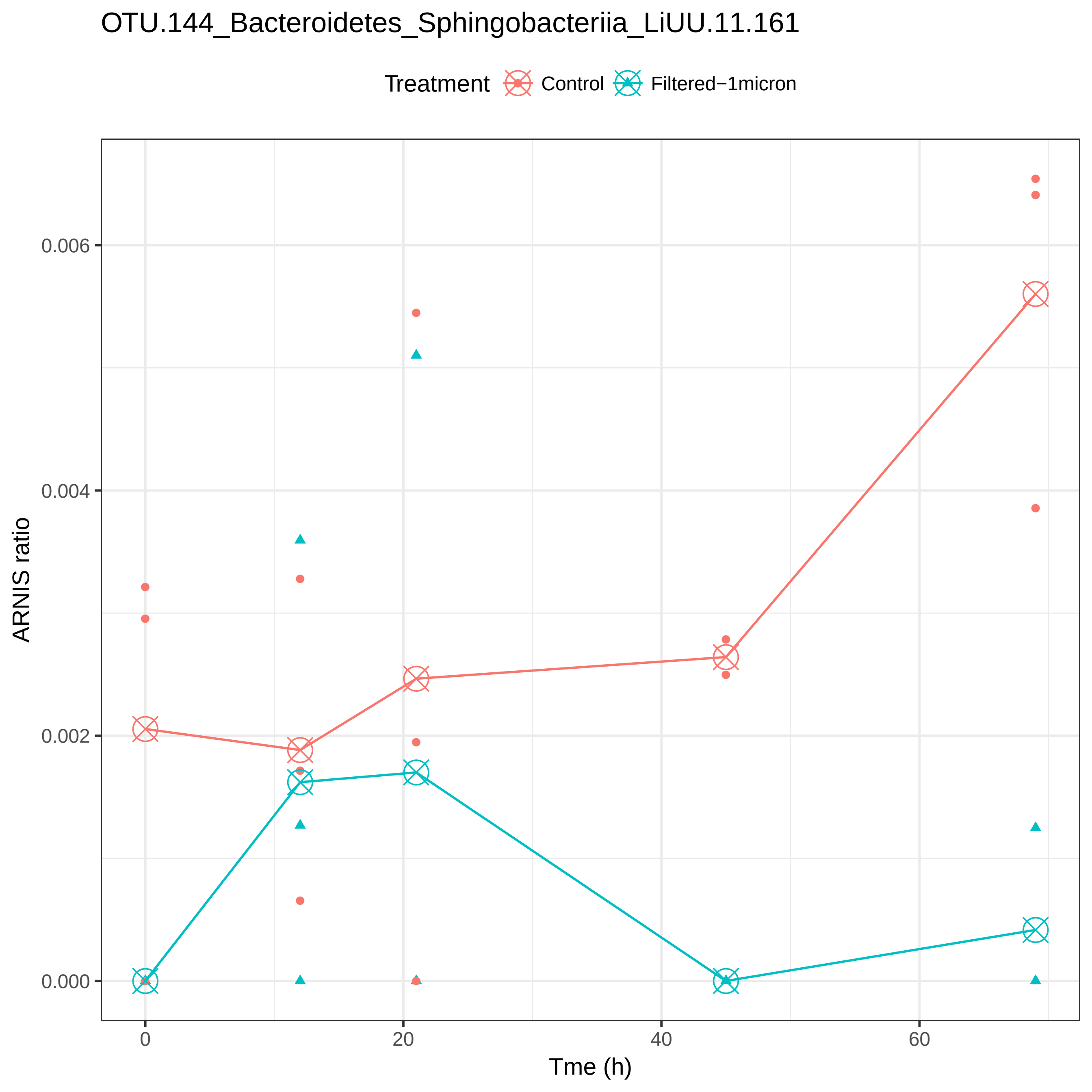

OTU.144_Bacteroidetes_Sphingobacteriia_LiUU.11.161
Treatment
Control
Filtered−1micron
ARNIS ratio
0.000
0.002
0.004
0.006
0
20
40
60
Tme (h)

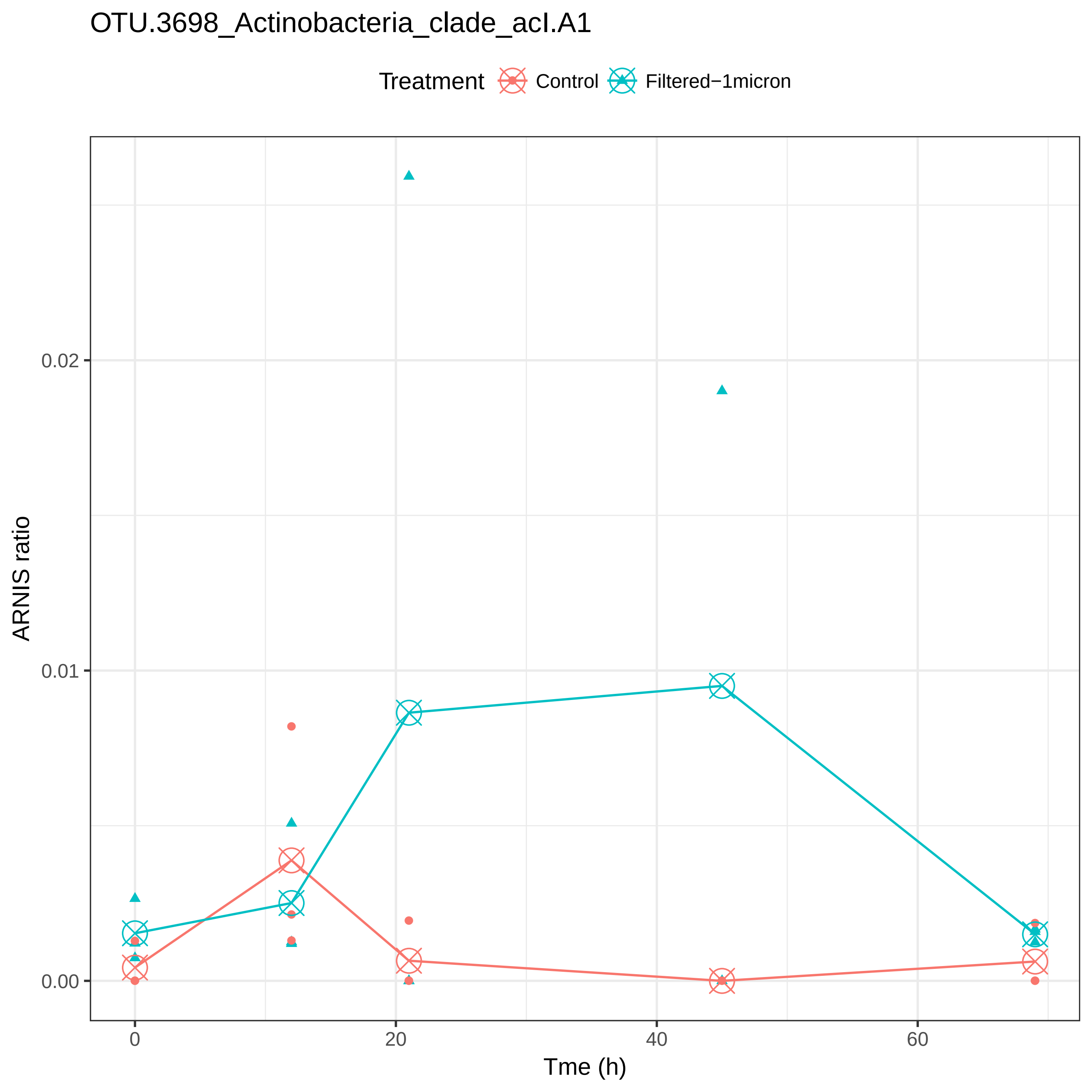
OTU.3698_Actinobacteria_clade_acI.A1
Treatment
Control
Filtered−1micron
ARNIS ratio
0.02
0.01
0.00
0
20
40
60
Tme (h)

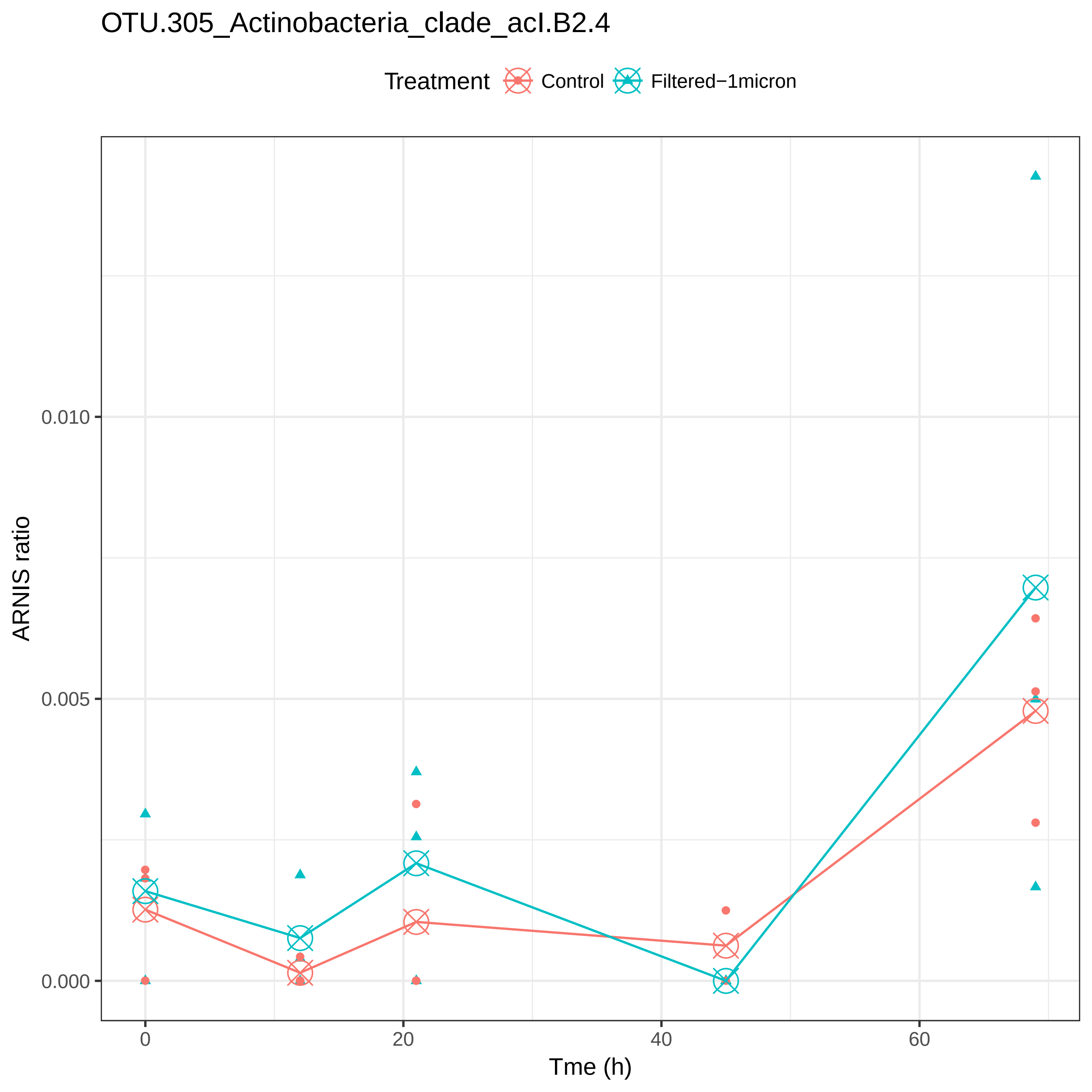

OTU.305_Actinobacteria_clade_acI.B2.4
Treatment
Control
Filtered−1micron
ARNIS ratio
0.010
0.005
0.000
0
20
40
60
Tme (h)

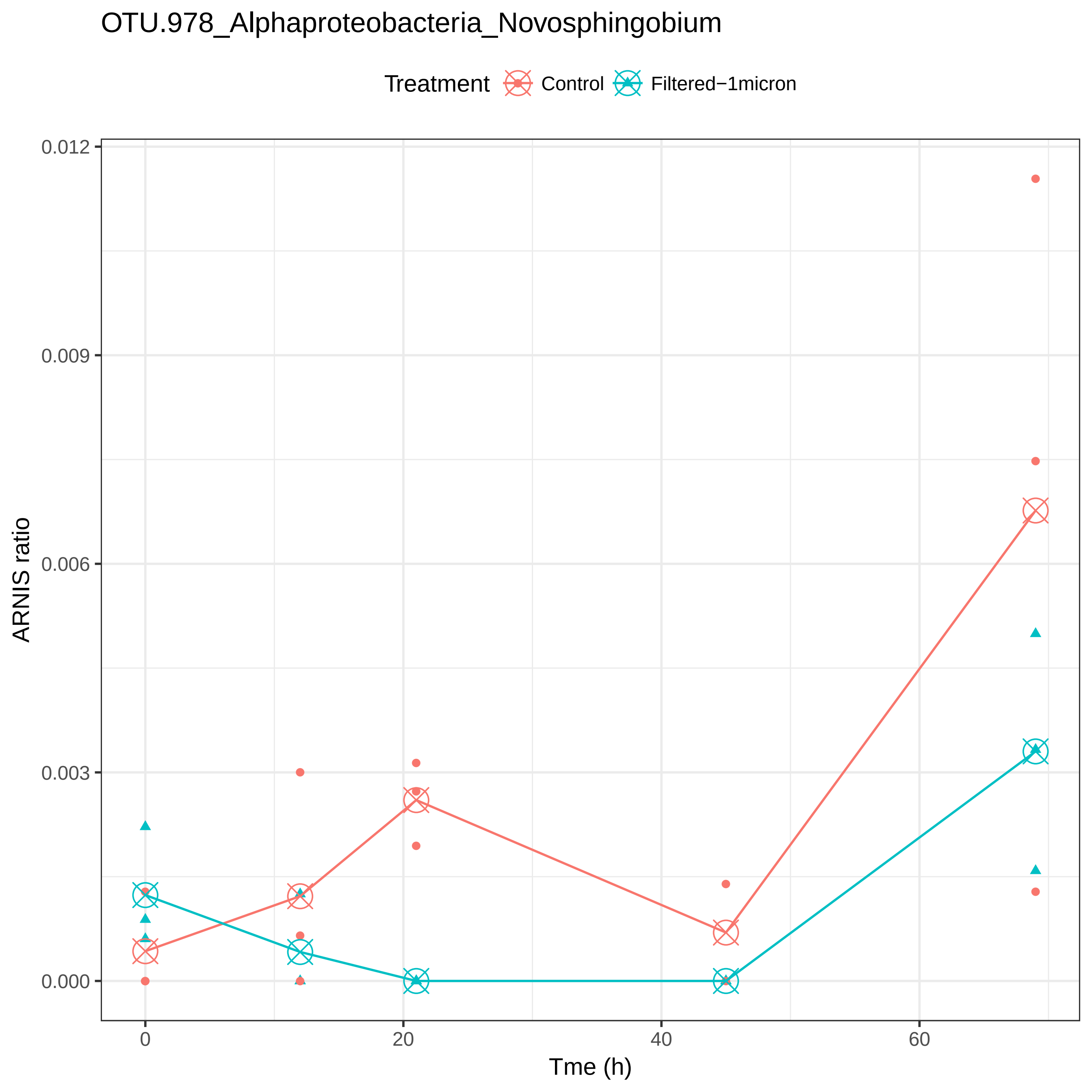

OTU.978_Alphaproteobacteria_Novosphingobium
Treatment
Control
Filtered−1micron
0.012
0.009
0.006
0.003
0.000
ARNIS ratio
0
20
40
60
Tme (h)

OTU.2951_Betaproteobacteria_Variovorax

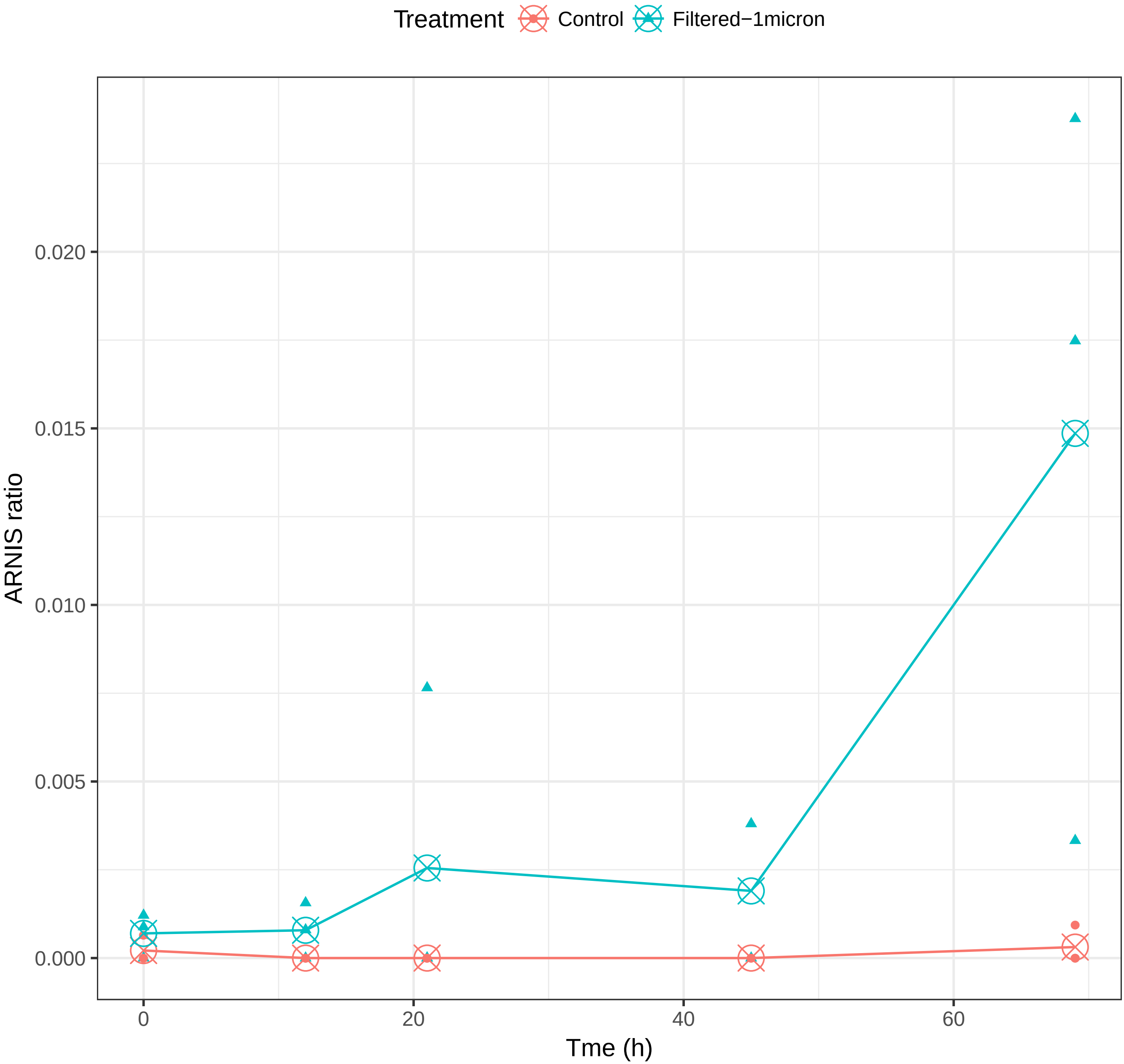

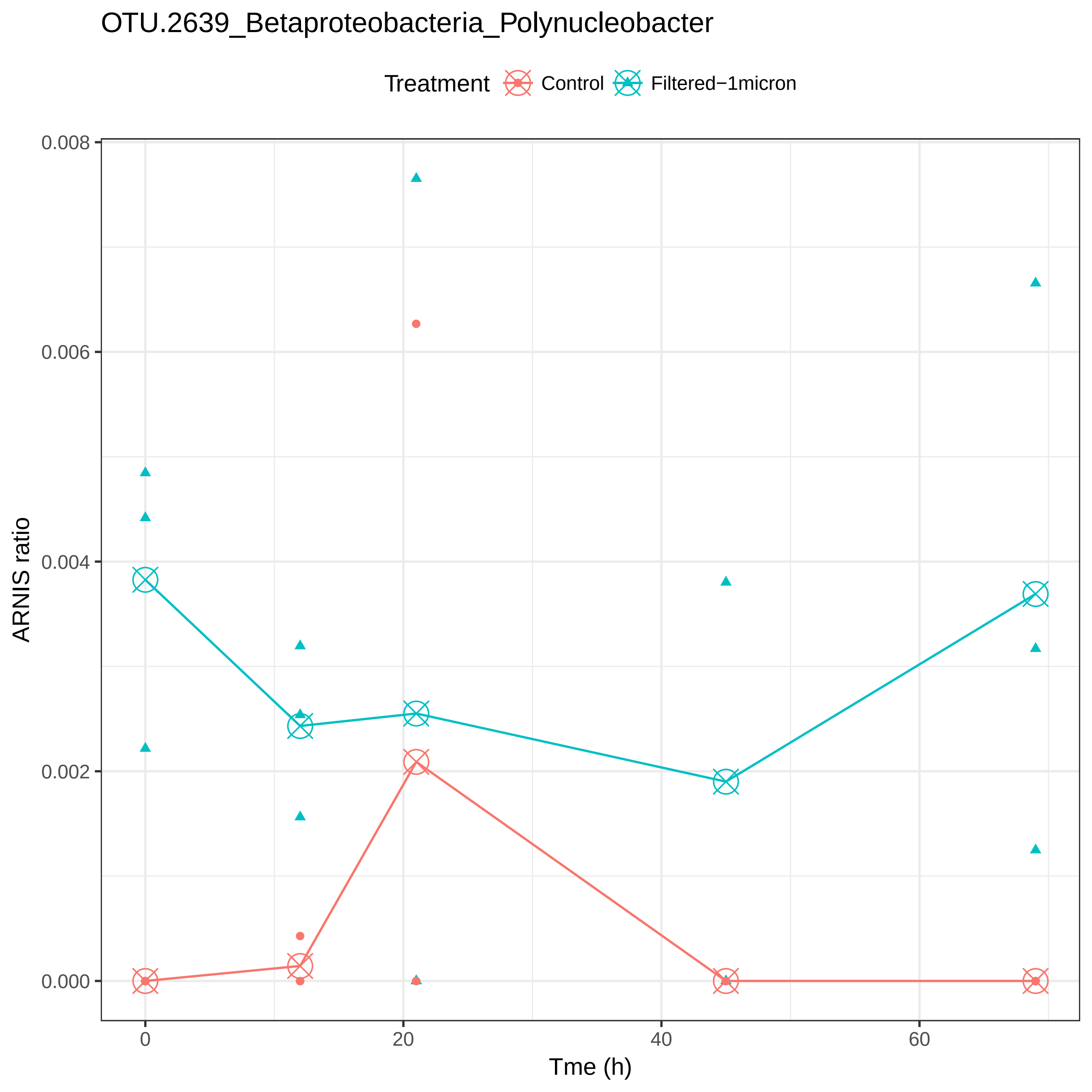

OTU.2639_Betaproteobacteria_Polynucleobacter
Treatment
Control
Filtered−1micron
0.008
0.006
0.004
0.002
0.000
ARNIS ratio
0
20
40
60
Tme (h)

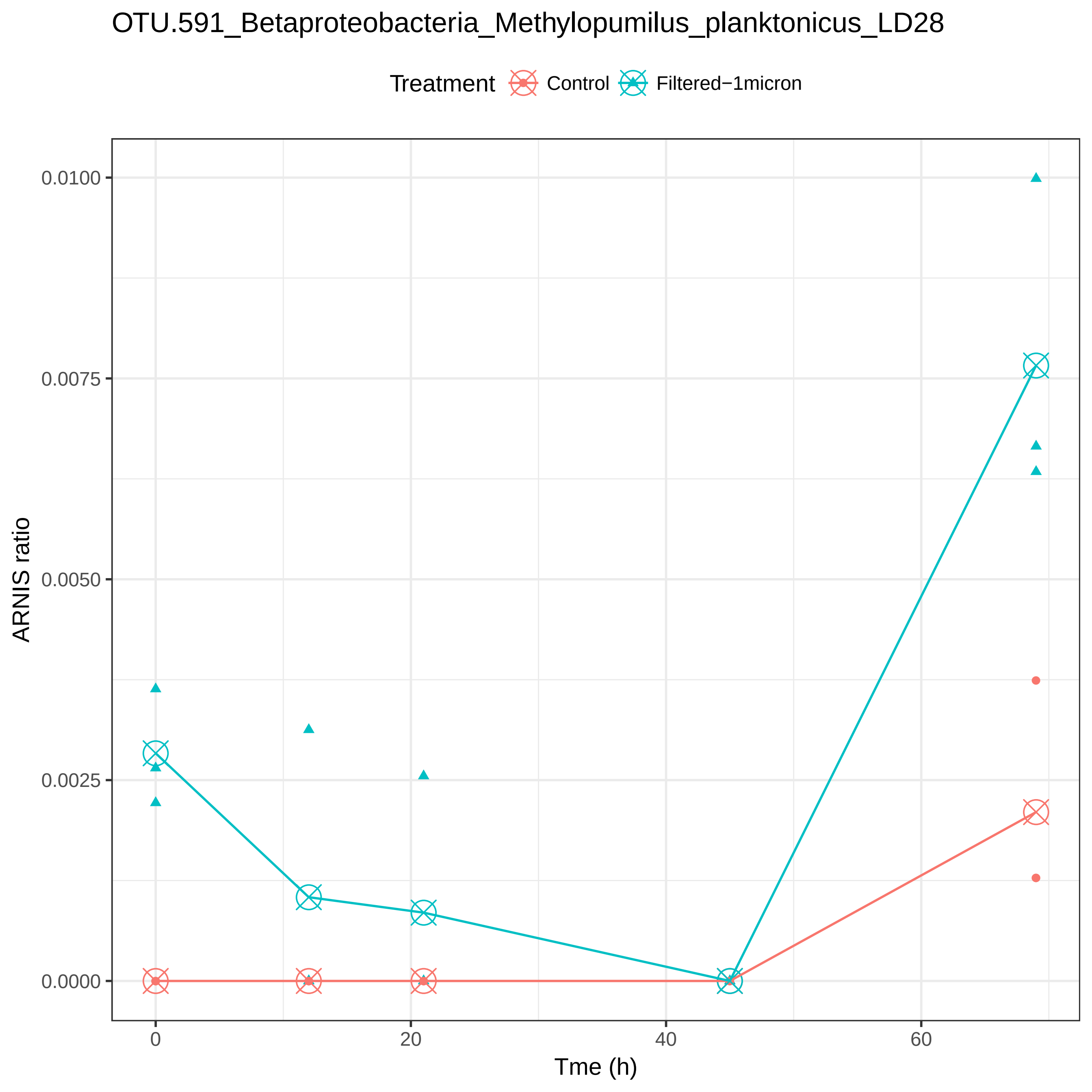

OTU.591_Betaproteobacteria_Methylopumilus_planktonicus_LD28
Treatment
Control
Filtered−1micron
ARNIS ratio
0.0100
0.0075
0.0050
0.0025
0.0000
0
20
40
60
Tme (h)

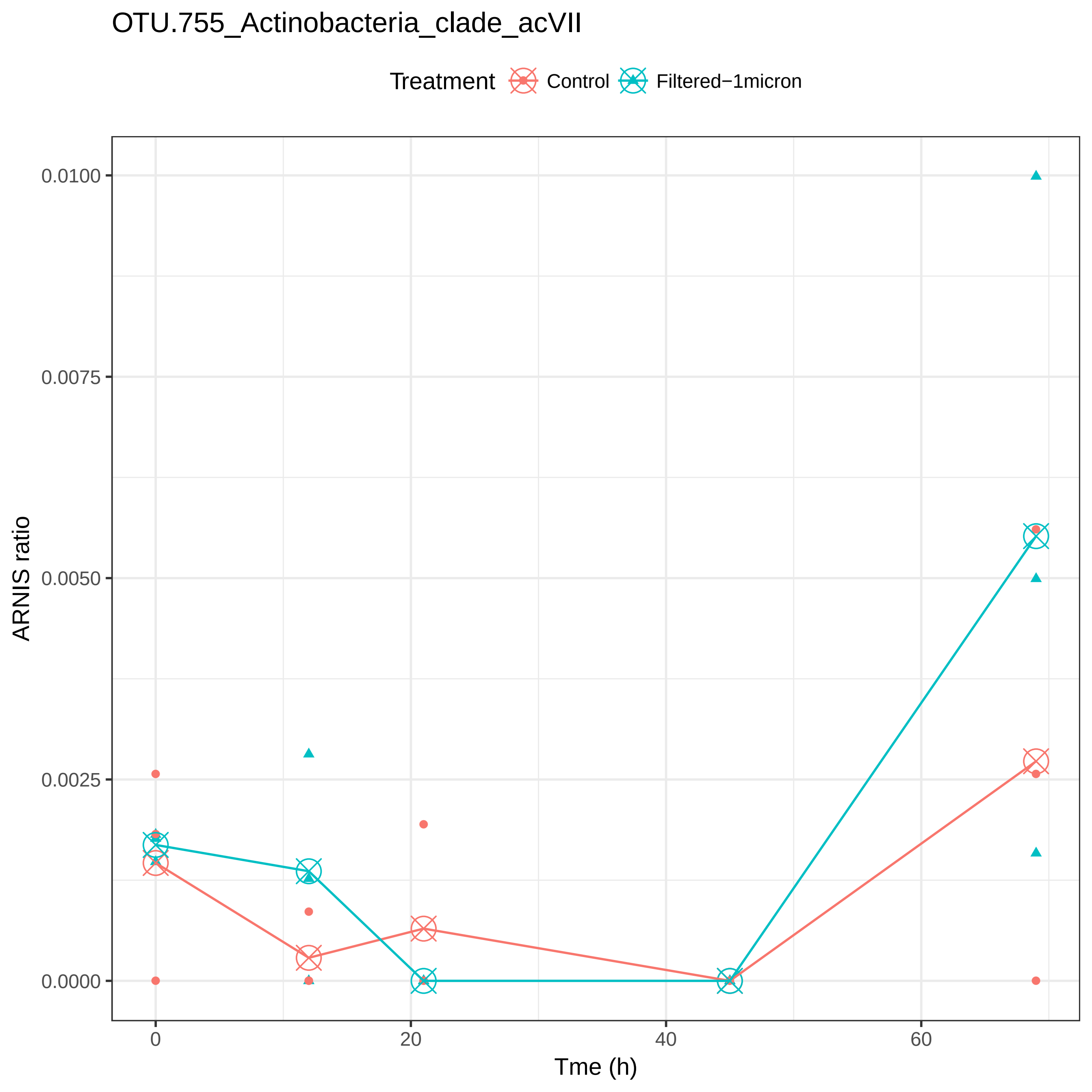

OTU.755_Actinobacteria_clade_acVII
Treatment
Control
Filtered−1micron
ARNIS ratio
0.0100
0.0075
0.0050
0.0025
0.0000
0
20
40
60
Tme (h)

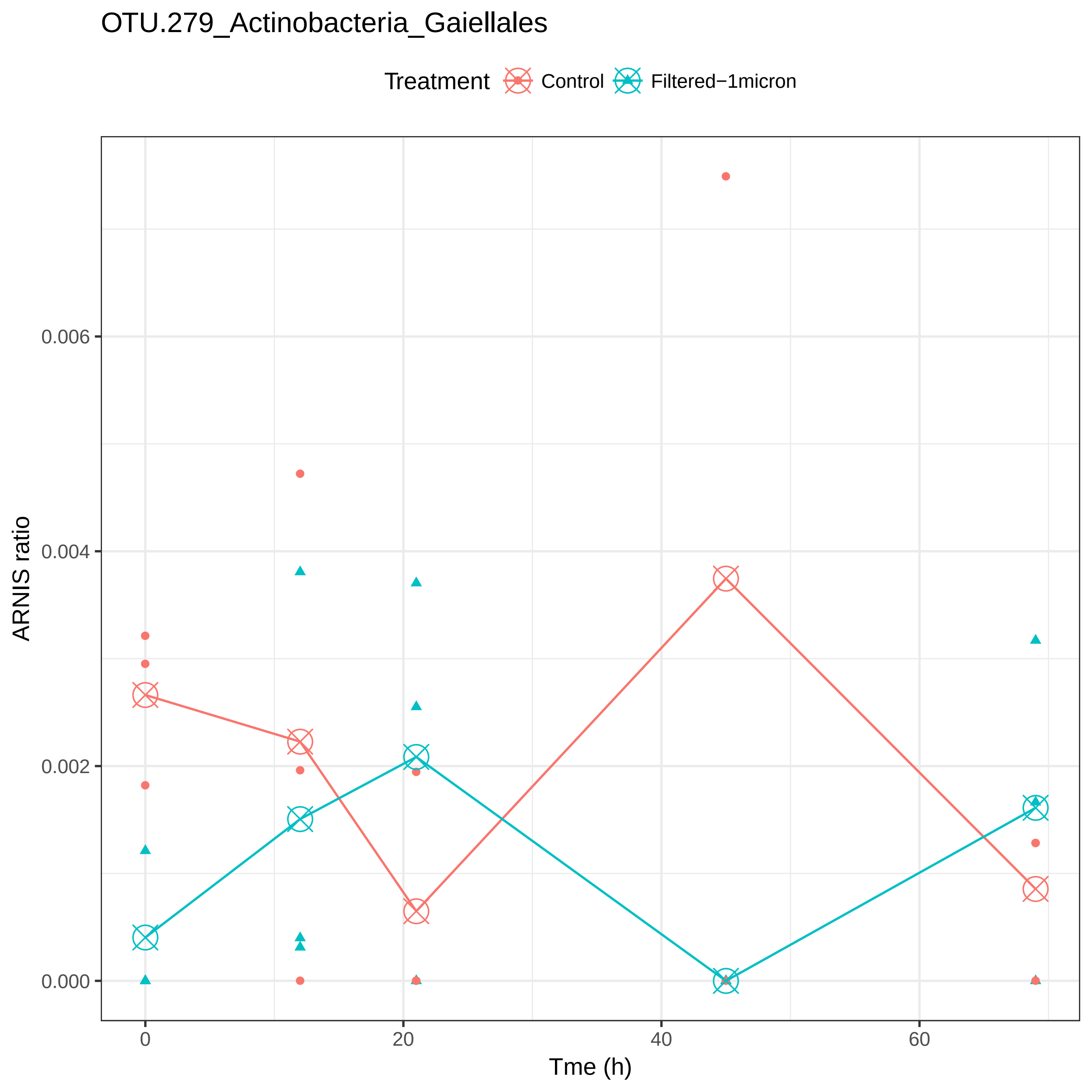

OTU.279_Actinobacteria_Gaiellales
Treatment
Control
Filtered−1micron
0.006
0.004
0.002
0.000
ARNIS ratio
0
20
40
60
Tme (h)

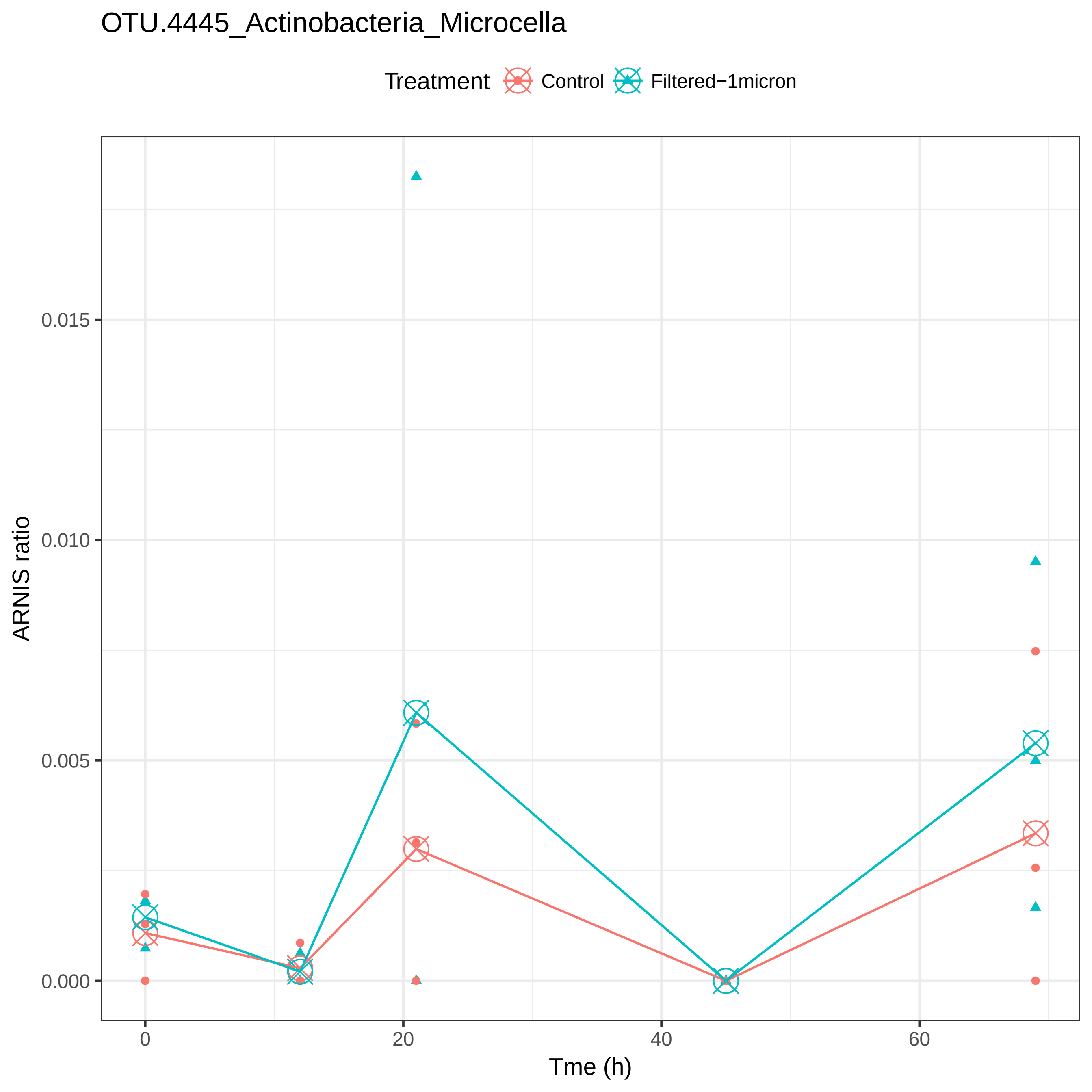
OTU.4445_Actinobacteria_Microcella
Treatment
Control
Filtered−1micron
ARNIS ratio
0.015
0.010
0.005
0.000
0
20
40
60
Tme (h)

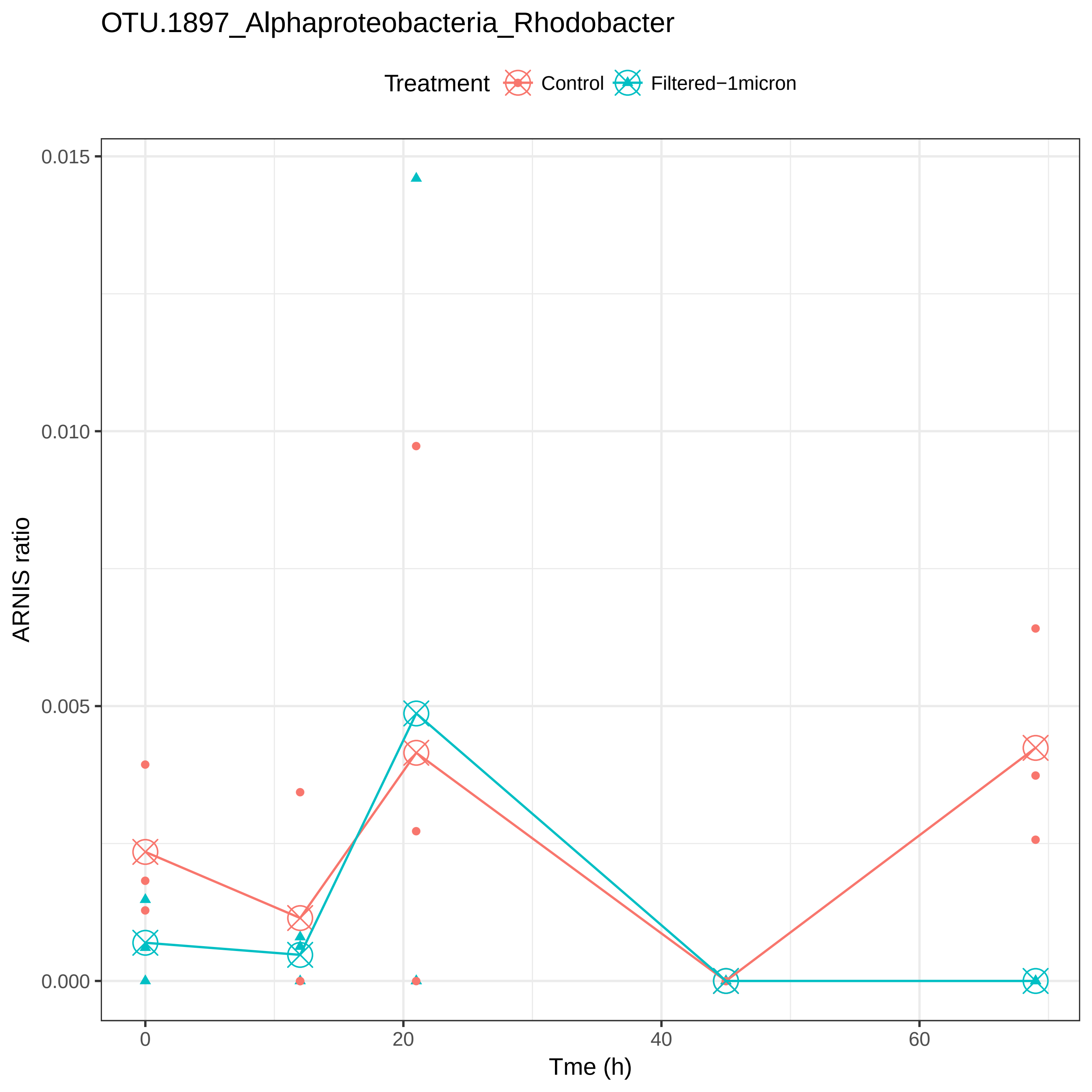

OTU.1897_Alphaproteobacteria_Rhodobacter
Treatment
Control
Filtered−1micron
0.015
0.010
0.005
0.000
ARNIS ratio
0
20
40
60
Tme (h)

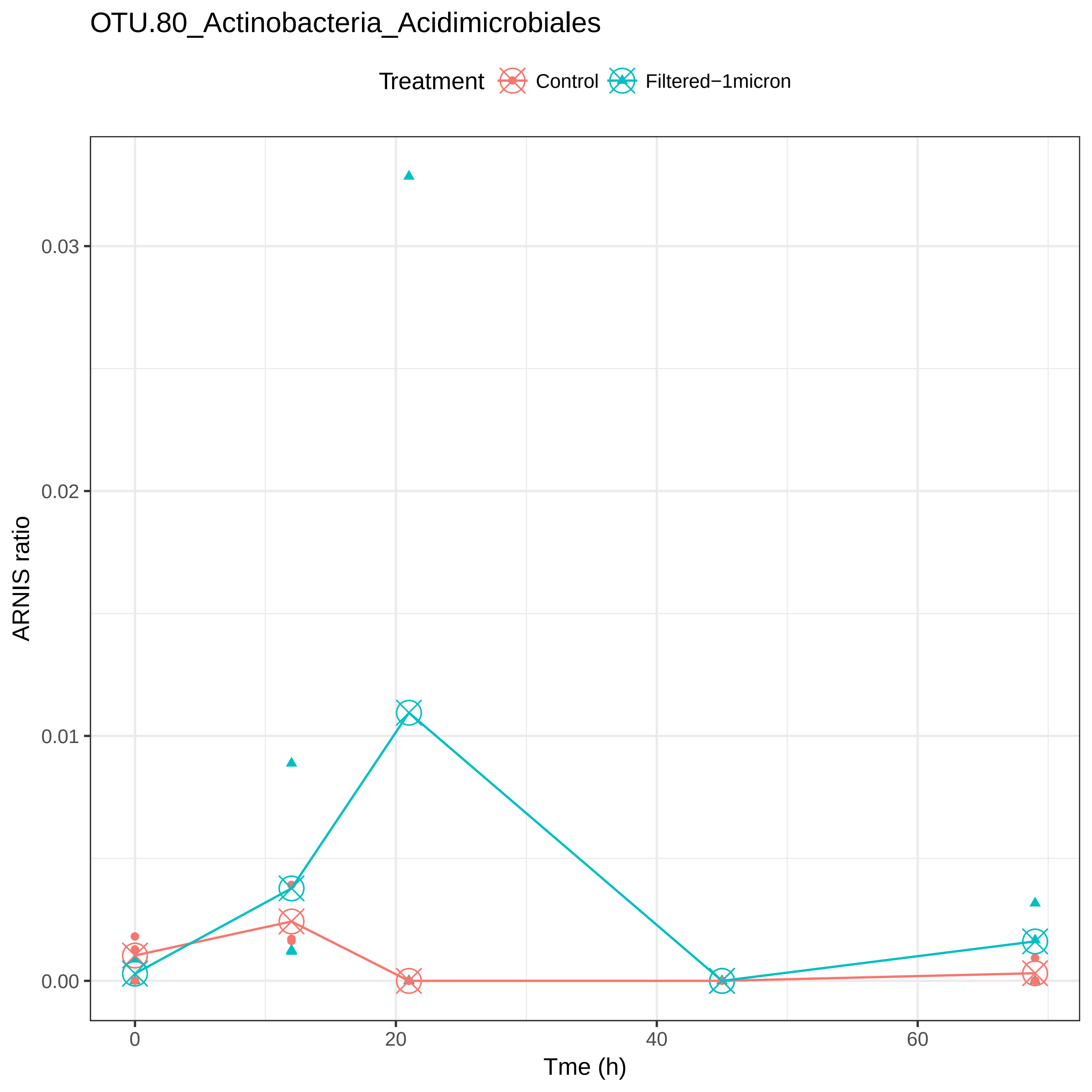

OTU.80_Actinobacteria_Acidimicrobiales
Treatment
Control
Filtered−1micron
ARNIS ratio
0.03
0.02
0.01
0.00
0
20
40
60
Tme (h)

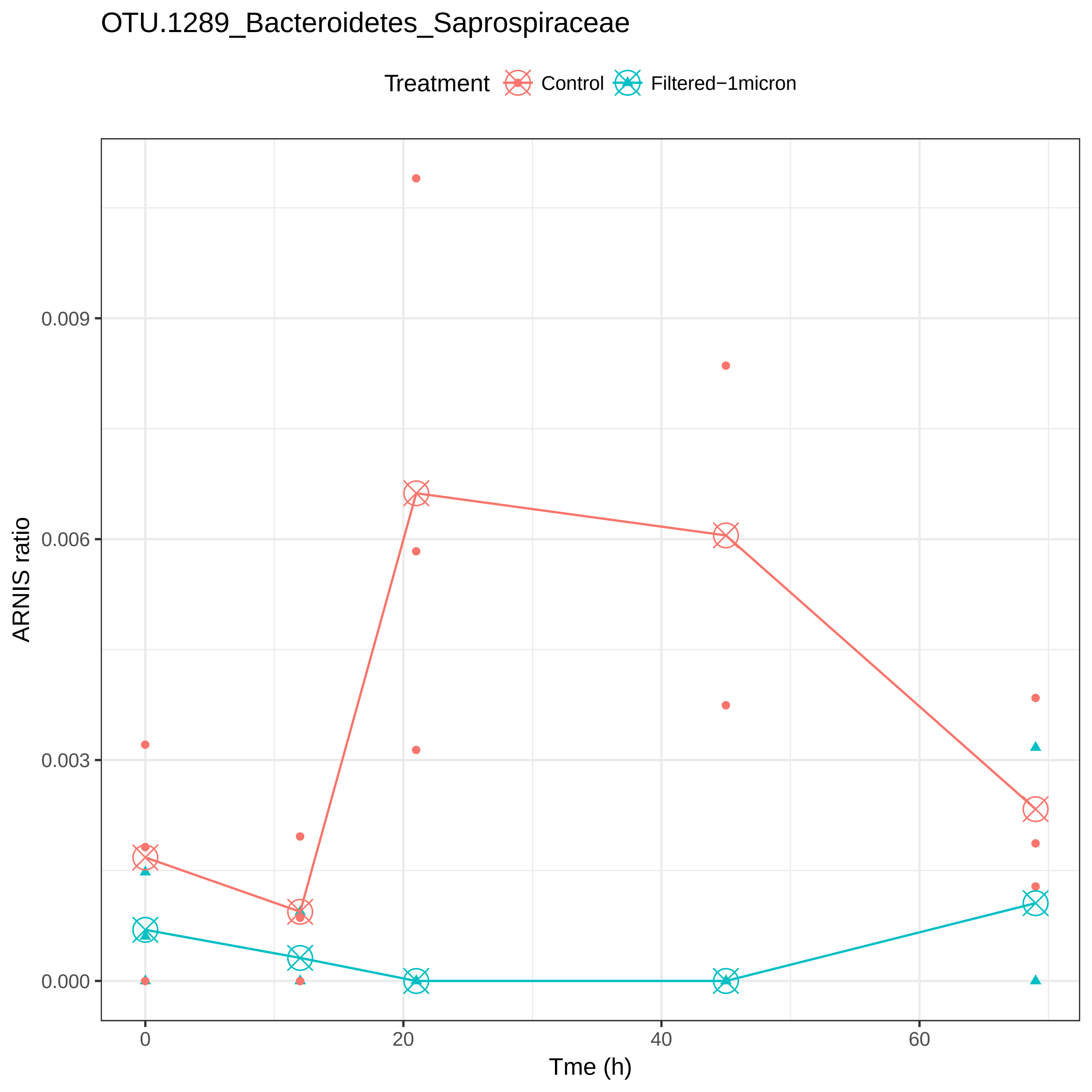

OTU.1289_Bacteroidetes_Saprospiraceae
Treatment
Control
Filtered−1micron
ARNIS ratio
0.009
0.006
0.003
0.000
0
20
40
60
Tme (h)

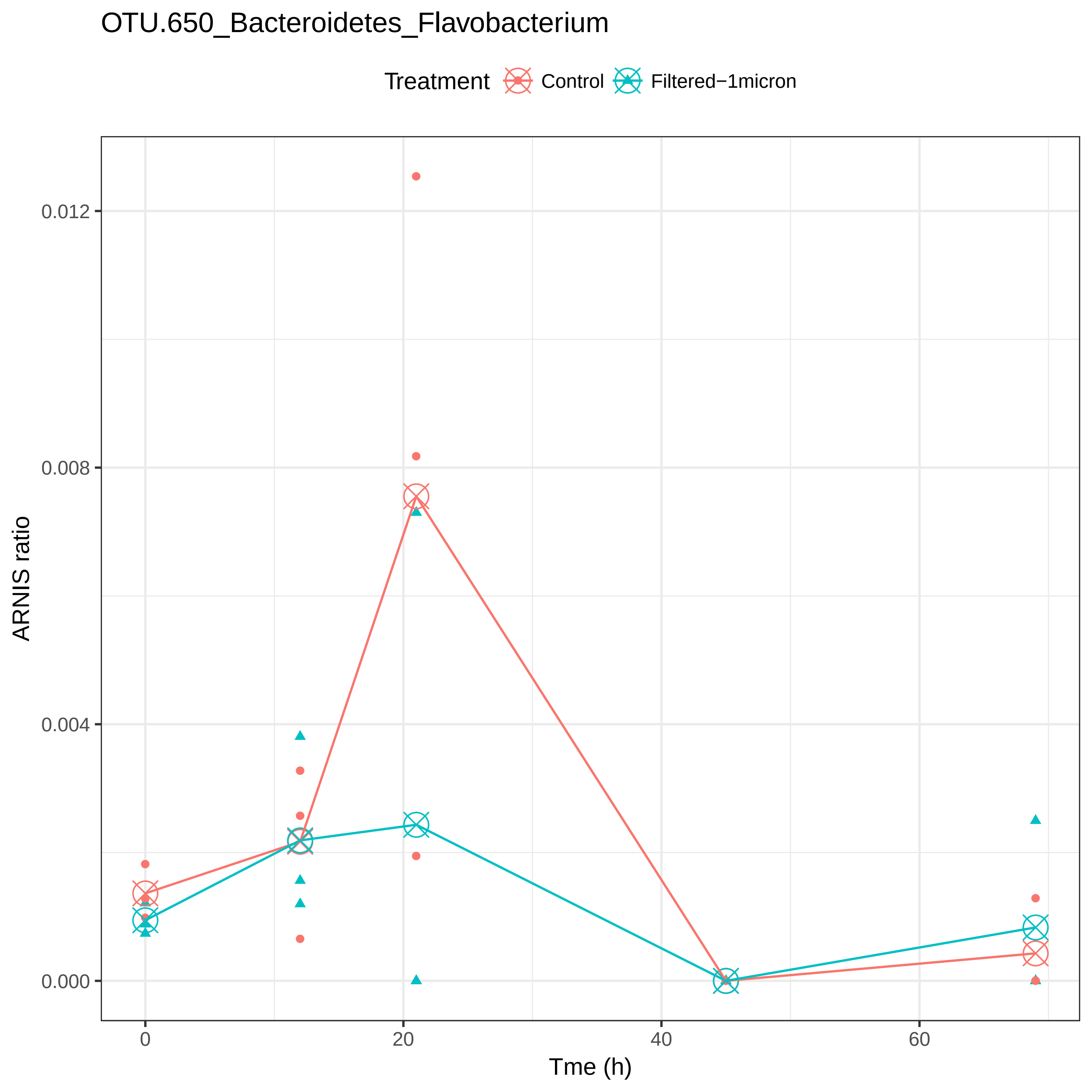

OTU.650_Bacteroidetes_Flavobacterium
Treatment
Control
Filtered−1micron
ARNIS ratio
0.012
0.008
0.004
0.000
0
20
40
60
Tme (h)

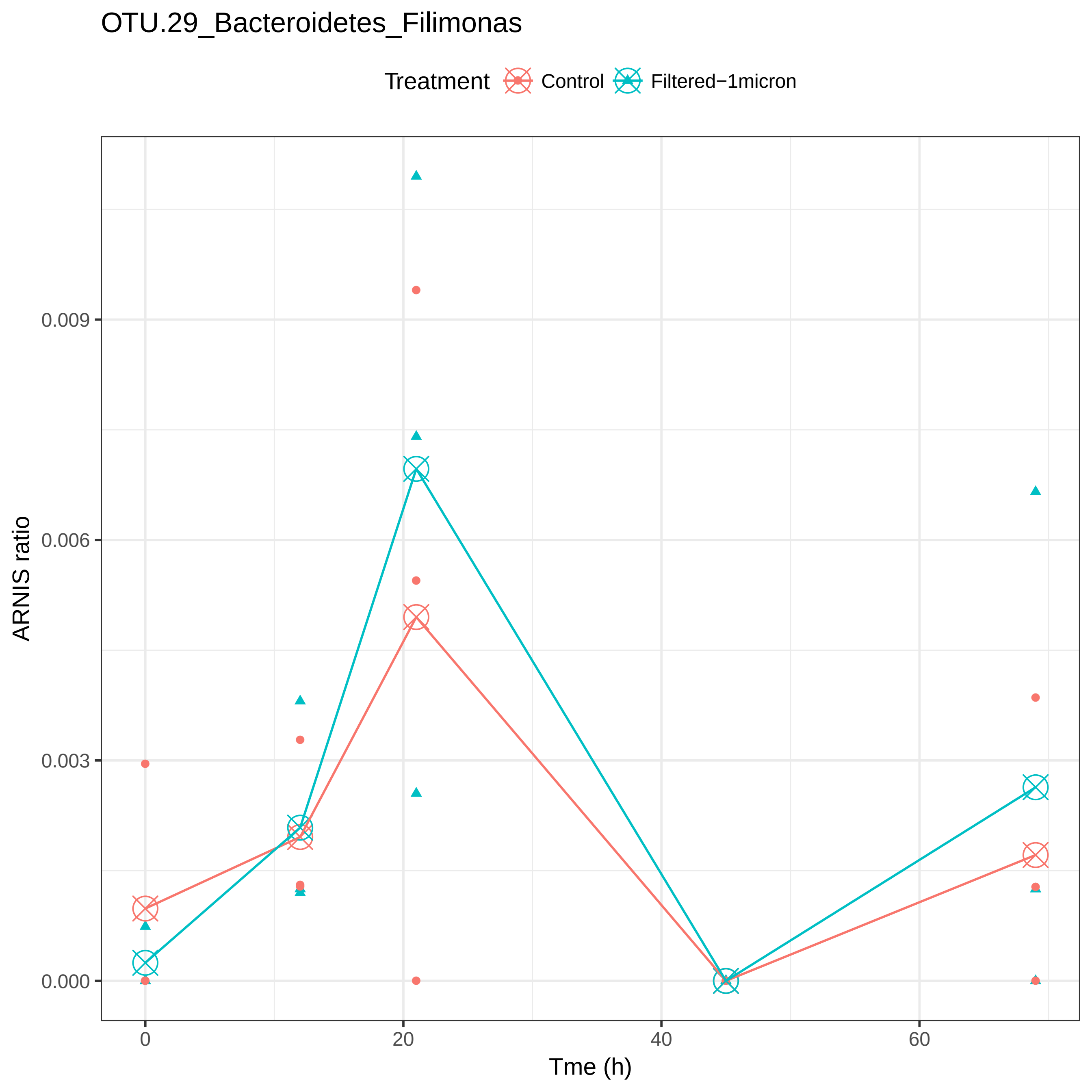

OTU.29_Bacteroidetes_Filimonas
Treatment
Control
Filtered−1micron
ARNIS ratio
0.009
0.006
0.003
0.000
0
20
40
60
Tme (h)

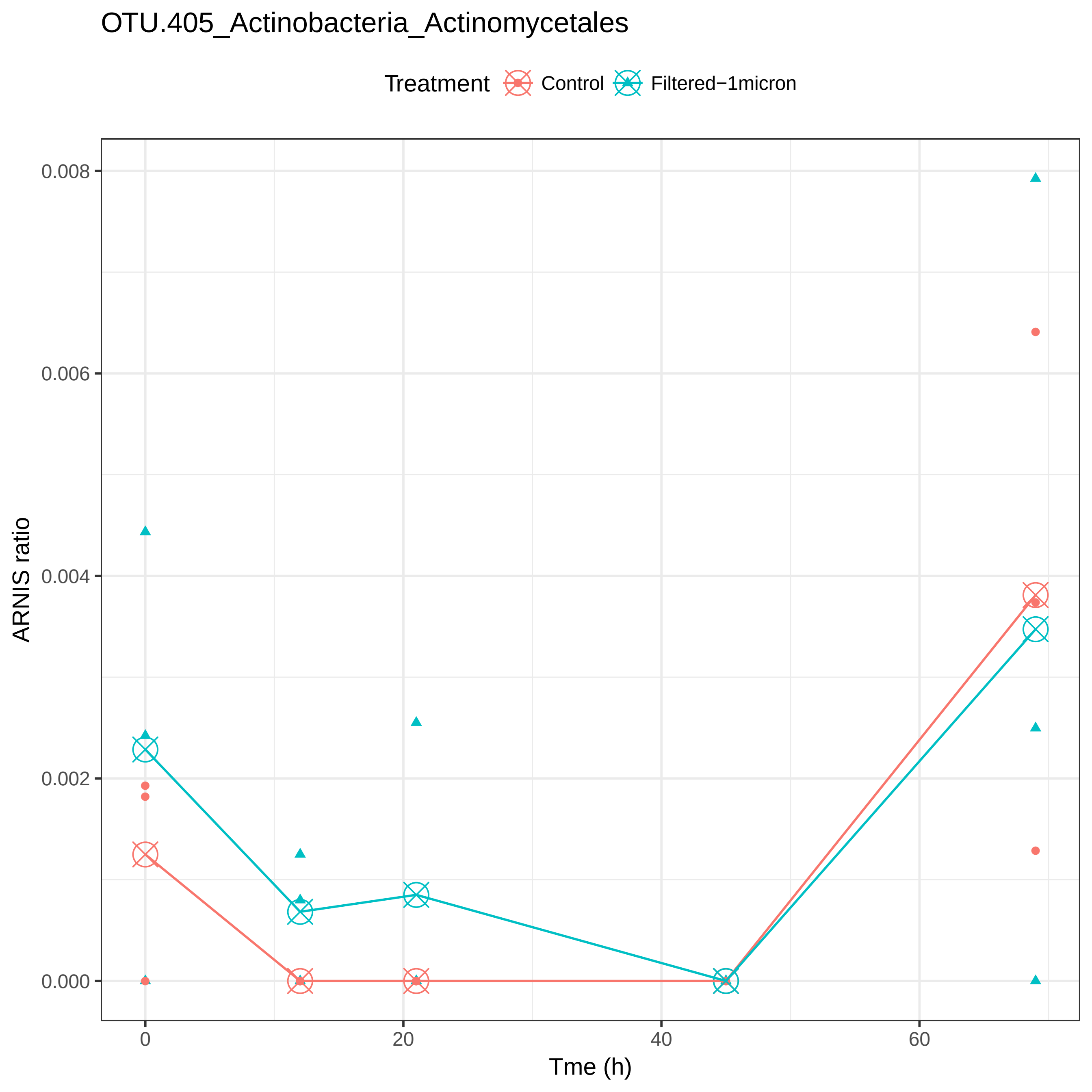
OTU.405_Actinobacteria_Actinomycetales
Treatment
Control
Filtered−1micron
0.008
0.006
0.004
0.002
0.000
ARNIS ratio
0
20
40
60
Tme (h)

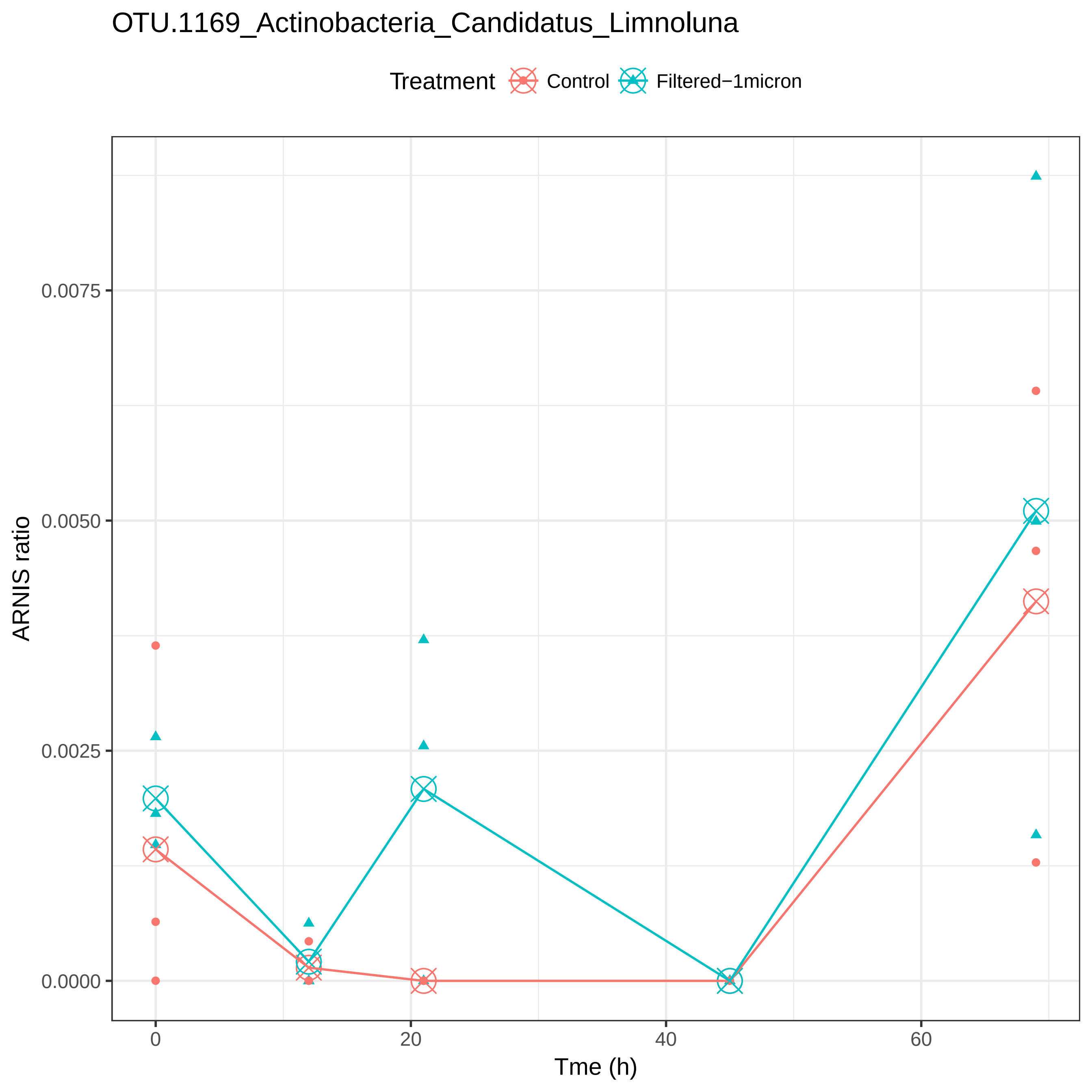
OTU.1169_Actinobacteria_Candidatus_Limnoluna
Treatment
Control
Filtered−1micron
ARNIS ratio
0.0075
0.0050
0.0025
0.0000
0
20
40
60
Tme (h)

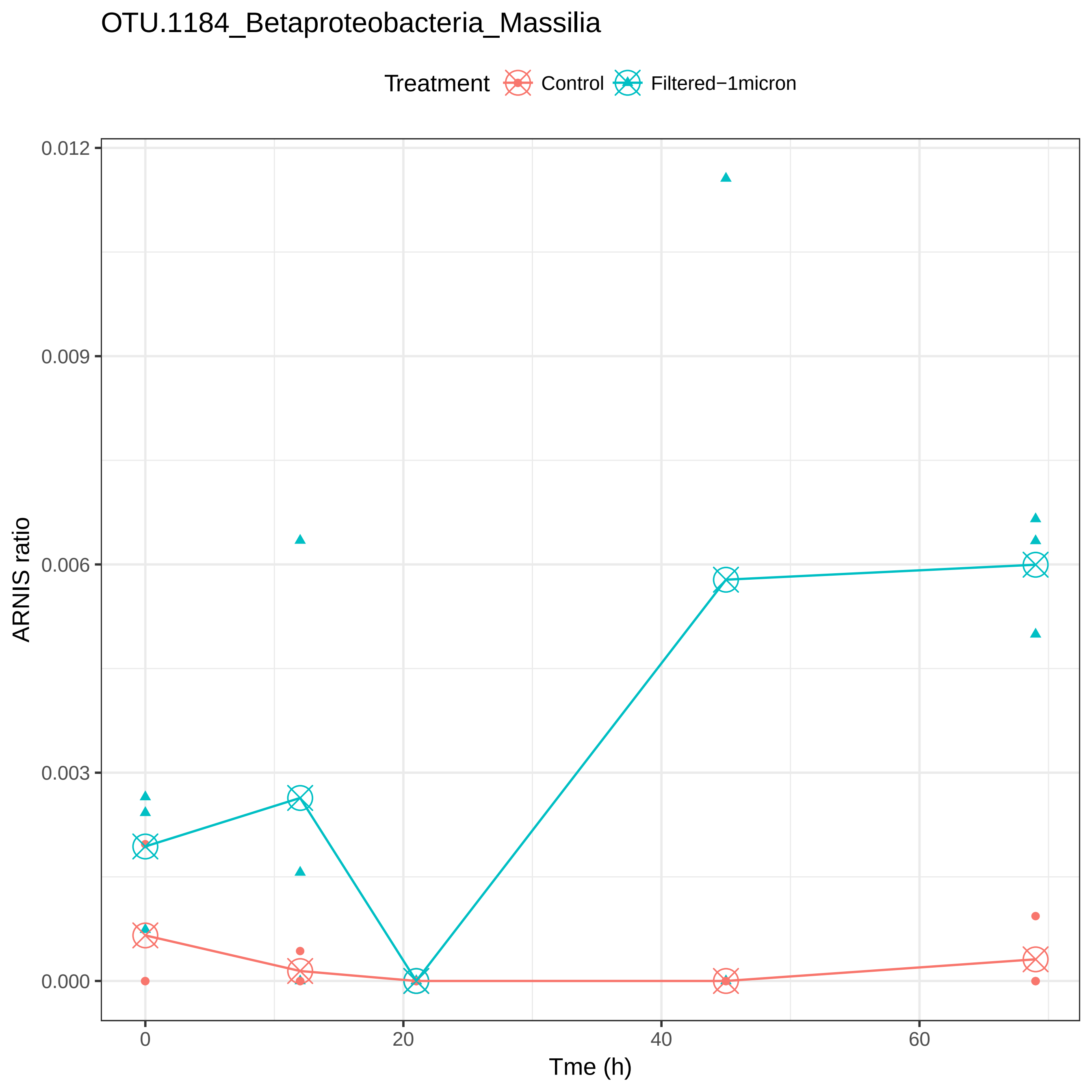
OTU.1184_Betaproteobacteria_Massilia
Treatment
Control
Filtered−1micron
0.012
0.009
0.006
0.003
0.000
ARNIS ratio
0
20
40
60
Tme (h)

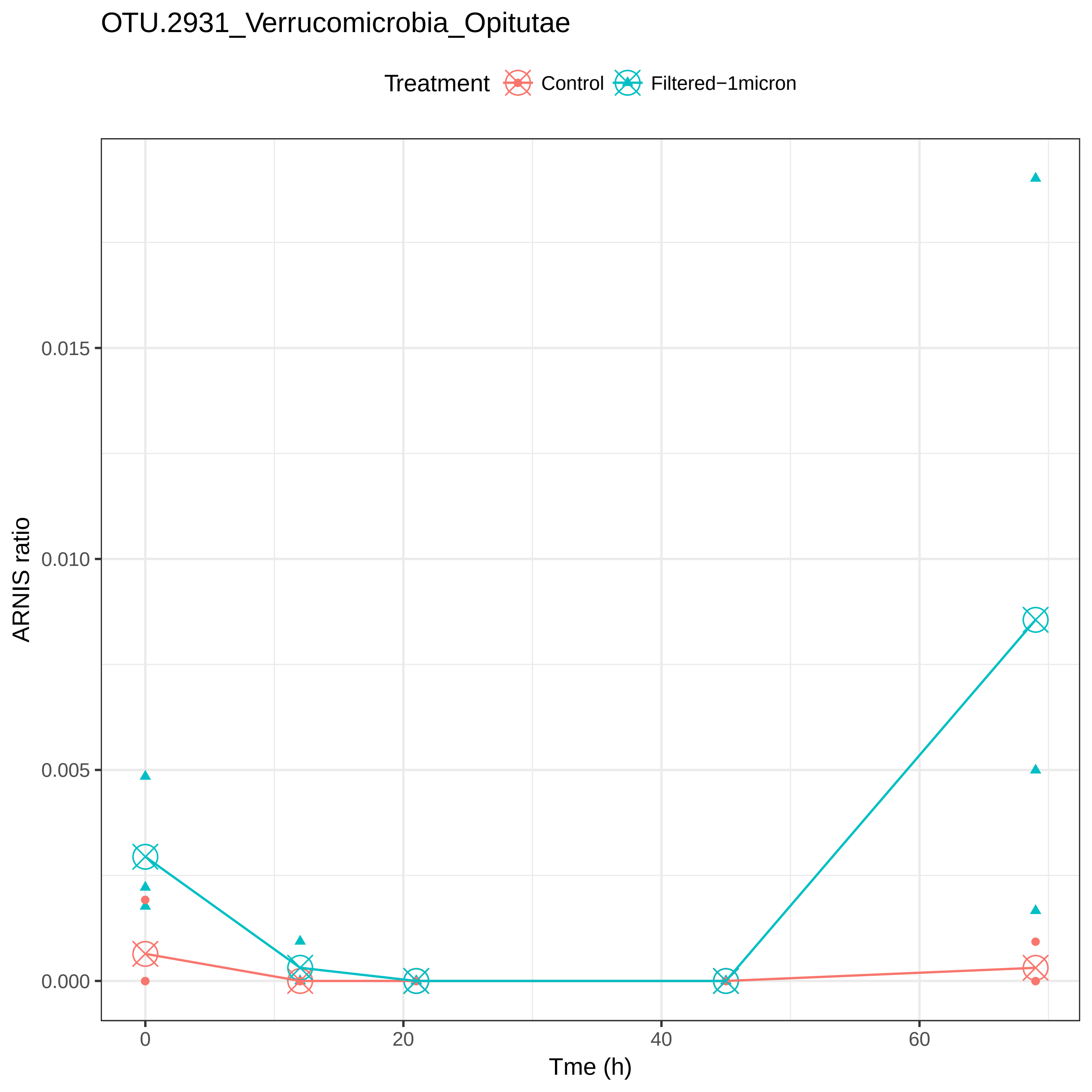

OTU.2931_Verrucomicrobia_Opitutae
Treatment
Control
Filtered−1micron
ARNIS ratio
0.015
0.010
0.005
0.000
0
20
40
60
Tme (h)

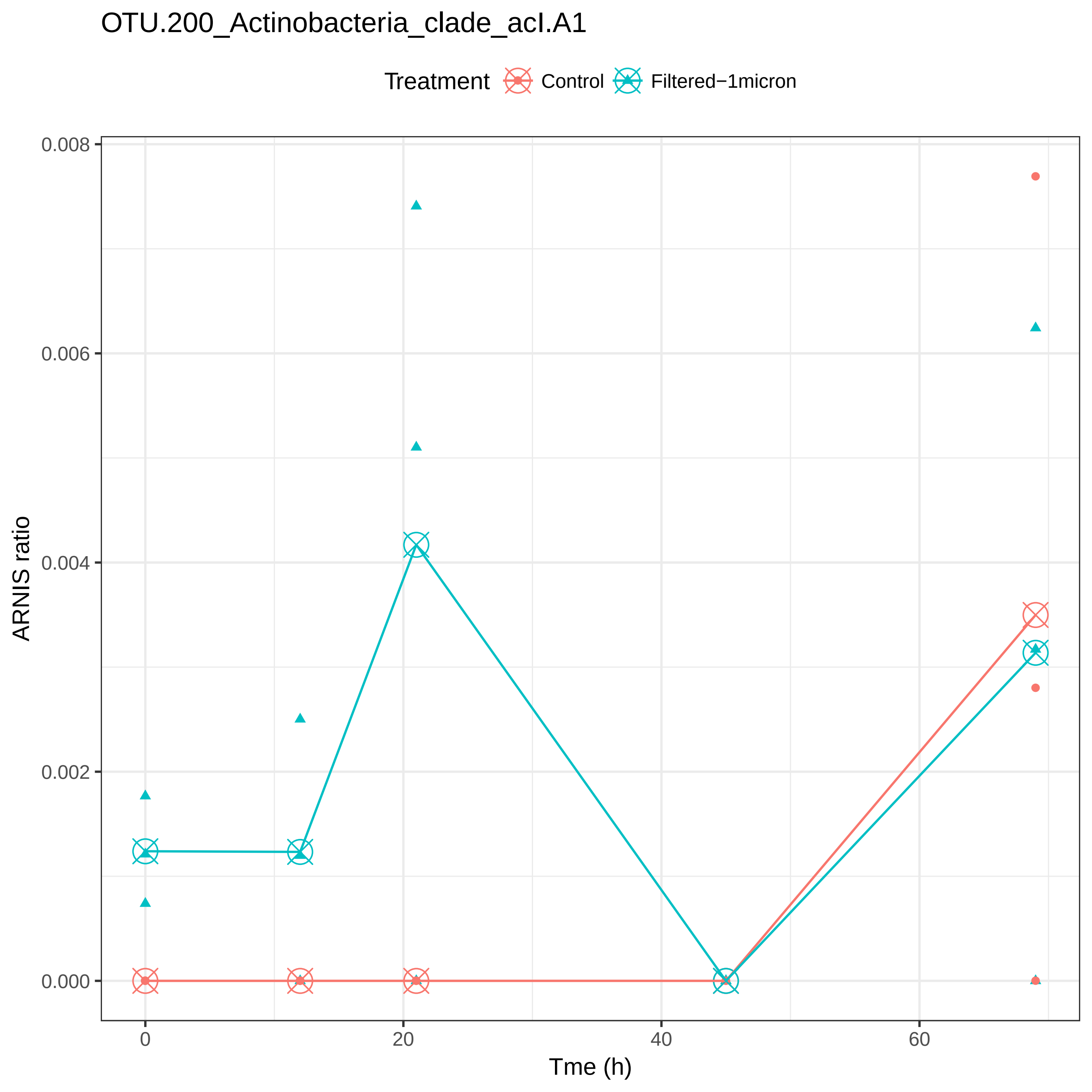

OTU.200_Actinobacteria_clade_acI.A1
Treatment
Control
Filtered−1micron
ARNIS ratio
0.008
0.006
0.004
0.002
0.000
0
20
40
60
Tme (h)

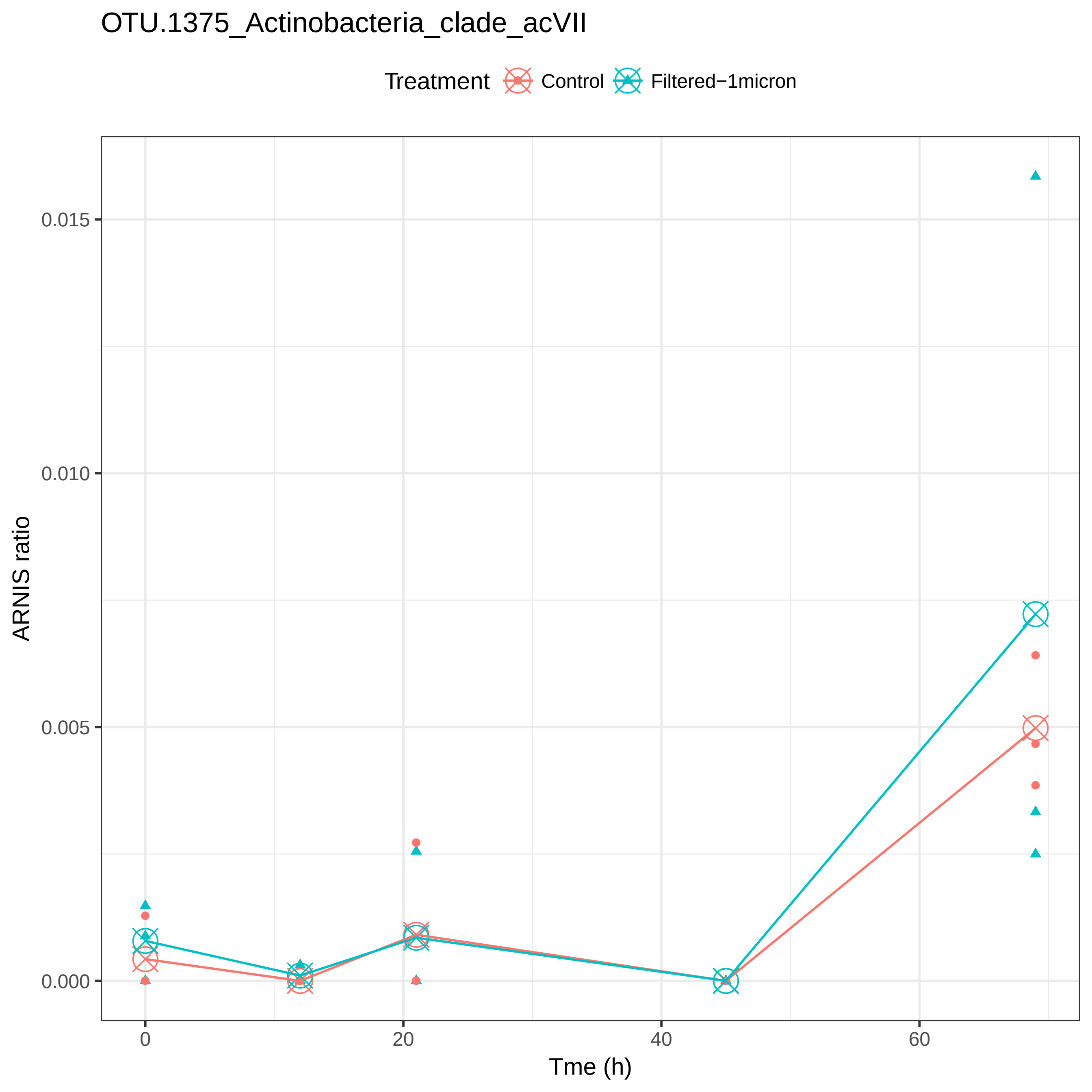
OTU.1375_Actinobacteria_clade_acVII
Treatment
Control
Filtered−1micron
0.015
0.010
0.005
0.000
ARNIS ratio
0
20
40
60
Tme (h)

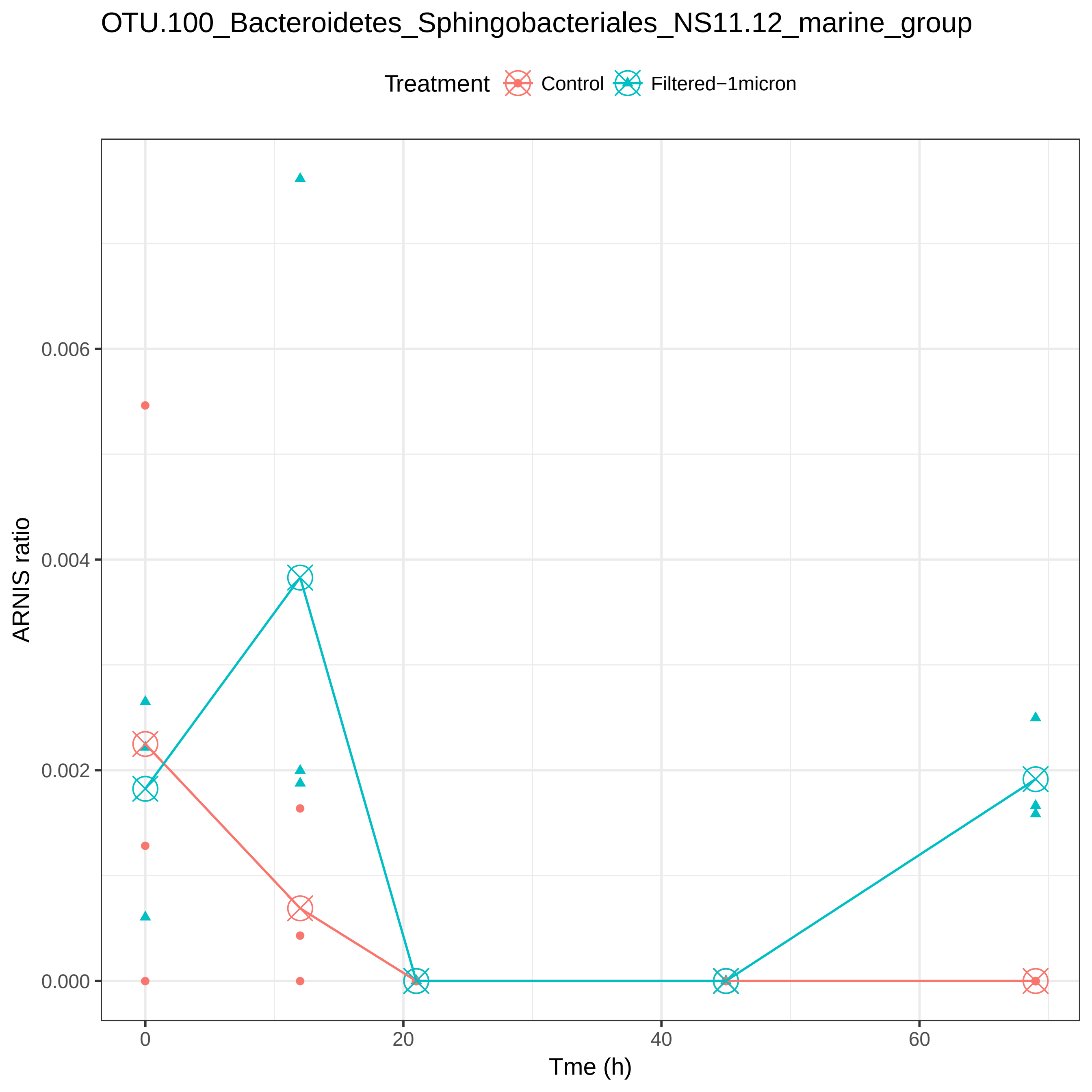

OTU.100_Bacteroidetes_Sphingobacteriales_NS11.12_marine_group
Treatment
Control
Filtered−1micron
ARNIS ratio
0.000
0.002
0.004
0.006
0
20
40
60
Tme (h)

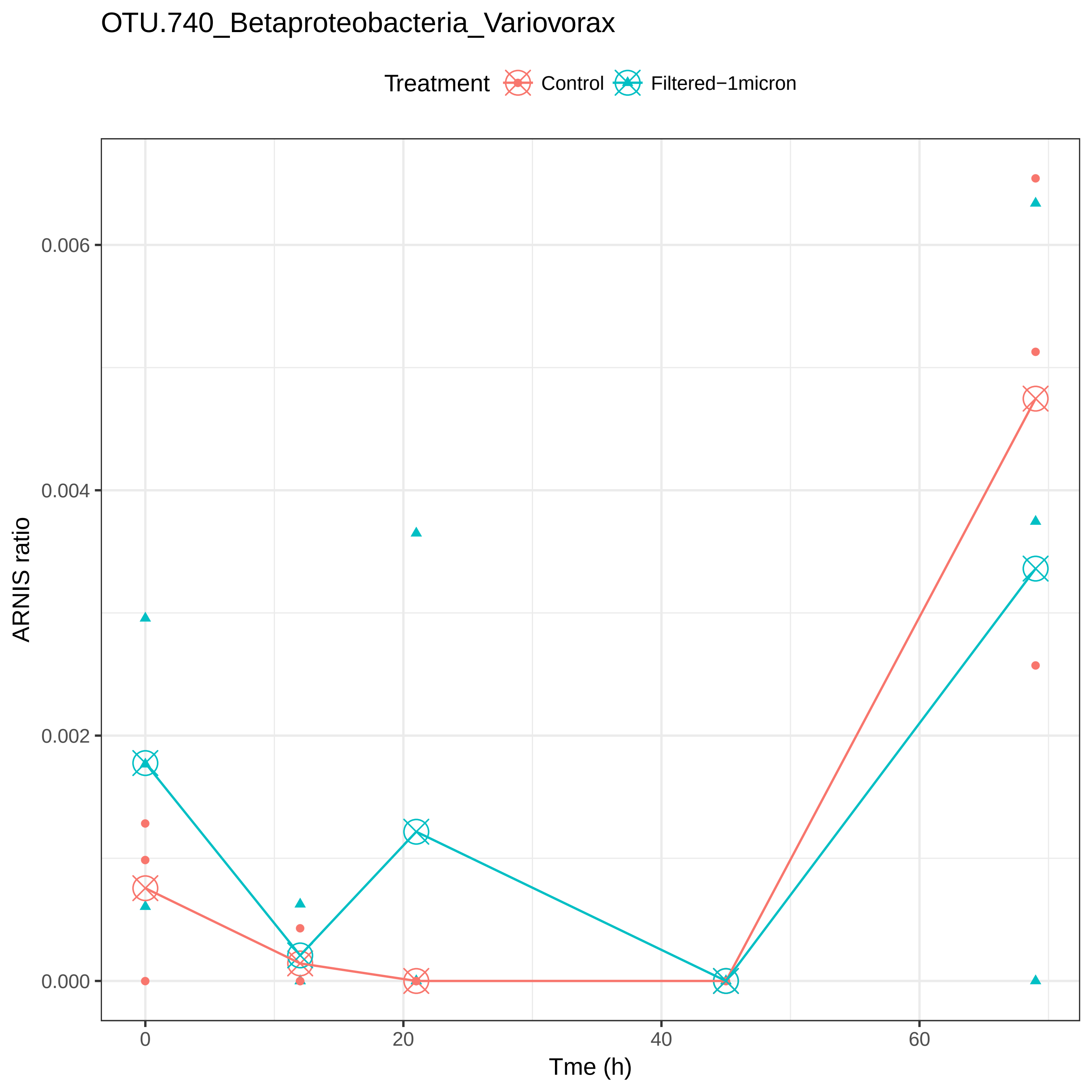

OTU.740_Betaproteobacteria_Variovorax
Treatment
Control
Filtered−1micron
0.006
0.004
0.002
0.000
ARNIS ratio
0
20
40
60
Tme (h)

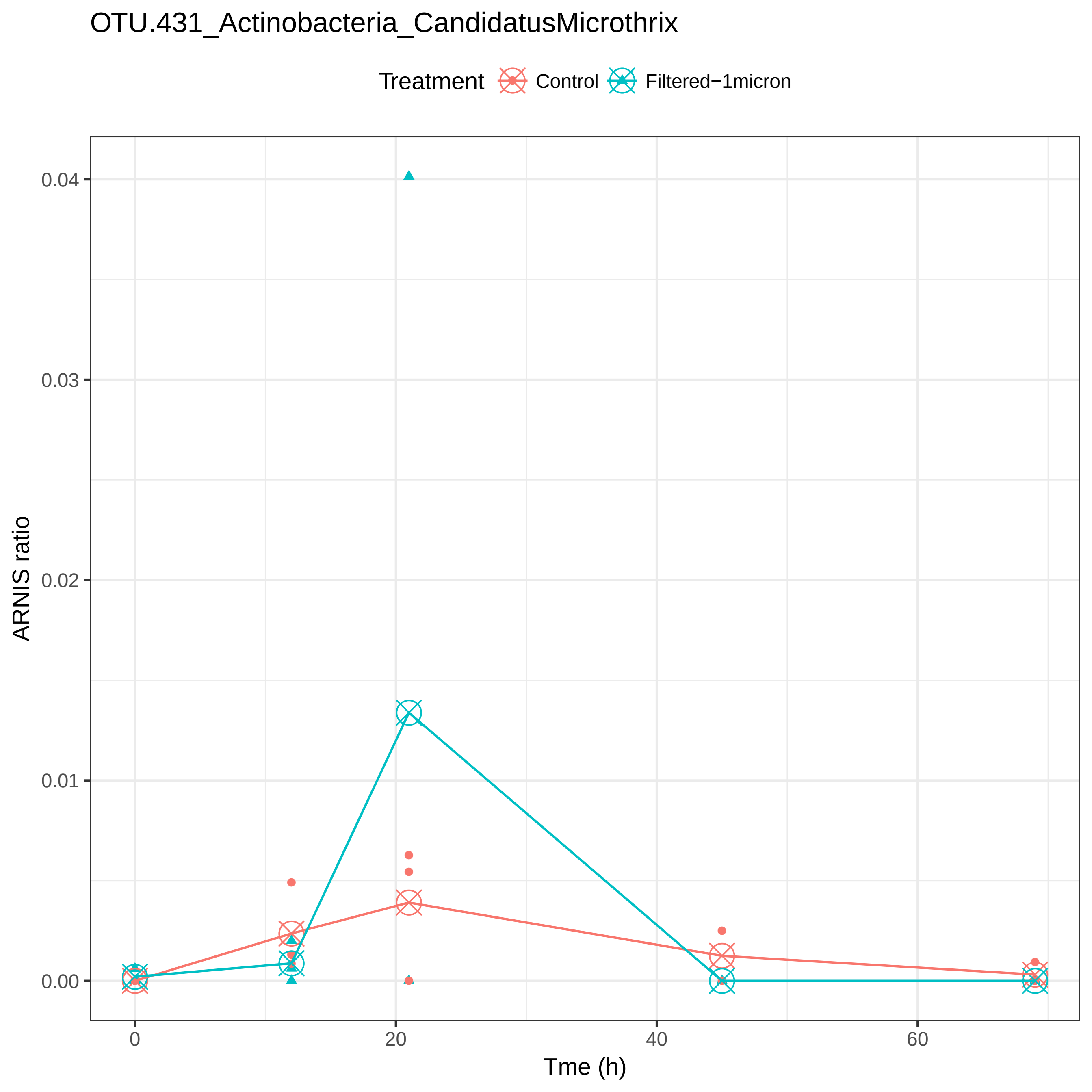
OTU.431_Actinobacteria_CandidatusMicrothrix
Treatment
Control
Filtered−1micron
ARNIS ratio
0.04
0.03
0.02
0.01
0.00
0
20
40
60
Tme (h)

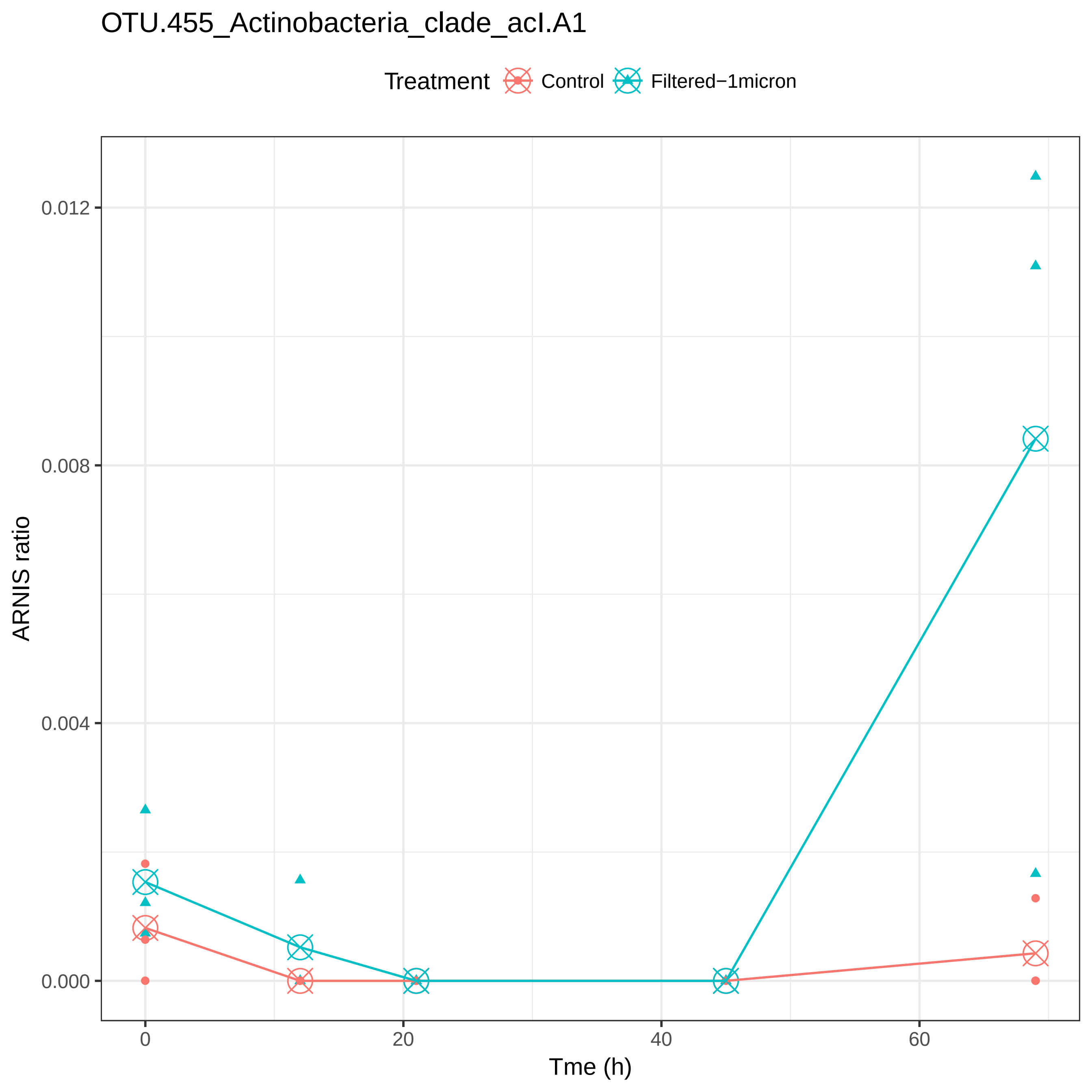

OTU.455_Actinobacteria_clade_acI.A1
Treatment
Control
Filtered−1micron
ARNIS ratio
0.012
0.008
0.004
0.000
0
20
40
60
Tme (h)

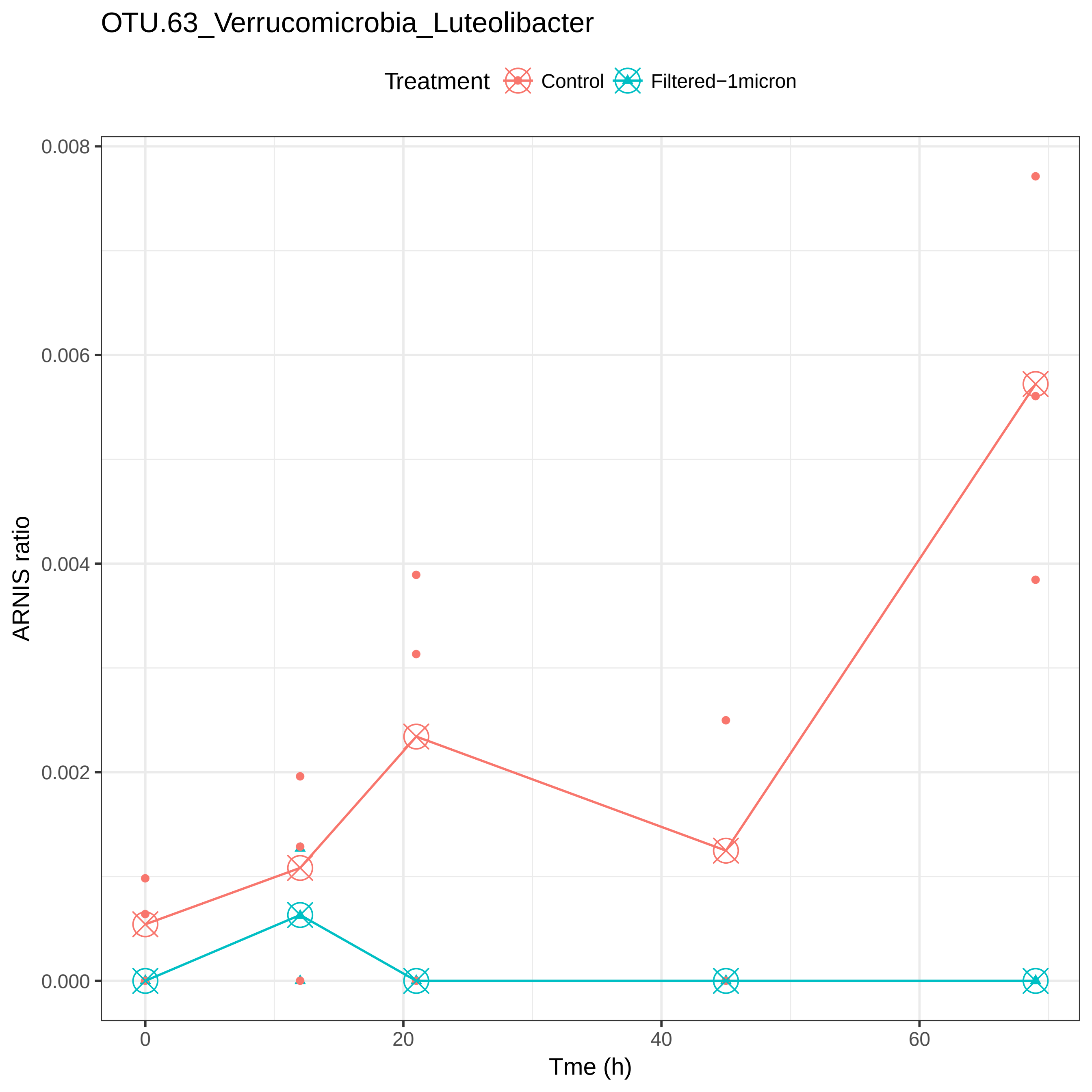

OTU.63_Verrucomicrobia_Luteolibacter
Treatment
Control
Filtered−1micron
ARNIS ratio
0.000
0.002
0.004
0.006
0.008
0
20
40
60
Tme (h)

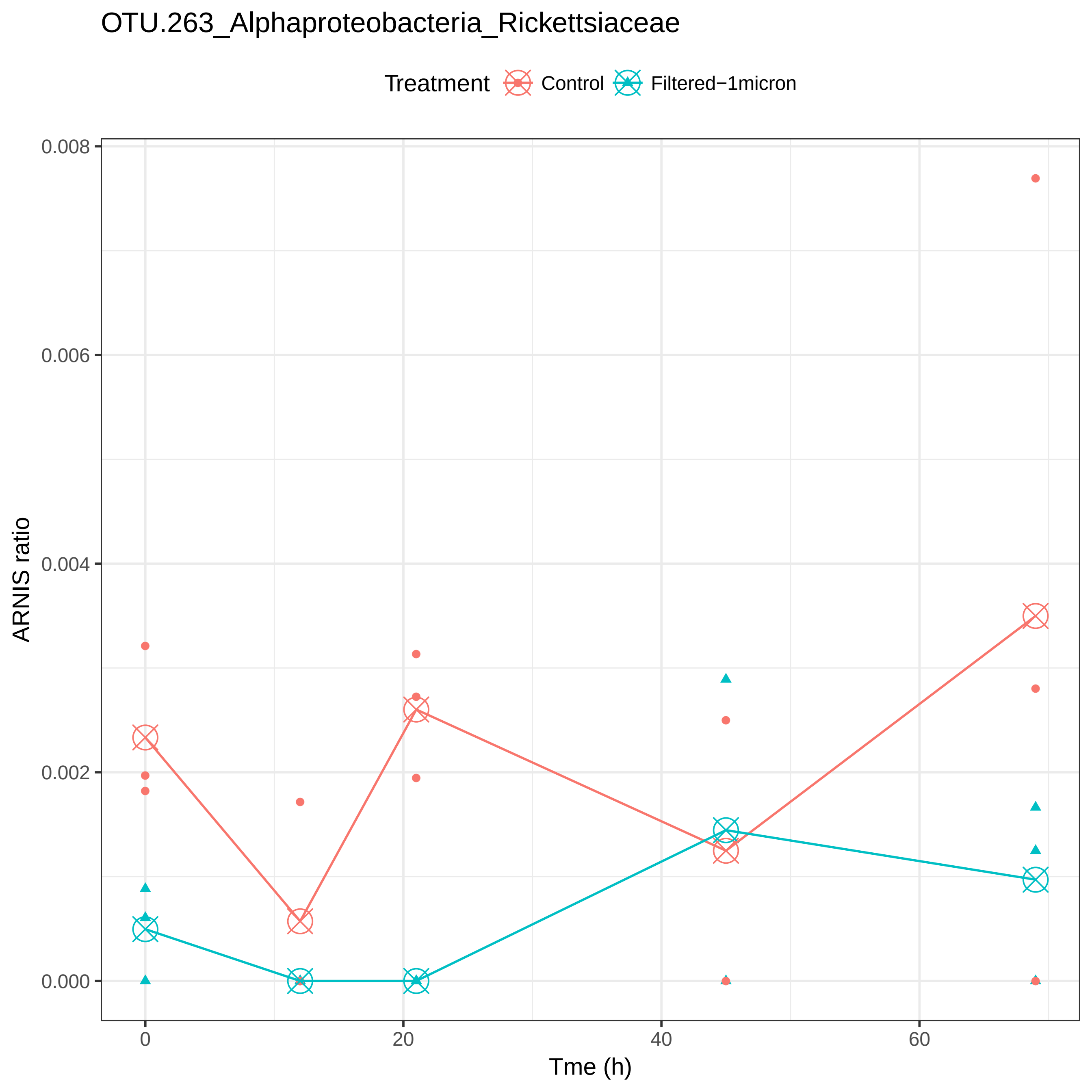
OTU.263_Alphaproteobacteria_Rickettsiaceae
Treatment
Control
Filtered−1micron
ARNIS ratio
0.008
0.006
0.004
0.002
0.000
0
20
40
60
Tme (h)

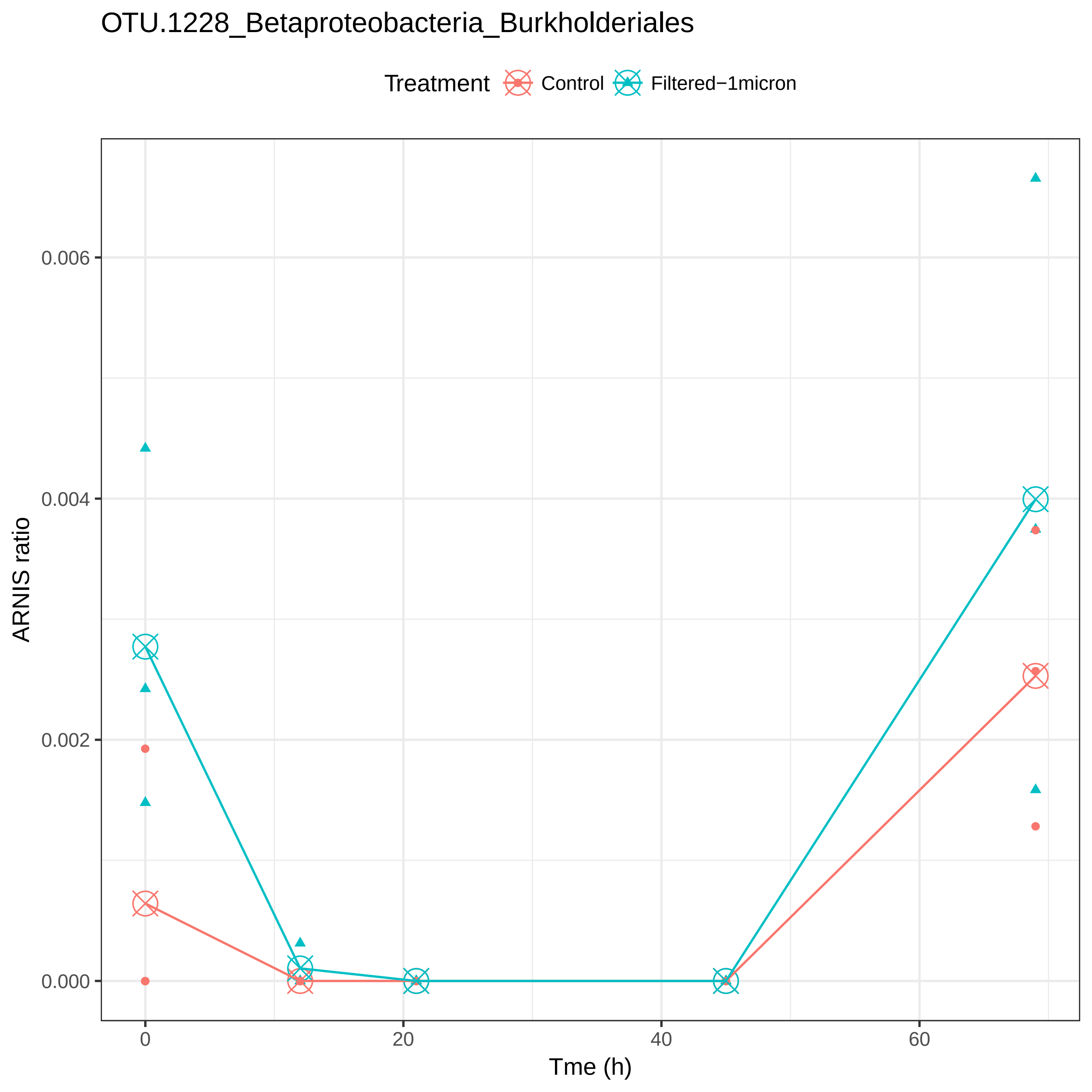

OTU.1228_Betaproteobacteria_Burkholderiales
Treatment
Control
Filtered−1micron
ARNIS ratio
0.006
0.004
0.002
0.000
0
20
40
60
Tme (h)

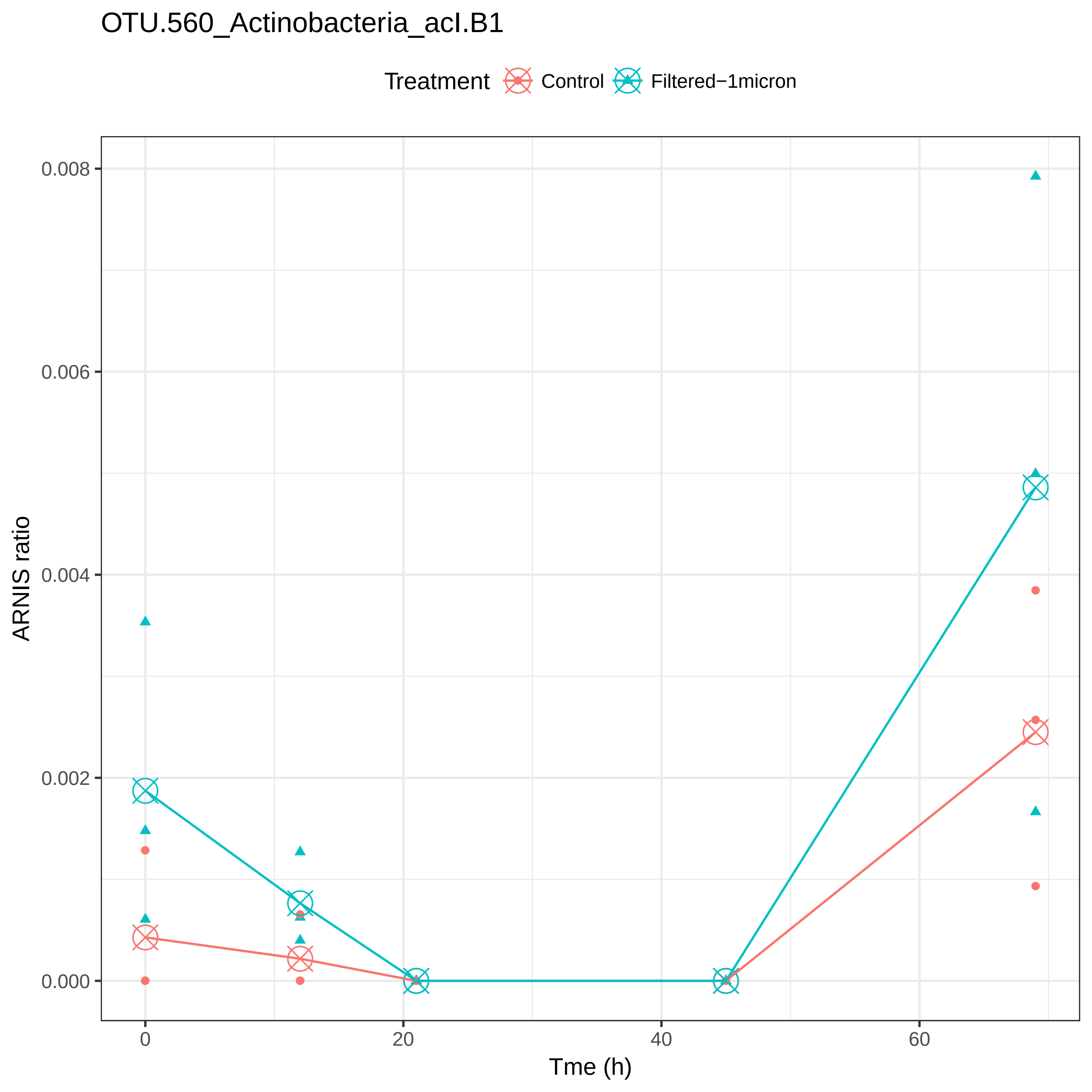

OTU.560_Actinobacteria_acI.B1
Treatment
Control
Filtered−1micron
ARNIS ratio
0.008
0.006
0.004
0.002
0.000
0
20
40
60
Tme (h)

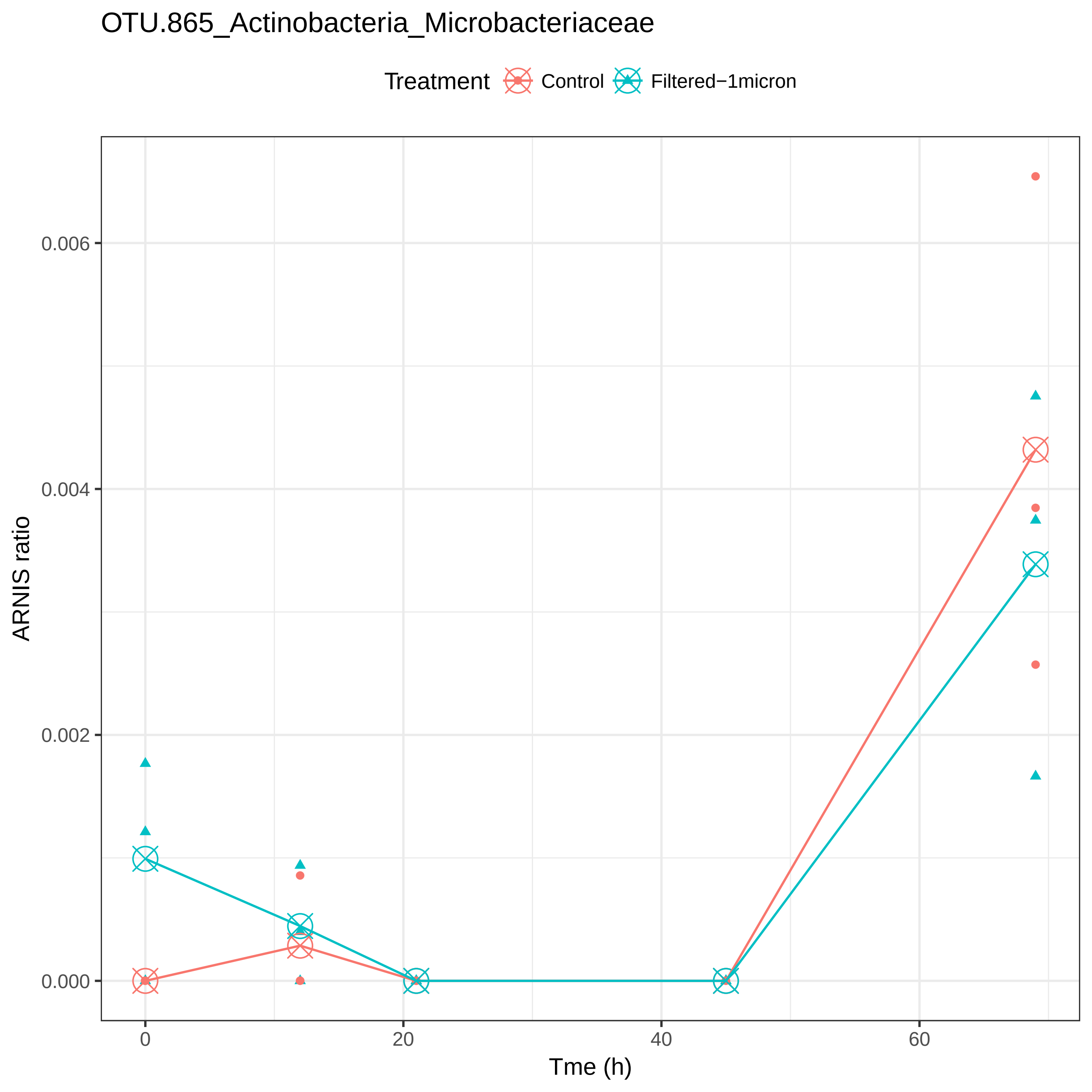

OTU.865_Actinobacteria_Microbacteriaceae
Treatment
Control
Filtered−1micron
ARNIS ratio
0.000
0.002
0.004
0.006
0
20
40
60
Tme (h)

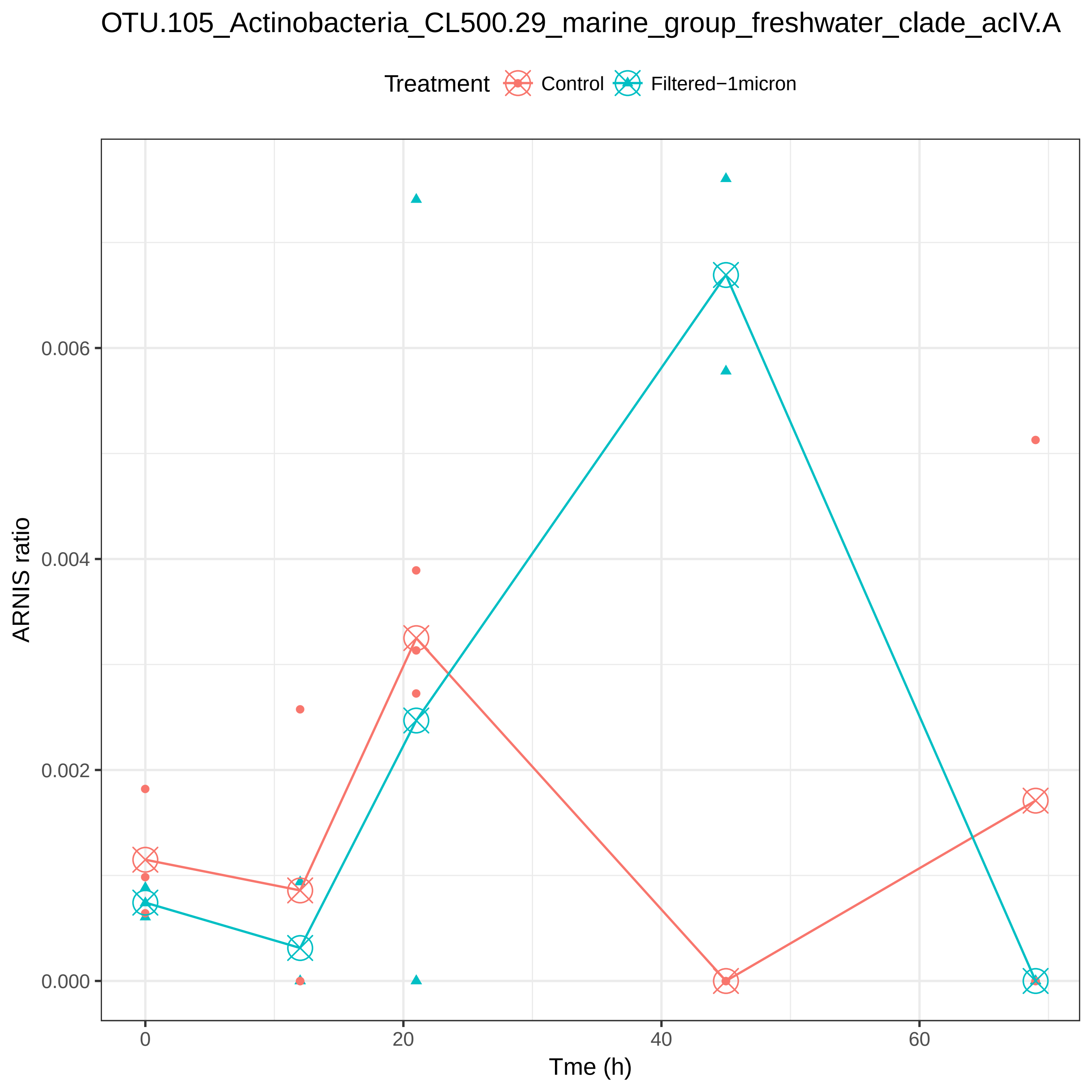
OTU.105_Actinobacteria_CL500.29_marine_group_freshwater_clade_acIV.A
Treatment
Control
Filtered−1micron
ARNIS ratio
0.000
0.002
0.004
0.006
0
20
40
60
Tme (h)

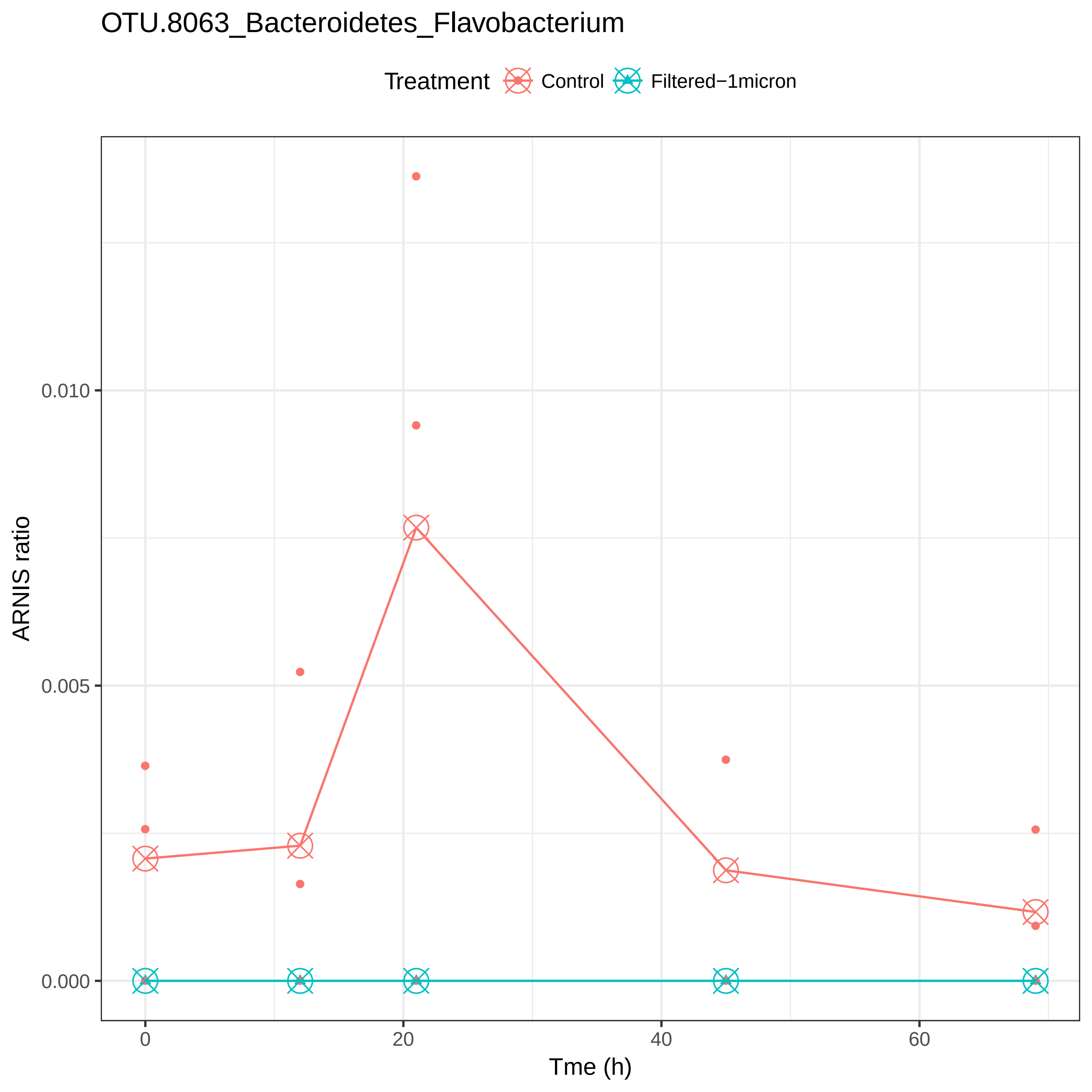
OTU.8063_Bacteroidetes_Flavobacterium
Treatment
Control
Filtered−1micron
ARNIS ratio
0.010
0.005
0.000
0
20
40
60
Tme (h)

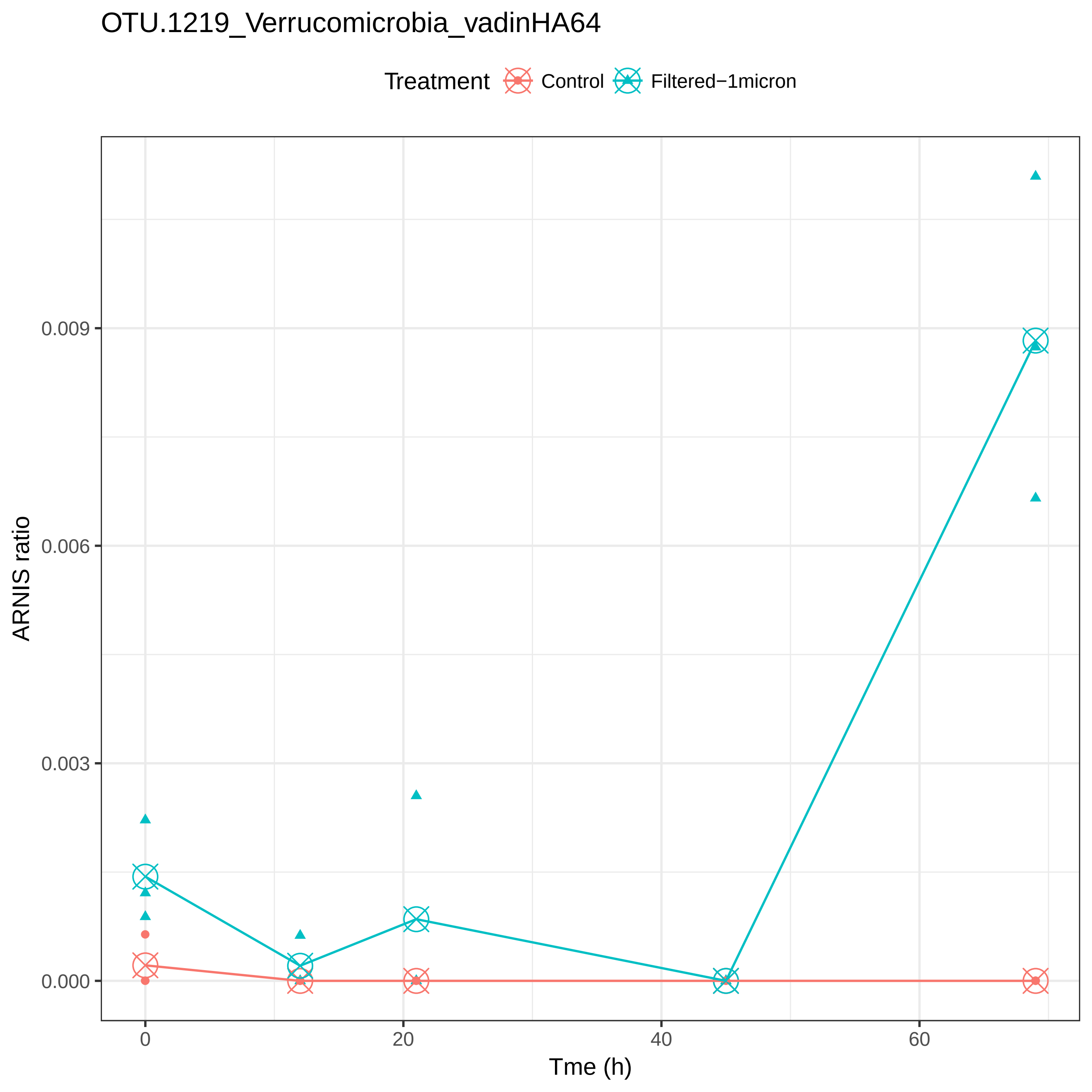

OTU.1219_Verrucomicrobia_vadinHA64
Treatment
Control
Filtered−1micron
0.009
0.006
0.003
0.000
ARNIS ratio
0
20
40
60
Tme (h)

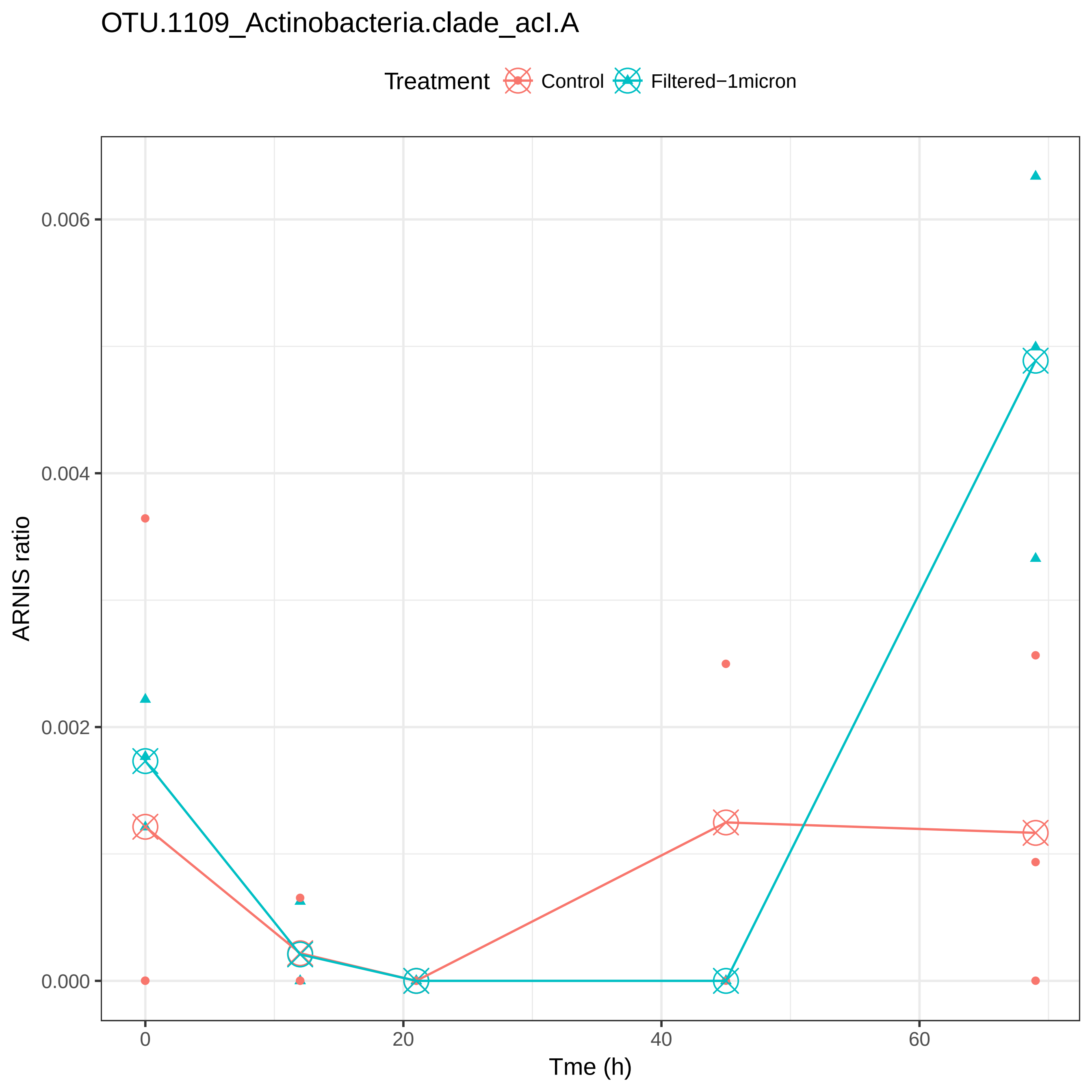
OTU.1109_Actinobacteria.clade_acI.A
Treatment
Control
Filtered−1micron
ARNIS ratio
0.000
0.002
0.004
0.006
0
20
40
60
Tme (h)

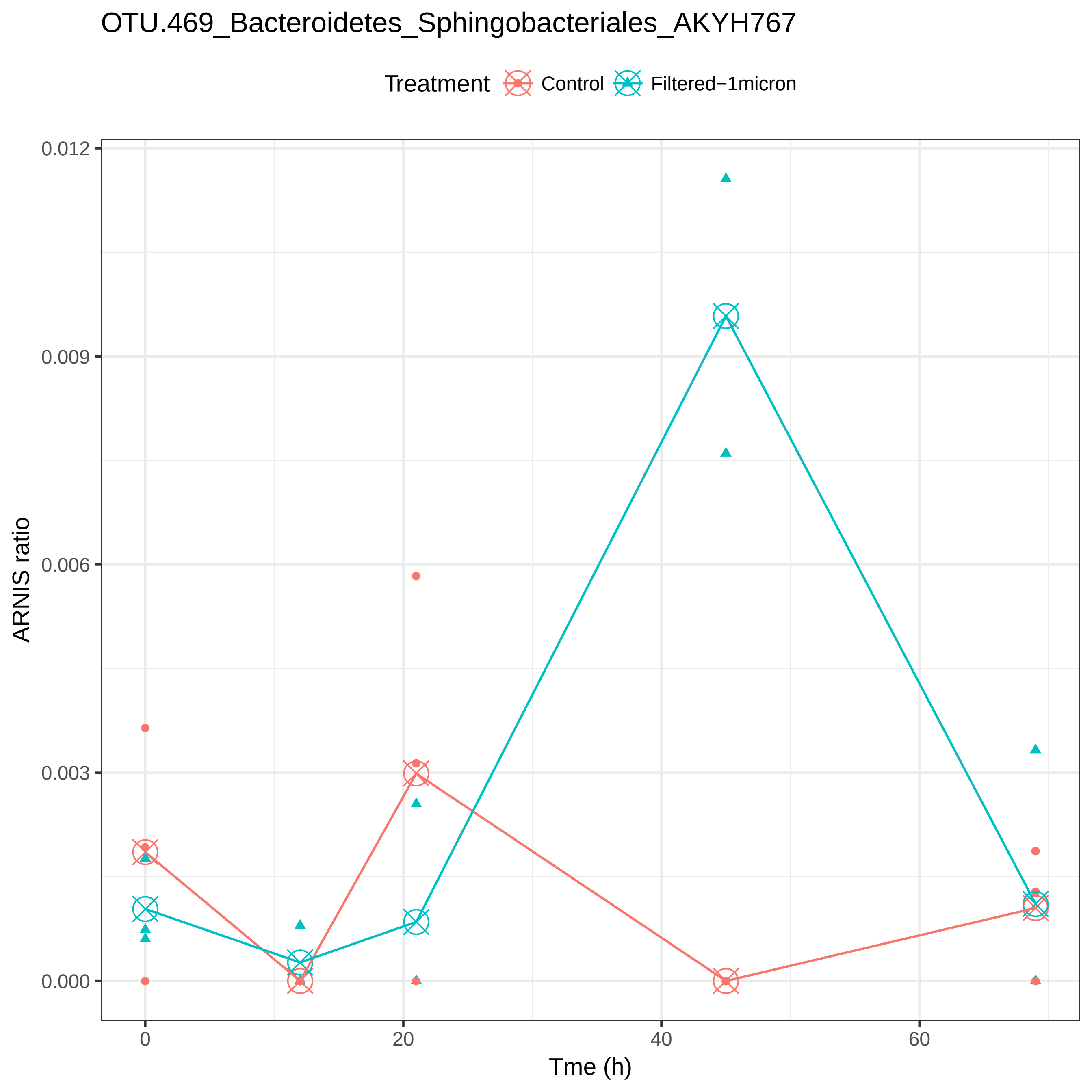
OTU.469_Bacteroidetes_Sphingobacteriales_AKYH767
Treatment
Control
Filtered−1micron
0.012
0.009
0.006
0.003
0.000
ARNIS ratio
0
20
40
60
Tme (h)

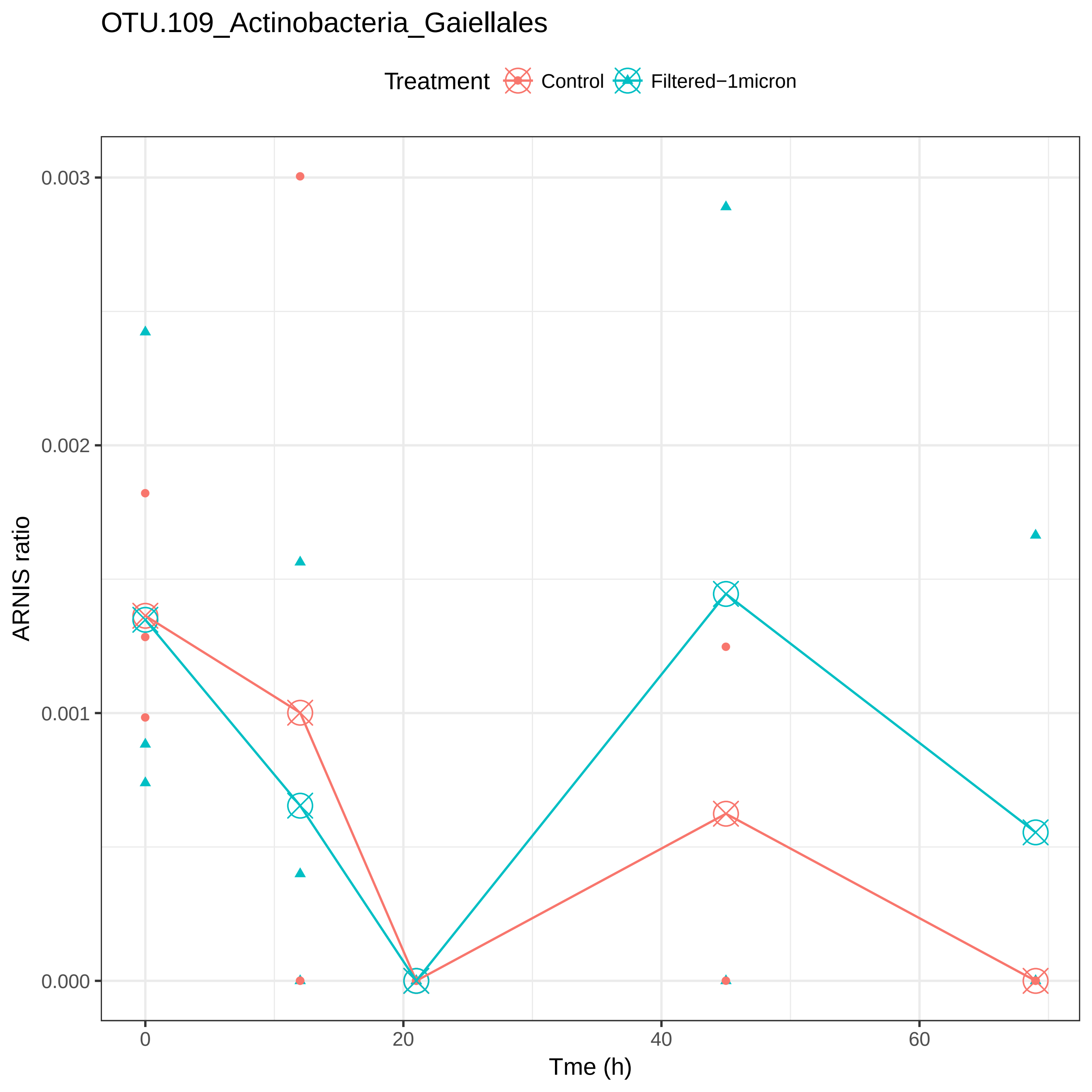
OTU.109_Actinobacteria_Gaiellales
Treatment
Control
Filtered−1micron
0.003
0.002
0.001
0.000
ARNIS ratio
0
20
40
60
Tme (h)

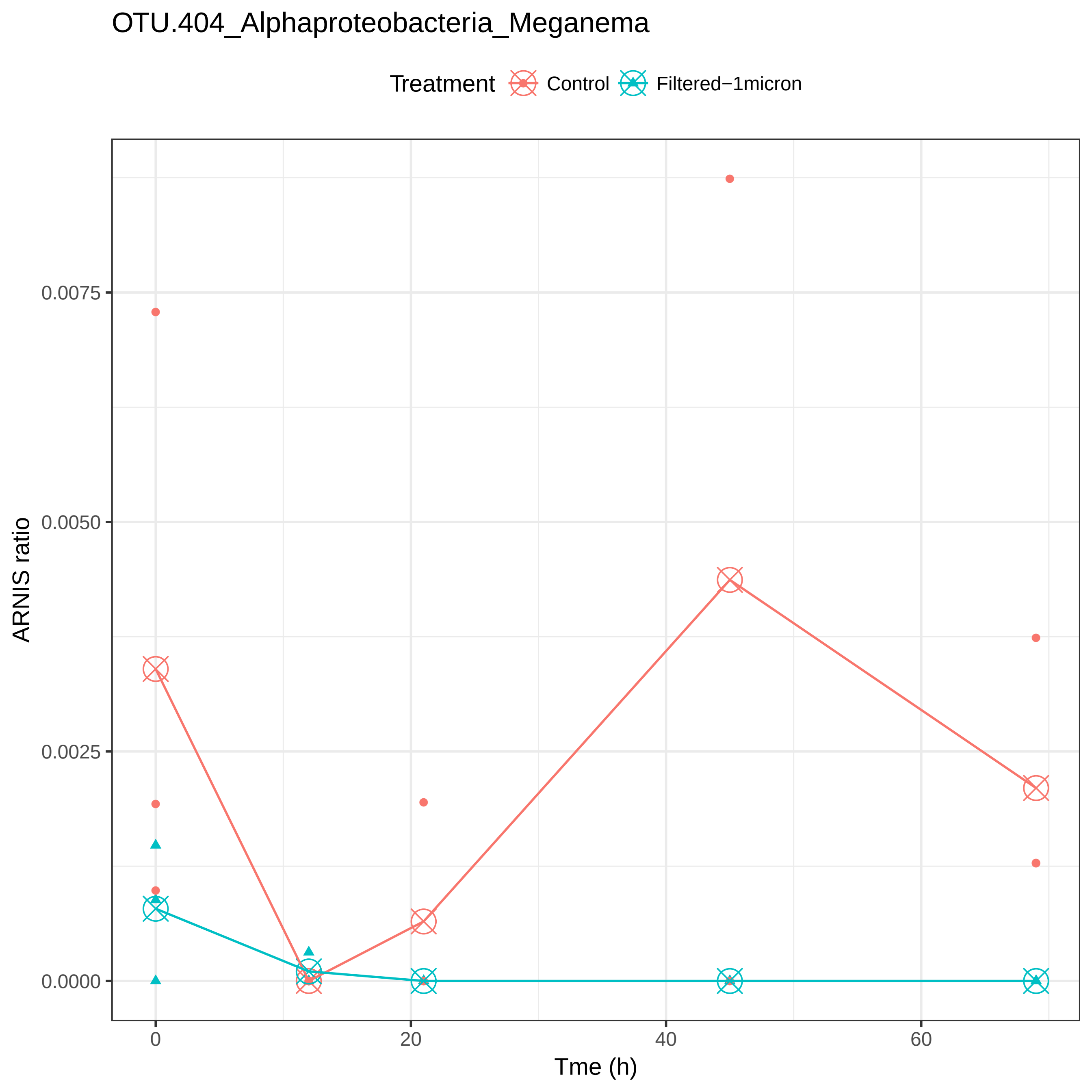

OTU.404_Alphaproteobacteria_Meganema
Treatment
Control
Filtered−1micron
ARNIS ratio
0.0075
0.0050
0.0025
0.0000
0
20
40
60
Tme (h)

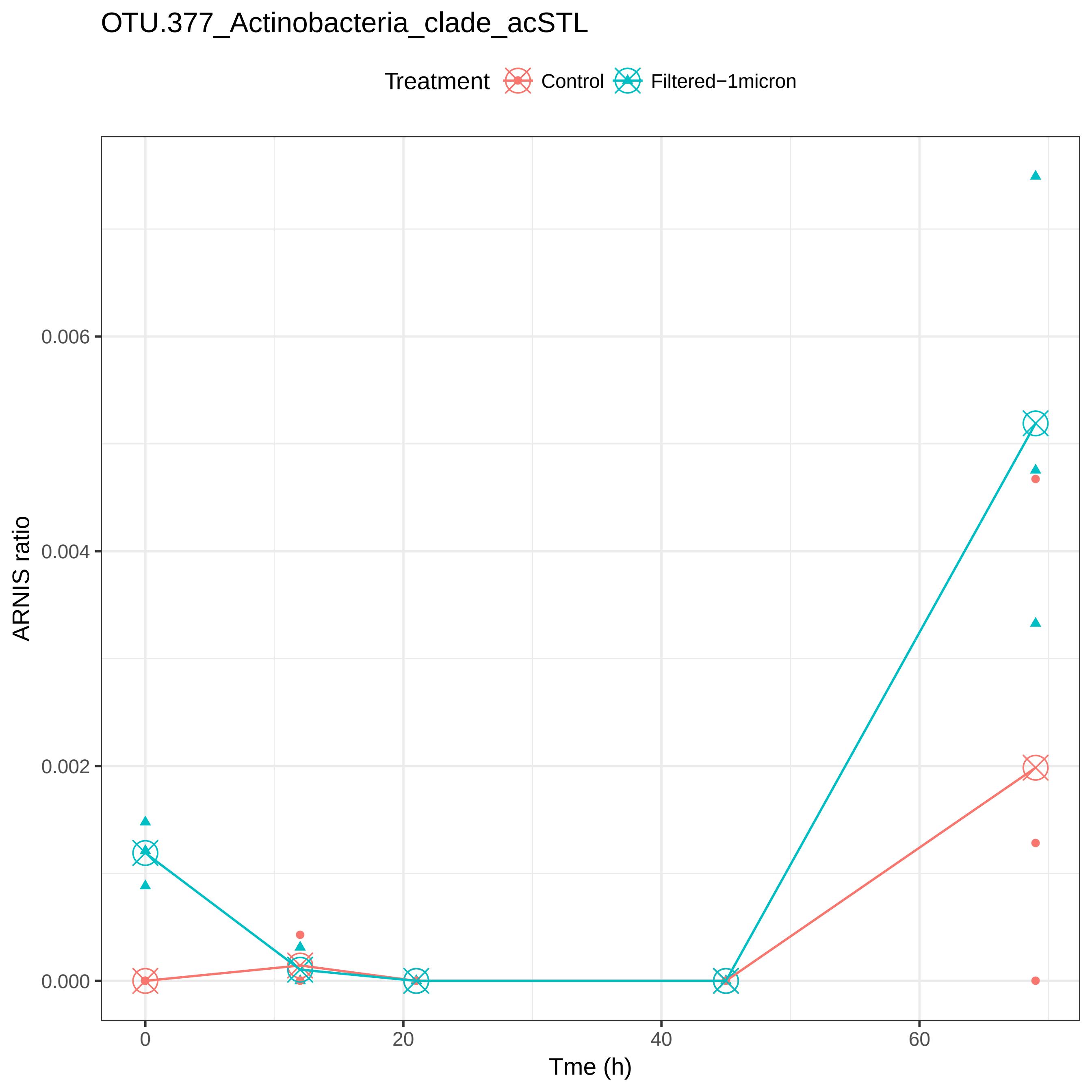
OTU.377_Actinobacteria_clade_acSTL
Treatment
Control
Filtered−1micron
ARNIS ratio
0.000
0.002
0.004
0.006
0
20
40
60
Tme (h)

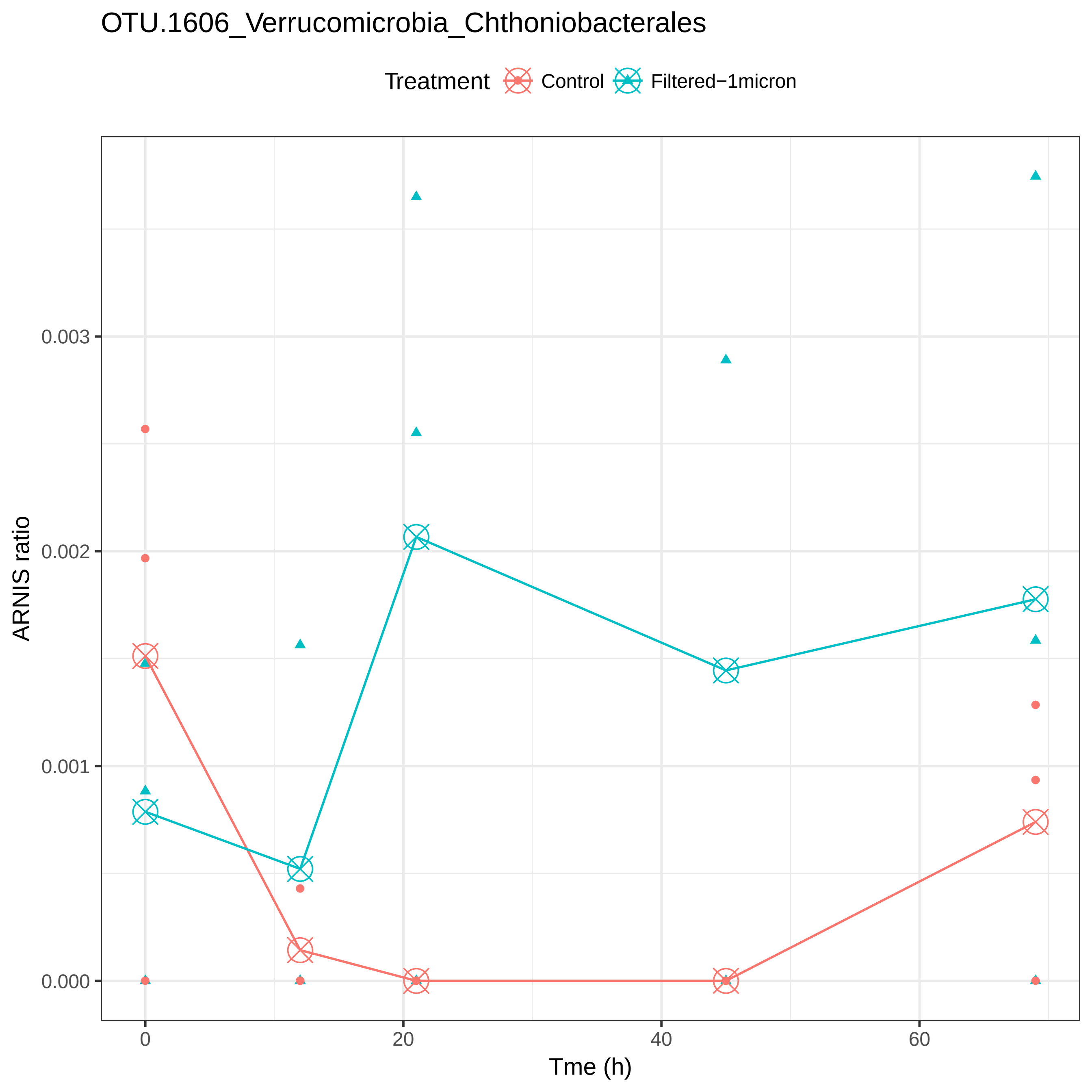

OTU.1606_Verrucomicrobia_Chthoniobacterales
Treatment
Control
Filtered−1micron
ARNIS ratio
0.000
0.001
0.002
0.003
0
20
40
60
Tme (h)

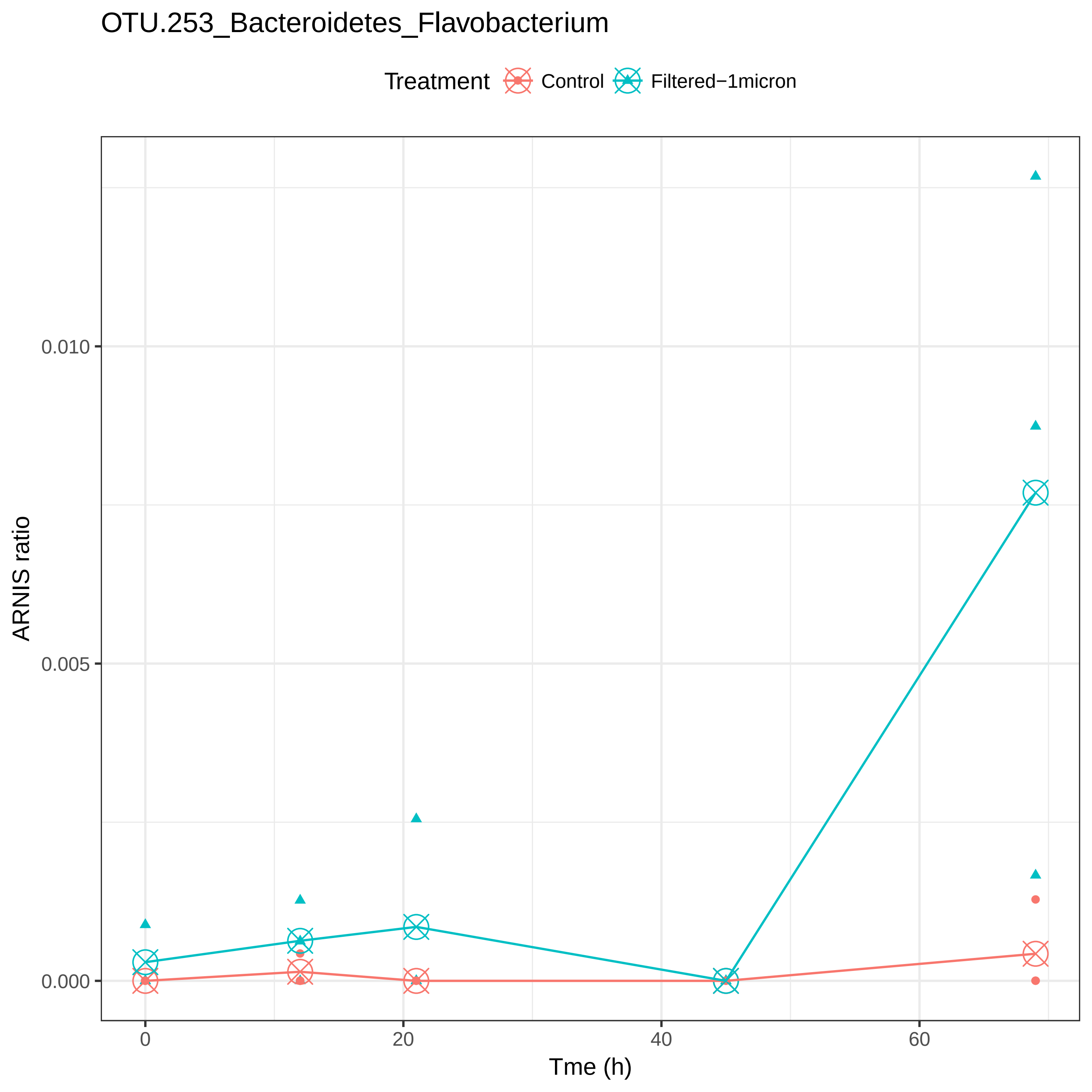

OTU.253_Bacteroidetes_Flavobacterium
Treatment
Control
Filtered−1micron
ARNIS ratio
0.010
0.005
0.000
0
20
40
60
Tme (h)
