# Supplementary material for: Determining lineage-specific bacterial growth curves with a novel approach based on amplicon reads normalization using internal standard (ARNIS)
Source: ISME J. 2018 Jul 6;12(11):2640–54. doi: 10.1038/s41396-018-0213-y (PMC6194029; doi:10.1038/s41396-018-0213-y)
Supplement: Supplementary file 3 — Supplementary Table 1 [file 41396_2018_213_MOESM3_ESM.pdf]

Supplementary Table 1. Expected (Exp.) and actual (Act., from microscopic counts) abundance of species in mock communities (10^5 cells mL^{-1}). Total bacterial abundance from DAPI counts.

**Species**	**Phyllum**	**M1**		**M2**		**M3**		**M4**		**M5**	
	**Class**	**Exp.**	**Act.**	**Exp.**	**Act.**	**Exp.**	**Act.**	**Exp.**	**Act.**	**Exp.**	**Act.**
Rhodoluna lacicola	Actinobacteria Microbacteriaceae	1.5	0.4	2.4	2.0	3.1	1.5	6.5	4.8	4.7	4.9
Sphingomonas sp. AAP5	Proteobacteria Alphaproteobacteria	3.4	2.6	3.3	2.7	1.4	1.0	17.6	16.9	18.2	14.8
Flavobacterium sp.	Bacteroidetes Flavobacteriia	0.08	0.8	0.08	3.7	0.7	1.6	8.8	11.1	22.7	20.5
Gemmatimonas phototrophica	Gemmaminonadetes	0.05	0.1	1.1	2.5	4.7	2.5	17.2	18.0	7.8	8.7
Total bacterial abundance		5.1	3.9	6.9	11.1	9.8	6.7	50.0	50.8	53.4	48.9
