# Supplementary material for: Determining lineage-specific bacterial growth curves with a novel approach based on amplicon reads normalization using internal standard (ARNIS)
Source: ISME J. 2018 Jul 6;12(11):2640–54. doi: 10.1038/s41396-018-0213-y (PMC6194029; doi:10.1038/s41396-018-0213-y)
Supplement: Supplementary file 4 — Supplementary Table 2 [file 41396_2018_213_MOESM4_ESM.pdf]

**Supplementary Table 2**. The list and sequences of CARD-FISH probes, competitors and helpers (H) used to verify the ARNIS approach. FA% – concentration of formamid in the hybridization buffer (at 37$^{o}$C).

| **Probe** | **Target Group** | **Probe Sequence (5'-3')** | **Competitor/Helper Sequence (5'-3')** | **FA%** | **Reference** |
|---|---|---|---|---|---|
| HGC69a | Actinobacteria | TATAGTTACCACCGCCGT | none | 30 | Roller et al 1994 |
| Ac1-852 | acI lineage of Actinibacteria | AATGCGTTAGCTGCGTCGCA | H1: AAACCGTGGAAGGTYCSCACAACTAG<br>H2: TCCCCAGGCGGGGCRCTT | 55 | Warnecke et al 2005 |
| ALF968 | Alphaproteobacteria | GGTAAGGTTCTGCGCGTT | none | 55 | Neef 1997 |
| CF968 | Bacteroidetes | GGTAAGGTTCCTCGCGTA | none | 55 | Acinas et al 2014 |
| BET42a | Betaproteobacteria | GCCTTCCCACTTCGTTT | Competitor: GCCTTCCCACATCGTTT | 55 | Manz et al 1992 |
| CF319a | Cytophaga-Flavobacteria | TGGTCCGTGTCTCAGTAC | none | 55 | Manz et al 1996 |
| R-BT065 | *Limnohabitans* spp. | GTTGCCCCCTCTACCGTT | none | 55 | Šimek et al 2001 |

## References


Acinas SG, Ferrera I, Sarmento H, Diez-Vives C, Forn I, Ruiz-Gonzalez C et al (2015). Validation of a new catalysed reporter deposition-fluorescence in situ hybridization probe for the accurate quantification of marine Bacteroidetes populations. Environ Microbiol 17: 3557-3569.

Manz W, Amann R, Ludwig W, Wagner M, Schleifer KH (1992). Phylogenetic Oligodeoxynucleotide Probes for the Major Subclasses of Proteobacteria - Problems and Solutions. Syst Appl Microbiol 15: 593-600.

Manz W, Amann R, Ludwig W, Vancanneyt M, Schleifer KH (1996). Application of a suite of 16S rRNA-specific oligonucleotide probes designed to investigate bacteria of the phylum cytophaga-flavobacter-bacteroides in the natural environment. Microbiology 142: 1097-1106.

Neef A (1997). Anwendung der in situ Einzelzell-Identifizierung von Bakterien zur Populationsanalyse in komplexen mikrobiellen Biozönosen, Technische Universität München.

Roller C, Wagner M, Amann R, Ludwig W, Schleifer KH (1994). In situ probing of Gram-positive bacteria with high DNA G+C content using 23S rRNA-targeted oligonucleotides. Microbiology 140: 2849-2858.

Šimek K, Pernthaler J, Weinbauer MG, Horňák K, Dolan JR, Nedoma J et al (2001). Changes in bacterial community composition and dynamics and viral mortality rates associated with enhanced flagellate grazing in a mesoeutrophic reservoir. Appl Environ Microbiol 67: 2723-2733.

Warnecke F, Sommaruga R, Sekar R, Hofer JS, Pernthaler J (2005). Abundances, identity, and growth state of actinobacteria in mountain lakes of different UV transparency. Appl Environ Microbiol 71: 5551-5559.
